# Inference and analysis of cell‐cell communication of non‐myeloid circulating cells in late sepsis based on single‐cell RNA‐seq

**DOI:** 10.1049/syb2.12109

**Published:** 2024-11-22

**Authors:** Yanyan Tao, Miaomiao Li, Cheng Liu

**Affiliations:** ^1^ Department of Emergency Medicine The First Affiliated Hospital of Bengbu Medical University Bengbu Anhui China; ^2^ Institute of Critical Care Medicine The First Affiliated Hospital of Bengbu Medical College Bengbu Anhui China; ^3^ Department of Critical Care Medicine The First Affiliated Hospital of Bengbu Medical University Bengbu Anhui China

**Keywords:** bioinformatics, biology computing, data analysis

## Abstract

Sepsis is a severe systemic inflammatory syndrome triggered by infection and is a leading cause of morbidity and mortality in intensive care units (ICUs). Immune dysfunction is a hallmark of sepsis. In this study, the authors investigated cell‐cell communication among lymphoid‐derived leucocytes using single‐cell RNA sequencing (scRNA‐seq) to gain a deeper understanding of the underlying mechanisms in late‐stage sepsis. The authors’ findings revealed that both the number and strength of cellular interactions were elevated in septic patients compared to healthy individuals, with several pathways showing significant alterations, particularly in conventional dendritic cells (cDCs) and plasmacytoid dendritic cells (pDCs). Notably, pathways such as CD6‐ALCAM were more activated in sepsis, potentially due to T cell suppression. This study offers new insights into the mechanisms of immunosuppression and provides potential avenues for clinical intervention in sepsis.

## Graphical abstract

In this study, the authors analysed the cell‐cell communication in lymphoid‐derived leucocytes using single‐cell transcriptomics sequencing (scRNA‐seq) to better understand the mechanisms in late sepsis.

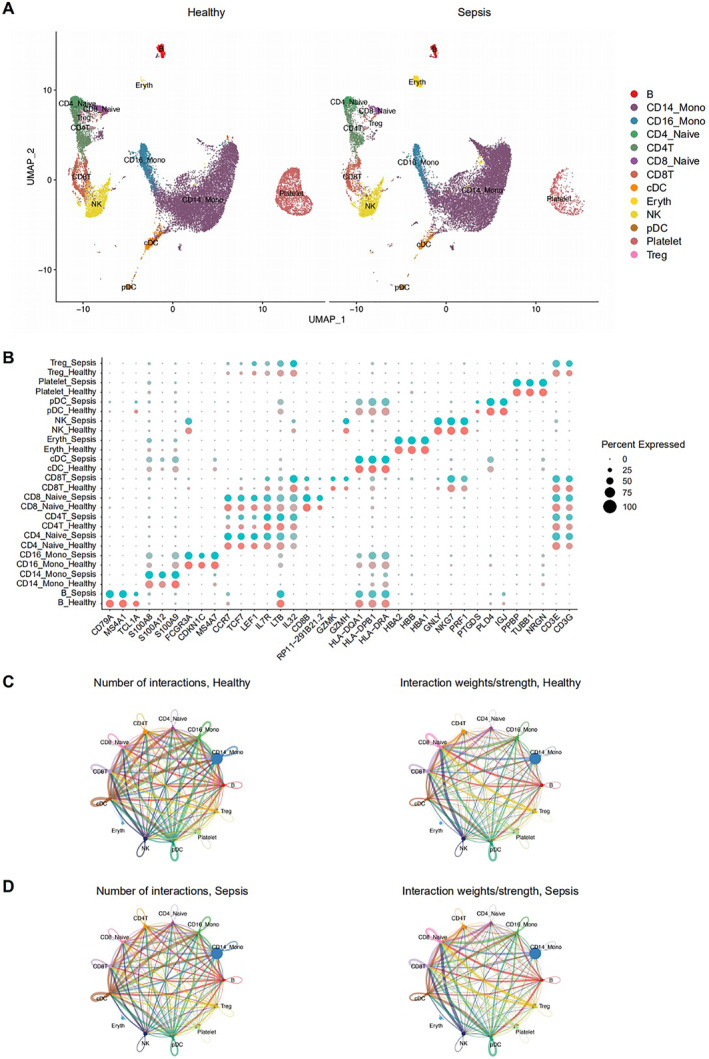

## 1 INTRODUCTION

Sepsis is a life‐threatening condition caused by a dysregulated host response to infection [1], and remains one of the leading causes of death in intensive care units (ICUs) [2, 3, 4, 5]. Despite advancements in diagnostic tools and clinical treatments that have reduced acute mortality, sepsis continues to impose significant economic and societal burdens, with its incidence rising globally each year [6, 7, 8]. The pathogenesis of sepsis remains poorly understood, with immunosuppression emerging as a major driver of morbidity [7, 9]. Both innate and adaptive immune cells exhibit long‐term dysfunction after clinical treatment, yet the detailed mechanisms governing inflammation and immune responses in sepsis are not fully elucidated. Given the lack of effective interventions to mitigate immune system dysfunction and improve survival rates, there is an urgent need for a deeper understanding of the basic mechanisms underlying sepsis.

Single‐cell RNA sequencing (scRNA‐seq) has proven to be an invaluable tool for identifying previously unrecognised cell types and characterising their gene expression profiles [10, 11, 12, 13, 14, 15, 16, 17]. Recently, this technology has been employed to investigate the pathogenesis of sepsis [18, 19]. Advances in transcriptomic sequencing have revealed alterations in the expression profiles of immune cells during sepsis [20, 21, 22]. However, most studies on immunosuppression have focused on specific immune cell types and their transcriptomic signatures, leaving the broader landscape of cellular interactions in sepsis largely unexplored [23, 24, 25]. In this study, we performed a comprehensive analysis of cell‐cell communication among non‐myeloid circulating cells in late‐stage sepsis using scRNA‐seq, aiming to uncover potential mechanisms driving immunosuppression.

## 2 DATASEST AND METHOD

### 2.1 Data collection and pre‐processing

scRNA‐seq profiles were obtained from the GEO (https://www.ncbi.nlm.nih.gov/geo/query/acc.cgi?acc=GSE175453) involving four septic patients (sepsis) and five normal individuals (healthy control) [18]. The cell counts of the four patients with sepsis were 958, 3848, 7962 and 5854, respectively, and five normal individuals were 1216, 769, 9143, 5111 and 6679, respectively. The UMI of 10X scRNA‐seq data was normalised by the function LogNormalize () in Seurat (v4.0.3) [26], and the following analysis is based on the expression matrix in TPM format.

### 2.2 Integration analysis and cell identification

To eliminate the batch effect between different patients and normal individuals [27], the function IntegrateData () of Seurat (v 4.0.3) was used for integration analysis. After integration, top 2000 highly variable genes were selected to perform principal component analysis (PCA) analysis and top 30 principal components were applied for uniform manifold approximation and projection (UMAP) analysis. Then, function FindCluster () was used to cluster the cells. The cell identity was defined by the Azimuth (v 0.4.3) algorithm which could infer the cell identity by integrating the research data with published highly credible data by cell‐to‐cell similarity.

### 2.3 Differential expression analysis

With the results of the Seurat analysis described above, the function FindAllMarkers () of Seurat was used to calculate differential genes for different cell types in Sepsis and Healthy data, respectively (P adj <0.05, |log2 Fold change| > 0.25). The differential expression genes of each cell type Top3 in the form of bubble plots was analysed by ggplot2 (v 3.3.5).

### 2.4 Cell‐cell communication analysis

The cellular communications between different cell types were analysed using CellChat (v 1.5.0) [28], and the labels of the cell types in the analysis were derived from the results of the Seurat workflow described above. Firstly the function computeCommunProb () was used to predict ligand‐receptor pairs in Sepsis and Healthy data, respectively. Then the function compareInteractions (), function netVisual_diffInteraction () and function netVisual_heatmap () were applied to analyse the number and strength of ligand‐receptor pairs separately for comparison, and finally the biological pathways related to cell communication were analysed using the function computeCommunProbPathway ().

## 3 RESULTS

### 3.1 Identification of cell types

**FIGURE 1:**
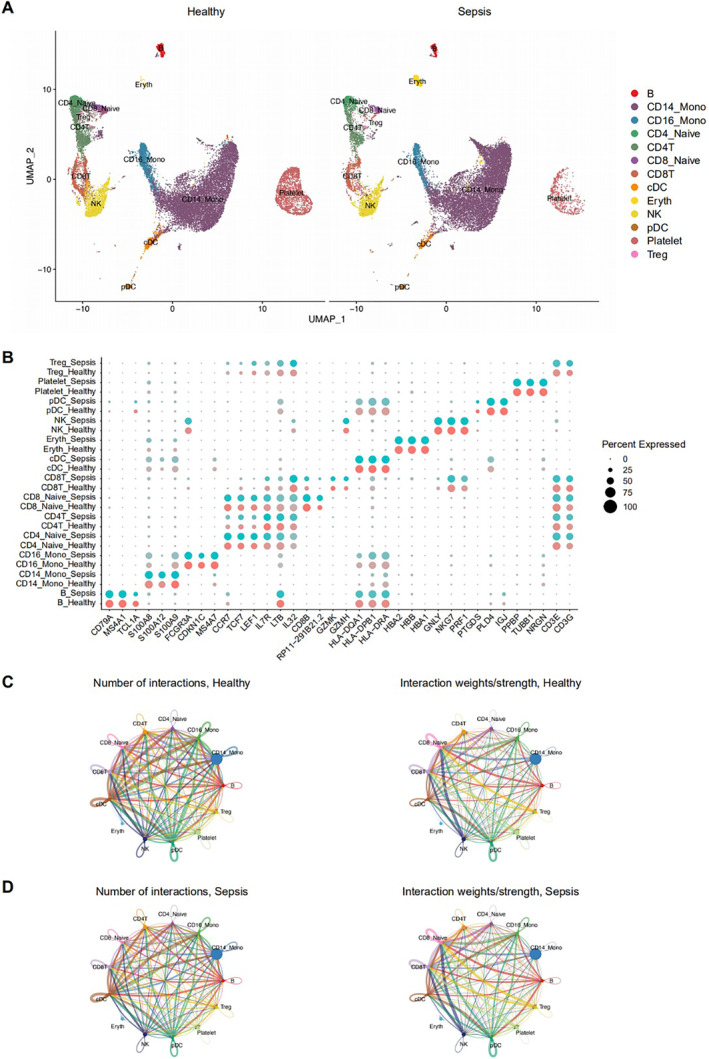
Analysis of scRNA‐seq data involving 5 normal individuals and 4 sepsis patients, and the cellular communication using CellChat (a) UMAP representation of cell clusters identified in healthy versus sepsis (b) Top3 of the differential expression genes of each cell type (c) The number and strength of cellular interactions in healthy and (d) The number and strength of cellular interactions in sepsis.

The UMI data from four septic patients and five healthy individuals were normalised using the LogNormalize () function in Seurat (v4.0.3) [26]. Statistical analysis of the scRNA‐seq profiles is shown in Figure S1A in Supporting Information S1. The results of integration and dimensionality reduction analyses demonstrated that cells from each sample were effectively integrated, indicating a well‐corrected batch effect (Figures S1B, C in Supporting Information S1). Using the FindCluster () function, 29 distinct clusters were identified (Figure S1D in Supporting Information S1), and the Azimuth algorithm (v0.4.3) was employed to enhance cell identity classification. Ultimately, 13 cell types present in both septic and healthy control groups were identified (Figure 1a), including B cells, CD14+ monocytes (CD14 Mono), CD16+ monocytes (CD16 Mono), CD4+ naive T cells (CD4 Naive), CD4+ T cells (CD4 T), CD8+ naive T cells (CD8 Naive), CD8+ T cells (CD8 T), conventional dendritic cells (cDC), erythroid cells (Eryth), natural killer cells (NK), plasmacytoid dendritic cells (pDC), platelets, and regulatory T cells (Treg). It was clear that the immune cell composition was markedly altered in sepsis compared with healthy controls, as evidenced by the proportions of each cell type (Figure S1E in Supporting Information S1), which might be associated/related with the abnormal cell‐cell communications between immune cells [22].

### 3.2 Differential gene expression

scRNA‐seq was used to identify differentially expressed genes (DEGs) within each cell cluster. As shown in Figure S1F in Supporting Information S1, septic patients exhibited a significantly higher number of DEGs in immune cells compared with healthy controls, and the DEGs differed markedly between cell types. To identify potential novel targets and enhance the accuracy of cell cluster identification, marker gene analysis was performed, highlighting genes that were highly differentially expressed in a specific cell cluster relative to all other clusters (Figure 1b, Table S1 in Supporting Information S2).

### 3.3 Difference of cellular communications between sepsis and healthy control

**FIGURE 2:**
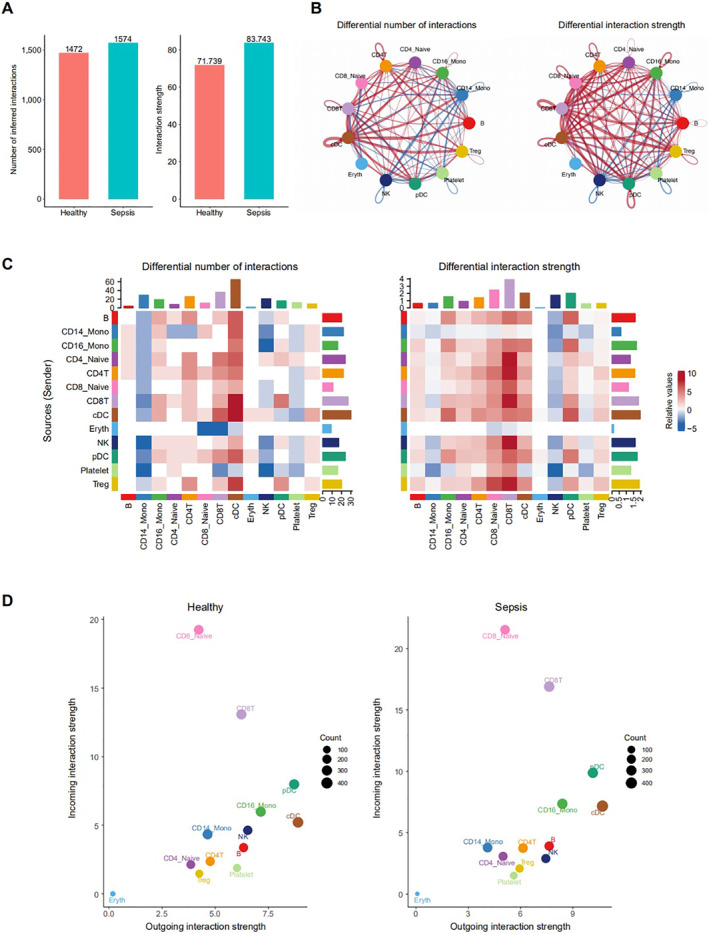
Analysis of cell‐cell communication in healthy and sepsis (a) The difference in cellular interactions between healthy and sepsis (b) The different number and strength of cellular interactions in healthy and sepsis. The interactions in sepsis were marked in red, and the interactions in healthy were marked in blue (c) The heatmap of the different number and strength of cellular interactions in healthy and sepsis. The interactions in sepsis were marked in red, and the interactions in healthy were marked in blue and (d) The outgoing/ingoing interaction strengths of different cell types in healthy and sepsis.

Cell‐cell communication between different cell types in septic and healthy individuals was analysed using CellChat (v1.5.0) [28]. Figures 1c and 1d depict cellular interactions among various cell types in healthy controls and septic patients, respectively. Figure 2a directly compares the number and strength of cell‐cell interactions between these groups. Overall, septic patients exhibited a greater number and stronger cell‐cell interactions, suggesting more complex signal transduction and immune regulation in sepsis. In Figures 2b and 2c, red indicates a higher number and strength of interactions in sepsis, while blue represents the same for healthy controls. These data clearly show that both the number and strength of cell‐cell communications were significantly elevated in sepsis, indicating more active cellular interactions in the disease state. Further analysis of outgoing and ingoing interaction strengths in different cell types revealed that cDCs and pDCs exhibited stronger outgoing interaction strength in both sepsis and healthy controls (Figure 2d). The abnormal changes observed in these two cell types during sepsis, as well as their downstream effects on other cells and pathways, were a key focus of the study.

### 3.4 Analysis of cellular communications in cDC and pDC

**FIGURE 3:**
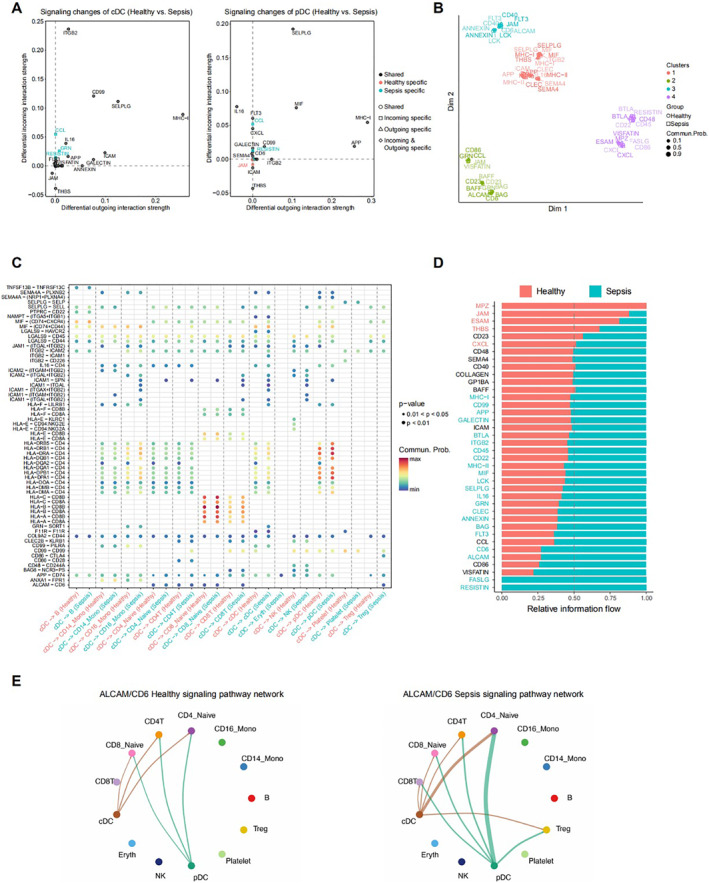
The analysis of cell‐cell communications in cDC and pDC (a) The changes of signalling pathways in cDC and pDC. Circle represented the shared pathway, Square represented pathways with incoming specific, Triangle represented pathways with outcoming specific, Rhombus represented pathways with both incoming and outcoming specific. Black represented pathways shared in healthy and sepsis, Pink represented pathways with healthy specific, Blue represented pathways with sepsis specific (b) The cluster analysis of expressed pathways. Different colour represented different cluster. Circle represented pathways in healthy, Square represented pathways in sepsis (c) The different expressions of receptor‐ligand pairs using cDC as ligand and all other cells as receptors (d) The different expressions of each pathway in healthy and sepsis. Red represented highly activated in healthy, Blue represented highly activated in sepsis and (e) The analysis of CD6‐ALCAM pathway in healthy (left) and sepsis (right).

We further investigated the differential expression of pathways involved in cDC and pDC communication between sepsis and healthy controls. The results showed that most pathways were more activated in sepsis (Figure 3a). Cluster analysis grouped these pathways into four categories, indicating that pathways in the same group likely share similar differential expression patterns (Figure 3b). Next, using cDCs as ligand cells and other cells as receptors, we analysed ligand‐receptor pair expression in sepsis and healthy controls to identify differentially expressed pathways (Figure 3c). Pathways with significant expression alterations may represent key factors in sepsis pathogenesis. The activation of each pathway was studied in both sepsis and control groups, revealing that more highly activated pathways were observed in sepsis compared to healthy individuals (Figure 3d). Finally, based on these analyses, the CD6‐ALCAM pathway was selected for further investigation due to its abnormal interaction strength in sepsis. As shown in Figure 3e, the CD6‐ALCAM pathway was more activated in sepsis than in healthy individuals, suggesting that this pathway may play a crucial role in the mechanisms underlying sepsis.

## 4 DISSCUSSION

Sepsis is one of the deadliest conditions in the ICU, characterised by an unknown aetiology and rapid progression [29, 30]. Both innate and adaptive immune responses remain altered long after clinical treatment in sepsis, making the investigation of these changes crucial for identifying potential therapeutic strategies for septic patients [31, 32]. Single‐cell RNA sequencing (scRNA‐seq), with its high resolution and accuracy, has proven instrumental in uncovering the cellular and molecular signatures underlying immune dysregulation in sepsis [33, 34, 35, 36]. Recent studies using transcriptomic sequencing have identified significant alterations and heterogeneity in immune cell subsets during polymicrobial sepsis [22]. Another study demonstrated that circulating myeloid‐derived suppressor cells (MDSCs) maintained an immunosuppressive transcriptomic profile in late‐stage sepsis using scRNA‐seq [19]. Similarly, circulating non‐myeloid cells have been investigated, revealing a unique transcriptomic pattern indicative of immunosuppression, inflammation, and dysfunction in late sepsis [18].These findings, based on scRNA‐seq technology, offer new perspectives on understanding immune dysregulation in sepsis, potentially informing clinical practice and treatment strategies.

In this study, we employed single‐cell transcriptomics to explore the cellular communication of non‐myeloid circulating cells in late‐stage sepsis. We identified 13 distinct immune cell types in both septic and healthy individuals, though their proportions varied significantly between the two groups. These differences are likely linked to altered immune regulation. Notably, the proportion of CD14+ monocytes (CD14 Mono) was significantly increased in sepsis. This increase has been observed in patients with Gram‐negative sepsis [37], potentially reflecting immune regulatory responses to the inflammatory environment in sepsis. CD14 Mono has also been proposed as a biomarker for diagnosis, prognosis, and prediction in neonatal sepsis [38], suggesting its potential as a clinical marker.

Another significant finding was the reduction in T‐lymphocyte subsets (CD4 Naive, CD4 T, CD8 Naive, CD8 T) in sepsis. T cell exhaustion is a hallmark of immunosuppression in septic patients [39, 40]. The depletion of T‐lymphocyte subsets is closely associated with immune dysregulation, which may contribute to the progression of sepsis [41]. Additionally, a notable decrease in platelet counts was observed in septic patients compared to healthy controls. Platelets have recently garnered attention for their role in the pathophysiology of infectious diseases, inflammation, and immunity [42]. Thrombocytopaenia in sepsis is often caused by bacterial infection and bone marrow suppression. Bacterial and viral infections in sepsis consume large amounts of coagulation factors, leading to hypercoagulability and platelet depletion [43, 44, 45]. Sepsis can also suppress bone marrow function, further contributing to reduced platelet counts [46, 47]. Low platelet levels are now considered a biomarker for disease severity in sepsis [48, 49].

Our analysis of cell identity and differential gene expression revealed significant alterations in immune cells between septic and healthy individuals, which directly affected cellular communication and signalling pathways. This study represents the first systematic exploration of cell‐cell communication alterations in sepsis, specifically among non‐myeloid circulating cells. The results demonstrated that both the number and strength of cellular interactions were significantly higher in sepsis, indicating more active intercellular communication and signal transduction, likely driven by immune dysregulation [50]. Analysis of differential cell‐cell communication between septic and healthy controls revealed numerous pathway alterations under pathological conditions, although the mechanisms of these more activated pathways remain unclear and require further investigation. Through receptor‐ligand analysis, cDCs and pDCs were identified as having stronger outgoing interaction strength, warranting further study.

Dendritic cells (DCs), including conventional dendritic cells (cDCs) and plasmacytoid dendritic cells (pDCs), are professional antigen‐presenting cells that serve as a bridge between innate and adaptive immunity [51]. These cells are essential for initiating humoral and cellular immune responses, as well as for protecting against infectious diseases [52, 53, 54]. A recent study identified a conserved, targetable immunoregulatory programme in DCs that was linked to hyperinflammation and organ dysfunction in sepsis. In this study, we focused on the altered cellular communication in cDCs and pDCs to investigate their potential role in the pathogenesis of sepsis. Despite a reduction in their numbers, the signalling pathways involving these two cell types were more activated in sepsis, suggesting that they play a critical role in disease‐related signalling. Cluster analysis revealed that pathways within the same group exhibited similar differential expression patterns, indicating that they may be regulated in a coordinated manner during sepsis.

Further investigation using cDCs as ligand cells identified several pathways with significantly altered expression, which may represent key factors in disease progression. Among these, the CD6‐ALCAM pathway, which was abnormally activated in sepsis, was selected for in‐depth analysis. CD6 is a co‐stimulatory receptor expressed on T cells, which binds to the activated leucocyte cell‐adhesion molecule (ALCAM), expressed on antigen‐presenting cells, as well as epithelial and endothelial tissues. The CD6‐ALCAM pathway modulates T cell activation, proliferation, and trafficking [55, 56]. It has been shown that CD6 and ALCAM form a key receptor‐ligand pair, playing an essential role in early DC‐T cell interactions and maintaining long‐term T cell proliferation after initial contact [57]. The CD6‐ALCAM pathway has been implicated in the pathogenesis of various diseases, including lupus nephritis, multiple sclerosis, and cancer [58, 59, 60, 61, 62]. In this study, we found that the CD6‐ALCAM pathway was abnormally activated in sepsis, which may be linked to the reduction in T‐lymphocyte subsets during disease. The CD6‐ALCAM pathway may influence the inflammatory response and immunosuppression by promoting T cell proliferation in sepsis. Further research is needed to clarify the specific mechanisms involved, but the CD6‐ALCAM pathway holds potential as a therapeutic target for sepsis.

## 5 CONCLUSION

In this study, we systematically analysed the cell‐cell communication of non‐myeloid circulating cells in late‐stage sepsis using scRNA‐seq. Our analysis revealed significant differences in the proportions of each cell type between septic and healthy individuals, indicating altered signal transduction between immune cells. A greater number of pathways were abnormally activated in sepsis compared to healthy controls. Both conventional dendritic cells (cDCs) and plasmacytoid dendritic cells (pDCs) exhibited stronger outgoing interaction strength in sepsis. By using cDCs as the ligand, we identified numerous altered pathways, among which the CD6‐ALCAM pathway was notably more highly activated. This activation may be closely associated with T cell suppression in sepsis. Overall, our findings provide new insights into the pathogenic mechanisms of sepsis and offer potential avenues for clinical intervention.

## AUTHOR CONTRIBUTIONS

**Yanyan Tao**: Data curation; investigation; resources. **Miao miao Li**: Writing ‐ original draft; writing ‐ review and editing. **Cheng Liu**: Project administration.

## CONFLICT OF INTEREST STATEMENT

The authors declared that they have no conflicts of interest to this work.

## Supporting information

Supporting Information S1

Supporting Information S2

Figure S1

Table S1

## Data Availability

Publicly available datasets were analysed in this study. This data can be found here: https://www.ncbi.nlm.nih.gov/geo/query/acc.cgi?acc=GSE175453.
