# Supplementary material for: Inference and analysis of cell‐cell communication of non‐myeloid circulating cells in late sepsis based on single‐cell RNA‐seq
Source: IET Syst Biol. 2024 Nov 22;18(6):218–26. doi: 10.1049/syb2.12109 (PMC11665843; doi:10.1049/syb2.12109)
Supplement: Supplementary file 2 — Supporting Information S2 [file SYB2-18-218-s002.docx]

Table S1. The significant marker genes for each given cell type.
