# Supplementary material for: Inference and analysis of cell‐cell communication of non‐myeloid circulating cells in late sepsis based on single‐cell RNA‐seq
Source: IET Syst Biol. 2024 Nov 22;18(6):218–26. doi: 10.1049/syb2.12109 (PMC11665843; doi:10.1049/syb2.12109)
Supplement: Supplementary file 4 — Table S1 [file SYB2-18-218-s001.pdf]

	p_val	avg_log2F(	pct.1	pct.2	p_val_adj	cluster	gene
CD79A	0	4.393558	0.986	0.011	0	B	CD79A
MS4A1	0	4.098149	0.976	0.005	0	B	MS4A1
TCL1A	0	3.500243	0.663	0.005	0	B	TCL1A
CD79B	0	2.853175	0.908	0.06	0	B	CD79B
CD74	0	2.689229	1	0.772	0	B	CD74
LINC0092(	0	2.667307	0.786	0.002	0	B	LINC00926
HLA-DQA	0	2.637921	0.962	0.178	0	B	HLA-DQA1
BANK1	0	2.61878	0.855	0.021	0	B	BANK1
RALGPS2	0	2.531797	0.798	0.036	0	B	RALGPS2
LTB	0	2.521531	0.963	0.274	0	B	LTB
FAM129C	0	2.484634	0.76	0.01	0	B	FAM129C
HLA-DRA	0	2.273584	1	0.489	0	B	HLA-DRA
CD37	0	2.240447	0.989	0.693	0	B	CD37
VPREB3	0	2.144828	0.621	0.001	0	B	VPREB3
FCER2	0	2.136954	0.616	0.003	0	B	FCER2
FAIM3	0	2.034693	0.776	0.127	0	B	FAIM3
HLA-DPB1	0	1.992268	0.982	0.417	0	B	HLA-DPB1
HVCN1	0	1.977166	0.788	0.164	0	B	HVCN1
RP11-693J	0	1.919796	0.608	0.002	0	B	RP11-693J15.5
BIRC3	0	1.882347	0.678	0.102	0	B	BIRC3
FCRLA	0	1.867858	0.598	0.002	0	B	FCRLA
PPAPDC1E	0	1.826315	0.585	0.05	0	B	PPAPDC1B
HLA-DRB1	0	1.82498	0.987	0.506	0	B	HLA-DRB1
HLA-DPA1	0	1.803004	0.986	0.44	0	B	HLA-DPA1
SPIB	0	1.788001	0.584	0.011	0	B	SPIB
RPS5	0	1.765948	0.989	0.703	0	B	RPS5
AFF3	0	1.749827	0.571	0.025	0	B	AFF3
PTPRCAP	0	1.682162	0.963	0.268	0	B	PTPRCAP
CD72	0	1.650509	0.472	0.013	0	B	CD72
HLA-DQB1	0	1.642353	0.945	0.352	0	B	HLA-DQB1
BLK	0	1.624533	0.55	0.003	0	B	BLK
IGLL5	0	1.551463	0.273	0.002	0	B	IGLL5
SWAP70	0	1.535309	0.658	0.108	0	B	SWAP70
RPL18A	0	1.495272	0.994	0.826	0	B	RPL18A
HLA-DOB	0	1.442027	0.484	0.006	0	B	HLA-DOB
BACH2	0	1.44019	0.408	0.027	0	B	BACH2
SYPL1	0	1.407428	0.656	0.154	0	B	SYPL1
CD22	0	1.403856	0.462	0.003	0	B	CD22
BLNK	0	1.395317	0.501	0.015	0	B	BLNK
RPS23	0	1.389842	0.993	0.824	0	B	RPS23
CD19	0	1.387609	0.455	0.001	0	B	CD19
CD180	0	1.380422	0.477	0.041	0	B	CD180
CD40	0	1.368805	0.501	0.052	0	B	CD40
GNG7	0	1.319185	0.45	0.023	0	B	GNG7
IGJ	0	1.307989	0.357	0.022	0	B	IGJ
TSPAN3	0	1.298187	0.507	0.065	0	B	TSPAN3
ADAM28	0	1.281939	0.422	0.028	0	B	ADAM28
BTLA	0	1.274985	0.452	0.018	0	B	BTLA
ID3	0	1.261791	0.449	0.041	0	B	ID3
CXXC5	0	1.258454	0.582	0.119	0	B	CXXC5
TPD52	0	1.214431	0.463	0.043	0	B	TPD52
FCRL1	0	1.211085	0.366	0.001	0	B	FCRL1
ARHGAP2	0	1.118563	0.433	0.067	0	B	ARHGAP24
SNX29P2	0	1.100267	0.349	0.024	0	B	SNX29P2
SP140	0	1.100039	0.447	0.07	0	B	SP140
KIAA0226L	0	1.096257	0.375	0.017	0	B	KIAA0226L
POU2AF1	0	1.088401	0.358	0.002	0	B	POU2AF1

COBLL1	0	1.075106	0.332	0.007	0	B	COBLL1
PAX5	0	1.062358	0.324	0.001	0	B	PAX5
TCF4	0	1.046416	0.429	0.061	0	B	TCF4
CDCA7L	0	1.0088	0.331	0.019	0	B	CDCA7L
AC079767	0	0.997954	0.254	0.002	0	B	AC079767.4
STRBP	0	0.993253	0.372	0.029	0	B	STRBP
RP11-164I	0	0.986673	0.223	0.001	0	B	RP11-164H13.1
QRSL1	0	0.966818	0.364	0.043	0	B	QRSL1
CXCR5	0	0.954363	0.286	0.004	0	B	CXCR5
RP5-887A	0	0.939419	0.166	0	0	B	RP5-887A10.1
CCR6	0	0.93922	0.33	0.023	0	B	CCR6
STAP1	0	0.935803	0.308	0.007	0	B	STAP1
TNFRSF13	0	0.92072	0.315	0.001	0	B	TNFRSF13C
TSPAN13	0	0.913477	0.354	0.023	0	B	TSPAN13
PKIG	0	0.901638	0.347	0.015	0	B	PKIG
SNX22	0	0.87262	0.273	0.004	0	B	SNX22
CD200	0	0.868495	0.239	0.002	0	B	CD200
TLR10	0	0.863194	0.277	0.011	0	B	TLR10
MZB1	0	0.838317	0.284	0.012	0	B	MZB1
P2RY10	0	0.828832	0.304	0.029	0	B	P2RY10
TMEM156	0	0.808241	0.284	0.027	0	B	TMEM156
PNOC	0	0.801488	0.28	0.006	0	B	PNOC
RAB30	0	0.76903	0.263	0.011	0	B	RAB30
CLEC17A	0	0.759295	0.246	0.003	0	B	CLEC17A
COL19A1	0	0.750454	0.21	0	0	B	COL19A1
BCL7A	0	0.732752	0.222	0.016	0	B	BCL7A
P2RX5	0	0.718119	0.226	0.008	0	B	P2RX5
KIAA0125	0	0.70848	0.188	0.004	0	B	KIAA0125
FCRL5	0	0.692513	0.186	0.001	0	B	FCRL5
3-Mar	0	0.673791	0.214	0.013	0	B	3-Mar
BACE2	0	0.648955	0.196	0.001	0	B	BACE2
RP11-38J2	0	0.598307	0.197	0.01	0	B	RP11-38J22.6
CPNE5	0	0.595378	0.223	0.01	0	B	CPNE5
EBF1	0	0.592449	0.183	0	0	B	EBF1
E2F5	0	0.586649	0.188	0.007	0	B	E2F5
PCDH9	0	0.580776	0.134	0.002	0	B	PCDH9
TNFRSF13I	0	0.551269	0.161	0	0	B	TNFRSF13B
KHDRBS2	0	0.550052	0.169	0.007	0	B	KHDRBS2
FCRL2	0	0.543781	0.159	0	0	B	FCRL2
OSBPL10	0	0.543122	0.182	0.001	0	B	OSBPL10
CNR2	0	0.525287	0.182	0.004	0	B	CNR2
KLHL14	0	0.523377	0.155	0	0	B	KLHL14
GYLTL1B	0	0.520449	0.145	0.002	0	B	GYLTL1B
RP11-297I	0	0.516791	0.141	0	0	B	RP11-297B17.3
RP11-689I	0	0.505492	0.159	0.001	0	B	RP11-689B22.2
DENND5B	0	0.473928	0.151	0.006	0	B	DENND5B
BEND5	0	0.465373	0.146	0.006	0	B	BEND5
CR2	0	0.454987	0.124	0.001	0	B	CR2
RASGRP3	0	0.454281	0.151	0.002	0	B	RASGRP3
PLEKHG1	0	0.452481	0.139	0.001	0	B	PLEKHG1
PTPRK	0	0.434818	0.134	0.002	0	B	PTPRK
RP11-731I	0	0.417803	0.116	0.001	0	B	RP11-731F5.2
ZNF860	0	0.413509	0.119	0	0	B	ZNF860
RP11-624(	0	0.406971	0.115	0	0	B	RP11-624C23.1
MACROD2	0	0.38672	0.102	0.001	0	B	MACROD2
PAWR	0	0.385289	0.129	0.004	0	B	PAWR
AC006129	0	0.379379	0.115	0.001	0	B	AC006129.4
DBNDD1	0	0.36705	0.101	0.002	0	B	DBNDD1

CTA-250D	0	0.336018	0.104	0	0	B	CTA-250D10.23
NT5E	#######	0.480823	0.131	0.006	#######	B	NT5E
C12orf42	#######	0.389345	0.119	0.005	#######	B	C12orf42
BCL11A	#######	0.843946	0.332	0.042	#######	B	BCL11A
CYB561A3	#######	1.255004	0.5	0.097	#######	B	CYB561A3
HS3ST1	#######	0.403261	0.111	0.004	#######	B	HS3ST1
RPS27	#######	1.505596	0.993	0.83	#######	B	RPS27
RPSA	#######	1.456046	0.989	0.696	#######	B	RPSA
BCAS4	#######	0.720038	0.251	0.025	#######	B	BCAS4
RPL13	#######	1.169323	0.997	0.861	#######	B	RPL13
CD52	#######	1.462879	0.99	0.646	#######	B	CD52
HIP1R	#######	0.489478	0.158	0.01	#######	B	HIP1R
SEL1L3	#######	0.721601	0.271	0.03	#######	B	SEL1L3
RPS18	#######	1.374372	0.994	0.787	#######	B	RPS18
ISG20	#######	1.368498	0.655	0.174	#######	B	ISG20
RPS8	#######	1.045517	0.997	0.878	#######	B	RPS8
PDLIM1	#######	0.265601	0.506	0.091	#######	B	PDLIM1
HLA-DOA	#######	0.80738	0.27	0.031	#######	B	HLA-DOA
RPL29	#######	1.081382	0.994	0.834	#######	B	RPL29
RPS19	#######	1.26072	0.993	0.814	#######	B	RPS19
RPL10A	#######	1.316464	0.986	0.685	#######	B	RPL10A
RIC3	#######	0.69993	0.257	0.029	#######	B	RIC3
MT-ND4L	#######	1.298742	0.99	0.772	#######	B	MT-ND4L
EEF1B2	#######	1.366107	0.984	0.67	#######	B	EEF1B2
RPS21	#######	1.045723	0.997	0.834	#######	B	RPS21
RPL8	#######	1.041965	0.994	0.857	#######	B	RPL8
RPL37	#######	0.956702	0.994	0.867	#######	B	RPL37
STAG3	#######	0.547529	0.107	0.005	#######	B	STAG3
RPL18	#######	0.970537	0.999	0.849	#######	B	RPL18
RPL19	#######	0.933339	0.999	0.869	#######	B	RPL19
RRAS2	#######	0.616536	0.24	0.026	#######	B	RRAS2
SNX2	#######	1.307301	0.767	0.286	#######	B	SNX2
RPL32	#######	0.961614	0.996	0.87	#######	B	RPL32
GNB2L1	#######	1.028986	0.99	0.798	#######	B	GNB2L1
RPL23A	#######	1.173961	0.986	0.738	#######	B	RPL23A
RPS25	#######	1.142492	0.99	0.774	#######	B	RPS25
ADAM19	#######	0.658038	0.281	0.037	#######	B	ADAM19
RPS6	#######	1.112262	0.996	0.78	#######	B	RPS6
RP11-861/	#######	0.514712	0.173	0.015	#######	B	RP11-861A13.4
RPS27A	#######	0.99254	0.996	0.867	#######	B	RPS27A
RPL7A	#######	0.950982	0.993	0.838	#######	B	RPL7A
RPL10	#######	0.972141	1	0.878	#######	B	RPL10
CD83	#######	0.672754	0.212	0.022	#######	B	CD83
RPL3	#######	1.221265	0.989	0.744	#######	B	RPL3
RPL11	#######	0.882492	0.999	0.882	#######	B	RPL11
MT-ATP8	#######	1.396741	0.98	0.734	#######	B	MT-ATP8
RPS2	#######	1.031356	0.994	0.814	#######	B	RPS2
ZCCHC7	#######	1.072198	0.445	0.096	#######	B	ZCCHC7
RPL30	#######	0.905698	0.999	0.886	#######	B	RPL30
LBH	#######	1.129862	0.607	0.161	#######	B	LBH
RPLP0	#######	1.151216	0.994	0.775	#######	B	RPLP0
EEF1A1	#######	0.993508	1	0.891	#######	B	EEF1A1
RPS15	#######	0.865737	0.999	0.865	#######	B	RPS15
EEF1G	#######	1.376765	0.916	0.519	#######	B	EEF1G
RPL12	#######	0.92812	0.994	0.867	#######	B	RPL12
CHD7	#######	0.657129	0.261	0.035	#######	B	CHD7
RPS3A	#######	0.91082	0.996	0.856	#######	B	RPS3A
CXCR4	#######	1.241782	0.58	0.163	#######	B	CXCR4

PARP15	#######	0.81178	0.327	0.054	#######	B		PARP15
RPS29	#######	1.140856	0.983	0.763	#######	B		RPS29
SNHG7	#######	1.178936	0.639	0.204	#######	B		SNHG7
RPS10	#######	1.116688	0.972	0.762	#######	B		RPS10
RHOH	#######	1.043944	0.462	0.105	#######	B		RHOH
AKAP2	#######	0.447799	0.185	0.018	#######	B		AKAP2
RPL5	#######	0.975679	0.994	0.799	#######	B		RPL5
RPS12	#######	0.928013	1	0.881	#######	B		RPS12
RPS15A	#######	0.897938	0.996	0.868	#######	B		RPS15A
CTB-133G	#######	1.076892	0.547	0.152	#######	B		CTB-133G6.1
RPS4X	#######	0.934038	0.993	0.811	#######	B		RPS4X
KMO	#######	0.649605	0.246	0.035	#######	B		KMO
IFT57	#######	0.818273	0.298	0.05	#######	B		IFT57
HLA-DMA	#######	1.137554	0.79	0.296	#######	B		HLA-DMA
CDK14	#######	0.394293	0.139	0.011	#######	B		CDK14
BTG1	#######	1.205868	0.979	0.68	#######	B		BTG1
RPL34	#######	0.803274	0.996	0.873	#######	B		RPL34
PARP1	#######	1.152104	0.612	0.206	#######	B		PARP1
RPL15	#######	0.92097	0.989	0.8	#######	B		RPL15
IRF8	#######	0.866714	0.462	0.115	#######	B		IRF8
RPS7	#######	0.767973	0.997	0.864	#######	B		RPS7
RPL26	#######	0.903267	0.99	0.805	#######	B		RPL26
RPL36	#######	0.83273	0.987	0.829	#######	B		RPL36
RPS3	#######	0.882399	0.996	0.83	#######	B		RPS3
RPL17	#######	1.073285	0.97	0.713	#######	B		RPL17
TCTN1	#######	0.492755	0.156	0.016	#######	B		TCTN1
RPS16	#######	0.903602	0.989	0.764	#######	B		RPS16
EAF2	#######	0.839584	0.315	0.061	#######	B		EAF2
FAM3C	#######	0.714498	0.295	0.054	#######	B		FAM3C
HLA-DMB	#######	1.030723	0.661	0.233	#######	B		HLA-DMB
MEF2C	#######	1.232244	0.783	0.336	#######	B		MEF2C
GLTSCR2	#######	1.116567	0.866	0.453	#######	B		GLTSCR2
PHACTR1	#######	0.591476	0.17	0.019	#######	B		PHACTR1
RPL35	#######	0.892082	0.986	0.768	#######	B		RPL35
IFNG-AS1	#######	0.402639	0.125	0.011	#######	B		IFNG-AS1
CCR7	#######	0.814204	0.419	0.098	#######	B		CCR7
RPL13A	#######	0.885505	0.98	0.765	#######	B		RPL13A
PLEKHF2	#######	0.837561	0.376	0.089	#######	B		PLEKHF2
PPM1K	#######	0.881624	0.422	0.109	#######	B		PPM1K
RP11-94L1	#######	0.636712	0.261	0.045	#######	B		RP11-94L15.2
C16orf74	#######	0.580208	0.202	0.028	#######	B		C16orf74
EEF2	#######	0.981395	0.969	0.696	#######	B		EEF2
TSTD1	#######	0.951082	0.534	0.168	#######	B		TSTD1
CNFN	#######	0.373003	0.119	0.01	#######	B		CNFN
RP11-421L	#######	0.466437	0.169	0.02	#######	B		RP11-421L21.3
HIST1H4C	#######	1.090071	0.875	0.479	#######	B		HIST1H4C
ARID5B	#######	0.76049	0.332	0.072	#######	B		ARID5B
RPL35A	#######	0.700298	0.991	0.87	#######	B		RPL35A
AIM2	#######	0.583059	0.183	0.024	#######	B		AIM2
RP11-796E	#######	0.849491	0.31	0.066	#######	B		RP11-796E2.4
SYNGR2	#######	1.052342	0.767	0.368	#######	B		SYNGR2
ADK	#######	1.020555	0.412	0.116	#######	B		ADK
RPL14	#######	0.749006	0.994	0.809	#######	B		RPL14
RPL21	#######	0.807024	0.989	0.814	#######	B		RPL21
RPL41	#######	0.705387	0.994	0.883	#######	B		RPL41
RPL28	#######	0.614705	1	0.896	#######	B		RPL28
LIMS2	#######	0.279727	0.104	0.008	#######	B		LIMS2
FCGR2B	#######	0.7147	0.276	0.055	#######	B		FCGR2B

SEC62	#######	0.971773	0.881	0.513	#######	B	SEC62
NPM1	#######	0.983614	0.898	0.521	#######	B	NPM1
TRAF5	#######	0.580102	0.22	0.036	#######	B	TRAF5
SMC6	#######	0.703894	0.341	0.081	#######	B	SMC6
IL4R	#######	1.066889	0.411	0.119	#######	B	IL4R
FADS3	#######	0.348231	0.101	0.008	#######	B	FADS3
SHMT2	#######	0.806074	0.411	0.119	#######	B	SHMT2
TAGAP	#######	0.995971	0.622	0.243	#######	B	TAGAP
RPL9	#######	0.752401	0.984	0.8	#######	B	RPL9
HSP90AB1	#######	1.026067	0.908	0.548	#######	B	HSP90AB1
RPL39	#######	0.580208	0.999	0.914	#######	B	RPL39
FAU	#######	0.570882	0.996	0.902	#######	B	FAU
RPL37A	#######	0.725328	0.983	0.807	#######	B	RPL37A
TMEM243	#######	0.900681	0.486	0.165	#######	B	TMEM243
MT-ND3	#######	0.663487	0.989	0.906	#######	B	MT-ND3
EZR	#######	1.00802	0.646	0.285	#######	B	EZR
CD82	#######	0.662047	0.433	0.128	#######	B	CD82
IKZF3	#######	0.54376	0.233	0.043	#######	B	IKZF3
SETBP1	#######	0.363535	0.135	0.016	#######	B	SETBP1
PDE4B	#######	0.640405	0.283	0.062	#######	B	PDE4B
SLC38A1	#######	0.800913	0.405	0.118	#######	B	SLC38A1
RPS28	#######	0.574462	0.997	0.899	#######	B	RPS28
CD1C	#######	0.636231	0.189	0.03	#######	B	CD1C
ITM2C	#######	0.496303	0.209	0.036	#######	B	ITM2C
SP100	#######	0.936053	0.723	0.36	#######	B	SP100
SIT1	#######	0.6439	0.317	0.075	#######	B	SIT1
FCRL3	#######	0.593354	0.168	0.024	#######	B	FCRL3
C11orf80	#######	0.304591	0.104	0.01	#######	B	C11orf80
KIAA0040	#######	0.795356	0.411	0.126	#######	B	KIAA0040
METTL8	#######	0.515732	0.183	0.03	#######	B	METTL8
PLD4	#######	0.615705	0.253	0.054	#######	B	PLD4
AGPAT5	#######	0.61153	0.267	0.06	#######	B	AGPAT5
KLF2	#######	0.901959	0.909	0.526	#######	B	KLF2
MT-CYB	#######	0.625439	0.994	0.93	#######	B	MT-CYB
CD81	#######	0.845658	0.585	0.24	#######	B	CD81
STK17A	#######	0.883146	0.572	0.222	#######	B	STK17A
C15orf57	#######	0.631042	0.284	0.069	#######	B	C15orf57
AC006129	#######	0.741583	0.345	0.094	#######	B	AC006129.2
ZNF107	#######	0.434409	0.161	0.025	#######	B	ZNF107
HMGN1	#######	0.757712	0.741	0.355	#######	B	HMGN1
LPAR5	#######	0.375371	0.169	0.027	#######	B	LPAR5
RPL38	#######	0.758016	0.963	0.724	#######	B	RPL38
LMBRD1	#######	0.757584	0.464	0.168	#######	B	LMBRD1
IL2RG	#######	0.619374	0.759	0.327	#######	B	IL2RG
RPS20	#######	0.817447	0.896	0.588	#######	B	RPS20
BTK	#######	0.706026	0.443	0.153	#######	B	BTK
GPR18	#######	0.402282	0.156	0.024	#######	B	GPR18
SMIM14	#######	0.868861	0.45	0.164	#######	B	SMIM14
LIMD2	#######	0.836485	0.903	0.587	1.30E-99	B	LIMD2
RABEP2	#######	0.566405	0.214	0.044	2.26E-99	B	RABEP2
TTN	#######	0.32648	0.118	0.014	2.37E-99	B	TTN
FOXP1	#######	0.938498	0.57	0.249	3.20E-99	B	FOXP1
P2RY14	#######	0.385847	0.109	0.013	1.04E-98	B	P2RY14
TIFA	#######	0.49541	0.224	0.047	1.52E-98	B	TIFA
LINC0066!	#######	0.297301	0.108	0.012	1.93E-98	B	LINC00665
RP11-220I	#######	0.701955	0.369	0.113	2.39E-98	B	RP11-220I1.1
ZNF296	#######	0.498866	0.188	0.035	2.56E-98	B	ZNF296
RPLP1	#######	0.543351	0.997	0.944	5.97E-98	B	RPLP1

POLD4	#######	0.856989	0.732	0.392	9.06E-98	B	POLD4
ADARB1	#######	0.437154	0.148	0.022	1.69E-97	B	ADARB1
RPS11	#######	0.679453	0.973	0.752	2.38E-95	B	RPS11
ORAI2	#######	0.574555	0.384	0.121	2.48E-95	B	ORAI2
MBD4	6.22E-99	0.764838	0.462	0.175	2.04E-94	B	MBD4
PPP3CC	2.18E-97	0.662052	0.381	0.121	7.12E-93	B	PPP3CC
RPS9	2.48E-97	0.617076	0.986	0.829	8.13E-93	B	RPS9
RPL24	5.99E-97	0.646355	0.99	0.799	1.96E-92	B	RPL24
GGA2	2.32E-95	0.647819	0.295	0.082	7.61E-91	B	GGA2
RPL6	3.38E-95	0.546642	0.994	0.863	1.11E-90	B	RPL6
RPSAP58	7.92E-95	0.721469	0.386	0.13	2.59E-90	B	RPSAP58
RPL27	1.73E-94	0.687276	0.976	0.735	5.66E-90	B	RPL27
TNFAIP8	1.82E-94	0.788637	0.595	0.279	5.95E-90	B	TNFAIP8
UVRAG	4.34E-94	0.750582	0.446	0.17	1.42E-89	B	UVRAG
FCHSD2	2.68E-93	0.617272	0.321	0.095	8.76E-89	B	FCHSD2
SH3BP5	5.24E-93	0.682205	0.472	0.18	1.72E-88	B	SH3BP5
RPL4	7.70E-93	0.702464	0.964	0.721	2.52E-88	B	RPL4
MOB3B	1.37E-92	0.295975	0.107	0.013	4.49E-88	B	MOB3B
RPS14	6.17E-92	0.516119	0.994	0.872	2.02E-87	B	RPS14
RP11-325I	5.13E-91	0.316761	0.108	0.014	1.68E-86	B	RP11-325F22.2
SMARCB1	5.46E-90	0.728427	0.425	0.161	1.79E-85	B	SMARCB1
RPL22L1	1.02E-89	0.803405	0.572	0.269	3.35E-85	B	RPL22L1
EEF1D	1.37E-88	0.609367	0.986	0.786	4.48E-84	B	EEF1D
TRAF4	1.60E-87	0.320011	0.115	0.016	5.24E-83	B	TRAF4
CAMK1D	1.80E-87	0.66196	0.352	0.116	5.89E-83	B	CAMK1D
RPLP2	5.96E-87	0.504487	0.996	0.876	1.95E-82	B	RPLP2
GAPT	1.67E-85	0.673341	0.415	0.153	5.45E-81	B	GAPT
9-Mar	7.26E-84	0.388713	0.165	0.032	2.38E-79	B	9-Mar
IMPDH2	1.44E-83	0.713262	0.366	0.131	4.71E-79	B	IMPDH2
PPP1R14A	2.95E-83	0.331735	0.156	0.028	9.66E-79	B	PPP1R14A
HINT1	5.02E-82	0.67453	0.906	0.608	1.64E-77	B	HINT1
ETS1	7.86E-82	0.556839	0.491	0.187	2.57E-77	B	ETS1
SNX29	2.52E-81	0.652688	0.358	0.125	8.25E-77	B	SNX29
ARHGAP1!	2.58E-81	0.652827	0.491	0.203	8.44E-77	B	ARHGAP15
SNHG8	3.65E-81	0.769793	0.592	0.291	1.19E-76	B	SNHG8
PTPN6	4.97E-81	0.669421	0.83	0.511	1.63E-76	B	PTPN6
FAM69A	3.25E-80	0.359679	0.142	0.025	1.07E-75	B	FAM69A
CAMK2D	2.41E-79	0.647844	0.288	0.089	7.90E-75	B	CAMK2D
DCK	1.67E-78	0.662243	0.375	0.138	5.46E-74	B	DCK
RP9	4.49E-78	0.679596	0.291	0.091	1.47E-73	B	RP9
RPS13	8.96E-78	0.449961	0.999	0.887	2.93E-73	B	RPS13
ANAPC16	1.77E-77	0.655598	0.636	0.334	5.80E-73	B	ANAPC16
MTSS1	1.40E-76	0.563326	0.405	0.149	4.59E-72	B	MTSS1
ELOVL5	1.73E-76	0.671516	0.445	0.185	5.66E-72	B	ELOVL5
STX7	2.36E-76	0.644707	0.53	0.245	7.72E-72	B	STX7
EIF1AY	5.04E-76	0.585895	0.354	0.124	1.65E-71	B	EIF1AY
RPL22	9.73E-76	0.500242	0.983	0.839	3.19E-71	B	RPL22
CCDC50	1.07E-75	0.521751	0.293	0.092	3.51E-71	B	CCDC50
NACA	2.27E-75	0.540823	0.991	0.819	7.42E-71	B	NACA
PDCD4	1.32E-74	0.681627	0.497	0.224	4.34E-70	B	PDCD4
MARCKSL1	1.44E-74	0.847894	0.375	0.144	4.72E-70	B	MARCKSL1
BIN1	2.78E-74	0.626995	0.401	0.151	9.09E-70	B	BIN1
SYVN1	4.00E-73	0.447571	0.19	0.045	1.31E-68	B	SYVN1
OFD1	2.68E-72	0.622073	0.314	0.106	8.77E-68	B	OFD1
USP6NL	4.59E-72	0.315211	0.151	0.03	1.50E-67	B	USP6NL
APPL1	5.69E-71	0.668908	0.48	0.221	1.86E-66	B	APPL1
SELL	3.81E-70	0.590685	0.795	0.459	1.25E-65	B	SELL
RPS26	1.93E-69	0.827228	0.967	0.828	6.32E-65	B	RPS26

LINC-PINT	1.04E-68	0.430645	0.226	0.063	3.40E-64	B	LINC-PINT
ACP5	4.48E-68	0.468867	0.233	0.067	1.47E-63	B	ACP5
BASP1	4.01E-67	0.251858	0.396	0.149	1.31E-62	B	BASP1
DRAM2	6.66E-67	0.647277	0.375	0.152	2.18E-62	B	DRAM2
CIITA	2.74E-66	0.534458	0.267	0.086	8.96E-62	B	CIITA
JUN	4.49E-66	0.542447	0.205	0.054	1.47E-61	B	JUN
HNRNPA1	4.50E-66	0.637823	0.911	0.627	1.47E-61	B	HNRNPA1
GPR183	1.02E-65	0.525797	0.259	0.08	3.34E-61	B	GPR183
GPR160	1.05E-65	0.311912	0.139	0.028	3.44E-61	B	GPR160
MT-CO1	1.56E-65	0.349719	1	0.986	5.11E-61	B	MT-CO1
EIF4A2	4.05E-65	0.635776	0.57	0.303	1.33E-60	B	EIF4A2
LY86	6.50E-65	0.550812	0.56	0.267	2.13E-60	B	LY86
OPN3	6.93E-65	0.616182	0.307	0.11	2.27E-60	B	OPN3
MT-CO2	3.13E-64	0.349981	1	0.976	1.03E-59	B	MT-CO2
PLCG2	4.04E-64	0.521173	0.274	0.091	1.32E-59	B	PLCG2
PDE7A	6.66E-64	0.509884	0.274	0.091	2.18E-59	B	PDE7A
ST6GAL1	1.18E-63	0.521779	0.27	0.09	3.86E-59	B	ST6GAL1
MALAT1	1.65E-63	0.411609	1	0.944	5.40E-59	B	MALAT1
CCNG2	3.05E-63	0.479889	0.298	0.105	9.98E-59	B	CCNG2
SLAMF1	4.83E-63	0.353453	0.146	0.032	1.58E-58	B	SLAMF1
SMAGP	1.38E-62	0.356455	0.132	0.027	4.52E-58	B	SMAGP
HHEX	1.50E-62	0.524565	0.365	0.147	4.91E-58	B	HHEX
PPP1R16B	7.84E-62	0.308245	0.118	0.022	2.57E-57	B	PPP1R16B
NT5C	8.20E-62	0.592027	0.378	0.16	2.68E-57	B	NT5C
PLEKHA2	9.00E-62	0.586467	0.334	0.13	2.95E-57	B	PLEKHA2
PHB2	7.55E-61	0.638985	0.608	0.346	2.47E-56	B	PHB2
NFKB2	1.64E-60	0.502405	0.283	0.099	5.37E-56	B	NFKB2
UGCG	3.28E-60	0.332373	0.236	0.072	1.07E-55	B	UGCG
CLECL1	1.52E-59	0.514883	0.142	0.032	4.98E-55	B	CLECL1
PNRC1	1.56E-59	0.549155	0.932	0.667	5.10E-55	B	PNRC1
PNISR	1.69E-59	0.625627	0.693	0.421	5.52E-55	B	PNISR
S1PR1	1.94E-59	0.484987	0.308	0.109	6.37E-55	B	S1PR1
FXYD1	3.03E-59	0.275528	0.108	0.02	9.93E-55	B	FXYD1
SEMA4B	4.29E-59	0.339683	0.156	0.037	1.40E-54	B	SEMA4B
HSPE1	4.55E-59	0.64778	0.493	0.245	1.49E-54	B	HSPE1
PEBP1	1.15E-58	0.60541	0.489	0.242	3.77E-54	B	PEBP1
HLA-DRB5	1.19E-58	0.629769	0.621	0.314	3.89E-54	B	HLA-DRB5
RAC2	2.05E-58	0.503734	0.868	0.577	6.70E-54	B	RAC2
ZNF92	3.82E-58	0.437082	0.165	0.042	1.25E-53	B	ZNF92
RNASEH2B	7.07E-58	0.58924	0.386	0.17	2.32E-53	B	RNASEH2B
RPS4Y1	1.04E-57	0.677193	0.739	0.462	3.41E-53	B	RPS4Y1
BCL2	2.08E-57	0.450518	0.203	0.059	6.81E-53	B	BCL2
CD53	3.00E-57	0.590293	0.722	0.46	9.83E-53	B	CD53
C12orf57	4.61E-57	0.467956	0.601	0.3	1.51E-52	B	C12orf57
ZDHHC21	8.81E-57	0.340113	0.155	0.038	2.88E-52	B	ZDHHC21
BTG2	4.44E-56	0.552477	0.466	0.227	1.45E-51	B	BTG2
MT-ND6	5.90E-56	0.66934	0.685	0.412	1.93E-51	B	MT-ND6
EIF3F	3.20E-55	0.553536	0.815	0.539	1.05E-50	B	EIF3F
LSM7	4.22E-55	0.530441	0.683	0.414	1.38E-50	B	LSM7
AIDA	8.45E-55	0.463299	0.26	0.091	2.77E-50	B	AIDA
SLC25A42	9.41E-54	0.257288	0.105	0.02	3.08E-49	B	SLC25A42
CIRBP	2.53E-53	0.544328	0.712	0.445	8.27E-49	B	CIRBP
PPIA	3.74E-53	0.49132	0.959	0.731	1.22E-48	B	PPIA
6-Sep	8.18E-53	0.405437	0.574	0.309	2.68E-48	B	6-Sep
C11orf24	1.39E-52	0.397762	0.169	0.047	4.57E-48	B	C11orf24
ATP6V1G1	3.23E-52	0.444208	0.834	0.571	1.06E-47	B	ATP6V1G1
BLOC1S2	5.49E-52	0.535528	0.388	0.178	1.80E-47	B	BLOC1S2
CERS4	6.61E-52	0.355526	0.143	0.035	2.16E-47	B	CERS4

NCR3	7.39E-52	0.339961	0.214	0.066	2.42E-47	B	NCR3
SNX3	1.22E-51	0.386533	0.759	0.503	3.99E-47	B	SNX3
RCSD1	1.94E-51	0.504438	0.804	0.565	6.35E-47	B	RCSD1
ISCU	2.31E-51	0.434575	0.589	0.34	7.56E-47	B	ISCU
FUT8	4.52E-51	0.263375	0.143	0.035	1.48E-46	B	FUT8
ZCCHC11	5.21E-51	0.566187	0.33	0.138	1.70E-46	B	ZCCHC11
GPATCH1:	9.32E-51	0.479274	0.239	0.083	3.05E-46	B	GPATCH11
FBL	1.22E-50	0.534624	0.523	0.285	4.00E-46	B	FBL
FAM43A	3.99E-49	0.392783	0.17	0.049	1.31E-44	B	FAM43A
UBE2D2	4.23E-49	0.483558	0.666	0.42	1.38E-44	B	UBE2D2
TXNIP	1.19E-48	0.503942	0.977	0.744	3.90E-44	B	TXNIP
NCL	1.73E-48	0.524093	0.761	0.496	5.68E-44	B	NCL
EIF3E	1.90E-48	0.532746	0.746	0.489	6.23E-44	B	EIF3E
ERP29	1.91E-48	0.517044	0.795	0.543	6.26E-44	B	ERP29
PHF1	3.41E-48	0.39968	0.214	0.071	1.12E-43	B	PHF1
CLEC2D	1.15E-47	0.500368	0.244	0.088	3.77E-43	B	CLEC2D
GUCD1	1.52E-47	0.403172	0.207	0.069	4.98E-43	B	GUCD1
15-Sep	1.66E-47	0.529985	0.509	0.284	5.44E-43	B	15-Sep
NUAK2	2.07E-47	0.378947	0.199	0.064	6.77E-43	B	NUAK2
CHCHD10	4.19E-47	0.531415	0.601	0.35	1.37E-42	B	CHCHD10
RAN	6.39E-47	0.479877	0.707	0.451	2.09E-42	B	RAN
IRF4	1.23E-46	0.322123	0.118	0.028	4.04E-42	B	IRF4
PXK	1.55E-46	0.476198	0.294	0.121	5.08E-42	B	PXK
RPL31	2.52E-45	0.447817	0.949	0.733	8.26E-41	B	RPL31
MAT2B	4.70E-45	0.483777	0.507	0.284	1.54E-40	B	MAT2B
FDFT1	7.63E-45	0.530944	0.422	0.215	2.50E-40	B	FDFT1
WHSC1L1	7.90E-45	0.563285	0.482	0.267	2.59E-40	B	WHSC1L1
NOC3L	8.54E-45	0.37932	0.17	0.052	2.80E-40	B	NOC3L
MAPK13	1.07E-44	0.251099	0.102	0.022	3.51E-40	B	MAPK13
HSH2D	1.85E-44	0.347628	0.25	0.093	6.04E-40	B	HSH2D
ARHGAP1	3.17E-44	0.498304	0.294	0.126	1.04E-39	B	ARHGAP17
SRSF7	4.14E-44	0.554083	0.567	0.342	1.36E-39	B	SRSF7
SPTBN1	5.92E-44	0.365504	0.213	0.074	1.94E-39	B	SPTBN1
CTD-3184	6.31E-44	0.435442	0.212	0.074	2.07E-39	B	CTD-3184A7.4
SIPA1L3	7.27E-44	0.258367	0.116	0.028	2.38E-39	B	SIPA1L3
PAOX	1.04E-43	0.305692	0.122	0.03	3.39E-39	B	PAOX
APEX1	1.23E-43	0.542622	0.462	0.251	4.04E-39	B	APEX1
HNRNPL	1.64E-43	0.453796	0.526	0.304	5.35E-39	B	HNRNPL
METTL21A	1.74E-43	0.398998	0.205	0.071	5.69E-39	B	METTL21A
LTBP3	2.39E-43	0.341304	0.149	0.043	7.81E-39	B	LTBP3
PRKCB	3.54E-43	0.402214	0.639	0.4	1.16E-38	B	PRKCB
LAT2	7.80E-43	0.421829	0.452	0.232	2.55E-38	B	LAT2
FGD2	2.07E-42	0.411896	0.364	0.172	6.76E-38	B	FGD2
ANXA6	3.20E-42	0.433338	0.601	0.361	1.05E-37	B	ANXA6
MAP4K1	5.43E-42	0.351652	0.21	0.074	1.78E-37	B	MAP4K1
POLD2	6.64E-42	0.382472	0.195	0.067	2.17E-37	B	POLD2
SF1	7.68E-42	0.402653	0.656	0.414	2.52E-37	B	SF1
TESK2	8.16E-42	0.270378	0.111	0.027	2.67E-37	B	TESK2
EVL	1.06E-41	0.364941	0.545	0.288	3.46E-37	B	EVL
UCP2	1.29E-41	0.483697	0.79	0.548	4.22E-37	B	UCP2
ANKAR	2.64E-41	0.29165	0.124	0.032	8.64E-37	B	ANKAR
WDR74	3.42E-41	0.543448	0.374	0.186	1.12E-36	B	WDR74
ZFAS1	4.04E-41	0.491797	0.714	0.48	1.32E-36	B	ZFAS1
ITPR1	4.27E-41	0.404647	0.207	0.075	1.40E-36	B	ITPR1
AL592284.	6.23E-41	0.418629	0.212	0.077	2.04E-36	B	AL592284.1
CCT3	1.09E-40	0.457259	0.446	0.243	3.58E-36	B	CCT3
MT-CO3	1.50E-40	0.28137	0.996	0.969	4.91E-36	B	MT-CO3
IL16	2.72E-40	0.42682	0.473	0.256	8.90E-36	B	IL16

SP110	3.25E-40	0.456644	0.54	0.317	1.06E-35	B	SP110
CYSLTR1	3.40E-40	0.377906	0.24	0.095	1.11E-35	B	CYSLTR1
PPP1CC	3.62E-40	0.476454	0.497	0.288	1.18E-35	B	PPP1CC
BEX2	4.05E-40	0.31844	0.161	0.049	1.33E-35	B	BEX2
HDAC1	6.63E-40	0.483176	0.411	0.217	2.17E-35	B	HDAC1
ZNF821	8.19E-40	0.250664	0.105	0.025	2.68E-35	B	ZNF821
SLAMF6	1.06E-39	0.313974	0.148	0.043	3.46E-35	B	SLAMF6
TAPT1	1.28E-39	0.33363	0.129	0.036	4.20E-35	B	TAPT1
1-Sep	2.30E-39	0.406912	0.324	0.142	7.53E-35	B	1-Sep
OCIAD2	4.17E-39	0.453312	0.259	0.105	1.37E-34	B	OCIAD2
MT-ND5	4.21E-39	0.389476	0.97	0.791	1.38E-34	B	MT-ND5
USP11	4.65E-39	0.388022	0.193	0.068	1.52E-34	B	USP11
DOPEY2	8.38E-39	0.376635	0.209	0.078	2.74E-34	B	DOPEY2
FAM96A	8.70E-39	0.4928	0.477	0.278	2.85E-34	B	FAM96A
ABLIM1	1.01E-38	0.283394	0.241	0.092	3.31E-34	B	ABLIM1
TMEM154	1.14E-38	0.265235	0.453	0.239	3.75E-34	B	TMEM154
1-Mar	1.73E-38	0.389048	0.544	0.31	5.66E-34	B	1-Mar
HSPA8	1.77E-38	0.374368	0.847	0.574	5.80E-34	B	HSPA8
UXT	2.14E-38	0.449236	0.662	0.432	7.02E-34	B	UXT
CYFIP2	2.24E-38	0.415067	0.352	0.169	7.35E-34	B	CYFIP2
RBM38	3.81E-38	0.29536	0.305	0.137	1.25E-33	B	RBM38
MIF	4.85E-38	0.445799	0.798	0.569	1.59E-33	B	MIF
RABEP1	6.41E-38	0.463695	0.274	0.12	2.10E-33	B	RABEP1
KPNA5	7.41E-38	0.319132	0.166	0.055	2.42E-33	B	KPNA5
PRMT1	1.34E-37	0.486809	0.341	0.169	4.40E-33	B	PRMT1
PIM2	1.50E-37	0.37195	0.213	0.08	4.92E-33	B	PIM2
MGAT5	1.72E-37	0.286164	0.134	0.039	5.64E-33	B	MGAT5
TBC1D10C	2.30E-37	0.409195	0.411	0.211	7.54E-33	B	TBC1D10C
CHD2	6.04E-37	0.47705	0.386	0.203	1.98E-32	B	CHD2
ZFP36L1	9.10E-37	0.359994	0.746	0.507	2.98E-32	B	ZFP36L1
SP140L	1.31E-36	0.48262	0.25	0.107	4.27E-32	B	SP140L
FAM65B	1.78E-36	0.362651	0.696	0.463	5.82E-32	B	FAM65B
NCOA3	2.29E-36	0.449365	0.351	0.179	7.48E-32	B	NCOA3
MYO1E	3.93E-36	0.348241	0.145	0.046	1.29E-31	B	MYO1E
THRAP3	5.94E-36	0.4345	0.543	0.331	1.95E-31	B	THRAP3
CHPT1	6.48E-36	0.433438	0.297	0.139	2.12E-31	B	CHPT1
CCT2	1.05E-35	0.485879	0.386	0.208	3.45E-31	B	CCT2
HDAC9	1.74E-35	0.408565	0.214	0.086	5.68E-31	B	HDAC9
EIF3D	1.77E-35	0.45658	0.527	0.325	5.80E-31	B	EIF3D
P2RY8	2.92E-35	0.395157	0.27	0.119	9.57E-31	B	P2RY8
LSM2	9.87E-35	0.430055	0.392	0.211	3.23E-30	B	LSM2
TUBB	1.26E-34	0.352409	0.601	0.38	4.14E-30	B	TUBB
CNN2	2.25E-34	0.339047	0.607	0.385	7.35E-30	B	CNN2
CARD11	3.53E-34	0.277832	0.143	0.045	1.15E-29	B	CARD11
ZNF581	4.04E-34	0.46155	0.276	0.129	1.32E-29	B	ZNF581
ALOX5	4.39E-34	0.324721	0.401	0.214	1.44E-29	B	ALOX5
LY9	7.94E-34	0.331119	0.18	0.065	2.60E-29	B	LY9
DCTPP1	1.15E-33	0.347146	0.202	0.08	3.77E-29	B	DCTPP1
SET	1.25E-33	0.393208	0.733	0.52	4.11E-29	B	SET
ATIC	1.69E-33	0.37464	0.224	0.093	5.53E-29	B	ATIC
DDHD2	2.07E-33	0.25861	0.119	0.035	6.76E-29	B	DDHD2
CDC123	3.63E-33	0.380772	0.311	0.154	1.19E-28	B	CDC123
CD69	3.84E-33	0.415777	0.172	0.061	1.26E-28	B	CD69
KIAA0922	4.48E-33	0.372826	0.294	0.14	1.47E-28	B	KIAA0922
SESN1	4.63E-33	0.423456	0.178	0.066	1.52E-28	B	SESN1
UBXN1	5.56E-33	0.428914	0.673	0.459	1.82E-28	B	UBXN1
C1QBP	9.13E-33	0.446249	0.477	0.287	2.99E-28	B	C1QBP
HERPUD1	1.84E-32	0.403803	0.46	0.272	6.01E-28	B	HERPUD1

ACAP1	1.95E-32	0.304767	0.457	0.253	6.37E-28	B	ACAP1
LAPTM5	2.32E-32	0.382364	0.946	0.749	7.59E-28	B	LAPTM5
FUS	4.61E-32	0.433228	0.636	0.426	1.51E-27	B	FUS
FAM107B	5.64E-32	0.393802	0.52	0.318	1.85E-27	B	FAM107B
MIR142	6.40E-32	0.342538	0.433	0.246	2.09E-27	B	MIR142
KDM4C	6.74E-32	0.374639	0.226	0.097	2.21E-27	B	KDM4C
SLC50A1	7.00E-32	0.323083	0.207	0.085	2.29E-27	B	SLC50A1
RPL36A	7.45E-32	0.402739	0.875	0.631	2.44E-27	B	RPL36A
SNORD3A	1.20E-31	0.34591	0.175	0.066	3.92E-27	B	SNORD3A
CCNI	2.04E-31	0.369907	0.898	0.691	6.68E-27	B	CCNI
CEPT1	3.79E-31	0.303173	0.202	0.082	1.24E-26	B	CEPT1
RCN2	5.51E-31	0.375558	0.263	0.122	1.80E-26	B	RCN2
DNPH1	5.83E-31	0.412074	0.268	0.13	1.91E-26	B	DNPH1
DDX6	6.05E-31	0.374925	0.348	0.183	1.98E-26	B	DDX6
CHMP7	7.23E-31	0.428327	0.226	0.099	2.37E-26	B	CHMP7
RFX5	7.29E-31	0.31276	0.169	0.063	2.39E-26	B	RFX5
TMEM19	8.37E-31	0.293043	0.149	0.052	2.74E-26	B	TMEM19
HNRNPDL	9.49E-31	0.374589	0.658	0.459	3.11E-26	B	HNRNPDL
C11orf31	9.86E-31	0.392375	0.678	0.474	3.23E-26	B	C11orf31
DDAH2	1.58E-30	0.332326	0.26	0.121	5.17E-26	B	DDAH2
PWP1	1.62E-30	0.417319	0.274	0.133	5.32E-26	B	PWP1
RPA3	1.69E-30	0.353288	0.254	0.117	5.54E-26	B	RPA3
FAM60A	1.75E-30	0.34715	0.25	0.114	5.72E-26	B	FAM60A
RP11-35G	3.10E-30	0.290407	0.132	0.043	1.01E-25	B	RP11-35G9.3
SNRPD2	3.53E-30	0.306082	0.815	0.573	1.16E-25	B	SNRPD2
TOMM7	4.25E-30	0.347258	0.923	0.7	1.39E-25	B	TOMM7
ZBTB20	4.30E-30	0.319268	0.162	0.06	1.41E-25	B	ZBTB20
PRKD2	4.45E-30	0.308158	0.185	0.072	1.46E-25	B	PRKD2
TAF1D	8.16E-30	0.482878	0.44	0.27	2.67E-25	B	TAF1D
FAM26F	8.23E-30	0.359219	0.348	0.18	2.69E-25	B	FAM26F
KRT10	1.04E-29	0.376199	0.516	0.325	3.40E-25	B	KRT10
MRPL39	1.57E-29	0.277491	0.135	0.046	5.14E-25	B	MRPL39
DARS	1.72E-29	0.433206	0.314	0.163	5.62E-25	B	DARS
FAM214A	2.22E-29	0.250152	0.145	0.051	7.28E-25	B	FAM214A
SPCS2	2.72E-29	0.329208	0.511	0.322	8.90E-25	B	SPCS2
SS18L2	3.21E-29	0.386155	0.325	0.173	1.05E-24	B	SS18L2
MYC	3.52E-29	0.418015	0.223	0.099	1.15E-24	B	MYC
REL	5.08E-29	0.390059	0.528	0.337	1.66E-24	B	REL
TBC1D5	5.90E-29	0.379044	0.345	0.187	1.93E-24	B	TBC1D5
ZNF880	6.67E-29	0.253954	0.118	0.037	2.18E-24	B	ZNF880
BBX	6.85E-29	0.393102	0.338	0.182	2.24E-24	B	BBX
MATR3	8.36E-29	0.381513	0.528	0.338	2.74E-24	B	MATR3
EPC1	1.03E-28	0.399795	0.473	0.295	3.36E-24	B	EPC1
MTG1	1.03E-28	0.311829	0.224	0.099	3.39E-24	B	MTG1
SSBP2	2.59E-28	0.29837	0.189	0.078	8.49E-24	B	SSBP2
CCNB1IP1	2.60E-28	0.312699	0.146	0.053	8.51E-24	B	CCNB1IP1
TMEM66	2.69E-28	0.297473	0.801	0.548	8.82E-24	B	TMEM66
ZBED5	3.31E-28	0.331008	0.213	0.094	1.08E-23	B	ZBED5
WDFY4	3.86E-28	0.266518	0.152	0.056	1.27E-23	B	WDFY4
TRAF3IP3	6.56E-28	0.328168	0.635	0.429	2.15E-23	B	TRAF3IP3
NASP	8.38E-28	0.373289	0.3	0.155	2.74E-23	B	NASP
PAPOLA	1.04E-27	0.30094	0.496	0.314	3.40E-23	B	PAPOLA
TRIM13	1.33E-27	0.252857	0.158	0.06	4.37E-23	B	TRIM13
STMN1	1.62E-27	0.301951	0.18	0.072	5.29E-23	B	STMN1
ZBTB24	1.80E-27	0.255873	0.142	0.051	5.89E-23	B	ZBTB24
ZNF431	1.84E-27	0.289042	0.172	0.068	6.04E-23	B	ZNF431
LHPP	1.96E-27	0.326228	0.178	0.073	6.41E-23	B	LHPP
ITSN2	1.98E-27	0.375982	0.436	0.263	6.48E-23	B	ITSN2

FNBP4	2.46E-27	0.377557	0.391	0.23	8.06E-23	B	FNBP4
NGLY1	3.14E-27	0.323167	0.236	0.11	1.03E-22	B	NGLY1
KIAA1033	3.94E-27	0.314977	0.391	0.225	1.29E-22	B	KIAA1033
CSK	5.55E-27	0.366122	0.621	0.435	1.82E-22	B	CSK
ATF7IP	5.90E-27	0.392407	0.295	0.154	1.93E-22	B	ATF7IP
COX7C	9.19E-27	0.368041	0.905	0.704	3.01E-22	B	COX7C
KRR1	1.10E-26	0.351644	0.206	0.091	3.61E-22	B	KRR1
TAF7	1.17E-26	0.399226	0.378	0.218	3.82E-22	B	TAF7
CCT4	1.21E-26	0.407204	0.433	0.272	3.96E-22	B	CCT4
LRMP	1.41E-26	0.415079	0.268	0.134	4.63E-22	B	LRMP
EIF2S3	2.25E-26	0.381991	0.491	0.32	7.37E-22	B	EIF2S3
IMP3	2.30E-26	0.36585	0.449	0.275	7.53E-22	B	IMP3
RNGTT	2.49E-26	0.310945	0.149	0.057	8.14E-22	B	RNGTT
SRSF5	2.52E-26	0.367612	0.649	0.463	8.24E-22	B	SRSF5
EIF3L	2.64E-26	0.340082	0.763	0.54	8.65E-22	B	EIF3L
TAPSAR1	2.66E-26	0.355038	0.402	0.239	8.70E-22	B	TAPSAR1
GLS	2.99E-26	0.364465	0.283	0.146	9.78E-22	B	GLS
HMGN4	5.29E-26	0.33875	0.219	0.101	1.73E-21	B	HMGN4
HSPD1	5.92E-26	0.411902	0.429	0.269	1.94E-21	B	HSPD1
NHP2	6.33E-26	0.378401	0.418	0.257	2.07E-21	B	NHP2
ZNF141	7.41E-26	0.287413	0.122	0.042	2.43E-21	B	ZNF141
ACADM	7.52E-26	0.363078	0.214	0.099	2.46E-21	B	ACADM
NUP88	7.86E-26	0.26679	0.131	0.047	2.57E-21	B	NUP88
ANKRD44	8.42E-26	0.43513	0.391	0.234	2.76E-21	B	ANKRD44
AES	1.19E-25	0.325311	0.629	0.419	3.89E-21	B	AES
ABHD14B	1.25E-25	0.366189	0.315	0.172	4.10E-21	B	ABHD14B
FOXO1	1.26E-25	0.3325	0.173	0.072	4.14E-21	B	FOXO1
SLC25A38	1.31E-25	0.296319	0.138	0.05	4.29E-21	B	SLC25A38
PCBP2	1.31E-25	0.328054	0.866	0.637	4.29E-21	B	PCBP2
KIF20B	1.40E-25	0.301369	0.131	0.047	4.57E-21	B	KIF20B
XRCC6	1.41E-25	0.351464	0.54	0.36	4.63E-21	B	XRCC6
IQCB1	1.53E-25	0.262248	0.138	0.051	5.00E-21	B	IQCB1
EML4	1.89E-25	0.346553	0.384	0.225	6.20E-21	B	EML4
TRIM22	2.83E-25	0.378107	0.422	0.259	9.28E-21	B	TRIM22
NGDN	4.56E-25	0.35745	0.239	0.117	1.49E-20	B	NGDN
ARL4A	5.51E-25	0.326306	0.161	0.066	1.80E-20	B	ARL4A
UBIAD1	6.70E-25	0.273118	0.135	0.05	2.19E-20	B	UBIAD1
CCT7	7.28E-25	0.392836	0.385	0.235	2.38E-20	B	CCT7
ARL14EP	7.39E-25	0.364295	0.256	0.13	2.42E-20	B	ARL14EP
NOP58	8.98E-25	0.389457	0.307	0.168	2.94E-20	B	NOP58
LAMTOR5	1.19E-24	0.291368	0.497	0.324	3.88E-20	B	LAMTOR5
PSIP1	1.23E-24	0.346675	0.384	0.228	4.03E-20	B	PSIP1
DNAJC10	1.82E-24	0.33725	0.27	0.139	5.95E-20	B	DNAJC10
BTN2A2	2.21E-24	0.251138	0.119	0.042	7.24E-20	B	BTN2A2
RPL27A	2.48E-24	0.30735	0.851	0.643	8.12E-20	B	RPL27A
SNX5	3.04E-24	0.357634	0.366	0.217	9.95E-20	B	SNX5
ERH	3.59E-24	0.300975	0.531	0.352	1.18E-19	B	ERH
AL592183.	4.16E-24	0.381782	0.388	0.236	1.36E-19	B	AL592183.1
NDUFAF4	4.65E-24	0.295475	0.19	0.085	1.52E-19	B	NDUFAF4
CDC40	4.79E-24	0.342102	0.388	0.235	1.57E-19	B	CDC40
ACBD3	6.99E-24	0.303384	0.266	0.138	2.29E-19	B	ACBD3
CENPC	7.60E-24	0.31159	0.217	0.103	2.49E-19	B	CENPC
CYB5A	9.33E-24	0.26867	0.18	0.079	3.05E-19	B	CYB5A
NDNL2	1.05E-23	0.331296	0.216	0.102	3.44E-19	B	NDNL2
HIST1H1D	1.07E-23	0.412819	0.26	0.133	3.52E-19	B	HIST1H1D
NHP2L1	1.16E-23	0.332162	0.534	0.356	3.79E-19	B	NHP2L1
ST13	1.17E-23	0.272863	0.616	0.434	3.84E-19	B	ST13
ACYP2	1.69E-23	0.266578	0.226	0.109	5.53E-19	B	ACYP2

SOD1	1.80E-23	0.2851	0.591	0.392	5.88E-19	B	SOD1
ZNF75A	1.85E-23	0.252246	0.153	0.062	6.05E-19	B	ZNF75A
RINL	2.14E-23	0.280144	0.158	0.065	6.99E-19	B	RINL
ATP10D	2.58E-23	0.29493	0.183	0.082	8.45E-19	B	ATP10D
TRMT112	5.21E-23	0.2533	0.619	0.435	1.71E-18	B	TRMT112
GTF3A	6.56E-23	0.321663	0.516	0.347	2.15E-18	B	GTF3A
TRIM38	7.03E-23	0.261741	0.395	0.241	2.30E-18	B	TRIM38
BTF3	8.96E-23	0.269727	0.939	0.74	2.93E-18	B	BTF3
CDKN1B	9.28E-23	0.33771	0.332	0.191	3.04E-18	B	CDKN1B
OSGEP	1.56E-22	0.316159	0.24	0.123	5.09E-18	B	OSGEP
SSR2	1.63E-22	0.338896	0.665	0.483	5.34E-18	B	SSR2
BRIX1	2.17E-22	0.299792	0.178	0.079	7.11E-18	B	BRIX1
WDR77	2.44E-22	0.279587	0.172	0.076	7.98E-18	B	WDR77
PRDM2	3.48E-22	0.364489	0.277	0.15	1.14E-17	B	PRDM2
HMGN2	3.80E-22	0.261227	0.727	0.526	1.24E-17	B	HMGN2
KIAA1430	5.39E-22	0.27859	0.209	0.1	1.77E-17	B	KIAA1430
EIF3H	5.86E-22	0.321352	0.713	0.527	1.92E-17	B	EIF3H
UBE2I	6.02E-22	0.257302	0.557	0.386	1.97E-17	B	UBE2I
UBTF	7.41E-22	0.316241	0.274	0.147	2.43E-17	B	UBTF
ADD1	8.52E-22	0.365175	0.376	0.232	2.79E-17	B	ADD1
SECISBP2L	1.21E-21	0.323878	0.25	0.132	3.98E-17	B	SECISBP2L
ZNF22	1.25E-21	0.319969	0.277	0.152	4.09E-17	B	ZNF22
UBA52	1.38E-21	0.270919	0.983	0.837	4.52E-17	B	UBA52
FAM58A	1.40E-21	0.293899	0.209	0.103	4.58E-17	B	FAM58A
NOB1	1.48E-21	0.322383	0.205	0.098	4.84E-17	B	NOB1
PIKFYVE	1.92E-21	0.255736	0.193	0.09	6.29E-17	B	PIKFYVE
EPB41L4A-	2.36E-21	0.302929	0.192	0.091	7.71E-17	B	EPB41L4A-AS1
L3MBTL3	3.72E-21	0.268494	0.146	0.061	1.22E-16	B	L3MBTL3
PHF14	4.11E-21	0.289566	0.274	0.15	1.35E-16	B	PHF14
EAPP	4.30E-21	0.2909	0.371	0.227	1.41E-16	B	EAPP
EIF4B	5.11E-21	0.346476	0.621	0.446	1.67E-16	B	EIF4B
CNPPD1	6.58E-21	0.314475	0.318	0.187	2.16E-16	B	CNPPD1
MCM5	7.34E-21	0.28275	0.176	0.081	2.40E-16	B	MCM5
MRPS18B	7.53E-21	0.34955	0.29	0.165	2.47E-16	B	MRPS18B
ZFP14	8.62E-21	0.264429	0.135	0.055	2.82E-16	B	ZFP14
EIF5A	1.05E-20	0.274686	0.578	0.406	3.45E-16	B	EIF5A
BLCAP	1.32E-20	0.302921	0.22	0.112	4.31E-16	B	BLCAP
MALT1	1.54E-20	0.305269	0.214	0.109	5.03E-16	B	MALT1
HNRNPA0	2.73E-20	0.386215	0.435	0.292	8.93E-16	B	HNRNPA0
ATXN10	3.41E-20	0.320558	0.32	0.19	1.12E-15	B	ATXN10
KHDRBS1	4.66E-20	0.296608	0.565	0.395	1.53E-15	B	KHDRBS1
MZT2B	5.08E-20	0.252519	0.673	0.485	1.66E-15	B	MZT2B
TNFRSF14	6.19E-20	0.284039	0.432	0.281	2.03E-15	B	TNFRSF14
NIPA2	1.14E-19	0.261434	0.206	0.103	3.73E-15	B	NIPA2
ATP5O	1.39E-19	0.269153	0.663	0.489	4.54E-15	B	ATP5O
PPHLN1	1.70E-19	0.342266	0.266	0.149	5.58E-15	B	PPHLN1
MED10	1.94E-19	0.32149	0.331	0.202	6.36E-15	B	MED10
NUP107	2.33E-19	0.271799	0.165	0.075	7.62E-15	B	NUP107
NGRN	2.51E-19	0.276498	0.175	0.083	8.23E-15	B	NGRN
RBBP7	2.90E-19	0.306282	0.27	0.153	9.49E-15	B	RBBP7
UBE2E2	2.96E-19	0.251683	0.256	0.141	9.69E-15	B	UBE2E2
SMDT1	3.07E-19	0.295415	0.565	0.412	1.00E-14	B	SMDT1
ZNF292	6.12E-19	0.297828	0.308	0.183	2.00E-14	B	ZNF292
PPIH	6.82E-19	0.280014	0.229	0.121	2.23E-14	B	PPIH
RB1	7.48E-19	0.296441	0.347	0.217	2.45E-14	B	RB1
UPF3B	8.04E-19	0.268325	0.182	0.088	2.63E-14	B	UPF3B
SMARCC1	1.04E-18	0.27916	0.298	0.176	3.40E-14	B	SMARCC1
SLC25A6	1.21E-18	0.269465	0.92	0.689	3.97E-14	B	SLC25A6

APH1A	1.35E-18	0.333423	0.384	0.253	4.42E-14	B	APH1A
C12orf65	1.66E-18	0.251483	0.185	0.09	5.44E-14	B	C12orf65
RRP15	1.85E-18	0.297978	0.153	0.07	6.05E-14	B	RRP15
UBE2N	2.13E-18	0.324414	0.318	0.194	6.97E-14	B	UBE2N
SEPHS2	2.18E-18	0.268049	0.281	0.164	7.13E-14	B	SEPHS2
CWF19L2	2.24E-18	0.355127	0.241	0.133	7.32E-14	B	CWF19L2
SGSM3	2.34E-18	0.252902	0.162	0.075	7.67E-14	B	SGSM3
DPF2	2.54E-18	0.260576	0.205	0.106	8.31E-14	B	DPF2
SRM	2.67E-18	0.307657	0.307	0.186	8.75E-14	B	SRM
MAP3K8	3.51E-18	0.282034	0.257	0.145	1.15E-13	B	MAP3K8
DEGS1	4.37E-18	0.312664	0.217	0.117	1.43E-13	B	DEGS1
TRAPPC6A	4.83E-18	0.256428	0.268	0.152	1.58E-13	B	TRAPPC6A
OXA1L	6.34E-18	0.342438	0.415	0.281	2.07E-13	B	OXA1L
KMT2E	9.24E-18	0.293578	0.527	0.376	3.02E-13	B	KMT2E
AP3B1	9.28E-18	0.309553	0.297	0.179	3.04E-13	B	AP3B1
ZNF302	1.12E-17	0.270143	0.195	0.098	3.67E-13	B	ZNF302
OCIAD1	1.70E-17	0.279655	0.425	0.291	5.57E-13	B	OCIAD1
RNPS1	1.93E-17	0.294083	0.408	0.275	6.32E-13	B	RNPS1
ANP32E	2.41E-17	0.259397	0.277	0.163	7.89E-13	B	ANP32E
ZC3H8	3.73E-17	0.262942	0.156	0.073	1.22E-12	B	ZC3H8
EGLN2	4.62E-17	0.25095	0.25	0.144	1.51E-12	B	EGLN2
MOB4	4.75E-17	0.294131	0.22	0.12	1.56E-12	B	MOB4
TTC3	4.91E-17	0.321488	0.359	0.229	1.61E-12	B	TTC3
WBSCR22	5.36E-17	0.268954	0.219	0.119	1.75E-12	B	WBSCR22
SNRNP40	5.91E-17	0.350237	0.237	0.134	1.94E-12	B	SNRNP40
UBA2	6.30E-17	0.273457	0.27	0.16	2.06E-12	B	UBA2
CLNS1A	1.10E-16	0.298286	0.391	0.261	3.60E-12	B	CLNS1A
MCM3	1.10E-16	0.270137	0.143	0.066	3.62E-12	B	MCM3
URI1	1.26E-16	0.267879	0.314	0.195	4.12E-12	B	URI1
KIAA0020	1.44E-16	0.313852	0.193	0.101	4.73E-12	B	KIAA0020
MZT2A	1.50E-16	0.282496	0.31	0.19	4.91E-12	B	MZT2A
APOBEC3C	1.84E-16	0.254042	0.253	0.147	6.03E-12	B	APOBEC3C
MPHOSPH	2.09E-16	0.316944	0.489	0.347	6.85E-12	B	MPHOSPH8
ABT1	2.21E-16	0.251962	0.219	0.121	7.23E-12	B	ABT1
VPS51	2.22E-16	0.285078	0.324	0.207	7.28E-12	B	VPS51
HSPA4	2.91E-16	0.267988	0.263	0.156	9.53E-12	B	HSPA4
GLO1	3.10E-16	0.302102	0.263	0.157	1.02E-11	B	GLO1
PSMC6	3.53E-16	0.261467	0.31	0.196	1.16E-11	B	PSMC6
SDHD	3.99E-16	0.328214	0.318	0.205	1.31E-11	B	SDHD
SMARCE1	5.21E-16	0.265359	0.32	0.203	1.71E-11	B	SMARCE1
TMEM109	2.83E-15	0.283538	0.241	0.141	9.25E-11	B	TMEM109
CDK4	5.60E-15	0.267433	0.189	0.104	1.83E-10	B	CDK4
MDH1	1.08E-14	0.269182	0.351	0.234	3.52E-10	B	MDH1
TMEM134	2.07E-14	0.256806	0.217	0.125	6.79E-10	B	TMEM134
PTOV1	3.65E-14	0.262485	0.229	0.135	1.19E-09	B	PTOV1
MITD1	4.36E-14	0.279998	0.263	0.163	1.43E-09	B	MITD1
MORC3	7.74E-14	0.27119	0.234	0.14	2.53E-09	B	MORC3
RSL1D1	9.75E-14	0.257016	0.509	0.373	3.19E-09	B	RSL1D1
MTRNR2L2	1.54E-13	0.269125	0.155	0.08	5.05E-09	B	MTRNR2L2
ARF6	2.57E-13	0.280179	0.381	0.269	8.41E-09	B	ARF6
UTP23	3.32E-13	0.262642	0.202	0.117	1.09E-08	B	UTP23
CCDC115	3.34E-13	0.255226	0.224	0.134	1.09E-08	B	CCDC115
TMEM230	4.08E-13	0.290839	0.342	0.237	1.34E-08	B	TMEM230
ABCE1	8.79E-13	0.266106	0.182	0.104	2.88E-08	B	ABCE1
MRFAP1L1	9.21E-13	0.261704	0.236	0.145	3.01E-08	B	MRFAP1L1
TOMM20	9.30E-13	0.255126	0.463	0.347	3.05E-08	B	TOMM20
BUB3	9.82E-13	0.253032	0.317	0.212	3.22E-08	B	BUB3
CCDC91	2.26E-12	0.253922	0.199	0.118	7.39E-08	B	CCDC91

SDR39U1	2.33E-12	0.253726	0.192	0.11	7.62E-08	B	SDR39U1
ARMC1	2.85E-12	0.260614	0.151	0.08	9.34E-08	B	ARMC1
C6orf48	3.33E-12	0.26004	0.227	0.142	1.09E-07	B	C6orf48
RHBDF2	5.03E-12	0.273555	0.185	0.106	1.65E-07	B	RHBDF2
UTP6	1.30E-11	0.256079	0.176	0.101	4.24E-07	B	UTP6
RP11-773I	2.02E-11	0.250862	0.158	0.087	6.61E-07	B	RP11-773D16.1
NDUFV1	2.06E-10	0.255174	0.335	0.238	6.73E-06	B	NDUFV1
YBX3	8.07E-10	0.293543	0.385	0.289	2.64E-05	B	YBX3
MRPS26	2.11E-09	0.255	0.207	0.135	6.91E-05	B	MRPS26
S100A8	0	5.874741	0.995	0.359	0	CD14_Mor	S100A8
S100A12	0	4.988377	0.852	0.073	0	CD14_Mor	S100A12
S100A9	0	4.852682	0.983	0.336	0	CD14_Mor	S100A9
VCAN	0	3.678074	0.834	0.133	0	CD14_Mor	VCAN
LYZ	0	3.49077	0.949	0.331	0	CD14_Mor	LYZ
MNDA	0	2.993589	0.884	0.199	0	CD14_Mor	MNDA
FOS	0	2.703207	0.656	0.117	0	CD14_Mor	FOS
CD14	0	2.620133	0.662	0.067	0	CD14_Mor	CD14
FCN1	0	2.483806	0.844	0.188	0	CD14_Mor	FCN1
S100A6	0	2.468651	0.99	0.762	0	CD14_Mor	S100A6
NEAT1	0	2.410878	0.898	0.464	0	CD14_Mor	NEAT1
CSTA	0	2.35878	0.721	0.151	0	CD14_Mor	CSTA
NCF1	0	2.3369	0.738	0.225	0	CD14_Mor	NCF1
CSF3R	0	2.297847	0.629	0.096	0	CD14_Mor	CSF3R
CTSS	0	2.297673	0.91	0.436	0	CD14_Mor	CTSS
RETN	0	2.267477	0.21	0.024	0	CD14_Mor	RETN
CEBPD	0	2.266255	0.822	0.299	0	CD14_Mor	CEBPD
PLBD1	0	2.231485	0.569	0.098	0	CD14_Mor	PLBD1
TSPO	0	2.209804	0.884	0.499	0	CD14_Mor	TSPO
MS4A6A	0	2.195375	0.694	0.116	0	CD14_Mor	MS4A6A
CYBB	0	2.162505	0.759	0.21	0	CD14_Mor	CYBB
TYMP	0	2.103274	0.759	0.244	0	CD14_Mor	TYMP
FPR1	0	2.102141	0.59	0.097	0	CD14_Mor	FPR1
GCA	0	1.890224	0.555	0.164	0	CD14_Mor	GCA
TNFAIP2	0	1.856951	0.583	0.13	0	CD14_Mor	TNFAIP2
APLP2	0	1.8567	0.651	0.225	0	CD14_Mor	APLP2
MPEG1	0	1.822475	0.635	0.173	0	CD14_Mor	MPEG1
RGS2	0	1.804045	0.464	0.113	0	CD14_Mor	RGS2
TYROBP	0	1.777712	0.942	0.411	0	CD14_Mor	TYROBP
GRN	0	1.76264	0.686	0.239	0	CD14_Mor	GRN
TKT	0	1.74383	0.746	0.391	0	CD14_Mor	TKT
LGALS2	0	1.691161	0.455	0.079	0	CD14_Mor	LGALS2
CD302	0	1.688418	0.564	0.149	0	CD14_Mor	CD302
AIF1	0	1.665987	0.892	0.29	0	CD14_Mor	AIF1
FGL2	0	1.658278	0.663	0.219	0	CD14_Mor	FGL2
NCF2	0	1.645571	0.587	0.165	0	CD14_Mor	NCF2
S100A11	0	1.634778	0.922	0.603	0	CD14_Mor	S100A11
S100A4	0	1.626248	0.969	0.741	0	CD14_Mor	S100A4
LRP1	0	1.621135	0.497	0.097	0	CD14_Mor	LRP1
KCTD12	0	1.613365	0.494	0.102	0	CD14_Mor	KCTD12
BRI3	0	1.582013	0.73	0.337	0	CD14_Mor	BRI3
SERPINA1	0	1.581058	0.79	0.192	0	CD14_Mor	SERPINA1
CLEC12A	0	1.574185	0.516	0.138	0	CD14_Mor	CLEC12A
NAIP	0	1.570339	0.463	0.083	0	CD14_Mor	NAIP
TNFSF13B	0	1.569795	0.553	0.168	0	CD14_Mor	TNFSF13B
AMICA1	0	1.568972	0.595	0.209	0	CD14_Mor	AMICA1
IL17RA	0	1.546839	0.577	0.248	0	CD14_Mor	IL17RA
CD93	0	1.542218	0.423	0.071	0	CD14_Mor	CD93
PYCARD	0	1.528445	0.751	0.392	0	CD14_Mor	PYCARD

C4orf48	0	1.527656	0.636	0.294	0	CD14_Mor	C4orf48
RBP7	0	1.52079	0.342	0.044	0	CD14_Mor	RBP7
DUSP6	0	1.517837	0.545	0.16	0	CD14_Mor	DUSP6
LGALS1	0	1.503589	0.787	0.409	0	CD14_Mor	LGALS1
CDA	0	1.474442	0.377	0.067	0	CD14_Mor	CDA
SPI1	0	1.462737	0.727	0.214	0	CD14_Mor	SPI1
CAPG	0	1.437986	0.53	0.177	0	CD14_Mor	CAPG
IGSF6	0	1.425412	0.545	0.165	0	CD14_Mor	IGSF6
CYP1B1	0	1.403044	0.29	0.028	0	CD14_Mor	CYP1B1
RNF130	0	1.401786	0.62	0.244	0	CD14_Mor	RNF130
LGALS3	0	1.38911	0.643	0.267	0	CD14_Mor	LGALS3
MEGF9	0	1.383218	0.408	0.106	0	CD14_Mor	MEGF9
S100A10	0	1.381068	0.835	0.69	0	CD14_Mor	S100A10
TMEM176I	0	1.380482	0.429	0.102	0	CD14_Mor	TMEM176B
PTPRE	0	1.376351	0.574	0.251	0	CD14_Mor	PTPRE
DMXL2	0	1.374057	0.398	0.093	0	CD14_Mor	DMXL2
CTSD	0	1.365997	0.687	0.412	0	CD14_Mor	CTSD
SLC11A1	0	1.365956	0.449	0.116	0	CD14_Mor	SLC11A1
CPVL	0	1.365567	0.524	0.153	0	CD14_Mor	CPVL
BLVRB	0	1.364134	0.534	0.193	0	CD14_Mor	BLVRB
LRRK2	0	1.363814	0.37	0.094	0	CD14_Mor	LRRK2
ASGR1	0	1.343632	0.388	0.059	0	CD14_Mor	ASGR1
DUSP1	0	1.338483	0.493	0.206	0	CD14_Mor	DUSP1
NUP214	0	1.328266	0.475	0.175	0	CD14_Mor	NUP214
PGD	0	1.321129	0.604	0.25	0	CD14_Mor	PGD
GSTP1	0	1.305163	0.78	0.54	0	CD14_Mor	GSTP1
LTA4H	0	1.297011	0.565	0.304	0	CD14_Mor	LTA4H
CTSB	0	1.293711	0.565	0.262	0	CD14_Mor	CTSB
ATP6V0B	0	1.291725	0.658	0.391	0	CD14_Mor	ATP6V0B
CLEC7A	0	1.287185	0.457	0.135	0	CD14_Mor	CLEC7A
SDCBP	0	1.276144	0.572	0.305	0	CD14_Mor	SDCBP
GABARAP	0	1.26128	0.872	0.758	0	CD14_Mor	GABARAP
EVI2B	0	1.25993	0.571	0.335	0	CD14_Mor	EVI2B
VIM	0	1.243973	0.919	0.735	0	CD14_Mor	VIM
TMEM176/	0	1.242345	0.335	0.067	0	CD14_Mor	TMEM176A
IRAK3	0	1.231614	0.379	0.108	0	CD14_Mor	IRAK3
CST3	0	1.231381	0.901	0.388	0	CD14_Mor	CST3
CFD	0	1.226194	0.604	0.171	0	CD14_Mor	CFD
11-Mar	0	1.22454	0.439	0.149	0	CD14_Mor	1-Mar
GAS7	0	1.219345	0.342	0.069	0	CD14_Mor	GAS7
POU2F2	0	1.21693	0.556	0.254	0	CD14_Mor	POU2F2
MT-ND1	0	1.214786	0.969	0.89	0	CD14_Mor	MT-ND1
FTL	0	1.212678	0.988	0.971	0	CD14_Mor	FTL
CFP	0	1.205915	0.546	0.185	0	CD14_Mor	CFP
ANXA5	0	1.202517	0.711	0.379	0	CD14_Mor	ANXA5
TIMP2	0	1.199748	0.367	0.085	0	CD14_Mor	TIMP2
SERPINB1	0	1.195352	0.678	0.462	0	CD14_Mor	SERPINB1
HEBP2	0	1.190194	0.497	0.272	0	CD14_Mor	HEBP2
AGTRAP	0	1.188756	0.528	0.253	0	CD14_Mor	AGTRAP
CTSH	0	1.18185	0.474	0.179	0	CD14_Mor	CTSH
FCGRT	0	1.179638	0.518	0.218	0	CD14_Mor	FCGRT
MYO1F	0	1.178605	0.592	0.338	0	CD14_Mor	MYO1F
CKAP4	0	1.1699	0.267	0.048	0	CD14_Mor	CKAP4
LAMTOR4	0	1.163237	0.738	0.541	0	CD14_Mor	LAMTOR4
GRINA	0	1.163024	0.463	0.223	0	CD14_Mor	GRINA
OGFRL1	0	1.162715	0.38	0.11	0	CD14_Mor	OGFRL1
PSAP	0	1.153655	0.851	0.549	0	CD14_Mor	PSAP
FCGR1A	0	1.152531	0.293	0.032	0	CD14_Mor	FCGR1A

HCK	0	1.151604	0.54	0.176	0	CD14_Mor	HCK
PRAM1	0	1.148084	0.39	0.123	0	CD14_Mor	PRAM1
CD44	0	1.147107	0.618	0.429	0	CD14_Mor	CD44
AP1S2	0	1.142781	0.633	0.353	0	CD14_Mor	AP1S2
VMP1	0	1.140701	0.443	0.214	0	CD14_Mor	VMP1
MCL1	0	1.136371	0.53	0.332	0	CD14_Mor	MCL1
PAK1	0	1.136234	0.464	0.187	0	CD14_Mor	PAK1
PTAFR	0	1.130932	0.337	0.056	0	CD14_Mor	PTAFR
ANPEP	0	1.122283	0.259	0.036	0	CD14_Mor	ANPEP
MGST1	0	1.120503	0.304	0.038	0	CD14_Mor	MGST1
MSRB1	0	1.114948	0.309	0.093	0	CD14_Mor	MSRB1
TNFSF10	0	1.112653	0.513	0.276	0	CD14_Mor	TNFSF10
CD36	0	1.112407	0.547	0.147	0	CD14_Mor	CD36
ZNF106	0	1.111859	0.489	0.246	0	CD14_Mor	ZNF106
LILRA5	0	1.100198	0.42	0.129	0	CD14_Mor	LILRA5
AHNAK	0	1.099387	0.683	0.503	0	CD14_Mor	AHNAK
SNX10	0	1.09567	0.448	0.205	0	CD14_Mor	SNX10
CD300E	0	1.092408	0.351	0.098	0	CD14_Mor	CD300E
RP11-114(	0	1.081283	0.268	0.035	0	CD14_Mor	RP11-1143G9.4
FBXL5	0	1.078244	0.471	0.258	0	CD14_Mor	FBXL5
CREB5	0	1.076935	0.264	0.036	0	CD14_Mor	CREB5
ITGAM	0	1.068044	0.354	0.098	0	CD14_Mor	ITGAM
RAB11FIP1	0	1.064062	0.382	0.18	0	CD14_Mor	RAB11FIP1
GLIPR2	0	1.063551	0.46	0.236	0	CD14_Mor	GLIPR2
SYK	0	1.0619	0.43	0.187	0	CD14_Mor	SYK
TGFBI	0	1.057544	0.365	0.113	0	CD14_Mor	TGFBI
NUMB	0	1.054656	0.38	0.157	0	CD14_Mor	NUMB
C19orf59	0	1.053411	0.205	0.018	0	CD14_Mor	C19orf59
CAST	0	1.046012	0.668	0.481	0	CD14_Mor	CAST
IQGAP1	0	1.036502	0.631	0.424	0	CD14_Mor	IQGAP1
FAM45A	0	1.03496	0.39	0.191	0	CD14_Mor	FAM45A
LILRB2	0	1.029903	0.498	0.16	0	CD14_Mor	LILRB2
TCEB2	0	1.02965	0.799	0.691	0	CD14_Mor	TCEB2
LY861	0	1.029578	0.382	0.126	0	CD14_Mor	LY86
LILRB3	0	1.026445	0.307	0.098	0	CD14_Mor	LILRB3
TNFRSF1B	0	1.024376	0.592	0.308	0	CD14_Mor	TNFRSF1B
BASP11	0	1.02435	0.211	0.078	0	CD14_Mor	BASP1
SULT1A1	0	1.023657	0.321	0.091	0	CD14_Mor	SULT1A1
NFAM1	0	1.023441	0.303	0.067	0	CD14_Mor	NFAM1
RNASE2	0	1.022995	0.228	0.022	0	CD14_Mor	RNASE2
PPT1	0	1.019928	0.425	0.212	0	CD14_Mor	PPT1
GLRX	0	1.016988	0.539	0.352	0	CD14_Mor	GLRX
NAGK	0	1.011664	0.448	0.22	0	CD14_Mor	NAGK
PLEC	0	1.010784	0.362	0.155	0	CD14_Mor	PLEC
FGR	0	1.009468	0.581	0.306	0	CD14_Mor	FGR
IFNGR2	0	1.007638	0.436	0.193	0	CD14_Mor	IFNGR2
TCIRG1	0	1.006371	0.44	0.22	0	CD14_Mor	TCIRG1
ATP5E	0	1.004015	0.97	0.916	0	CD14_Mor	ATP5E
CTSZ	0	1.00192	0.561	0.267	0	CD14_Mor	CTSZ
TLR4	0	0.992191	0.296	0.08	0	CD14_Mor	TLR4
RXRA	0	0.991686	0.347	0.112	0	CD14_Mor	RXRA
RNASE6	0	0.991133	0.353	0.089	0	CD14_Mor	RNASE6
MARCKS	0	0.990898	0.305	0.124	0	CD14_Mor	MARCKS
HIF1A	0	0.990427	0.372	0.206	0	CD14_Mor	HIF1A
METTL9	0	0.988414	0.441	0.267	0	CD14_Mor	METTL9
C19orf38	0	0.987573	0.382	0.135	0	CD14_Mor	C19orf38
CECR1	0	0.986132	0.422	0.203	0	CD14_Mor	CECR1
CD163	0	0.983186	0.267	0.023	0	CD14_Mor	CD163

SAMHD1	0	0.982978	0.68	0.489	0	CD14_Mor	SAMHD1
C10orf54	0	0.979382	0.615	0.391	0	CD14_Mor	C10orf54
NCF4	0	0.976867	0.283	0.09	0	CD14_Mor	NCF4
CNPY3	0	0.975231	0.567	0.371	0	CD14_Mor	CNPY3
FAM200B	0	0.969958	0.359	0.161	0	CD14_Mor	FAM200B
LST1	0	0.965445	0.826	0.242	0	CD14_Mor	LST1
CLEC4A	0	0.964969	0.313	0.096	0	CD14_Mor	CLEC4A
BST1	0	0.964001	0.265	0.044	0	CD14_Mor	BST1
SIRPA	0	0.957548	0.259	0.034	0	CD14_Mor	SIRPA
STAB1	0	0.955703	0.263	0.02	0	CD14_Mor	STAB1
HK3	0	0.954142	0.288	0.082	0	CD14_Mor	HK3
ADRBK1	0	0.95285	0.402	0.198	0	CD14_Mor	ADRBK1
EHBP1L1	0	0.947552	0.345	0.143	0	CD14_Mor	EHBP1L1
ASGR2	0	0.94519	0.215	0.021	0	CD14_Mor	ASGR2
ATP6V0D1	0	0.941016	0.492	0.299	0	CD14_Mor	ATP6V0D1
EMILIN2	0	0.940416	0.324	0.103	0	CD14_Mor	EMILIN2
ZNF385A	0	0.937168	0.342	0.113	0	CD14_Mor	ZNF385A
LYST	0	0.937119	0.463	0.238	0	CD14_Mor	LYST
CRTAP	0	0.935594	0.424	0.203	0	CD14_Mor	CRTAP
RASSF2	0	0.931801	0.335	0.136	0	CD14_Mor	RASSF2
ACAP2	0	0.930651	0.562	0.405	0	CD14_Mor	ACAP2
CYP27A1	0	0.926597	0.19	0.008	0	CD14_Mor	CYP27A1
HMGB2	0	0.923199	0.39	0.239	0	CD14_Mor	HMGB2
ANXA2	0	0.922261	0.613	0.393	0	CD14_Mor	ANXA2
KCNE3	0	0.921234	0.308	0.084	0	CD14_Mor	KCNE3
PLXDC2	0	0.920369	0.364	0.119	0	CD14_Mor	PLXDC2
ALDH2	0	0.920214	0.323	0.094	0	CD14_Mor	ALDH2
BACH1	0	0.919746	0.336	0.134	0	CD14_Mor	BACH1
VNN2	0	0.918184	0.222	0.058	0	CD14_Mor	VNN2
GRB2	0	0.914262	0.6	0.42	0	CD14_Mor	GRB2
ZEB2	0	0.913762	0.522	0.293	0	CD14_Mor	ZEB2
CCDC88A	0	0.909856	0.375	0.146	0	CD14_Mor	CCDC88A
RAC1	0	0.90848	0.749	0.547	0	CD14_Mor	RAC1
PGLS	0	0.907863	0.58	0.408	0	CD14_Mor	PGLS
CLEC4E	0	0.906268	0.222	0.01	0	CD14_Mor	CLEC4E
TLR2	0	0.902766	0.26	0.056	0	CD14_Mor	TLR2
FES	0	0.899794	0.291	0.093	0	CD14_Mor	FES
LILRA2	0	0.894913	0.321	0.111	0	CD14_Mor	LILRA2
GNAI2	0	0.894253	0.74	0.601	0	CD14_Mor	GNAI2
IFI30	0	0.893898	0.391	0.155	0	CD14_Mor	IFI30
SLC16A3	0	0.893799	0.344	0.168	0	CD14_Mor	SLC16A3
UQCRQ	0	0.893685	0.611	0.496	0	CD14_Mor	UQCRQ
JUNB	0	0.891629	0.541	0.387	0	CD14_Mor	JUNB
UBE2D1	0	0.891091	0.332	0.158	0	CD14_Mor	UBE2D1
SLC7A7	0	0.890912	0.375	0.121	0	CD14_Mor	SLC7A7
H2AFY	0	0.886652	0.525	0.34	0	CD14_Mor	H2AFY
GAPDH	0	0.876488	0.938	0.904	0	CD14_Mor	GAPDH
CKLF	0	0.875742	0.49	0.353	0	CD14_Mor	CKLF
NDUFB1	0	0.875586	0.631	0.516	0	CD14_Mor	NDUFB1
CEBPB	0	0.875302	0.511	0.294	0	CD14_Mor	CEBPB
ENTPD1	0	0.87491	0.225	0.051	0	CD14_Mor	ENTPD1
NACC2	0	0.871188	0.249	0.067	0	CD14_Mor	NACC2
CD33	0	0.868058	0.306	0.095	0	CD14_Mor	CD33
POLE4	0	0.867792	0.406	0.252	0	CD14_Mor	POLE4
C5AR1	0	0.867337	0.282	0.1	0	CD14_Mor	C5AR1
GLIPR1	0	0.86342	0.52	0.35	0	CD14_Mor	GLIPR1
AQP9	0	0.858137	0.17	0.017	0	CD14_Mor	AQP9
KIAA0930	0	0.85582	0.286	0.099	0	CD14_Mor	KIAA0930

OSCAR	0	0.85356	0.236	0.058	0	CD14_Mor	OSCAR
P2RY13	0	0.85017	0.276	0.088	0	CD14_Mor	P2RY13
ALOX51	0	0.848688	0.291	0.12	0	CD14_Mor	ALOX5
GNS	0	0.846556	0.334	0.141	0	CD14_Mor	GNS
HNMT	0	0.845086	0.267	0.065	0	CD14_Mor	HNMT
AGTPBP1	0	0.844239	0.307	0.155	0	CD14_Mor	AGTPBP1
DPYSL2	0	0.842677	0.31	0.122	0	CD14_Mor	DPYSL2
KIAA1598	0	0.841668	0.254	0.067	0	CD14_Mor	KIAA1598
FAM198B	0	0.838241	0.226	0.031	0	CD14_Mor	FAM198B
PAK2	0	0.836181	0.547	0.426	0	CD14_Mor	PAK2
NBPF10	0	0.835434	0.334	0.155	0	CD14_Mor	NBPF10
MEF2C1	0	0.83213	0.438	0.219	0	CD14_Mor	MEF2C
APOBR	0	0.8313	0.269	0.097	0	CD14_Mor	APOBR
RAB3D	0	0.830412	0.237	0.049	0	CD14_Mor	RAB3D
NADK	0	0.823395	0.303	0.144	0	CD14_Mor	NADK
RAB27A	0	0.819838	0.313	0.165	0	CD14_Mor	RAB27A
ACSL1	0	0.817423	0.179	0.041	0	CD14_Mor	ACSL1
BAZ2B	0	0.815718	0.262	0.107	0	CD14_Mor	BAZ2B
RBM47	0	0.813856	0.235	0.064	0	CD14_Mor	RBM47
ANXA1	0	0.811337	0.661	0.494	0	CD14_Mor	ANXA1
SERP1	0	0.811057	0.738	0.645	0	CD14_Mor	SERP1
AP2S1	0	0.808549	0.639	0.448	0	CD14_Mor	AP2S1
LY96	0	0.808082	0.274	0.104	0	CD14_Mor	LY96
TMEM167/	0	0.805435	0.36	0.197	0	CD14_Mor	TMEM167A
USP3	0	0.804453	0.389	0.246	0	CD14_Mor	USP3
TALDO1	0	0.804432	0.758	0.544	0	CD14_Mor	TALDO1
RAB31	0	0.803656	0.544	0.224	0	CD14_Mor	RAB31
ATP6V1B2	0	0.803058	0.4	0.204	0	CD14_Mor	ATP6V1B2
EVI2A	0	0.79667	0.331	0.179	0	CD14_Mor	EVI2A
COX8A	0	0.795254	0.679	0.582	0	CD14_Mor	COX8A
PLSCR1	0	0.795047	0.281	0.136	0	CD14_Mor	PLSCR1
SCPEP1	0	0.79488	0.302	0.133	0	CD14_Mor	SCPEP1
QKI	0	0.794542	0.297	0.121	0	CD14_Mor	QKI
PILRA	0	0.786771	0.384	0.146	0	CD14_Mor	PILRA
CPPED1	0	0.784583	0.34	0.151	0	CD14_Mor	CPPED1
SULF2	0	0.783433	0.269	0.102	0	CD14_Mor	SULF2
SMCO4	0	0.782298	0.31	0.137	0	CD14_Mor	SMCO4
UBE2R2	0	0.781764	0.309	0.142	0	CD14_Mor	UBE2R2
NPC2	0	0.777253	0.603	0.367	0	CD14_Mor	NPC2
MT-ND4	0	0.775182	0.946	0.91	0	CD14_Mor	MT-ND4
THEMIS2	0	0.771988	0.376	0.207	0	CD14_Mor	THEMIS2
SLC43A2	0	0.771747	0.243	0.073	0	CD14_Mor	SLC43A2
CCR2	0	0.771507	0.251	0.058	0	CD14_Mor	CCR2
LCP1	0	0.767766	0.735	0.604	0	CD14_Mor	LCP1
DYSF	0	0.765669	0.147	0.012	0	CD14_Mor	DYSF
TMEM205	0	0.76445	0.29	0.124	0	CD14_Mor	TMEM205
HRH2	0	0.763602	0.268	0.119	0	CD14_Mor	HRH2
SGK1	0	0.763528	0.184	0.035	0	CD14_Mor	SGK1
NLRP12	0	0.762156	0.179	0.02	0	CD14_Mor	NLRP12
CCNY	0	0.755382	0.273	0.087	0	CD14_Mor	CCNY
SLC8A1	0	0.751776	0.243	0.092	0	CD14_Mor	SLC8A1
LRRFIP1	0	0.751561	0.679	0.559	0	CD14_Mor	LRRFIP1
MAP3K3	0	0.749648	0.246	0.085	0	CD14_Mor	MAP3K3
MTDH	0	0.749263	0.564	0.459	0	CD14_Mor	MTDH
PDXK	0	0.745658	0.273	0.122	0	CD14_Mor	PDXK
GNG5	0	0.745475	0.659	0.558	0	CD14_Mor	GNG5
CR1	0	0.745243	0.172	0.034	0	CD14_Mor	CR1
DICER1	0	0.744909	0.313	0.16	0	CD14_Mor	DICER1

LINC00937	0	0.743953	0.164	0.009	0	CD14_Mor	LINC00937
SMARCD3	0	0.743008	0.206	0.034	0	CD14_Mor	SMARCD3
MT-ATP6	0	0.74244	0.955	0.901	0	CD14_Mor	MT-ATP6
CCR1	0	0.741554	0.221	0.054	0	CD14_Mor	CCR1
TET2	0	0.738332	0.254	0.105	0	CD14_Mor	TET2
PLAUR	0	0.73642	0.253	0.106	0	CD14_Mor	PLAUR
EIF4EBP1	0	0.736243	0.292	0.133	0	CD14_Mor	EIF4EBP1
ATP5G2	0	0.735592	0.836	0.772	0	CD14_Mor	ATP5G2
LTB4R	0	0.733872	0.164	0.022	0	CD14_Mor	LTB4R
RHOG	0	0.729733	0.609	0.488	0	CD14_Mor	RHOG
ARHGAP26	0	0.72786	0.242	0.092	0	CD14_Mor	ARHGAP26
ARRB2	0	0.725764	0.512	0.377	0	CD14_Mor	ARRB2
ATP5I	0	0.714498	0.668	0.607	0	CD14_Mor	ATP5I
1-Mar	0	0.712174	0.14	0.006	0	CD14_Mor	1-Mar
ARAP1	0	0.71196	0.291	0.134	0	CD14_Mor	ARAP1
ODF3B	0	0.711528	0.271	0.12	0	CD14_Mor	ODF3B
APAF1	0	0.709744	0.239	0.097	0	CD14_Mor	APAF1
STXBP2	0	0.706924	0.619	0.378	0	CD14_Mor	STXBP2
LILRA6	0	0.706558	0.208	0.053	0	CD14_Mor	LILRA6
HSBP1	0	0.705549	0.475	0.308	0	CD14_Mor	HSBP1
RASSF4	0	0.703388	0.266	0.105	0	CD14_Mor	RASSF4
PLXNB2	0	0.702532	0.267	0.101	0	CD14_Mor	PLXNB2
ACTR2	0	0.701357	0.607	0.505	0	CD14_Mor	ACTR2
PPP1R9B	0	0.700712	0.254	0.104	0	CD14_Mor	PPP1R9B
GM2A	0	0.700015	0.203	0.056	0	CD14_Mor	GM2A
LGALS9	0	0.699202	0.347	0.189	0	CD14_Mor	LGALS9
DOK3	0	0.695525	0.241	0.073	0	CD14_Mor	DOK3
CHP1	0	0.694399	0.25	0.098	0	CD14_Mor	CHP1
GLT1D1	0	0.692177	0.16	0.018	0	CD14_Mor	GLT1D1
LACTB	0	0.691357	0.285	0.142	0	CD14_Mor	LACTB
GNB2	0	0.687394	0.611	0.513	0	CD14_Mor	GNB2
EIF4A1	0	0.682512	0.66	0.595	0	CD14_Mor	EIF4A1
ITGB2	0	0.68174	0.735	0.604	0	CD14_Mor	ITGB2
LTBR	0	0.679432	0.222	0.073	0	CD14_Mor	LTBR
TFEC	0	0.678265	0.183	0.029	0	CD14_Mor	TFEC
IRF2BP2	0	0.677327	0.27	0.13	0	CD14_Mor	IRF2BP2
CRISPLD2	0	0.674739	0.152	0.006	0	CD14_Mor	CRISPLD2
UBR4	0	0.674139	0.278	0.132	0	CD14_Mor	UBR4
ATP6V0A1	0	0.669259	0.171	0.038	0	CD14_Mor	ATP6V0A1
BLVRA	0	0.66768	0.305	0.15	0	CD14_Mor	BLVRA
FOLR3	0	0.667105	0.119	0.004	0	CD14_Mor	FOLR3
LMO2	0	0.667103	0.265	0.11	0	CD14_Mor	LMO2
MT-ND2	0	0.667056	0.926	0.905	0	CD14_Mor	MT-ND2
MEFV	0	0.666495	0.2	0.058	0	CD14_Mor	MEFV
ZNF467	0	0.660852	0.188	0.041	0	CD14_Mor	ZNF467
IER3	0	0.658886	0.169	0.034	0	CD14_Mor	IER3
DSC2	0	0.651668	0.139	0.01	0	CD14_Mor	DSC2
SIRPB2	0	0.647646	0.193	0.045	0	CD14_Mor	SIRPB2
PYGL	0	0.646873	0.275	0.087	0	CD14_Mor	PYGL
PRKCD	0	0.646265	0.327	0.163	0	CD14_Mor	PRKCD
RASGRP4	0	0.644779	0.198	0.066	0	CD14_Mor	RASGRP4
CD86	0	0.638867	0.267	0.119	0	CD14_Mor	CD86
GMFG	0	0.638443	0.725	0.683	0	CD14_Mor	GMFG
EFHD2	0	0.637776	0.667	0.45	0	CD14_Mor	EFHD2
MAFB	0	0.636265	0.289	0.12	0	CD14_Mor	MAFB
SKAP2	0	0.634645	0.413	0.243	0	CD14_Mor	SKAP2
SIRPB1	0	0.634562	0.195	0.062	0	CD14_Mor	SIRPB1
RHOQ	0	0.633466	0.215	0.075	0	CD14_Mor	RHOQ

CYBA	0	0.629714	0.861	0.754	0	CD14_Mor	CYBA
LAPTM51	0	0.628236	0.769	0.731	0	CD14_Mor	LAPTM5
CD1D	0	0.627188	0.202	0.058	0	CD14_Mor	CD1D
STEAP4	0	0.626596	0.109	0.004	0	CD14_Mor	STEAP4
FYB	0	0.624809	0.738	0.658	0	CD14_Mor	FYB
LRRC25	0	0.623775	0.327	0.147	0	CD14_Mor	LRRC25
COTL1	0	0.620683	0.854	0.558	0	CD14_Mor	COTL1
IMPA2	0	0.618764	0.209	0.074	0	CD14_Mor	IMPA2
TMSB10	0	0.615332	0.956	0.851	0	CD14_Mor	TMSB10
SLC15A3	0	0.612781	0.191	0.058	0	CD14_Mor	SLC15A3
IL1RN	0	0.599726	0.144	0.024	0	CD14_Mor	IL1RN
TLR8	0	0.591665	0.171	0.043	0	CD14_Mor	TLR8
IL13RA1	0	0.586595	0.185	0.049	0	CD14_Mor	IL13RA1
TBXAS1	0	0.585866	0.376	0.196	0	CD14_Mor	TBXAS1
AGO4	0	0.584957	0.161	0.044	0	CD14_Mor	AGO4
FCGR1B	0	0.5848	0.139	0.022	0	CD14_Mor	FCGR1B
CES1	0	0.580042	0.149	0.026	0	CD14_Mor	CES1
NRG1	0	0.578649	0.147	0.008	0	CD14_Mor	NRG1
TPP1	0	0.57588	0.409	0.252	0	CD14_Mor	TPP1
CYFIP1	0	0.575081	0.201	0.063	0	CD14_Mor	CYFIP1
QPCT	0	0.574734	0.138	0.014	0	CD14_Mor	QPCT
ARPC3	0	0.572291	0.82	0.777	0	CD14_Mor	ARPC3
KIF13A	0	0.568077	0.158	0.033	0	CD14_Mor	KIF13A
TMEM170I	0	0.565003	0.14	0.027	0	CD14_Mor	TMEM170B
CREG1	0	0.562877	0.29	0.115	0	CD14_Mor	CREG1
HOMER3	0	0.557265	0.137	0.012	0	CD14_Mor	HOMER3
NLRP3	0	0.548462	0.147	0.033	0	CD14_Mor	NLRP3
RPL7	0	0.541806	0.819	0.76	0	CD14_Mor	RPL7
CLEC4D	0	0.531881	0.106	0.004	0	CD14_Mor	CLEC4D
FCGR2A	0	0.51859	0.339	0.135	0	CD14_Mor	FCGR2A
APBB3	0	0.512153	0.13	0.021	0	CD14_Mor	APBB3
ALDH1A1	0	0.510614	0.109	0.008	0	CD14_Mor	ALDH1A1
AL627309.	0	0.509544	0.101	0.011	0	CD14_Mor	AL627309.1
F5	0	0.501196	0.113	0.012	0	CD14_Mor	F5
CLMN	0	0.495342	0.136	0.022	0	CD14_Mor	CLMN
SOD2	0	0.487534	0.466	0.293	0	CD14_Mor	SOD2
SLC25A37	0	0.482543	0.26	0.115	0	CD14_Mor	SLC25A37
PLEKHO1	0	0.46741	0.394	0.234	0	CD14_Mor	PLEKHO1
COX4I1	0	0.460192	0.836	0.811	0	CD14_Mor	COX4I1
NFE2	0	0.444418	0.302	0.093	0	CD14_Mor	NFE2
FCER1G	0	0.438706	0.863	0.487	0	CD14_Mor	FCER1G
PFDN5	0	0.423144	0.818	0.791	0	CD14_Mor	PFDN5
SERF2	0	0.374196	0.951	0.956	0	CD14_Mor	SERF2
H3F3A	0	0.25545	0.941	0.942	0	CD14_Mor	H3F3A
SECTM1	#######	0.617513	0.229	0.096	#######	CD14_Mor	SECTM1
PRELID1	#######	0.648356	0.602	0.501	#######	CD14_Mor	PRELID1
ARHGEF4(	#######	0.443349	0.108	0.016	#######	CD14_Mor	ARHGEF40
GNAQ	#######	0.516921	0.248	0.11	#######	CD14_Mor	GNAQ
PARVG	#######	0.77362	0.401	0.257	#######	CD14_Mor	PARVG
ARID3A	#######	0.625143	0.19	0.068	#######	CD14_Mor	ARID3A
PLXND1	#######	0.452564	0.149	0.04	#######	CD14_Mor	PLXND1
UBXN11	#######	0.466786	0.287	0.139	#######	CD14_Mor	UBXN11
JUND	#######	0.78674	0.321	0.181	#######	CD14_Mor	JUND
RPS24	#######	0.3349	0.925	0.83	#######	CD14_Mor	RPS24
PHC2	#######	0.644246	0.165	0.051	#######	CD14_Mor	PHC2
UQCR11.1	#######	0.493565	0.758	0.751	#######	CD14_Mor	UQCR11.1
LILRB1	#######	0.657269	0.317	0.164	#######	CD14_Mor	LILRB1
ADRBK2	#######	0.608483	0.208	0.083	#######	CD14_Mor	ADRBK2

FAM101B	#######	0.833296	0.171	0.055	#######	CD14_Mor	FAM101B
INPPL1	#######	0.465795	0.145	0.038	#######	CD14_Mor	INPPL1
TWF2	#######	0.552684	0.167	0.052	#######	CD14_Mor	TWF2
HLA-DMB	#######	0.653847	0.303	0.157	#######	CD14_Mor	HLA-DMB
CDC42EP3	#######	0.922907	0.416	0.282	#######	CD14_Mor	CDC42EP3
CSF2RA	#######	0.567378	0.184	0.067	#######	CD14_Mor	CSF2RA
RASSF3	#######	0.722898	0.246	0.113	#######	CD14_Mor	RASSF3
C14orf2	#######	0.557055	0.621	0.561	#######	CD14_Mor	C14orf2
ATP6V1A	#######	0.588313	0.25	0.116	#######	CD14_Mor	ATP6V1A
ROMO1	#######	0.820259	0.539	0.432	#######	CD14_Mor	ROMO1
MARCO	#######	0.430712	0.133	0.031	#######	CD14_Mor	MARCO
CD4	#######	0.765172	0.349	0.21	#######	CD14_Mor	CD4
NUDT16	#######	0.630442	0.271	0.135	#######	CD14_Mor	NUDT16
TXN	#######	0.749721	0.507	0.383	#######	CD14_Mor	TXN
TRAPPC5	#######	0.620847	0.467	0.337	#######	CD14_Mor	TRAPPC5
VAMP8	#######	0.629189	0.615	0.55	#######	CD14_Mor	VAMP8
TK2	#######	0.569324	0.179	0.062	#######	CD14_Mor	TK2
IRS2	#######	0.550958	0.131	0.03	#######	CD14_Mor	IRS2
EVI5	#######	0.56649	0.189	0.071	#######	CD14_Mor	EVI5
SMAP2	#######	0.764793	0.548	0.448	#######	CD14_Mor	SMAP2
SLCO3A1	#######	0.678395	0.239	0.109	#######	CD14_Mor	SLCO3A1
FPR2	#######	0.552872	0.119	0.024	#######	CD14_Mor	FPR2
TRPS1	#######	0.537881	0.15	0.043	#######	CD14_Mor	TRPS1
PADI4	#######	0.500023	0.195	0.068	#######	CD14_Mor	PADI4
METTL7A	#######	0.579931	0.235	0.106	#######	CD14_Mor	METTL7A
CARD16	#######	0.628785	0.588	0.473	#######	CD14_Mor	CARD16
KDM6B	#######	0.664343	0.186	0.068	#######	CD14_Mor	KDM6B
C9orf72	#######	0.630606	0.198	0.079	#######	CD14_Mor	C9orf72
RTN3	#######	0.427188	0.415	0.265	#######	CD14_Mor	RTN3
RBPJ	#######	0.756412	0.337	0.199	#######	CD14_Mor	RBPJ
ADAP2	#######	0.562113	0.201	0.081	#######	CD14_Mor	ADAP2
PKN2	#######	0.85452	0.293	0.161	#######	CD14_Mor	PKN2
CCDC88B	#######	0.7298	0.226	0.101	#######	CD14_Mor	CCDC88B
ETV6	#######	0.688341	0.283	0.149	#######	CD14_Mor	ETV6
GAA	#######	0.55814	0.185	0.07	#######	CD14_Mor	GAA
SORL1	#######	0.827684	0.364	0.226	#######	CD14_Mor	SORL1
IL6R	#######	0.689311	0.27	0.138	#######	CD14_Mor	IL6R
TRMT1	#######	0.702351	0.282	0.152	#######	CD14_Mor	TRMT1
FKBP15	#######	0.685076	0.264	0.135	#######	CD14_Mor	FKBP15
CTDNEP1	#######	0.596439	0.227	0.103	#######	CD14_Mor	CTDNEP1
FAM120A	#######	0.663854	0.305	0.172	#######	CD14_Mor	FAM120A
CLTC	#######	0.691949	0.357	0.223	#######	CD14_Mor	CLTC
SEMA4A	#######	0.616872	0.18	0.068	#######	CD14_Mor	SEMA4A
PLIN3	#######	0.587185	0.247	0.12	#######	CD14_Mor	PLIN3
MBOAT7	#######	0.636945	0.231	0.108	#######	CD14_Mor	MBOAT7
PLP2	#######	0.694662	0.438	0.306	#######	CD14_Mor	PLP2
CSF2RB	#######	0.557825	0.142	0.042	#######	CD14_Mor	CSF2RB
DPYD	#######	0.712746	0.383	0.252	#######	CD14_Mor	DPYD
CHST15	#######	0.525323	0.175	0.064	#######	CD14_Mor	CHST15
TMEM154	#######	0.820168	0.298	0.169	#######	CD14_Mor	TMEM154
DKFZP761	#######	0.391785	0.109	0.022	#######	CD14_Mor	DKFZP761J1410
HLA-DQB	#######	0.319804	0.436	0.265	#######	CD14_Mor	HLA-DQB1
WDFY3	#######	0.472733	0.112	0.024	#######	CD14_Mor	WDFY3
CAPNS1	#######	0.565171	0.424	0.289	#######	CD14_Mor	CAPNS1
RNASEK	#######	0.635486	0.655	0.614	#######	CD14_Mor	RNASEK
RP11-295	#######	0.559333	0.118	0.027	#######	CD14_Mor	RP11-295G20.2
LAT21	#######	0.628295	0.294	0.159	#######	CD14_Mor	LAT2
NAPRT1	#######	0.789759	0.266	0.142	#######	CD14_Mor	NAPRT1

SPTLC2	#######	0.652784	0.276	0.148	#######	CD14_Mor	SPTLC2
TTC7A	#######	0.511709	0.176	0.067	#######	CD14_Mor	TTC7A
TLR5	#######	0.453168	0.123	0.031	#######	CD14_Mor	TLR5
MYADM	#######	0.707129	0.32	0.191	#######	CD14_Mor	MYADM
CD300LF	#######	0.600297	0.187	0.075	#######	CD14_Mor	CD300LF
TNRC18	#######	0.514576	0.159	0.055	#######	CD14_Mor	TNRC18
PICALM	#######	0.673676	0.293	0.165	#######	CD14_Mor	PICALM
STAT2	#######	0.670972	0.248	0.126	#######	CD14_Mor	STAT2
MLXIP	#######	0.516488	0.211	0.092	#######	CD14_Mor	MLXIP
PLXNC1	#######	0.672976	0.224	0.106	#######	CD14_Mor	PLXNC1
ATP5H	#######	0.703026	0.563	0.482	#######	CD14_Mor	ATP5H
C1orf162	#######	0.611301	0.53	0.395	#######	CD14_Mor	C1orf162
ARID1A	#######	0.680121	0.311	0.183	#######	CD14_Mor	ARID1A
TPCN1	#######	0.402523	0.129	0.035	#######	CD14_Mor	TPCN1
LAMP2	#######	0.641678	0.26	0.135	#######	CD14_Mor	LAMP2
LFNG	#######	0.626705	0.24	0.117	#######	CD14_Mor	LFNG
GTF2I	#######	0.700593	0.35	0.221	#######	CD14_Mor	GTF2I
MLKL	#######	0.508197	0.144	0.045	#######	CD14_Mor	MLKL
SNX27	#######	0.607138	0.256	0.133	#######	CD14_Mor	SNX27
GIMAP8	#######	0.634128	0.274	0.147	#######	CD14_Mor	GIMAP8
NPL	#######	0.464584	0.146	0.047	#######	CD14_Mor	NPL
ATG16L2	#######	0.713267	0.287	0.162	#######	CD14_Mor	ATG16L2
TYK2	#######	0.599307	0.204	0.091	#######	CD14_Mor	TYK2
ALDH3B1	#######	0.524415	0.17	0.065	#######	CD14_Mor	ALDH3B1
DUSP3	#######	0.404279	0.161	0.058	#######	CD14_Mor	DUSP3
SQRDL	#######	0.725611	0.287	0.164	#######	CD14_Mor	SQRDL
HLA-DMA	#######	0.50995	0.368	0.22	#######	CD14_Mor	HLA-DMA
ZSWIM6	#######	0.435605	0.136	0.042	#######	CD14_Mor	ZSWIM6
CD300LB	#######	0.400026	0.108	0.024	#######	CD14_Mor	CD300LB
DEK	#######	0.692435	0.551	0.467	#######	CD14_Mor	DEK
NOTCH2	#######	0.623954	0.356	0.227	#######	CD14_Mor	NOTCH2
AHR	#######	0.501409	0.192	0.081	#######	CD14_Mor	AHR
PSTPIP1	#######	0.703148	0.298	0.172	#######	CD14_Mor	PSTPIP1
COLGALT1	#######	0.540694	0.175	0.071	#######	CD14_Mor	COLGALT1
COX5B	#######	0.565821	0.658	0.63	#######	CD14_Mor	COX5B
ASAH1	#######	0.253176	0.49	0.344	#######	CD14_Mor	ASAH1
HIPK3	#######	0.555831	0.216	0.102	#######	CD14_Mor	HIPK3
RP11-108I	#######	0.59111	0.254	0.134	#######	CD14_Mor	RP11-108M9.4
FOSL2	#######	0.536362	0.176	0.07	#######	CD14_Mor	FOSL2
DIAPH2	#######	0.644361	0.213	0.101	#######	CD14_Mor	DIAPH2
NBEAL2	#######	0.639008	0.234	0.117	#######	CD14_Mor	NBEAL2
NLRC4	#######	0.471185	0.128	0.038	#######	CD14_Mor	NLRC4
BMP2K	#######	0.413452	0.134	0.043	#######	CD14_Mor	BMP2K
CPD	#######	0.523328	0.167	0.066	#######	CD14_Mor	CPD
CTNNA1	#######	0.496726	0.236	0.12	#######	CD14_Mor	CTNNA1
TRIM25	#######	0.585862	0.171	0.07	#######	CD14_Mor	TRIM25
FGD4	#######	0.547743	0.161	0.063	#######	CD14_Mor	FGD4
JDP2	#######	0.401862	0.107	0.026	#######	CD14_Mor	JDP2
RPS111	#######	0.433518	0.76	0.75	#######	CD14_Mor	RPS11
ASRGL1	#######	0.331775	0.124	0.036	#######	CD14_Mor	ASRGL1
CAMKK2	#######	0.60528	0.194	0.089	#######	CD14_Mor	CAMKK2
ITGAX	#######	0.613727	0.248	0.132	#######	CD14_Mor	ITGAX
HEXB	#######	0.591804	0.276	0.158	#######	CD14_Mor	HEXB
NAMPT	#######	1.144327	0.24	0.13	#######	CD14_Mor	NAMPT
TBL1X	#######	0.577366	0.203	0.096	#######	CD14_Mor	TBL1X
USP15	#######	0.786003	0.45	0.355	#######	CD14_Mor	USP15
DNAJC5	#######	0.556897	0.189	0.085	#######	CD14_Mor	DNAJC5
SAT2	#######	0.65294	0.307	0.189	#######	CD14_Mor	SAT2

GPCPD1	#######	0.610883	0.261	0.145	#######	CD14_Mor	GPCPD1
TLR6	#######	0.449788	0.105	0.026	#######	CD14_Mor	TLR6
HSPA6	#######	0.53479	0.139	0.048	#######	CD14_Mor	HSPA6
SCAND1	#######	0.691341	0.47	0.377	#######	CD14_Mor	SCAND1
ZDHHC7	#######	0.507829	0.217	0.108	#######	CD14_Mor	ZDHHC7
ZFHX3	#######	0.413575	0.131	0.043	#######	CD14_Mor	ZFHX3
ZFYVE16	#######	0.521716	0.17	0.071	#######	CD14_Mor	ZFYVE16
PRKACA	#######	0.508277	0.203	0.096	#######	CD14_Mor	PRKACA
QSOX1	#######	0.537184	0.172	0.072	#######	CD14_Mor	QSOX1
SCIMP	#######	0.512907	0.263	0.144	#######	CD14_Mor	SCIMP
MAP3K1	#######	0.623595	0.29	0.175	#######	CD14_Mor	MAP3K1
TNS3	#######	0.338923	0.105	0.027	#######	CD14_Mor	TNS3
ATOX1	#######	0.640491	0.379	0.26	#######	CD14_Mor	ATOX1
STX10	#######	0.635369	0.336	0.223	#######	CD14_Mor	STX10
MIS18BP1	#######	0.647017	0.351	0.233	#######	CD14_Mor	MIS18BP1
KIAA0319L	#######	0.570268	0.178	0.078	#######	CD14_Mor	KIAA0319L
COMT	#######	0.528202	0.365	0.249	#######	CD14_Mor	COMT
ITPRIPL2	#######	0.358727	0.104	0.027	#######	CD14_Mor	ITPRIPL2
OSBPL11	#######	0.430289	0.132	0.045	#######	CD14_Mor	OSBPL11
SPECC1	#######	0.472201	0.173	0.075	#######	CD14_Mor	SPECC1
PLEKHO2	#######	0.476474	0.151	0.059	#######	CD14_Mor	PLEKHO2
MAP3K11	#######	0.606774	0.233	0.125	#######	CD14_Mor	MAP3K11
EMR2	#######	0.518685	0.217	0.108	#######	CD14_Mor	EMR2
SORT1	#######	0.387467	0.166	0.07	#######	CD14_Mor	SORT1
MAPK14	#######	0.629398	0.24	0.13	#######	CD14_Mor	MAPK14
LILRA1	#######	0.515576	0.22	0.111	#######	CD14_Mor	LILRA1
TNFRSF1A	#######	0.651881	0.351	0.237	#######	CD14_Mor	TNFRSF1A
HOOK3	#######	0.565801	0.305	0.191	#######	CD14_Mor	HOOK3
RILPL2	#######	0.537692	0.348	0.232	#######	CD14_Mor	RILPL2
LYN	#######	0.33112	0.486	0.322	#######	CD14_Mor	LYN
MTMR3	#######	0.517716	0.166	0.071	#######	CD14_Mor	MTMR3
RPL23	#######	0.502457	0.674	0.655	#######	CD14_Mor	RPL23
MSRB2	#######	0.576435	0.202	0.101	#######	CD14_Mor	MSRB2
ZFAND5	#######	0.558567	0.361	0.241	#######	CD14_Mor	ZFAND5
BCL6	#######	0.564968	0.13	0.047	#######	CD14_Mor	BCL6
AGFG1	#######	0.543565	0.182	0.086	#######	CD14_Mor	AGFG1
PPM1F	#######	0.51938	0.17	0.076	#######	CD14_Mor	PPM1F
DYNLT1	#######	0.668341	0.386	0.28	#######	CD14_Mor	DYNLT1
COX6B1	#######	0.414442	0.723	0.732	#######	CD14_Mor	COX6B1
ATP11A	#######	0.428178	0.133	0.049	#######	CD14_Mor	ATP11A
S100Z	#######	0.488894	0.141	0.055	#######	CD14_Mor	S100Z
CATSPER1	#######	0.317824	0.106	0.031	#######	CD14_Mor	CATSPER1
SCO2	#######	0.586925	0.183	0.088	#######	CD14_Mor	SCO2
ITGA5	#######	0.460677	0.161	0.07	#######	CD14_Mor	ITGA5
CEBPA	#######	0.441396	0.154	0.066	#######	CD14_Mor	CEBPA
FMNL1	#######	0.671017	0.437	0.338	#######	CD14_Mor	FMNL1
MICAL2	#######	0.352841	0.12	0.041	#######	CD14_Mor	MICAL2
LILRA3	#######	0.460663	0.169	0.075	#######	CD14_Mor	LILRA3
C6orf62	#######	0.532636	0.27	0.163	#######	CD14_Mor	C6orf62
GAPT1	#######	0.520984	0.2	0.101	#######	CD14_Mor	GAPT
EIF4EBP2	#######	0.539761	0.234	0.132	#######	CD14_Mor	EIF4EBP2
CASP1	#######	0.557926	0.425	0.311	#######	CD14_Mor	CASP1
KLF6	#######	0.728001	0.542	0.47	#######	CD14_Mor	KLF6
DEF8	#######	0.475124	0.151	0.064	#######	CD14_Mor	DEF8
SH3BP2	#######	0.571839	0.275	0.168	#######	CD14_Mor	SH3BP2
DOCK8	#######	0.653453	0.462	0.367	#######	CD14_Mor	DOCK8
ACSL4	#######	0.505858	0.214	0.115	#######	CD14_Mor	ACSL4
SLC6A6	#######	0.477311	0.17	0.079	#######	CD14_Mor	SLC6A6

MYO9B	#######	0.62355	0.293	0.188	#######	CD14_Mor	MYO9B
FRAT2	#######	0.561161	0.215	0.118	#######	CD14_Mor	FRAT2
SP1	#######	0.45159	0.169	0.079	#######	CD14_Mor	SP1
ENO1	#######	0.479873	0.616	0.583	#######	CD14_Mor	ENO1
ASAP1	#######	0.45739	0.178	0.086	#######	CD14_Mor	ASAP1
TESC	#######	0.625064	0.258	0.153	#######	CD14_Mor	TESC
TXNDC17	#######	0.621261	0.368	0.268	#######	CD14_Mor	TXNDC17
ARPC5	#######	0.287366	0.709	0.68	#######	CD14_Mor	ARPC5
AKAP13	#######	0.633959	0.516	0.443	#######	CD14_Mor	AKAP13
DENND5A	#######	0.421302	0.136	0.055	#######	CD14_Mor	DENND5A
SLFN12	#######	0.434329	0.127	0.048	#######	CD14_Mor	SLFN12
TBC1D9	#######	0.358125	0.106	0.035	#######	CD14_Mor	TBC1D9
CSF1R	#######	0.272871	0.263	0.145	#######	CD14_Mor	CSF1R
ATP5L	#######	0.366012	0.77	0.78	#######	CD14_Mor	ATP5L
ENY2	#######	0.641131	0.417	0.326	#######	CD14_Mor	ENY2
BLOC1S1	#######	0.617134	0.357	0.254	#######	CD14_Mor	BLOC1S1
SLC31A2	#######	0.423657	0.18	0.088	#######	CD14_Mor	SLC31A2
SLC25A24	#######	0.557456	0.206	0.111	#######	CD14_Mor	SLC25A24
FAR1	#######	0.543695	0.204	0.11	#######	CD14_Mor	FAR1
LILRB4	#######	0.36035	0.173	0.082	#######	CD14_Mor	LILRB4
NFIC	#######	0.464447	0.194	0.101	#######	CD14_Mor	NFIC
ATP6V1F	#######	0.530484	0.502	0.443	#######	CD14_Mor	ATP6V1F
SOX4	#######	0.591563	0.208	0.113	#######	CD14_Mor	SOX4
SIGLEC9	#######	0.410857	0.149	0.066	#######	CD14_Mor	SIGLEC9
SLC24A4	#######	0.379942	0.107	0.036	#######	CD14_Mor	SLC24A4
LPGAT1	#######	0.603711	0.281	0.179	#######	CD14_Mor	LPGAT1
SPG21	#######	0.614524	0.335	0.236	#######	CD14_Mor	SPG21
RNF24	#######	0.348739	0.165	0.076	#######	CD14_Mor	RNF24
RCSD11	#######	0.644807	0.586	0.546	#######	CD14_Mor	RCSD1
BNIP3L	#######	0.257481	0.401	0.289	#######	CD14_Mor	BNIP3L
KIAA0513	#######	0.338901	0.158	0.071	#######	CD14_Mor	KIAA0513
CARS2	#######	0.585938	0.219	0.125	#######	CD14_Mor	CARS2
WASF2	#######	0.509248	0.515	0.437	#######	CD14_Mor	WASF2
CD63	#######	0.423742	0.509	0.406	#######	CD14_Mor	CD63
IRF5	#######	0.457929	0.187	0.097	#######	CD14_Mor	IRF5
ARPC4	#######	0.512793	0.407	0.306	#######	CD14_Mor	ARPC4
PLD3	#######	0.475009	0.271	0.172	#######	CD14_Mor	PLD3
BCKDK	#######	0.419271	0.181	0.092	#######	CD14_Mor	BCKDK
CELF1	#######	0.606435	0.284	0.186	#######	CD14_Mor	CELF1
CCDC69	#######	0.612435	0.337	0.238	#######	CD14_Mor	CCDC69
PABPC1	#######	0.300474	0.84	0.834	#######	CD14_Mor	PABPC1
TBC1D10B	#######	0.40471	0.131	0.053	#######	CD14_Mor	TBC1D10B
AOAH	#######	0.494438	0.375	0.262	#######	CD14_Mor	AOAH
STX3	#######	0.411973	0.102	0.034	#######	CD14_Mor	STX3
ABHD2	#######	0.48784	0.202	0.109	#######	CD14_Mor	ABHD2
NDUFA1	#######	0.538366	0.651	0.64	#######	CD14_Mor	NDUFA1
APOBEC3/	#######	0.462698	0.127	0.05	#######	CD14_Mor	APOBEC3A
GNB1	#######	0.523879	0.355	0.255	#######	CD14_Mor	GNB1
LPCAT2	#######	0.490109	0.156	0.074	#######	CD14_Mor	LPCAT2
MXD1	#######	0.498728	0.147	0.066	#######	CD14_Mor	MXD1
ADAM15	#######	0.428244	0.169	0.084	#######	CD14_Mor	ADAM15
MLTK	#######	0.389205	0.127	0.053	#######	CD14_Mor	MLTK
DOCK2	#######	0.580628	0.342	0.245	#######	CD14_Mor	DOCK2
PTK2B	#######	0.603338	0.284	0.187	#######	CD14_Mor	PTK2B
PNPLA6	#######	0.517219	0.179	0.093	#######	CD14_Mor	PNPLA6
MED13L	#######	0.468646	0.19	0.102	#######	CD14_Mor	MED13L
YWHAG	#######	0.410407	0.208	0.116	#######	CD14_Mor	YWHAG
TUG1	#######	0.386824	0.134	0.057	#######	CD14_Mor	TUG1

ACOX1	#######	0.462685	0.14	0.063	#######	CD14_Mor	ACOX1
TGOLN2	#######	0.534354	0.426	0.337	#######	CD14_Mor	TGOLN2
SERPINB8	#######	0.365796	0.119	0.047	#######	CD14_Mor	SERPINB8
CD55	#######	0.654948	0.363	0.267	#######	CD14_Mor	CD55
TBC1D2	#######	0.314623	0.101	0.035	#######	CD14_Mor	TBC1D2
NUDT3	#######	0.502465	0.327	0.228	#######	CD14_Mor	NUDT3
IFNGR1	#######	0.547279	0.328	0.232	#######	CD14_Mor	IFNGR1
MAP3K2	#######	0.598916	0.263	0.17	#######	CD14_Mor	MAP3K2
STAT6	#######	0.59676	0.314	0.22	#######	CD14_Mor	STAT6
C20orf24	#######	0.569979	0.405	0.323	#######	CD14_Mor	C20orf24
YWHAB	#######	0.376901	0.752	0.746	#######	CD14_Mor	YWHAB
SLC12A9	#######	0.489881	0.166	0.085	#######	CD14_Mor	SLC12A9
PHF21A	#######	0.518451	0.188	0.102	#######	CD14_Mor	PHF21A
IFI16	#######	0.614036	0.428	0.345	#######	CD14_Mor	IFI16
CPEB4	#######	0.390924	0.138	0.061	#######	CD14_Mor	CPEB4
EIF3A	#######	0.581712	0.45	0.374	#######	CD14_Mor	EIF3A
LAMTOR2	#######	0.588563	0.37	0.279	#######	CD14_Mor	LAMTOR2
FURIN	#######	0.25292	0.111	0.041	#######	CD14_Mor	FURIN
PLCB2	#######	0.565493	0.245	0.153	#######	CD14_Mor	PLCB2
AGPAT2	#######	0.442903	0.181	0.097	#######	CD14_Mor	AGPAT2
DENND1A	#######	0.408271	0.137	0.062	#######	CD14_Mor	DENND1A
NPLOC4	#######	0.388612	0.128	0.055	#######	CD14_Mor	NPLOC4
FAM129A	#######	0.63186	0.206	0.118	#######	CD14_Mor	FAM129A
USF2	#######	0.381096	0.222	0.129	#######	CD14_Mor	USF2
LAP3	#######	0.607343	0.278	0.186	#######	CD14_Mor	LAP3
ROCK1	#######	0.598432	0.435	0.354	#######	CD14_Mor	ROCK1
LMNB1	#######	0.472797	0.151	0.073	#######	CD14_Mor	LMNB1
POLR2L	#######	0.505552	0.586	0.559	#######	CD14_Mor	POLR2L
FNDC3B	#######	0.445917	0.161	0.079	#######	CD14_Mor	FNDC3B
PTTG1IP	#######	0.322666	0.342	0.241	#######	CD14_Mor	PTTG1IP
BOD1L1	#######	0.648046	0.305	0.214	#######	CD14_Mor	BOD1L1
ARL11	#######	0.373332	0.12	0.051	#######	CD14_Mor	ARL11
MICU1	#######	0.32177	0.23	0.136	#######	CD14_Mor	MICU1
MRPL23	#######	0.61786	0.398	0.318	#######	CD14_Mor	MRPL23
JOSD2	#######	0.509431	0.23	0.14	#######	CD14_Mor	JOSD2
XRN2	#######	0.625119	0.407	0.328	#######	CD14_Mor	XRN2
CYB5R4	#######	0.526259	0.233	0.144	#######	CD14_Mor	CYB5R4
GLUL	#######	0.250306	0.341	0.235	#######	CD14_Mor	GLUL
WSB1	#######	0.617437	0.454	0.369	#######	CD14_Mor	WSB1
ATP5J2	#######	0.519918	0.602	0.583	#######	CD14_Mor	ATP5J2
PSMB3	#######	0.583895	0.503	0.454	#######	CD14_Mor	PSMB3
VMA21	#######	0.562273	0.274	0.184	#######	CD14_Mor	VMA21
MBD2	#######	0.48103	0.217	0.13	#######	CD14_Mor	MBD2
NFKBIA	#######	0.710778	0.391	0.312	#######	CD14_Mor	NFKBIA
PARP14	#######	0.556684	0.26	0.17	#######	CD14_Mor	PARP14
ARHGAP2	#######	0.49094	0.213	0.126	#######	CD14_Mor	ARHGAP27
PISD	#######	0.392596	0.133	0.06	#######	CD14_Mor	PISD
VAMP5	#######	0.65896	0.342	0.252	#######	CD14_Mor	VAMP5
TAOK1	#######	0.471858	0.21	0.125	#######	CD14_Mor	TAOK1
MYO15B	#######	0.339935	0.101	0.038	#######	CD14_Mor	MYO15B
NAGA	#######	0.393213	0.193	0.11	#######	CD14_Mor	NAGA
NIN	#######	0.559489	0.314	0.224	#######	CD14_Mor	NIN
RRP12	#######	0.394414	0.107	0.043	#######	CD14_Mor	RRP12
GGA3	#######	0.445355	0.138	0.066	#######	CD14_Mor	GGA3
JAK2	#######	0.458319	0.215	0.13	#######	CD14_Mor	JAK2
TMEM33	#######	0.457952	0.19	0.108	#######	CD14_Mor	TMEM33
TRIOBP	#######	0.360306	0.125	0.056	#######	CD14_Mor	TRIOBP
RRBP1	#######	0.516793	0.253	0.164	#######	CD14_Mor	RRBP1

SRGAP2	#######	0.304266	0.1	0.039	#######	CD14_Mor	SRGAP2
BICD2	#######	0.384018	0.212	0.127	#######	CD14_Mor	BICD2
TNFSF13	#######	0.362833	0.163	0.086	#######	CD14_Mor	TNFSF13
WAS	#######	0.560461	0.46	0.39	#######	CD14_Mor	WAS
ATG3	#######	0.524377	0.378	0.288	#######	CD14_Mor	ATG3
IMPDH1	#######	0.487049	0.251	0.164	#######	CD14_Mor	IMPDH1
ARHGAP24	#######	0.306001	0.1	0.038	#######	CD14_Mor	ARHGAP24
IRAK1	#######	0.329176	0.119	0.053	#######	CD14_Mor	IRAK1
TMEM127	#######	0.421733	0.179	0.1	#######	CD14_Mor	TMEM127
PID1	#######	0.262327	0.104	0.042	#######	CD14_Mor	PID1
SLFN11	#######	0.300613	0.107	0.044	#######	CD14_Mor	SLFN11
DOCK5	#######	0.433321	0.161	0.085	#######	CD14_Mor	DOCK5
MAP4K4	#######	0.494598	0.198	0.116	#######	CD14_Mor	MAP4K4
OAZ2	#######	0.478945	0.191	0.112	#######	CD14_Mor	OAZ2
DENND4B	#######	0.435108	0.163	0.087	#######	CD14_Mor	DENND4B
PIK3AP1	#######	0.485242	0.259	0.167	#######	CD14_Mor	PIK3AP1
TRIP12	#######	0.488524	0.263	0.176	#######	CD14_Mor	TRIP12
PRR13	#######	0.577977	0.49	0.431	#######	CD14_Mor	PRR13
RNF144B	#######	0.446142	0.16	0.085	#######	CD14_Mor	RNF144B
MTF1	#######	0.456315	0.149	0.077	#######	CD14_Mor	MTF1
TOM1	#######	0.457555	0.173	0.096	#######	CD14_Mor	TOM1
SIGLEC7	#######	0.355156	0.147	0.074	#######	CD14_Mor	SIGLEC7
AZI2	#######	0.468444	0.2	0.119	#######	CD14_Mor	AZI2
SHOC2	#######	0.559706	0.348	0.266	#######	CD14_Mor	SHOC2
TBC1D2B	#######	0.346284	0.101	0.041	#######	CD14_Mor	TBC1D2B
RPS6KA4	#######	0.385081	0.18	0.103	#######	CD14_Mor	RPS6KA4
MNT	#######	0.344864	0.107	0.045	#######	CD14_Mor	MNT
GK	#######	0.282918	0.118	0.053	#######	CD14_Mor	GK
TLE3	#######	0.446833	0.137	0.068	#######	CD14_Mor	TLE3
MSL3	#######	0.623282	0.267	0.186	#######	CD14_Mor	MSL3
SPOPL	#######	0.378852	0.124	0.058	#######	CD14_Mor	SPOPL
CARD9	#######	0.32567	0.122	0.057	#######	CD14_Mor	CARD9
TFEB	#######	0.352742	0.132	0.065	#######	CD14_Mor	TFEB
FAM214B	#######	0.394067	0.133	0.065	#######	CD14_Mor	FAM214B
BCL3	#######	0.441702	0.152	0.081	#######	CD14_Mor	BCL3
RAB34	#######	0.283177	0.12	0.056	#######	CD14_Mor	RAB34
KIAA2013	#######	0.335301	0.119	0.055	#######	CD14_Mor	KIAA2013
VPS13C	#######	0.55881	0.363	0.285	#######	CD14_Mor	VPS13C
HNRNPUL	#######	0.330093	0.126	0.06	#######	CD14_Mor	HNRNPUL2
WARS	#######	0.395051	0.288	0.194	#######	CD14_Mor	WARS
COL4A3BP	#######	0.526326	0.244	0.163	#######	CD14_Mor	COL4A3BP
KLF10	#######	0.423998	0.169	0.096	#######	CD14_Mor	KLF10
MANBA	#######	0.473958	0.223	0.145	#######	CD14_Mor	MANBA
AP1B1	#######	0.432934	0.207	0.129	#######	CD14_Mor	AP1B1
ATG7	#######	0.382529	0.14	0.072	#######	CD14_Mor	ATG7
TMBIM4.1	#######	0.552066	0.406	0.334	#######	CD14_Mor	TMBIM4.1
ANKRD13D	#######	0.539545	0.271	0.191	#######	CD14_Mor	ANKRD13D
PLEKHB2	#######	0.465846	0.267	0.185	#######	CD14_Mor	PLEKHB2
NINJ1	#######	0.41309	0.273	0.185	#######	CD14_Mor	NINJ1
TUBA1A	#######	0.648831	0.437	0.362	#######	CD14_Mor	TUBA1A
DDX60L	#######	0.473848	0.181	0.107	#######	CD14_Mor	DDX60L
REL1	#######	0.568518	0.375	0.294	#######	CD14_Mor	REL
SCARB2	#######	0.384065	0.164	0.092	#######	CD14_Mor	SCARB2
SCAF11	#######	0.592918	0.471	0.415	#######	CD14_Mor	SCAF11
RCBTB2	#######	0.420768	0.183	0.108	#######	CD14_Mor	RCBTB2
PSMA7	#######	0.470535	0.616	0.609	#######	CD14_Mor	PSMA7
NLRP1	#######	0.484538	0.242	0.162	#######	CD14_Mor	NLRP1
STAC3	#######	0.27892	0.108	0.049	#######	CD14_Mor	STAC3

CHD1	#######	0.438266	0.181	0.108	#######	CD14_Mor	CHD1
PAFAH1B2	#######	0.446129	0.171	0.1	#######	CD14_Mor	PAFAH1B2
HHEX1	#######	0.394316	0.182	0.109	#######	CD14_Mor	HHEX
RBM5	#######	0.562204	0.261	0.184	#######	CD14_Mor	RBM5
CSTB	#######	0.416711	0.567	0.511	#######	CD14_Mor	CSTB
RNF135	#######	0.397501	0.151	0.083	#######	CD14_Mor	RNF135
FAM49A	#######	0.433439	0.206	0.13	#######	CD14_Mor	FAM49A
SHKBP1	#######	0.531308	0.345	0.27	#######	CD14_Mor	SHKBP1
DDX17	#######	0.544048	0.536	0.497	#######	CD14_Mor	DDX17
CREBBP	#######	0.419135	0.193	0.119	#######	CD14_Mor	CREBBP
MTHFR	#######	0.349884	0.137	0.072	#######	CD14_Mor	MTHFR
AGPAT9	#######	0.453187	0.123	0.062	#######	CD14_Mor	AGPAT9
SBNO2	#######	0.342319	0.107	0.049	#######	CD14_Mor	SBNO2
RNPEP	#######	0.434172	0.237	0.161	#######	CD14_Mor	RNPEP
KLF4	#######	0.378838	0.159	0.091	#######	CD14_Mor	KLF4
AGPAT3	#######	0.352027	0.152	0.085	#######	CD14_Mor	AGPAT3
CDV3	#######	0.460387	0.27	0.194	#######	CD14_Mor	CDV3
NABP1	#######	0.441509	0.135	0.071	#######	CD14_Mor	NABP1
NISCH	#######	0.311869	0.116	0.056	3.11E-99	CD14_Mor	NISCH
CCDC159	#######	0.309646	0.13	0.067	3.48E-99	CD14_Mor	CCDC159
KMT2C	#######	0.495282	0.314	0.237	3.93E-99	CD14_Mor	KMT2C
SOS2	#######	0.414325	0.147	0.081	3.93E-99	CD14_Mor	SOS2
CYSTM1	#######	0.648642	0.13	0.069	4.46E-99	CD14_Mor	CYSTM1
CLCN7	#######	0.318584	0.112	0.054	5.08E-99	CD14_Mor	CLCN7
KLHL8	#######	0.348263	0.117	0.058	4.24E-98	CD14_Mor	KLHL8
PRKAR1A	#######	0.447873	0.394	0.321	9.17E-98	CD14_Mor	PRKAR1A
HIGD2A	#######	0.427149	0.615	0.59	2.68E-96	CD14_Mor	HIGD2A
ZFP36L11	#######	0.671481	0.524	0.494	3.79E-96	CD14_Mor	ZFP36L1
MSL1	#######	0.41822	0.149	0.084	2.71E-95	CD14_Mor	MSL1
OSBPL8	#######	0.536774	0.423	0.364	3.14E-95	CD14_Mor	OSBPL8
MGAT1	1.42E-99	0.499117	0.34	0.269	4.64E-95	CD14_Mor	MGAT1
FBP1	3.73E-99	0.305832	0.167	0.099	1.22E-94	CD14_Mor	FBP1
CEP170	4.83E-99	0.384357	0.154	0.089	1.58E-94	CD14_Mor	CEP170
HIPK1	9.53E-99	0.319042	0.144	0.079	3.12E-94	CD14_Mor	HIPK1
WDR26	1.80E-98	0.317099	0.151	0.085	5.88E-94	CD14_Mor	WDR26
SLC36A4	3.79E-98	0.361917	0.123	0.064	1.24E-93	CD14_Mor	SLC36A4
UQCRC1	5.75E-98	0.495944	0.372	0.305	1.88E-93	CD14_Mor	UQCRC1
EP300	5.84E-98	0.468445	0.223	0.151	1.91E-93	CD14_Mor	EP300
ZCCHC6	1.56E-97	0.499085	0.234	0.161	5.11E-93	CD14_Mor	ZCCHC6
NARS	2.70E-96	0.458592	0.241	0.169	8.85E-92	CD14_Mor	NARS
SERPINB6	5.14E-96	0.379781	0.277	0.199	1.68E-91	CD14_Mor	SERPINB6
DENND3	5.46E-96	0.402147	0.13	0.071	1.79E-91	CD14_Mor	DENND3
RP2	1.14E-95	0.356817	0.126	0.068	3.72E-91	CD14_Mor	RP2
RCOR1	1.19E-95	0.403826	0.171	0.105	3.90E-91	CD14_Mor	RCOR1
GALNT1	1.43E-95	0.36375	0.133	0.073	4.68E-91	CD14_Mor	GALNT1
LSP1	3.01E-95	0.423732	0.68	0.661	9.87E-91	CD14_Mor	LSP1
ATXN1	6.07E-95	0.347788	0.121	0.062	1.99E-90	CD14_Mor	ATXN1
LENG8	1.02E-94	0.507325	0.243	0.172	3.35E-90	CD14_Mor	LENG8
WASH4P	1.51E-94	0.316175	0.135	0.073	4.93E-90	CD14_Mor	WASH4P
SRRM2	1.61E-94	0.509838	0.549	0.521	5.27E-90	CD14_Mor	SRRM2
FAM91A1	1.68E-94	0.367003	0.136	0.076	5.51E-90	CD14_Mor	FAM91A1
DHRS4L2	2.96E-94	0.303034	0.106	0.052	9.67E-90	CD14_Mor	DHRS4L2
USP7	1.88E-93	0.37502	0.154	0.09	6.14E-89	CD14_Mor	USP7
TPD52L2	3.58E-93	0.382011	0.176	0.109	1.17E-88	CD14_Mor	TPD52L2
CPNE8	4.12E-93	0.258274	0.101	0.048	1.35E-88	CD14_Mor	CPNE8
XAF1	6.46E-93	0.561953	0.246	0.175	2.11E-88	CD14_Mor	XAF1
TFE3	3.76E-92	0.288615	0.113	0.057	1.23E-87	CD14_Mor	TFE3
FAM26F1	5.44E-92	0.327346	0.216	0.139	1.78E-87	CD14_Mor	FAM26F

RNPEPL1	2.01E-91	0.401392	0.239	0.166	6.59E-87	CD14_Mor	RNPEPL1
TMEM219	2.02E-91	0.255229	0.475	0.413	6.61E-87	CD14_Mor	TMEM219
TIMM8B	1.66E-90	0.53087	0.315	0.248	5.45E-86	CD14_Mor	TIMM8B
ADAM17	1.99E-90	0.36622	0.137	0.078	6.51E-86	CD14_Mor	ADAM17
NCOA1	2.32E-90	0.362526	0.169	0.104	7.58E-86	CD14_Mor	NCOA1
RENBP	5.35E-90	0.264995	0.146	0.084	1.75E-85	CD14_Mor	RENBP
PTEN	1.02E-89	0.520049	0.311	0.243	3.34E-85	CD14_Mor	PTEN
SLC31A1	1.05E-89	0.290775	0.105	0.052	3.43E-85	CD14_Mor	SLC31A1
REEP4	4.50E-89	0.28591	0.128	0.07	1.47E-84	CD14_Mor	REEP4
GPBAR1	4.79E-89	0.292699	0.176	0.107	1.57E-84	CD14_Mor	GPBAR1
EIF4E3	6.28E-89	0.425268	0.197	0.128	2.05E-84	CD14_Mor	EIF4E3
ZBTB7B	7.70E-89	0.2898	0.128	0.07	2.52E-84	CD14_Mor	ZBTB7B
CFLAR	4.42E-88	0.489426	0.422	0.36	1.45E-83	CD14_Mor	CFLAR
HDLBP	6.39E-88	0.393264	0.259	0.189	2.09E-83	CD14_Mor	HDLBP
KIAA10331	1.36E-87	0.484611	0.257	0.189	4.44E-83	CD14_Mor	KIAA1033
NOP10	1.95E-87	0.553319	0.487	0.46	6.37E-83	CD14_Mor	NOP10
UBA1	8.76E-87	0.431977	0.248	0.179	2.87E-82	CD14_Mor	UBA1
RNF181	2.58E-86	0.481148	0.384	0.328	8.44E-82	CD14_Mor	RNF181
FLII	4.52E-86	0.38854	0.265	0.195	1.48E-81	CD14_Mor	FLII
KLF13	8.53E-86	0.450718	0.262	0.191	2.79E-81	CD14_Mor	KLF13
MOSPD2	1.54E-85	0.375379	0.124	0.069	5.03E-81	CD14_Mor	MOSPD2
IL10RB	2.95E-85	0.470111	0.243	0.175	9.65E-81	CD14_Mor	IL10RB
JARID2	4.05E-85	0.320114	0.163	0.1	1.33E-80	CD14_Mor	JARID2
MGST2	1.24E-84	0.287343	0.142	0.083	4.05E-80	CD14_Mor	MGST2
ST8SIA4	1.28E-84	0.40718	0.162	0.101	4.18E-80	CD14_Mor	ST8SIA4
ARHGEF2	1.46E-84	0.474681	0.229	0.162	4.77E-80	CD14_Mor	ARHGEF2
C3AR1	3.13E-84	0.384662	0.145	0.086	1.02E-79	CD14_Mor	C3AR1
MBD6	4.65E-84	0.282206	0.105	0.054	1.52E-79	CD14_Mor	MBD6
BRD4	5.20E-84	0.453648	0.27	0.203	1.70E-79	CD14_Mor	BRD4
BRI3BP	2.23E-83	0.359939	0.145	0.087	7.31E-79	CD14_Mor	BRI3BP
HEBP1	3.75E-83	0.330875	0.206	0.141	1.23E-78	CD14_Mor	HEBP1
NDUFA3	9.94E-83	0.511068	0.602	0.6	3.25E-78	CD14_Mor	NDUFA3
CBWD1	1.14E-82	0.323377	0.135	0.079	3.72E-78	CD14_Mor	CBWD1
GNB4	1.24E-82	0.327747	0.129	0.074	4.05E-78	CD14_Mor	GNB4
SBF2	3.49E-82	0.374595	0.141	0.084	1.14E-77	CD14_Mor	SBF2
NCOR2	8.07E-82	0.360113	0.167	0.107	2.64E-77	CD14_Mor	NCOR2
STX71	9.27E-82	0.351562	0.28	0.21	3.03E-77	CD14_Mor	STX7
PPP1CB	1.08E-81	0.472562	0.27	0.205	3.54E-77	CD14_Mor	PPP1CB
SETX	1.71E-81	0.483289	0.307	0.242	5.60E-77	CD14_Mor	SETX
MAN2C1	3.79E-81	0.325565	0.132	0.076	1.24E-76	CD14_Mor	MAN2C1
TMEM30A	4.26E-81	0.409465	0.202	0.138	1.39E-76	CD14_Mor	TMEM30A
GAK	6.76E-81	0.40705	0.169	0.109	2.21E-76	CD14_Mor	GAK
CSGALNA(	6.14E-80	0.368118	0.157	0.098	2.01E-75	CD14_Mor	CSGALNACT2
NXF1	7.44E-80	0.331457	0.132	0.077	2.44E-75	CD14_Mor	NXF1
FGD21	8.75E-80	0.372802	0.203	0.138	2.87E-75	CD14_Mor	FGD2
RREB1	1.93E-79	0.288002	0.105	0.055	6.32E-75	CD14_Mor	RREB1
COPA	2.73E-79	0.413978	0.255	0.19	8.95E-75	CD14_Mor	COPA
NFE2L2	3.35E-79	0.47677	0.344	0.282	1.10E-74	CD14_Mor	NFE2L2
UNC93B1	9.06E-79	0.388823	0.208	0.145	2.97E-74	CD14_Mor	UNC93B1
HNRNPH1	1.09E-78	0.467972	0.305	0.241	3.56E-74	CD14_Mor	HNRNPH1
TM9SF2	2.80E-78	0.4587	0.329	0.264	9.16E-74	CD14_Mor	TM9SF2
IQSEC1	5.98E-78	0.398443	0.171	0.111	1.96E-73	CD14_Mor	IQSEC1
CDK19	2.00E-77	0.304372	0.115	0.064	6.54E-73	CD14_Mor	CDK19
MOB3A	3.15E-77	0.44624	0.251	0.186	1.03E-72	CD14_Mor	MOB3A
NUFIP2	1.51E-76	0.4096	0.186	0.126	4.96E-72	CD14_Mor	NUFIP2
LARP1	1.59E-76	0.324109	0.164	0.106	5.22E-72	CD14_Mor	LARP1
MRPL33	1.61E-76	0.498248	0.35	0.295	5.27E-72	CD14_Mor	MRPL33
FRAT1	3.48E-76	0.329811	0.119	0.067	1.14E-71	CD14_Mor	FRAT1

C15orf39	5.62E-76	0.37955	0.168	0.11	1.84E-71	CD14_Mor	C15orf39
SEC61B	6.15E-76	0.41966	0.551	0.531	2.01E-71	CD14_Mor	SEC61B
RB1CC1	1.03E-75	0.436103	0.251	0.187	3.36E-71	CD14_Mor	RB1CC1
MED13	4.24E-75	0.31546	0.133	0.079	1.39E-70	CD14_Mor	MED13
DAPK1	6.91E-75	0.31125	0.136	0.082	2.26E-70	CD14_Mor	DAPK1
HSPA1A	6.99E-75	0.456968	0.278	0.211	2.29E-70	CD14_Mor	HSPA1A
TCF25	1.02E-74	0.468769	0.528	0.499	3.33E-70	CD14_Mor	TCF25
NAAA	2.21E-74	0.366947	0.346	0.274	7.25E-70	CD14_Mor	NAAA
FAM111A	2.96E-74	0.404297	0.186	0.126	9.68E-70	CD14_Mor	FAM111A
FKBP5	4.02E-74	0.462685	0.256	0.193	1.32E-69	CD14_Mor	FKBP5
EIF4E2	9.41E-74	0.467453	0.305	0.244	3.08E-69	CD14_Mor	EIF4E2
CXorf21	1.31E-73	0.307874	0.116	0.067	4.30E-69	CD14_Mor	CXorf21
FUOM	1.60E-73	0.375864	0.155	0.099	5.24E-69	CD14_Mor	FUOM
CDC42	3.02E-73	0.298065	0.624	0.629	9.89E-69	CD14_Mor	CDC42
MT2A	4.20E-73	0.719519	0.469	0.424	1.37E-68	CD14_Mor	MT2A
TRIM381	7.47E-73	0.495541	0.27	0.209	2.44E-68	CD14_Mor	TRIM38
TPI1	1.14E-72	0.319798	0.629	0.62	3.72E-68	CD14_Mor	TPI1
VASP	1.24E-72	0.34812	0.449	0.38	4.05E-68	CD14_Mor	VASP
ADPGK	2.48E-72	0.409608	0.21	0.15	8.11E-68	CD14_Mor	ADPGK
LRCH4	2.65E-72	0.410521	0.178	0.121	8.68E-68	CD14_Mor	LRCH4
LINC0093(	5.28E-72	0.432408	0.13	0.079	1.73E-67	CD14_Mor	LINC00936
PXN	1.01E-71	0.392517	0.246	0.177	3.29E-67	CD14_Mor	PXN
DDX3X	2.04E-71	0.454089	0.301	0.242	6.67E-67	CD14_Mor	DDX3X
SH3KBP1	6.50E-71	0.508432	0.418	0.371	2.13E-66	CD14_Mor	SH3KBP1
CSNK2B	8.59E-71	0.530767	0.454	0.422	2.81E-66	CD14_Mor	CSNK2B
CBL	8.68E-71	0.372228	0.207	0.147	2.84E-66	CD14_Mor	CBL
DNAJC13	9.11E-71	0.338304	0.138	0.085	2.98E-66	CD14_Mor	DNAJC13
UBE2E21	2.22E-70	0.389645	0.167	0.112	7.27E-66	CD14_Mor	UBE2E2
FUCA2	5.82E-70	0.283008	0.154	0.099	1.91E-65	CD14_Mor	FUCA2
AIM1	2.21E-69	0.418353	0.204	0.146	7.24E-65	CD14_Mor	AIM1
FAM105A	2.56E-69	0.369114	0.197	0.138	8.37E-65	CD14_Mor	FAM105A
IER2	3.54E-69	0.787576	0.443	0.42	1.16E-64	CD14_Mor	IER2
MAST3	5.39E-69	0.369507	0.122	0.072	1.76E-64	CD14_Mor	MAST3
MYOF	1.89E-68	0.271512	0.125	0.075	6.18E-64	CD14_Mor	MYOF
C20orf27	5.27E-68	0.38815	0.266	0.204	1.73E-63	CD14_Mor	C20orf27
SPAG9	5.16E-67	0.390958	0.173	0.118	1.69E-62	CD14_Mor	SPAG9
CD300C	5.27E-67	0.25077	0.135	0.084	1.72E-62	CD14_Mor	CD300C
KLHL18	1.01E-66	0.358709	0.129	0.08	3.30E-62	CD14_Mor	KLHL18
SIK3	1.08E-66	0.33303	0.135	0.084	3.55E-62	CD14_Mor	SIK3
MRPL52	1.16E-66	0.488554	0.374	0.329	3.79E-62	CD14_Mor	MRPL52
DDX21	1.79E-66	0.523704	0.399	0.353	5.87E-62	CD14_Mor	DDX21
MCMBP	3.33E-66	0.403739	0.166	0.113	1.09E-61	CD14_Mor	MCMBP
GALK1	6.31E-66	0.304544	0.15	0.097	2.07E-61	CD14_Mor	GALK1
SEC11A	6.36E-66	0.404105	0.456	0.421	2.08E-61	CD14_Mor	SEC11A
JMJD1C	1.09E-65	0.455091	0.273	0.215	3.56E-61	CD14_Mor	JMJD1C
IDH1	2.22E-65	0.26574	0.123	0.074	7.27E-61	CD14_Mor	IDH1
RELB	4.08E-65	0.350736	0.146	0.095	1.33E-60	CD14_Mor	RELB
RHOT1	1.33E-64	0.361995	0.15	0.098	4.36E-60	CD14_Mor	RHOT1
MYO5A	1.36E-64	0.332021	0.134	0.084	4.46E-60	CD14_Mor	MYO5A
MX2	1.52E-64	0.518113	0.219	0.164	4.97E-60	CD14_Mor	MX2
OS9	3.45E-64	0.412176	0.311	0.255	1.13E-59	CD14_Mor	OS9
PQLC1	4.01E-64	0.311704	0.122	0.075	1.31E-59	CD14_Mor	PQLC1
PET100	1.82E-63	0.429403	0.499	0.479	5.96E-59	CD14_Mor	PET100
ERICH1	1.83E-63	0.419604	0.317	0.259	5.99E-59	CD14_Mor	ERICH1
RNF149	2.00E-63	0.604192	0.417	0.371	6.54E-59	CD14_Mor	RNF149
CHN2	2.25E-63	0.330086	0.129	0.081	7.37E-59	CD14_Mor	CHN2
KYNU	3.92E-63	0.306279	0.127	0.08	1.28E-58	CD14_Mor	KYNU
REEP5	2.95E-62	0.440169	0.411	0.367	9.66E-58	CD14_Mor	REEP5

XIST	5.08E-62	0.384336	0.143	0.093	1.66E-57	CD14_Mor	XIST
KDM7A	7.81E-62	0.272425	0.134	0.084	2.56E-57	CD14_Mor	KDM7A
KIAA0232	2.37E-61	0.341922	0.143	0.093	7.75E-57	CD14_Mor	KIAA0232
HOTAIRM1	3.58E-61	0.355784	0.184	0.13	1.17E-56	CD14_Mor	HOTAIRM1
SH3GLB1	3.83E-61	0.471825	0.336	0.285	1.25E-56	CD14_Mor	SH3GLB1
LSMD1	6.05E-61	0.330623	0.451	0.413	1.98E-56	CD14_Mor	LSMD1
DHRS4	2.26E-60	0.354452	0.232	0.177	7.41E-56	CD14_Mor	DHRS4
OGFR	2.35E-60	0.46282	0.314	0.261	7.70E-56	CD14_Mor	OGFR
ATF5	2.55E-60	0.260035	0.101	0.058	8.36E-56	CD14_Mor	ATF5
AURKAIP1	1.07E-59	0.49569	0.405	0.369	3.50E-55	CD14_Mor	AURKAIP1
CCPG1	1.42E-59	0.351186	0.274	0.214	4.65E-55	CD14_Mor	CCPG1
PJA2	1.94E-59	0.40463	0.261	0.206	6.37E-55	CD14_Mor	PJA2
CMTM7	2.63E-59	0.423996	0.266	0.212	8.61E-55	CD14_Mor	CMTM7
SOAT1	6.19E-59	0.31319	0.153	0.103	2.03E-54	CD14_Mor	SOAT1
ARHGAP3(	1.54E-58	0.507257	0.422	0.386	5.05E-54	CD14_Mor	ARHGAP30
A1BG	1.75E-58	0.346465	0.182	0.128	5.73E-54	CD14_Mor	A1BG
RAB21	2.60E-58	0.275862	0.112	0.068	8.50E-54	CD14_Mor	RAB21
TRIM8	1.01E-57	0.272428	0.134	0.087	3.29E-53	CD14_Mor	TRIM8
CREBRF	1.17E-57	0.425895	0.201	0.149	3.83E-53	CD14_Mor	CREBRF
RNF141	1.30E-57	0.365398	0.163	0.113	4.26E-53	CD14_Mor	RNF141
CHCHD5	1.31E-57	0.469422	0.258	0.206	4.30E-53	CD14_Mor	CHCHD5
RIN3	1.33E-57	0.426038	0.283	0.227	4.36E-53	CD14_Mor	RIN3
ITGAE	1.49E-57	0.379953	0.194	0.14	4.87E-53	CD14_Mor	ITGAE
ABR	2.04E-57	0.298923	0.126	0.08	6.67E-53	CD14_Mor	ABR
VAMP3	2.37E-57	0.345733	0.194	0.141	7.74E-53	CD14_Mor	VAMP3
PHF20L1	2.38E-57	0.342653	0.267	0.211	7.79E-53	CD14_Mor	PHF20L1
PLCB1	6.88E-57	0.256641	0.107	0.063	2.25E-52	CD14_Mor	PLCB1
KIAA0100	2.13E-56	0.334014	0.17	0.12	6.97E-52	CD14_Mor	KIAA0100
RC3H1	4.29E-56	0.476758	0.269	0.218	1.40E-51	CD14_Mor	RC3H1
KDM2A	4.77E-56	0.362726	0.177	0.126	1.56E-51	CD14_Mor	KDM2A
FBXO9	6.47E-56	0.419142	0.231	0.179	2.12E-51	CD14_Mor	FBXO9
EMB	1.97E-55	0.540071	0.297	0.246	6.46E-51	CD14_Mor	EMB
CLIP1	4.48E-55	0.306739	0.208	0.154	1.47E-50	CD14_Mor	CLIP1
PGAM1	4.57E-55	0.438448	0.477	0.462	1.50E-50	CD14_Mor	PGAM1
MYD88	6.32E-55	0.3903	0.262	0.208	2.07E-50	CD14_Mor	MYD88
SLC27A3	7.11E-55	0.302689	0.142	0.095	2.33E-50	CD14_Mor	SLC27A3
EDEM3	9.19E-55	0.410742	0.194	0.144	3.01E-50	CD14_Mor	EDEM3
EXOC3	9.95E-55	0.292432	0.13	0.085	3.26E-50	CD14_Mor	EXOC3
PPFIA1	1.41E-54	0.353586	0.172	0.122	4.63E-50	CD14_Mor	PPFIA1
TBC1D14	2.93E-54	0.295863	0.121	0.077	9.59E-50	CD14_Mor	TBC1D14
IFNAR1	7.03E-54	0.464447	0.257	0.208	2.30E-49	CD14_Mor	IFNAR1
PSEN1	7.84E-54	0.341044	0.148	0.102	2.57E-49	CD14_Mor	PSEN1
SGK3	8.20E-54	0.275301	0.114	0.071	2.68E-49	CD14_Mor	SGK3
ATP5D	1.01E-53	0.385637	0.591	0.604	3.31E-49	CD14_Mor	ATP5D
KDM4B	1.95E-53	0.298222	0.113	0.071	6.38E-49	CD14_Mor	KDM4B
MTX1	1.97E-53	0.350671	0.159	0.112	6.46E-49	CD14_Mor	MTX1
FAM46A	3.05E-53	0.316362	0.151	0.105	9.97E-49	CD14_Mor	FAM46A
HSD17B11	4.26E-53	0.469979	0.334	0.291	1.40E-48	CD14_Mor	HSD17B11
CHMP4B	1.67E-52	0.405023	0.394	0.356	5.47E-48	CD14_Mor	CHMP4B
SEC16A	1.75E-52	0.263751	0.119	0.076	5.74E-48	CD14_Mor	SEC16A
ACTN4	1.99E-52	0.403079	0.318	0.266	6.53E-48	CD14_Mor	ACTN4
RAB24	2.08E-52	0.354891	0.265	0.21	6.80E-48	CD14_Mor	RAB24
SH3TC1	2.26E-52	0.299348	0.117	0.075	7.41E-48	CD14_Mor	SH3TC1
RP11-349/	3.50E-52	0.363887	0.19	0.14	1.14E-47	CD14_Mor	RP11-349A22.5
DUSP23	3.59E-52	0.409242	0.216	0.165	1.18E-47	CD14_Mor	DUSP23
IL4R1	4.54E-52	0.360854	0.144	0.097	1.49E-47	CD14_Mor	IL4R
ATF6	1.43E-51	0.306187	0.157	0.11	4.68E-47	CD14_Mor	ATF6
LRRC58	3.03E-51	0.281381	0.134	0.089	9.91E-47	CD14_Mor	LRRC58

NDUFB11	4.25E-51	0.407425	0.559	0.564	1.39E-46	CD14_Mor	NDUFB11
UBL5	6.17E-51	0.31378	0.665	0.703	2.02E-46	CD14_Mor	UBL5
ORAI3	1.84E-50	0.282562	0.163	0.116	6.02E-46	CD14_Mor	ORAI3
WAC	3.76E-50	0.424022	0.28	0.233	1.23E-45	CD14_Mor	WAC
PREX1	5.14E-50	0.419629	0.265	0.216	1.68E-45	CD14_Mor	PREX1
SELL1	5.25E-50	0.295168	0.493	0.427	1.72E-45	CD14_Mor	SELL
STX16	1.43E-49	0.389014	0.187	0.139	4.70E-45	CD14_Mor	STX16
CHMP2A	2.11E-49	0.40433	0.341	0.3	6.90E-45	CD14_Mor	CHMP2A
ROGDI	2.62E-49	0.284151	0.13	0.088	8.59E-45	CD14_Mor	ROGDI
SRA1	6.13E-49	0.425259	0.238	0.19	2.01E-44	CD14_Mor	SRA1
PSMB4	7.99E-49	0.407189	0.302	0.257	2.62E-44	CD14_Mor	PSMB4
USP32	1.16E-48	0.294618	0.113	0.073	3.79E-44	CD14_Mor	USP32
NPEPPS	2.30E-48	0.327668	0.203	0.154	7.53E-44	CD14_Mor	NPEPPS
KHSRP	2.34E-48	0.267735	0.114	0.074	7.65E-44	CD14_Mor	KHSRP
RNASET2	9.36E-48	0.278008	0.488	0.432	3.06E-43	CD14_Mor	RNASET2
FBXO11	1.00E-47	0.26487	0.102	0.064	3.28E-43	CD14_Mor	FBXO11
RTN4	1.34E-47	0.276735	0.452	0.416	4.38E-43	CD14_Mor	RTN4
RP11-139I	1.89E-47	0.376669	0.177	0.131	6.18E-43	CD14_Mor	RP11-139H15.1
CLTA	2.64E-47	0.32419	0.45	0.43	8.66E-43	CD14_Mor	CLTA
FOXO3	2.83E-47	0.257691	0.155	0.108	9.25E-43	CD14_Mor	FOXO3
PELI1	3.50E-47	0.284227	0.112	0.073	1.15E-42	CD14_Mor	PELI1
SH2B3	4.33E-47	0.285377	0.188	0.141	1.42E-42	CD14_Mor	SH2B3
NUP62	5.22E-47	0.289928	0.141	0.098	1.71E-42	CD14_Mor	NUP62
PTPN61	7.16E-47	0.298865	0.534	0.493	2.34E-42	CD14_Mor	PTPN6
RELT	1.59E-46	0.282019	0.121	0.081	5.20E-42	CD14_Mor	RELT
MEF2A	2.32E-46	0.333851	0.206	0.16	7.58E-42	CD14_Mor	MEF2A
OAS1	2.32E-46	0.270931	0.207	0.157	7.59E-42	CD14_Mor	OAS1
ANAPC11	3.01E-46	0.37467	0.425	0.396	9.85E-42	CD14_Mor	ANAPC11
PRR14L	3.62E-46	0.30914	0.145	0.102	1.18E-41	CD14_Mor	PRR14L
AKR1A1	3.73E-46	0.392103	0.29	0.247	1.22E-41	CD14_Mor	AKR1A1
SMARCD2	1.60E-45	0.300505	0.14	0.098	5.24E-41	CD14_Mor	SMARCD2
PPP3CA	1.76E-45	0.362562	0.184	0.138	5.76E-41	CD14_Mor	PPP3CA
ARF5	3.06E-45	0.323773	0.51	0.498	1.00E-40	CD14_Mor	ARF5
GDE1	4.77E-45	0.252639	0.141	0.098	1.56E-40	CD14_Mor	GDE1
PRKAG2	2.33E-44	0.296419	0.143	0.101	7.62E-40	CD14_Mor	PRKAG2
HCFC1R1	6.77E-44	0.310469	0.197	0.151	2.22E-39	CD14_Mor	HCFC1R1
SMCHD1	1.01E-43	0.56145	0.403	0.378	3.31E-39	CD14_Mor	SMCHD1
KDM1B	1.48E-43	0.286858	0.138	0.097	4.85E-39	CD14_Mor	KDM1B
NDUFA2	4.08E-43	0.442167	0.399	0.372	1.34E-38	CD14_Mor	NDUFA2
DYNC1LI2	4.92E-43	0.329584	0.156	0.114	1.61E-38	CD14_Mor	DYNC1LI2
CEBPG	5.08E-43	0.289849	0.157	0.115	1.66E-38	CD14_Mor	CEBPG
HELZ	7.86E-43	0.352488	0.219	0.173	2.57E-38	CD14_Mor	HELZ
SLC38A2	1.14E-42	0.370788	0.255	0.209	3.73E-38	CD14_Mor	SLC38A2
NT5C2	3.16E-42	0.2564	0.121	0.082	1.04E-37	CD14_Mor	NT5C2
CPSF2	6.14E-42	0.306686	0.158	0.117	2.01E-37	CD14_Mor	CPSF2
MYEOV2	1.27E-41	0.402627	0.45	0.434	4.15E-37	CD14_Mor	MYEOV2
GLTP	2.58E-41	0.395455	0.225	0.181	8.44E-37	CD14_Mor	GLTP
ERBB2IP	3.41E-41	0.300496	0.213	0.169	1.12E-36	CD14_Mor	ERBB2IP
MIDN	3.83E-41	0.355041	0.2	0.155	1.25E-36	CD14_Mor	MIDN
HCLS1	9.10E-41	0.367336	0.582	0.59	2.98E-36	CD14_Mor	HCLS1
DYNC1I2	1.16E-40	0.396762	0.272	0.231	3.81E-36	CD14_Mor	DYNC1I2
SYF2	1.30E-40	0.422718	0.447	0.437	4.26E-36	CD14_Mor	SYF2
NDUFA11	1.45E-40	0.403162	0.319	0.281	4.73E-36	CD14_Mor	NDUFA11
ETS2	2.61E-40	0.283497	0.114	0.078	8.53E-36	CD14_Mor	ETS2
RNF213	2.65E-40	0.364555	0.539	0.532	8.67E-36	CD14_Mor	RNF213
SPCS3	2.93E-40	0.370605	0.319	0.279	9.59E-36	CD14_Mor	SPCS3
DNAJC101	5.73E-40	0.285613	0.159	0.118	1.88E-35	CD14_Mor	DNAJC10
NBPF14	1.05E-39	0.253168	0.12	0.083	3.43E-35	CD14_Mor	NBPF14

CTBP2	1.73E-39	0.283424	0.169	0.126	5.65E-35	CD14_Mor	CTBP2
IARS2	2.10E-39	0.38653	0.223	0.181	6.88E-35	CD14_Mor	IARS2
TLR1	2.92E-39	0.278106	0.119	0.082	9.57E-35	CD14_Mor	TLR1
LARP4B	8.38E-39	0.284957	0.135	0.097	2.74E-34	CD14_Mor	LARP4B
EIF3K	1.04E-38	0.279713	0.691	0.715	3.40E-34	CD14_Mor	EIF3K
TMEM256	1.25E-38	0.394118	0.338	0.303	4.10E-34	CD14_Mor	TMEM256
AKAP10	1.93E-38	0.283203	0.133	0.095	6.32E-34	CD14_Mor	AKAP10
NDUFV3	2.22E-38	0.337126	0.2	0.159	7.28E-34	CD14_Mor	NDUFV3
TSEN34	2.73E-38	0.362656	0.195	0.154	8.92E-34	CD14_Mor	TSEN34
PBX2	3.00E-38	0.251636	0.125	0.088	9.81E-34	CD14_Mor	PBX2
TMEM165	3.25E-38	0.392657	0.263	0.224	1.07E-33	CD14_Mor	TMEM165
SPEN	9.38E-38	0.344951	0.214	0.172	3.07E-33	CD14_Mor	SPEN
MCOLN1	1.80E-37	0.274121	0.114	0.079	5.91E-33	CD14_Mor	MCOLN1
YTHDF3	2.12E-37	0.2624	0.111	0.076	6.95E-33	CD14_Mor	YTHDF3
ZNHIT1	2.99E-37	0.402811	0.381	0.356	9.79E-33	CD14_Mor	ZNHIT1
REEP3	5.09E-37	0.262393	0.156	0.117	1.67E-32	CD14_Mor	REEP3
MXD4	5.58E-37	0.256549	0.13	0.093	1.83E-32	CD14_Mor	MXD4
ANKRD13/	9.41E-37	0.344478	0.182	0.143	3.08E-32	CD14_Mor	ANKRD13A
UPP1	1.21E-36	0.394656	0.241	0.197	3.95E-32	CD14_Mor	UPP1
RNF145	1.51E-36	0.493022	0.245	0.207	4.94E-32	CD14_Mor	RNF145
LPAR6	2.26E-36	0.346568	0.22	0.177	7.38E-32	CD14_Mor	LPAR6
STK38L	2.33E-36	0.278988	0.107	0.074	7.63E-32	CD14_Mor	STK38L
UBAC1	2.34E-36	0.253746	0.168	0.129	7.66E-32	CD14_Mor	UBAC1
KDELR1	5.87E-36	0.333909	0.338	0.304	1.92E-31	CD14_Mor	KDELR1
MAPKAPK	6.45E-36	0.277977	0.236	0.192	2.11E-31	CD14_Mor	MAPKAPK3
SLC38A10	6.52E-36	0.326689	0.171	0.133	2.13E-31	CD14_Mor	SLC38A10
TM9SF3	8.25E-36	0.321615	0.205	0.165	2.70E-31	CD14_Mor	TM9SF3
COX5A	1.23E-35	0.368776	0.492	0.488	4.01E-31	CD14_Mor	COX5A
FEZ2	1.55E-35	0.298504	0.217	0.176	5.07E-31	CD14_Mor	FEZ2
RB11	1.72E-35	0.365582	0.236	0.197	5.65E-31	CD14_Mor	RB1
ABTB1	1.97E-35	0.352347	0.258	0.218	6.47E-31	CD14_Mor	ABTB1
SNX291	2.95E-35	0.262133	0.145	0.108	9.65E-31	CD14_Mor	SNX29
CHMP1B	3.25E-35	0.273418	0.133	0.097	1.06E-30	CD14_Mor	CHMP1B
DDT	4.23E-35	0.397682	0.362	0.333	1.38E-30	CD14_Mor	DDT
SLA	4.59E-35	0.441832	0.259	0.22	1.50E-30	CD14_Mor	SLA
HNRNPH2	1.00E-34	0.295823	0.153	0.116	3.28E-30	CD14_Mor	HNRNPH2
SAMSN1	1.04E-34	0.391296	0.155	0.118	3.39E-30	CD14_Mor	SAMSN1
ANAPC15	1.52E-34	0.338326	0.201	0.162	4.97E-30	CD14_Mor	ANAPC15
IGF2R	1.56E-34	0.320623	0.236	0.191	5.12E-30	CD14_Mor	IGF2R
ITPK1	1.84E-34	0.269434	0.148	0.111	6.01E-30	CD14_Mor	ITPK1
NFKBIZ	2.73E-34	0.359797	0.188	0.148	8.94E-30	CD14_Mor	NFKBIZ
DCP2	3.37E-34	0.377846	0.268	0.23	1.10E-29	CD14_Mor	DCP2
FLOT1	3.78E-34	0.34817	0.271	0.23	1.24E-29	CD14_Mor	FLOT1
CEP350	4.28E-34	0.373714	0.275	0.238	1.40E-29	CD14_Mor	CEP350
USP48	4.82E-34	0.345563	0.192	0.154	1.58E-29	CD14_Mor	USP48
DNTTIP1	5.39E-34	0.282264	0.159	0.123	1.77E-29	CD14_Mor	DNTTIP1
MINOS1	7.81E-34	0.39826	0.45	0.435	2.56E-29	CD14_Mor	MINOS1
SMIM4	9.27E-34	0.283055	0.154	0.117	3.04E-29	CD14_Mor	SMIM4
RAF1	1.28E-33	0.300542	0.154	0.117	4.20E-29	CD14_Mor	RAF1
HM13	1.69E-33	0.375129	0.344	0.314	5.53E-29	CD14_Mor	HM13
AKAP9	1.93E-33	0.416504	0.356	0.327	6.31E-29	CD14_Mor	AKAP9
WWP2	3.82E-33	0.306927	0.117	0.084	1.25E-28	CD14_Mor	WWP2
PRKAR2A	3.83E-33	0.322772	0.173	0.136	1.25E-28	CD14_Mor	PRKAR2A
CD46	4.99E-33	0.411239	0.306	0.273	1.63E-28	CD14_Mor	CD46
NANS	8.55E-33	0.250044	0.154	0.118	2.80E-28	CD14_Mor	NANS
RALB	2.03E-32	0.271645	0.169	0.132	6.63E-28	CD14_Mor	RALB
NDUFB9	8.37E-32	0.424665	0.481	0.49	2.74E-27	CD14_Mor	NDUFB9
PGP	8.63E-32	0.31558	0.162	0.126	2.83E-27	CD14_Mor	PGP

MYCBP2	9.92E-32	0.360112	0.36	0.334	3.25E-27	CD14_Mor	MYCBP2
RPS6KA3	1.68E-31	0.30836	0.169	0.134	5.50E-27	CD14_Mor	RPS6KA3
NQO2	1.83E-31	0.298019	0.13	0.097	5.99E-27	CD14_Mor	NQO2
ETF1	2.90E-31	0.270074	0.147	0.112	9.50E-27	CD14_Mor	ETF1
LMO4	3.62E-31	0.360462	0.244	0.208	1.18E-26	CD14_Mor	LMO4
PTPN2	4.65E-31	0.430439	0.298	0.267	1.52E-26	CD14_Mor	PTPN2
MDM4	1.02E-30	0.339714	0.185	0.149	3.33E-26	CD14_Mor	MDM4
AFTPH	2.94E-30	0.306931	0.187	0.152	9.63E-26	CD14_Mor	AFTPH
AHCYL1	3.44E-30	0.273348	0.17	0.135	1.13E-25	CD14_Mor	AHCYL1
TACC1	6.97E-30	0.265335	0.292	0.255	2.28E-25	CD14_Mor	TACC1
SIPA1L1	7.85E-30	0.290333	0.107	0.077	2.57E-25	CD14_Mor	SIPA1L1
YY1	1.34E-29	0.31668	0.361	0.334	4.39E-25	CD14_Mor	YY1
SPG11	1.38E-29	0.318394	0.181	0.146	4.52E-25	CD14_Mor	SPG11
KIDINS22C	1.75E-29	0.257415	0.168	0.133	5.71E-25	CD14_Mor	KIDINS220
PABPN1	4.95E-29	0.254027	0.229	0.192	1.62E-24	CD14_Mor	PABPN1
ADSS	1.13E-28	0.4089	0.278	0.248	3.70E-24	CD14_Mor	ADSS
CANX	2.66E-28	0.288301	0.402	0.382	8.70E-24	CD14_Mor	CANX
KPNA6	5.55E-28	0.257448	0.131	0.1	1.82E-23	CD14_Mor	KPNA6
KIF22	6.34E-28	0.304255	0.169	0.136	2.08E-23	CD14_Mor	KIF22
PTBP3	7.74E-28	0.357285	0.297	0.267	2.53E-23	CD14_Mor	PTBP3
ANP32A	1.10E-27	0.490962	0.418	0.409	3.60E-23	CD14_Mor	ANP32A
STAT1	1.16E-27	0.337959	0.287	0.254	3.81E-23	CD14_Mor	STAT1
POMP	1.28E-27	0.374824	0.431	0.42	4.18E-23	CD14_Mor	POMP
HIATL1	1.97E-27	0.275633	0.129	0.098	6.45E-23	CD14_Mor	HIATL1
SNX21	2.98E-27	0.302761	0.308	0.276	9.76E-23	CD14_Mor	SNX2
PUM1	4.49E-27	0.334348	0.201	0.169	1.47E-22	CD14_Mor	PUM1
ZSCAN16-	7.97E-27	0.299833	0.205	0.171	2.61E-22	CD14_Mor	ZSCAN16-AS1
XPO6	1.33E-26	0.286106	0.123	0.093	4.35E-22	CD14_Mor	XPO6
SLC2A6	1.96E-26	0.27446	0.133	0.102	6.43E-22	CD14_Mor	SLC2A6
KIAA0430	7.38E-26	0.276861	0.155	0.124	2.42E-21	CD14_Mor	KIAA0430
STAT5A	7.69E-26	0.293968	0.15	0.119	2.52E-21	CD14_Mor	STAT5A
KDM5A	8.13E-26	0.377545	0.293	0.264	2.66E-21	CD14_Mor	KDM5A
ZNF524	9.45E-26	0.285165	0.19	0.158	3.09E-21	CD14_Mor	ZNF524
MAN2B1	1.01E-25	0.261672	0.248	0.215	3.30E-21	CD14_Mor	MAN2B1
SEC61G	1.03E-25	0.395329	0.488	0.493	3.36E-21	CD14_Mor	SEC61G
HDAC7	1.40E-25	0.261442	0.126	0.095	4.57E-21	CD14_Mor	HDAC7
PCNX	5.39E-25	0.309267	0.109	0.081	1.76E-20	CD14_Mor	PCNX
MYO1G	7.49E-25	0.328888	0.376	0.348	2.45E-20	CD14_Mor	MYO1G
CCNL1	1.18E-24	0.430074	0.293	0.269	3.88E-20	CD14_Mor	CCNL1
MT1F	3.63E-24	0.312791	0.123	0.093	1.19E-19	CD14_Mor	MT1F
ETHE1	3.91E-24	0.307491	0.219	0.187	1.28E-19	CD14_Mor	ETHE1
CYTH4	6.24E-24	0.434216	0.248	0.22	2.04E-19	CD14_Mor	CYTH4
TAPBP	8.81E-24	0.371299	0.421	0.41	2.88E-19	CD14_Mor	TAPBP
GSK3B	1.12E-23	0.319789	0.205	0.174	3.68E-19	CD14_Mor	GSK3B
NBPF15	1.47E-23	0.27345	0.158	0.129	4.81E-19	CD14_Mor	NBPF15
ETFB	2.26E-23	0.319165	0.248	0.22	7.38E-19	CD14_Mor	ETFB
ADAR	2.27E-23	0.411355	0.335	0.315	7.42E-19	CD14_Mor	ADAR
MIIP	3.22E-23	0.285332	0.156	0.127	1.05E-18	CD14_Mor	MIIP
GNG10	4.79E-23	0.361181	0.19	0.161	1.57E-18	CD14_Mor	GNG10
CD97	8.48E-23	0.33932	0.413	0.388	2.78E-18	CD14_Mor	CD97
RNASEH2(	9.30E-23	0.313283	0.265	0.236	3.04E-18	CD14_Mor	RNASEH2C
CTBS	1.34E-22	0.330917	0.174	0.146	4.38E-18	CD14_Mor	CTBS
RBM23	1.50E-22	0.360386	0.21	0.181	4.92E-18	CD14_Mor	RBM23
HN1	2.37E-22	0.519796	0.332	0.315	7.75E-18	CD14_Mor	HN1
USP8	6.23E-22	0.330772	0.21	0.181	2.04E-17	CD14_Mor	USP8
PTRHD1	6.66E-22	0.287892	0.26	0.231	2.18E-17	CD14_Mor	PTRHD1
NIPBL	9.12E-22	0.346832	0.299	0.276	2.98E-17	CD14_Mor	NIPBL
NRBF2	9.94E-22	0.372051	0.187	0.159	3.26E-17	CD14_Mor	NRBF2

PAXBP1	1.31E-21	0.273262	0.13	0.104	4.29E-17	CD14_Mor	PAXBP1
GRIPAP1	1.82E-21	0.250486	0.137	0.11	5.95E-17	CD14_Mor	GRIPAP1
RPS6KA1	3.77E-21	0.426232	0.278	0.253	1.23E-16	CD14_Mor	RPS6KA1
LRPAP1	4.17E-21	0.324083	0.281	0.257	1.36E-16	CD14_Mor	LRPAP1
GMIP	4.33E-21	0.335212	0.201	0.173	1.42E-16	CD14_Mor	GMIP
CCDC23	5.23E-21	0.293016	0.177	0.149	1.71E-16	CD14_Mor	CCDC23
SREK1IP1	5.48E-21	0.373742	0.236	0.209	1.79E-16	CD14_Mor	SREK1IP1
LASP1	9.22E-21	0.270855	0.245	0.215	3.02E-16	CD14_Mor	LASP1
MKKS	1.85E-20	0.270572	0.173	0.145	6.07E-16	CD14_Mor	MKKS
NCKAP1L	2.18E-20	0.326538	0.277	0.252	7.15E-16	CD14_Mor	NCKAP1L
DPEP2	2.63E-20	0.292836	0.161	0.134	8.60E-16	CD14_Mor	DPEP2
CAPZA1	2.84E-20	0.336089	0.383	0.372	9.30E-16	CD14_Mor	CAPZA1
WAC-AS1	3.31E-20	0.275495	0.177	0.149	1.08E-15	CD14_Mor	WAC-AS1
ATP2B1	4.39E-20	0.31613	0.262	0.235	1.44E-15	CD14_Mor	ATP2B1
N4BP1	5.47E-20	0.291422	0.109	0.085	1.79E-15	CD14_Mor	N4BP1
ZKSCAN1	6.15E-20	0.299933	0.175	0.148	2.01E-15	CD14_Mor	ZKSCAN1
MT1X	9.64E-20	0.432044	0.198	0.17	3.15E-15	CD14_Mor	MT1X
PARL	1.51E-19	0.30181	0.196	0.169	4.93E-15	CD14_Mor	PARL
EXOC1	1.55E-19	0.270923	0.155	0.129	5.06E-15	CD14_Mor	EXOC1
FAM96A1	1.61E-19	0.312048	0.291	0.268	5.28E-15	CD14_Mor	FAM96A
DNAJC15	1.92E-19	0.272928	0.355	0.338	6.27E-15	CD14_Mor	DNAJC15
TUBGCP2	1.95E-19	0.283846	0.213	0.186	6.37E-15	CD14_Mor	TUBGCP2
STAG2	2.20E-19	0.347836	0.32	0.302	7.21E-15	CD14_Mor	STAG2
UQCR10	3.11E-19	0.30026	0.576	0.615	1.02E-14	CD14_Mor	UQCR10
BAZ1A	4.59E-19	0.39406	0.305	0.287	1.50E-14	CD14_Mor	BAZ1A
POR	1.19E-18	0.259998	0.13	0.105	3.89E-14	CD14_Mor	POR
EEA1	1.46E-18	0.26139	0.176	0.15	4.77E-14	CD14_Mor	EEA1
RBM33	1.61E-18	0.274377	0.165	0.139	5.26E-14	CD14_Mor	RBM33
SF3B5	2.84E-18	0.349701	0.394	0.391	9.30E-14	CD14_Mor	SF3B5
PSMA4	4.42E-18	0.314865	0.327	0.307	1.45E-13	CD14_Mor	PSMA4
HEXA	4.66E-18	0.261831	0.19	0.162	1.53E-13	CD14_Mor	HEXA
TDG	5.19E-18	0.310961	0.193	0.168	1.70E-13	CD14_Mor	TDG
CHD4	8.18E-18	0.274042	0.235	0.211	2.68E-13	CD14_Mor	CHD4
CARD8	8.64E-18	0.287863	0.218	0.193	2.83E-13	CD14_Mor	CARD8
DHX8	1.19E-17	0.264701	0.137	0.113	3.90E-13	CD14_Mor	DHX8
TIMM13	1.32E-17	0.299057	0.238	0.215	4.32E-13	CD14_Mor	TIMM13
AKNA	1.62E-17	0.344373	0.302	0.281	5.31E-13	CD14_Mor	AKNA
GINM1	2.46E-17	0.255904	0.165	0.14	8.04E-13	CD14_Mor	GINM1
DGAT1	4.26E-17	0.27638	0.118	0.095	1.39E-12	CD14_Mor	DGAT1
ME2	6.39E-17	0.291567	0.183	0.16	2.09E-12	CD14_Mor	ME2
SCNM1	7.97E-17	0.322887	0.234	0.211	2.61E-12	CD14_Mor	SCNM1
FAM105B	8.27E-17	0.306109	0.138	0.115	2.71E-12	CD14_Mor	FAM105B
KIAA1109	1.58E-16	0.295786	0.158	0.135	5.16E-12	CD14_Mor	KIAA1109
CNOT1	2.11E-16	0.256623	0.168	0.144	6.91E-12	CD14_Mor	CNOT1
BAZ2A	2.98E-16	0.291399	0.221	0.198	9.76E-12	CD14_Mor	BAZ2A
DNAJC7	3.59E-16	0.307718	0.289	0.27	1.17E-11	CD14_Mor	DNAJC7
COPE	4.55E-16	0.271986	0.452	0.464	1.49E-11	CD14_Mor	COPE
TAF11	4.66E-16	0.279001	0.198	0.175	1.52E-11	CD14_Mor	TAF11
GDI2	5.15E-16	0.293258	0.438	0.43	1.68E-11	CD14_Mor	GDI2
APOL6	5.45E-16	0.28994	0.234	0.21	1.78E-11	CD14_Mor	APOL6
NBR1	6.16E-16	0.255706	0.182	0.158	2.02E-11	CD14_Mor	NBR1
YIPF4	7.33E-16	0.30653	0.199	0.176	2.40E-11	CD14_Mor	YIPF4
CAT	8.09E-16	0.272836	0.298	0.274	2.65E-11	CD14_Mor	CAT
SLC2A3	1.13E-15	0.368293	0.23	0.205	3.68E-11	CD14_Mor	SLC2A3
RNF13	1.26E-15	0.31072	0.262	0.241	4.14E-11	CD14_Mor	RNF13
GYG1	1.80E-15	0.474058	0.22	0.199	5.90E-11	CD14_Mor	GYG1
GOLGB1	2.44E-15	0.259409	0.201	0.179	8.00E-11	CD14_Mor	GOLGB1
MARK2	3.28E-15	0.272051	0.159	0.136	1.07E-10	CD14_Mor	MARK2

DTX3L	3.41E-15	0.269725	0.164	0.141	1.12E-10	CD14_Mor	DTX3L
POLK	3.90E-15	0.251948	0.13	0.109	1.28E-10	CD14_Mor	POLK
RALBP1	4.31E-15	0.311444	0.252	0.232	1.41E-10	CD14_Mor	RALBP1
SDCCAG8	6.28E-15	0.279517	0.166	0.144	2.06E-10	CD14_Mor	SDCCAG8
RNF44	9.24E-15	0.270799	0.16	0.138	3.03E-10	CD14_Mor	RNF44
ZFP36	1.47E-14	0.370751	0.422	0.429	4.82E-10	CD14_Mor	ZFP36
SIPA1	1.87E-14	0.328482	0.205	0.183	6.11E-10	CD14_Mor	SIPA1
RBMS1	2.30E-14	0.380705	0.332	0.323	7.53E-10	CD14_Mor	RBMS1
PRDX3	4.98E-14	0.309009	0.28	0.265	1.63E-09	CD14_Mor	PRDX3
DPM3	5.12E-14	0.256342	0.252	0.232	1.68E-09	CD14_Mor	DPM3
TMEM131	5.63E-14	0.267284	0.146	0.125	1.84E-09	CD14_Mor	TMEM131
TMPO	5.96E-14	0.270714	0.25	0.229	1.95E-09	CD14_Mor	TMPO
C1orf63	6.14E-14	0.388134	0.446	0.456	2.01E-09	CD14_Mor	C1orf63
RNF169	7.83E-14	0.25031	0.131	0.11	2.56E-09	CD14_Mor	RNF169
NUP50	9.05E-14	0.261077	0.175	0.153	2.96E-09	CD14_Mor	NUP50
NMI	1.03E-13	0.309963	0.225	0.206	3.38E-09	CD14_Mor	NMI
ATP6AP2	1.17E-13	0.293265	0.352	0.347	3.82E-09	CD14_Mor	ATP6AP2
EXOC6	1.52E-13	0.254292	0.1	0.082	4.97E-09	CD14_Mor	EXOC6
CHD8	2.06E-13	0.269239	0.154	0.133	6.76E-09	CD14_Mor	CHD8
SHFM1	3.54E-13	0.309468	0.449	0.462	1.16E-08	CD14_Mor	SHFM1
TMEM183,	4.47E-13	0.267026	0.203	0.183	1.46E-08	CD14_Mor	TMEM183A
UBR2	6.14E-13	0.270057	0.171	0.151	2.01E-08	CD14_Mor	UBR2
MTMR14	7.77E-13	0.263211	0.205	0.185	2.54E-08	CD14_Mor	MTMR14
LBR	9.63E-13	0.357631	0.262	0.245	3.15E-08	CD14_Mor	LBR
BPTF	1.72E-12	0.290959	0.291	0.276	5.62E-08	CD14_Mor	BPTF
PHIP	4.51E-12	0.312377	0.249	0.233	1.48E-07	CD14_Mor	PHIP
MRPL41	5.10E-12	0.346965	0.338	0.331	1.67E-07	CD14_Mor	MRPL41
NUPL1	6.35E-12	0.280893	0.127	0.109	2.08E-07	CD14_Mor	NUPL1
ARRDC1	7.56E-12	0.300897	0.214	0.197	2.47E-07	CD14_Mor	ARRDC1
TRAPPC2L	9.05E-12	0.304023	0.205	0.188	2.96E-07	CD14_Mor	TRAPPC2L
TNRC6A	1.04E-11	0.274585	0.171	0.151	3.42E-07	CD14_Mor	TNRC6A
CASP4	1.32E-11	0.296048	0.364	0.358	4.32E-07	CD14_Mor	CASP4
SMC1A	1.79E-11	0.252184	0.222	0.204	5.87E-07	CD14_Mor	SMC1A
COA3	2.59E-11	0.285568	0.239	0.223	8.48E-07	CD14_Mor	COA3
RAB2A	1.24E-10	0.305665	0.346	0.344	4.04E-06	CD14_Mor	RAB2A
ZNF217	1.34E-10	0.259862	0.196	0.179	4.39E-06	CD14_Mor	ZNF217
MKNK2	3.64E-10	0.317314	0.223	0.209	1.19E-05	CD14_Mor	MKNK2
SNF8	5.98E-10	0.297911	0.265	0.254	1.96E-05	CD14_Mor	SNF8
EIF2S2	1.08E-09	0.302596	0.414	0.425	3.55E-05	CD14_Mor	EIF2S2
DYNC1LI1	1.53E-09	0.2741	0.17	0.155	5.02E-05	CD14_Mor	DYNC1LI1
ARL6IP4	3.11E-09	0.280334	0.517	0.542	0.000102	CD14_Mor	ARL6IP4
ARHGAP4	3.55E-09	0.335623	0.288	0.28	0.000116	CD14_Mor	ARHGAP4
TNRC6B	3.81E-09	0.310158	0.297	0.289	0.000125	CD14_Mor	TNRC6B
MRPS5	3.91E-09	0.297841	0.253	0.242	0.000128	CD14_Mor	MRPS5
SNX18	3.92E-09	0.264696	0.164	0.148	0.000128	CD14_Mor	SNX18
ALOX5AP	5.07E-09	0.653654	0.318	0.32	0.000166	CD14_Mor	ALOX5AP
METAP2	6.77E-09	0.301844	0.297	0.29	0.000222	CD14_Mor	METAP2
DYNC1H1	1.00E-08	0.266484	0.26	0.249	0.000327	CD14_Mor	DYNC1H1
NMT1	1.25E-08	0.286915	0.182	0.168	0.00041	CD14_Mor	NMT1
RUNX1	1.98E-08	0.260446	0.143	0.128	0.000647	CD14_Mor	RUNX1
CIR1	2.76E-08	0.319731	0.275	0.268	0.000905	CD14_Mor	CIR1
KTN1	2.79E-08	0.29505	0.42	0.435	0.000914	CD14_Mor	KTN1
TANK	4.45E-08	0.26923	0.207	0.195	0.001458	CD14_Mor	TANK
HADHA	6.29E-08	0.287196	0.412	0.427	0.002059	CD14_Mor	HADHA
C19orf24	1.24E-07	0.307915	0.233	0.223	0.004044	CD14_Mor	C19orf24
TBCA	1.49E-07	0.344528	0.473	0.498	0.004864	CD14_Mor	TBCA
STK17B	3.26E-07	0.375994	0.361	0.366	0.01066	CD14_Mor	STK17B
SSR1	3.26E-07	0.251782	0.304	0.3	0.010669	CD14_Mor	SSR1

SSH2	1.95E-06	0.262508	0.281	0.273	0.063751	CD14_Mor	SSH2
SLTM	2.05E-06	0.301055	0.315	0.315	0.0672	CD14_Mor	SLTM
VPS35	2.82E-06	0.275291	0.306	0.301	0.092402	CD14_Mor	VPS35
RTF1	3.55E-06	0.32872	0.278	0.275	0.116317	CD14_Mor	RTF1
SRRM1	4.76E-06	0.269622	0.462	0.489	0.155927	CD14_Mor	SRRM1
ZNF207	5.74E-06	0.307829	0.324	0.328	0.187818	CD14_Mor	ZNF207
BCL2A1	1.25E-05	0.250481	0.177	0.164	0.4099	CD14_Mor	BCL2A1
SNRNP70	1.53E-05	0.300807	0.237	0.231	0.501181	CD14_Mor	SNRNP70
PLAC8	1.70E-05	0.432836	0.392	0.401	0.555347	CD14_Mor	PLAC8
PKN1	2.46E-05	0.259296	0.249	0.242	0.803993	CD14_Mor	PKN1
GIT2	3.02E-05	0.280313	0.207	0.2	0.988956	CD14_Mor	GIT2
PRPF40A	4.01E-05	0.263565	0.337	0.342	1	CD14_Mor	PRPF40A
IRF1	7.62E-05	0.266639	0.475	0.489	1	CD14_Mor	IRF1
GBP2	0.000149	0.281276	0.263	0.259	1	CD14_Mor	GBP2
ERGIC3	0.000154	0.266834	0.335	0.343	1	CD14_Mor	ERGIC3
MTIF3	0.000156	0.269249	0.251	0.248	1	CD14_Mor	MTIF3
HNRNPU	0.000616	0.293562	0.472	0.507	1	CD14_Mor	HNRNPU
TLE4	0.000853	0.252613	0.217	0.212	1	CD14_Mor	TLE4
RAE1	0.000854	0.262479	0.14	0.134	1	CD14_Mor	RAE1
NDUFB7	0.002169	0.266195	0.4	0.42	1	CD14_Mor	NDUFB7
DDX46	0.0038	0.272867	0.334	0.346	1	CD14_Mor	DDX46
C8orf59	0.004382	0.312252	0.424	0.453	1	CD14_Mor	C8orf59
COX17	0.004804	0.259586	0.374	0.389	1	CD14_Mor	COX17
ARHGAP9	0.005156	0.301142	0.265	0.267	1	CD14_Mor	ARHGAP9
EIF3M	0.005207	0.276341	0.422	0.451	1	CD14_Mor	EIF3M
FCGR3A	0	3.235915	0.974	0.125	0	CD16_Mor	FCGR3A
CDKN1C	0	2.62778	0.78	0.01	0	CD16_Mor	CDKN1C
MS4A7	0	2.576883	0.958	0.234	0	CD16_Mor	MS4A7
RP11-290I	0	2.375671	0.945	0.199	0	CD16_Mor	RP11-290F20.3
IFITM3	0	2.196818	0.979	0.439	0	CD16_Mor	IFITM3
LST11	0	2.195449	0.999	0.549	0	CD16_Mor	LST1
LYPD2	0	2.101625	0.272	0.001	0	CD16_Mor	LYPD2
C1QA	0	2.002887	0.349	0.011	0	CD16_Mor	C1QA
HMOX1	0	1.865485	0.855	0.17	0	CD16_Mor	HMOX1
MTSS11	0	1.85401	0.837	0.113	0	CD16_Mor	MTSS1
RHOC	0	1.73888	0.828	0.156	0	CD16_Mor	RHOC
SAT1	0	1.728312	0.986	0.611	0	CD16_Mor	SAT1
CSF1R1	0	1.726001	0.863	0.174	0	CD16_Mor	CSF1R
HLA-DPA1	0	1.7255	0.976	0.418	0	CD16_Mor	HLA-DPA1
TCF7L2	0	1.638997	0.734	0.058	0	CD16_Mor	TCF7L2
CFD1	0	1.563294	0.955	0.386	0	CD16_Mor	CFD
RHOB	0	1.531933	0.714	0.082	0	CD16_Mor	RHOB
C1QB	0	1.517011	0.134	0.003	0	CD16_Mor	C1QB
WARS1	0	1.484783	0.829	0.213	0	CD16_Mor	WARS
HLA-DRB5	0	1.474782	0.811	0.29	0	CD16_Mor	HLA-DRB5
MAFB1	0	1.462227	0.824	0.18	0	CD16_Mor	MAFB
COTL11	0	1.452068	0.997	0.71	0	CD16_Mor	COTL1
AIF11	0	1.447352	0.999	0.611	0	CD16_Mor	AIF1
LYN1	0	1.443061	0.93	0.385	0	CD16_Mor	LYN
BCL2A11	0	1.439425	0.747	0.138	0	CD16_Mor	BCL2A1
SERPINA1	0	1.423953	0.981	0.505	0	CD16_Mor	SERPINA1
ABI3	0	1.386232	0.785	0.164	0	CD16_Mor	ABI3
SIGLEC10	0	1.362122	0.675	0.071	0	CD16_Mor	SIGLEC10
LILRB21	0	1.345728	0.917	0.319	0	CD16_Mor	LILRB2
NAAA1	0	1.341442	0.864	0.282	0	CD16_Mor	NAAA
HES4	0	1.340481	0.511	0.003	0	CD16_Mor	HES4
PSAP1	0	1.3371	0.994	0.704	0	CD16_Mor	PSAP
FAM26F2	0	1.325598	0.575	0.16	0	CD16_Mor	FAM26F

FCER1G1	0	1.308735	0.997	0.683	0	CD16_Mor	FCER1G
RNASET21	0	1.303363	0.942	0.436	0	CD16_Mor	RNASET2
GPBAR11	0	1.278586	0.694	0.113	0	CD16_Mor	GPBAR1
SPN	0	1.269805	0.811	0.249	0	CD16_Mor	SPN
PILRA1	0	1.266144	0.849	0.248	0	CD16_Mor	PILRA
LRRC251	0	1.257315	0.825	0.215	0	CD16_Mor	LRRC25
BID	0	1.226681	0.799	0.224	0	CD16_Mor	BID
INSIG1	0	1.213715	0.614	0.089	0	CD16_Mor	INSIG1
CUX1	0	1.211713	0.746	0.242	0	CD16_Mor	CUX1
NPC21	0	1.19332	0.948	0.475	0	CD16_Mor	NPC2
HLA-DPB1	0	1.186812	0.92	0.397	0	CD16_Mor	HLA-DPB1
HLA-DRB1	0	1.181212	0.965	0.487	0	CD16_Mor	HLA-DRB1
TNFRSF1B	0	1.175211	0.948	0.441	0	CD16_Mor	TNFRSF1B
IFITM2	0	1.172427	0.991	0.775	0	CD16_Mor	IFITM2
DRAP1	0	1.157825	0.918	0.425	0	CD16_Mor	DRAP1
FTL1	0	1.15762	1	0.979	0	CD16_Mor	FTL
HCK1	0	1.150869	0.91	0.352	0	CD16_Mor	HCK
CEBPB1	0	1.147094	0.92	0.388	0	CD16_Mor	CEBPB
SPI11	0	1.130727	0.968	0.478	0	CD16_Mor	SPI1
LILRB11	0	1.113489	0.802	0.218	0	CD16_Mor	LILRB1
CD68	0	1.111849	0.918	0.391	0	CD16_Mor	CD68
LY6E	0	1.104708	0.841	0.347	0	CD16_Mor	LY6E
CTSC	0	1.098657	0.858	0.372	0	CD16_Mor	CTSC
CKB	0	1.096494	0.359	0.008	0	CD16_Mor	CKB
PTPN62	0	1.088079	0.926	0.492	0	CD16_Mor	PTPN6
IFI301	0	1.079501	0.81	0.258	0	CD16_Mor	IFI30
CST31	0	1.078455	0.998	0.661	0	CD16_Mor	CST3
FAM110A	0	1.077754	0.712	0.152	0	CD16_Mor	FAM110A
IER5	0	1.064819	0.642	0.112	0	CD16_Mor	IER5
HLA-DRA1	0	1.044307	0.97	0.469	0	CD16_Mor	HLA-DRA
CAMK1	0	1.04012	0.62	0.096	0	CD16_Mor	CAMK1
RPS191	0	1.02118	0.997	0.806	0	CD16_Mor	RPS19
LILRA31	0	1.021068	0.528	0.105	0	CD16_Mor	LILRA3
CSTB1	0	1.016034	0.927	0.52	0	CD16_Mor	CSTB
HLA-DQA	0	1.012639	0.693	0.162	0	CD16_Mor	HLA-DQA1
NFKBIZ1	0	1.011277	0.654	0.142	0	CD16_Mor	NFKBIZ
TUBA1B	0	1.005605	0.887	0.419	0	CD16_Mor	TUBA1B
LILRA51	0	0.997825	0.808	0.264	0	CD16_Mor	LILRA5
SYNGR21	0	0.990799	0.847	0.347	0	CD16_Mor	SYNGR2
YBX1	0	0.98896	0.968	0.669	0	CD16_Mor	YBX1
OAS11	0	0.988959	0.645	0.158	0	CD16_Mor	OAS1
MARCKSL1	0	0.968868	0.505	0.127	0	CD16_Mor	MARCKSL1
CTSL	0	0.962956	0.438	0.025	0	CD16_Mor	CTSL
SCIMP1	0	0.954963	0.712	0.182	0	CD16_Mor	SCIMP
EMR21	0	0.952344	0.667	0.141	0	CD16_Mor	EMR2
CXCL16	0	0.952145	0.562	0.088	0	CD16_Mor	CXCL16
LILRA11	0	0.944992	0.656	0.144	0	CD16_Mor	LILRA1
RP11-362I	0	0.933815	0.418	0.026	0	CD16_Mor	RP11-362F19.1
UNC119	0	0.930018	0.616	0.141	0	CD16_Mor	UNC119
CALM2	0	0.925036	0.954	0.654	0	CD16_Mor	CALM2
RGS19	0	0.910826	0.81	0.314	0	CD16_Mor	RGS19
DUSP5	0	0.905986	0.374	0.029	0	CD16_Mor	DUSP5
CD741	0	0.90424	0.997	0.763	0	CD16_Mor	CD74
VMO1	0	0.902707	0.227	0.001	0	CD16_Mor	VMO1
CHCHD10	0	0.892515	0.816	0.327	0	CD16_Mor	CHCHD10
SLC7A71	0	0.891333	0.748	0.237	0	CD16_Mor	SLC7A7
TBC1D8	0	0.886554	0.505	0.064	0	CD16_Mor	TBC1D8
C5AR11	0	0.882955	0.707	0.173	0	CD16_Mor	C5AR1

PRELID11	0	0.876707	0.935	0.536	0	CD16_Mor	PRELID1
CX3CR1	0	0.875665	0.717	0.226	0	CD16_Mor	CX3CR1
CALML4	0	0.872283	0.458	0.039	0	CD16_Mor	CALML4
MAPKAPK:	0	0.868207	0.677	0.19	0	CD16_Mor	MAPKAPK3
CD521	0	0.865681	0.954	0.634	0	CD16_Mor	CD52
SIDT2	0	0.864046	0.54	0.087	0	CD16_Mor	SIDT2
POU2F21	0	0.862048	0.9	0.397	0	CD16_Mor	POU2F2
CASP11	0	0.85739	0.835	0.348	0	CD16_Mor	CASP1
ZFAND51	0	0.854755	0.764	0.282	0	CD16_Mor	ZFAND5
SLC2A61	0	0.849865	0.531	0.096	0	CD16_Mor	SLC2A6
RRAS	0	0.84356	0.502	0.074	0	CD16_Mor	RRAS
NR4A1	0	0.833114	0.332	0.008	0	CD16_Mor	NR4A1
RAB241	0	0.832033	0.687	0.215	0	CD16_Mor	RAB24
ITGA4	0	0.827316	0.751	0.294	0	CD16_Mor	ITGA4
ATG31	0	0.825922	0.79	0.312	0	CD16_Mor	ATG3
ZNF703	0	0.822291	0.413	0.025	0	CD16_Mor	ZNF703
BATF3	0	0.821989	0.377	0.024	0	CD16_Mor	BATF3
NAP1L1	0	0.820995	0.951	0.513	0	CD16_Mor	NAP1L1
RNH1	0	0.818381	0.83	0.349	0	CD16_Mor	RNH1
KLF41	0	0.815048	0.531	0.106	0	CD16_Mor	KLF4
PIK3AP11	0	0.813336	0.675	0.192	0	CD16_Mor	PIK3AP1
STXBP21	0	0.800599	0.921	0.491	0	CD16_Mor	STXBP2
TUBA1A1	0	0.799487	0.82	0.38	0	CD16_Mor	TUBA1A
CFP1	0	0.797123	0.858	0.363	0	CD16_Mor	CFP
FTH1	0	0.795131	1	0.968	0	CD16_Mor	FTH1
UTRN	0	0.793433	0.704	0.254	0	CD16_Mor	UTRN
VASP1	0	0.792571	0.855	0.393	0	CD16_Mor	VASP
DUSP61	0	0.790629	0.885	0.349	0	CD16_Mor	DUSP6
MYD881	0	0.786956	0.682	0.213	0	CD16_Mor	MYD88
MARCKS1	0	0.78616	0.674	0.2	0	CD16_Mor	MARCKS
PLAGL2	0	0.783909	0.43	0.056	0	CD16_Mor	PLAGL2
9-Sep	0	0.782593	0.801	0.375	0	CD16_Mor	9-Sep
TBXAS11	0	0.779321	0.776	0.27	0	CD16_Mor	TBXAS1
CSK1	0	0.775068	0.861	0.413	0	CD16_Mor	CSK
ABRACL	0	0.774314	0.833	0.418	0	CD16_Mor	ABRACL
FGD22	0	0.769822	0.597	0.15	0	CD16_Mor	FGD2
CD79B1	0	0.762363	0.444	0.052	0	CD16_Mor	CD79B
CD971	0	0.760704	0.817	0.378	0	CD16_Mor	CD97
ICAM4	0	0.75195	0.325	0.02	0	CD16_Mor	ICAM4
CYTIP	0	0.749119	0.662	0.245	0	CD16_Mor	CYTIP
APOBEC3/	0	0.743695	0.356	0.078	0	CD16_Mor	APOBEC3A
ITGAL	0	0.743233	0.673	0.227	0	CD16_Mor	ITGAL
SLC25A61	0	0.742697	0.963	0.677	0	CD16_Mor	SLC25A6
SLC25A5	0	0.741763	0.888	0.487	0	CD16_Mor	SLC25A5
ATP6V0C	0	0.740411	0.917	0.528	0	CD16_Mor	ATP6V0C
MS4A4A	0	0.739905	0.404	0.056	0	CD16_Mor	MS4A4A
PTGES3	0	0.728333	0.816	0.426	0	CD16_Mor	PTGES3
CARD161	0	0.725046	0.931	0.515	0	CD16_Mor	CARD16
DUSP11	0	0.724683	0.824	0.342	0	CD16_Mor	DUSP1
C20orf271	0	0.723889	0.666	0.214	0	CD16_Mor	C20orf27
LYST1	0	0.721695	0.818	0.339	0	CD16_Mor	LYST
MYOF1	0	0.716849	0.448	0.083	0	CD16_Mor	MYOF
NEURL1	0	0.715883	0.336	0.012	0	CD16_Mor	NEURL1
WSB11	0	0.710757	0.825	0.393	0	CD16_Mor	WSB1
S100A111	0	0.708209	0.994	0.772	0	CD16_Mor	S100A11
ZNF706	0	0.708068	0.774	0.331	0	CD16_Mor	ZNF706
CPPED11	0	0.699674	0.697	0.233	0	CD16_Mor	CPPED1
ID2	0	0.696834	0.639	0.221	0	CD16_Mor	ID2

C3AR11	0	0.694369	0.489	0.098	0	CD16_Mor	C3AR1
PKN11	0	0.69304	0.67	0.22	0	CD16_Mor	PKN1
SECTM11	0	0.684458	0.596	0.146	0	CD16_Mor	SECTM1
PGK1	0	0.683321	0.895	0.531	0	CD16_Mor	PGK1
RNF144B1	0	0.683205	0.461	0.107	0	CD16_Mor	RNF144B
CD300A	0	0.681718	0.602	0.17	0	CD16_Mor	CD300A
LGALS91	0	0.676245	0.706	0.254	0	CD16_Mor	LGALS9
TESC1	0	0.675637	0.639	0.187	0	CD16_Mor	TESC
ATP1B3	0	0.674653	0.566	0.175	0	CD16_Mor	ATP1B3
ARPC31	0	0.673014	0.987	0.79	0	CD16_Mor	ARPC3
C19orf381	0	0.672537	0.737	0.248	0	CD16_Mor	C19orf38
PAG1	0	0.671311	0.507	0.124	0	CD16_Mor	PAG1
WASF21	0	0.667689	0.89	0.457	0	CD16_Mor	WASF2
SLC31A21	0	0.66616	0.486	0.12	0	CD16_Mor	SLC31A2
JUNB1	0	0.665967	0.858	0.452	0	CD16_Mor	JUNB
GDI21	0	0.65995	0.837	0.411	0	CD16_Mor	GDI2
SH2D3C	0	0.658626	0.477	0.11	0	CD16_Mor	SH2D3C
FGR1	0	0.657021	0.89	0.437	0	CD16_Mor	FGR
CTSZ1	0	0.655693	0.873	0.408	0	CD16_Mor	CTSZ
ARPC2	0	0.65009	0.986	0.787	0	CD16_Mor	ARPC2
VPS351	0	0.648845	0.712	0.28	0	CD16_Mor	VPS35
RP11-750I	0	0.643265	0.368	0.036	0	CD16_Mor	RP11-750H9.5
ASAH11	0	0.641179	0.864	0.401	0	CD16_Mor	ASAH1
KDM1B1	0	0.631311	0.47	0.099	0	CD16_Mor	KDM1B
RNF1491	0	0.628848	0.834	0.371	0	CD16_Mor	RNF149
CMTM6	0	0.627843	0.79	0.35	0	CD16_Mor	CMTM6
IFNGR21	0	0.622711	0.756	0.306	0	CD16_Mor	IFNGR2
RHOG1	0	0.620717	0.929	0.535	0	CD16_Mor	RHOG
CDH23	0	0.613802	0.363	0.044	0	CD16_Mor	CDH23
TMPO1	0	0.611436	0.602	0.22	0	CD16_Mor	TMPO
ZBTB7A	0	0.611297	0.698	0.289	0	CD16_Mor	ZBTB7A
MYO1G1	0	0.609497	0.762	0.34	0	CD16_Mor	MYO1G
CALHM2	0	0.606619	0.448	0.118	0	CD16_Mor	CALHM2
CTD-2006	0	0.599102	0.316	0.025	0	CD16_Mor	CTD-2006K23.1
FMNL2	0	0.597822	0.277	0.006	0	CD16_Mor	FMNL2
FAM49A1	0	0.596433	0.535	0.152	0	CD16_Mor	FAM49A
PFN1	0	0.59532	0.999	0.907	0	CD16_Mor	PFN1
SNX181	0	0.593053	0.506	0.136	0	CD16_Mor	SNX18
UNC93B11	0	0.592275	0.547	0.159	0	CD16_Mor	UNC93B1
PPM1N	0	0.581915	0.252	0.012	0	CD16_Mor	PPM1N
RARA	0	0.579347	0.432	0.098	0	CD16_Mor	RARA
CD300C1	0	0.579154	0.407	0.096	0	CD16_Mor	CD300C
PHF19	0	0.572414	0.35	0.057	0	CD16_Mor	PHF19
HSBP11	0	0.571556	0.832	0.378	0	CD16_Mor	HSBP1
GCH1	0	0.570734	0.439	0.088	0	CD16_Mor	GCH1
CD861	0	0.56871	0.566	0.182	0	CD16_Mor	CD86
KLF3	0	0.568025	0.642	0.243	0	CD16_Mor	KLF3
CNIH4	0	0.564337	0.544	0.194	0	CD16_Mor	CNIH4
TIMP1	0	0.562161	0.923	0.499	0	CD16_Mor	TIMP1
CCT5	0	0.561539	0.665	0.27	0	CD16_Mor	CCT5
SMCO41	0	0.559803	0.617	0.213	0	CD16_Mor	SMCO4
SH3BP21	0	0.559554	0.615	0.206	0	CD16_Mor	SH3BP2
EPB41L3	0	0.55945	0.404	0.079	0	CD16_Mor	EPB41L3
PMVK	0	0.55855	0.442	0.113	0	CD16_Mor	PMVK
BLVRA1	0	0.555312	0.61	0.216	0	CD16_Mor	BLVRA
PLAUR1	0	0.553337	0.574	0.166	0	CD16_Mor	PLAUR
HOTAIRM1	0	0.552106	0.508	0.14	0	CD16_Mor	HOTAIRM1
SH2B31	0	0.547249	0.512	0.148	0	CD16_Mor	SH2B3

AP2A1	0	0.54578	0.54	0.162	0	CD16_Mor	AP2A1
ADA	0	0.542346	0.332	0.055	0	CD16_Mor	ADA
TNFRSF8	0	0.541417	0.286	0.022	0	CD16_Mor	TNFRSF8
DAPK11	0	0.539381	0.425	0.094	0	CD16_Mor	DAPK1
PLXNB21	0	0.537415	0.543	0.175	0	CD16_Mor	PLXNB2
RAB10	0	0.535536	0.735	0.298	0	CD16_Mor	RAB10
ACAA1	0	0.529997	0.567	0.199	0	CD16_Mor	ACAA1
C15orf391	0	0.527534	0.483	0.123	0	CD16_Mor	C15orf39
NINJ11	0	0.52609	0.583	0.214	0	CD16_Mor	NINJ1
HSPH1	0	0.518303	0.373	0.101	0	CD16_Mor	HSPH1
TNFSF131	0	0.517699	0.425	0.113	0	CD16_Mor	TNFSF13
ZDHHC1	0	0.517281	0.343	0.063	0	CD16_Mor	ZDHHC1
RNF131	0	0.515531	0.612	0.232	0	CD16_Mor	RNF13
LFNG1	0	0.50753	0.519	0.167	0	CD16_Mor	LFNG
HN11	0	0.501572	0.726	0.3	0	CD16_Mor	HN1
AMPD2	0	0.498525	0.413	0.098	0	CD16_Mor	AMPD2
TMEM134	0	0.497377	0.404	0.111	0	CD16_Mor	TMEM134
EPS8	0	0.497316	0.244	0.017	0	CD16_Mor	EPS8
NUDT161	0	0.495405	0.569	0.191	0	CD16_Mor	NUDT16
ZMIZ1	0	0.490547	0.4	0.104	0	CD16_Mor	ZMIZ1
BTK1	0	0.487339	0.496	0.138	0	CD16_Mor	BTK
PDLIM5	0	0.485947	0.485	0.134	0	CD16_Mor	PDLIM5
RABGAP1L	0	0.480682	0.566	0.197	0	CD16_Mor	RABGAP1L
CDC42EP2	0	0.477966	0.288	0.043	0	CD16_Mor	CDC42EP2
CHST2	0	0.476909	0.293	0.047	0	CD16_Mor	CHST2
CHST151	0	0.475125	0.435	0.109	0	CD16_Mor	CHST15
PTP4A3	0	0.47463	0.234	0.019	0	CD16_Mor	PTP4A3
CLEC7A1	0	0.470949	0.748	0.293	0	CD16_Mor	CLEC7A
RBBP8	0	0.46782	0.287	0.04	0	CD16_Mor	RBBP8
LILRA21	0	0.466866	0.608	0.208	0	CD16_Mor	LILRA2
ADAP21	0	0.462417	0.448	0.132	0	CD16_Mor	ADAP2
CASP5	0	0.460987	0.226	0.008	0	CD16_Mor	CASP5
MBD21	0	0.459321	0.502	0.16	0	CD16_Mor	MBD2
RELT1	0	0.456787	0.371	0.088	0	CD16_Mor	RELT
PAPSS2	0	0.45602	0.228	0.02	0	CD16_Mor	PAPSS2
SWAP701	0	0.454737	0.397	0.101	0	CD16_Mor	SWAP70
ITGAX1	0	0.453095	0.545	0.177	0	CD16_Mor	ITGAX
MPC1	0	0.452749	0.494	0.162	0	CD16_Mor	MPC1
RXRA1	0	0.450944	0.619	0.223	0	CD16_Mor	RXRA
STX11	0	0.450557	0.504	0.145	0	CD16_Mor	STX11
PHTF2	0	0.449469	0.343	0.069	0	CD16_Mor	PHTF2
ARAP2	0	0.447822	0.392	0.108	0	CD16_Mor	ARAP2
WDR11	0	0.447618	0.379	0.1	0	CD16_Mor	WDR11
MXD3	0	0.442949	0.282	0.039	0	CD16_Mor	MXD3
TAGLN	0	0.441457	0.24	0.024	0	CD16_Mor	TAGLN
ARRB1	0	0.438859	0.501	0.141	0	CD16_Mor	ARRB1
PCGF5	0	0.43734	0.482	0.15	0	CD16_Mor	PCGF5
LMO21	0	0.437031	0.536	0.178	0	CD16_Mor	LMO2
SCLT1	0	0.433376	0.448	0.131	0	CD16_Mor	SCLT1
CD300LF1	0	0.431771	0.448	0.12	0	CD16_Mor	CD300LF
ICAM2	0	0.431554	0.645	0.229	0	CD16_Mor	ICAM2
TPPP3	0	0.428388	0.235	0.037	0	CD16_Mor	TPPP3
MCOLN11	0	0.422803	0.355	0.084	0	CD16_Mor	MCOLN1
MAP3K81	0	0.419769	0.435	0.129	0	CD16_Mor	MAP3K8
SNAPC3	0	0.417405	0.307	0.067	0	CD16_Mor	SNAPC3
RNF1411	0	0.416884	0.435	0.124	0	CD16_Mor	RNF141
CKS1B	0	0.416179	0.247	0.026	0	CD16_Mor	CKS1B
EMR1	0	0.415744	0.286	0.044	0	CD16_Mor	EMR1

IFIT2	0	0.415202	0.306	0.059	0	CD16_Mor	IFIT2
RALB1	0	0.411294	0.46	0.135	0	CD16_Mor	RALB
PRAM11	0	0.406701	0.671	0.251	0	CD16_Mor	PRAM1
GPR137B	0	0.404932	0.277	0.037	0	CD16_Mor	GPR137B
SLC11A11	0	0.403964	0.723	0.281	0	CD16_Mor	SLC11A1
NEDD9	0	0.402755	0.337	0.085	0	CD16_Mor	NEDD9
FCGR3B	0	0.399093	0.277	0.021	0	CD16_Mor	FCGR3B
PRADC1	0	0.398592	0.257	0.043	0	CD16_Mor	PRADC1
SCRN1	0	0.39771	0.233	0.035	0	CD16_Mor	SCRN1
SLC1A5	0	0.396156	0.327	0.083	0	CD16_Mor	SLC1A5
IFIT3	0	0.393901	0.256	0.046	0	CD16_Mor	IFIT3
PLD41	0	0.393638	0.293	0.043	0	CD16_Mor	PLD4
EMR3	0	0.39115	0.219	0.021	0	CD16_Mor	EMR3
HK31	0	0.388345	0.556	0.178	0	CD16_Mor	HK3
KCNQ1OT	0	0.380263	0.182	0.013	0	CD16_Mor	KCNQ1OT1
ULK2	0	0.376405	0.258	0.044	0	CD16_Mor	ULK2
RP11-100٤	0	0.37551	0.134	0	0	CD16_Mor	RP11-1008C21.1
LINC00877	0	0.373123	0.265	0.049	0	CD16_Mor	LINC00877
DOCK51	0	0.37139	0.399	0.112	0	CD16_Mor	DOCK5
APH1B	0	0.370478	0.306	0.066	0	CD16_Mor	APH1B
HCAR3	0	0.368937	0.148	0.002	0	CD16_Mor	HCAR3
SFMBT2	0	0.364418	0.209	0.034	0	CD16_Mor	SFMBT2
UBASH3B	0	0.363056	0.244	0.047	0	CD16_Mor	UBASH3B
FCGR2B1	0	0.361787	0.247	0.048	0	CD16_Mor	FCGR2B
LPCAT21	0	0.356414	0.392	0.104	0	CD16_Mor	LPCAT2
MRAS	0	0.354256	0.182	0.016	0	CD16_Mor	MRAS
SPATA6	0	0.348278	0.207	0.028	0	CD16_Mor	SPATA6
TNF	0	0.348253	0.202	0.024	0	CD16_Mor	TNF
PPCDC	0	0.344318	0.231	0.039	0	CD16_Mor	PPCDC
SNX9	0	0.343794	0.306	0.069	0	CD16_Mor	SNX9
SPRED1	0	0.341178	0.189	0.011	0	CD16_Mor	SPRED1
CHST7	0	0.331144	0.19	0.022	0	CD16_Mor	CHST7
AC011899	0	0.330726	0.225	0.03	0	CD16_Mor	AC011899.9
SCGB3A1	0	0.326263	0.105	0.003	0	CD16_Mor	SCGB3A1
C20orf201	0	0.319641	0.127	0.002	0	CD16_Mor	C20orf201
CDK2AP1	0	0.31951	0.508	0.134	0	CD16_Mor	CDK2AP1
EAF21	0	0.317451	0.26	0.054	0	CD16_Mor	EAF2
IL3RA	0	0.310643	0.16	0.017	0	CD16_Mor	IL3RA
RIN1	0	0.30811	0.216	0.034	0	CD16_Mor	RIN1
SH2B2	0	0.30608	0.227	0.036	0	CD16_Mor	SH2B2
CLEC4F	0	0.301616	0.131	0.002	0	CD16_Mor	CLEC4F
HEG1	0	0.301001	0.176	0.02	0	CD16_Mor	HEG1
RP11-63P1	0	0.30068	0.201	0.032	0	CD16_Mor	RP11-63P12.6
HES1	0	0.285845	0.112	0.001	0	CD16_Mor	HES1
MSR1	0	0.284272	0.148	0.017	0	CD16_Mor	MSR1
GNGT2	0	0.279201	0.195	0.032	0	CD16_Mor	GNGT2
CD831	0	0.274814	0.15	0.018	0	CD16_Mor	CD83
PWWP2B	#######	0.276634	0.182	0.029	#######	CD16_Mor	PWWP2B
NAGA1	#######	0.450046	0.445	0.14	#######	CD16_Mor	NAGA
POLD41	#######	0.661786	0.779	0.375	#######	CD16_Mor	POLD4
PIK3CG	#######	0.341862	0.274	0.061	#######	CD16_Mor	PIK3CG
BRI31	#######	0.602344	0.954	0.537	#######	CD16_Mor	BRI3
VPS29	#######	0.580335	0.716	0.313	#######	CD16_Mor	VPS29
ARAP11	#######	0.43025	0.566	0.203	#######	CD16_Mor	ARAP1
TSPAN14	#######	0.4574	0.589	0.217	#######	CD16_Mor	TSPAN14
CEBPA1	#######	0.379163	0.365	0.101	#######	CD16_Mor	CEBPA
FAM126A	#######	0.359956	0.286	0.067	#######	CD16_Mor	FAM126A
TCIRG11	#######	0.482269	0.752	0.321	#######	CD16_Mor	TCIRG1

ODF3B1	#######	0.414836	0.535	0.186	#######	CD16_Mor	ODF3B
RFTN1	#######	0.40541	0.355	0.096	#######	CD16_Mor	RFTN1
SYTL1	#######	0.503795	0.466	0.151	#######	CD16_Mor	SYTL1
PFKL	#######	0.4469	0.512	0.178	#######	CD16_Mor	PFKL
ACP2	#######	0.316726	0.225	0.044	#######	CD16_Mor	ACP2
UCP21	#######	0.680164	0.909	0.531	#######	CD16_Mor	UCP2
IGSF61	#######	0.544477	0.813	0.356	#######	CD16_Mor	IGSF6
C11orf21	#######	0.377192	0.367	0.103	#######	CD16_Mor	C11orf21
RP11-312(	#######	0.343403	0.345	0.093	#######	CD16_Mor	RP11-312O7.2
MTHFD2	#######	0.387367	0.43	0.135	#######	CD16_Mor	MTHFD2
PSME2	#######	0.711116	0.889	0.504	#######	CD16_Mor	PSME2
CAMK1D1	#######	0.410763	0.371	0.106	#######	CD16_Mor	CAMK1D
TMEM176I	#######	0.533046	0.673	0.265	#######	CD16_Mor	TMEM176B
ATP2B11	#######	0.525706	0.594	0.23	#######	CD16_Mor	ATP2B1
FBP11	#######	0.561996	0.396	0.122	#######	CD16_Mor	FBP1
FGL21	#######	0.560031	0.918	0.445	#######	CD16_Mor	FGL2
PTP4A2	#######	0.571927	0.932	0.59	#######	CD16_Mor	PTP4A2
TYROBP1	#######	0.655843	0.997	0.696	#######	CD16_Mor	TYROBP
EML41	#######	0.486421	0.562	0.208	#######	CD16_Mor	EML4
SLC8A11	#######	0.372726	0.48	0.16	#######	CD16_Mor	SLC8A1
THEMIS21	#######	0.469858	0.682	0.28	#######	CD16_Mor	THEMIS2
C1orf1621	#######	0.621465	0.868	0.448	#######	CD16_Mor	C1orf162
H2AFZ	#######	0.602222	0.848	0.459	#######	CD16_Mor	H2AFZ
TRIM14	#######	0.410106	0.385	0.114	#######	CD16_Mor	TRIM14
ARRB21	#######	0.579636	0.84	0.431	#######	CD16_Mor	ARRB2
PDK4	#######	0.345924	0.242	0.052	#######	CD16_Mor	PDK4
NACA1	#######	0.625426	0.994	0.812	#######	CD16_Mor	NACA
ANXA51	#######	0.60754	0.947	0.546	#######	CD16_Mor	ANXA5
GBP4	#######	0.455017	0.405	0.124	#######	CD16_Mor	GBP4
DPEP21	#######	0.388324	0.425	0.133	#######	CD16_Mor	DPEP2
OAZ21	#######	0.362903	0.439	0.14	#######	CD16_Mor	OAZ2
TNFSF101	#######	0.643577	0.787	0.389	#######	CD16_Mor	TNFSF10
ALDH3B11	#######	0.337462	0.377	0.11	#######	CD16_Mor	ALDH3B1
PPM1F1	#######	0.359895	0.384	0.114	#######	CD16_Mor	PPM1F
CD300E1	#######	0.403908	0.592	0.221	#######	CD16_Mor	CD300E
KLF21	#######	0.624492	0.915	0.509	#######	CD16_Mor	KLF2
ZNF2171	#######	0.461902	0.487	0.171	#######	CD16_Mor	ZNF217
PTGER4	#######	0.418267	0.384	0.115	#######	CD16_Mor	PTGER4
CORO1B	#######	0.509714	0.541	0.205	#######	CD16_Mor	CORO1B
EPN1	#######	0.395696	0.501	0.175	#######	CD16_Mor	EPN1
TAF10	#######	0.544187	0.839	0.446	#######	CD16_Mor	TAF10
HRH21	#######	0.367581	0.523	0.185	#######	CD16_Mor	HRH2
MICAL1	#######	0.362547	0.364	0.107	#######	CD16_Mor	MICAL1
M6PR	#######	0.553983	0.716	0.328	#######	CD16_Mor	M6PR
FKBP1A	#######	0.466383	0.918	0.549	#######	CD16_Mor	FKBP1A
C10orf541	#######	0.599402	0.888	0.496	#######	CD16_Mor	C10orf54
ANXA21	#######	0.63045	0.904	0.495	#######	CD16_Mor	ANXA2
LIMD1	#######	0.288581	0.207	0.041	#######	CD16_Mor	LIMD1
HMGN21	#######	0.571659	0.873	0.509	#######	CD16_Mor	HMGN2
UBE2J1	#######	0.35896	0.597	0.235	#######	CD16_Mor	UBE2J1
TNNI2	#######	0.326317	0.22	0.046	#######	CD16_Mor	TNNI2
PTPRC	#######	0.580604	0.974	0.742	#######	CD16_Mor	PTPRC
PLXNC11	#######	0.310828	0.463	0.156	#######	CD16_Mor	PLXNC1
PAK11	#######	0.366924	0.756	0.32	#######	CD16_Mor	PAK1
MRPS35	#######	0.419235	0.437	0.147	#######	CD16_Mor	MRPS35
PQLC3	#######	0.425368	0.478	0.167	#######	CD16_Mor	PQLC3
CACUL1	#######	0.339383	0.392	0.121	#######	CD16_Mor	CACUL1
SEC11A1	#######	0.557042	0.825	0.418	#######	CD16_Mor	SEC11A

HHEX2	#######	0.339296	0.419	0.135	#######	CD16_Mor	HHEX
ADK1	#######	0.426992	0.357	0.107	#######	CD16_Mor	ADK
GNB21	#######	0.533351	0.92	0.548	#######	CD16_Mor	GNB2
SSBP4	#######	0.44129	0.495	0.178	#######	CD16_Mor	SSBP4
SKIL	#######	0.284983	0.242	0.055	#######	CD16_Mor	SKIL
MIDN1	#######	0.389512	0.47	0.163	#######	CD16_Mor	MIDN
ADRBK11	#######	0.371522	0.689	0.292	#######	CD16_Mor	ADRBK1
POFUT1	#######	0.263354	0.189	0.036	#######	CD16_Mor	POFUT1
KYNU1	#######	0.342205	0.329	0.094	#######	CD16_Mor	KYNU
SNX23	#######	0.434756	0.649	0.273	#######	CD16_Mor	SNX2
ATP5B	#######	0.595318	0.846	0.47	#######	CD16_Mor	ATP5B
NFATC1	#######	0.298885	0.212	0.044	#######	CD16_Mor	NFATC1
RASSF5	#######	0.408837	0.537	0.202	#######	CD16_Mor	RASSF5
ABHD6	#######	0.251668	0.169	0.029	#######	CD16_Mor	ABHD6
PABPC4	#######	0.463947	0.505	0.188	#######	CD16_Mor	PABPC4
CMTM71	#######	0.403041	0.571	0.224	#######	CD16_Mor	CMTM7
MIS18BP1	#######	0.414951	0.659	0.279	#######	CD16_Mor	MIS18BP1
RNPEP1	#######	0.408841	0.504	0.187	#######	CD16_Mor	RNPEP
PRDX4	#######	0.364346	0.331	0.095	#######	CD16_Mor	PRDX4
PDCL3	#######	0.388758	0.335	0.098	#######	CD16_Mor	PDCL3
NOTCH21	#######	0.390338	0.656	0.279	#######	CD16_Mor	NOTCH2
RAP1GAP2	#######	0.316994	0.292	0.077	#######	CD16_Mor	RAP1GAP2
PSMA41	#######	0.498819	0.67	0.297	#######	CD16_Mor	PSMA4
SRD5A3	#######	0.292136	0.218	0.047	#######	CD16_Mor	SRD5A3
STK10	#######	0.461505	0.634	0.27	#######	CD16_Mor	STK10
MSN	#######	0.547259	0.895	0.54	#######	CD16_Mor	MSN
FAM89B	#######	0.358649	0.424	0.143	#######	CD16_Mor	FAM89B
ITPK11	#######	0.368898	0.376	0.118	#######	CD16_Mor	ITPK1
AP001053.	#######	0.281632	0.201	0.041	#######	CD16_Mor	AP001053.11
LIPA	#######	0.388667	0.469	0.169	#######	CD16_Mor	LIPA
RIN31	#######	0.391676	0.593	0.239	#######	CD16_Mor	RIN3
HSPA1A1	#######	0.441431	0.571	0.23	#######	CD16_Mor	HSPA1A
AGPAT31	#######	0.343363	0.357	0.109	#######	CD16_Mor	AGPAT3
PLIN2	#######	0.390624	0.423	0.142	#######	CD16_Mor	PLIN2
VAMP51	#######	0.615004	0.633	0.284	#######	CD16_Mor	VAMP5
NOTCH2N	#######	0.307124	0.321	0.092	#######	CD16_Mor	NOTCH2NL
LCP2	#######	0.479761	0.66	0.296	#######	CD16_Mor	LCP2
EHBP1L11	#######	0.362196	0.596	0.238	#######	CD16_Mor	EHBP1L1
OLIG1	#######	0.298813	0.22	0.049	#######	CD16_Mor	OLIG1
DOCK21	#######	0.42427	0.65	0.28	#######	CD16_Mor	DOCK2
CAT1	#######	0.483812	0.621	0.268	#######	CD16_Mor	CAT
PSMB9	#######	0.570789	0.896	0.53	#######	CD16_Mor	PSMB9
ALOX52	#######	0.272652	0.528	0.199	#######	CD16_Mor	ALOX5
SFT2D2	#######	0.381085	0.46	0.166	#######	CD16_Mor	SFT2D2
KIAA10332	#######	0.376397	0.538	0.209	#######	CD16_Mor	KIAA1033
HCLS11	#######	0.535681	0.915	0.566	#######	CD16_Mor	HCLS1
HLA-DQB1	#######	0.447682	0.744	0.339	#######	CD16_Mor	HLA-DQB1
MAP3K12	#######	0.369784	0.559	0.221	#######	CD16_Mor	MAP3K1
SH3BP1	#######	0.396131	0.505	0.191	#######	CD16_Mor	SH3BP1
SELPLG	#######	0.447639	0.782	0.398	#######	CD16_Mor	SELPLG
SOD11	#######	0.601239	0.741	0.374	#######	CD16_Mor	SOD1
MT2A1	#######	0.733492	0.802	0.429	#######	CD16_Mor	MT2A
GNG2	#######	0.419196	0.562	0.228	#######	CD16_Mor	GNG2
CARD91	#######	0.306669	0.294	0.082	#######	CD16_Mor	CARD9
CHMP4B1	#######	0.422054	0.745	0.356	#######	CD16_Mor	CHMP4B
TTYH3	#######	0.289115	0.284	0.078	#######	CD16_Mor	TTYH3
SSH21	#######	0.400371	0.607	0.258	#######	CD16_Mor	SSH2
TMC6	#######	0.391072	0.427	0.148	#######	CD16_Mor	TMC6

IRF51	#######	0.32206	0.398	0.133	#######	CD16_Mor	IRF5
CYFIP21	#######	0.366822	0.446	0.156	#######	CD16_Mor	CYFIP2
CCDC1151	#######	0.383399	0.372	0.122	#######	CD16_Mor	CCDC115
GNG51	#######	0.480233	0.94	0.596	#######	CD16_Mor	GNG5
FAM45A1	#######	0.272465	0.664	0.283	#######	CD16_Mor	FAM45A
SNX51	#######	0.407071	0.517	0.202	#######	CD16_Mor	SNX5
ADRBK21	#######	0.293149	0.411	0.139	#######	CD16_Mor	ADRBK2
CHN21	#######	0.271487	0.322	0.095	#######	CD16_Mor	CHN2
GBP1	#######	0.494895	0.423	0.15	#######	CD16_Mor	GBP1
HIGD2A1	#######	0.542698	0.926	0.585	#######	CD16_Mor	HIGD2A
MTHFS	#######	0.381142	0.413	0.143	#######	CD16_Mor	MTHFS
LIMD21	#######	0.575041	0.917	0.573	#######	CD16_Mor	LIMD2
SCARB21	#######	0.326327	0.369	0.119	#######	CD16_Mor	SCARB2
KLF11	#######	0.278667	0.194	0.041	#######	CD16_Mor	KLF11
TBCB	#######	0.431898	0.673	0.307	#######	CD16_Mor	TBCB
GUSB	#######	0.353394	0.414	0.145	#######	CD16_Mor	GUSB
ALDH16A1	#######	0.304405	0.277	0.076	#######	CD16_Mor	ALDH16A1
GNB41	#######	0.281124	0.313	0.093	#######	CD16_Mor	GNB4
ARHGAP2	#######	0.319743	0.446	0.16	#######	CD16_Mor	ARHGAP27
PGLS1	#######	0.444102	0.88	0.484	#######	CD16_Mor	PGLS
RP3-395M	#######	0.261491	0.19	0.04	#######	CD16_Mor	RP3-395M20.12
S100Z1	#######	0.281232	0.311	0.092	#######	CD16_Mor	S100Z
NANS1	#######	0.34238	0.373	0.125	#######	CD16_Mor	NANS
TLR11	#######	0.293012	0.308	0.091	#######	CD16_Mor	TLR1
ST3GAL5	#######	0.280116	0.268	0.072	#######	CD16_Mor	ST3GAL5
DOCK81	#######	0.444649	0.777	0.4	#######	CD16_Mor	DOCK8
SVIL	#######	0.273282	0.29	0.082	#######	CD16_Mor	SVIL
CLEC2B	#######	0.396136	0.574	0.239	#######	CD16_Mor	CLEC2B
SNX101	#######	0.336662	0.707	0.321	#######	CD16_Mor	SNX10
IMPDH11	#######	0.30902	0.506	0.196	#######	CD16_Mor	IMPDH1
PRDX1	#######	0.541043	0.744	0.378	#######	CD16_Mor	PRDX1
PPP1CA	#######	0.497189	0.873	0.525	#######	CD16_Mor	PPP1CA
EMP3	#######	0.63344	0.973	0.734	#######	CD16_Mor	EMP3
LAPTM4A	#######	0.413784	0.61	0.269	#######	CD16_Mor	LAPTM4A
CYTH41	#######	0.274961	0.542	0.217	#######	CD16_Mor	CYTH4
ERGIC1	#######	0.297984	0.388	0.132	#######	CD16_Mor	ERGIC1
MT-CO11	#######	0.386498	0.997	0.985	#######	CD16_Mor	MT-CO1
ATP1A1	#######	0.407562	0.556	0.233	#######	CD16_Mor	ATP1A1
CCDC12	#######	0.392603	0.576	0.245	#######	CD16_Mor	CCDC12
SLIRP	#######	0.425781	0.605	0.263	#######	CD16_Mor	SLIRP
MFSD12	#######	0.274809	0.237	0.061	#######	CD16_Mor	MFSD12
PIEZO1	#######	0.298537	0.3	0.089	#######	CD16_Mor	PIEZO1
ACOT9	#######	0.294218	0.324	0.102	#######	CD16_Mor	ACOT9
FCGRT1	#######	0.397814	0.778	0.365	#######	CD16_Mor	FCGRT
CBL1	#######	0.284459	0.446	0.165	#######	CD16_Mor	CBL
LGALS31	#######	0.473915	0.88	0.457	#######	CD16_Mor	LGALS3
NCKAP1L1	#######	0.359218	0.584	0.247	#######	CD16_Mor	NCKAP1L
PCK2	#######	0.264106	0.214	0.052	#######	CD16_Mor	PCK2
CTNND1	#######	0.281691	0.225	0.057	#######	CD16_Mor	CTNND1
GNS1	#######	0.299182	0.565	0.232	#######	CD16_Mor	GNS
UQCRB	#######	0.544641	0.957	0.668	#######	CD16_Mor	UQCRB
LRRFIP11	#######	0.474036	0.935	0.609	#######	CD16_Mor	LRRFIP1
TMBIM4.1	#######	0.397447	0.721	0.355	#######	CD16_Mor	TMBIM4.1
CRIP1	#######	0.746683	0.836	0.512	#######	CD16_Mor	CRIP1
CTSS1	#######	0.567541	0.992	0.688	#######	CD16_Mor	CTSS
CYC1	#######	0.397543	0.54	0.224	#######	CD16_Mor	CYC1
SERPINB9	#######	0.355479	0.386	0.135	#######	CD16_Mor	SERPINB9
ITSN21	#######	0.38519	0.576	0.248	#######	CD16_Mor	ITSN2

DNAJA1	#######	0.415761	0.705	0.345	#######	CD16_Mor	DNAJA1
SORT11	#######	0.250625	0.342	0.112	#######	CD16_Mor	SORT1
CAPNS11	#######	0.346069	0.723	0.344	#######	CD16_Mor	CAPNS1
REPS1	#######	0.253422	0.238	0.062	#######	CD16_Mor	REPS1
PYCARD1	#######	0.424546	0.957	0.575	#######	CD16_Mor	PYCARD
ARL6IP41	#######	0.482306	0.864	0.508	#######	CD16_Mor	ARL6IP4
SH3BGRL	#######	0.35784	0.867	0.512	#######	CD16_Mor	SH3BGRL
GNAI21	#######	0.468208	0.954	0.664	#######	CD16_Mor	GNAI2
CD48	#######	0.529857	0.892	0.559	#######	CD16_Mor	CD48
COX7B	#######	0.449094	0.812	0.44	#######	CD16_Mor	COX7B
OXR1	#######	0.30953	0.354	0.119	#######	CD16_Mor	OXR1
AAMP	#######	0.335202	0.432	0.162	#######	CD16_Mor	AAMP
SNAP29	#######	0.27008	0.407	0.148	#######	CD16_Mor	SNAP29
SOX41	#######	0.334813	0.412	0.153	#######	CD16_Mor	SOX4
YIF1A	#######	0.337776	0.351	0.12	#######	CD16_Mor	YIF1A
HVCN11	#######	0.25913	0.428	0.159	#######	CD16_Mor	HVCN1
DEDD2	#######	0.281105	0.333	0.109	#######	CD16_Mor	DEDD2
LTA4H1	#######	0.477515	0.807	0.431	#######	CD16_Mor	LTA4H
PLCB21	#######	0.269854	0.481	0.189	#######	CD16_Mor	PLCB2
IDH2	#######	0.365707	0.482	0.195	#######	CD16_Mor	IDH2
DDX211	#######	0.379306	0.723	0.358	#######	CD16_Mor	DDX21
MINOS11	#######	0.417335	0.796	0.423	#######	CD16_Mor	MINOS1
SP1101	#######	0.373102	0.647	0.302	#######	CD16_Mor	SP110
SH3TC11	#######	0.261864	0.287	0.088	#######	CD16_Mor	SH3TC1
ARPC1A	#######	0.317133	0.445	0.172	#######	CD16_Mor	ARPC1A
TPI11	#######	0.455218	0.935	0.607	#######	CD16_Mor	TPI1
HSP90AA1	#######	0.55945	0.916	0.601	#######	CD16_Mor	HSP90AA1
ENY21	#######	0.329754	0.732	0.357	#######	CD16_Mor	ENY2
RHOA	#######	0.388065	0.97	0.751	#######	CD16_Mor	RHOA
STK32C	#######	0.295307	0.248	0.07	#######	CD16_Mor	STK32C
UQCRC11	#######	0.356953	0.683	0.323	#######	CD16_Mor	UQCRC1
ATP5G3	#######	0.486848	0.843	0.475	#######	CD16_Mor	ATP5G3
PRKACA1	#######	0.261377	0.393	0.143	#######	CD16_Mor	PRKACA
PDCD6IP	#######	0.338494	0.535	0.228	#######	CD16_Mor	PDCD6IP
PITPNM1	#######	0.29626	0.235	0.064	#######	CD16_Mor	PITPNM1
SDHB	#######	0.332713	0.467	0.188	#######	CD16_Mor	SDHB
NDUFS7	#######	0.410003	0.679	0.324	#######	CD16_Mor	NDUFS7
HSPA81	#######	0.471226	0.922	0.559	#######	CD16_Mor	HSPA8
IFNAR2	#######	0.325711	0.452	0.179	#######	CD16_Mor	IFNAR2
NRROS	#######	0.302209	0.428	0.163	#######	CD16_Mor	NRROS
CD371	#######	0.449825	0.951	0.683	#######	CD16_Mor	CD37
VAV1	#######	0.286728	0.369	0.13	#######	CD16_Mor	VAV1
REL2	#######	0.350294	0.668	0.32	#######	CD16_Mor	REL
BTG21	#######	0.311504	0.513	0.214	#######	CD16_Mor	BTG2
MVP	#######	0.326077	0.566	0.248	#######	CD16_Mor	MVP
IVD	#######	0.319368	0.278	0.085	#######	CD16_Mor	IVD
NDUFB5	#######	0.365934	0.581	0.257	#######	CD16_Mor	NDUFB5
ARHGAP1	#######	0.285044	0.334	0.113	#######	CD16_Mor	ARHGAP1
GPI	#######	0.301396	0.552	0.242	#######	CD16_Mor	GPI
BST2	#######	0.437518	0.768	0.402	#######	CD16_Mor	BST2
CTB-133G	#######	0.317595	0.397	0.145	#######	CD16_Mor	CTB-133G6.1
CBWD2	#######	0.309597	0.374	0.136	#######	CD16_Mor	CBWD2
CYBA1	#######	0.494548	0.983	0.805	#######	CD16_Mor	CYBA
TMEM179I	#######	0.32963	0.581	0.258	#######	CD16_Mor	TMEM179B
OGDH	#######	0.26508	0.344	0.12	#######	CD16_Mor	OGDH
PSMB8	#######	0.424422	0.808	0.445	#######	CD16_Mor	PSMB8
HAGH	#######	0.300701	0.376	0.136	#######	CD16_Mor	HAGH
LNPEP	#######	0.33608	0.525	0.225	#######	CD16_Mor	LNPEP

NECAP2	#######	0.329108	0.44	0.175	#######	CD16_Mor	NECAP2
CNDP2	#######	0.302125	0.432	0.168	#######	CD16_Mor	CNDP2
CORO1A	#######	0.48961	0.96	0.72	#######	CD16_Mor	CORO1A
TPMT	#######	0.290377	0.255	0.076	#######	CD16_Mor	TPMT
KIAA0141	#######	0.262538	0.309	0.102	#######	CD16_Mor	KIAA0141
GIMAP81	#######	0.283662	0.494	0.203	#######	CD16_Mor	GIMAP8
TBC1D1	#######	0.291197	0.405	0.154	#######	CD16_Mor	TBC1D1
UBAC11	#######	0.265305	0.377	0.138	#######	CD16_Mor	UBAC1
COX5B1	#######	0.431904	0.944	0.628	#######	CD16_Mor	COX5B
RILPL21	#######	0.257305	0.617	0.279	#######	CD16_Mor	RILPL2
FAM46A1	#######	0.259945	0.342	0.119	#######	CD16_Mor	FAM46A
PSMC4	#######	0.353363	0.42	0.164	#######	CD16_Mor	PSMC4
AP1M1	#######	0.296938	0.402	0.152	#######	CD16_Mor	AP1M1
ZFP361	#######	0.435511	0.754	0.405	#######	CD16_Mor	ZFP36
ERICH11	#######	0.256027	0.601	0.273	#######	CD16_Mor	ERICH1
PDCD5	#######	0.361698	0.526	0.227	#######	CD16_Mor	PDCD5
CTBP21	#######	0.264787	0.375	0.137	#######	CD16_Mor	CTBP2
C14orf159	#######	0.284605	0.313	0.105	#######	CD16_Mor	C14orf159
GSTK1	#######	0.441374	0.855	0.494	#######	CD16_Mor	GSTK1
ACTR3	#######	0.437263	0.858	0.517	#######	CD16_Mor	ACTR3
ARRDC11	#######	0.272849	0.466	0.191	#######	CD16_Mor	ARRDC1
DOK2	#######	0.338515	0.689	0.345	#######	CD16_Mor	DOK2
SNRPC	#######	0.335231	0.559	0.25	#######	CD16_Mor	SNRPC
LSM6	#######	0.30436	0.601	0.281	#######	CD16_Mor	LSM6
TRANK1	#######	0.261567	0.304	0.1	#######	CD16_Mor	TRANK1
CD41	#######	0.261252	0.601	0.27	#######	CD16_Mor	CD4
NME1-NM	#######	0.454033	0.896	0.549	#######	CD16_Mor	NME1-NME2
CYCS	#######	0.30776	0.492	0.209	#######	CD16_Mor	CYCS
FNIP2	#######	0.292081	0.193	0.05	#######	CD16_Mor	FNIP2
TBC1D22A	#######	0.25553	0.323	0.112	#######	CD16_Mor	TBC1D22A
TSC22D3	#######	0.284909	0.795	0.445	#######	CD16_Mor	TSC22D3
HM131	#######	0.339432	0.643	0.312	#######	CD16_Mor	HM13
EIF4E21	#######	0.280401	0.576	0.261	#######	CD16_Mor	EIF4E2
SSNA1	#######	0.326851	0.58	0.267	#######	CD16_Mor	SSNA1
UBE2L6	#######	0.375742	0.629	0.305	#######	CD16_Mor	UBE2L6
TREX1	#######	0.298095	0.408	0.161	#######	CD16_Mor	TREX1
MLX	#######	0.294086	0.467	0.195	#######	CD16_Mor	MLX
SUPT4H1	#######	0.252412	0.653	0.319	#######	CD16_Mor	SUPT4H1
SLC25A3	#######	0.455688	0.883	0.546	#######	CD16_Mor	SLC25A3
CAP1	#######	0.309889	0.929	0.628	#######	CD16_Mor	CAP1
CD551	#######	0.262784	0.629	0.303	#######	CD16_Mor	CD55
AGA	#######	0.252659	0.23	0.067	#######	CD16_Mor	AGA
NBPF101	#######	0.267923	0.543	0.24	#######	CD16_Mor	NBPF10
CBR1	#######	0.288251	0.408	0.161	#######	CD16_Mor	CBR1
CERK	#######	0.275027	0.341	0.124	#######	CD16_Mor	CERK
IRF11	#######	0.416617	0.808	0.462	#######	CD16_Mor	IRF1
PARVG1	#######	0.250544	0.664	0.32	#######	CD16_Mor	PARVG
RFK	#######	0.252965	0.241	0.073	#######	CD16_Mor	RFK
RUNX3	#######	0.25255	0.295	0.098	#######	CD16_Mor	RUNX3
FAM195A	#######	0.254461	0.257	0.082	#######	CD16_Mor	FAM195A
MIEN1	#######	0.278591	0.591	0.275	#######	CD16_Mor	MIEN1
MAN2B11	#######	0.276301	0.503	0.218	#######	CD16_Mor	MAN2B1
DECR1	#######	0.325535	0.519	0.232	#######	CD16_Mor	DECR1
CNPPD11	#######	0.272704	0.428	0.175	#######	CD16_Mor	CNPPD1
SNRPB	#######	0.34582	0.63	0.31	#######	CD16_Mor	SNRPB
VDAC1	#######	0.332872	0.549	0.251	#######	CD16_Mor	VDAC1
DNPH11	#######	0.342487	0.327	0.12	#######	CD16_Mor	DNPH1
NBN	#######	0.265999	0.376	0.145	#######	CD16_Mor	NBN

EEF1A11	#######	0.310242	0.998	0.886	#######	CD16_Mor	EEF1A1
TNFRSF14.	#######	0.324469	0.571	0.267	#######	CD16_Mor	TNFRSF14
TMA16	#######	0.302233	0.306	0.108	#######	CD16_Mor	TMA16
ERP44	#######	0.272245	0.449	0.188	#######	CD16_Mor	ERP44
ARHGAP4:	#######	0.274914	0.571	0.268	#######	CD16_Mor	ARHGAP4
CD47	#######	0.284019	0.608	0.295	#######	CD16_Mor	CD47
NDUFA13	#######	0.375604	0.809	0.456	#######	CD16_Mor	NDUFA13
CLTB	#######	0.259166	0.582	0.27	#######	CD16_Mor	CLTB
TAOK3	#######	0.259966	0.704	0.357	#######	CD16_Mor	TAOK3
EIF6	#######	0.293435	0.499	0.219	#######	CD16_Mor	EIF6
REEP51	#######	0.282595	0.725	0.372	#######	CD16_Mor	REEP5
NAGK1	#######	0.264324	0.679	0.331	#######	CD16_Mor	NAGK
UCHL3	#######	0.266999	0.306	0.109	#######	CD16_Mor	UCHL3
POMP1	#######	0.297804	0.757	0.406	#######	CD16_Mor	POMP
PGAM11	#######	0.327026	0.806	0.451	#######	CD16_Mor	PGAM1
MDH2	#######	0.337635	0.676	0.336	#######	CD16_Mor	MDH2
RBM3	#######	0.428166	0.789	0.45	#######	CD16_Mor	RBM3
IKZF1	#######	0.377569	0.64	0.327	#######	CD16_Mor	IKZF1
LYSMD2	#######	0.275996	0.451	0.191	#######	CD16_Mor	LYSMD2
SSR3	#######	0.299603	0.628	0.305	#######	CD16_Mor	SSR3
SRI	#######	0.345501	0.565	0.268	#######	CD16_Mor	SRI
GBP21	#######	0.301766	0.535	0.245	#######	CD16_Mor	GBP2
RPL81	#######	0.398022	0.997	0.851	#######	CD16_Mor	RPL8
BANF1	#######	0.322261	0.635	0.314	#######	CD16_Mor	BANF1
ZEB21	#######	0.254714	0.775	0.403	#######	CD16_Mor	ZEB2
PSENEN	#######	0.273933	0.438	0.185	#######	CD16_Mor	PSENEN
COX5A1	#######	0.36452	0.832	0.47	#######	CD16_Mor	COX5A
PSMB10	#######	0.389037	0.814	0.473	#######	CD16_Mor	PSMB10
SIVA1	#######	0.292008	0.54	0.249	#######	CD16_Mor	SIVA1
BAX	#######	0.319282	0.687	0.356	#######	CD16_Mor	BAX
ERCC1	#######	0.260998	0.394	0.16	#######	CD16_Mor	ERCC1
KRAS	#######	0.281741	0.515	0.235	#######	CD16_Mor	KRAS
LSP11	#######	0.426971	0.918	0.657	#######	CD16_Mor	LSP1
NDUFS6	#######	0.313959	0.651	0.325	#######	CD16_Mor	NDUFS6
UQCRC2	#######	0.294084	0.495	0.221	#######	CD16_Mor	UQCRC2
IRF2	#######	0.252767	0.536	0.248	#######	CD16_Mor	IRF2
ADCY7	#######	0.258897	0.368	0.146	#######	CD16_Mor	ADCY7
ELOVL1	#######	0.272662	0.392	0.16	#######	CD16_Mor	ELOVL1
ARPC51	#######	0.28701	0.95	0.682	#######	CD16_Mor	ARPC5
NBPF141	#######	0.251546	0.272	0.094	#######	CD16_Mor	NBPF14
FAM162A	#######	0.308596	0.34	0.131	#######	CD16_Mor	FAM162A
S1PR4	#######	0.294852	0.549	0.265	#######	CD16_Mor	S1PR4
HSPB1	#######	0.28589	0.463	0.205	#######	CD16_Mor	HSPB1
UQCC2	#######	0.29194	0.344	0.134	#######	CD16_Mor	UQCC2
CDK2AP2	#######	0.261977	0.562	0.265	#######	CD16_Mor	CDK2AP2
NKTR	#######	0.27904	0.499	0.228	#######	CD16_Mor	NKTR
MRPL13	#######	0.260511	0.307	0.113	#######	CD16_Mor	MRPL13
LGALS11	#######	0.360852	0.963	0.604	#######	CD16_Mor	LGALS1
AHCY	#######	0.255494	0.241	0.079	#######	CD16_Mor	AHCY
UBXN12	#######	0.366464	0.778	0.444	#######	CD16_Mor	UBXN1
GPR65	#######	0.260046	0.498	0.226	#######	CD16_Mor	GPR65
COX6C	#######	0.39672	0.894	0.564	#######	CD16_Mor	COX6C
CIRBP1	#######	0.322484	0.769	0.431	#######	CD16_Mor	CIRBP
ISG15	#######	0.266721	0.416	0.177	#######	CD16_Mor	ISG15
VDAC2	#######	0.298999	0.581	0.284	#######	CD16_Mor	VDAC2
LSM4	#######	0.312989	0.496	0.228	#######	CD16_Mor	LSM4
MT-CO21	#######	0.297268	0.997	0.975	#######	CD16_Mor	MT-CO2
NDUFA12	#######	0.310267	0.679	0.35	#######	CD16_Mor	NDUFA12

LAMTOR4	#######	0.275933	0.946	0.636	#######	CD16_Mor	LAMTOR4
FAM96A2	#######	0.266317	0.557	0.265	#######	CD16_Mor	FAM96A
HNRNPDL	#######	0.329746	0.779	0.443	#######	CD16_Mor	HNRNPDL
TUBB1	#######	0.264244	0.69	0.365	#######	CD16_Mor	TUBB
CACYBP	#######	0.286883	0.457	0.205	#######	CD16_Mor	CACYBP
C19orf70	#######	0.295336	0.59	0.292	#######	CD16_Mor	C19orf70
SNX17	#######	0.298399	0.594	0.299	#######	CD16_Mor	SNX17
WAS1	#######	0.265418	0.747	0.411	#######	CD16_Mor	WAS
CHCHD2	#######	0.362429	0.91	0.608	#######	CD16_Mor	CHCHD2
SNHG81	#######	0.305982	0.568	0.28	#######	CD16_Mor	SNHG8
RPS27L	#######	0.312555	0.661	0.348	#######	CD16_Mor	RPS27L
PSMD8	#######	0.273949	0.592	0.295	#######	CD16_Mor	PSMD8
ACTR21	#######	0.312758	0.868	0.545	#######	CD16_Mor	ACTR2
C9orf78	#######	0.284682	0.555	0.275	#######	CD16_Mor	C9orf78
SDHD1	#######	0.267156	0.435	0.193	#######	CD16_Mor	SDHD
TES	#######	0.261016	0.546	0.266	#######	CD16_Mor	TES
OTUB1	#######	0.262552	0.609	0.307	#######	CD16_Mor	OTUB1
HLA-DMA	#######	0.269637	0.594	0.287	#######	CD16_Mor	HLA-DMA
ATP5C1	#######	0.287307	0.667	0.351	#######	CD16_Mor	ATP5C1
NDUFA4	#######	0.303859	0.837	0.521	#######	CD16_Mor	NDUFA4
NDUFB2	#######	0.320607	0.866	0.528	#######	CD16_Mor	NDUFB2
PSME1	#######	0.358711	0.951	0.677	#######	CD16_Mor	PSME1
CIB1	#######	0.252561	0.66	0.349	#######	CD16_Mor	CIB1
ATP5O1	#######	0.323101	0.812	0.473	#######	CD16_Mor	ATP5O
IL10RA	#######	0.267589	0.613	0.317	#######	CD16_Mor	IL10RA
S100A41	#######	0.406192	1	0.863	#######	CD16_Mor	S100A4
LDHA	#######	0.263299	0.694	0.373	#######	CD16_Mor	LDHA
MYL12B	#######	0.32981	0.91	0.625	#######	CD16_Mor	MYL12B
SNRPG	#######	0.269914	0.734	0.407	#######	CD16_Mor	SNRPG
ARHGEF1	#######	0.268285	0.589	0.3	#######	CD16_Mor	ARHGEF1
CCT6A	#######	0.286969	0.564	0.284	#######	CD16_Mor	CCT6A
PEA15	#######	0.256022	0.326	0.134	#######	CD16_Mor	PEA15
LCP11	#######	0.330271	0.936	0.663	#######	CD16_Mor	LCP1
SAP18	#######	0.287803	0.718	0.409	#######	CD16_Mor	SAP18
COX6A1	#######	0.309012	0.894	0.598	#######	CD16_Mor	COX6A1
MT-ND51	#######	0.382076	0.977	0.783	#######	CD16_Mor	MT-ND5
COX7A2	#######	0.277952	0.87	0.543	#######	CD16_Mor	COX7A2
EIF1	#######	0.272869	0.988	0.873	#######	CD16_Mor	EIF1
C11orf311	#######	0.28667	0.779	0.459	#######	CD16_Mor	C11orf31
EIF4A11	#######	0.320668	0.914	0.615	#######	CD16_Mor	EIF4A1
COX6B11	#######	0.287798	0.961	0.713	#######	CD16_Mor	COX6B1
DDX5	#######	0.331566	0.891	0.591	#######	CD16_Mor	DDX5
HSP90AB1	#######	0.350141	0.844	0.537	#######	CD16_Mor	HSP90AB1
SRSF51	#######	0.28582	0.762	0.449	#######	CD16_Mor	SRSF5
UBC	#######	0.317639	0.951	0.749	#######	CD16_Mor	UBC
ICAM3	#######	0.274273	0.663	0.389	4.05E-99	CD16_Mor	ICAM3
EZR1	#######	0.276385	0.523	0.278	4.53E-99	CD16_Mor	EZR
MT-ATP81	1.25E-97	0.376418	0.933	0.727	4.08E-93	CD16_Mor	MT-ATP8
MT-ND31	3.29E-97	0.280925	0.993	0.902	1.08E-92	CD16_Mor	MT-ND3
GSTP11	1.73E-96	0.270537	0.957	0.66	5.68E-92	CD16_Mor	GSTP1
TXNIP1	3.17E-96	0.347624	0.957	0.736	1.04E-91	CD16_Mor	TXNIP
SUMO2	1.07E-92	0.256549	0.887	0.595	3.50E-88	CD16_Mor	SUMO2
YWHAB1	2.16E-90	0.264543	0.962	0.737	7.07E-86	CD16_Mor	YWHAB
PTMA	3.34E-84	0.256252	0.999	0.9	1.09E-79	CD16_Mor	PTMA
FCN11	3.75E-84	0.26778	0.905	0.54	1.23E-79	CD16_Mor	FCN1
CCNI1	4.48E-83	0.276422	0.926	0.681	1.47E-78	CD16_Mor	CCNI
PFDN51	6.58E-83	0.291377	0.978	0.796	2.15E-78	CD16_Mor	PFDN5
PPIA1	3.47E-80	0.279473	0.964	0.721	1.13E-75	CD16_Mor	PPIA

COX7C1	2.71E-78	0.293859	0.952	0.693	8.86E-74	CD16_Mor	COX7C
RPL36A1	1.81E-75	0.255632	0.913	0.618	5.94E-71	CD16_Mor	RPL36A
TMSB101	5.95E-71	0.271723	1	0.906	1.95E-66	CD16_Mor	TMSB10
RPL261	1.54E-54	0.252202	0.98	0.798	5.05E-50	CD16_Mor	RPL26
MTRNR2L:	1.25E-17	0.343268	0.208	0.14	4.11E-13	CD16_Mor	MTRNR2L12
CCR71	0	2.560262	0.902	0.082	0	CD4_Naive	CCR7
TCF7	0	2.085306	0.835	0.1	0	CD4_Naive	TCF7
LEF1	0	2.045239	0.797	0.073	0	CD4_Naive	LEF1
CD3E	0	2.035583	0.986	0.189	0	CD4_Naive	CD3E
LDHB	0	2.030867	0.988	0.378	0	CD4_Naive	LDHB
LINC00861	0	1.989204	0.891	0.171	0	CD4_Naive	LINC00861
RPS51	0	1.849583	1	0.7	0	CD4_Naive	RPS5
IL7R	0	1.846208	0.89	0.142	0	CD4_Naive	IL7R
C12orf571	0	1.845335	0.941	0.289	0	CD4_Naive	C12orf57
RPS271	0	1.818549	1	0.828	0	CD4_Naive	RPS27
LTB1	0	1.810493	0.97	0.267	0	CD4_Naive	LTB
TMEM661	0	1.741399	0.993	0.541	0	CD4_Naive	TMEM66
CD3D	0	1.719009	0.891	0.155	0	CD4_Naive	CD3D
EEF1B21	0	1.717598	0.999	0.667	0	CD4_Naive	EEF1B2
NOSIP	0	1.697908	0.873	0.298	0	CD4_Naive	NOSIP
RPS251	0	1.658257	1	0.771	0	CD4_Naive	RPS25
RPS121	0	1.63519	1	0.88	0	CD4_Naive	RPS12
NPM11	0	1.613026	0.984	0.516	0	CD4_Naive	NPM1
PRKCQ-AS	0	1.610024	0.713	0.103	0	CD4_Naive	PRKCQ-AS1
RPL33	0	1.589431	1	0.741	0	CD4_Naive	RPL3
EEF1G1	0	1.577618	0.899	0.516	0	CD4_Naive	EEF1G
RPS181	0	1.559491	1	0.785	0	CD4_Naive	RPS18
RPS291	0	1.550111	0.998	0.761	0	CD4_Naive	RPS29
RPS15A1	0	1.546789	1	0.866	0	CD4_Naive	RPS15A
RPL321	0	1.546349	1	0.868	0	CD4_Naive	RPL32
CD3G	0	1.542955	0.815	0.141	0	CD4_Naive	CD3G
RPS31	0	1.533931	1	0.829	0	CD4_Naive	RPS3
RPS61	0	1.523364	1	0.778	0	CD4_Naive	RPS6
RPS3A1	0	1.521836	1	0.855	0	CD4_Naive	RPS3A
RPS231	0	1.51191	1	0.822	0	CD4_Naive	RPS23
RPL51	0	1.502462	1	0.797	0	CD4_Naive	RPL5
RPS27A1	0	1.498979	1	0.865	0	CD4_Naive	RPS27A
EEF1A12	0	1.497308	1	0.89	0	CD4_Naive	EEF1A1
RPL10A1	0	1.488368	0.999	0.682	0	CD4_Naive	RPL10A
RPSA1	0	1.484917	0.999	0.693	0	CD4_Naive	RPSA
MAL	0	1.479138	0.592	0.061	0	CD4_Naive	MAL
CAMK4	0	1.475486	0.617	0.068	0	CD4_Naive	CAMK4
RPL301	0	1.46202	1	0.885	0	CD4_Naive	RPL30
GIMAP5	0	1.451522	0.835	0.263	0	CD4_Naive	GIMAP5
CD27	0	1.44349	0.672	0.089	0	CD4_Naive	CD27
RPS4X1	0	1.440503	1	0.809	0	CD4_Naive	RPS4X
BTG11	0	1.433407	0.998	0.677	0	CD4_Naive	BTG1
RPL101	0	1.393867	1	0.877	0	CD4_Naive	RPL10
RPL141	0	1.392169	1	0.807	0	CD4_Naive	RPL14
RPL131	0	1.386551	1	0.86	0	CD4_Naive	RPL13
RPS211	0	1.377172	1	0.833	0	CD4_Naive	RPS21
PIK3IP1	0	1.375744	0.577	0.099	0	CD4_Naive	PIK3IP1
GIMAP7	0	1.371341	0.978	0.565	0	CD4_Naive	GIMAP7
LBH1	0	1.366149	0.703	0.154	0	CD4_Naive	LBH
GLTSCR21	0	1.346616	0.906	0.448	0	CD4_Naive	GLTSCR2
CD7	0	1.309484	0.862	0.187	0	CD4_Naive	CD7
RPL111	0	1.296981	1	0.881	0	CD4_Naive	RPL11
RPL341	0	1.296248	1	0.872	0	CD4_Naive	RPL34

RPS101	0	1.289295	0.97	0.76	0	CD4_Naive	RPS10
RPL191	0	1.275539	1	0.868	0	CD4_Naive	RPL19
RPS141	0	1.270687	1	0.871	0	CD4_Naive	RPS14
RPL35A1	0	1.267774	1	0.869	0	CD4_Naive	RPL35A
RPL18A1	0	1.26689	1	0.824	0	CD4_Naive	RPL18A
ETS11	0	1.257985	0.675	0.18	0	CD4_Naive	ETS1
RPL291	0	1.23818	1	0.832	0	CD4_Naive	RPL29
RPL381	0	1.218722	0.995	0.722	0	CD4_Naive	RPL38
RPL371	0	1.211537	1	0.865	0	CD4_Naive	RPL37
RPS161	0	1.208554	1	0.761	0	CD4_Naive	RPS16
FAIM31	0	1.208292	0.6	0.126	0	CD4_Naive	FAIM3
RPL361	0	1.193311	1	0.828	0	CD4_Naive	RPL36
ABLIM11	0	1.192203	0.542	0.083	0	CD4_Naive	ABLIM1
RPL181	0	1.191449	1	0.847	0	CD4_Naive	RPL18
FLT3LG	0	1.174668	0.581	0.132	0	CD4_Naive	FLT3LG
RGCC	0	1.174127	0.531	0.083	0	CD4_Naive	RGCC
PCED1B	0	1.171305	0.496	0.095	0	CD4_Naive	PCED1B
TRABD2A	0	1.169289	0.456	0.046	0	CD4_Naive	TRABD2A
RPLP01	0	1.16865	1	0.773	0	CD4_Naive	RPLP0
LCK	0	1.167151	0.731	0.179	0	CD4_Naive	LCK
OXNAD1	0	1.166174	0.478	0.067	0	CD4_Naive	OXNAD1
RPS81	0	1.16597	1	0.877	0	CD4_Naive	RPS8
RPL23A1	0	1.151619	0.999	0.735	0	CD4_Naive	RPL23A
RPL91	0	1.14334	0.999	0.799	0	CD4_Naive	RPL9
RHOH1	0	1.128281	0.522	0.1	0	CD4_Naive	RHOH
RPS192	0	1.115842	1	0.812	0	CD4_Naive	RPS19
SH3YL1	0	1.114599	0.453	0.069	0	CD4_Naive	SH3YL1
RPS22	0	1.110808	1	0.813	0	CD4_Naive	RPS2
RPL211	0	1.100712	1	0.812	0	CD4_Naive	RPL21
RCAN3	0	1.073446	0.488	0.105	0	CD4_Naive	RCAN3
IFITM1	0	1.070841	0.98	0.322	0	CD4_Naive	IFITM1
RPL221	0	1.069353	1	0.837	0	CD4_Naive	RPL22
GNB2L11	0	1.063116	1	0.796	0	CD4_Naive	GNB2L1
RPS151	0	1.06045	1	0.864	0	CD4_Naive	RPS15
RPL7A1	0	1.058072	1	0.836	0	CD4_Naive	RPL7A
RPL241	0	1.049899	0.998	0.797	0	CD4_Naive	RPL24
RPS281	0	1.049287	1	0.898	0	CD4_Naive	RPS28
TPT1	0	1.029651	1	0.91	0	CD4_Naive	TPT1
TRAT1	0	1.023382	0.417	0.059	0	CD4_Naive	TRAT1
ITM2A	0	1.021453	0.499	0.105	0	CD4_Naive	ITM2A
NACA2	0	1.003042	0.999	0.817	0	CD4_Naive	NACA
RPS131	0	0.97419	1	0.886	0	CD4_Naive	RPS13
PTPRCAP1	0	0.960818	0.928	0.263	0	CD4_Naive	PTPRCAP
RPL121	0	0.955646	1	0.866	0	CD4_Naive	RPL12
RPS71	0	0.94747	1	0.863	0	CD4_Naive	RPS7
TC2N	0	0.936489	0.5	0.107	0	CD4_Naive	TC2N
OCIAD21	0	0.921291	0.462	0.098	0	CD4_Naive	OCIAD2
ID31	0	0.910992	0.327	0.04	0	CD4_Naive	ID3
LRRN3	0	0.906163	0.27	0.015	0	CD4_Naive	LRRN3
EPHX2	0	0.905354	0.32	0.025	0	CD4_Naive	EPHX2
TESPA1	0	0.899757	0.388	0.062	0	CD4_Naive	TESPA1
RPL61	0	0.897351	1	0.862	0	CD4_Naive	RPL6
C14orf64	0	0.893357	0.319	0.029	0	CD4_Naive	C14orf64
RPL391	0	0.879778	1	0.913	0	CD4_Naive	RPL39
CD6	0	0.853592	0.361	0.062	0	CD4_Naive	CD6
LDLRAP1	0	0.843469	0.456	0.086	0	CD4_Naive	LDLRAP1
ITGA6	0	0.826314	0.341	0.055	0	CD4_Naive	ITGA6
ADTRP	0	0.822691	0.257	0.013	0	CD4_Naive	ADTRP

RPL281	0	0.805946	1	0.895	0	CD4_Naive	RPL28
FHIT	0	0.78243	0.267	0.029	0	CD4_Naive	FHIT
CD40LG	0	0.778048	0.306	0.04	0	CD4_Naive	CD40LG
TSHZ2	0	0.74676	0.228	0.017	0	CD4_Naive	TSHZ2
NOG	0	0.742508	0.245	0.009	0	CD4_Naive	NOG
RP11-18H	0	0.727066	0.281	0.036	0	CD4_Naive	RP11-18H21.1
TMEM204	0	0.719411	0.311	0.044	0	CD4_Naive	TMEM204
BACH21	0	0.712378	0.27	0.028	0	CD4_Naive	BACH2
AK5	0	0.566491	0.188	0.017	0	CD4_Naive	AK5
ANKRD55	0	0.530283	0.176	0.01	0	CD4_Naive	ANKRD55
MDS2	0	0.459656	0.152	0.011	0	CD4_Naive	MDS2
IL32	0	0.443558	0.8	0.204	0	CD4_Naive	IL32
RP11-664I	0	0.406091	0.136	0.008	0	CD4_Naive	RP11-664D1.1
RPS261	#######	1.521777	0.991	0.826	#######	CD4_Naive	RPS26
RPL262	#######	0.971958	0.998	0.803	#######	CD4_Naive	RPL26
BCL11B	#######	0.842609	0.345	0.06	#######	CD4_Naive	BCL11B
RPL171	#######	1.194674	0.977	0.71	#######	CD4_Naive	RPL17
SPOCK2	#######	0.905046	0.498	0.116	#######	CD4_Naive	SPOCK2
LEPROTL1	#######	1.113924	0.688	0.242	#######	CD4_Naive	LEPROTL1
RPL82	#######	0.856933	1	0.855	#######	CD4_Naive	RPL8
EVL1	#######	1.069195	0.777	0.28	#######	CD4_Naive	EVL
AES1	#######	1.158869	0.894	0.41	#######	CD4_Naive	AES
IL6ST	#######	0.915489	0.543	0.148	#######	CD4_Naive	IL6ST
TMIGD2	#######	0.632258	0.27	0.038	#######	CD4_Naive	TMIGD2
CHI3L2	#######	0.602928	0.192	0.02	#######	CD4_Naive	CHI3L2
SNHG82	#######	1.180525	0.728	0.285	#######	CD4_Naive	SNHG8
RPLP21	#######	0.732852	1	0.875	#######	CD4_Naive	RPLP2
KLF22	#######	1.160812	0.965	0.521	#######	CD4_Naive	KLF2
RPL42	#######	1.030547	0.986	0.718	#######	CD4_Naive	RPL4
MYC1	#######	1.053271	0.412	0.093	#######	CD4_Naive	MYC
FAM134B	#######	0.64302	0.244	0.034	#######	CD4_Naive	FAM134B
PLEKHB1	#######	0.493105	0.17	0.016	#######	CD4_Naive	PLEKHB1
APBB1	#######	0.627014	0.238	0.032	#######	CD4_Naive	APBB1
CD247	#######	0.658242	0.606	0.164	#######	CD4_Naive	CD247
EEF1D1	#######	0.909549	0.997	0.783	#######	CD4_Naive	EEF1D
INPP4B	#######	0.711692	0.306	0.052	#######	CD4_Naive	INPP4B
FBLN5	#######	0.59649	0.201	0.023	#######	CD4_Naive	FBLN5
CD2	#######	0.811724	0.606	0.17	#######	CD4_Naive	CD2
ITK	#######	0.827975	0.359	0.071	#######	CD4_Naive	ITK
RPL411	#######	0.947741	1	0.882	#######	CD4_Naive	RPL41
GYPC	#######	1.021113	0.689	0.247	#######	CD4_Naive	GYPC
AC006129	#######	0.901723	0.402	0.09	#######	CD4_Naive	AC006129.2
HIST1H4C.	#######	1.11247	0.904	0.475	#######	CD4_Naive	HIST1H4C
THEM4	#######	0.794635	0.358	0.075	#######	CD4_Naive	THEM4
SCML4	#######	0.788979	0.349	0.071	#######	CD4_Naive	SCML4
RPL311	#######	0.910902	0.994	0.73	#######	CD4_Naive	RPL31
AQP3	#######	0.735245	0.351	0.072	#######	CD4_Naive	AQP3
TOMM71	#######	0.92811	0.985	0.696	#######	CD4_Naive	TOMM7
HSPA82	#######	0.987781	0.967	0.569	#######	CD4_Naive	HSPA8
SATB1	#######	0.893801	0.438	0.116	#######	CD4_Naive	SATB1
EIF3E1	#######	1.056642	0.876	0.483	#######	CD4_Naive	EIF3E
AC097713	#######	0.36056	0.109	0.008	#######	CD4_Naive	AC097713.4
RPL351	#######	0.835327	0.997	0.766	#######	CD4_Naive	RPL35
RPL37A1	#######	0.773512	0.996	0.805	#######	CD4_Naive	RPL37A
SUSD3	#######	0.649429	0.326	0.068	#######	CD4_Naive	SUSD3
NELL2	#######	0.616247	0.214	0.031	#######	CD4_Naive	NELL2
RPS91	#######	0.737	0.997	0.827	#######	CD4_Naive	RPS9
NUCB2	#######	0.848652	0.517	0.158	#######	CD4_Naive	NUCB2

AC013264	#######	0.435681	0.137	0.013	#######	CD4_Naive	AC013264.2
STMN11	#######	0.723132	0.322	0.067	#######	CD4_Naive	STMN1
CD5	#######	0.623839	0.271	0.049	#######	CD4_Naive	CD5
RPL13A1	#######	0.808853	0.995	0.762	#######	CD4_Naive	RPL13A
HNRNPA1	#######	0.938287	0.964	0.623	#######	CD4_Naive	HNRNPA1
EEF21	#######	0.913178	0.982	0.693	#######	CD4_Naive	EEF2
11-Sep	#######	0.824941	0.475	0.137	#######	CD4_Naive	1-Sep
BEX21	#######	0.617005	0.256	0.046	#######	CD4_Naive	BEX2
SIRPG	#######	0.562973	0.219	0.034	#######	CD4_Naive	SIRPG
MT-ND4L	#######	0.832647	1	0.77	#######	CD4_Naive	MT-ND4L
MALAT11	#######	0.816766	1	0.943	#######	CD4_Naive	MALAT1
CYTIP1	#######	0.94598	0.64	0.259	#######	CD4_Naive	CYTIP
DENND2D	#######	0.838253	0.549	0.183	#######	CD4_Naive	DENND2D
MLLT3	#######	0.612387	0.259	0.049	#######	CD4_Naive	MLLT3
TRAF3IP31	#######	0.902363	0.822	0.422	#######	CD4_Naive	TRAF3IP3
RPL151	#######	0.725	0.997	0.798	#######	CD4_Naive	RPL15
RPS201	#######	0.919868	0.927	0.584	#######	CD4_Naive	RPS20
SELL2	#######	0.869851	0.884	0.454	#######	CD4_Naive	SELL
DGKA	#######	0.78357	0.4	0.111	#######	CD4_Naive	DGKA
PIM21	#######	0.749801	0.326	0.076	#######	CD4_Naive	PIM2
SELM	#######	0.606474	0.271	0.055	#######	CD4_Naive	SELM
FAU1	#######	0.536647	1	0.901	#######	CD4_Naive	FAU
LAT	#######	0.532452	0.532	0.171	#######	CD4_Naive	LAT
ANXA2R	#######	0.738314	0.341	0.084	#######	CD4_Naive	ANXA2R
SKAP1	#######	0.718462	0.466	0.137	#######	CD4_Naive	SKAP1
CLEC2D1	#######	0.68367	0.343	0.084	#######	CD4_Naive	CLEC2D
CTB-133G	#######	0.900972	0.461	0.151	#######	CD4_Naive	CTB-133G6.1
TCEA3	#######	0.430392	0.146	0.018	#######	CD4_Naive	TCEA3
PDE7A1	#######	0.713683	0.34	0.088	#######	CD4_Naive	PDE7A
FAM102A	#######	0.521579	0.192	0.031	#######	CD4_Naive	FAM102A
PEBP11	#######	0.839552	0.589	0.238	#######	CD4_Naive	PEBP1
RPLP11	#######	0.582053	1	0.943	#######	CD4_Naive	RPLP1
CD28	#######	0.466329	0.203	0.035	#######	CD4_Naive	CD28
STMN3	#######	0.512432	0.223	0.042	#######	CD4_Naive	STMN3
PSIP11	#######	0.78243	0.561	0.222	#######	CD4_Naive	PSIP1
RPS4Y11	#######	1.188508	0.735	0.46	#######	CD4_Naive	RPS4Y1
TXK	#######	0.597811	0.29	0.068	#######	CD4_Naive	TXK
SOD12	#######	0.777571	0.752	0.386	#######	CD4_Naive	SOD1
RIC31	#######	0.468014	0.179	0.029	#######	CD4_Naive	RIC3
PRKCA	#######	0.592457	0.272	0.064	#######	CD4_Naive	PRKCA
FOXP11	#######	0.823073	0.577	0.246	#######	CD4_Naive	FOXP1
STK17A1	#######	0.688622	0.573	0.219	#######	CD4_Naive	STK17A
HINT11	#######	0.755519	0.935	0.605	#######	CD4_Naive	HINT1
RPL271	#######	0.721527	0.985	0.733	#######	CD4_Naive	RPL27
UBASH3A	#######	0.42267	0.16	0.025	#######	CD4_Naive	UBASH3A
OLFM2	#######	0.35517	0.127	0.016	#######	CD4_Naive	OLFM2
COX7C2	#######	0.729181	0.97	0.7	#######	CD4_Naive	COX7C
SNRPD21	#######	0.740992	0.908	0.569	#######	CD4_Naive	SNRPD2
TSTD11	#######	0.706931	0.465	0.166	#######	CD4_Naive	TSTD1
RPL36A2	#######	0.766775	0.947	0.627	#######	CD4_Naive	RPL36A
S1PR11	#######	0.613344	0.364	0.106	#######	CD4_Naive	S1PR1
SNRPN	#######	0.541908	0.333	0.094	#######	CD4_Naive	SNRPN
GIMAP1	#######	0.822256	0.751	0.404	#######	CD4_Naive	GIMAP1
MZT2B1	#######	0.722744	0.826	0.48	#######	CD4_Naive	MZT2B
MT-ATP82	#######	0.778868	0.968	0.732	#######	CD4_Naive	MT-ATP8
ST131	#######	0.745038	0.762	0.428	#######	CD4_Naive	ST13
CHMP71	#######	0.686808	0.326	0.095	#######	CD4_Naive	CHMP7
BTF31	#######	0.65205	0.984	0.737	#######	CD4_Naive	BTF3

LIME1	#######	0.605726	0.333	0.095	#######	CD4_Naive	LIME1
THEMIS	#######	0.468702	0.2	0.04	#######	CD4_Naive	THEMIS
BIN11	#######	0.689428	0.424	0.149	#######	CD4_Naive	BIN1
PPP1R2	#######	0.733292	0.582	0.268	#######	CD4_Naive	PPP1R2
Sep-61	#######	0.622945	0.644	0.305	#######	CD4_Naive	6-Sep
TMEM243	#######	0.689413	0.435	0.164	#######	CD4_Naive	TMEM243
SSR21	#######	0.751158	0.808	0.478	#######	CD4_Naive	SSR2
GPR1831	#######	0.61319	0.287	0.078	#######	CD4_Naive	GPR183
EIF3H1	#######	0.722843	0.857	0.522	#######	CD4_Naive	EIF3H
MZT2A1	#######	0.707808	0.467	0.185	#######	CD4_Naive	MZT2A
ACAP11	#######	0.696577	0.566	0.248	#######	CD4_Naive	ACAP1
HSPB11	#######	0.635996	0.509	0.212	#######	CD4_Naive	HSPB1
IL2RG1	#######	0.484292	0.724	0.324	#######	CD4_Naive	IL2RG
RPL36AL	#######	0.657551	0.974	0.718	#######	CD4_Naive	RPL36AL
PRDX2	#######	0.700506	0.51	0.216	#######	CD4_Naive	PRDX2
ADPRM	#######	0.402461	0.151	0.026	#######	CD4_Naive	ADPRM
HMGN11	#######	0.573661	0.712	0.352	#######	CD4_Naive	HMGN1
PRMT2	#######	0.665018	0.715	0.384	#######	CD4_Naive	PRMT2
RNF144A	#######	0.360756	0.137	0.022	#######	CD4_Naive	RNF144A
EIF3F1	#######	0.709802	0.853	0.536	#######	CD4_Naive	EIF3F
FBL1	#######	0.727935	0.581	0.281	#######	CD4_Naive	FBL
HSP90AB1	#######	0.664564	0.872	0.546	#######	CD4_Naive	HSP90AB1
ARHGAP1!	#######	0.642453	0.491	0.201	#######	CD4_Naive	ARHGAP15
NDFIP1	#######	0.683925	0.633	0.332	#######	CD4_Naive	NDFIP1
CXCR41	#######	0.612217	0.431	0.163	#######	CD4_Naive	CXCR4
UXT1	#######	0.692116	0.742	0.428	#######	CD4_Naive	UXT
SVIP	#######	0.283744	0.404	0.14	#######	CD4_Naive	SVIP
PLCG1	#######	0.40577	0.171	0.034	#######	CD4_Naive	PLCG1
SLC38A11	#######	0.577333	0.351	0.116	#######	CD4_Naive	SLC38A1
LY91	#######	0.51134	0.241	0.062	#######	CD4_Naive	LY9
MT-CYB1	#######	0.469745	0.999	0.93	#######	CD4_Naive	MT-CYB
CCDC109E	#######	0.718488	0.569	0.283	#######	CD4_Naive	CCDC109B
BAG3	#######	0.341625	0.134	0.023	#######	CD4_Naive	BAG3
GCC2	#######	0.677004	0.502	0.223	#######	CD4_Naive	GCC2
ISG201	#######	0.583641	0.449	0.175	#######	CD4_Naive	ISG20
ATP6V0E2	#######	0.495461	0.225	0.058	#######	CD4_Naive	ATP6V0E2
UQCRB1	#######	0.620246	0.952	0.677	#######	CD4_Naive	UQCRB
SERINC5	#######	0.471044	0.223	0.058	#######	CD4_Naive	SERINC5
SNHG71	#######	0.650391	0.471	0.205	#######	CD4_Naive	SNHG7
SIT11	#######	0.52598	0.263	0.075	#######	CD4_Naive	SIT1
C12orf651	#######	0.51675	0.283	0.086	#######	CD4_Naive	C12orf65
THOC3	#######	0.599366	0.293	0.093	#######	CD4_Naive	THOC3
EIF4B1	#######	0.680288	0.741	0.441	#######	CD4_Naive	EIF4B
SCML1	#######	0.310947	0.114	0.018	#######	CD4_Naive	SCML1
RPSAP581	#######	0.685139	0.349	0.129	#######	CD4_Naive	RPSAP58
CMTM8	#######	0.373261	0.134	0.024	#######	CD4_Naive	CMTM8
EIF4A21	#######	0.690321	0.582	0.3	#######	CD4_Naive	EIF4A2
EIF3L1	#######	0.585976	0.849	0.535	#######	CD4_Naive	EIF3L
PASK	#######	0.453053	0.164	0.035	#######	CD4_Naive	PASK
GIMAP4	#######	0.642971	0.829	0.506	#######	CD4_Naive	GIMAP4
AKTIP	#######	0.422535	0.179	0.041	1.41E-99	CD4_Naive	AKTIP
APBA2	#######	0.329258	0.132	0.024	2.99E-99	CD4_Naive	APBA2
C6orf481	#######	0.63611	0.357	0.138	2.64E-98	CD4_Naive	C6orf48
TAGAP1	#######	0.697776	0.516	0.242	2.69E-97	CD4_Naive	TAGAP
DPP4	#######	0.337551	0.128	0.023	1.76E-96	CD4_Naive	DPP4
TMC8	1.14E-99	0.595146	0.383	0.151	3.75E-95	CD4_Naive	TMC8
PDE3B	8.27E-99	0.468044	0.223	0.062	2.71E-94	CD4_Naive	PDE3B
PABPC11	1.37E-98	0.467998	0.996	0.834	4.49E-94	CD4_Naive	PABPC1

IMPDH21	1.85E-98	0.597199	0.339	0.129	6.05E-94	CD4_Naive	IMPDH2
CIRBP2	2.14E-97	0.578292	0.739	0.442	6.99E-93	CD4_Naive	CIRBP
ZNF862	2.25E-96	0.509201	0.25	0.077	7.37E-92	CD4_Naive	ZNF862
MGAT4A	4.68E-95	0.526221	0.312	0.111	1.53E-90	CD4_Naive	MGAT4A
LIMD22	4.61E-94	0.565146	0.895	0.585	1.51E-89	CD4_Naive	LIMD2
SFXN1	1.25E-93	0.433107	0.252	0.078	4.09E-89	CD4_Naive	SFXN1
CSGALNA(	3.59E-93	0.334913	0.157	0.035	1.18E-88	CD4_Naive	CSGALNACT1
HSPD11	3.21E-92	0.577975	0.527	0.265	1.05E-87	CD4_Naive	HSPD1
RARRES3	9.69E-91	0.446707	0.679	0.358	3.17E-86	CD4_Naive	RARRES3
AAK1	1.14E-90	0.537586	0.521	0.258	3.73E-86	CD4_Naive	AAK1
EPB41L4A-	1.16E-90	0.517743	0.264	0.088	3.78E-86	CD4_Naive	EPB41L4A-AS1
RASGRP1	1.47E-89	0.399889	0.191	0.05	4.82E-85	CD4_Naive	RASGRP1
RBL2	5.10E-89	0.581029	0.51	0.252	1.67E-84	CD4_Naive	RBL2
FUS1	6.77E-89	0.579163	0.7	0.423	2.22E-84	CD4_Naive	FUS
FAM159A	7.94E-87	0.30219	0.121	0.024	2.60E-82	CD4_Naive	FAM159A
CD522	1.97E-86	0.489322	0.97	0.644	6.45E-82	CD4_Naive	CD52
HIST1H1D	2.67E-86	0.585189	0.333	0.13	8.75E-82	CD4_Naive	HIST1H1D
NSA2	3.01E-86	0.549085	0.76	0.474	9.85E-82	CD4_Naive	NSA2
TMEM256	5.97E-85	0.516191	0.308	0.117	1.95E-80	CD4_Naive	TMEM256-PLSCR3
MARCKSL1	9.90E-85	0.587173	0.35	0.143	3.24E-80	CD4_Naive	MARCKSL1
SUPT3H	3.45E-84	0.406406	0.17	0.043	1.13E-79	CD4_Naive	SUPT3H
C12orf23	3.69E-84	0.35571	0.158	0.039	1.21E-79	CD4_Naive	C12orf23
IL161	1.03E-83	0.567308	0.498	0.254	3.37E-79	CD4_Naive	IL16
BCL21	1.50E-82	0.435726	0.2	0.057	4.91E-78	CD4_Naive	BCL2
COMMD6	4.18E-82	0.463835	0.943	0.696	1.37E-77	CD4_Naive	COMMD6
PRKCH	1.64E-81	0.433251	0.305	0.11	5.38E-77	CD4_Naive	PRKCH
USP53	2.28E-80	0.274807	0.111	0.021	7.45E-76	CD4_Naive	USP53
GGT7	6.76E-80	0.26789	0.116	0.023	2.21E-75	CD4_Naive	GGT7
TNFAIP81	2.14E-78	0.498281	0.526	0.278	7.00E-74	CD4_Naive	TNFAIP8
ARL4C	2.44E-78	0.429397	0.426	0.186	8.00E-74	CD4_Naive	ARL4C
PITPNA-A	2.81E-78	0.413832	0.186	0.053	9.19E-74	CD4_Naive	PITPNA-AS1
TOMM201	3.20E-78	0.564939	0.592	0.342	1.05E-73	CD4_Naive	TOMM20
RPS241	9.11E-78	0.395853	1	0.881	2.98E-73	CD4_Naive	RPS24
CNN21	2.59E-77	0.474372	0.647	0.382	8.48E-73	CD4_Naive	CNN2
C1QBP1	4.87E-77	0.569194	0.521	0.284	1.60E-72	CD4_Naive	C1QBP
ZAP70	7.13E-77	0.413809	0.318	0.12	2.33E-72	CD4_Naive	ZAP70
TECR	1.28E-76	0.539526	0.449	0.226	4.20E-72	CD4_Naive	TECR
CD96	1.44E-76	0.387008	0.27	0.095	4.73E-72	CD4_Naive	CD96
RPL71	1.49E-75	0.399058	0.991	0.788	4.88E-71	CD4_Naive	RPL7
TMEM123	2.59E-75	0.493698	0.583	0.334	8.48E-71	CD4_Naive	TMEM123
RSL1D11	3.36E-75	0.559196	0.61	0.369	1.10E-70	CD4_Naive	RSL1D1
DDX24	2.37E-74	0.479617	0.618	0.362	7.76E-70	CD4_Naive	DDX24
ZFAS11	2.35E-73	0.494902	0.746	0.477	7.70E-69	CD4_Naive	ZFAS1
SMDT11	5.67E-73	0.482196	0.667	0.408	1.86E-68	CD4_Naive	SMDT1
LINS	9.10E-73	0.398278	0.191	0.058	2.98E-68	CD4_Naive	LINS
PHF11	2.15E-72	0.407028	0.214	0.07	7.05E-68	CD4_Naive	PHF1
GIMAP2	8.19E-72	0.554504	0.407	0.199	2.68E-67	CD4_Naive	GIMAP2
ABHD14B1	4.41E-71	0.509515	0.367	0.169	1.44E-66	CD4_Naive	ABHD14B
UBA521	9.50E-71	0.401303	0.993	0.835	3.11E-66	CD4_Naive	UBA52
MIF1	1.07E-69	0.43352	0.839	0.566	3.50E-65	CD4_Naive	MIF
ANP32B	1.36E-69	0.453529	0.738	0.479	4.44E-65	CD4_Naive	ANP32B
C11orf1	4.41E-69	0.324222	0.148	0.039	1.45E-64	CD4_Naive	C11orf1
SLFN5	5.34E-69	0.452979	0.394	0.182	1.75E-64	CD4_Naive	SLFN5
PBXIP1	6.46E-69	0.413473	0.288	0.114	2.11E-64	CD4_Naive	PBXIP1
PNRC11	6.58E-69	0.459091	0.918	0.665	2.15E-64	CD4_Naive	PNRC1
ENOSF1	1.71E-68	0.348485	0.151	0.041	5.60E-64	CD4_Naive	ENOSF1
RP11-796I	7.50E-68	0.381609	0.203	0.067	2.46E-63	CD4_Naive	RP11-796E2.4
BNIP3	9.37E-68	0.349689	0.176	0.053	3.07E-63	CD4_Naive	BNIP3

ATP5O2	9.42E-68	0.481104	0.749	0.485	3.08E-63	CD4_Naive	ATP5O
CELA1	1.24E-67	0.254276	0.103	0.021	4.06E-63	CD4_Naive	CELA1
LINC00649	1.97E-67	0.314011	0.147	0.039	6.45E-63	CD4_Naive	LINC00649
NMT2	2.25E-67	0.300961	0.13	0.032	7.36E-63	CD4_Naive	NMT2
TBC1D10C	1.43E-66	0.46029	0.426	0.209	4.68E-62	CD4_Naive	TBC1D10C
BCAS41	1.80E-65	0.283689	0.116	0.027	5.90E-61	CD4_Naive	BCAS4
EIF2S31	2.21E-65	0.490511	0.544	0.317	7.22E-61	CD4_Naive	EIF2S3
NCL1	2.41E-65	0.4677	0.754	0.494	7.90E-61	CD4_Naive	NCL
ADD3	6.58E-65	0.409058	0.58	0.341	2.15E-60	CD4_Naive	ADD3
ANAPC161	1.56E-64	0.4478	0.565	0.334	5.11E-60	CD4_Naive	ANAPC16
RAC21	1.36E-63	0.375364	0.854	0.575	4.47E-59	CD4_Naive	RAC2
PNISR1	2.50E-63	0.424775	0.668	0.419	8.17E-59	CD4_Naive	PNISR
DNAJB1	5.80E-63	0.450542	0.303	0.132	1.90E-58	CD4_Naive	DNAJB1
IPCEF1	1.06E-62	0.394746	0.259	0.101	3.45E-58	CD4_Naive	IPCEF1
LGALS3BP	2.01E-61	0.253945	0.117	0.029	6.59E-57	CD4_Naive	LGALS3BP
MAML2	2.05E-61	0.41117	0.264	0.107	6.70E-57	CD4_Naive	MAML2
GOLGA8B	1.58E-60	0.335896	0.159	0.048	5.18E-56	CD4_Naive	GOLGA8B
HSPE11	5.70E-60	0.495523	0.448	0.244	1.87E-55	CD4_Naive	HSPE1
UBXN13	5.72E-60	0.448582	0.7	0.456	1.87E-55	CD4_Naive	UBXN1
TNIK	1.43E-59	0.295816	0.199	0.067	4.68E-55	CD4_Naive	TNIK
DDIT4	2.49E-59	0.398747	0.259	0.101	8.14E-55	CD4_Naive	DDIT4
U2AF1L4	3.54E-59	0.36498	0.199	0.07	1.16E-54	CD4_Naive	U2AF1L4
STX17	1.25E-58	0.387708	0.216	0.08	4.09E-54	CD4_Naive	STX17
HMOX2	6.62E-58	0.435264	0.356	0.172	2.17E-53	CD4_Naive	HMOX2
CCT41	1.03E-57	0.45888	0.47	0.269	3.38E-53	CD4_Naive	CCT4
ATF7IP2	4.84E-57	0.328497	0.144	0.043	1.59E-52	CD4_Naive	ATF7IP2
UBE2D21	9.96E-57	0.39961	0.641	0.419	3.26E-52	CD4_Naive	UBE2D2
LSM5	3.28E-56	0.502183	0.391	0.209	1.07E-51	CD4_Naive	LSM5
ORMDL3	3.33E-56	0.353114	0.188	0.066	1.09E-51	CD4_Naive	ORMDL3
CUTA	6.81E-56	0.407304	0.606	0.382	2.23E-51	CD4_Naive	CUTA
PCBP21	3.17E-55	0.404979	0.877	0.635	1.04E-50	CD4_Naive	PCBP2
CHD3	7.36E-55	0.464404	0.38	0.196	2.41E-50	CD4_Naive	CHD3
CRLF3	1.05E-54	0.422545	0.346	0.172	3.44E-50	CD4_Naive	CRLF3
SESN11	1.14E-53	0.397992	0.184	0.065	3.74E-49	CD4_Naive	SESN1
C19orf53	2.61E-53	0.414755	0.633	0.419	8.53E-49	CD4_Naive	C19orf53
ASF1A	1.49E-52	0.395814	0.22	0.088	4.89E-48	CD4_Naive	ASF1A
CCDC104	2.29E-52	0.284083	0.147	0.046	7.49E-48	CD4_Naive	CCDC104
FOXO11	2.31E-52	0.330191	0.192	0.071	7.57E-48	CD4_Naive	FOXO1
ZBTB25	2.44E-52	0.301129	0.152	0.048	7.99E-48	CD4_Naive	ZBTB25
PCSK1N	5.00E-52	0.275437	0.1	0.025	1.64E-47	CD4_Naive	PCSK1N
PPA1	5.22E-52	0.431663	0.444	0.252	1.71E-47	CD4_Naive	PPA1
RAN1	7.00E-52	0.36508	0.686	0.45	2.29E-47	CD4_Naive	RAN
CYFIP22	2.24E-51	0.39359	0.341	0.168	7.34E-47	CD4_Naive	CYFIP2
ALKBH7	4.49E-51	0.427142	0.511	0.313	1.47E-46	CD4_Naive	ALKBH7
CISH	9.90E-51	0.48518	0.207	0.081	3.24E-46	CD4_Naive	CISH
ZRANB2	2.14E-50	0.407668	0.47	0.275	7.01E-46	CD4_Naive	ZRANB2
HNRNPA0	2.46E-50	0.413848	0.481	0.289	8.05E-46	CD4_Naive	HNRNPA0
GPX7	2.49E-50	0.254665	0.119	0.034	8.16E-46	CD4_Naive	GPX7
SLC2A4RG	2.63E-50	0.307313	0.155	0.052	8.61E-46	CD4_Naive	SLC2A4RG
BUB31	4.03E-50	0.429305	0.386	0.209	1.32E-45	CD4_Naive	BUB3
NIPAL3	3.26E-49	0.326645	0.145	0.047	1.07E-44	CD4_Naive	NIPAL3
ATM	5.98E-49	0.447903	0.46	0.272	1.96E-44	CD4_Naive	ATM
EPC11	7.93E-49	0.428667	0.484	0.293	2.60E-44	CD4_Naive	EPC1
APEX11	9.36E-49	0.434897	0.434	0.25	3.06E-44	CD4_Naive	APEX1
PDCD4-AS	1.02E-48	0.275111	0.116	0.033	3.35E-44	CD4_Naive	PDCD4-AS1
COX6C1	1.88E-48	0.33848	0.824	0.576	6.15E-44	CD4_Naive	COX6C
PIM1	1.98E-48	0.367939	0.343	0.171	6.50E-44	CD4_Naive	PIM1
CHD71	2.04E-48	0.260066	0.123	0.036	6.69E-44	CD4_Naive	CHD7

KIAA0391	3.46E-48	0.258746	0.108	0.029	1.13E-43	CD4_Naive	KIAA0391
CDKN1B1	6.94E-48	0.389297	0.359	0.189	2.27E-43	CD4_Naive	CDKN1B
IMP31	7.44E-48	0.388575	0.457	0.273	2.43E-43	CD4_Naive	IMP3
TNFRSF25	9.12E-48	0.26794	0.108	0.029	2.98E-43	CD4_Naive	TNFRSF25
SLC7A6	1.07E-47	0.317211	0.18	0.067	3.51E-43	CD4_Naive	SLC7A6
NMRK1	1.52E-47	0.301413	0.19	0.073	4.98E-43	CD4_Naive	NMRK1
ILF3-AS1	2.95E-47	0.381871	0.259	0.118	9.64E-43	CD4_Naive	ILF3-AS1
ZCCHC111	9.21E-47	0.397619	0.288	0.137	3.02E-42	CD4_Naive	ZCCHC11
LAGE3	1.47E-46	0.374097	0.296	0.146	4.81E-42	CD4_Naive	LAGE3
CNBP	1.85E-46	0.331055	0.762	0.519	6.04E-42	CD4_Naive	CNBP
CDC25B	2.14E-46	0.34378	0.209	0.085	7.00E-42	CD4_Naive	CDC25B
TXNIP2	2.19E-46	0.299081	0.979	0.742	7.18E-42	CD4_Naive	TXNIP
ISCU1	4.17E-46	0.250103	0.541	0.339	1.37E-41	CD4_Naive	ISCU
MAPKAPK	2.30E-45	0.351831	0.238	0.106	7.53E-41	CD4_Naive	MAPKAPK5-AS1
APRT	2.50E-45	0.356801	0.747	0.511	8.19E-41	CD4_Naive	APRT
POLR1E	4.07E-45	0.26284	0.118	0.036	1.33E-40	CD4_Naive	POLR1E
CYLD	4.81E-45	0.377976	0.373	0.205	1.57E-40	CD4_Naive	CYLD
PAQR8	6.93E-45	0.27662	0.129	0.041	2.27E-40	CD4_Naive	PAQR8
SLC25A45	1.33E-44	0.301449	0.15	0.052	4.34E-40	CD4_Naive	SLC25A45
TMEM238	2.17E-44	0.30228	0.165	0.06	7.09E-40	CD4_Naive	TMEM238
TUBB2	2.48E-44	0.287521	0.583	0.378	8.12E-40	CD4_Naive	TUBB
LYRM5	3.15E-44	0.324635	0.171	0.065	1.03E-39	CD4_Naive	LYRM5
PRKACB	5.08E-44	0.311994	0.297	0.146	1.66E-39	CD4_Naive	PRKACB
PRKCQ	1.24E-43	0.250491	0.132	0.042	4.05E-39	CD4_Naive	PRKCQ
SFI1	3.21E-43	0.253378	0.151	0.053	1.05E-38	CD4_Naive	SFI1
PTPLAD1	4.61E-43	0.292563	0.146	0.051	1.51E-38	CD4_Naive	PTPLAD1
RASA2	4.67E-43	0.362549	0.274	0.132	1.53E-38	CD4_Naive	RASA2
DNAJC19	4.93E-43	0.368815	0.301	0.154	1.62E-38	CD4_Naive	DNAJC19
PFDN52	7.01E-43	0.316555	0.99	0.801	2.30E-38	CD4_Naive	PFDN5
RPL27A1	1.17E-42	0.331453	0.882	0.641	3.81E-38	CD4_Naive	RPL27A
STK4	1.23E-42	0.295198	0.652	0.443	4.02E-38	CD4_Naive	STK4
PPP1R3E	2.35E-42	0.278801	0.159	0.059	7.70E-38	CD4_Naive	PPP1R3E
CCNI2	1.08E-41	0.317055	0.922	0.689	3.53E-37	CD4_Naive	CCNI
SNHG12	1.35E-41	0.30366	0.152	0.056	4.42E-37	CD4_Naive	SNHG12
SBDS	1.59E-41	0.334418	0.246	0.115	5.20E-37	CD4_Naive	SBDS
ZFAND1	1.78E-41	0.330624	0.227	0.103	5.83E-37	CD4_Naive	ZFAND1
ST3GAL1	3.69E-41	0.342784	0.282	0.14	1.21E-36	CD4_Naive	ST3GAL1
CCDC66	4.68E-41	0.325876	0.185	0.075	1.53E-36	CD4_Naive	CCDC66
CD481	8.99E-41	0.306101	0.812	0.572	2.94E-36	CD4_Naive	CD48
EIF3G	1.05E-40	0.333216	0.723	0.515	3.43E-36	CD4_Naive	EIF3G
TMEM106I	1.47E-40	0.314961	0.199	0.085	4.81E-36	CD4_Naive	TMEM106B
KRT101	3.04E-40	0.355627	0.503	0.324	9.97E-36	CD4_Naive	KRT10
ZBTB201	3.69E-40	0.284569	0.156	0.059	1.21E-35	CD4_Naive	ZBTB20
SH2D1A	8.08E-40	0.251099	0.173	0.067	2.65E-35	CD4_Naive	SH2D1A
MRFAP1L1	1.11E-39	0.365862	0.281	0.143	3.64E-35	CD4_Naive	MRFAP1L1
TAF1D1	3.59E-39	0.386873	0.433	0.269	1.18E-34	CD4_Naive	TAF1D
AL592284.	6.90E-39	0.325369	0.184	0.077	2.26E-34	CD4_Naive	AL592284.1
FAM162A1	1.03E-38	0.396853	0.272	0.14	3.37E-34	CD4_Naive	FAM162A
RBM4	1.30E-38	0.371227	0.337	0.189	4.27E-34	CD4_Naive	RBM4
SF11	4.27E-38	0.289238	0.599	0.413	1.40E-33	CD4_Naive	SF1
ERP291	4.93E-38	0.310225	0.755	0.542	1.61E-33	CD4_Naive	ERP29
NPAT	8.20E-38	0.310854	0.195	0.085	2.69E-33	CD4_Naive	NPAT
SNORA76	8.25E-38	0.329406	0.18	0.076	2.70E-33	CD4_Naive	SNORA76
HAX1	1.12E-37	0.33253	0.357	0.206	3.66E-33	CD4_Naive	HAX1
CCT31	1.71E-37	0.383613	0.399	0.243	5.59E-33	CD4_Naive	CCT3
ATIC1	2.13E-37	0.344664	0.205	0.093	6.99E-33	CD4_Naive	ATIC
SYPL11	2.99E-37	0.325268	0.296	0.159	9.79E-33	CD4_Naive	SYPL1
VPS511	3.51E-37	0.385691	0.352	0.205	1.15E-32	CD4_Naive	VPS51

NDNL21	4.05E-37	0.326126	0.218	0.101	1.33E-32	CD4_Naive	NDNL2
NGRN1	6.28E-37	0.323511	0.188	0.082	2.06E-32	CD4_Naive	NGRN
CCT71	7.10E-37	0.352341	0.386	0.233	2.32E-32	CD4_Naive	CCT7
SUCLG2	7.25E-37	0.345825	0.227	0.109	2.37E-32	CD4_Naive	SUCLG2
DDX61	1.01E-36	0.341922	0.328	0.182	3.32E-32	CD4_Naive	DDX6
EIF3D1	1.38E-36	0.363755	0.49	0.324	4.52E-32	CD4_Naive	EIF3D
CCT21	3.16E-36	0.364543	0.353	0.207	1.03E-31	CD4_Naive	CCT2
STAT4	4.11E-36	0.252929	0.179	0.073	1.34E-31	CD4_Naive	STAT4
UBAC2	8.16E-36	0.251175	0.319	0.176	2.67E-31	CD4_Naive	UBAC2
TRDMT1	8.44E-36	0.257096	0.125	0.045	2.76E-31	CD4_Naive	TRDMT1
NOP581	8.49E-36	0.351005	0.304	0.167	2.78E-31	CD4_Naive	NOP58
THAP7	8.68E-36	0.316757	0.182	0.079	2.84E-31	CD4_Naive	THAP7
KIAA14301	1.15E-35	0.332432	0.212	0.099	3.77E-31	CD4_Naive	KIAA1430
BEX4	1.40E-35	0.258435	0.197	0.088	4.58E-31	CD4_Naive	BEX4
RASA3	1.43E-35	0.253553	0.282	0.146	4.69E-31	CD4_Naive	RASA3
MED101	1.62E-35	0.324461	0.346	0.201	5.29E-31	CD4_Naive	MED10
FAM175A	1.89E-35	0.314175	0.161	0.067	6.20E-31	CD4_Naive	FAM175A
DEF6	2.51E-35	0.31509	0.392	0.235	8.21E-31	CD4_Naive	DEF6
LETMD1	2.52E-35	0.334936	0.236	0.117	8.25E-31	CD4_Naive	LETMD1
BIRC31	2.81E-35	0.293549	0.229	0.109	9.21E-31	CD4_Naive	BIRC3
EPB41	2.84E-35	0.259021	0.359	0.206	9.28E-31	CD4_Naive	EPB41
GS1-251I9	3.42E-35	0.323637	0.27	0.14	1.12E-30	CD4_Naive	GS1-251I9.4
HOXB2	1.14E-34	0.263534	0.112	0.038	3.73E-30	CD4_Naive	HOXB2
NDUFA121	1.15E-34	0.29179	0.541	0.364	3.76E-30	CD4_Naive	NDUFA12
LINC-PINT	1.52E-34	0.272943	0.156	0.063	4.99E-30	CD4_Naive	LINC-PINT
SESN3	2.18E-34	0.36006	0.212	0.101	7.12E-30	CD4_Naive	SESN3
C16orf54	2.58E-34	0.29948	0.245	0.124	8.46E-30	CD4_Naive	C16orf54
ZNF493	6.87E-34	0.251456	0.138	0.053	2.25E-29	CD4_Naive	ZNF493
PPP3CC1	7.07E-34	0.321368	0.245	0.123	2.31E-29	CD4_Naive	PPP3CC
SRSF71	1.52E-33	0.344479	0.507	0.342	4.97E-29	CD4_Naive	SRSF7
DBP	1.58E-33	0.319732	0.197	0.091	5.18E-29	CD4_Naive	DBP
MATR31	2.00E-33	0.281538	0.505	0.337	6.55E-29	CD4_Naive	MATR3
SRSF52	2.08E-33	0.281885	0.65	0.462	6.80E-29	CD4_Naive	SRSF5
SNRPF	2.28E-33	0.320702	0.445	0.29	7.47E-29	CD4_Naive	SNRPF
RPA2	2.77E-33	0.297196	0.263	0.138	9.06E-29	CD4_Naive	RPA2
HNRNPDL	5.81E-33	0.292533	0.643	0.458	1.90E-28	CD4_Naive	HNRNPDL
PRR4	5.86E-33	0.250765	0.164	0.069	1.92E-28	CD4_Naive	PRR4
DYRK2	6.16E-33	0.270632	0.162	0.068	2.02E-28	CD4_Naive	DYRK2
PHB21	6.94E-33	0.336572	0.51	0.346	2.27E-28	CD4_Naive	PHB2
CWF19L21	6.97E-33	0.318772	0.254	0.132	2.28E-28	CD4_Naive	CWF19L2
IKZF11	8.08E-33	0.300973	0.507	0.34	2.64E-28	CD4_Naive	IKZF1
N4BP2L2	2.20E-32	0.26858	0.541	0.373	7.21E-28	CD4_Naive	N4BP2L2
RBMX	2.23E-32	0.311114	0.472	0.316	7.29E-28	CD4_Naive	RBMX
PPM1K1	2.30E-32	0.278651	0.227	0.111	7.53E-28	CD4_Naive	PPM1K
SETD5-AS	2.88E-32	0.308504	0.242	0.125	9.42E-28	CD4_Naive	SETD5-AS1
TAPSAR11	3.22E-32	0.332073	0.384	0.238	1.05E-27	CD4_Naive	TAPSAR1
PIGC	6.23E-32	0.30052	0.218	0.108	2.04E-27	CD4_Naive	PIGC
HDAC11	9.57E-32	0.302055	0.358	0.216	3.13E-27	CD4_Naive	HDAC1
SRP9	1.81E-31	0.271011	0.442	0.287	5.91E-27	CD4_Naive	SRP9
SYNJ2BP	2.08E-31	0.264061	0.15	0.063	6.81E-27	CD4_Naive	SYNJ2BP
NSMCE1	2.90E-31	0.353463	0.291	0.165	9.50E-27	CD4_Naive	NSMCE1
ERGIC2	3.62E-31	0.347519	0.234	0.122	1.19E-26	CD4_Naive	ERGIC2
SUN2	4.03E-31	0.26771	0.438	0.281	1.32E-26	CD4_Naive	SUN2
URI11	9.06E-31	0.311663	0.328	0.193	2.96E-26	CD4_Naive	URI1
H1FX	1.25E-30	0.310662	0.579	0.403	4.09E-26	CD4_Naive	H1FX
MAT2B1	1.30E-30	0.281771	0.436	0.284	4.27E-26	CD4_Naive	MAT2B
NUCKS1	3.24E-30	0.301814	0.428	0.281	1.06E-25	CD4_Naive	NUCKS1
SSBP1	4.84E-30	0.275535	0.464	0.311	1.58E-25	CD4_Naive	SSBP1

DDX18	1.31E-29	0.282595	0.48	0.328	4.29E-25	CD4_Naive	DDX18
RSL24D1	3.01E-29	0.276988	0.506	0.348	9.86E-25	CD4_Naive	RSL24D1
KPNA51	3.35E-29	0.251794	0.134	0.055	1.10E-24	CD4_Naive	KPNA5
UBA21	1.07E-28	0.299607	0.277	0.159	3.51E-24	CD4_Naive	UBA2
CHURC1	1.12E-28	0.259356	0.532	0.372	3.68E-24	CD4_Naive	CHURC1
EPHB6	1.75E-28	0.269979	0.129	0.052	5.72E-24	CD4_Naive	EPHB6
ITFG2	1.76E-28	0.305214	0.192	0.094	5.76E-24	CD4_Naive	ITFG2
Sep-91	2.31E-28	0.292693	0.561	0.394	7.56E-24	CD4_Naive	9-Sep
TRAPPC6A	3.15E-28	0.323597	0.265	0.151	1.03E-23	CD4_Naive	TRAPPC6A
UBQLN2	4.75E-28	0.29449	0.22	0.116	1.56E-23	CD4_Naive	UBQLN2
RPIA	8.97E-28	0.29234	0.213	0.111	2.94E-23	CD4_Naive	RPIA
POLR2C	1.38E-27	0.272648	0.191	0.095	4.52E-23	CD4_Naive	POLR2C
C1orf228	1.51E-27	0.266774	0.18	0.086	4.96E-23	CD4_Naive	C1orf228
EIF2A	1.85E-27	0.321802	0.315	0.194	6.07E-23	CD4_Naive	EIF2A
HDDC2	7.07E-27	0.269832	0.247	0.136	2.31E-22	CD4_Naive	HDDC2
MRPS18B1	7.42E-27	0.332642	0.278	0.165	2.43E-22	CD4_Naive	MRPS18B
ADH5	1.11E-26	0.275543	0.303	0.181	3.63E-22	CD4_Naive	ADH5
DUSP111	2.12E-26	0.252714	0.176	0.086	6.95E-22	CD4_Naive	DUSP11
OCIAD11	2.48E-26	0.250067	0.431	0.29	8.13E-22	CD4_Naive	OCIAD1
NGDN1	3.00E-26	0.265807	0.219	0.117	9.83E-22	CD4_Naive	NGDN
KLHDC2	5.70E-26	0.26508	0.188	0.095	1.87E-21	CD4_Naive	KLHDC2
CCDC101	7.14E-26	0.287427	0.186	0.093	2.34E-21	CD4_Naive	CCDC101
FXN	1.78E-25	0.250807	0.155	0.072	5.83E-21	CD4_Naive	FXN
BOLA3	4.91E-25	0.265353	0.162	0.077	1.61E-20	CD4_Naive	BOLA3
ZMYM6NB	1.45E-24	0.286437	0.24	0.136	4.75E-20	CD4_Naive	ZMYM6NB
ILF2	2.45E-24	0.270422	0.375	0.251	8.03E-20	CD4_Naive	ILF2
MPHOSPH	2.56E-24	0.251467	0.484	0.346	8.40E-20	CD4_Naive	MPHOSPH8
CDK41	3.91E-24	0.250692	0.195	0.103	1.28E-19	CD4_Naive	CDK4
MRPS27	4.05E-24	0.263129	0.142	0.066	1.32E-19	CD4_Naive	MRPS27
CLPP	4.18E-24	0.295286	0.256	0.152	1.37E-19	CD4_Naive	CLPP
TAF71	6.49E-24	0.279018	0.337	0.217	2.13E-19	CD4_Naive	TAF7
CCDC911	1.24E-23	0.282903	0.213	0.117	4.07E-19	CD4_Naive	CCDC91
RRP1B	1.96E-23	0.257108	0.203	0.109	6.43E-19	CD4_Naive	RRP1B
THYN1	8.79E-23	0.261988	0.257	0.154	2.88E-18	CD4_Naive	THYN1
SSB	1.82E-22	0.270362	0.427	0.299	5.96E-18	CD4_Naive	SSB
ZC3HAV1	2.25E-22	0.272396	0.312	0.2	7.35E-18	CD4_Naive	ZC3HAV1
METTL5	2.32E-22	0.250981	0.238	0.139	7.59E-18	CD4_Naive	METTL5
KRR11	4.10E-22	0.25345	0.175	0.091	1.34E-17	CD4_Naive	KRR1
CLNS1A1	6.76E-22	0.283883	0.376	0.26	2.21E-17	CD4_Naive	CLNS1A
SIGIRR	9.17E-22	0.262393	0.387	0.267	3.00E-17	CD4_Naive	SIGIRR
ZNF428	1.69E-21	0.262749	0.281	0.175	5.53E-17	CD4_Naive	ZNF428
ZNHIT3	2.27E-21	0.270339	0.223	0.13	7.43E-17	CD4_Naive	ZNHIT3
CNOT7	3.57E-21	0.258007	0.323	0.214	1.17E-16	CD4_Naive	CNOT7
NDUFAF41	4.74E-21	0.253506	0.163	0.084	1.55E-16	CD4_Naive	NDUFAF4
MRPS33	6.06E-21	0.251506	0.228	0.134	1.98E-16	CD4_Naive	MRPS33
CACYBP1	1.38E-20	0.256819	0.324	0.217	4.52E-16	CD4_Naive	CACYBP
SDR39U11	3.21E-20	0.274675	0.194	0.11	1.05E-15	CD4_Naive	SDR39U1
AIP	1.39E-19	0.251447	0.36	0.251	4.57E-15	CD4_Naive	AIP
TTC31	1.77E-18	0.254311	0.335	0.229	5.79E-14	CD4_Naive	TTC3
MTRNR2L1	0.000231	0.43048	0.18	0.143	1	CD4_Naive	MTRNR2L12
IL7R1	0	3.040603	0.888	0.103	0	CD4T	IL7R
LTB2	0	2.909383	0.94	0.233	0	CD4T	LTB
IL321	0	2.258849	0.875	0.166	0	CD4T	IL32
CD3E1	0	2.176522	0.939	0.15	0	CD4T	CD3E
LDHB1	0	2.009718	0.929	0.351	0	CD4T	LDHB
CD3D1	0	1.921325	0.873	0.118	0	CD4T	CD3D
AQP31	0	1.744777	0.599	0.037	0	CD4T	AQP3
CD3G1	0	1.661406	0.782	0.108	0	CD4T	CD3G

TCF71	0	1.624314	0.641	0.077	0	CD4T	TCF7
RPS272	0	1.579076	0.983	0.82	0	CD4T	RPS27
RPSA2	0	1.557608	0.963	0.68	0	CD4T	RPSA
MAL1	0	1.546055	0.535	0.038	0	CD4T	MAL
CD21	0	1.531853	0.734	0.137	0	CD4T	CD2
IFITM11	0	1.514403	0.948	0.29	0	CD4T	IFITM1
RPS182	0	1.512104	0.976	0.775	0	CD4T	RPS18
RPS52	0	1.510855	0.964	0.687	0	CD4T	RPS5
GIMAP51	0	1.504626	0.799	0.236	0	CD4T	GIMAP5
C12orf572	0	1.50076	0.839	0.263	0	CD4T	C12orf57
RPL310	0	1.481896	0.968	0.73	0	CD4T	RPL3
NOSIP1	0	1.479043	0.759	0.276	0	CD4T	NOSIP
RPS122	0	1.474714	0.994	0.874	0	CD4T	RPS12
RPS32	0	1.474144	0.984	0.821	0	CD4T	RPS3
RPS252	0	1.463106	0.972	0.762	0	CD4T	RPS25
SPOCK21	0	1.442505	0.615	0.086	0	CD4T	SPOCK2
EEF1B22	0	1.439787	0.96	0.652	0	CD4T	EEF1B2
EEF1A13	0	1.43139	0.989	0.885	0	CD4T	EEF1A1
RPS292	0	1.427006	0.965	0.751	0	CD4T	RPS29
CCR72	0	1.416443	0.538	0.068	0	CD4T	CCR7
LINC00861	0	1.408528	0.709	0.148	0	CD4T	LINC00861
RPS4X2	0	1.398444	0.984	0.8	0	CD4T	RPS4X
AES2	0	1.379964	0.869	0.387	0	CD4T	AES
PTPRCAP2	0	1.367907	0.91	0.229	0	CD4T	PTPRCAP
RPL142	0	1.367166	0.987	0.798	0	CD4T	RPL14
NPM12	0	1.364427	0.915	0.497	0	CD4T	NPM1
RPL102	0	1.359435	0.993	0.871	0	CD4T	RPL10
LCK1	0	1.358765	0.738	0.15	0	CD4T	LCK
FLT3LG1	0	1.348752	0.594	0.107	0	CD4T	FLT3LG
PRKCQ-AS	0	1.341868	0.56	0.084	0	CD4T	PRKCQ-AS1
RPL52	0	1.332162	0.982	0.787	0	CD4T	RPL5
KLF23	0	1.326901	0.93	0.5	0	CD4T	KLF2
RPS62	0	1.322459	0.971	0.769	0	CD4T	RPS6
ETS12	0	1.319267	0.661	0.155	0	CD4T	ETS1
RPS27A2	0	1.31664	0.989	0.859	0	CD4T	RPS27A
RPLP02	0	1.311813	0.974	0.763	0	CD4T	RPLP0
RPS15A2	0	1.310859	0.987	0.86	0	CD4T	RPS15A
RPL10A2	0	1.309807	0.952	0.669	0	CD4T	RPL10A
LEF11	0	1.298374	0.506	0.058	0	CD4T	LEF1
TMEM662	0	1.286102	0.92	0.523	0	CD4T	TMEM66
HSPA83	0	1.278963	0.94	0.55	0	CD4T	HSPA8
RPL132	0	1.263518	0.987	0.853	0	CD4T	RPL13
RPL302	0	1.260374	0.992	0.879	0	CD4T	RPL30
TRAT11	0	1.255799	0.473	0.036	0	CD4T	TRAT1
ARHGAP1	0	1.248811	0.631	0.174	0	CD4T	ARHGAP15
RPL322	0	1.247147	0.988	0.862	0	CD4T	RPL32
EEF1G2	0	1.242688	0.795	0.504	0	CD4T	EEF1G
GIMAP71	0	1.220158	0.931	0.547	0	CD4T	GIMAP7
CD271	0	1.219079	0.523	0.07	0	CD4T	CD27
RPS193	0	1.200032	0.979	0.804	0	CD4T	RPS19
RPS212	0	1.197792	0.983	0.825	0	CD4T	RPS21
EVL2	0	1.190347	0.766	0.255	0	CD4T	EVL
RPS232	0	1.186778	0.983	0.814	0	CD4T	RPS23
INPP4B1	0	1.182343	0.419	0.029	0	CD4T	INPP4B
RPL23A2	0	1.174056	0.963	0.724	0	CD4T	RPL23A
CISH1	0	1.173221	0.363	0.061	0	CD4T	CISH
RPL18A2	0	1.171543	0.986	0.816	0	CD4T	RPL18A
GPR1832	0	1.160153	0.432	0.055	0	CD4T	GPR183

RPS3A2	0	1.15997	0.991	0.848	0	CD4T	RPS3A
GLTSCR22	0	1.157514	0.843	0.429	0	CD4T	GLTSCR2
CAMK41	0	1.147694	0.469	0.05	0	CD4T	CAMK4
RPL362	0	1.142449	0.977	0.82	0	CD4T	RPL36
IL2RG2	0	1.134022	0.834	0.295	0	CD4T	IL2RG
RPL112	0	1.129229	0.992	0.876	0	CD4T	RPL11
RPL412	0	1.126328	0.989	0.877	0	CD4T	RPL41
RPL342	0	1.118327	0.988	0.866	0	CD4T	RPL34
TAGAP2	0	1.112075	0.627	0.219	0	CD4T	TAGAP
ITM2A1	0	1.109822	0.505	0.083	0	CD4T	ITM2A
RPL292	0	1.089214	0.979	0.825	0	CD4T	RPL29
HINT12	0	1.06658	0.921	0.588	0	CD4T	HINT1
RPL182	0	1.065533	0.988	0.84	0	CD4T	RPL18
RPL192	0	1.065288	0.988	0.862	0	CD4T	RPL19
RPS210	0	1.046096	0.982	0.804	0	CD4T	RPS2
RGCC1	0	1.040134	0.461	0.065	0	CD4T	RGCC
RPS142	0	1.034422	0.987	0.865	0	CD4T	RPS14
RPS82	0	1.03255	0.991	0.871	0	CD4T	RPS8
RPL7A2	0	1.024986	0.987	0.829	0	CD4T	RPL7A
RPL35A2	0	1.020839	0.985	0.863	0	CD4T	RPL35A
RPS162	0	1.020588	0.963	0.752	0	CD4T	RPS16
CD40LG1	0	1.016646	0.37	0.021	0	CD4T	CD40LG
PEBP12	0	1.014312	0.629	0.216	0	CD4T	PEBP1
BTG12	0	0.99861	0.937	0.665	0	CD4T	BTG1
RPL172	0	0.997676	0.914	0.701	0	CD4T	RPL17
ISG202	0	0.995918	0.565	0.151	0	CD4T	ISG20
RHOH2	0	0.991917	0.468	0.082	0	CD4T	RHOH
TC2N1	0	0.982121	0.482	0.088	0	CD4T	TC2N
RPL372	0	0.981948	0.985	0.86	0	CD4T	RPL37
SIT12	0	0.973744	0.416	0.052	0	CD4T	SIT1
RPS102	0	0.973684	0.914	0.754	0	CD4T	RPS10
RCAN31	0	0.968996	0.467	0.086	0	CD4T	RCAN3
LBH2	0	0.964713	0.546	0.138	0	CD4T	LBH
PRDX21	0	0.96442	0.582	0.195	0	CD4T	PRDX2
PCED1B1	0	0.95574	0.425	0.08	0	CD4T	PCED1B
SNHG83	0	0.952024	0.629	0.269	0	CD4T	SNHG8
CD61	0	0.951551	0.377	0.044	0	CD4T	CD6
GNB2L12	0	0.951224	0.972	0.787	0	CD4T	GNB2L1
RPL382	0	0.949981	0.938	0.712	0	CD4T	RPL38
LIME11	0	0.948955	0.435	0.075	0	CD4T	LIME1
OCIAD22	0	0.947469	0.434	0.081	0	CD4T	OCIAD2
S1PR12	0	0.940597	0.464	0.085	0	CD4T	S1PR1
FAIM32	0	0.932606	0.48	0.111	0	CD4T	FAIM3
SKAP11	0	0.932528	0.522	0.115	0	CD4T	SKAP1
CD523	0	0.932113	0.932	0.63	0	CD4T	CD52
RPS152	0	0.930636	0.988	0.858	0	CD4T	RPS15
PIM22	0	0.923575	0.37	0.059	0	CD4T	PIM2
MYC2	0	0.920262	0.377	0.079	0	CD4T	MYC
CD51	0	0.918087	0.34	0.032	0	CD4T	CD5
HNRNPA1	0	0.917043	0.91	0.609	0	CD4T	HNRNPA1
TPT11	0	0.915256	0.984	0.907	0	CD4T	TPT1
HIST1H4C:	0	0.904596	0.828	0.458	0	CD4T	HIST1H4C
DGKA1	0	0.898173	0.426	0.093	0	CD4T	DGKA
TRAF3IP32	0	0.89042	0.782	0.405	0	CD4T	TRAF3IP3
RPL242	0	0.88928	0.973	0.788	0	CD4T	RPL24
RPS72	0	0.880029	0.987	0.856	0	CD4T	RPS7
LEPROTL1	0	0.87879	0.605	0.226	0	CD4T	LEPROTL1
EEF22	0	0.877695	0.932	0.682	0	CD4T	EEF2

RPL92	0	0.875874	0.96	0.791	0	CD4T	RPL9
PBXIP11	0	0.875648	0.407	0.095	0	CD4T	PBXIP1
RPS282	0	0.874958	0.992	0.893	0	CD4T	RPS28
BIN12	0	0.861583	0.496	0.128	0	CD4T	BIN1
PIK3IP11	0	0.859846	0.414	0.087	0	CD4T	PIK3IP1
12-Sep	0	0.857754	0.478	0.119	0	CD4T	1-Sep
MT-ND4L:	0	0.856782	0.974	0.76	0	CD4T	MT-ND4L
RPL122	0	0.855966	0.985	0.86	0	CD4T	RPL12
RPL36A3	0	0.852295	0.9	0.613	0	CD4T	RPL36A
RPL352	0	0.848736	0.957	0.757	0	CD4T	RPL35
RPL83	0	0.846942	0.983	0.849	0	CD4T	RPL8
MIF2	0	0.84467	0.876	0.548	0	CD4T	MIF
ITK1	0	0.842447	0.364	0.056	0	CD4T	ITK
SOD13	0	0.840929	0.752	0.366	0	CD4T	SOD1
RPL43	0	0.837438	0.936	0.708	0	CD4T	RPL4
PIM11	0	0.836784	0.469	0.151	0	CD4T	PIM1
RPL212	0	0.83609	0.965	0.805	0	CD4T	RPL21
TESPA11	0	0.835126	0.36	0.047	0	CD4T	TESPA1
THEM41	0	0.831069	0.358	0.06	0	CD4T	THEM4
RPL282	0	0.827723	0.991	0.89	0	CD4T	RPL28
RPL62	0	0.824662	0.984	0.856	0	CD4T	RPL6
BCL22	0	0.820728	0.304	0.041	0	CD4T	BCL2
AC006129	0	0.818098	0.381	0.075	0	CD4T	AC006129.2
RPL13A2	0	0.818077	0.957	0.753	0	CD4T	RPL13A
ACAP12	0	0.817487	0.608	0.228	0	CD4T	ACAP1
MZT2A2	0	0.808605	0.491	0.168	0	CD4T	MZT2A
TMEM256	0	0.808423	0.398	0.099	0	CD4T	TMEM256-PLSCR3
SYNE2	0	0.792694	0.429	0.101	0	CD4T	SYNE2
EEF1D2	0	0.789454	0.965	0.775	0	CD4T	EEF1D
SH3YL11	0	0.785032	0.344	0.058	0	CD4T	SH3YL1
BCL11B1	0	0.784285	0.32	0.047	0	CD4T	BCL11B
GIMAP41	0	0.780929	0.815	0.49	0	CD4T	GIMAP4
TRADD	0	0.777931	0.4	0.127	0	CD4T	TRADD
TRABD2A1	0	0.773448	0.3	0.037	0	CD4T	TRABD2A
BIRC32	0	0.770623	0.347	0.093	0	CD4T	BIRC3
RPL263	0	0.765496	0.965	0.795	0	CD4T	RPL26
RPS202	0	0.762812	0.862	0.572	0	CD4T	RPS20
CD71	0	0.762103	0.625	0.17	0	CD4T	CD7
RORA	0	0.752585	0.351	0.068	0	CD4T	RORA
RPL392	0	0.752174	0.99	0.909	0	CD4T	RPL39
TOMM72	0	0.751929	0.932	0.685	0	CD4T	TOMM7
MZT2B2	0	0.749629	0.798	0.464	0	CD4T	MZT2B
SNRPD22	0	0.74635	0.868	0.554	0	CD4T	SNRPD2
TTC39C	0	0.744827	0.402	0.115	0	CD4T	TTC39C
GYPC1	0	0.74253	0.571	0.234	0	CD4T	GYPC
SLFN51	0	0.73951	0.451	0.166	0	CD4T	SLFN5
SSR22	0	0.737273	0.787	0.462	0	CD4T	SSR2
RPL222	0	0.735893	0.98	0.83	0	CD4T	RPL22
TSTD12	0	0.73506	0.47	0.15	0	CD4T	TSTD1
THOC31	0	0.733797	0.34	0.079	0	CD4T	THOC3
CTB-133G	0	0.731103	0.423	0.138	0	CD4T	CTB-133G6.1
CD281	0	0.729646	0.277	0.02	0	CD4T	CD28
NACA3	0	0.727187	0.977	0.809	0	CD4T	NACA
GATA3	0	0.72709	0.278	0.039	0	CD4T	GATA3
RPL312	0	0.725608	0.946	0.72	0	CD4T	RPL31
SCML41	0	0.724167	0.329	0.058	0	CD4T	SCML4
EIF3E2	0	0.72396	0.785	0.47	0	CD4T	EIF3E
RPL36AL1	0	0.722298	0.943	0.707	0	CD4T	RPL36AL

PASK1	0	0.719039	0.205	0.025	0	CD4T	PASK
CLEC2D2	0	0.714417	0.353	0.07	0	CD4T	CLEC2D
OXNAD11	0	0.711521	0.321	0.058	0	CD4T	OXNAD1
CYTIP2	0	0.707583	0.575	0.244	0	CD4T	CYTIP
SLC38A12	0	0.70667	0.373	0.102	0	CD4T	SLC38A1
OPTN	0	0.698147	0.374	0.092	0	CD4T	OPTN
ABLIM12	0	0.692866	0.355	0.074	0	CD4T	ABLIM1
PSIP12	0	0.692785	0.514	0.208	0	CD4T	PSIP1
CDC25B1	0	0.690352	0.312	0.07	0	CD4T	CDC25B
RPLP22	0	0.681434	0.986	0.869	0	CD4T	RPLP2
ARL4C1	0	0.681129	0.497	0.168	0	CD4T	ARL4C
PPP1R21	0	0.680697	0.556	0.254	0	CD4T	PPP1R2
ZAP701	0	0.672178	0.408	0.103	0	CD4T	ZAP70
TSHZ21	0	0.659897	0.194	0.009	0	CD4T	TSHZ2
RPL152	0	0.657028	0.962	0.79	0	CD4T	RPL15
BTF32	0	0.65612	0.954	0.727	0	CD4T	BTF3
ID32	0	0.648297	0.212	0.034	0	CD4T	ID3
RPL272	0	0.647625	0.931	0.724	0	CD4T	RPL27
RPS132	0	0.646069	0.988	0.881	0	CD4T	RPS13
CCND2	0	0.645182	0.307	0.069	0	CD4T	CCND2
RP11-18H	0	0.64301	0.241	0.026	0	CD4T	RP11-18H21.1
SLAMF11	0	0.642703	0.23	0.018	0	CD4T	SLAMF1
TBC1D10C	0	0.630653	0.489	0.193	0	CD4T	TBC1D10C
ITGA61	0	0.626977	0.27	0.045	0	CD4T	ITGA6
RAC22	0	0.62466	0.874	0.559	0	CD4T	RAC2
ARID5B1	0	0.615484	0.271	0.06	0	CD4T	ARID5B
RPL37A2	0	0.606734	0.957	0.798	0	CD4T	RPL37A
SIRPG1	0	0.606421	0.229	0.024	0	CD4T	SIRPG
STMN31	0	0.605073	0.235	0.032	0	CD4T	STMN3
RPLP12	0	0.604744	0.992	0.941	0	CD4T	RPLP1
CRIP2	0	0.595019	0.17	0.011	0	CD4T	CRIP2
MT-CYB2	0	0.594324	0.992	0.927	0	CD4T	MT-CYB
LY92	0	0.592853	0.266	0.051	0	CD4T	LY9
TNFRSF25	0	0.591725	0.206	0.017	0	CD4T	TNFRSF25
LAT1	0	0.590873	0.558	0.149	0	CD4T	LAT
SUSD31	0	0.585849	0.307	0.056	0	CD4T	SUSD3
TNFRSF4	0	0.584214	0.171	0.006	0	CD4T	TNFRSF4
SELM1	0	0.581762	0.251	0.045	0	CD4T	SELM
RARRES31	0	0.562599	0.697	0.339	0	CD4T	RARRES3
TMEM204	0	0.556171	0.246	0.036	0	CD4T	TMEM204
SFXN11	0	0.552499	0.277	0.067	0	CD4T	SFXN1
CD961	0	0.54739	0.323	0.081	0	CD4T	CD96
B2M	0	0.54114	0.995	0.978	0	CD4T	B2M
TMEM238	0	0.538407	0.244	0.049	0	CD4T	TMEM238
CCR61	0	0.532902	0.171	0.016	0	CD4T	CCR6
CD2471	0	0.532155	0.56	0.144	0	CD4T	CD247
THEMIS1	0	0.531476	0.212	0.031	0	CD4T	THEMIS
RASGRP11	0	0.531122	0.227	0.04	0	CD4T	RASGRP1
C14orf641	0	0.526509	0.184	0.025	0	CD4T	C14orf64
BEX22	0	0.518698	0.231	0.037	0	CD4T	BEX2
LDLRAP11	0	0.513652	0.317	0.077	0	CD4T	LDLRAP1
ODF2L	0	0.507468	0.254	0.065	0	CD4T	ODF2L
FAU2	0	0.494067	0.987	0.897	0	CD4T	FAU
FAM102A	0	0.483248	0.192	0.023	0	CD4T	FAM102A
FBLN51	0	0.480296	0.176	0.016	0	CD4T	FBLN5
DPP41	0	0.474828	0.167	0.014	0	CD4T	DPP4
CCDC1041	0	0.457533	0.202	0.036	0	CD4T	CCDC104
EPHX21	0	0.444302	0.166	0.022	0	CD4T	EPHX2

NPDC1	0	0.439376	0.147	0.015	0	CD4T	NPDC1
SUPT3H1	0	0.434647	0.187	0.035	0	CD4T	SUPT3H
ADTRP1	0	0.433153	0.129	0.01	0	CD4T	ADTRP
TRIB2	0	0.427519	0.161	0.026	0	CD4T	TRIB2
NELL21	0	0.425386	0.164	0.025	0	CD4T	NELL2
PLCG11	0	0.424181	0.174	0.027	0	CD4T	PLCG1
KCNA3	0	0.420061	0.226	0.04	0	CD4T	KCNA3
PI16	0	0.410758	0.128	0.003	0	CD4T	PI16
CMTM81	0	0.409541	0.147	0.017	0	CD4T	CMTM8
NSG1	0	0.409053	0.141	0.014	0	CD4T	NSG1
UBASH3A1	0	0.409003	0.163	0.017	0	CD4T	UBASH3A
RIC32	0	0.408992	0.155	0.023	0	CD4T	RIC3
TRAF1	0	0.40327	0.155	0.024	0	CD4T	TRAF1
LSR	0	0.38027	0.14	0.017	0	CD4T	LSR
GPR171	0	0.370288	0.139	0.016	0	CD4T	GPR171
GCSAM	0	0.344513	0.123	0.013	0	CD4T	GCSAM
TCEA31	0	0.334652	0.119	0.013	0	CD4T	TCEA3
ATP8B2	0	0.331259	0.132	0.014	0	CD4T	ATP8B2
ANK3	0	0.281625	0.106	0.009	0	CD4T	ANK3
MALAT12	#######	0.664354	0.994	0.941	#######	CD4T	MALAT1
DENND2D	#######	0.559529	0.459	0.171	#######	CD4T	DENND2D
ATP6V0E2	#######	0.447113	0.219	0.05	#######	CD4T	ATP6V0E2
HMGN12	#######	0.571287	0.673	0.337	#######	CD4T	HMGN1
PRKCH1	#######	0.523361	0.329	0.098	#######	CD4T	PRKCH
PLEKHB11	#######	0.300123	0.111	0.013	#######	CD4T	PLEKHB1
HSP90AB1	#######	0.708825	0.855	0.53	#######	CD4T	HSP90AB1
GSTK11	#######	0.759225	0.786	0.493	#######	CD4T	GSTK1
KIAA03911	#######	0.318705	0.143	0.022	#######	CD4T	KIAA0391
RAN2	#######	0.679907	0.75	0.432	#######	CD4T	RAN
ANXA2R1	#######	0.517034	0.276	0.076	#######	CD4T	ANXA2R
Sep-62	#######	0.581492	0.604	0.29	#######	CD4T	6-Sep
FXYD5	#######	0.612689	0.943	0.746	#######	CD4T	FXYD5
PHF12	#######	0.485514	0.24	0.06	#######	CD4T	PHF1
PCSK1N1	#######	0.385497	0.128	0.018	#######	CD4T	PCSK1N
RBL21	#######	0.639351	0.527	0.237	#######	CD4T	RBL2
MDFIC	#######	0.645616	0.37	0.136	#######	CD4T	MDFIC
LINC00649	#######	0.382624	0.168	0.032	#######	CD4T	LINC00649
COX7C3	#######	0.624804	0.925	0.69	#######	CD4T	COX7C
LTA	#######	0.319505	0.111	0.014	#######	CD4T	LTA
HMOX21	#######	0.563526	0.416	0.158	#######	CD4T	HMOX2
FXYD11	#######	0.29203	0.11	0.014	#######	CD4T	FXYD1
KLRB1	#######	0.787685	0.305	0.091	#######	CD4T	KLRB1
EIF3F2	#######	0.648806	0.82	0.522	#######	CD4T	EIF3F
CXCR42	#######	0.590303	0.402	0.152	#######	CD4T	CXCR4
CNN22	#######	0.628276	0.673	0.366	#######	CD4T	CNN2
HSPB12	#######	0.572887	0.471	0.2	#######	CD4T	HSPB1
CTC-523E2	#######	0.308337	0.12	0.017	#######	CD4T	CTC-523E23.11
SERINC51	#######	0.448414	0.207	0.05	#######	CD4T	SERINC5
RPSAP582	#######	0.612915	0.337	0.118	#######	CD4T	RPSAP58
SOCS1	#######	0.357715	0.132	0.021	#######	CD4T	SOCS1
TNFAIP82	#######	0.581483	0.547	0.263	#######	CD4T	TNFAIP8
CXCR3	#######	0.539979	0.18	0.039	#######	CD4T	CXCR3
EIF4A22	#######	0.617091	0.568	0.286	#######	CD4T	EIF4A2
AC006369	#######	0.290286	0.121	0.018	#######	CD4T	AC006369.2
TECR1	#######	0.568177	0.475	0.212	#######	CD4T	TECR
PRKCQ1	#######	0.331789	0.168	0.035	#######	CD4T	PRKCQ
CD691	#######	0.451946	0.206	0.051	#######	CD4T	CD69
RPS4Y12	#######	0.953926	0.66	0.451	#######	CD4T	RPS4Y1

NMRK11	#######	0.408594	0.23	0.063	#######	CD4T	NMRK1
DDX241	#######	0.606495	0.639	0.347	#######	CD4T	DDX24
MYL12A	#######	0.268844	0.951	0.746	#######	CD4T	MYL12A
ASF1A1	#######	0.469078	0.255	0.078	#######	CD4T	ASF1A
SH2D3A	#######	0.260869	0.101	0.014	#######	CD4T	SH2D3A
CLDND1	#######	0.516321	0.329	0.119	#######	CD4T	CLDND1
TNIK1	#######	0.352572	0.221	0.059	#######	CD4T	TNIK
FAM159A1	#######	0.30791	0.117	0.019	#######	CD4T	FAM159A
APBB11	#######	0.369545	0.146	0.029	#######	CD4T	APBB1
MGAT4A1	#######	0.494888	0.299	0.101	#######	CD4T	MGAT4A
TMEM123	#######	0.589167	0.594	0.32	#######	CD4T	TMEM123
CYLD1	#######	0.553964	0.434	0.192	#######	CD4T	CYLD
FKBP11	#######	0.478079	0.256	0.078	#######	CD4T	FKBP11
RPS262	#######	0.918123	0.964	0.819	#######	CD4T	RPS26
HSPE12	#######	0.604021	0.487	0.23	#######	CD4T	HSPE1
CCDC167	#######	0.482787	0.218	0.061	#######	CD4T	CCDC167
ABHD14B2	#######	0.543681	0.382	0.158	#######	CD4T	ABHD14B
KIAA14302	#######	0.475936	0.271	0.088	#######	CD4T	KIAA1430
MT-ATP83	#######	0.627813	0.899	0.725	#######	CD4T	MT-ATP8
C12orf652	#######	0.468562	0.252	0.078	#######	CD4T	C12orf65
MLLT31	#######	0.359877	0.182	0.044	#######	CD4T	MLLT3
CDKN1B2	#######	0.535733	0.408	0.176	#######	CD4T	CDKN1B
FAM134B1	#######	0.328206	0.146	0.031	#######	CD4T	FAM134B
ITGB7	#######	0.467165	0.31	0.11	#######	CD4T	ITGB7
PPP3CC2	#######	0.461756	0.31	0.111	#######	CD4T	PPP3CC
FAM107B1	#######	0.480286	0.575	0.301	#######	CD4T	FAM107B
GOLGA8B1	#######	0.373246	0.17	0.041	#######	CD4T	GOLGA8B
SNRPN1	#######	0.317613	0.266	0.087	#######	CD4T	SNRPN
GIMAP11	#######	0.55545	0.675	0.392	#######	CD4T	GIMAP1
PRKCA1	#######	0.376318	0.206	0.059	#######	CD4T	PRKCA
RPS92	#######	0.461362	0.948	0.822	#######	CD4T	RPS9
GCC21	#######	0.585901	0.452	0.212	#######	CD4T	GCC2
CD320	#######	0.372941	0.188	0.05	#######	CD4T	CD320
ANKRD12	#######	0.536379	0.645	0.373	#######	CD4T	ANKRD12
NMT21	#######	0.311803	0.131	0.027	#######	CD4T	NMT2
STK17A2	#######	0.366491	0.471	0.209	#######	CD4T	STK17A
BOLA31	#######	0.401611	0.219	0.068	#######	CD4T	BOLA3
CCDC109B	#######	0.54233	0.518	0.272	#######	CD4T	CCDC109B
ZMYM6NB	#######	0.4588	0.315	0.125	#######	CD4T	ZMYM6NB
LINS1	#######	0.358519	0.186	0.052	#######	CD4T	LINS
ADD31	#######	0.445117	0.591	0.328	#######	CD4T	ADD3
EML42	#######	0.531752	0.441	0.211	#######	CD4T	EML4
BNIP31	#######	0.341857	0.175	0.047	#######	CD4T	BNIP3
APRT1	#######	0.513421	0.766	0.497	#######	CD4T	APRT
GPRIN3	#######	0.474838	0.262	0.094	#######	CD4T	GPRIN3
HSPD12	#######	0.621108	0.491	0.254	#######	CD4T	HSPD1
CORO1B1	#######	0.62652	0.423	0.208	#######	CD4T	CORO1B
HELB	#######	0.317517	0.151	0.037	#######	CD4T	HELB
EIF3H2	#######	0.530051	0.774	0.511	#######	CD4T	EIF3H
CRIP11	#######	0.58179	0.784	0.509	#######	CD4T	CRIP1
EPC12	#######	0.519244	0.526	0.28	#######	CD4T	EPC1
CALM1	#######	0.393461	0.941	0.743	#######	CD4T	CALM1
SLFN12L	#######	0.326796	0.151	0.037	#######	CD4T	SLFN12L
DNAJB11	#######	0.500546	0.307	0.123	#######	CD4T	DNAJB1
AKTIP1	#######	0.307837	0.149	0.036	#######	CD4T	AKTIP
IL162	#######	0.517919	0.476	0.243	#######	CD4T	IL16
CIRBP3	#######	0.476558	0.705	0.429	#######	CD4T	CIRBP
UXT2	#######	0.526466	0.673	0.417	#######	CD4T	UXT

ORMDL31	#######	0.349015	0.196	0.058	#######	CD4T	ORMDL3
CYFIP23	#######	0.449429	0.364	0.157	#######	CD4T	CYFIP2
PPA11	#######	0.506646	0.47	0.239	#######	CD4T	PPA1
SNHG121	#######	0.375753	0.175	0.049	#######	CD4T	SNHG12
RHOF	#######	0.388061	0.353	0.15	#######	CD4T	RHOF
SOCS2	#######	0.262846	0.105	0.019	#######	CD4T	SOCS2
NCL2	#######	0.519209	0.753	0.48	#######	CD4T	NCL
KLF12	#######	0.376406	0.195	0.059	#######	CD4T	KLF12
ICAM21	#######	0.296273	0.475	0.235	#######	CD4T	ICAM2
MAT2B2	#######	0.469848	0.508	0.27	#######	CD4T	MAT2B
AP3M2	#######	0.267984	0.111	0.022	#######	CD4T	AP3M2
SPTAN1	#######	0.48509	0.344	0.149	#######	CD4T	SPTAN1
P2RY101	#######	0.278035	0.122	0.026	#######	CD4T	P2RY10
AAK11	#######	0.448125	0.488	0.247	#######	CD4T	AAK1
TMEM243	#######	0.452162	0.351	0.156	#######	CD4T	TMEM243
SYTL11	#######	0.440987	0.355	0.154	#######	CD4T	SYTL1
TNFSF8	#######	0.28391	0.122	0.026	#######	CD4T	TNFSF8
CDC14A	#######	0.29642	0.161	0.044	#######	CD4T	CDC14A
CUTA1	#######	0.464575	0.621	0.369	#######	CD4T	CUTA
FBL2	#######	0.50954	0.501	0.272	#######	CD4T	FBL
IMPDH22	#######	0.460171	0.297	0.122	#######	CD4T	IMPDH2
COMMD6	#######	0.435757	0.911	0.686	#######	CD4T	COMMD6
BUB32	#######	0.480427	0.408	0.198	#######	CD4T	BUB3
TXNIP3	#######	0.4644	0.944	0.732	#######	CD4T	TXNIP
ZNF101	#######	0.353016	0.169	0.049	#######	CD4T	ZNF101
MAF	#######	0.384186	0.141	0.036	#######	CD4T	MAF
NIPAL31	#######	0.288937	0.153	0.041	#######	CD4T	NIPAL3
EPB411	#######	0.376211	0.406	0.194	#######	CD4T	EPB41
EIF4B2	#######	0.493057	0.679	0.43	#######	CD4T	EIF4B
STAT41	#######	0.30487	0.206	0.066	#######	CD4T	STAT4
TMC81	#######	0.448108	0.329	0.143	#######	CD4T	TMC8
FBXO32	#######	0.3001	0.116	0.026	#######	CD4T	FBXO32
LY6E1	#######	0.375255	0.63	0.355	#######	CD4T	LY6E
PDE7A2	#######	0.387618	0.23	0.083	#######	CD4T	PDE7A
MRFAP1L1	#######	0.40433	0.311	0.133	#######	CD4T	MRFAP1L1
CNBP1	#######	0.424674	0.761	0.507	#######	CD4T	CNBP
PDE3B1	#######	0.358016	0.182	0.057	#######	CD4T	PDE3B
CHMP72	#######	0.419532	0.24	0.09	#######	CD4T	CHMP7
FOXP12	#######	0.536225	0.448	0.239	#######	CD4T	FOXP1
ZBTB251	#######	0.298615	0.152	0.043	#######	CD4T	ZBTB25
C1QBP2	#######	0.465904	0.493	0.274	#######	CD4T	C1QBP
ERN1	#######	0.419963	0.232	0.086	#######	CD4T	ERN1
UBE2D22	#######	0.421043	0.65	0.406	#######	CD4T	UBE2D2
KDSR	#######	0.391679	0.207	0.073	#######	CD4T	KDSR
NGRN2	#######	0.36971	0.21	0.075	#######	CD4T	NGRN
C16orf541	#######	0.386674	0.277	0.115	#######	CD4T	C16orf54
TOMM202	#######	0.466808	0.56	0.332	#######	CD4T	TOMM20
ANXA61	#######	0.340944	0.596	0.347	#######	CD4T	ANXA6
FAM162A2	#######	0.397791	0.3	0.13	#######	CD4T	FAM162A
REXO2	#######	0.36164	0.254	0.101	#######	CD4T	REXO2
PRDM1	#######	0.283016	0.145	0.04	#######	CD4T	PRDM1
CCSER2	#######	0.348024	0.246	0.096	#######	CD4T	CCSER2
TUBB3	#######	0.372101	0.605	0.366	#######	CD4T	TUBB
HNRNPF	#######	0.431436	0.609	0.373	#######	CD4T	HNRNPF
SH2D1A1	#######	0.294763	0.185	0.06	#######	CD4T	SH2D1A
HNRNPA0	#######	0.430052	0.493	0.278	#######	CD4T	HNRNPA0
SMDT12	#######	0.48445	0.626	0.397	#######	CD4T	SMDT1
LINC-PINT	#######	0.298198	0.175	0.057	#######	CD4T	LINC-PINT

PDCD41	#######	0.434377	0.413	0.214	#######	CD4T	PDCD4
SKP1	#######	0.359398	0.802	0.556	#######	CD4T	SKP1
HNRNPLL	#######	0.299741	0.161	0.05	#######	CD4T	HNRNPLL
NFATC2	#######	0.299915	0.207	0.073	#######	CD4T	NFATC2
LIMD23	#######	0.432735	0.825	0.574	#######	CD4T	LIMD2
GPX71	#######	0.255525	0.117	0.029	#######	CD4T	GPX7
HAX11	#######	0.384631	0.385	0.196	#######	CD4T	HAX1
DDIT41	#######	0.369773	0.243	0.094	#######	CD4T	DDIT4
MEI1	#######	0.254008	0.138	0.039	#######	CD4T	MEI1
URI12	#######	0.421829	0.368	0.183	#######	CD4T	URI1
CCT72	#######	0.43609	0.417	0.223	#######	CD4T	CCT7
CLPP1	#######	0.397057	0.308	0.142	#######	CD4T	CLPP
ESYT1	#######	0.378758	0.247	0.101	#######	CD4T	ESYT1
ST132	#######	0.417814	0.651	0.419	#######	CD4T	ST13
WHSC1L11	#######	0.38897	0.462	0.256	#######	CD4T	WHSC1L1
MED102	#######	0.387136	0.378	0.19	#######	CD4T	MED10
SATB11	#######	0.408627	0.265	0.113	#######	CD4T	SATB1
ZC3HAV11	#######	0.411515	0.375	0.189	#######	CD4T	ZC3HAV1
ZNF8621	#######	0.329384	0.199	0.072	#######	CD4T	ZNF862
SS18L21	#######	0.405912	0.336	0.163	#######	CD4T	SS18L2
PTGES31	#######	0.389118	0.67	0.43	#######	CD4T	PTGES3
GTF3A1	#######	0.407788	0.551	0.334	#######	CD4T	GTF3A
PTGER2	#######	0.425509	0.249	0.103	#######	CD4T	PTGER2
HIST1H1D	#######	0.40747	0.283	0.124	#######	CD4T	HIST1H1D
ANAPC162	#######	0.411753	0.54	0.323	#######	CD4T	ANAPC16
C12orf231	#######	0.25228	0.127	0.035	#######	CD4T	C12orf23
CASP6	#######	0.254712	0.13	0.037	#######	CD4T	CASP6
GIMAP21	#######	0.385389	0.375	0.191	#######	CD4T	GIMAP2
NDFIP11	#######	0.37411	0.538	0.324	#######	CD4T	NDFIP1
CD59	#######	0.274977	0.132	0.038	#######	CD4T	CD59
ATIC2	#######	0.353771	0.218	0.086	#######	CD4T	ATIC
TPD521	#######	0.266201	0.143	0.043	#######	CD4T	TPD52
CCT42	#######	0.402045	0.462	0.259	#######	CD4T	CCT4
SLC9A3R1	#######	0.280095	0.531	0.303	#######	CD4T	SLC9A3R1
SLC2A4RG	#######	0.297162	0.149	0.047	#######	CD4T	SLC2A4RG
ICAM31	#######	0.33237	0.622	0.387	#######	CD4T	ICAM3
PDE4B1	#######	0.267657	0.169	0.057	#######	CD4T	PDE4B
STK41	#######	0.353041	0.658	0.432	#######	CD4T	STK4
SESN12	#######	0.391696	0.171	0.059	#######	CD4T	SESN1
SUN21	#######	0.378096	0.475	0.27	#######	CD4T	SUN2
PHB22	#######	0.396101	0.545	0.335	#######	CD4T	PHB2
C19orf531	#######	0.37736	0.628	0.408	#######	CD4T	C19orf53
S1PR41	#######	0.361335	0.464	0.266	#######	CD4T	S1PR4
DIMT1	#######	0.323073	0.156	0.053	#######	CD4T	DIMT1
TNFRSF10A	#######	0.282539	0.127	0.037	#######	CD4T	TNFRSF10A
RSL1D12	#######	0.413401	0.574	0.359	#######	CD4T	RSL1D1
ANP32B1	#######	0.333612	0.694	0.469	#######	CD4T	ANP32B
ATF7IP21	#######	0.285059	0.129	0.038	#######	CD4T	ATF7IP2
SPTBN11	#######	0.283555	0.185	0.068	#######	CD4T	SPTBN1
TXK1	#######	0.255219	0.183	0.065	#######	CD4T	TXK
SRP91	#######	0.337509	0.475	0.276	#######	CD4T	SRP9
SH3BP51	#######	0.316719	0.346	0.172	#######	CD4T	SH3BP5
TMEM106I	#######	0.295528	0.201	0.078	#######	CD4T	TMEM106B
SIGIRR1	#######	0.341469	0.451	0.256	#######	CD4T	SIGIRR
PITPNA-A	#######	0.303567	0.149	0.049	#######	CD4T	PITPNA-AS1
PNRC12	#######	0.365754	0.881	0.654	#######	CD4T	PNRC1
CCDC661	#######	0.289612	0.185	0.07	#######	CD4T	CCDC66
ORAI1	#######	0.284527	0.41	0.222	#######	CD4T	ORAI1

RRP1B1	#######	0.339592	0.235	0.102	#######	CD4T	RRP1B
SLC25A38	#######	0.250213	0.14	0.045	#######	CD4T	SLC25A38
CDC42SE2	#######	0.302343	0.397	0.216	#######	CD4T	CDC42SE2
SRSF72	#######	0.371778	0.536	0.331	#######	CD4T	SRSF7
MAPKAPK	#######	0.322704	0.232	0.099	#######	CD4T	MAPKAPK5-AS1
ADH51	#######	0.34363	0.338	0.172	#######	CD4T	ADH5
SBDS1	#######	0.356757	0.244	0.109	#######	CD4T	SBDS
ERGIC21	#######	0.330612	0.253	0.114	#######	CD4T	ERGIC2
PPM1K2	#######	0.331304	0.24	0.104	#######	CD4T	PPM1K
SP1401	#######	0.272526	0.181	0.068	#######	CD4T	SP140
EIF5A1	#######	0.354193	0.605	0.393	#######	CD4T	EIF5A
CD482	#######	0.365219	0.794	0.561	#######	CD4T	CD48
PARP11	#######	0.368955	0.37	0.2	#######	CD4T	PARP1
IMP32	#######	0.360822	0.451	0.264	#######	CD4T	IMP3
PIK3R1	#######	0.384315	0.371	0.201	#######	CD4T	PIK3R1
PTPN7	#######	0.260758	0.17	0.062	#######	CD4T	PTPN7
UBE2N1	#######	0.35441	0.346	0.184	#######	CD4T	UBE2N
TRAPPC6A	#######	0.359153	0.289	0.143	#######	CD4T	TRAPPC6A
TBC1D4	#######	0.310741	0.109	0.031	#######	CD4T	TBC1D4
HMGN41	#######	0.325116	0.219	0.094	#######	CD4T	HMGN4
TAF1D2	#######	0.379563	0.444	0.259	#######	CD4T	TAF1D
HNRNPDL	#######	0.356692	0.672	0.445	#######	CD4T	HNRNPDL
DAD1	#######	0.357396	0.606	0.391	#######	CD4T	DAD1
ENOSF11	#######	0.255944	0.122	0.037	#######	CD4T	ENOSF1
NHP2L11	#######	0.359336	0.545	0.345	#######	CD4T	NHP2L1
FUT7	#######	0.335327	0.154	0.055	#######	CD4T	FUT7
FAS	#######	0.27765	0.144	0.049	#######	CD4T	FAS
RPL27A2	#######	0.35706	0.846	0.631	#######	CD4T	RPL27A
DUT	#######	0.397136	0.427	0.25	#######	CD4T	DUT
C14orf1	#######	0.265917	0.154	0.055	#######	CD4T	C14orf1
ABRACL1	#######	0.319793	0.645	0.424	#######	CD4T	ABRACL
PRPS1	#######	0.264291	0.157	0.056	#######	CD4T	PRPS1
THAP71	#######	0.305795	0.184	0.073	#######	CD4T	THAP7
UBXN14	#######	0.336749	0.665	0.446	#######	CD4T	UBXN1
DNAJB9	#######	0.261427	0.164	0.061	#######	CD4T	DNAJB9
CCT32	#######	0.403791	0.404	0.234	#######	CD4T	CCT3
DNAJC191	#######	0.341199	0.294	0.147	1.79E-99	CD4T	DNAJC19
ZCCHC11	#######	0.303549	0.274	0.13	2.04E-99	CD4T	ZCCHC11
NSA21	#######	0.33256	0.678	0.466	6.00E-99	CD4T	NSA2
POLR3GL	#######	0.337643	0.355	0.192	8.85E-98	CD4T	POLR3GL
EIF3L2	#######	0.303817	0.755	0.526	1.50E-97	CD4T	EIF3L
TMC61	#######	0.335571	0.301	0.152	1.89E-97	CD4T	TMC6
KRR12	#######	0.315344	0.2	0.085	2.64E-97	CD4T	KRR1
CA5B	#######	0.275907	0.185	0.075	3.18E-97	CD4T	CA5B
MAP4K11	#######	0.297624	0.174	0.069	4.28E-97	CD4T	MAP4K1
NAP1L4	#######	0.286048	0.434	0.255	1.09E-96	CD4T	NAP1L4
SNHG72	#######	0.353099	0.361	0.2	1.17E-95	CD4T	SNHG7
DDX62	#######	0.30613	0.33	0.174	1.44E-95	CD4T	DDX6
NOP582	#######	0.350503	0.307	0.159	1.94E-95	CD4T	NOP58
COX6C2	5.03E-99	0.333432	0.795	0.565	1.65E-94	CD4T	COX6C
FUS2	8.69E-99	0.334628	0.622	0.415	2.84E-94	CD4T	FUS
SPCS1	5.51E-98	0.274655	0.633	0.416	1.80E-93	CD4T	SPCS1
RASSF7	7.21E-98	0.257508	0.159	0.06	2.36E-93	CD4T	RASSF7
CCDC107	9.65E-98	0.351022	0.316	0.167	3.16E-93	CD4T	CCDC107
FNTA	1.02E-97	0.323562	0.366	0.205	3.36E-93	CD4T	FNTA
XRCC61	1.04E-97	0.302946	0.548	0.348	3.40E-93	CD4T	XRCC6
RBM41	1.54E-97	0.28908	0.335	0.181	5.05E-93	CD4T	RBM4
UBIAD11	7.07E-97	0.252459	0.131	0.045	2.32E-92	CD4T	UBIAD1

STX171	9.99E-96	0.276955	0.183	0.076	3.27E-91	CD4T	STX17
UBA22	1.33E-95	0.316918	0.293	0.152	4.37E-91	CD4T	UBA2
HMCES	1.91E-95	0.255329	0.154	0.058	6.26E-91	CD4T	HMCES
LSM51	2.56E-95	0.351588	0.358	0.202	8.37E-91	CD4T	LSM5
RASA31	5.87E-95	0.254145	0.28	0.139	1.92E-90	CD4T	RASA3
SORBS3	7.55E-95	0.256106	0.118	0.039	2.47E-90	CD4T	SORBS3
STMN12	7.99E-94	0.291037	0.168	0.066	2.61E-89	CD4T	STMN1
MFNG	1.37E-93	0.28637	0.308	0.163	4.47E-89	CD4T	MFNG
TBCC	1.53E-93	0.318939	0.219	0.101	5.00E-89	CD4T	TBCC
NDNL22	2.29E-93	0.277163	0.213	0.096	7.48E-89	CD4T	NDNL2
THAP11	3.82E-93	0.265738	0.266	0.134	1.25E-88	CD4T	THAP11
RPA21	4.20E-93	0.287714	0.266	0.131	1.38E-88	CD4T	RPA2
ATP1A11	5.18E-93	0.339984	0.404	0.239	1.69E-88	CD4T	ATP1A1
ADSL	2.04E-92	0.321726	0.288	0.151	6.67E-88	CD4T	ADSL
ISCU2	2.13E-91	0.261612	0.518	0.33	6.99E-87	CD4T	ISCU
DEF61	2.42E-91	0.283736	0.392	0.227	7.91E-87	CD4T	DEF6
GORASP2	2.77E-91	0.304877	0.248	0.122	9.08E-87	CD4T	GORASP2
SYPL12	3.96E-91	0.295391	0.289	0.152	1.30E-86	CD4T	SYPL1
SSB1	1.09E-90	0.354487	0.461	0.29	3.58E-86	CD4T	SSB
MEAF6	1.80E-90	0.305364	0.374	0.214	5.90E-86	CD4T	MEAF6
LMAN1	3.62E-90	0.296534	0.278	0.142	1.18E-85	CD4T	LMAN1
GLOD4	5.20E-90	0.332147	0.263	0.133	1.70E-85	CD4T	GLOD4
GALT	8.75E-90	0.266253	0.163	0.066	2.86E-85	CD4T	GALT
PRMT21	2.15E-89	0.255926	0.573	0.378	7.05E-85	CD4T	PRMT2
PCBP1	2.24E-89	0.288638	0.849	0.639	7.34E-85	CD4T	PCBP1
APEX12	2.55E-89	0.359213	0.407	0.243	8.34E-85	CD4T	APEX1
RCN21	2.85E-89	0.297398	0.239	0.115	9.33E-85	CD4T	RCN2
PPIA2	6.55E-89	0.323228	0.923	0.72	2.14E-84	CD4T	PPIA
DBP1	6.74E-89	0.291636	0.194	0.086	2.21E-84	CD4T	DBP
METTL51	7.61E-89	0.312614	0.261	0.132	2.49E-84	CD4T	METTL5
RNF214	1.06E-88	0.26931	0.14	0.053	3.47E-84	CD4T	RNF214
NDUFV2	1.65E-88	0.276736	0.498	0.315	5.42E-84	CD4T	NDUFV2
SF12	1.99E-88	0.295938	0.598	0.403	6.53E-84	CD4T	SF1
Sep-92	9.21E-88	0.331212	0.578	0.384	3.01E-83	CD4T	9-Sep
TOB1	9.86E-88	0.262426	0.184	0.08	3.23E-83	CD4T	TOB1
PDCD51	1.71E-87	0.330984	0.388	0.232	5.58E-83	CD4T	PDCD5
P2RY81	3.24E-87	0.291328	0.235	0.113	1.06E-82	CD4T	P2RY8
NUDCD2	2.90E-86	0.278295	0.238	0.117	9.49E-82	CD4T	NUDCD2
ELOVL51	5.28E-86	0.301401	0.323	0.179	1.73E-81	CD4T	ELOVL5
TSR2	1.02E-85	0.272634	0.211	0.098	3.33E-81	CD4T	TSR2
DARS1	3.77E-85	0.282058	0.291	0.156	1.24E-80	CD4T	DARS
ARHGEF3	1.08E-84	0.259274	0.275	0.141	3.55E-80	CD4T	ARHGEF3
MRPS18B2	1.91E-84	0.29077	0.291	0.158	6.26E-80	CD4T	MRPS18B
LPIN2	2.80E-84	0.262017	0.28	0.147	9.17E-80	CD4T	LPIN2
DOCK10	2.81E-84	0.299424	0.261	0.135	9.20E-80	CD4T	DOCK10
ST3GAL11	3.12E-84	0.292446	0.263	0.134	1.02E-79	CD4T	ST3GAL1
DYRK21	4.40E-84	0.252551	0.157	0.064	1.44E-79	CD4T	DYRK2
TAPSAR12	4.79E-84	0.288977	0.387	0.23	1.57E-79	CD4T	TAPSAR1
ERH1	5.03E-84	0.314961	0.524	0.341	1.65E-79	CD4T	ERH
METTL23	5.77E-84	0.288291	0.233	0.115	1.89E-79	CD4T	METTL23
EZR2	7.32E-84	0.30772	0.449	0.279	2.40E-79	CD4T	EZR
GS1-251I9	9.63E-84	0.260276	0.263	0.134	3.15E-79	CD4T	GS1-251I9.4
G3BP1	1.13E-83	0.31112	0.334	0.19	3.70E-79	CD4T	G3BP1
SRM1	2.95E-83	0.321883	0.317	0.177	9.65E-79	CD4T	SRM
AIP1	3.12E-83	0.289088	0.4	0.242	1.02E-78	CD4T	AIP
TRMT1121	3.52E-83	0.261711	0.618	0.424	1.15E-78	CD4T	TRMT112
PAFAH1B3	1.49E-82	0.284446	0.168	0.072	4.88E-78	CD4T	PAFAH1B3
C6orf482	3.99E-82	0.307899	0.257	0.135	1.31E-77	CD4T	C6orf48

GLS1	9.23E-82	0.289284	0.267	0.139	3.02E-77	CD4T	GLS
BRIX11	2.21E-81	0.277434	0.17	0.074	7.22E-77	CD4T	BRIX1
TMEM173	2.35E-81	0.310922	0.319	0.181	7.68E-77	CD4T	TMEM173
NOL7	2.54E-81	0.251261	0.446	0.281	8.33E-77	CD4T	NOL7
KRT102	8.15E-81	0.307195	0.488	0.315	2.67E-76	CD4T	KRT10
UBQLN21	3.11E-80	0.254432	0.223	0.11	1.02E-75	CD4T	UBQLN2
CACYBP2	4.64E-80	0.273399	0.355	0.209	1.52E-75	CD4T	CACYBP
MPHOSPH	5.39E-80	0.282122	0.511	0.336	1.76E-75	CD4T	MPHOSPH8
RNF167	2.67E-79	0.255449	0.354	0.206	8.72E-75	CD4T	RNF167
RPL72	1.11E-78	0.254347	0.949	0.781	3.64E-74	CD4T	RPL7
UQCRB2	1.48E-78	0.284175	0.867	0.67	4.84E-74	CD4T	UQCRB
CDK6	4.24E-78	0.260703	0.143	0.058	1.39E-73	CD4T	CDK6
HDAC12	7.56E-78	0.263209	0.354	0.209	2.47E-73	CD4T	HDAC1
EIF2S32	8.18E-78	0.28263	0.477	0.311	2.68E-73	CD4T	EIF2S3
LDHA1	2.03E-77	0.275202	0.56	0.377	6.64E-73	CD4T	LDHA
SDAD1	2.44E-77	0.321242	0.267	0.144	7.99E-73	CD4T	SDAD1
CCT22	4.13E-77	0.312553	0.34	0.201	1.35E-72	CD4T	CCT2
DDX181	7.75E-77	0.266054	0.486	0.32	2.54E-72	CD4T	DDX18
ZFAS12	8.26E-77	0.269866	0.671	0.469	2.70E-72	CD4T	ZFAS1
KHDRBS11	1.18E-76	0.263921	0.563	0.385	3.87E-72	CD4T	KHDRBS1
SEC11C	2.65E-76	0.280327	0.311	0.176	8.66E-72	CD4T	SEC11C
BLOC1S4	3.06E-76	0.282073	0.25	0.131	1.00E-71	CD4T	BLOC1S4
KLHDC21	3.41E-76	0.255552	0.192	0.09	1.12E-71	CD4T	KLHDC2
CIB11	2.85E-75	0.253459	0.527	0.354	9.34E-71	CD4T	CIB1
FAM60A1	3.23E-75	0.270702	0.218	0.109	1.06E-70	CD4T	FAM60A
MDH11	9.55E-75	0.330555	0.371	0.225	3.13E-70	CD4T	MDH1
IKZF12	1.63E-74	0.256466	0.501	0.332	5.32E-70	CD4T	IKZF1
ATP5O3	1.71E-74	0.265924	0.682	0.476	5.61E-70	CD4T	ATP5O
TNFRSF14	2.08E-74	0.267441	0.429	0.272	6.80E-70	CD4T	TNFRSF14
AKR1B1	3.43E-74	0.260697	0.258	0.137	1.12E-69	CD4T	AKR1B1
RPS27L1	4.62E-74	0.311648	0.523	0.353	1.51E-69	CD4T	RPS27L
VPS512	7.53E-74	0.261728	0.336	0.199	2.46E-69	CD4T	VPS51
MATR32	8.28E-73	0.255107	0.495	0.329	2.71E-68	CD4T	MATR3
CCNI3	1.25E-72	0.260037	0.872	0.681	4.08E-68	CD4T	CCNI
GBP5	1.46E-72	0.351498	0.284	0.155	4.77E-68	CD4T	GBP5
PPIH1	1.48E-72	0.280958	0.224	0.114	4.83E-68	CD4T	PPIH
KIAA1551	4.80E-72	0.254511	0.454	0.295	1.57E-67	CD4T	KIAA1551
NHP21	1.28E-71	0.284099	0.395	0.249	4.21E-67	CD4T	NHP2
C9orf781	8.40E-71	0.257896	0.434	0.279	2.75E-66	CD4T	C9orf78
PRMT11	9.45E-70	0.293846	0.286	0.162	3.09E-65	CD4T	PRMT1
SNRPF1	9.90E-70	0.275751	0.436	0.282	3.24E-65	CD4T	SNRPF
PCBP22	1.62E-69	0.274762	0.832	0.626	5.31E-65	CD4T	PCBP2
NSMCE11	1.95E-69	0.254929	0.282	0.16	6.38E-65	CD4T	NSMCE1
CEP57	4.77E-69	0.283939	0.277	0.156	1.56E-64	CD4T	CEP57
DEGS11	8.06E-69	0.257045	0.215	0.111	2.64E-64	CD4T	DEGS1
MT-ND61	2.82E-68	0.267769	0.581	0.404	9.22E-64	CD4T	MT-ND6
SESN31	3.13E-68	0.321325	0.194	0.097	1.03E-63	CD4T	SESN3
SYNRG	5.48E-67	0.276016	0.323	0.192	1.80E-62	CD4T	SYNRG
SSBP11	1.84E-66	0.266276	0.457	0.303	6.03E-62	CD4T	SSBP1
EIF3D2	3.24E-66	0.261898	0.473	0.317	1.06E-61	CD4T	EIF3D
MRPL17	5.14E-66	0.253916	0.195	0.098	1.68E-61	CD4T	MRPL17
ILF21	8.25E-66	0.256445	0.385	0.244	2.70E-61	CD4T	ILF2
SNRPA	2.71E-65	0.281336	0.324	0.197	8.87E-61	CD4T	SNRPA
CDK42	1.09E-64	0.254093	0.194	0.098	3.55E-60	CD4T	CDK4
TMED4	1.25E-64	0.254549	0.296	0.175	4.10E-60	CD4T	TMED4
MAD1L1	3.01E-64	0.277294	0.214	0.112	9.86E-60	CD4T	MAD1L1
GIMAP6	3.33E-64	0.288623	0.266	0.15	1.09E-59	CD4T	GIMAP6
IL27RA	1.03E-63	0.281823	0.29	0.169	3.38E-59	CD4T	IL27RA

C14orf166	3.02E-63	0.253246	0.508	0.346	9.87E-59	CD4T	C14orf166
REST	1.17E-62	0.263311	0.333	0.205	3.83E-58	CD4T	REST
ZNHIT31	1.52E-60	0.251755	0.227	0.125	4.96E-56	CD4T	ZNHIT3
TAF9	2.09E-60	0.270971	0.318	0.196	6.86E-56	CD4T	TAF9
DNPH12	4.94E-60	0.264775	0.225	0.125	1.62E-55	CD4T	DNPH1
ARL14EP1	1.72E-59	0.275004	0.227	0.125	5.63E-55	CD4T	ARL14EP
NOB11	8.55E-59	0.253618	0.183	0.094	2.80E-54	CD4T	NOB1
MRPS331	6.22E-58	0.257591	0.231	0.129	2.04E-53	CD4T	MRPS33
DNMT1	3.77E-57	0.25982	0.283	0.17	1.23E-52	CD4T	DNMT1
NUCKS11	7.42E-57	0.262526	0.41	0.275	2.43E-52	CD4T	NUCKS1
MTRNR2L1	6.52E-54	0.812846	0.235	0.136	2.13E-49	CD4T	MTRNR2L12
LPAR61	4.83E-53	0.287953	0.307	0.193	1.58E-48	CD4T	LPAR6
ITGB1	8.38E-53	0.296982	0.538	0.42	2.74E-48	CD4T	ITGB1
CCT8	2.47E-49	0.251835	0.435	0.301	8.07E-45	CD4T	CCT8
GZMK	7.37E-43	0.404099	0.102	0.045	2.41E-38	CD4T	GZMK
RN7SL1	3.06E-28	0.270222	0.219	0.144	1.00E-23	CD4T	RN7SL1
CD8B	0	3.119672	0.979	0.037	0	CD8_Naive	CD8B
CCR73	0	2.402575	0.894	0.088	0	CD8_Naive	CCR7
RP11-291I	0	2.197969	0.609	0.013	0	CD8_Naive	RP11-291B21.2
CD8A	0	2.091365	0.856	0.046	0	CD8_Naive	CD8A
CD3E2	0	2.039234	0.992	0.194	0	CD8_Naive	CD3E
RPS53	0	1.913638	0.997	0.702	0	CD8_Naive	RPS5
LEF12	0	1.886025	0.779	0.078	0	CD8_Naive	LEF1
LDHB2	0	1.783682	0.986	0.382	0	CD8_Naive	LDHB
CD3D2	0	1.740684	0.937	0.159	0	CD8_Naive	CD3D
TCF72	0	1.739598	0.808	0.105	0	CD8_Naive	TCF7
EEF1B23	0	1.684997	0.997	0.669	0	CD8_Naive	EEF1B2
RPS123	0	1.651219	1	0.88	0	CD8_Naive	RPS12
IL7R2	0	1.650874	0.887	0.147	0	CD8_Naive	IL7R
NPM13	0	1.647079	0.99	0.519	0	CD8_Naive	NPM1
C12orf573	0	1.646333	0.936	0.293	0	CD8_Naive	C12orf57
RPS273	0	1.606552	0.999	0.829	0	CD8_Naive	RPS27
TMEM663	0	1.580478	0.992	0.544	0	CD8_Naive	TMEM66
RPS183	0	1.579633	1	0.786	0	CD8_Naive	RPS18
CD272	0	1.551489	0.753	0.091	0	CD8_Naive	CD27
RPL313	0	1.538298	0.999	0.743	0	CD8_Naive	RPL3
RPS63	0	1.536575	1	0.78	0	CD8_Naive	RPS6
RPL10A3	0	1.529721	0.997	0.685	0	CD8_Naive	RPL10A
NOSIP2	0	1.519144	0.867	0.302	0	CD8_Naive	NOSIP
CD3G2	0	1.514713	0.826	0.145	0	CD8_Naive	CD3G
RPS293	0	1.511376	0.997	0.763	0	CD8_Naive	RPS29
RPSA3	0	1.51081	1	0.695	0	CD8_Naive	RPSA
RPS253	0	1.499824	1	0.773	0	CD8_Naive	RPS25
RPS33	0	1.479582	1	0.83	0	CD8_Naive	RPS3
RPL323	0	1.472753	1	0.869	0	CD8_Naive	RPL32
LINC00861	0	1.459994	0.804	0.178	0	CD8_Naive	LINC00861
RPL53	0	1.440045	1	0.798	0	CD8_Naive	RPL5
PCED1B2	0	1.430928	0.622	0.096	0	CD8_Naive	PCED1B
EEF1A14	0	1.428692	1	0.891	0	CD8_Naive	EEF1A1
RPS3A3	0	1.427105	1	0.856	0	CD8_Naive	RPS3A
RPL143	0	1.406208	1	0.809	0	CD8_Naive	RPL14
RPS15A3	0	1.398275	1	0.867	0	CD8_Naive	RPS15A
RPS233	0	1.395869	1	0.823	0	CD8_Naive	RPS23
LTB3	0	1.386397	0.945	0.273	0	CD8_Naive	LTB
CD73	0	1.377543	0.882	0.191	0	CD8_Naive	CD7
RPS4X3	0	1.373607	0.999	0.81	0	CD8_Naive	RPS4X
LBH3	0	1.371635	0.726	0.158	0	CD8_Naive	LBH
PRKCQ-AS	0	1.371555	0.655	0.109	0	CD8_Naive	PRKCQ-AS1

RPL133	0	1.366515	1	0.861	0	CD8_Naive	RPL13
RPS213	0	1.347007	1	0.834	0	CD8_Naive	RPS21
NELL22	0	1.337756	0.493	0.027	0	CD8_Naive	NELL2
RPS27A3	0	1.33681	1	0.866	0	CD8_Naive	RPS27A
RPL103	0	1.31534	1	0.878	0	CD8_Naive	RPL10
OXNAD12	0	1.302429	0.591	0.067	0	CD8_Naive	OXNAD1
RPL303	0	1.258953	1	0.885	0	CD8_Naive	RPL30
CAMK42	0	1.254875	0.602	0.072	0	CD8_Naive	CAMK4
LCK2	0	1.245122	0.825	0.181	0	CD8_Naive	LCK
PTPRCAP3	0	1.215765	0.965	0.266	0	CD8_Naive	PTPRCAP
RPL18A3	0	1.21263	1	0.825	0	CD8_Naive	RPL18A
RPL193	0	1.199924	1	0.868	0	CD8_Naive	RPL19
RPS214	0	1.197095	0.999	0.814	0	CD8_Naive	RPS2
RPS143	0	1.192735	1	0.872	0	CD8_Naive	RPS14
RPL343	0	1.178371	1	0.872	0	CD8_Naive	RPL34
TRABD2A2	0	1.161277	0.505	0.048	0	CD8_Naive	TRABD2A
PIK3IP12	0	1.155964	0.575	0.102	0	CD8_Naive	PIK3IP1
RPL293	0	1.152	0.999	0.833	0	CD8_Naive	RPL29
RPL373	0	1.136594	1	0.866	0	CD8_Naive	RPL37
PASK2	0	1.136064	0.429	0.031	0	CD8_Naive	PASK
RPS83	0	1.135522	1	0.877	0	CD8_Naive	RPS8
FLT3LG2	0	1.127964	0.625	0.134	0	CD8_Naive	FLT3LG
ABLIM13	0	1.124294	0.574	0.086	0	CD8_Naive	ABLIM1
RPL35A3	0	1.119469	0.999	0.87	0	CD8_Naive	RPL35A
MAL2	0	1.109848	0.515	0.066	0	CD8_Naive	MAL
RPL113	0	1.10632	1	0.882	0	CD8_Naive	RPL11
RPL183	0	1.105452	1	0.849	0	CD8_Naive	RPL18
LRRN31	0	1.093433	0.377	0.014	0	CD8_Naive	LRRN3
RPL123	0	1.067362	1	0.867	0	CD8_Naive	RPL12
SPOCK22	0	1.059834	0.61	0.116	0	CD8_Naive	SPOCK2
RHOH3	0	1.043082	0.564	0.102	0	CD8_Naive	RHOH
RPS283	0	1.039217	1	0.899	0	CD8_Naive	RPS28
RPS153	0	1.016075	1	0.865	0	CD8_Naive	RPS15
RGCC2	0	0.983356	0.523	0.087	0	CD8_Naive	RGCC
SH3YL12	0	0.956491	0.449	0.072	0	CD8_Naive	SH3YL1
LDLRAP12	0	0.937916	0.535	0.087	0	CD8_Naive	LDLRAP1
THEMIS3	0	0.891931	0.39	0.038	0	CD8_Naive	THEMIS
IL322	0	0.862712	0.943	0.205	0	CD8_Naive	IL32
BACH22	0	0.711979	0.292	0.029	0	CD8_Naive	BACH2
PCSK1N2	0	0.707196	0.268	0.022	0	CD8_Naive	PCSK1N
C14orf642	0	0.667577	0.293	0.031	0	CD8_Naive	C14orf64
EPHX22	0	0.659457	0.277	0.028	0	CD8_Naive	EPHX2
CD248	0	0.602807	0.215	0.002	0	CD8_Naive	CD248
NOG1	0	0.57756	0.208	0.011	0	CD8_Naive	NOG
DSEL	0	0.349417	0.125	0.004	0	CD8_Naive	DSEL
RP11-641A	0	0.31398	0.111	0.004	0	CD8_Naive	RP11-641A6.2
RPL363	#######	1.099145	0.999	0.829	#######	CD8_Naive	RPL36
RPS194	#######	1.2124	1	0.813	#######	CD8_Naive	RPS19
RPL7A3	#######	1.051809	1	0.837	#######	CD8_Naive	RPL7A
RPL283	#######	0.90321	1	0.896	#######	CD8_Naive	RPL28
IFITM12	#######	1.196671	0.99	0.327	#######	CD8_Naive	IFITM1
EEF1G3	#######	1.585253	0.943	0.518	#######	CD8_Naive	EEF1G
CLEC2D3	#######	0.876827	0.467	0.084	#######	CD8_Naive	CLEC2D
SIRPG2	#######	0.643276	0.292	0.034	#######	CD8_Naive	SIRPG
NUCB21	#######	1.098785	0.636	0.158	#######	CD8_Naive	NUCB2
MT-ND4L3	#######	1.287168	1	0.772	#######	CD8_Naive	MT-ND4L
PDE3B2	#######	0.884203	0.384	0.06	#######	CD8_Naive	PDE3B
RPLP03	#######	1.220241	1	0.775	#######	CD8_Naive	RPLP0

RPS103	#######	1.298109	0.982	0.761	#######	CD8_Naive	RPS10
TMEM204	#######	0.706906	0.335	0.046	#######	CD8_Naive	TMEM204
RPS163	#######	1.143142	0.999	0.763	#######	CD8_Naive	RPS16
RPL23A3	#######	1.163009	0.997	0.737	#######	CD8_Naive	RPL23A
BCL11B2	#######	0.799619	0.38	0.061	#######	CD8_Naive	BCL11B
NT5E1	#######	0.297065	0.118	0.006	#######	CD8_Naive	NT5E
ID33	#######	0.707879	0.313	0.043	#######	CD8_Naive	ID3
RPL213	#######	1.039871	0.999	0.813	#######	CD8_Naive	RPL21
GNB2L13	#######	1.006017	1	0.797	#######	CD8_Naive	GNB2L1
FAIM33	#######	0.95387	0.577	0.13	#######	CD8_Naive	FAIM3
RPL223	#######	0.943351	0.999	0.838	#######	CD8_Naive	RPL22
MYC3	#######	1.0532	0.463	0.094	#######	CD8_Naive	MYC
CD23	#######	0.906287	0.681	0.172	#######	CD8_Naive	CD2
RPL93	#######	1.028695	0.999	0.8	#######	CD8_Naive	RPL9
ITK2	#######	0.782232	0.409	0.072	#######	CD8_Naive	ITK
RPS133	#######	0.864489	1	0.887	#######	CD8_Naive	RPS13
ETS13	#######	1.02125	0.685	0.183	#######	CD8_Naive	ETS1
RPL383	#######	1.136201	0.988	0.724	#######	CD8_Naive	RPL38
APBB12	#######	0.615965	0.266	0.033	#######	CD8_Naive	APBB1
OCIAD23	#######	0.832467	0.486	0.1	#######	CD8_Naive	OCIAD2
RPS73	#######	0.889912	1	0.863	#######	CD8_Naive	RPS7
RPS263	#######	1.540265	0.995	0.827	#######	CD8_Naive	RPS26
GYPC2	#######	1.111628	0.766	0.249	#######	CD8_Naive	GYPC
RPL173	#######	1.223139	0.982	0.712	#######	CD8_Naive	RPL17
SNHG84	#######	1.259999	0.781	0.287	#######	CD8_Naive	SNHG8
SCML42	#######	0.746881	0.403	0.072	#######	CD8_Naive	SCML4
RPL63	#######	0.868549	1	0.863	#######	CD8_Naive	RPL6
BTG13	#######	1.228703	0.996	0.679	#######	CD8_Naive	BTG1
LEPROTL1	#######	1.102962	0.73	0.245	#######	CD8_Naive	LEPROTL1
RCAN32	#######	0.942251	0.484	0.108	#######	CD8_Naive	RCAN3
NACA4	#######	0.919401	1	0.818	#######	CD8_Naive	NACA
EVL3	#######	1.028069	0.81	0.283	#######	CD8_Naive	EVL
TPT12	#######	0.808926	1	0.911	#######	CD8_Naive	TPT1
AC013264	#######	0.401471	0.16	0.013	#######	CD8_Naive	AC013264.2
TESPA12	#######	0.729476	0.36	0.065	#######	CD8_Naive	TESPA1
AES3	#######	1.073128	0.925	0.413	#######	CD8_Naive	AES
DENND2D	#######	0.89392	0.642	0.183	#######	CD8_Naive	DENND2D
RPL44	#######	1.049381	0.994	0.72	#######	CD8_Naive	RPL4
ITGA62	#######	0.65463	0.336	0.057	#######	CD8_Naive	ITGA6
CTSW	#######	0.260279	0.713	0.185	#######	CD8_Naive	CTSW
RPL264	#######	0.942261	0.997	0.804	#######	CD8_Naive	RPL26
RPL243	#######	0.883462	1	0.798	#######	CD8_Naive	RPL24
MT-ATP84	#######	1.272177	0.981	0.734	#######	CD8_Naive	MT-ATP8
STK17A3	#######	0.916939	0.699	0.219	#######	CD8_Naive	STK17A
GIMAP52	#######	1.058372	0.759	0.269	#######	CD8_Naive	GIMAP5
GLTSCR23	#######	1.100016	0.915	0.451	#######	CD8_Naive	GLTSCR2
RPL393	#######	0.704386	1	0.914	#######	CD8_Naive	RPL39
RP11-18H	#######	0.561137	0.26	0.038	#######	CD8_Naive	RP11-18H21.1
RPLP23	#######	0.71872	1	0.876	#######	CD8_Naive	RPLP2
HSPA84	#######	1.048315	0.983	0.571	#######	CD8_Naive	HSPA8
HIST1H4C	#######	1.081635	0.931	0.477	#######	CD8_Naive	HIST1H4C
APBA21	#######	0.445839	0.198	0.023	#######	CD8_Naive	APBA2
MT-CYB3	#######	0.800184	1	0.93	#######	CD8_Naive	MT-CYB
RIC33	#######	0.537604	0.223	0.029	#######	CD8_Naive	RIC3
HNRNPA1	#######	1.017259	0.977	0.625	#######	CD8_Naive	HNRNPA1
STMN13	#######	0.739383	0.346	0.069	#######	CD8_Naive	STMN1
RPL84	#######	0.76388	1	0.856	#######	CD8_Naive	RPL8
CD54	#######	0.574984	0.293	0.05	#######	CD8_Naive	CD5

STMN32	#######	0.577185	0.265	0.043	#######	CD8_Naive	STMN3
GZMM	#######	0.675854	0.529	0.134	#######	CD8_Naive	GZMM
S100B	#######	0.899996	0.157	0.016	#######	CD8_Naive	S100B
13-Sep	#######	0.796647	0.515	0.138	#######	CD8_Naive	1-Sep
RPL37A3	#######	0.807947	0.995	0.806	#######	CD8_Naive	RPL37A
RPL413	#######	0.757745	1	0.883	#######	CD8_Naive	RPL41
ATP6V0E2	#######	0.616409	0.306	0.058	#######	CD8_Naive	ATP6V0E2
TXK2	#######	0.619202	0.344	0.068	#######	CD8_Naive	TXK
FAM134B2	#######	0.540259	0.234	0.035	#######	CD8_Naive	FAM134B
SNRPN2	#######	0.594796	0.408	0.094	#######	CD8_Naive	SNRPN
AQP32	#######	0.644976	0.355	0.073	#######	CD8_Naive	AQP3
SATB12	#######	0.772461	0.45	0.118	#######	CD8_Naive	SATB1
HSP90AB1	#######	0.967935	0.951	0.547	#######	CD8_Naive	HSP90AB1
RPL153	#######	0.790618	0.999	0.799	#######	CD8_Naive	RPL15
RPLP13	#######	0.650935	1	0.944	#######	CD8_Naive	RPLP1
CRTAM	#######	0.476082	0.229	0.035	#######	CD8_Naive	CRTAM
GIMAP72	#######	0.977399	0.956	0.568	#######	CD8_Naive	GIMAP7
THEM42	#######	0.653149	0.35	0.077	#######	CD8_Naive	THEM4
EEF1D3	#######	0.810683	0.996	0.785	#######	CD8_Naive	EEF1D
ISG203	#######	0.750657	0.568	0.174	#######	CD8_Naive	ISG20
ANXA2R2	#######	0.66361	0.372	0.085	#######	CD8_Naive	ANXA2R
BEX23	#######	0.556822	0.266	0.047	#######	CD8_Naive	BEX2
RPL353	#######	0.80646	0.996	0.768	#######	CD8_Naive	RPL35
LY93	#######	0.610385	0.31	0.062	#######	CD8_Naive	LY9
RPS93	#######	0.729069	0.997	0.828	#######	CD8_Naive	RPS9
ORMDL32	#######	0.598299	0.313	0.064	#######	CD8_Naive	ORMDL3
RPL314	#######	0.818698	0.991	0.732	#######	CD8_Naive	RPL31
PRMT22	#######	0.867614	0.806	0.384	#######	CD8_Naive	PRMT2
CXCR43	#######	0.720163	0.525	0.163	#######	CD8_Naive	CXCR4
CD962	#######	0.580947	0.385	0.094	#######	CD8_Naive	CD96
TC2N2	#######	0.681973	0.425	0.111	#######	CD8_Naive	TC2N
CARS	#######	0.734588	0.378	0.098	#######	CD8_Naive	CARS
RPS4Y13	#######	1.261372	0.767	0.461	#######	CD8_Naive	RPS4Y1
PLEKHB12	#######	0.363949	0.148	0.018	#######	CD8_Naive	PLEKHB1
TSTD13	#######	0.694064	0.52	0.167	#######	CD8_Naive	TSTD1
FAM102A2	#######	0.435958	0.201	0.032	#######	CD8_Naive	FAM102A
SKAP12	#######	0.630867	0.48	0.139	#######	CD8_Naive	SKAP1
SLC38A13	#######	0.650846	0.42	0.117	#######	CD8_Naive	SLC38A1
EIF3E3	#######	0.881564	0.876	0.486	#######	CD8_Naive	EIF3E
LAT3	#######	0.400137	0.553	0.173	#######	CD8_Naive	LAT
ACAP13	#######	0.775052	0.644	0.249	#######	CD8_Naive	ACAP1
RPS203	#######	0.852947	0.933	0.587	#######	CD8_Naive	RPS20
LIME12	#######	0.637162	0.377	0.096	#######	CD8_Naive	LIME1
IL2RG3	#######	0.586053	0.813	0.326	#######	CD8_Naive	IL2RG
AC006129	#######	0.665215	0.366	0.093	#######	CD8_Naive	AC006129.2
RPL13A3	#######	0.748325	0.995	0.764	#######	CD8_Naive	RPL13A
SFXN12	#######	0.580348	0.326	0.078	#######	CD8_Naive	SFXN1
CD62	#######	0.533299	0.297	0.065	#######	CD8_Naive	CD6
LINC00649	#######	0.449753	0.219	0.038	#######	CD8_Naive	LINC00649
RUNX2	#######	0.481565	0.215	0.038	#######	CD8_Naive	RUNX2
BTF33	#######	0.72782	0.995	0.739	#######	CD8_Naive	BTF3
CYTIP3	#######	0.818975	0.645	0.262	#######	CD8_Naive	CYTIP
PLCG12	#######	0.427843	0.206	0.035	#######	CD8_Naive	PLCG1
UXT3	#######	0.793213	0.835	0.428	#######	CD8_Naive	UXT
PEBP13	#######	0.798149	0.609	0.24	#######	CD8_Naive	PEBP1
TMIGD21	#######	0.439371	0.225	0.041	#######	CD8_Naive	TMIGD2
EEF23	#######	0.765741	0.99	0.695	#######	CD8_Naive	EEF2
RPL36A4	#######	0.814482	0.958	0.629	#######	CD8_Naive	RPL36A

MALAT13	#######	0.710692	1	0.943	#######	CD8_Naive	MALAT1
TRAF3IP33	#######	0.785063	0.824	0.425	#######	CD8_Naive	TRAF3IP3
RPSAP583	#######	0.71648	0.417	0.129	#######	CD8_Naive	RPSAP58
UBASH3A2	#######	0.366521	0.169	0.025	#######	CD8_Naive	UBASH3A
KLF24	#######	0.762169	0.942	0.524	#######	CD8_Naive	KLF2
RPL273	#######	0.729744	0.99	0.734	#######	CD8_Naive	RPL27
DGKA2	#######	0.603838	0.387	0.113	#######	CD8_Naive	DGKA
SUSD32	#######	0.49663	0.295	0.07	#######	CD8_Naive	SUSD3
COX7C4	#######	0.735915	0.991	0.702	#######	CD8_Naive	COX7C
MT-ND32	#######	0.592936	1	0.906	#######	CD8_Naive	MT-ND3
PIM23	#######	0.5759	0.311	0.077	#######	CD8_Naive	PIM2
HINT13	#######	0.730168	0.961	0.606	#######	CD8_Naive	HINT1
AKTIP2	#######	0.427721	0.216	0.041	#######	CD8_Naive	AKTIP
HSPE13	#######	0.726394	0.596	0.243	#######	CD8_Naive	HSPE1
SUPT3H2	#######	0.482731	0.22	0.043	#######	CD8_Naive	SUPT3H
FBL3	#######	0.750609	0.642	0.282	#######	CD8_Naive	FBL
CCDC109B	#######	0.696485	0.649	0.283	#######	CD8_Naive	CCDC109B
MDS21	#######	0.318078	0.112	0.013	#######	CD8_Naive	MDS2
BIN13	#######	0.61964	0.459	0.15	#######	CD8_Naive	BIN1
LSR1	#######	0.332854	0.156	0.024	#######	CD8_Naive	LSR
SELM2	#######	0.471674	0.254	0.057	#######	CD8_Naive	SELM
LTA1	#######	0.307483	0.136	0.019	#######	CD8_Naive	LTA
HSPB13	#######	0.612511	0.548	0.214	#######	CD8_Naive	HSPB1
TMEM2433	#######	0.609435	0.467	0.165	#######	CD8_Naive	TMEM243
OLFM21	#######	0.307973	0.127	0.017	#######	CD8_Naive	OLFM2
FBLN52	#######	0.397749	0.16	0.025	#######	CD8_Naive	FBLN5
RBL22	#######	0.633923	0.606	0.252	#######	CD8_Naive	RBL2
PRKCA2	#######	0.451001	0.27	0.066	#######	CD8_Naive	PRKCA
Sep-63	#######	0.640293	0.677	0.306	#######	CD8_Naive	6-Sep
ITM2A2	#######	0.504079	0.377	0.11	#######	CD8_Naive	ITM2A
BCAS42	#######	0.353031	0.161	0.026	#######	CD8_Naive	BCAS4
SIT13	#######	0.491628	0.296	0.075	#######	CD8_Naive	SIT1
CD2472	#######	0.255531	0.511	0.168	#######	CD8_Naive	CD247
USP531	#######	0.351557	0.142	0.021	#######	CD8_Naive	USP53
ZAP702	#######	0.491827	0.396	0.12	#######	CD8_Naive	ZAP70
IMPDH23	#######	0.639667	0.389	0.13	#######	CD8_Naive	IMPDH2
PRDX22	#######	0.671854	0.539	0.217	#######	CD8_Naive	PRDX2
SSR23	#######	0.706628	0.849	0.48	#######	CD8_Naive	SSR2
GCC22	#######	0.661388	0.55	0.224	1.48E-99	CD8_Naive	GCC2
TOMM73	#######	0.65484	0.977	0.698	7.80E-99	CD8_Naive	TOMM7
MZT2B3	#######	0.668704	0.847	0.482	1.72E-98	CD8_Naive	MZT2B
HMGN13	#######	0.545963	0.744	0.354	1.92E-98	CD8_Naive	HMGN1
CTB-133G	#######	0.631036	0.438	0.154	3.55E-98	CD8_Naive	CTB-133G6.1
PRKCH2	#######	0.522587	0.368	0.111	4.57E-98	CD8_Naive	PRKCH
NDFIP12	#######	0.705468	0.673	0.333	5.13E-98	CD8_Naive	NDFIP1
FOXP13	#######	0.673132	0.577	0.248	1.65E-97	CD8_Naive	FOXP1
SERINC52	#######	0.480953	0.243	0.058	2.08E-97	CD8_Naive	SERINC5
PSIP13	#######	0.623467	0.548	0.224	6.99E-97	CD8_Naive	PSIP1
FAU3	#######	0.442589	1	0.902	9.97E-97	CD8_Naive	FAU
SOD14	5.06E-99	0.61276	0.755	0.388	1.66E-94	CD8_Naive	SOD1
SNRPD23	3.26E-98	0.630485	0.925	0.571	1.07E-93	CD8_Naive	SNRPD2
ARHGAP15	3.54E-98	0.561364	0.519	0.202	1.16E-93	CD8_Naive	ARHGAP15
SELL3	3.95E-98	0.617133	0.847	0.457	1.29E-93	CD8_Naive	SELL
USP111	1.72E-95	0.472348	0.259	0.067	5.64E-91	CD8_Naive	USP11
EIF3H3	7.22E-95	0.675585	0.873	0.524	2.36E-90	CD8_Naive	EIF3H
UQCRB3	1.13E-94	0.640711	0.963	0.679	3.69E-90	CD8_Naive	UQCRB
MLLT32	3.02E-94	0.369352	0.223	0.051	9.88E-90	CD8_Naive	MLLT3
PDE7A3	7.32E-94	0.545791	0.305	0.09	2.40E-89	CD8_Naive	PDE7A

DDIT42	1.36E-93	0.517065	0.336	0.101	4.46E-89	CD8_Naive	DDIT4
EIF4A23	5.84E-93	0.654632	0.631	0.301	1.91E-88	CD8_Naive	EIF4A2
PITPNA-A	8.11E-93	0.429212	0.224	0.053	2.65E-88	CD8_Naive	PITPNA-AS1
FBXO321	9.25E-93	0.400038	0.161	0.03	3.03E-88	CD8_Naive	FBXO32
TBC1D10C	4.48E-92	0.575092	0.516	0.209	1.47E-87	CD8_Naive	TBC1D10C
C12orf653	1.95E-91	0.505588	0.3	0.087	6.37E-87	CD8_Naive	C12orf65
SNHG73	6.00E-90	0.584656	0.497	0.206	1.96E-85	CD8_Naive	SNHG7
MZT2A3	1.23E-87	0.562685	0.467	0.187	4.01E-83	CD8_Naive	MZT2A
AK51	1.75E-87	0.274164	0.125	0.02	5.73E-83	CD8_Naive	AK5
ST133	1.17E-86	0.602287	0.766	0.43	3.82E-82	CD8_Naive	ST13
GOLGA8B2	1.09E-85	0.378295	0.205	0.048	3.57E-81	CD8_Naive	GOLGA8B
PPA12	1.33E-85	0.612525	0.553	0.251	4.35E-81	CD8_Naive	PPA1
RARRES32	1.41E-85	0.476116	0.73	0.359	4.62E-81	CD8_Naive	RARRES3
RPS242	3.93E-85	0.456755	1	0.882	1.29E-80	CD8_Naive	RPS24
CDC25B2	1.30E-84	0.467988	0.286	0.084	4.25E-80	CD8_Naive	CDC25B
SLFN52	2.75E-84	0.528078	0.463	0.182	9.02E-80	CD8_Naive	SLFN5
BCL23	3.77E-84	0.467442	0.227	0.058	1.24E-79	CD8_Naive	BCL2
IL163	3.28E-82	0.58679	0.555	0.254	1.07E-77	CD8_Naive	IL16
HSPD13	5.72E-82	0.610895	0.566	0.266	1.87E-77	CD8_Naive	HSPD1
TCEA32	4.52E-81	0.278862	0.118	0.019	1.48E-76	CD8_Naive	TCEA3
RAC23	6.91E-81	0.548955	0.903	0.576	2.26E-76	CD8_Naive	RAC2
RPL36AL2	1.67E-80	0.552129	0.983	0.719	5.46E-76	CD8_Naive	RPL36AL
HSF2	3.49E-79	0.282646	0.135	0.025	1.14E-74	CD8_Naive	HSF2
RASGRP12	8.32E-79	0.389676	0.206	0.051	2.72E-74	CD8_Naive	RASGRP1
EIF3F3	2.77E-77	0.584639	0.88	0.538	9.07E-73	CD8_Naive	EIF3F
STAT42	2.85E-77	0.392655	0.256	0.073	9.34E-73	CD8_Naive	STAT4
RNF144A1	2.75E-75	0.270527	0.126	0.023	8.99E-71	CD8_Naive	RNF144A
ATM1	2.95E-74	0.581357	0.561	0.271	9.66E-70	CD8_Naive	ATM
C12orf232	5.58E-74	0.339045	0.171	0.039	1.83E-69	CD8_Naive	C12orf23
FAM162A3	1.34E-73	0.492768	0.366	0.139	4.38E-69	CD8_Naive	FAM162A
RAN3	1.06E-72	0.547596	0.773	0.45	3.48E-68	CD8_Naive	RAN
GIMAP12	3.64E-72	0.565834	0.727	0.407	1.19E-67	CD8_Naive	GIMAP1
SNORA761	4.94E-71	0.455734	0.247	0.075	1.62E-66	CD8_Naive	SNORA76
CISH2	5.63E-71	0.582176	0.259	0.08	1.84E-66	CD8_Naive	CISH
S1PR13	1.09E-70	0.412764	0.323	0.109	3.58E-66	CD8_Naive	S1PR1
PRKACB1	2.40E-70	0.387653	0.38	0.145	7.84E-66	CD8_Naive	PRKACB
C11orf11	3.41E-70	0.352665	0.167	0.04	1.12E-65	CD8_Naive	C11orf1
MIF3	1.25E-68	0.485092	0.902	0.566	4.09E-64	CD8_Naive	MIF
HIST1H1D	1.62E-68	0.454276	0.35	0.131	5.32E-64	CD8_Naive	HIST1H1D
LIMD24	2.26E-68	0.523587	0.914	0.587	7.39E-64	CD8_Naive	LIMD2
BNIP32	2.57E-68	0.353927	0.199	0.054	8.43E-64	CD8_Naive	BNIP3
EIF3L3	3.69E-68	0.49903	0.858	0.537	1.21E-63	CD8_Naive	EIF3L
UBE2D23	1.87E-67	0.459066	0.734	0.418	6.13E-63	CD8_Naive	UBE2D2
U2AF1L41	3.82E-67	0.381098	0.233	0.07	1.25E-62	CD8_Naive	U2AF1L4
THOC32	4.41E-67	0.443182	0.281	0.095	1.44E-62	CD8_Naive	THOC3
MGAT4A2	1.62E-66	0.429587	0.314	0.112	5.32E-62	CD8_Naive	MGAT4A
TECR2	2.60E-66	0.46464	0.488	0.226	8.50E-62	CD8_Naive	TECR
GIMAP42	3.60E-66	0.52814	0.839	0.508	1.18E-61	CD8_Naive	GIMAP4
C1QBP3	4.75E-66	0.578102	0.551	0.285	1.56E-61	CD8_Naive	C1QBP
COMMD62	1.51E-65	0.444563	0.973	0.697	4.95E-61	CD8_Naive	COMMD6
NAA16	3.76E-65	0.350592	0.233	0.071	1.23E-60	CD8_Naive	NAA16
HMOX22	2.56E-64	0.444216	0.411	0.173	8.39E-60	CD8_Naive	HMOX2
CNN23	6.93E-64	0.499633	0.677	0.383	2.27E-59	CD8_Naive	CNN2
ENOSF12	8.02E-64	0.310981	0.166	0.041	2.63E-59	CD8_Naive	ENOSF1
ZNF8622	1.37E-63	0.378169	0.246	0.079	4.49E-59	CD8_Naive	ZNF862
NAP1L41	3.20E-63	0.423806	0.534	0.263	1.05E-58	CD8_Naive	NAP1L4
ATP5O4	3.61E-63	0.456217	0.807	0.486	1.18E-58	CD8_Naive	ATP5O
PRR41	4.41E-63	0.374477	0.225	0.069	1.44E-58	CD8_Naive	PRR4

CCT33	1.16E-62	0.519935	0.495	0.242	3.81E-58	CD8_Naive	CCT3
CHMP73	1.28E-62	0.405	0.279	0.098	4.19E-58	CD8_Naive	CHMP7
C6orf483	1.52E-62	0.518104	0.342	0.14	4.99E-58	CD8_Naive	C6orf48
LSM52	3.67E-62	0.509759	0.449	0.21	1.20E-57	CD8_Naive	LSM5
CD282	4.47E-62	0.305011	0.154	0.037	1.46E-57	CD8_Naive	CD28
RSL1D13	4.84E-62	0.519068	0.655	0.37	1.59E-57	CD8_Naive	RSL1D1
ZFAS13	5.75E-62	0.492555	0.784	0.478	1.88E-57	CD8_Naive	ZFAS1
ATF7IP22	6.39E-62	0.322102	0.167	0.043	2.09E-57	CD8_Naive	ATF7IP2
C1orf2281	9.81E-62	0.391415	0.255	0.085	3.21E-57	CD8_Naive	C1orf228
CCT23	1.43E-61	0.466909	0.445	0.206	4.68E-57	CD8_Naive	CCT2
FUS3	9.12E-61	0.490471	0.725	0.424	2.98E-56	CD8_Naive	FUS
CCT43	2.56E-60	0.480057	0.53	0.27	8.37E-56	CD8_Naive	CCT4
IGSF8	5.60E-60	0.351828	0.181	0.05	1.83E-55	CD8_Naive	IGSF8
SMDT13	1.37E-59	0.460764	0.708	0.409	4.48E-55	CD8_Naive	SMDT1
FHIT1	1.43E-59	0.283913	0.142	0.033	4.67E-55	CD8_Naive	FHIT
PRR5	2.62E-59	0.28086	0.156	0.038	8.56E-55	CD8_Naive	PRR5
PPP1R22	3.98E-59	0.476129	0.533	0.271	1.30E-54	CD8_Naive	PPP1R2
APEX13	2.74E-58	0.451231	0.506	0.25	8.98E-54	CD8_Naive	APEX1
EPB41L4A-	1.77E-57	0.393933	0.256	0.09	5.79E-53	CD8_Naive	EPB41L4A-AS1
EIF4B3	2.51E-57	0.493347	0.737	0.443	8.23E-53	CD8_Naive	EIF4B
RRP1B2	3.08E-57	0.39003	0.288	0.108	1.01E-52	CD8_Naive	RRP1B
RASA21	7.68E-57	0.387881	0.331	0.132	2.51E-52	CD8_Naive	RASA2
NSA22	1.19E-56	0.449439	0.775	0.476	3.88E-52	CD8_Naive	NSA2
CIRBP4	1.44E-56	0.447917	0.748	0.444	4.71E-52	CD8_Naive	CIRBP
DDX242	1.47E-56	0.403514	0.654	0.363	4.81E-52	CD8_Naive	DDX24
THYN11	2.24E-56	0.37356	0.36	0.153	7.34E-52	CD8_Naive	THYN1
CYFIP24	2.71E-56	0.417809	0.389	0.168	8.86E-52	CD8_Naive	CYFIP2
AC092580	1.13E-55	0.375731	0.169	0.046	3.68E-51	CD8_Naive	AC092580.4
ST3GAL12	1.77E-55	0.392745	0.341	0.14	5.81E-51	CD8_Naive	ST3GAL1
CUTA2	1.52E-54	0.396736	0.673	0.382	4.97E-50	CD8_Naive	CUTA
ALKBH71	1.73E-54	0.48023	0.569	0.314	5.68E-50	CD8_Naive	ALKBH7
PABPC12	6.95E-54	0.378307	0.999	0.835	2.28E-49	CD8_Naive	PABPC1
TMEM106I	4.42E-53	0.35799	0.241	0.084	1.45E-48	CD8_Naive	TMEM106B
NIPAL32	4.43E-53	0.304447	0.167	0.047	1.45E-48	CD8_Naive	NIPAL3
SPNS3	4.83E-53	0.292453	0.152	0.04	1.58E-48	CD8_Naive	SPNS3
PPM1K3	6.15E-53	0.408244	0.287	0.111	2.01E-48	CD8_Naive	PPM1K
AL592284.	9.54E-53	0.333413	0.227	0.077	3.12E-48	CD8_Naive	AL592284.1
BEX41	1.63E-52	0.298625	0.248	0.088	5.34E-48	CD8_Naive	BEX4
ADPRM1	2.01E-52	0.268615	0.12	0.027	6.59E-48	CD8_Naive	ADPRM
PDCD4-AS	2.53E-52	0.265283	0.134	0.033	8.28E-48	CD8_Naive	PDCD4-AS1
TMEM123	3.51E-52	0.438295	0.595	0.336	1.15E-47	CD8_Naive	TMEM123
CD524	1.57E-51	0.383395	0.978	0.646	5.15E-47	CD8_Naive	CD52
DNAJB12	2.93E-51	0.407659	0.319	0.133	9.59E-47	CD8_Naive	DNAJB1
RPL73	7.69E-51	0.36926	0.992	0.79	2.52E-46	CD8_Naive	RPL7
CRLF31	1.58E-49	0.358941	0.377	0.173	5.18E-45	CD8_Naive	CRLF3
ZNF221	4.24E-49	0.395379	0.344	0.151	1.39E-44	CD8_Naive	ZNF22
ASF1A2	9.41E-48	0.35471	0.239	0.089	3.08E-43	CD8_Naive	ASF1A
SUN22	2.01E-47	0.360481	0.525	0.281	6.59E-43	CD8_Naive	SUN2
TBCC1	2.32E-47	0.359235	0.269	0.106	7.59E-43	CD8_Naive	TBCC
PHF13	2.51E-47	0.306106	0.208	0.071	8.22E-43	CD8_Naive	PHF1
TNIK2	2.76E-47	0.257028	0.206	0.068	9.02E-43	CD8_Naive	TNIK
URI13	3.55E-47	0.396429	0.399	0.193	1.16E-42	CD8_Naive	URI1
CLNS1A2	7.52E-47	0.390292	0.488	0.259	2.46E-42	CD8_Naive	CLNS1A
RBM42	9.95E-47	0.380975	0.391	0.189	3.26E-42	CD8_Naive	RBM4
RP11-796I	1.02E-46	0.344109	0.199	0.068	3.33E-42	CD8_Naive	RP11-796E2.4
C19orf532	3.22E-46	0.405954	0.681	0.419	1.06E-41	CD8_Naive	C19orf53
ZNF4281	5.52E-46	0.348678	0.375	0.174	1.81E-41	CD8_Naive	ZNF428
MT-CO31	6.52E-46	0.252586	1	0.969	2.14E-41	CD8_Naive	MT-CO3

PNISR2	9.72E-46	0.368256	0.682	0.421	3.18E-41	CD8_Naive	PNISR
NPAT1	1.23E-45	0.333442	0.229	0.085	4.03E-41	CD8_Naive	NPAT
TUBB4	3.28E-45	0.2986	0.65	0.379	1.07E-40	CD8_Naive	TUBB
MAPKAPK	6.43E-45	0.322737	0.265	0.106	2.10E-40	CD8_Naive	MAPKAPK5-AS1
SMPD1	6.58E-45	0.260761	0.203	0.07	2.15E-40	CD8_Naive	SMPD1
NDUFAF42	1.37E-44	0.32334	0.225	0.084	4.49E-40	CD8_Naive	NDUFAF4
TNRC6C	1.65E-44	0.276566	0.144	0.041	5.40E-40	CD8_Naive	TNRC6C
MAT2B3	1.78E-44	0.379275	0.515	0.284	5.83E-40	CD8_Naive	MAT2B
IMP33	1.82E-44	0.356184	0.507	0.273	5.95E-40	CD8_Naive	IMP3
LINS2	2.03E-44	0.28221	0.181	0.06	6.64E-40	CD8_Naive	LINS
AAK12	2.19E-44	0.351732	0.489	0.26	7.18E-40	CD8_Naive	AAK1
COX6C3	3.78E-44	0.367955	0.867	0.577	1.24E-39	CD8_Naive	COX6C
NCL3	5.45E-44	0.381533	0.772	0.495	1.78E-39	CD8_Naive	NCL
ILF3-AS11	5.97E-44	0.35585	0.282	0.118	1.96E-39	CD8_Naive	ILF3-AS1
CCT73	2.21E-43	0.398565	0.441	0.233	7.25E-39	CD8_Naive	CCT7
FOXO12	8.29E-43	0.297122	0.201	0.071	2.71E-38	CD8_Naive	FOXO1
PDCD42	1.07E-42	0.347648	0.439	0.225	3.51E-38	CD8_Naive	PDCD4
IL6ST1	1.51E-42	0.267265	0.338	0.154	4.95E-38	CD8_Naive	IL6ST
TMC82	6.42E-42	0.349804	0.335	0.154	2.10E-37	CD8_Naive	TMC8
PAICS	1.54E-41	0.258652	0.142	0.042	5.03E-37	CD8_Naive	PAICS
RDH14	1.72E-41	0.29263	0.197	0.07	5.63E-37	CD8_Naive	RDH14
TFB1M	2.23E-41	0.297374	0.172	0.058	7.31E-37	CD8_Naive	TFB1M
ATIC3	3.14E-41	0.352459	0.234	0.093	1.03E-36	CD8_Naive	ATIC
DYRK22	8.62E-41	0.322524	0.192	0.068	2.82E-36	CD8_Naive	DYRK2
ZNF1011	2.07E-40	0.307787	0.167	0.056	6.79E-36	CD8_Naive	ZNF101
UBAC21	2.08E-40	0.285306	0.363	0.176	6.82E-36	CD8_Naive	UBAC2
AKR1B11	2.41E-40	0.331226	0.313	0.142	7.90E-36	CD8_Naive	AKR1B1
UBXN15	2.70E-40	0.361808	0.723	0.457	8.83E-36	CD8_Naive	UBXN1
NGRN3	5.19E-40	0.318047	0.215	0.082	1.70E-35	CD8_Naive	NGRN
PHB23	1.00E-39	0.415819	0.569	0.346	3.29E-35	CD8_Naive	PHB2
ZFAND11	2.34E-39	0.290359	0.25	0.104	7.66E-35	CD8_Naive	ZFAND1
FARSB	3.48E-39	0.258114	0.17	0.058	1.14E-34	CD8_Naive	FARSB
KRT103	4.49E-39	0.346499	0.553	0.324	1.47E-34	CD8_Naive	KRT10
EIF2A1	1.60E-38	0.368472	0.377	0.193	5.24E-34	CD8_Naive	EIF2A
NOP583	2.05E-38	0.35443	0.342	0.167	6.72E-34	CD8_Naive	NOP58
ANAPC163	2.64E-38	0.324784	0.564	0.335	8.66E-34	CD8_Naive	ANAPC16
ABHD14B3	2.79E-38	0.310291	0.351	0.171	9.14E-34	CD8_Naive	ABHD14B
CTD-3184	5.24E-38	0.260493	0.198	0.074	1.71E-33	CD8_Naive	CTD-3184A7.4
BUB33	7.78E-38	0.346316	0.4	0.21	2.55E-33	CD8_Naive	BUB3
RPL27A3	1.30E-37	0.346171	0.901	0.642	4.27E-33	CD8_Naive	RPL27A
DDX182	2.95E-37	0.279472	0.557	0.328	9.67E-33	CD8_Naive	DDX18
TMEM2382	3.42E-37	0.26336	0.174	0.061	1.12E-32	CD8_Naive	TMEM238
TXNIP4	3.52E-37	0.318584	0.983	0.744	1.15E-32	CD8_Naive	TXNIP
EIF5A2	4.25E-37	0.308496	0.659	0.404	1.39E-32	CD8_Naive	EIF5A
HMCES1	4.52E-37	0.25919	0.178	0.063	1.48E-32	CD8_Naive	HMCES
CDKN1B3	9.87E-37	0.357311	0.369	0.19	3.23E-32	CD8_Naive	CDKN1B
PPIH2	9.94E-37	0.316798	0.269	0.12	3.25E-32	CD8_Naive	PPIH
SYPL13	1.51E-36	0.340012	0.324	0.159	4.94E-32	CD8_Naive	SYPL1
SNHG9	2.03E-36	0.285761	0.293	0.135	6.66E-32	CD8_Naive	SNHG9
MAML21	2.38E-36	0.286513	0.252	0.108	7.78E-32	CD8_Naive	MAML2
EIF2S33	7.07E-36	0.378755	0.529	0.319	2.32E-31	CD8_Naive	EIF2S3
ATP5A1	1.68E-35	0.325936	0.696	0.435	5.50E-31	CD8_Naive	ATP5A1
PPP1R3E1	1.97E-35	0.261457	0.167	0.059	6.45E-31	CD8_Naive	PPP1R3E
RHOF1	2.07E-35	0.255154	0.335	0.162	6.78E-31	CD8_Naive	RHOF
TNFAIP83	2.44E-35	0.297782	0.486	0.281	7.99E-31	CD8_Naive	TNFAIP8
NAE1	3.29E-35	0.264438	0.178	0.065	1.08E-30	CD8_Naive	NAE1
CCND21	4.49E-35	0.256581	0.212	0.084	1.47E-30	CD8_Naive	CCND2
NHP2L12	1.12E-34	0.276097	0.583	0.355	3.68E-30	CD8_Naive	NHP2L1

EGLN21	1.27E-34	0.258596	0.3	0.143	4.15E-30	CD8_Naive	EGLN2
SNRPF2	2.45E-34	0.356597	0.493	0.29	8.01E-30	CD8_Naive	SNRPF
PPWD1	3.85E-34	0.286662	0.257	0.115	1.26E-29	CD8_Naive	PPWD1
CCDC662	3.85E-34	0.262579	0.194	0.076	1.26E-29	CD8_Naive	CCDC66
KRR13	4.54E-34	0.253927	0.22	0.091	1.49E-29	CD8_Naive	KRR1
TATDN1	4.78E-34	0.298652	0.227	0.097	1.57E-29	CD8_Naive	TATDN1
PRMT12	5.12E-34	0.333377	0.333	0.169	1.68E-29	CD8_Naive	PRMT1
NHP22	6.26E-34	0.323904	0.45	0.256	2.05E-29	CD8_Naive	NHP2
NSMCE12	6.63E-34	0.313216	0.329	0.166	2.17E-29	CD8_Naive	NSMCE1
ZRANB21	1.08E-33	0.282699	0.475	0.276	3.53E-29	CD8_Naive	ZRANB2
RPA22	1.15E-33	0.300848	0.292	0.138	3.75E-29	CD8_Naive	RPA2
MRPL1	1.57E-33	0.266743	0.171	0.064	5.15E-29	CD8_Naive	MRPL1
ARHGEF31	1.71E-33	0.302571	0.308	0.148	5.59E-29	CD8_Naive	ARHGEF3
ZNF3021	1.71E-33	0.26902	0.229	0.098	5.61E-29	CD8_Naive	ZNF302
C11orf312	2.03E-33	0.284083	0.731	0.472	6.65E-29	CD8_Naive	C11orf31
TAF1D3	2.26E-33	0.321303	0.467	0.269	7.40E-29	CD8_Naive	TAF1D
ANP32B2	3.55E-33	0.269713	0.722	0.481	1.16E-28	CD8_Naive	ANP32B
TAF72	3.93E-33	0.310011	0.399	0.217	1.29E-28	CD8_Naive	TAF7
TOMM203	8.29E-33	0.357968	0.552	0.345	2.71E-28	CD8_Naive	TOMM20
SRSF73	1.32E-32	0.322963	0.557	0.342	4.33E-28	CD8_Naive	SRSF7
ZMYM6NE	1.55E-32	0.316016	0.284	0.136	5.08E-28	CD8_Naive	ZMYM6NB
BZW2	1.57E-32	0.295769	0.216	0.093	5.15E-28	CD8_Naive	BZW2
CD483	2.01E-32	0.311758	0.835	0.573	6.57E-28	CD8_Naive	CD48
ESYT11	2.62E-32	0.31466	0.242	0.109	8.57E-28	CD8_Naive	ESYT1
RCN22	2.82E-32	0.287831	0.264	0.122	9.24E-28	CD8_Naive	RCN2
LETMD11	4.14E-32	0.300442	0.254	0.117	1.35E-27	CD8_Naive	LETMD1
TMEM256	4.68E-32	0.320734	0.257	0.119	1.53E-27	CD8_Naive	TMEM256-PLSCR3
PMPCB	6.57E-32	0.321472	0.332	0.173	2.15E-27	CD8_Naive	PMPCB
TGFBR2	7.90E-32	0.26523	0.479	0.279	2.59E-27	CD8_Naive	TGFBR2
C14orf166	1.06E-31	0.32424	0.57	0.354	3.48E-27	CD8_Naive	C14orf166
METTL52	1.67E-31	0.279755	0.284	0.139	5.48E-27	CD8_Naive	METTL5
GIMAP22	1.74E-31	0.315906	0.372	0.201	5.70E-27	CD8_Naive	GIMAP2
MRFAP1L1	1.79E-31	0.272156	0.296	0.143	5.85E-27	CD8_Naive	MRFAP1L1
MED103	4.47E-31	0.286168	0.372	0.201	1.46E-26	CD8_Naive	MED10
ERGIC22	5.34E-31	0.267179	0.26	0.122	1.75E-26	CD8_Naive	ERGIC2
ITFG21	6.08E-31	0.294994	0.218	0.095	1.99E-26	CD8_Naive	ITFG2
TTC32	8.24E-30	0.309822	0.403	0.228	2.70E-25	CD8_Naive	TTC3
MRTO4	8.51E-30	0.254749	0.16	0.061	2.79E-25	CD8_Naive	MRTO4
POLR2C1	1.30E-29	0.264751	0.216	0.095	4.25E-25	CD8_Naive	POLR2C
SBDS2	1.60E-29	0.277462	0.247	0.116	5.24E-25	CD8_Naive	SBDS
HDDC21	1.83E-29	0.28004	0.278	0.136	5.98E-25	CD8_Naive	HDDC2
ZCCHC113	2.02E-29	0.271399	0.283	0.138	6.61E-25	CD8_Naive	ZCCHC11
CWF19L22	2.34E-29	0.289618	0.272	0.132	7.66E-25	CD8_Naive	CWF19L2
MRPS332	1.02E-28	0.276804	0.27	0.134	3.33E-24	CD8_Naive	MRPS33
TAGAP3	1.27E-28	0.308074	0.426	0.246	4.15E-24	CD8_Naive	TAGAP
FNTA1	1.37E-28	0.270722	0.38	0.214	4.48E-24	CD8_Naive	FNTA
PCBP23	1.45E-28	0.277613	0.882	0.637	4.73E-24	CD8_Naive	PCBP2
TRAPPC6A	3.49E-28	0.322406	0.292	0.151	1.14E-23	CD8_Naive	TRAPPC6A
BRIX12	3.76E-28	0.290941	0.187	0.079	1.23E-23	CD8_Naive	BRIX1
UBA522	5.91E-28	0.272366	0.991	0.836	1.93E-23	CD8_Naive	UBA52
HAX12	9.09E-28	0.275405	0.368	0.207	2.98E-23	CD8_Naive	HAX1
EPC13	1.27E-27	0.269229	0.48	0.295	4.16E-23	CD8_Naive	EPC1
TAPSAR13	1.31E-27	0.277024	0.411	0.239	4.28E-23	CD8_Naive	TAPSAR1
NIFK	1.69E-27	0.269773	0.326	0.174	5.53E-23	CD8_Naive	NIFK
NOLC1	1.73E-27	0.274876	0.211	0.095	5.66E-23	CD8_Naive	NOLC1
MRPL38	2.09E-27	0.288375	0.224	0.105	6.85E-23	CD8_Naive	MRPL38
SUCLG21	3.25E-27	0.281631	0.232	0.11	1.06E-22	CD8_Naive	SUCLG2
DNAJC192	3.39E-27	0.262996	0.297	0.155	1.11E-22	CD8_Naive	DNAJC19

MRPS271	7.92E-27	0.251602	0.162	0.066	2.59E-22	CD8_Naive	MRPS27
APRT2	8.25E-27	0.256986	0.757	0.512	2.70E-22	CD8_Naive	APRT
G3BP11	1.18E-26	0.285914	0.351	0.198	3.86E-22	CD8_Naive	G3BP1
SRP92	1.24E-26	0.277915	0.462	0.288	4.06E-22	CD8_Naive	SRP9
HNRNPA0	1.99E-26	0.277949	0.471	0.291	6.50E-22	CD8_Naive	HNRNPA0
CLPP2	2.02E-26	0.265594	0.29	0.152	6.61E-22	CD8_Naive	CLPP
DCK1	4.23E-26	0.258012	0.273	0.139	1.38E-21	CD8_Naive	DCK
MRPL50	5.68E-26	0.268756	0.184	0.081	1.86E-21	CD8_Naive	MRPL50
CDK43	6.00E-26	0.269594	0.218	0.103	1.96E-21	CD8_Naive	CDK4
EIF3D3	4.48E-25	0.30189	0.502	0.325	1.47E-20	CD8_Naive	EIF3D
AP1M11	1.15E-24	0.254093	0.3	0.164	3.75E-20	CD8_Naive	AP1M1
PPIA3	1.31E-24	0.285404	0.965	0.731	4.28E-20	CD8_Naive	PPIA
MRPS261	2.20E-23	0.279806	0.254	0.134	7.20E-19	CD8_Naive	MRPS26
SSBP12	8.11E-23	0.270921	0.484	0.312	2.65E-18	CD8_Naive	SSBP1
PFDN53	1.24E-22	0.257099	0.995	0.803	4.07E-18	CD8_Naive	PFDN5
SDHC	9.26E-22	0.250687	0.367	0.224	3.03E-17	CD8_Naive	SDHC
IL323	0	2.988994	0.964	0.177	0	CD8T	IL32
GZMK1	0	2.941095	0.554	0.021	0	CD8T	GZMK
GZMH	0	2.58773	0.519	0.06	0	CD8T	GZMH
NKG7	0	2.490116	0.94	0.18	0	CD8T	NKG7
CCL5	0	2.450216	0.953	0.2	0	CD8T	CCL5
GZMA	0	2.41139	0.87	0.092	0	CD8T	GZMA
CD8B1	0	2.33712	0.538	0.028	0	CD8T	CD8B
CD3D3	0	2.322241	0.901	0.133	0	CD8T	CD3D
CD3E3	0	2.288643	0.952	0.167	0	CD8T	CD3E
CD8A1	0	2.265713	0.609	0.03	0	CD8T	CD8A
PTPRCAP4	0	2.107338	0.971	0.241	0	CD8T	PTPRCAP
CST7	0	2.060977	0.891	0.138	0	CD8T	CST7
CD3G3	0	2.055427	0.837	0.12	0	CD8T	CD3G
KLRG1	0	1.896212	0.621	0.052	0	CD8T	KLRG1
CTSW1	0	1.89566	0.915	0.154	0	CD8T	CTSW
IL7R3	0	1.887072	0.599	0.137	0	CD8T	IL7R
CD24	0	1.867666	0.761	0.149	0	CD8T	CD2
GZMM1	0	1.764353	0.751	0.107	0	CD8T	GZMM
LCK3	0	1.740055	0.808	0.159	0	CD8T	LCK
KLRB11	0	1.608573	0.38	0.092	0	CD8T	KLRB1
IL2RG4	0	1.598444	0.902	0.303	0	CD8T	IL2RG
IFITM13	0	1.590476	0.97	0.304	0	CD8T	IFITM1
RARRES33	0	1.513216	0.856	0.338	0	CD8T	RARRES3
PRF1	0	1.482044	0.77	0.094	0	CD8T	PRF1
KLRD1	0	1.456518	0.552	0.073	0	CD8T	KLRD1
CMC1	0	1.42916	0.505	0.137	0	CD8T	CMC1
CCL4	0	1.402762	0.59	0.069	0	CD8T	CCL4
SYNE21	0	1.387112	0.571	0.101	0	CD8T	SYNE2
ARL4C2	0	1.341587	0.648	0.166	0	CD8T	ARL4C
LINC00861	0	1.332713	0.701	0.161	0	CD8T	LINC00861
RPS274	0	1.328314	0.98	0.824	0	CD8T	RPS27
SAMD3	0	1.309408	0.504	0.063	0	CD8T	SAMD3
GBP51	0	1.308218	0.548	0.143	0	CD8T	GBP5
GIMAP53	0	1.28639	0.745	0.252	0	CD8T	GIMAP5
STK17A4	0	1.247912	0.679	0.203	0	CD8T	STK17A
HOPX	0	1.220594	0.563	0.085	0	CD8T	HOPX
AC092580	0	1.219972	0.386	0.029	0	CD8T	AC092580.4
SH2D1A2	0	1.207284	0.413	0.05	0	CD8T	SH2D1A
HSPA85	0	1.207219	0.956	0.558	0	CD8T	HSPA8
C12orf574	0	1.207184	0.789	0.278	0	CD8T	C12orf57
TC2N3	0	1.178803	0.523	0.094	0	CD8T	TC2N
MT-ND4L4	0	1.161617	0.982	0.764	0	CD8T	MT-ND4L

HCST	0	1.156735	0.94	0.598	0	CD8T	HCST
DENND2D	0	1.156362	0.586	0.17	0	CD8T	DENND2D
APOBEC3(	0	1.145952	0.526	0.137	0	CD8T	APOBEC3G
FGFBP2	0	1.145625	0.366	0.063	0	CD8T	FGFBP2
KLF25	0	1.137078	0.946	0.509	0	CD8T	KLF2
SKAP13	0	1.135708	0.556	0.122	0	CD8T	SKAP1
SPOCK23	0	1.132157	0.489	0.105	0	CD8T	SPOCK2
NCR31	0	1.12654	0.34	0.053	0	CD8T	NCR3
MATK	0	1.125576	0.483	0.071	0	CD8T	MATK
ISG204	0	1.122953	0.565	0.16	0	CD8T	ISG20
ITM2A3	0	1.121868	0.47	0.095	0	CD8T	ITM2A
RPS34	0	1.117304	0.99	0.824	0	CD8T	RPS3
RPS294	0	1.117059	0.969	0.756	0	CD8T	RPS29
MT-ATP85	0	1.114198	0.948	0.727	0	CD8T	MT-ATP8
AES4	0	1.102798	0.847	0.399	0	CD8T	AES
RPL315	0	1.101795	0.974	0.735	0	CD8T	RPL3
SLC9A3R1	0	1.100929	0.722	0.297	0	CD8T	SLC9A3R1
LBH4	0	1.098479	0.574	0.145	0	CD8T	LBH
DUSP2	0	1.097631	0.304	0.034	0	CD8T	DUSP2
CD811	0	1.091991	0.629	0.225	0	CD8T	CD81
14-Sep	0	1.07875	0.51	0.125	0	CD8T	1-Sep
ETS14	0	1.077776	0.602	0.169	0	CD8T	ETS1
LYAR	0	1.065736	0.475	0.125	0	CD8T	LYAR
GIMAP73	0	1.060507	0.935	0.555	0	CD8T	GIMAP7
CLEC2D4	0	1.054309	0.413	0.073	0	CD8T	CLEC2D
B2M1	0	1.052541	0.999	0.978	0	CD8T	B2M
EVL4	0	1.044303	0.721	0.268	0	CD8T	EVL
CD2473	0	1.041837	0.653	0.148	0	CD8T	CD247
BIN14	0	1.028863	0.518	0.135	0	CD8T	BIN1
LIME13	0	1.028227	0.423	0.083	0	CD8T	LIME1
ZAP703	0	1.024371	0.507	0.104	0	CD8T	ZAP70
S1PR14	0	1.017976	0.456	0.093	0	CD8T	S1PR1
XBP1	0	1.01737	0.615	0.248	0	CD8T	XBP1
RPSA4	0	1.009375	0.966	0.686	0	CD8T	RPSA
RAC24	0	1.009076	0.914	0.564	0	CD8T	RAC2
RPL23A4	0	1.004734	0.971	0.729	0	CD8T	RPL23A
CD75	0	1.004302	0.69	0.177	0	CD8T	CD7
EOMES	0	1.004215	0.341	0.028	0	CD8T	EOMES
RPS195	0	1.003692	0.986	0.807	0	CD8T	RPS19
CD273	0	0.992175	0.38	0.088	0	CD8T	CD27
LITAF	0	0.982579	0.778	0.372	0	CD8T	LITAF
PYHIN1	0	0.982212	0.394	0.063	0	CD8T	PYHIN1
GIMAP43	0	0.979686	0.858	0.495	0	CD8T	GIMAP4
RP11-291I	0	0.976724	0.173	0.016	0	CD8T	RP11-291B21.2
OPTN1	0	0.976529	0.446	0.094	0	CD8T	OPTN
HLA-C	0	0.965385	0.987	0.882	0	CD8T	HLA-C
SIT14	0	0.964595	0.38	0.062	0	CD8T	SIT1
C9orf142	0	0.95878	0.647	0.275	0	CD8T	C9orf142
HLA-A	0	0.947651	0.984	0.859	0	CD8T	HLA-A
GYPC3	0	0.942794	0.621	0.238	0	CD8T	GYPC
RPS15A4	0	0.937836	0.99	0.863	0	CD8T	RPS15A
RPS254	0	0.927936	0.967	0.766	0	CD8T	RPS25
GZMB	0	0.927548	0.386	0.082	0	CD8T	GZMB
TIGIT	0	0.921787	0.263	0.03	0	CD8T	TIGIT
PRKCH3	0	0.919706	0.437	0.097	0	CD8T	PRKCH
RPS184	0	0.895988	0.977	0.78	0	CD8T	RPS18
RORA1	0	0.889004	0.369	0.073	0	CD8T	RORA
TBC1D10C	0	0.887075	0.53	0.197	0	CD8T	TBC1D10C

CXCR31	0	0.885545	0.283	0.037	0	CD8T	CXCR3
ITGB71	0	0.883807	0.403	0.11	0	CD8T	ITGB7
PSMB91	0	0.878436	0.861	0.533	0	CD8T	PSMB9
RPS124	0	0.877935	0.994	0.876	0	CD8T	RPS12
MYBL1	0	0.876016	0.328	0.045	0	CD8T	MYBL1
EEF1A15	0	0.875572	0.988	0.887	0	CD8T	EEF1A1
TBX21	0	0.870682	0.345	0.055	0	CD8T	TBX21
PTPN4	0	0.869668	0.411	0.095	0	CD8T	PTPN4
ARHGAP1!	0	0.866308	0.512	0.191	0	CD8T	ARHGAP15
RPS27A4	0	0.866012	0.991	0.862	0	CD8T	RPS27A
RPL144	0	0.857915	0.985	0.803	0	CD8T	RPL14
RPS54	0	0.84472	0.969	0.693	0	CD8T	RPS5
NPM14	0	0.842541	0.87	0.508	0	CD8T	NPM1
RPS4X4	0	0.825303	0.984	0.804	0	CD8T	RPS4X
CALM11	0	0.795589	0.973	0.745	0	CD8T	CALM1
SH2D2A	0	0.79477	0.324	0.054	0	CD8T	SH2D2A
RPL10A4	0	0.789168	0.956	0.675	0	CD8T	RPL10A
FCRL6	0	0.786772	0.265	0.035	0	CD8T	FCRL6
S1PR5	0	0.784199	0.302	0.044	0	CD8T	S1PR5
CD963	0	0.783691	0.384	0.083	0	CD8T	CD96
FKBP111	0	0.781632	0.337	0.078	0	CD8T	FKBP11
RPL104	0	0.780556	0.995	0.874	0	CD8T	RPL10
PPP3CC3	0	0.779616	0.378	0.112	0	CD8T	PPP3CC
LDHB3	0	0.77437	0.764	0.373	0	CD8T	LDHB
MT-CYB4	0	0.764266	0.993	0.928	0	CD8T	MT-CYB
APMAP	0	0.76131	0.441	0.145	0	CD8T	APMAP
RHOH4	0	0.743587	0.37	0.097	0	CD8T	RHOH
STAT43	0	0.736341	0.319	0.062	0	CD8T	STAT4
SCML43	0	0.734276	0.3	0.065	0	CD8T	SCML4
RPS64	0	0.732216	0.977	0.773	0	CD8T	RPS6
LY94	0	0.725714	0.291	0.054	0	CD8T	LY9
THEMIS4	0	0.725692	0.25	0.033	0	CD8T	THEMIS
OCIAD24	0	0.725363	0.357	0.093	0	CD8T	OCIAD2
MYL12A1	0	0.723243	0.978	0.749	0	CD8T	MYL12A
TSEN54	0	0.721173	0.296	0.068	0	CD8T	TSEN54
PLEKHF1	0	0.720252	0.286	0.054	0	CD8T	PLEKHF1
RPL7A4	0	0.712783	0.986	0.832	0	CD8T	RPL7A
RPS215	0	0.705495	0.985	0.829	0	CD8T	RPS21
PTGDR	0	0.705112	0.27	0.053	0	CD8T	PTGDR
CHST12	0	0.701038	0.361	0.093	0	CD8T	CHST12
CD692	0	0.696984	0.271	0.051	0	CD8T	CD69
HLA-B	0	0.693217	0.988	0.92	0	CD8T	HLA-B
RPL134	0	0.687606	0.992	0.856	0	CD8T	RPL13
RPL54	0	0.681689	0.984	0.792	0	CD8T	RPL5
TGFBR3	0	0.669405	0.231	0.035	0	CD8T	TGFBR3
RPL304	0	0.654472	0.991	0.882	0	CD8T	RPL30
RPS234	0	0.653664	0.984	0.818	0	CD8T	RPS23
RP11-94L1	0	0.651912	0.225	0.038	0	CD8T	RP11-94L15.2
C12orf75	0	0.645386	0.518	0.129	0	CD8T	C12orf75
LAG3	0	0.639855	0.189	0.005	0	CD8T	LAG3
PPP2R2B	0	0.627941	0.206	0.024	0	CD8T	PPP2R2B
SLAMF61	0	0.613347	0.226	0.035	0	CD8T	SLAMF6
CXCR6	0	0.606485	0.151	0.005	0	CD8T	CXCR6
RPS144	0	0.606102	0.991	0.868	0	CD8T	RPS14
LAT4	0	0.597158	0.535	0.16	0	CD8T	LAT
RPS74	0	0.586742	0.988	0.859	0	CD8T	RPS7
C1orf21	0	0.586432	0.217	0.038	0	CD8T	C1orf21
XCL2	0	0.571968	0.209	0.034	0	CD8T	XCL2

AC006369	0	0.525564	0.175	0.018	0	CD8T	AC006369.2
KIF21A	0	0.496142	0.159	0.017	0	CD8T	KIF21A
GPR1711	0	0.48009	0.164	0.018	0	CD8T	GPR171
COL6A2	0	0.438233	0.127	0.011	0	CD8T	COL6A2
JAKMIP1	0	0.434745	0.139	0.008	0	CD8T	JAKMIP1
RP11-222I	0	0.39977	0.129	0.011	0	CD8T	RP11-222K16.2
TTC16	0	0.343945	0.106	0.007	0	CD8T	TTC16
RPL194	#######	0.573123	0.991	0.864	#######	CD8T	RPL19
ACAP14	#######	0.827869	0.575	0.239	#######	CD8T	ACAP1
ARPC5L	#######	0.866764	0.511	0.203	#######	CD8T	ARPC5L
RPL36AL3	#######	0.751534	0.953	0.711	#######	CD8T	RPL36AL
CLEC2B1	#######	0.898966	0.561	0.24	#######	CD8T	CLEC2B
RPL174	#######	0.808619	0.946	0.704	#######	CD8T	RPL17
ADRB2	#######	0.731587	0.302	0.077	#######	CD8T	ADRB2
RPS3A4	#######	0.58122	0.99	0.851	#######	CD8T	RPS3A
CDC25B3	#######	0.655903	0.299	0.076	#######	CD8T	CDC25B
PCED1B3	#######	0.629268	0.338	0.093	#######	CD8T	PCED1B
CLIC3	#######	0.542462	0.306	0.073	#######	CD8T	CLIC3
FLT3LG3	#######	0.71985	0.402	0.129	#######	CD8T	FLT3LG
SLC38A14	#######	0.717746	0.363	0.109	#######	CD8T	SLC38A1
IKZF31	#######	0.55729	0.202	0.037	#######	CD8T	IKZF3
RASGRP13	#######	0.579761	0.222	0.044	#######	CD8T	RASGRP1
RPL18A4	#######	0.604044	0.986	0.82	#######	CD8T	RPL18A
LY6E2	#######	0.809665	0.717	0.356	#######	CD8T	LY6E
FAIM34	#######	0.708212	0.398	0.124	#######	CD8T	FAIM3
CD525	#######	0.846753	0.933	0.636	#######	CD8T	CD52
RPL354	#######	0.669383	0.972	0.761	#######	CD8T	RPL35
SSR24	#######	0.804152	0.792	0.469	#######	CD8T	SSR2
RPL324	#######	0.536962	0.99	0.865	#######	CD8T	RPL32
PTPN71	#######	0.602351	0.254	0.059	#######	CD8T	PTPN7
RPL364	#######	0.587777	0.981	0.824	#######	CD8T	RPL36
SLFN53	#######	0.834908	0.46	0.172	#######	CD8T	SLFN5
RPS154	#######	0.543351	0.985	0.861	#######	CD8T	RPS15
EEF1B24	#######	0.698346	0.948	0.66	#######	CD8T	EEF1B2
ZBTB38	#######	0.753439	0.376	0.123	#######	CD8T	ZBTB38
GPR56	#######	0.643353	0.221	0.045	#######	CD8T	GPR56
CBLB	#######	0.62231	0.275	0.07	#######	CD8T	CBLB
NFATC21	#######	0.64068	0.282	0.072	#######	CD8T	NFATC2
RPL265	#######	0.654442	0.978	0.798	#######	CD8T	RPL26
CD56	#######	0.54745	0.218	0.046	#######	CD8T	CD5
SYTL2	#######	0.49493	0.178	0.031	#######	CD8T	SYTL2
TSTD14	#######	0.727563	0.437	0.159	#######	CD8T	TSTD1
HMOX23	#######	0.747365	0.435	0.163	#######	CD8T	HMOX2
ANXA2R3	#######	0.62596	0.294	0.079	#######	CD8T	ANXA2R
ANXA62	#######	0.732809	0.681	0.348	#######	CD8T	ANXA6
GNLY	#######	0.753675	0.345	0.098	#######	CD8T	GNLY
RHOF2	#######	0.688006	0.418	0.151	#######	CD8T	RHOF
KLRC3	#######	0.748972	0.144	0.021	#######	CD8T	KLRC3
HLA-E	#######	0.424706	0.972	0.798	#######	CD8T	HLA-E
CD64	#######	0.598954	0.247	0.059	#######	CD8T	CD6
C5orf56	#######	0.710447	0.378	0.128	#######	CD8T	C5orf56
CCDC1071	#######	0.731541	0.427	0.164	#######	CD8T	CCDC107
PRR51	#######	0.477782	0.177	0.033	#######	CD8T	PRR5
RPL344	#######	0.525718	0.988	0.868	#######	CD8T	RPL34
SNRPD24	#######	0.708493	0.859	0.562	#######	CD8T	SNRPD2
RAB7L1	#######	0.676646	0.374	0.129	#######	CD8T	RAB7L1
TAGAP4	#######	0.772552	0.531	0.234	#######	CD8T	TAGAP
CD99	#######	0.531772	0.896	0.573	#######	CD8T	CD99

RPLP04	#######	0.663308	0.976	0.768	#######	CD8T	RPLP0
PDCD43	#######	0.788083	0.494	0.214	#######	CD8T	PDCD4
PPIB	#######	0.713482	0.877	0.575	#######	CD8T	PPIB
RPL374	#######	0.501241	0.986	0.862	#######	CD8T	RPL37
MALAT14	#######	0.61017	0.996	0.942	#######	CD8T	MALAT1
PTPRC1	#######	0.701696	0.958	0.744	#######	CD8T	PTPRC
KLRK1	#######	0.289107	0.107	0.012	#######	CD8T	KLRK1
PEBP14	#######	0.746506	0.516	0.231	#######	CD8T	PEBP1
SOD15	#######	0.66813	0.695	0.378	#######	CD8T	SOD1
MIF4	#######	0.708575	0.848	0.557	#######	CD8T	MIF
TRAT12	#######	0.540704	0.239	0.058	#######	CD8T	TRAT1
ABHD17A	#######	0.659313	0.515	0.225	#######	CD8T	ABHD17A
HNRNPA1	#######	0.690138	0.909	0.616	#######	CD8T	HNRNPA1
FYN	#######	0.747601	0.542	0.254	#######	CD8T	FYN
RPL35A4	#######	0.494612	0.985	0.866	#######	CD8T	RPL35A
TAP1	#######	0.777402	0.511	0.233	#######	CD8T	TAP1
SLAMF7	#######	0.557638	0.25	0.066	#######	CD8T	SLAMF7
CALR	#######	0.893034	0.643	0.356	#######	CD8T	CALR
EEF1D4	#######	0.567671	0.969	0.779	#######	CD8T	EEF1D
HIST1H4C4	#######	0.732823	0.784	0.469	#######	CD8T	HIST1H4C
TCF73	#######	0.59213	0.34	0.106	#######	CD8T	TCF7
CD3201	#######	0.549107	0.216	0.051	#######	CD8T	CD320
TMEM664	#######	0.63818	0.854	0.536	#######	CD8T	TMEM66
BCL11B3	#######	0.507923	0.232	0.058	#######	CD8T	BCL11B
ITK3	#######	0.562474	0.256	0.069	#######	CD8T	ITK
SLAMF12	#######	0.418468	0.152	0.027	#######	CD8T	SLAMF1
BUB34	#######	0.741975	0.459	0.199	#######	CD8T	BUB3
SLFN12L1	#######	0.433315	0.182	0.037	#######	CD8T	SLFN12L
PDIA3	#######	0.740769	0.704	0.406	#######	CD8T	PDIA3
RPL294	#######	0.507914	0.984	0.828	#######	CD8T	RPL29
TRAF3IP34	#######	0.715933	0.719	0.417	#######	CD8T	TRAF3IP3
RPL414	#######	0.584121	0.984	0.879	#######	CD8T	RPL41
RPL114	#######	0.448679	0.994	0.878	#######	CD8T	RPL11
CTSC1	#######	0.76012	0.679	0.383	#######	CD8T	CTSC
RPS216	#######	0.554795	0.981	0.808	#######	CD8T	RPS2
PARP8	#######	0.658501	0.387	0.148	#######	CD8T	PARP8
TXNIP5	#######	0.659473	0.959	0.736	#######	CD8T	TXNIP
XCL1	#######	0.384985	0.116	0.017	#######	CD8T	XCL1
MYL12B1	#######	0.623019	0.889	0.627	#######	CD8T	MYL12B
PIM12	#######	0.617078	0.41	0.162	#######	CD8T	PIM1
PRMT23	#######	0.619459	0.678	0.376	#######	CD8T	PRMT2
PSME11	#######	0.640163	0.924	0.679	#######	CD8T	PSME1
UBB	#######	0.4789	0.856	0.568	#######	CD8T	UBB
RPS104	#######	0.594488	0.934	0.756	#######	CD8T	RPS10
ZMYM6NE	#######	0.644724	0.343	0.127	#######	CD8T	ZMYM6NB
HINT14	#######	0.638008	0.873	0.598	#######	CD8T	HINT1
7-Sep	#######	0.571454	0.763	0.476	#######	CD8T	7-Sep
NUCB22	#######	0.744364	0.388	0.155	#######	CD8T	NUCB2
SUN23	#######	0.666213	0.543	0.271	#######	CD8T	SUN2
LTB4	#######	0.934539	0.556	0.27	#######	CD8T	LTB
RPL284	#######	0.445444	0.992	0.893	#######	CD8T	RPL28
RAN4	#######	0.639074	0.726	0.44	#######	CD8T	RAN
PTGER21	#######	0.608043	0.304	0.103	#######	CD8T	PTGER2
RBL23	#######	0.689529	0.505	0.245	#######	CD8T	RBL2
PPP2R5C	#######	0.659936	0.444	0.198	#######	CD8T	PPP2R5C
SYNE1	#######	0.480552	0.385	0.147	#######	CD8T	SYNE1
HSP90AB1	#######	0.709189	0.816	0.539	#######	CD8T	HSP90AB1
HNRNPF1	#######	0.65339	0.647	0.376	#######	CD8T	HNRNPF

KCNA31	#######	0.351783	0.191	0.046	#######	CD8T	KCNA3
ODF2L1	#######	0.526386	0.239	0.07	#######	CD8T	ODF2L
DDX243	#######	0.654364	0.629	0.354	#######	CD8T	DDX24
GATA31	#######	0.458752	0.197	0.049	#######	CD8T	GATA3
NCALD	#######	0.344073	0.139	0.026	#######	CD8T	NCALD
IL2RB	#######	0.350607	0.226	0.059	#######	CD8T	IL2RB
RPS4Y14	#######	0.71658	0.741	0.451	#######	CD8T	RPS4Y1
EEF1G4	#######	0.669983	0.796	0.51	#######	CD8T	EEF1G
CYTIP4	#######	0.675825	0.516	0.255	#######	CD8T	CYTIP
NSG11	#######	0.336012	0.115	0.019	#######	CD8T	NSG1
NCL4	#######	0.643538	0.766	0.485	#######	CD8T	NCL
RPL64	#######	0.447867	0.989	0.858	#######	CD8T	RPL6
PRDM11	#######	0.443154	0.174	0.041	#######	CD8T	PRDM1
CD160	#######	0.437761	0.143	0.028	#######	CD8T	CD160
FCRL31	#######	0.396484	0.122	0.021	#######	CD8T	FCRL3
OFD11	#######	0.552003	0.288	0.099	#######	CD8T	OFD1
CCDC1671	#######	0.482305	0.222	0.064	#######	CD8T	CCDC167
PRKACB2	#######	0.543954	0.353	0.138	#######	CD8T	PRKACB
LINC00649	#######	0.394055	0.159	0.035	#######	CD8T	LINC00649
PCSK1N3	#######	0.415039	0.12	0.021	#######	CD8T	PCSK1N
GLTSCR24	#######	0.62956	0.723	0.445	#######	CD8T	GLTSCR2
MT-ND52	#######	0.562609	0.971	0.784	#######	CD8T	MT-ND5
RPL184	#######	0.435747	0.989	0.844	#######	CD8T	RPL18
RNF1671	#######	0.622647	0.437	0.205	#######	CD8T	RNF167
CNN24	#######	0.643594	0.636	0.375	#######	CD8T	CNN2
SP1402	#######	0.5319	0.228	0.068	#######	CD8T	SP140
TNFSF14	#######	0.432982	0.195	0.052	#######	CD8T	TNFSF14
PFN11	#######	0.520274	0.991	0.908	#######	CD8T	PFN1
MAT2B4	#######	0.610496	0.522	0.275	#######	CD8T	MAT2B
TUBB5	#######	0.58819	0.635	0.369	#######	CD8T	TUBB
PRDX23	#######	0.608067	0.45	0.211	#######	CD8T	PRDX2
YWHAQ	#######	0.526933	0.513	0.265	#######	CD8T	YWHAQ
PPIA4	#######	0.56366	0.942	0.723	#######	CD8T	PPIA
EZR3	#######	0.639259	0.527	0.278	#######	CD8T	EZR
NPDC11	#######	0.330293	0.113	0.02	#######	CD8T	NPDC1
ERN11	#######	0.554503	0.261	0.088	#######	CD8T	ERN1
HSP90AA1	#######	0.586789	0.861	0.605	#######	CD8T	HSP90AA1
UBASH3A3	#######	0.358065	0.122	0.023	#######	CD8T	UBASH3A
PITPNC1	#######	0.529797	0.29	0.105	#######	CD8T	PITPNC1
RPL214	#######	0.496671	0.963	0.808	#######	CD8T	RPL21
AC006129	#######	0.506392	0.267	0.089	#######	CD8T	AC006129.2
CCSER21	#######	0.523166	0.277	0.097	#######	CD8T	CCSER2
BTG14	#######	0.580394	0.913	0.672	#######	CD8T	BTG1
SYTL12	#######	0.575913	0.373	0.157	#######	CD8T	SYTL1
SYTL3	#######	0.55904	0.247	0.08	#######	CD8T	SYTL3
RASAL3	#######	0.583322	0.333	0.132	#######	CD8T	RASAL3
CLDND11	#######	0.619648	0.317	0.125	#######	CD8T	CLDND1
RPS264	#######	0.788531	0.973	0.822	#######	CD8T	RPS26
PRSS23	#######	0.426921	0.16	0.037	#######	CD8T	PRSS23
GCC23	#######	0.631631	0.45	0.218	#######	CD8T	GCC2
RASSF1	#######	0.583353	0.358	0.151	#######	CD8T	RASSF1
RUNX31	#######	0.5635	0.279	0.1	#######	CD8T	RUNX3
RPS164	#######	0.483152	0.962	0.757	#######	CD8T	RPS16
CISH3	#######	0.590551	0.236	0.075	#######	CD8T	CISH
SPCS21	#######	0.61748	0.554	0.312	#######	CD8T	SPCS2
TECR3	#######	0.632635	0.443	0.219	#######	CD8T	TECR
ARL6IP1	#######	0.56118	0.519	0.28	#######	CD8T	ARL6IP1
MRFAP1L1	#######	0.562788	0.333	0.136	#######	CD8T	MRFAP1L1

IMP34	#######	0.588457	0.499	0.265	#######	CD8T	IMP3
IL164	#######	0.596742	0.48	0.247	#######	CD8T	IL16
PRKCQ2	#######	0.367042	0.159	0.038	#######	CD8T	PRKCQ
SNRPN3	#######	0.391368	0.264	0.091	#######	CD8T	SNRPN
ARHGEF32	#######	0.555224	0.34	0.14	#######	CD8T	ARHGEF3
PLCG13	#######	0.360007	0.142	0.032	#######	CD8T	PLCG1
LMAN11	#######	0.486051	0.341	0.141	#######	CD8T	LMAN1
SYNRG1	#######	0.596575	0.405	0.19	#######	CD8T	SYNRG
RPA23	#######	0.541399	0.323	0.131	#######	CD8T	RPA2
CAMK43	#######	0.461571	0.232	0.073	#######	CD8T	CAMK4
CRTAM1	#######	0.406332	0.143	0.033	#######	CD8T	CRTAM
EIF4A24	#######	0.607966	0.531	0.295	#######	CD8T	EIF4A2
SPN1	#######	0.590614	0.513	0.268	#######	CD8T	SPN
PRKCQ-AS	#######	0.438357	0.3	0.109	#######	CD8T	PRKCQ-AS1
HSPA5	#######	0.632653	0.545	0.306	#######	CD8T	HSPA5
HSPE14	#######	0.631563	0.461	0.237	#######	CD8T	HSPE1
RPL384	#######	0.489399	0.943	0.716	#######	CD8T	RPL38
SIRPG3	#######	0.386392	0.143	0.033	#######	CD8T	SIRPG
TNIK3	#######	0.386401	0.209	0.063	#######	CD8T	TNIK
ARHGDIB	#######	0.345011	0.96	0.78	#######	CD8T	ARHGDIB
HMGN14	#######	0.448864	0.614	0.347	#######	CD8T	HMGN1
PDIA6	#######	0.620148	0.486	0.265	#######	CD8T	PDIA6
MZT2B4	#######	0.507418	0.737	0.474	#######	CD8T	MZT2B
HLA-F	#######	0.564653	0.612	0.368	#######	CD8T	HLA-F
SRSF74	#######	0.576227	0.569	0.334	#######	CD8T	SRSF7
USP112	#######	0.432384	0.203	0.063	#######	CD8T	USP11
GNG21	#######	0.564121	0.461	0.235	#######	CD8T	GNG2
RRAS21	#######	0.302956	0.12	0.025	#######	CD8T	RRAS2
SS18L22	#######	0.545348	0.362	0.165	#######	CD8T	SS18L2
GLCCI1	#######	0.373049	0.156	0.04	#######	CD8T	GLCCI1
FAM107B2	#######	0.539551	0.55	0.308	#######	CD8T	FAM107B
PHF15	#######	0.428564	0.208	0.066	#######	CD8T	PHF1
DDIT43	#######	0.464082	0.267	0.096	#######	CD8T	DDIT4
BTN3A2	#######	0.531179	0.398	0.189	#######	CD8T	BTN3A2
OASL	#######	0.360484	0.141	0.034	#######	CD8T	OASL
CCND22	#######	0.446347	0.231	0.078	#######	CD8T	CCND2
INPP4B2	#######	0.40685	0.18	0.051	#######	CD8T	INPP4B
TRAF11	#######	0.341635	0.128	0.029	#######	CD8T	TRAF1
FASLG	#######	0.3017	0.114	0.023	#######	CD8T	FASLG
CEP78	#######	0.467892	0.192	0.058	#######	CD8T	CEP78
HSP90B1	#######	0.540179	0.716	0.462	#######	CD8T	HSP90B1
Sep-64	#######	0.49435	0.54	0.301	#######	CD8T	6-Sep
MBP	#######	0.501052	0.622	0.38	#######	CD8T	MBP
GUK1	#######	0.477768	0.744	0.497	#######	CD8T	GUK1
ORMDL33	#######	0.418528	0.197	0.061	#######	CD8T	ORMDL3
ZNF222	#######	0.548564	0.33	0.144	#######	CD8T	ZNF22
PSIP14	#######	0.546232	0.434	0.219	#######	CD8T	PSIP1
LIMA1	#######	0.340771	0.125	0.028	#######	CD8T	LIMA1
CYFIP25	#######	0.479162	0.362	0.162	#######	CD8T	CYFIP2
KLF121	#######	0.426663	0.196	0.062	#######	CD8T	KLF12
TOX	#######	0.310909	0.112	0.023	#######	CD8T	TOX
SEC11C1	#######	0.524794	0.37	0.176	#######	CD8T	SEC11C
GIMAP13	#######	0.552988	0.638	0.4	#######	CD8T	GIMAP1
TOMM74	#######	0.450193	0.924	0.691	#######	CD8T	TOMM7
MT-CO12	#######	0.291882	0.999	0.985	#######	CD8T	MT-CO1
KRT104	#######	0.552431	0.54	0.316	#######	CD8T	KRT10
KLRC1	#######	0.537534	0.12	0.027	#######	CD8T	KLRC1
CDK2AP21	#######	0.598047	0.485	0.27	#######	CD8T	CDK2AP2

ARHGAP2!	#######	0.502932	0.357	0.166	#######	CD8T	ARHGAP25
KRTCAP2	#######	0.446525	0.725	0.485	#######	CD8T	KRTCAP2
PARP151	#######	0.414912	0.174	0.052	#######	CD8T	PARP15
GNB2L14	#######	0.415436	0.972	0.792	#######	CD8T	GNB2L1
ZNF4282	#######	0.489307	0.357	0.168	#######	CD8T	ZNF428
ORAI11	#######	0.444423	0.432	0.225	#######	CD8T	ORAI1
MAP4K12	#######	0.439843	0.204	0.07	#######	CD8T	MAP4K1
TTC38	#######	0.44739	0.208	0.069	#######	CD8T	TTC38
RPS284	#######	0.340729	0.991	0.895	#######	CD8T	RPS28
PSMA5	#######	0.548321	0.491	0.283	#######	CD8T	PSMA5
PBXIP12	#######	0.500649	0.272	0.11	#######	CD8T	PBXIP1
BATF	#######	0.468223	0.263	0.104	#######	CD8T	BATF
SELM3	#######	0.372592	0.175	0.054	#######	CD8T	SELM
ICAM32	#######	0.444962	0.634	0.391	#######	CD8T	ICAM3
C12orf654	#######	0.462089	0.229	0.084	#######	CD8T	C12orf65
CNBP2	#######	0.482357	0.746	0.513	#######	CD8T	CNBP
PSMB81	#######	0.517396	0.681	0.453	#######	CD8T	PSMB8
RP11-473I	#######	0.304713	0.127	0.031	#######	CD8T	RP11-473M20.7
CD531	#######	0.472586	0.681	0.452	#######	CD8T	CD53
SUPT3H3	#######	0.341252	0.148	0.041	#######	CD8T	SUPT3H
STMN14	#######	0.472442	0.199	0.067	#######	CD8T	STMN1
LAIR2	#######	0.417266	0.104	0.022	#######	CD8T	LAIR2
CCT74	#######	0.497056	0.423	0.227	#######	CD8T	CCT7
MT2A2	#######	0.279679	0.694	0.436	#######	CD8T	MT2A
SLC25A38:	#######	0.361553	0.157	0.046	#######	CD8T	SLC25A38
C19orf66	#######	0.461332	0.295	0.127	#######	CD8T	C19orf66
MT1E	#######	0.460653	0.172	0.054	#######	CD8T	MT1E
DHRS7	#######	0.434888	0.593	0.364	#######	CD8T	DHRS7
TPD522	#######	0.32867	0.154	0.044	#######	CD8T	TPD52
WDR54	#######	0.33151	0.144	0.04	#######	CD8T	WDR54
ZFP36L2	#######	0.472808	0.883	0.645	#######	CD8T	ZFP36L2
RPL154	#######	0.40956	0.97	0.794	#######	CD8T	RPL15
DAXX	#######	0.507009	0.315	0.145	#######	CD8T	DAXX
RPL244	#######	0.384014	0.967	0.793	#######	CD8T	RPL24
SFXN13	#######	0.376164	0.21	0.075	#######	CD8T	SFXN1
HMGN42	#######	0.451365	0.24	0.095	#######	CD8T	HMGN4
NOSIP3	#######	0.605247	0.512	0.301	#######	CD8T	NOSIP
MZT2A4	#######	0.499093	0.367	0.182	#######	CD8T	MZT2A
SUB1	#######	0.490027	0.766	0.544	#######	CD8T	SUB1
TRAF51	#######	0.30288	0.131	0.034	#######	CD8T	TRAF5
MED104	#######	0.489706	0.376	0.195	#######	CD8T	MED10
CIRBP5	#######	0.472161	0.662	0.438	#######	CD8T	CIRBP
BOLA32	#######	0.407832	0.203	0.073	#######	CD8T	BOLA3
OXCT1	#######	0.298821	0.127	0.033	#######	CD8T	OXCT1
BEX24	#######	0.350352	0.154	0.046	#######	CD8T	BEX2
DNAJB91	#######	0.351698	0.183	0.062	#######	CD8T	DNAJB9
GOLGA8B:	#######	0.348033	0.151	0.045	#######	CD8T	GOLGA8B
PPP1CA1	#######	0.461763	0.759	0.532	#######	CD8T	PPP1CA
CD471	#######	0.392959	0.514	0.301	#######	CD8T	CD47
DAD11	#######	0.403008	0.61	0.396	#######	CD8T	DAD1
HNRNPA0	#######	0.49014	0.483	0.284	#######	CD8T	HNRNPA0
SPCS11	#######	0.409489	0.647	0.42	#######	CD8T	SPCS1
RP11-47L:	#######	0.298026	0.122	0.031	#######	CD8T	RP11-47L3.1
RP11-18H	#######	0.313831	0.134	0.037	#######	CD8T	RP11-18H21.1
CRIP12	#######	0.360429	0.785	0.515	#######	CD8T	CRIP1
AAK13	#######	0.414553	0.459	0.254	#######	CD8T	AAK1
HSPB111	#######	0.479251	0.294	0.135	#######	CD8T	HSPB11
GTF3A2	#######	0.506629	0.542	0.339	#######	CD8T	GTF3A

LIMD25	#######	0.470394	0.813	0.58	#######	CD8T	LIMD2
TMA7	#######	0.34011	0.962	0.803	#######	CD8T	TMA7
TMEM109:	#######	0.486959	0.292	0.134	#######	CD8T	TMEM109
SIGIRR2	#######	0.479332	0.455	0.26	#######	CD8T	SIGIRR
NELL23	#######	0.309678	0.119	0.031	#######	CD8T	NELL2
EIF5A3	#######	0.445753	0.607	0.397	#######	CD8T	EIF5A
DEF62	#######	0.445254	0.418	0.229	#######	CD8T	DEF6
POLR3GL1	#######	0.480262	0.373	0.194	#######	CD8T	POLR3GL
PPM1K4	#######	0.411042	0.254	0.106	#######	CD8T	PPM1K
KLRF1	#######	0.3804	0.186	0.061	#######	CD8T	KLRF1
TERF2IP	#######	0.395559	0.519	0.315	#######	CD8T	TERF2IP
ATP6V0E2	#######	0.341027	0.17	0.056	#######	CD8T	ATP6V0E2
NMT22	#######	0.297661	0.116	0.03	#######	CD8T	NMT2
SKP11	#######	0.347176	0.784	0.562	#######	CD8T	SKP1
KIAA1430:	#######	0.411774	0.233	0.094	#######	CD8T	KIAA1430
JAK1	#######	0.325707	0.761	0.533	#######	CD8T	JAK1
ARAP21	#######	0.421658	0.267	0.116	#######	CD8T	ARAP2
CORO1A1	#######	0.452176	0.921	0.723	1.03E-99	CD8T	CORO1A
ADH52	#######	0.452287	0.347	0.175	1.20E-99	CD8T	ADH5
PARP12	#######	0.458425	0.379	0.203	7.49E-99	CD8T	PARP1
BLOC1S41	#######	0.43623	0.285	0.131	1.59E-98	CD8T	BLOC1S4
PYURF	#######	0.439292	0.483	0.289	1.94E-98	CD8T	PYURF
MANF	#######	0.438085	0.252	0.108	2.84E-97	CD8T	MANF
CCDC88C	#######	0.425551	0.321	0.156	3.65E-97	CD8T	CCDC88C
SRI1	#######	0.456593	0.463	0.274	4.69E-97	CD8T	SRI
LEPROTL1:	#######	0.448358	0.432	0.244	5.00E-97	CD8T	LEPROTL1
BTN3A1	#######	0.437035	0.239	0.099	1.34E-96	CD8T	BTN3A1
RPL316	#######	0.390818	0.938	0.725	1.70E-96	CD8T	RPL31
LRBA	#######	0.299525	0.198	0.073	2.20E-96	CD8T	LRBA
APRT3	#######	0.440069	0.722	0.505	1.02E-95	CD8T	APRT
MIB2	#######	0.370254	0.183	0.065	1.08E-95	CD8T	MIB2
PTGES32	#######	0.425673	0.645	0.437	2.06E-95	CD8T	PTGES3
SELK	1.02E-99	0.434751	0.411	0.231	3.33E-95	CD8T	SELK
ATPIF1	1.58E-99	0.421617	0.605	0.405	5.17E-95	CD8T	ATPIF1
GIMAP61	1.89E-99	0.44005	0.312	0.15	6.19E-95	CD8T	GIMAP6
C14orf11	1.15E-98	0.382715	0.164	0.056	3.77E-94	CD8T	C14orf1
ASF1A3	2.53E-98	0.409382	0.212	0.085	8.27E-94	CD8T	ASF1A
TTC39C1	9.74E-98	0.502148	0.277	0.128	3.19E-93	CD8T	TTC39C
RPL274	1.02E-97	0.402944	0.939	0.728	3.35E-93	CD8T	RPL27
URI14	1.29E-97	0.458932	0.356	0.188	4.23E-93	CD8T	URI1
LCP21	1.31E-97	0.40547	0.502	0.306	4.30E-93	CD8T	LCP2
HNRNPDL	2.32E-97	0.419306	0.663	0.451	7.61E-93	CD8T	HNRNPDL
MEAF61	5.30E-97	0.448379	0.39	0.217	1.74E-92	CD8T	MEAF6
NPRL2	1.02E-96	0.36741	0.181	0.066	3.34E-92	CD8T	NPRL2
TMEM238:	2.96E-96	0.344023	0.166	0.057	9.70E-92	CD8T	TMEM238
TMEM14A	4.44E-96	0.262042	0.104	0.027	1.45E-91	CD8T	TMEM14A
RPL124	4.98E-96	0.319242	0.987	0.863	1.63E-91	CD8T	RPL12
IDH21	1.28E-95	0.490341	0.367	0.202	4.20E-91	CD8T	IDH2
S1PR42	3.44E-95	0.427488	0.457	0.271	1.13E-90	CD8T	S1PR4
CARD111	3.86E-95	0.333825	0.136	0.042	1.26E-90	CD8T	CARD11
MT-ND62	1.38E-94	0.526691	0.612	0.406	4.53E-90	CD8T	MT-ND6
C19orf12	2.56E-94	0.33237	0.162	0.055	8.37E-90	CD8T	C19orf12
USP28	2.86E-94	0.36702	0.163	0.056	9.37E-90	CD8T	USP28
CYB561	5.09E-94	0.282744	0.105	0.027	1.67E-89	CD8T	CYB561
NAP1L42	6.90E-94	0.392341	0.442	0.259	2.26E-89	CD8T	NAP1L4
PCID2	1.76E-93	0.369123	0.201	0.079	5.77E-89	CD8T	PCID2
GPRIN31	3.12E-92	0.436641	0.23	0.1	1.02E-87	CD8T	GPRIN3
RCN23	3.69E-92	0.427164	0.257	0.117	1.21E-87	CD8T	RCN2

EBP	4.51E-92	0.350357	0.174	0.064	1.48E-87	CD8T	EBP
PTPN22	1.47E-91	0.355458	0.212	0.086	4.82E-87	CD8T	PTPN22
CDC42SE2	4.21E-91	0.394689	0.394	0.221	1.38E-86	CD8T	CDC42SE2
RP11-660l	4.84E-91	0.254327	0.104	0.028	1.58E-86	CD8T	RP11-660L16.2
STK42	6.65E-91	0.375747	0.64	0.438	2.18E-86	CD8T	STK4
TMC62	8.44E-91	0.405108	0.31	0.155	2.76E-86	CD8T	TMC6
THRAP31	1.61E-90	0.384107	0.514	0.325	5.28E-86	CD8T	THRAP3
TMC83	2.18E-90	0.428051	0.302	0.149	7.14E-86	CD8T	TMC8
LLGL2	2.87E-90	0.264637	0.114	0.032	9.41E-86	CD8T	LLGL2
FUS4	3.04E-90	0.444143	0.615	0.42	9.97E-86	CD8T	FUS
PNRC13	4.13E-90	0.37924	0.877	0.66	1.35E-85	CD8T	PNRC1
TFDP2	1.30E-89	0.353814	0.172	0.063	4.25E-85	CD8T	TFDP2
EIF3F4	1.93E-89	0.405269	0.749	0.533	6.31E-85	CD8T	EIF3F
NGRN4	3.92E-89	0.380799	0.195	0.078	1.28E-84	CD8T	NGRN
SHISA5	4.07E-89	0.404441	0.447	0.27	1.33E-84	CD8T	SHISA5
DNAJB13	5.06E-89	0.42141	0.272	0.129	1.66E-84	CD8T	DNAJB1
ITGA41	5.42E-89	0.505017	0.498	0.31	1.78E-84	CD8T	ITGA4
TRMT1122	1.14E-88	0.372377	0.626	0.428	3.72E-84	CD8T	TRMT112
OXNAD13	1.21E-88	0.337423	0.188	0.071	3.97E-84	CD8T	OXNAD1
AKR1B12	1.95E-88	0.416861	0.283	0.138	6.39E-84	CD8T	AKR1B1
XRCC62	2.46E-88	0.407524	0.542	0.353	8.04E-84	CD8T	XRCC6
NHP2L13	9.44E-88	0.419384	0.533	0.35	3.09E-83	CD8T	NHP2L1
PRMT13	2.01E-87	0.442547	0.314	0.164	6.58E-83	CD8T	PRMT1
NUCKS12	2.01E-87	0.445992	0.451	0.275	6.59E-83	CD8T	NUCKS1
BZW1	3.22E-87	0.415731	0.491	0.31	1.05E-82	CD8T	BZW1
PATL2	1.26E-86	0.311907	0.13	0.041	4.14E-82	CD8T	PATL2
CD283	2.00E-86	0.25668	0.118	0.035	6.56E-82	CD8T	CD28
ABT11	2.23E-86	0.412636	0.248	0.116	7.31E-82	CD8T	ABT1
MEI11	4.48E-86	0.295867	0.131	0.042	1.47E-81	CD8T	MEI1
RPS204	6.63E-86	0.370291	0.806	0.581	2.17E-81	CD8T	RPS20
LINC-PINT	8.70E-86	0.345754	0.164	0.06	2.85E-81	CD8T	LINC-PINT
ABHD14B4	1.20E-85	0.414527	0.317	0.166	3.92E-81	CD8T	ABHD14B
PTPLAD11	1.25E-85	0.334822	0.143	0.049	4.10E-81	CD8T	PTPLAD1
GLS2	1.29E-85	0.477229	0.283	0.141	4.23E-81	CD8T	GLS
GIPC1	1.80E-85	0.260041	0.117	0.035	5.88E-81	CD8T	GIPC1
DNAJC193	2.01E-85	0.411212	0.296	0.15	6.58E-81	CD8T	DNAJC19
GPR1833	2.71E-85	0.434543	0.192	0.077	8.89E-81	CD8T	GPR183
C14orf643	2.97E-85	0.258604	0.112	0.032	9.73E-81	CD8T	C14orf64
RALGDS	3.15E-85	0.307123	0.153	0.053	1.03E-80	CD8T	RALGDS
CUTA3	4.27E-85	0.357649	0.571	0.378	1.40E-80	CD8T	CUTA
TMEM256	1.34E-84	0.430935	0.248	0.115	4.40E-80	CD8T	TMEM256-PLSCR3
SSBP41	3.65E-84	0.451331	0.34	0.187	1.19E-79	CD8T	SSBP4
NDNL23	1.07E-83	0.371158	0.221	0.098	3.51E-79	CD8T	NDNL2
VPS13A	1.36E-83	0.338477	0.157	0.057	4.45E-79	CD8T	VPS13A
PPP1R18	2.52E-83	0.297516	0.586	0.393	8.25E-79	CD8T	PPP1R18
COX7C5	3.39E-83	0.368871	0.915	0.695	1.11E-78	CD8T	COX7C
NOP584	4.36E-83	0.41394	0.31	0.162	1.43E-78	CD8T	NOP58
ATP2B4	6.18E-83	0.409622	0.283	0.14	2.02E-78	CD8T	ATP2B4
SAP181	8.39E-83	0.338931	0.605	0.416	2.75E-78	CD8T	SAP18
BTN3A3	8.84E-83	0.360902	0.206	0.087	2.89E-78	CD8T	BTN3A3
GS1-251I9	2.95E-82	0.381118	0.277	0.136	9.67E-78	CD8T	GS1-251I9.4
DNAJC1	5.81E-82	0.414566	0.324	0.174	1.90E-77	CD8T	DNAJC1
AIP2	9.49E-82	0.425182	0.407	0.246	3.11E-77	CD8T	AIP
MFSD10	9.76E-82	0.457488	0.308	0.164	3.20E-77	CD8T	MFSD10
LINC00623	2.14E-81	0.300312	0.14	0.048	7.00E-77	CD8T	LINC00623
TOMM5	2.98E-81	0.329812	0.433	0.263	9.77E-77	CD8T	TOMM5
UBLCP1	1.08E-80	0.376651	0.278	0.14	3.54E-76	CD8T	UBLCP1
EPB412	1.10E-80	0.326351	0.363	0.202	3.60E-76	CD8T	EPB41

POLR2K	1.30E-80	0.388523	0.295	0.154	4.24E-76	CD8T	POLR2K
AQP33	1.58E-80	0.414561	0.184	0.073	5.18E-76	CD8T	AQP3
ZNRD1	1.68E-80	0.397145	0.333	0.183	5.48E-76	CD8T	ZNRD1
HENMT1	1.95E-80	0.292667	0.141	0.049	6.38E-76	CD8T	HENMT1
FUT11	1.99E-80	0.340206	0.179	0.071	6.51E-76	CD8T	FUT11
ACAA2	2.79E-80	0.454362	0.287	0.148	9.13E-76	CD8T	ACAA2
P2RY82	9.27E-80	0.385273	0.245	0.115	3.03E-75	CD8T	P2RY8
MAD1L11	9.45E-80	0.421309	0.239	0.113	3.09E-75	CD8T	MAD1L1
HDAC13	9.90E-80	0.427808	0.366	0.212	3.24E-75	CD8T	HDAC1
KIAA1551	1.46E-79	0.437473	0.471	0.297	4.79E-75	CD8T	KIAA1551
PTRH1	1.88E-79	0.273118	0.127	0.042	6.17E-75	CD8T	PTRH1
ANKRD12	1.97E-79	0.411334	0.565	0.384	6.44E-75	CD8T	ANKRD12
ATP8A1	3.08E-79	0.297857	0.146	0.052	1.01E-74	CD8T	ATP8A1
GBP11	6.75E-79	0.41858	0.305	0.157	2.21E-74	CD8T	GBP1
TMEM59	8.58E-79	0.367272	0.598	0.411	2.81E-74	CD8T	TMEM59
RPS27L2	1.02E-78	0.416565	0.531	0.357	3.34E-74	CD8T	RPS27L
RPL37A4	1.43E-78	0.337522	0.966	0.801	4.68E-74	CD8T	RPL37A
ZCCHC11	1.62E-78	0.371788	0.272	0.133	5.31E-74	CD8T	ZCCHC11
RPSAP584	1.21E-77	0.406559	0.259	0.127	3.96E-73	CD8T	RPSAP58
EDF1	1.27E-77	0.335892	0.743	0.543	4.15E-73	CD8T	EDF1
PLEKHA1	3.48E-77	0.319356	0.176	0.07	1.14E-72	CD8T	PLEKHA1
DIP2A	4.40E-77	0.344594	0.291	0.148	1.44E-72	CD8T	DIP2A
ANAPC16	5.31E-77	0.364926	0.505	0.33	1.74E-72	CD8T	ANAPC16
CCT44	6.80E-77	0.39089	0.427	0.266	2.23E-72	CD8T	CCT4
GSTK12	7.15E-77	0.390432	0.7	0.504	2.34E-72	CD8T	GSTK1
BTF34	7.69E-77	0.327864	0.937	0.733	2.52E-72	CD8T	BTF3
CDKN1B4	9.46E-77	0.402398	0.334	0.186	3.10E-72	CD8T	CDKN1B
TBCC2	1.09E-76	0.358484	0.223	0.103	3.55E-72	CD8T	TBCC
GIMAP23	1.29E-76	0.428222	0.348	0.197	4.24E-72	CD8T	GIMAP2
MAGOH	1.69E-76	0.416123	0.385	0.231	5.52E-72	CD8T	MAGOH
NAE11	2.74E-76	0.310303	0.161	0.062	8.99E-72	CD8T	NAE1
EPC14	7.44E-76	0.379136	0.458	0.289	2.44E-71	CD8T	EPC1
OSTC	9.65E-76	0.407111	0.421	0.261	3.16E-71	CD8T	OSTC
GPKOW	1.36E-75	0.291307	0.139	0.05	4.46E-71	CD8T	GPKOW
C19orf43	1.98E-75	0.336111	0.778	0.584	6.47E-71	CD8T	C19orf43
SUMO21	8.86E-75	0.328965	0.81	0.6	2.90E-70	CD8T	SUMO2
GLOD41	1.36E-74	0.387285	0.266	0.136	4.46E-70	CD8T	GLOD4
MT-ND33	1.40E-74	0.324153	0.984	0.903	4.60E-70	CD8T	MT-ND3
MRPL10	1.42E-74	0.385132	0.201	0.089	4.64E-70	CD8T	MRPL10
AMZ2	1.57E-74	0.33838	0.197	0.086	5.14E-70	CD8T	AMZ2
SSBP13	1.68E-74	0.40486	0.467	0.306	5.49E-70	CD8T	SSBP1
CCT34	4.28E-74	0.407792	0.392	0.238	1.40E-69	CD8T	CCT3
RUVBL1	5.87E-74	0.315121	0.152	0.058	1.92E-69	CD8T	RUVBL1
COX6C4	8.59E-74	0.33071	0.779	0.571	2.81E-69	CD8T	COX6C
UBE2N2	1.47E-73	0.387157	0.33	0.188	4.82E-69	CD8T	UBE2N
PRR5L	2.74E-73	0.289077	0.115	0.037	8.96E-69	CD8T	PRR5L
C9orf782	3.78E-73	0.360684	0.443	0.282	1.24E-68	CD8T	C9orf78
TRPV2	4.06E-73	0.352036	0.214	0.099	1.33E-68	CD8T	TRPV2
SDF2L1	1.65E-72	0.409624	0.291	0.156	5.39E-68	CD8T	SDF2L1
DDOST	4.70E-72	0.40807	0.388	0.237	1.54E-67	CD8T	DDOST
MATR33	5.42E-72	0.369554	0.499	0.333	1.78E-67	CD8T	MATR3
PSME21	5.71E-72	0.332567	0.728	0.514	1.87E-67	CD8T	PSME2
PRPS11	1.05E-71	0.291247	0.152	0.059	3.43E-67	CD8T	PRPS1
UXT4	1.24E-71	0.338476	0.609	0.426	4.07E-67	CD8T	UXT
ARF61	1.43E-71	0.396632	0.419	0.263	4.69E-67	CD8T	ARF6
RNF126	1.95E-71	0.371387	0.272	0.142	6.37E-67	CD8T	RNF126
REXO21	3.48E-71	0.320428	0.225	0.106	1.14E-66	CD8T	REXO2
LPXN	4.22E-71	0.39927	0.322	0.182	1.38E-66	CD8T	LPXN

RPL36A5	8.64E-71	0.339593	0.843	0.623	2.83E-66	CD8T	RPL36A
HAX13	1.73E-70	0.390364	0.345	0.202	5.65E-66	CD8T	HAX1
SET1	2.51E-70	0.325897	0.702	0.513	8.22E-66	CD8T	SET
SCP2	2.59E-70	0.318726	0.414	0.26	8.47E-66	CD8T	SCP2
KHDRBS12	2.93E-70	0.371063	0.56	0.389	9.60E-66	CD8T	KHDRBS1
UBE2D24	3.51E-70	0.299397	0.591	0.415	1.15E-65	CD8T	UBE2D2
TNFAIP84	4.34E-70	0.364044	0.436	0.276	1.42E-65	CD8T	TNFAIP8
CCT24	6.36E-70	0.415005	0.345	0.203	2.08E-65	CD8T	CCT2
SEPW1	7.10E-70	0.303411	0.521	0.349	2.32E-65	CD8T	SEPW1
RNPS11	9.19E-70	0.337795	0.426	0.269	3.01E-65	CD8T	RNPS1
HNRNPA3	1.46E-69	0.370637	0.622	0.452	4.79E-65	CD8T	HNRNPA3
NACA5	1.95E-69	0.291189	0.974	0.813	6.39E-65	CD8T	NACA
CD484	1.97E-69	0.326109	0.773	0.567	6.43E-65	CD8T	CD48
BCL24	2.45E-69	0.284677	0.148	0.056	8.02E-65	CD8T	BCL2
DDX63	2.56E-69	0.367499	0.319	0.178	8.37E-65	CD8T	DDX6
HDDC22	3.49E-69	0.409684	0.257	0.133	1.14E-64	CD8T	HDDC2
TAF73	3.69E-69	0.344974	0.36	0.212	1.21E-64	CD8T	TAF7
RPLP24	3.76E-69	0.256095	0.986	0.872	1.23E-64	CD8T	RPLP2
IRF12	8.75E-69	0.361599	0.659	0.471	2.87E-64	CD8T	IRF1
SLC2A4RG	1.08E-68	0.254185	0.135	0.05	3.52E-64	CD8T	SLC2A4RG
DTD1	1.43E-68	0.30751	0.186	0.082	4.70E-64	CD8T	DTD1
UFC1	1.95E-68	0.354535	0.517	0.356	6.38E-64	CD8T	UFC1
ZNF600	2.00E-68	0.314636	0.161	0.065	6.56E-64	CD8T	ZNF600
TMEM223	2.65E-68	0.332276	0.181	0.079	8.69E-64	CD8T	TMEM223
RPL85	3.28E-68	0.281137	0.985	0.852	1.07E-63	CD8T	RPL8
RBM8A	3.89E-68	0.349302	0.507	0.347	1.27E-63	CD8T	RBM8A
LMAN2	4.03E-68	0.35493	0.46	0.304	1.32E-63	CD8T	LMAN2
EMC4	6.41E-68	0.376742	0.325	0.189	2.10E-63	CD8T	EMC4
ITGB1BP1	7.13E-68	0.381142	0.304	0.171	2.33E-63	CD8T	ITGB1BP1
SRPR	8.96E-68	0.353542	0.391	0.242	2.93E-63	CD8T	SRPR
PCBP11	1.13E-67	0.271465	0.85	0.643	3.71E-63	CD8T	PCBP1
TES1	1.38E-67	0.350432	0.429	0.273	4.52E-63	CD8T	TES
C11orf58	3.47E-67	0.266053	0.633	0.452	1.14E-62	CD8T	C11orf58
SPTAN11	3.99E-67	0.368958	0.289	0.157	1.31E-62	CD8T	SPTAN1
TMCO1	4.41E-67	0.338956	0.445	0.289	1.44E-62	CD8T	TMCO1
PSMA6	6.00E-67	0.324227	0.489	0.327	1.96E-62	CD8T	PSMA6
ILF22	1.29E-66	0.375193	0.391	0.246	4.22E-62	CD8T	ILF2
CLPP3	1.57E-66	0.347837	0.274	0.148	5.13E-62	CD8T	CLPP
KRR14	1.94E-66	0.328142	0.193	0.088	6.36E-62	CD8T	KRR1
CCDC912	2.14E-66	0.37277	0.228	0.113	7.00E-62	CD8T	CCDC91
HIST1H1D	2.57E-66	0.475339	0.252	0.129	8.41E-62	CD8T	HIST1H1D
PILRB	5.65E-66	0.306903	0.17	0.072	1.85E-61	CD8T	PILRB
SNRNP401	7.00E-66	0.393047	0.249	0.129	2.29E-61	CD8T	SNRNP40
ARHGEF11	1.07E-65	0.398858	0.464	0.308	3.50E-61	CD8T	ARHGEF1
BCL7C	1.43E-65	0.356522	0.339	0.199	4.70E-61	CD8T	BCL7C
KMT2E1	1.54E-65	0.304375	0.541	0.369	5.04E-61	CD8T	KMT2E
PIK3R11	2.02E-65	0.38525	0.348	0.207	6.62E-61	CD8T	PIK3R1
CISD3	3.51E-65	0.377142	0.333	0.195	1.15E-60	CD8T	CISD3
NCOA7	3.71E-65	0.311405	0.239	0.12	1.21E-60	CD8T	NCOA7
TMEM230	3.93E-65	0.356114	0.373	0.231	1.29E-60	CD8T	TMEM230
PIK3IP13	6.03E-65	0.324429	0.221	0.105	1.97E-60	CD8T	PIK3IP1
UBC1	6.10E-65	0.267895	0.922	0.751	2.00E-60	CD8T	UBC
CYCS1	1.48E-64	0.351683	0.357	0.218	4.85E-60	CD8T	CYCS
EMP31	3.35E-64	0.286508	0.93	0.737	1.10E-59	CD8T	EMP3
FNTA2	4.73E-64	0.362741	0.348	0.21	1.55E-59	CD8T	FNTA
TBCB1	5.26E-64	0.321383	0.474	0.32	1.72E-59	CD8T	TBCB
THAP111	6.09E-64	0.301649	0.258	0.137	2.00E-59	CD8T	THAP11
ERH2	8.11E-64	0.33967	0.502	0.346	2.66E-59	CD8T	ERH

TMED2	1.34E-63	0.347427	0.371	0.23	4.39E-59	CD8T	TMED2
THEM43	1.43E-63	0.306772	0.176	0.077	4.69E-59	CD8T	THEM4
GORASP21	1.54E-63	0.334772	0.242	0.125	5.04E-59	CD8T	GORASP2
METTL231	1.73E-63	0.331545	0.232	0.118	5.65E-59	CD8T	METTL23
SDAD11	2.23E-63	0.376798	0.269	0.146	7.31E-59	CD8T	SDAD1
ACP1	2.31E-63	0.296412	0.379	0.234	7.56E-59	CD8T	ACP1
RBM43	2.73E-63	0.379291	0.317	0.186	8.94E-59	CD8T	RBM4
RNF113A	2.77E-63	0.328685	0.211	0.103	9.05E-59	CD8T	RNF113A
PIM24	2.83E-63	0.276844	0.177	0.077	9.28E-59	CD8T	PIM2
EAPP1	1.14E-62	0.363044	0.36	0.222	3.72E-58	CD8T	EAPP
PSMB101	1.32E-62	0.358398	0.659	0.483	4.32E-58	CD8T	PSMB10
PWP11	2.08E-62	0.369099	0.244	0.129	6.82E-58	CD8T	PWP1
UBE2L61	5.48E-62	0.348334	0.471	0.315	1.79E-57	CD8T	UBE2L6
EIF3G1	6.23E-62	0.297975	0.692	0.51	2.04E-57	CD8T	EIF3G
RNF125	9.84E-62	0.354716	0.254	0.134	3.22E-57	CD8T	RNF125
PUF60	1.02E-61	0.348526	0.319	0.19	3.32E-57	CD8T	PUF60
RINL1	1.35E-61	0.320459	0.149	0.061	4.43E-57	CD8T	RINL
IGSF81	1.39E-61	0.256064	0.127	0.048	4.55E-57	CD8T	IGSF8
ATM2	1.49E-61	0.345509	0.418	0.268	4.87E-57	CD8T	ATM
ECHDC2	1.93E-61	0.268407	0.107	0.037	6.32E-57	CD8T	ECHDC2
PPP1R35	2.91E-61	0.299441	0.188	0.087	9.54E-57	CD8T	PPP1R35
JADE2	3.08E-61	0.299246	0.153	0.064	1.01E-56	CD8T	JADE2
CTB-133G	3.08E-61	0.352467	0.278	0.152	1.01E-56	CD8T	CTB-133G6.1
PPIH3	3.60E-61	0.335574	0.228	0.117	1.18E-56	CD8T	PPIH
PA2G4	4.87E-61	0.311059	0.576	0.414	1.59E-56	CD8T	PA2G4
RAB9A	9.25E-61	0.347193	0.222	0.113	3.03E-56	CD8T	RAB9A
RASSF71	1.05E-60	0.289997	0.15	0.063	3.45E-56	CD8T	RASSF7
ESYT12	1.14E-60	0.344647	0.213	0.106	3.74E-56	CD8T	ESYT1
STMN33	1.30E-60	0.264836	0.117	0.043	4.24E-56	CD8T	STMN3
FAM162A4	2.33E-60	0.371921	0.254	0.137	7.63E-56	CD8T	FAM162A
FAS1	2.77E-60	0.261889	0.133	0.052	9.06E-56	CD8T	FAS
LSM21	3.20E-60	0.333502	0.34	0.207	1.05E-55	CD8T	LSM2
PSMC5	4.35E-60	0.306705	0.421	0.278	1.42E-55	CD8T	PSMC5
NDUFAF43	4.78E-60	0.344896	0.177	0.081	1.57E-55	CD8T	NDUFAF4
SRP93	4.79E-60	0.348135	0.427	0.283	1.57E-55	CD8T	SRP9
SESN13	5.79E-60	0.322074	0.15	0.063	1.90E-55	CD8T	SESN1
C19orf533	7.19E-60	0.274585	0.582	0.415	2.35E-55	CD8T	C19orf53
GNL1	1.16E-59	0.263761	0.161	0.07	3.78E-55	CD8T	GNL1
DDX51	1.24E-59	0.302701	0.79	0.597	4.07E-55	CD8T	DDX5
CIB12	1.52E-59	0.313203	0.518	0.358	4.97E-55	CD8T	CIB1
METTL53	1.77E-59	0.335404	0.251	0.135	5.79E-55	CD8T	METTL5
ID21	1.86E-59	0.354272	0.383	0.236	6.08E-55	CD8T	ID2
TMED9	1.98E-59	0.358473	0.373	0.238	6.49E-55	CD8T	TMED9
HMCES2	2.30E-59	0.251308	0.147	0.061	7.53E-55	CD8T	HMCES
NIP7	2.69E-59	0.276958	0.156	0.067	8.80E-55	CD8T	NIP7
GPR651	4.21E-59	0.399632	0.37	0.234	1.38E-54	CD8T	GPR65
PIN1	7.50E-59	0.321758	0.329	0.199	2.46E-54	CD8T	PIN1
PPHLN11	9.20E-59	0.335095	0.262	0.145	3.01E-54	CD8T	PPHLN1
IL12RB1	1.04E-58	0.278385	0.135	0.054	3.41E-54	CD8T	IL12RB1
REST1	1.10E-58	0.347835	0.341	0.207	3.59E-54	CD8T	REST
SUMO1	1.15E-58	0.294396	0.426	0.285	3.76E-54	CD8T	SUMO1
KLHDC22	1.41E-58	0.307829	0.192	0.092	4.63E-54	CD8T	KLHDC2
SH3BP52	1.45E-58	0.253564	0.312	0.178	4.75E-54	CD8T	SH3BP5
CDK17	2.00E-58	0.278691	0.153	0.065	6.55E-54	CD8T	CDK17
SDHA	2.70E-58	0.30914	0.286	0.163	8.84E-54	CD8T	SDHA
VBP1	3.02E-58	0.315482	0.247	0.133	9.90E-54	CD8T	VBP1
RPL13A4	3.59E-58	0.28547	0.955	0.758	1.17E-53	CD8T	RPL13A
SNRPB1	4.15E-58	0.362812	0.466	0.32	1.36E-53	CD8T	SNRPB

EML43	6.06E-58	0.366768	0.353	0.221	1.99E-53	CD8T	EML4
PLA2G16	6.25E-58	0.317038	0.223	0.113	2.04E-53	CD8T	PLA2G16
NUDCD21	6.37E-58	0.314938	0.229	0.12	2.09E-53	CD8T	NUDCD2
N4BP2L21	7.17E-58	0.290685	0.526	0.369	2.35E-53	CD8T	N4BP2L2
ARID5A	8.85E-58	0.290103	0.186	0.087	2.90E-53	CD8T	ARID5A
DDX183	1.82E-57	0.3189	0.474	0.324	5.96E-53	CD8T	DDX18
ADA1	2.03E-57	0.302711	0.151	0.066	6.63E-53	CD8T	ADA
ERGIC23	2.83E-57	0.328844	0.228	0.119	9.26E-53	CD8T	ERGIC2
ECH1	7.21E-57	0.325395	0.424	0.283	2.36E-52	CD8T	ECH1
MGAT4A3	7.46E-57	0.310115	0.218	0.11	2.44E-52	CD8T	MGAT4A
YARS	8.64E-57	0.326917	0.181	0.085	2.83E-52	CD8T	YARS
DENND1C	1.16E-56	0.31592	0.216	0.11	3.79E-52	CD8T	DENND1C
SNRPG1	1.64E-56	0.28913	0.582	0.417	5.37E-52	CD8T	SNRPG
VCP	2.18E-56	0.294464	0.473	0.324	7.15E-52	CD8T	VCP
UBE2V1	3.04E-56	0.289386	0.42	0.28	9.94E-52	CD8T	UBE2V1
ZRANB22	3.28E-56	0.303842	0.413	0.272	1.08E-51	CD8T	ZRANB2
LSM14A	3.49E-56	0.316564	0.407	0.269	1.14E-51	CD8T	LSM14A
NDUFA5	6.96E-56	0.261137	0.31	0.185	2.28E-51	CD8T	NDUFA5
PPP1R7	7.39E-56	0.349889	0.322	0.197	2.42E-51	CD8T	PPP1R7
MPHOSPH	1.08E-55	0.298805	0.492	0.341	3.55E-51	CD8T	MPHOSPH8
UCP22	1.23E-55	0.348989	0.718	0.543	4.04E-51	CD8T	UCP2
PRKD21	2.43E-55	0.254151	0.158	0.069	7.95E-51	CD8T	PRKD2
TGIF1	2.64E-55	0.260493	0.123	0.049	8.65E-51	CD8T	TGIF1
ZMYND11	2.91E-55	0.26308	0.155	0.068	9.52E-51	CD8T	ZMYND11
RFC1	4.65E-55	0.327219	0.299	0.176	1.52E-50	CD8T	RFC1
MCM31	8.42E-55	0.286634	0.146	0.063	2.76E-50	CD8T	MCM3
SRPK1	1.56E-54	0.260939	0.199	0.098	5.11E-50	CD8T	SRPK1
CDC1231	1.98E-54	0.302734	0.266	0.151	6.48E-50	CD8T	CDC123
RRP1B3	2.30E-54	0.309352	0.208	0.106	7.54E-50	CD8T	RRP1B
PPP1R23	2.53E-54	0.299261	0.409	0.269	8.29E-50	CD8T	PPP1R2
UBXN16	6.50E-54	0.293646	0.62	0.454	2.13E-49	CD8T	UBXN1
RAD21	8.52E-54	0.308625	0.43	0.292	2.79E-49	CD8T	RAD21
TSC22D4	9.48E-54	0.274222	0.36	0.228	3.11E-49	CD8T	TSC22D4
RASA22	1.09E-53	0.277927	0.242	0.13	3.57E-49	CD8T	RASA2
NDUFA12:	1.71E-53	0.320823	0.508	0.361	5.61E-49	CD8T	NDUFA12
SRM2	1.75E-53	0.37145	0.299	0.181	5.74E-49	CD8T	SRM
G3BP12	2.35E-53	0.329776	0.317	0.194	7.68E-49	CD8T	G3BP1
TRADD1	2.97E-53	0.335932	0.251	0.141	9.72E-49	CD8T	TRADD
PHB	2.97E-53	0.322913	0.306	0.186	9.73E-49	CD8T	PHB
MRPS6	3.80E-53	0.299084	0.362	0.229	1.24E-48	CD8T	MRPS6
FBL4	4.30E-53	0.365963	0.415	0.282	1.41E-48	CD8T	FBL
CASP8	6.22E-53	0.33889	0.267	0.153	2.04E-48	CD8T	CASP8
PTMS	1.06E-52	0.271408	0.143	0.062	3.46E-48	CD8T	PTMS
C14orf119	1.38E-52	0.293148	0.301	0.181	4.51E-48	CD8T	C14orf119
UCHL5	2.51E-52	0.318261	0.211	0.11	8.21E-48	CD8T	UCHL5
NDUFV21	3.10E-52	0.296012	0.461	0.321	1.02E-47	CD8T	NDUFV2
TMEM50B	3.86E-52	0.326192	0.213	0.112	1.26E-47	CD8T	TMEM50B
CDK44	4.61E-52	0.310234	0.196	0.1	1.51E-47	CD8T	CDK4
SSB2	9.16E-52	0.337296	0.428	0.296	3.00E-47	CD8T	SSB
TRAPPC4	2.32E-51	0.321349	0.229	0.125	7.60E-47	CD8T	TRAPPC4
ATIC4	3.15E-51	0.307849	0.183	0.091	1.03E-46	CD8T	ATIC
APBB1IP	4.63E-51	0.310433	0.577	0.427	1.52E-46	CD8T	APBB1IP
HNRNPR	5.06E-51	0.297168	0.433	0.296	1.66E-46	CD8T	HNRNPR
MAP2K2	5.45E-51	0.290811	0.435	0.3	1.78E-46	CD8T	MAP2K2
PHB24	9.46E-51	0.304919	0.484	0.343	3.10E-46	CD8T	PHB2
COPS8	1.02E-50	0.281648	0.176	0.086	3.35E-46	CD8T	COPS8
ATP5G31	1.30E-50	0.291278	0.664	0.486	4.25E-46	CD8T	ATP5G3
TEX264	2.49E-50	0.313607	0.294	0.179	8.15E-46	CD8T	TEX264

DYRK23	3.52E-50	0.279442	0.147	0.066	1.15E-45	CD8T	DYRK2
BRIX13	3.92E-50	0.297156	0.161	0.076	1.28E-45	CD8T	BRIX1
SRSF2	4.07E-50	0.337406	0.399	0.27	1.33E-45	CD8T	SRSF2
PSMD81	4.15E-50	0.28608	0.439	0.305	1.36E-45	CD8T	PSMD8
NGDN2	4.87E-50	0.320513	0.213	0.114	1.60E-45	CD8T	NGDN
CCNDBP1	5.38E-50	0.289396	0.371	0.246	1.76E-45	CD8T	CCNDBP1
MIR1421	8.28E-50	0.253569	0.376	0.242	2.71E-45	CD8T	MIR142
PBDC1	8.57E-50	0.297406	0.216	0.116	2.81E-45	CD8T	PBDC1
SMARCE11	1.15E-49	0.330804	0.317	0.198	3.76E-45	CD8T	SMARCE1
ECI2	1.21E-49	0.301781	0.174	0.086	3.97E-45	CD8T	ECI2
GBP41	1.30E-49	0.330735	0.243	0.134	4.26E-45	CD8T	GBP4
POLR2C2	1.76E-49	0.295895	0.184	0.093	5.77E-45	CD8T	POLR2C
SNRPA2	2.34E-49	0.319055	0.317	0.2	7.65E-45	CD8T	SNRPA
CDK61	3.25E-49	0.267241	0.137	0.06	1.06E-44	CD8T	CDK6
DNAJB14	5.28E-49	0.303156	0.29	0.175	1.73E-44	CD8T	DNAJB14
DERL1	5.83E-49	0.302369	0.245	0.139	1.91E-44	CD8T	DERL1
HNRNPL1	9.00E-49	0.280345	0.436	0.3	2.95E-44	CD8T	HNRNPL
SPSB3	1.23E-48	0.295479	0.31	0.193	4.04E-44	CD8T	SPSB3
ARID5B2	2.31E-48	0.280649	0.155	0.072	7.56E-44	CD8T	ARID5B
TAPSAR14	2.42E-48	0.31466	0.36	0.235	7.92E-44	CD8T	TAPSAR1
MLLT6	3.18E-48	0.257118	0.172	0.084	1.04E-43	CD8T	MLLT6
DUSP112	3.54E-48	0.264198	0.17	0.083	1.16E-43	CD8T	DUSP11
NOL71	5.33E-48	0.264238	0.417	0.287	1.74E-43	CD8T	NOL7
C14orf166	5.50E-48	0.268362	0.488	0.351	1.80E-43	CD8T	C14orf166
GADD45B	6.29E-48	0.312091	0.228	0.127	2.06E-43	CD8T	GADD45B
HSPA9	7.27E-48	0.337599	0.306	0.193	2.38E-43	CD8T	HSPA9
PDLIM2	8.07E-48	0.309853	0.3	0.187	2.64E-43	CD8T	PDLIM2
NFATC3	8.17E-48	0.34909	0.253	0.146	2.67E-43	CD8T	NFATC3
COX20	1.92E-47	0.308897	0.289	0.176	6.29E-43	CD8T	COX20
PSMA3	1.95E-47	0.290761	0.334	0.215	6.40E-43	CD8T	PSMA3
DUT1	2.11E-47	0.321358	0.379	0.257	6.89E-43	CD8T	DUT
DCK2	2.99E-47	0.274103	0.24	0.136	9.80E-43	CD8T	DCK
ATAD1	5.21E-47	0.262803	0.135	0.06	1.71E-42	CD8T	ATAD1
SAMD9	6.56E-47	0.312357	0.266	0.157	2.15E-42	CD8T	SAMD9
RDH141	9.56E-47	0.263023	0.147	0.069	3.13E-42	CD8T	RDH14
MDH12	1.07E-46	0.312831	0.346	0.23	3.49E-42	CD8T	MDH1
SNRPF3	1.17E-46	0.29304	0.415	0.287	3.82E-42	CD8T	SNRPF
PDCD52	1.25E-46	0.310072	0.357	0.238	4.09E-42	CD8T	PDCD5
OTUB11	1.96E-46	0.28609	0.446	0.317	6.41E-42	CD8T	OTUB1
MMADHC	2.10E-46	0.305769	0.317	0.203	6.87E-42	CD8T	MMADHC
SRPRB	2.16E-46	0.283178	0.145	0.067	7.06E-42	CD8T	SRPRB
MRPL43	6.18E-46	0.277888	0.331	0.215	2.02E-41	CD8T	MRPL43
OCIAD12	6.76E-46	0.280305	0.413	0.287	2.21E-41	CD8T	OCIAD1
GHITM	8.48E-46	0.260754	0.492	0.357	2.78E-41	CD8T	GHITM
RPAP2	2.14E-45	0.252684	0.144	0.067	7.02E-41	CD8T	RPAP2
PSMC41	2.22E-45	0.30866	0.279	0.173	7.28E-41	CD8T	PSMC4
CXCR44	2.87E-45	0.284497	0.277	0.164	9.38E-41	CD8T	CXCR4
FNBP41	5.28E-45	0.334031	0.345	0.226	1.73E-40	CD8T	FNBP4
NELFCD	6.33E-45	0.286503	0.191	0.102	2.07E-40	CD8T	NELFCD
ZNF276	6.47E-45	0.256201	0.176	0.089	2.12E-40	CD8T	ZNF276
RBBP71	6.57E-45	0.26012	0.253	0.149	2.15E-40	CD8T	RBBP7
PITHD1	6.71E-45	0.255409	0.198	0.107	2.20E-40	CD8T	PITHD1
UBA23	7.70E-45	0.289889	0.26	0.157	2.52E-40	CD8T	UBA2
SDHC1	9.88E-45	0.302238	0.334	0.221	3.24E-40	CD8T	SDHC
CDC26	1.13E-44	0.278473	0.274	0.167	3.69E-40	CD8T	CDC26
THYN12	2.01E-44	0.274223	0.254	0.151	6.59E-40	CD8T	THYN1
PPA13	2.02E-44	0.276342	0.373	0.25	6.61E-40	CD8T	PPA1
UBTF1	3.22E-44	0.323158	0.244	0.144	1.06E-39	CD8T	UBTF

BCAS2	4.23E-44	0.294818	0.19	0.101	1.39E-39	CD8T	BCAS2
CRELD2	4.87E-44	0.255265	0.171	0.087	1.59E-39	CD8T	CRELD2
ANKRD441	5.34E-44	0.290455	0.349	0.231	1.75E-39	CD8T	ANKRD44
SBDS3	5.80E-44	0.273572	0.207	0.114	1.90E-39	CD8T	SBDS
MAP4	5.94E-44	0.307399	0.205	0.113	1.94E-39	CD8T	MAP4
GADD45G	1.67E-43	0.289791	0.355	0.24	5.45E-39	CD8T	GADD45GIP1
CYB5B	1.69E-43	0.307861	0.198	0.108	5.54E-39	CD8T	CYB5B
ERAP2	3.14E-43	0.298246	0.22	0.123	1.03E-38	CD8T	ERAP2
U2SURP	3.49E-43	0.283084	0.402	0.282	1.14E-38	CD8T	U2SURP
MRPL501	5.78E-43	0.255714	0.158	0.078	1.89E-38	CD8T	MRPL50
ABI31	7.23E-43	0.317618	0.307	0.192	2.37E-38	CD8T	ABI3
RWDD1	8.30E-43	0.284865	0.472	0.344	2.72E-38	CD8T	RWDD1
ENOPH1	9.13E-43	0.263946	0.149	0.073	2.99E-38	CD8T	ENOPH1
POP4	1.55E-42	0.310732	0.209	0.118	5.06E-38	CD8T	POP4
SNHG85	1.58E-42	0.336111	0.411	0.289	5.17E-38	CD8T	SNHG8
NHP23	1.79E-42	0.298849	0.371	0.253	5.85E-38	CD8T	NHP2
ZGPAT	1.80E-42	0.257326	0.153	0.076	5.90E-38	CD8T	ZGPAT
TAF1D4	1.96E-42	0.300031	0.386	0.267	6.42E-38	CD8T	TAF1D
FGFR1OP2	2.55E-42	0.250683	0.339	0.225	8.35E-38	CD8T	FGFR1OP2
SCAMP2	2.56E-42	0.25675	0.398	0.277	8.38E-38	CD8T	SCAMP2
WBSCR221	3.16E-42	0.311849	0.207	0.116	1.03E-37	CD8T	WBSCR22
SRSF11	3.91E-42	0.255698	0.521	0.39	1.28E-37	CD8T	SRSF11
ALDH9A1	4.40E-42	0.287151	0.233	0.137	1.44E-37	CD8T	ALDH9A1
PSMD6	4.45E-42	0.250469	0.316	0.205	1.46E-37	CD8T	PSMD6
ISG151	5.70E-42	0.255442	0.293	0.184	1.87E-37	CD8T	ISG15
FRG1B	6.51E-42	0.281507	0.168	0.086	2.13E-37	CD8T	FRG1B
TROVE2	7.43E-42	0.283107	0.28	0.175	2.43E-37	CD8T	TROVE2
SNRPD3	1.72E-41	0.259077	0.435	0.313	5.62E-37	CD8T	SNRPD3
MRPL34	3.04E-41	0.284989	0.345	0.233	9.95E-37	CD8T	MRPL34
TRANK11	3.62E-41	0.266277	0.195	0.107	1.18E-36	CD8T	TRANK1
TRIM221	4.56E-41	0.277012	0.372	0.255	1.49E-36	CD8T	TRIM22
COPS6	5.46E-41	0.265292	0.334	0.224	1.79E-36	CD8T	COPS6
RNF5	6.09E-41	0.287684	0.242	0.145	1.99E-36	CD8T	RNF5
SARS	6.27E-41	0.292265	0.343	0.232	2.05E-36	CD8T	SARS
HERPUD2	7.99E-41	0.258506	0.2	0.111	2.61E-36	CD8T	HERPUD2
ABCF1	8.50E-41	0.315617	0.3	0.195	2.78E-36	CD8T	ABCF1
VPS513	1.10E-40	0.300911	0.311	0.203	3.61E-36	CD8T	VPS51
SASH3	2.44E-40	0.253243	0.376	0.26	7.97E-36	CD8T	SASH3
LPIN1	2.89E-40	0.254889	0.147	0.072	9.47E-36	CD8T	LPIN1
BAX1	3.00E-40	0.300363	0.493	0.368	9.82E-36	CD8T	BAX
PSMC2	3.05E-40	0.278157	0.215	0.125	9.99E-36	CD8T	PSMC2
SNRPA1	3.06E-40	0.296081	0.213	0.124	1.00E-35	CD8T	SNRPA1
MRPS18B3	3.85E-40	0.288914	0.261	0.162	1.26E-35	CD8T	MRPS18B
TINF2	5.71E-40	0.272933	0.251	0.153	1.87E-35	CD8T	TINF2
LDHA2	8.11E-40	0.263137	0.516	0.384	2.66E-35	CD8T	LDHA
APOBEC3C	8.29E-40	0.27794	0.239	0.144	2.71E-35	CD8T	APOBEC3C
HMGN3	2.00E-39	0.267136	0.475	0.349	6.56E-35	CD8T	HMGN3
PDIA4	4.48E-39	0.261026	0.232	0.139	1.47E-34	CD8T	PDIA4
LRIF1	4.61E-39	0.251226	0.147	0.074	1.51E-34	CD8T	LRIF1
TMED41	1.49E-38	0.291038	0.277	0.178	4.89E-34	CD8T	TMED4
ANP32E1	1.56E-38	0.289847	0.257	0.159	5.11E-34	CD8T	ANP32E
TMEM2434	1.89E-38	0.262264	0.263	0.165	6.18E-34	CD8T	TMEM243
FDX1	1.98E-38	0.284391	0.23	0.139	6.49E-34	CD8T	FDX1
NUDT5	3.17E-38	0.267782	0.239	0.146	1.04E-33	CD8T	NUDT5
PDCD7	3.92E-38	0.27421	0.235	0.143	1.28E-33	CD8T	PDCD7
ITGAL1	5.11E-38	0.307295	0.359	0.246	1.67E-33	CD8T	ITGAL
APOA1BP	6.59E-38	0.253187	0.267	0.168	2.16E-33	CD8T	APOA1BP
CEP571	6.79E-38	0.256989	0.258	0.16	2.22E-33	CD8T	CEP57

NUDT16L1	1.32E-37	0.284444	0.178	0.098	4.33E-33	CD8T	NUDT16L1
POP5	1.59E-37	0.294387	0.22	0.131	5.19E-33	CD8T	POP5
MTG11	3.87E-37	0.266385	0.177	0.097	1.27E-32	CD8T	MTG1
CACYBP3	5.39E-37	0.287295	0.314	0.214	1.77E-32	CD8T	CACYBP
IER3IP1.1	9.46E-37	0.250084	0.26	0.165	3.10E-32	CD8T	IER3IP1.1
NCK1	1.25E-36	0.267636	0.222	0.133	4.09E-32	CD8T	NCK1
KRAS1	1.45E-36	0.256036	0.353	0.245	4.74E-32	CD8T	KRAS
ATXN101	2.06E-36	0.299206	0.284	0.187	6.73E-32	CD8T	ATXN10
ITFG22	2.80E-36	0.272608	0.17	0.093	9.17E-32	CD8T	ITFG2
CCDC109E	4.16E-36	0.265761	0.392	0.285	1.36E-31	CD8T	CCDC109B
ADRM1	4.95E-36	0.263526	0.338	0.235	1.62E-31	CD8T	ADRM1
TAF15	1.60E-35	0.27308	0.381	0.273	5.25E-31	CD8T	TAF15
PHAX	1.91E-35	0.256368	0.187	0.107	6.26E-31	CD8T	PHAX
PDCD2	2.07E-35	0.283283	0.282	0.188	6.79E-31	CD8T	PDCD2
INO80E	4.27E-35	0.275248	0.217	0.132	1.40E-30	CD8T	INO80E
TRAPPC6A	5.37E-35	0.258955	0.239	0.149	1.76E-30	CD8T	TRAPPC6A
HARS	7.40E-35	0.250596	0.169	0.093	2.42E-30	CD8T	HARS
EMC7	9.83E-35	0.259941	0.258	0.167	3.22E-30	CD8T	EMC7
MICU2	1.21E-34	0.26067	0.217	0.132	3.96E-30	CD8T	MICU2
CYB561D2	1.48E-34	0.254489	0.235	0.148	4.84E-30	CD8T	CYB561D2
MDFIC1	1.66E-34	0.273625	0.237	0.148	5.42E-30	CD8T	MDFIC
BBX1	1.84E-34	0.280961	0.275	0.18	6.02E-30	CD8T	BBX
C1QBP4	2.23E-34	0.252682	0.391	0.284	7.29E-30	CD8T	C1QBP
TXNDC9	3.80E-34	0.252961	0.185	0.107	1.25E-29	CD8T	TXNDC9
PDAP1	6.13E-34	0.251243	0.299	0.203	2.01E-29	CD8T	PDAP1
TMED10	9.49E-34	0.268387	0.378	0.274	3.11E-29	CD8T	TMED10
EIF5B	1.04E-33	0.262098	0.388	0.281	3.41E-29	CD8T	EIF5B
PPM1G	1.36E-33	0.256101	0.399	0.292	4.46E-29	CD8T	PPM1G
LYPLA2	1.95E-33	0.260966	0.23	0.143	6.37E-29	CD8T	LYPLA2
ARL14EP2	2.09E-33	0.264806	0.21	0.128	6.84E-29	CD8T	ARL14EP
MESDC2	3.26E-33	0.261174	0.326	0.227	1.07E-28	CD8T	MESDC2
EPRS	3.34E-33	0.276181	0.284	0.191	1.09E-28	CD8T	EPRS
CX3CR11	4.21E-33	0.448291	0.345	0.249	1.38E-28	CD8T	CX3CR1
CWF19L23	8.40E-33	0.273562	0.215	0.13	2.75E-28	CD8T	CWF19L2
NT5C1	8.64E-33	0.285645	0.247	0.159	2.83E-28	CD8T	NT5C
ZNF800	1.11E-32	0.256134	0.267	0.176	3.63E-28	CD8T	ZNF800
HSPD14	1.42E-32	0.258157	0.369	0.266	4.64E-28	CD8T	HSPD1
PSMC3	1.46E-32	0.251787	0.271	0.18	4.78E-28	CD8T	PSMC3
SDHD2	1.54E-32	0.26974	0.294	0.202	5.05E-28	CD8T	SDHD
GNPTAB	4.80E-32	0.253301	0.254	0.163	1.57E-27	CD8T	GNPTAB
ARIH2	1.21E-30	0.268346	0.264	0.176	3.95E-26	CD8T	ARIH2
NXT1	2.49E-30	0.253758	0.143	0.078	8.15E-26	CD8T	NXT1
NAA10	4.27E-30	0.260177	0.298	0.207	1.40E-25	CD8T	NAA10
PMPCB1	2.30E-29	0.259741	0.254	0.171	7.52E-25	CD8T	PMPCB
TGS1	5.20E-29	0.26445	0.194	0.12	1.70E-24	CD8T	TGS1
AHSA1	4.50E-28	0.254126	0.21	0.134	1.47E-23	CD8T	AHSA1
AL592183.	2.73E-27	0.254392	0.324	0.234	8.92E-23	CD8T	AL592183.1
MRPL3	1.98E-26	0.264098	0.222	0.147	6.50E-22	CD8T	MRPL3
MRPS12	2.90E-25	0.251904	0.253	0.176	9.50E-21	CD8T	MRPS12
NDUFA9	4.40E-25	0.250249	0.2	0.13	1.44E-20	CD8T	NDUFA9
RANBP1	2.36E-23	0.267505	0.259	0.185	7.72E-19	CD8T	RANBP1
STAT11	1.68E-16	0.282448	0.34	0.269	5.48E-12	CD8T	STAT1
HLA-DQA	0	3.828079	0.992	0.176	0	cDC	HLA-DQA1
HLA-DPB1	0	3.288452	0.997	0.415	0	cDC	HLA-DPB1
HLA-DRA2	0	3.28431	1	0.488	0	cDC	HLA-DRA
FCER1A	0	3.275833	0.884	0.017	0	cDC	FCER1A
HLA-DPA1	0	3.264096	1	0.439	0	cDC	HLA-DPA1
HLA-DRB1	0	3.094317	0.999	0.504	0	cDC	HLA-DRB1

CD742	0	3.034951	1	0.772	0	cDC	CD74
HLA-DQB1	0	2.814965	0.994	0.349	0	cDC	HLA-DQB1
HLA-DRB5	0	2.510532	0.952	0.307	0	cDC	HLA-DRB5
CLEC10A	0	2.27854	0.708	0.03	0	cDC	CLEC10A
CD1C1	0	2.269777	0.789	0.018	0	cDC	CD1C
HLA-DMA	0	1.849106	0.976	0.291	0	cDC	HLA-DMA
ENHO	0	1.823703	0.697	0.007	0	cDC	ENHO
CST32	0	1.775686	1	0.673	0	cDC	CST3
PLD42	0	1.238165	0.782	0.043	0	cDC	PLD4
LGALS21	0	1.233561	0.926	0.28	0	cDC	LGALS2
HLA-DQA	0	1.216107	0.313	0.02	0	cDC	HLA-DQA2
HLA-DMB	0	1.150552	0.932	0.227	0	cDC	HLA-DMB
ALDH21	0	1.12411	0.897	0.211	0	cDC	ALDH2
PPA14	0	1.099234	0.894	0.244	0	cDC	PPA1
PRCP	0	0.96293	0.835	0.168	0	cDC	PRCP
CIITA1	0	0.936874	0.73	0.077	0	cDC	CIITA
FLT3	0	0.928421	0.594	0.03	0	cDC	FLT3
PEA151	0	0.87411	0.747	0.133	0	cDC	PEA15
LY862	0	0.868198	0.918	0.259	0	cDC	LY86
IL18	0	0.837636	0.665	0.064	0	cDC	IL18
GSN	0	0.832498	0.896	0.193	0	cDC	GSN
TMEM14C	0	0.827824	0.804	0.175	0	cDC	TMEM14C
RTN1	0	0.824939	0.65	0.068	0	cDC	RTN1
BASP12	0	0.800741	0.73	0.142	0	cDC	BASP1
HLA-DOA	0	0.787225	0.567	0.025	0	cDC	HLA-DOA
AHR1	0	0.776472	0.714	0.133	0	cDC	AHR
SPATS2L	0	0.753229	0.551	0.043	0	cDC	SPATS2L
UVRAG1	0	0.72102	0.765	0.163	0	cDC	UVRAG
NDRG2	0	0.707133	0.479	0.008	0	cDC	NDRG2
PKIB	0	0.706183	0.374	0.004	0	cDC	PKIB
IL13RA11	0	0.705181	0.679	0.116	0	cDC	IL13RA1
AGPAT91	0	0.700317	0.684	0.085	0	cDC	AGPAT9
HMGA1	0	0.694985	0.706	0.133	0	cDC	HMGA1
EIF2AK4	0	0.665932	0.652	0.115	0	cDC	EIF2AK4
CD1D1	0	0.66417	0.692	0.129	0	cDC	CD1D
CD331	0	0.651676	0.823	0.203	0	cDC	CD33
FABP5	0	0.636717	0.541	0.087	0	cDC	FABP5
AFF31	0	0.6312	0.488	0.025	0	cDC	AFF3
FCGR2B2	0	0.629127	0.403	0.052	0	cDC	FCGR2B
CLIC2	0	0.621955	0.469	0.011	0	cDC	CLIC2
CACNA2D	0	0.618748	0.482	0.027	0	cDC	CACNA2D3
MAP4K13	0	0.604924	0.57	0.067	0	cDC	MAP4K1
TMEM109	0	0.596379	0.662	0.133	0	cDC	TMEM109
NREP	0	0.579163	0.502	0.037	0	cDC	NREP
SPINT2	0	0.577318	0.849	0.189	0	cDC	SPINT2
MGST21	0	0.573853	0.673	0.106	0	cDC	MGST2
ITGB72	0	0.573298	0.653	0.115	0	cDC	ITGB7
ADAM281	0	0.57269	0.484	0.026	0	cDC	ADAM28
FAM162A5	0	0.56997	0.664	0.133	0	cDC	FAM162A
RASA4	0	0.564666	0.486	0.058	0	cDC	RASA4
C12orf45	0	0.55058	0.547	0.067	0	cDC	C12orf45
DEGS12	0	0.541999	0.61	0.109	0	cDC	DEGS1
BATF31	0	0.539403	0.423	0.037	0	cDC	BATF3
PON2	0	0.539017	0.479	0.026	0	cDC	PON2
HDAC91	0	0.513383	0.53	0.079	0	cDC	HDAC9
KCNK6	0	0.503699	0.589	0.09	0	cDC	KCNK6
CD1E	0	0.4937	0.281	0.002	0	cDC	CD1E
ETS21	0	0.49144	0.536	0.09	0	cDC	ETS2

ALCAM	0	0.48776	0.462	0.049	0	cDC	ALCAM
IGFBP7	0	0.476846	0.658	0.13	0	cDC	IGFBP7
ATP1B1	0	0.474363	0.387	0.017	0	cDC	ATP1B1
NAV1	0	0.470995	0.352	0.021	0	cDC	NAV1
UPK3A	0	0.458628	0.39	0.03	0	cDC	UPK3A
MYCL	0	0.457831	0.467	0.062	0	cDC	MYCL
PHACTR11	0	0.451031	0.357	0.015	0	cDC	PHACTR1
HIP1	0	0.433645	0.436	0.045	0	cDC	HIP1
BZW21	0	0.425289	0.516	0.087	0	cDC	BZW2
COL9A2	0	0.418755	0.345	0.012	0	cDC	COL9A2
MYO1E1	0	0.413283	0.375	0.041	0	cDC	MYO1E
P2RY6	0	0.409359	0.301	0.007	0	cDC	P2RY6
NIPSNAP3	0	0.402797	0.508	0.087	0	cDC	NIPSNAP3A
CYP2S1	0	0.395315	0.336	0.015	0	cDC	CYP2S1
MAGEF1	0	0.392688	0.389	0.044	0	cDC	MAGEF1
GAS6	0	0.361798	0.313	0.025	0	cDC	GAS6
CD1801	0	0.353563	0.355	0.042	0	cDC	CD180
LGMN	0	0.353094	0.269	0.014	0	cDC	LGMN
MS4A4E	0	0.349327	0.236	0.011	0	cDC	MS4A4E
ARHGAP3	0	0.330284	0.346	0.039	0	cDC	ARHGAP31
HLA-DQB	0	0.326917	0.239	0.008	0	cDC	HLA-DQB2
CRIP3	0	0.326361	0.247	0.004	0	cDC	CRIP3
SERPINF2	0	0.325862	0.214	0.009	0	cDC	SERPINF2
IRF41	0	0.325766	0.289	0.024	0	cDC	IRF4
DST	0	0.318532	0.308	0.033	0	cDC	DST
EPB41L2	0	0.314559	0.278	0.025	0	cDC	EPB41L2
CD200R1	0	0.30933	0.263	0.016	0	cDC	CD200R1
C10orf128	0	0.307096	0.278	0.019	0	cDC	C10orf128
UCK2	0	0.295302	0.274	0.024	0	cDC	UCK2
HLA-DOB	0	0.28627	0.185	0.01	0	cDC	HLA-DOB
P2RY141	0	0.281824	0.245	0.01	0	cDC	P2RY14
GNG71	0	0.281085	0.296	0.025	0	cDC	GNG7
CCSER1	0	0.275041	0.208	0.002	0	cDC	CCSER1
TMEM8B	0	0.272994	0.249	0.021	0	cDC	TMEM8B
TMEM97	0	0.272069	0.21	0.01	0	cDC	TMEM97
LILRB41	#######	0.586402	0.618	0.124	#######	cDC	LILRB4
CD300C2	#######	0.459592	0.561	0.104	#######	cDC	CD300C
BCL11A1	#######	0.305968	0.332	0.041	#######	cDC	BCL11A
STK38L1	#######	0.330638	0.502	0.085	#######	cDC	STK38L
ADAM151	#######	0.488746	0.613	0.123	#######	cDC	ADAM15
SPNS31	#######	0.259135	0.313	0.037	#######	cDC	SPNS3
CCDC501	#######	0.373999	0.502	0.088	#######	cDC	CCDC50
NRROS1	#######	0.558059	0.731	0.167	#######	cDC	NRROS
CPVL1	#######	1.31594	0.948	0.352	#######	cDC	CPVL
ST14	#######	0.258973	0.353	0.047	#######	cDC	ST14
OPN31	#######	0.434863	0.552	0.105	#######	cDC	OPN3
PEBP15	#######	0.738789	0.855	0.235	#######	cDC	PEBP1
PRPF19	#######	0.437042	0.535	0.101	#######	cDC	PRPF19
APEX14	#######	0.766255	0.852	0.243	#######	cDC	APEX1
SEMA4A1	#######	0.325819	0.6	0.123	#######	cDC	SEMA4A
HPS5	#######	0.31213	0.394	0.06	#######	cDC	HPS5
REPIN1	#######	0.361642	0.445	0.075	#######	cDC	REPIN1
C7orf50	#######	0.419246	0.667	0.148	#######	cDC	C7orf50
MLTK1	#######	0.361239	0.483	0.088	#######	cDC	MLTK
CCDC88A	#######	0.792427	0.881	0.264	#######	cDC	CCDC88A
PPP1R14B	#######	0.300237	0.452	0.078	#######	cDC	PPP1R14B
ARHGAP5	#######	0.271422	0.342	0.048	#######	cDC	ARHGAP5
CCNB1IP1	#######	0.293049	0.345	0.049	#######	cDC	CCNB1IP1

RPLP05	#######	1.071242	0.995	0.775	#######	cDC	RPLP0
DCTPP11	#######	0.377402	0.438	0.075	#######	cDC	DCTPP1
WDR41	#######	0.290044	0.389	0.061	#######	cDC	WDR41
RUFY3	#######	0.297556	0.384	0.06	#######	cDC	RUFY3
CLEC4A1	#######	0.481615	0.795	0.208	#######	cDC	CLEC4A
MAN2B12	#######	0.633918	0.815	0.223	#######	cDC	MAN2B1
CAMK1D2	#######	0.407067	0.55	0.112	#######	cDC	CAMK1D
RAB341	#######	0.346762	0.465	0.085	#######	cDC	RAB34
UBE2E22	#######	0.386762	0.611	0.134	#######	cDC	UBE2E2
PCBD1	#######	0.36811	0.493	0.094	#######	cDC	PCBD1
MRPL31	#######	0.480469	0.63	0.142	#######	cDC	MRPL3
TRAP1	#######	0.267738	0.352	0.052	#######	cDC	TRAP1
BCKDHA	#######	0.317977	0.459	0.083	#######	cDC	BCKDHA
TCTN3	#######	0.309473	0.314	0.043	#######	cDC	TCTN3
IGFLR1	#######	0.429138	0.62	0.138	#######	cDC	IGFLR1
IMPA21	#######	0.458293	0.624	0.142	#######	cDC	IMPA2
RNH11	#######	0.908043	0.938	0.365	#######	cDC	RNH1
CTSZ2	#######	1.074662	0.965	0.424	#######	cDC	CTSZ
PTRHD11	#######	0.604721	0.833	0.236	#######	cDC	PTRHD1
C20orf272	#######	0.583755	0.813	0.228	#######	cDC	C20orf27
SLC1A51	#######	0.385974	0.47	0.089	#######	cDC	SLC1A5
LRRK1	#######	0.259571	0.37	0.059	#######	cDC	LRRK1
CCDC6	#######	0.322361	0.478	0.091	#######	cDC	CCDC6
AHCY1	#######	0.372513	0.444	0.081	#######	cDC	AHCY
NME1-NM	#######	1.072148	0.977	0.561	#######	cDC	NME1-NME2
DSE	#######	0.325787	0.56	0.121	#######	cDC	DSE
SLC25A51	#######	1.070506	0.969	0.501	#######	cDC	SLC25A5
LMNA	#######	0.693669	0.356	0.057	#######	cDC	LMNA
DUSP231	#######	0.444135	0.726	0.184	#######	cDC	DUSP23
SYNGR22	#######	0.891023	0.937	0.364	#######	cDC	SYNGR2
AD000671	#######	0.321105	0.44	0.08	#######	cDC	AD000671.6
DECR11	#######	0.595307	0.828	0.237	#######	cDC	DECR1
CD862	#######	0.474927	0.742	0.193	#######	cDC	CD86
FGD23	#######	0.463223	0.673	0.165	#######	cDC	FGD2
HAVCR2	#######	0.27466	0.411	0.073	#######	cDC	HAVCR2
IDH3A	#######	0.321702	0.409	0.073	#######	cDC	IDH3A
MTMR141	#######	0.473049	0.713	0.187	#######	cDC	MTMR14
SEC13	#######	0.535532	0.779	0.214	#######	cDC	SEC13
SLC38A15	#######	0.380917	0.541	0.114	#######	cDC	SLC38A1
IMPDH24	#######	0.402141	0.569	0.126	#######	cDC	IMPDH2
C1QBP5	#######	0.683761	0.87	0.279	#######	cDC	C1QBP
BTN2A21	#######	0.270114	0.282	0.039	#######	cDC	BTN2A2
CAT2	#######	0.6954	0.853	0.277	#######	cDC	CAT
PAK12	#######	0.755304	0.935	0.333	#######	cDC	PAK1
SAMHD11	#######	1.114991	0.986	0.59	#######	cDC	SAMHD1
RNASE61	#######	0.573065	0.803	0.228	#######	cDC	RNASE6
ADAM8	#######	0.290595	0.586	0.134	#######	cDC	ADAM8
CTSH1	#######	0.749736	0.932	0.336	#######	cDC	CTSH
CTNND11	#######	0.25917	0.36	0.061	#######	cDC	CTNND1
HDAC3	#######	0.299029	0.531	0.115	#######	cDC	HDAC3
PPIA5	#######	0.913149	0.985	0.73	#######	cDC	PPIA
CYC11	#######	0.57795	0.792	0.231	#######	cDC	CYC1
MRPS15	#######	0.395229	0.672	0.169	#######	cDC	MRPS15
MTMR11	#######	0.355726	0.382	0.069	#######	cDC	MTMR11
ACAA11	#######	0.537832	0.741	0.209	#######	cDC	ACAA1
WDFY41	#######	0.338995	0.326	0.052	#######	cDC	WDFY4
PPA2	#######	0.321388	0.502	0.107	#######	cDC	PPA2
CTNNBIP1	#######	0.259534	0.547	0.122	#######	cDC	CTNNBIP1

SPECC11	#######	0.302717	0.543	0.123	#######	cDC	SPECC1
ELMO1	#######	0.332445	0.519	0.114	#######	cDC	ELMO1
LGALS92	#######	0.569764	0.862	0.268	#######	cDC	LGALS9
MTCH2	#######	0.386794	0.672	0.172	#######	cDC	MTCH2
RPL155	#######	0.814623	0.989	0.799	#######	cDC	RPL15
TNFAIP8L2	#######	0.451431	0.701	0.189	#######	cDC	TNFAIP8L2
ATXN102	#######	0.435065	0.689	0.182	#######	cDC	ATXN10
CSF2RA1	#######	0.316711	0.55	0.126	#######	cDC	CSF2RA
IL1B	#######	0.304562	0.242	0.031	#######	cDC	IL1B
NIT2	#######	0.26183	0.358	0.062	#######	cDC	NIT2
AIMP2	#######	0.28409	0.425	0.083	#######	cDC	AIMP2
ANXA22	#######	1.066564	0.962	0.509	#######	cDC	ANXA2
ZNF385A1	#######	0.489849	0.795	0.232	#######	cDC	ZNF385A
EEF1E1	#######	0.276313	0.42	0.082	#######	cDC	EEF1E1
OSTC1	#######	0.600566	0.821	0.258	#######	cDC	OSTC
FAM105A1	#######	0.36012	0.64	0.162	#######	cDC	FAM105A
ETHE11	#######	0.444599	0.709	0.195	#######	cDC	ETHE1
SAMM50	#######	0.3281	0.522	0.117	#######	cDC	SAMM50
CXorf211	#######	0.267993	0.434	0.088	#######	cDC	CXorf21
ETV61	#######	0.359861	0.756	0.215	#######	cDC	ETV6
SNRNP25	#######	0.265568	0.379	0.07	#######	cDC	SNRNP25
OXA1L1	#######	0.641409	0.83	0.273	#######	cDC	OXA1L
RPS217	#######	0.824266	0.99	0.814	#######	cDC	RPS2
DNPH13	#######	0.361472	0.536	0.124	#######	cDC	DNPH1
EIF3L4	#######	0.898243	0.971	0.535	#######	cDC	EIF3L
ELOVL11	#######	0.380849	0.633	0.164	#######	cDC	ELOVL1
ATP2B12	#######	0.593239	0.78	0.24	#######	cDC	ATP2B1
WDFY2	#######	0.3159	0.413	0.082	#######	cDC	WDFY2
FNIP21	#######	0.283505	0.317	0.053	#######	cDC	FNIP2
OLA1	#######	0.389286	0.659	0.173	#######	cDC	OLA1
MRPL15	#######	0.294305	0.424	0.086	#######	cDC	MRPL15
MLEC	#######	0.44017	0.776	0.23	#######	cDC	MLEC
HMGN15	#######	0.75094	0.893	0.351	#######	cDC	HMGN1
PTMS1	#######	0.267881	0.342	0.061	#######	cDC	PTMS
GLB1	#######	0.293342	0.575	0.14	#######	cDC	GLB1
WBSCR222	#######	0.316295	0.501	0.113	#######	cDC	WBSCR22
LSP12	#######	0.904383	0.984	0.666	#######	cDC	LSP1
DNTTIP11	#######	0.271051	0.561	0.135	#######	cDC	DNTTIP1
H2AFY1	#######	0.816785	0.96	0.435	#######	cDC	H2AFY
DDOST1	#######	0.517417	0.775	0.235	#######	cDC	DDOST
RPS235	#######	0.791205	0.999	0.823	#######	cDC	RPS23
UNC1191	#######	0.351694	0.618	0.159	#######	cDC	UNC119
C19orf10	#######	0.518372	0.762	0.227	#######	cDC	C19orf10
SRM3	#######	0.381339	0.665	0.178	#######	cDC	SRM
GGCT	#######	0.268744	0.477	0.105	#######	cDC	GGCT
TUBA1B1	#######	0.845086	0.947	0.436	#######	cDC	TUBA1B
EXOSC5	#######	0.302807	0.444	0.094	#######	cDC	EXOSC5
CALHM21	#######	0.392285	0.532	0.129	#######	cDC	CALHM2
ABI32	#######	0.396459	0.689	0.189	#######	cDC	ABI3
KDM2B	#######	0.268032	0.358	0.067	#######	cDC	KDM2B
WDR771	#######	0.255899	0.374	0.072	#######	cDC	WDR77
VIM1	#######	1.090162	0.997	0.836	#######	cDC	VIM
VDAC11	#######	0.540096	0.805	0.257	#######	cDC	VDAC1
COMMD9	#######	0.33239	0.574	0.143	#######	cDC	COMMD9
CFP2	#######	0.817142	0.928	0.38	#######	cDC	CFP
NUDT1	#######	0.323755	0.566	0.143	#######	cDC	NUDT1
WDR61	#######	0.32968	0.535	0.129	#######	cDC	WDR61
SHMT21	#######	0.315992	0.501	0.116	#######	cDC	SHMT2

MRPS24	#######	0.418872	0.795	0.244	#######	cDC	MRPS24
CNPPD12	#######	0.350772	0.657	0.18	#######	cDC	CNPPD1
C1orf1622	#######	0.834602	0.961	0.462	#######	cDC	C1orf162
ELOVL52	#######	0.374417	0.652	0.18	#######	cDC	ELOVL5
ATP5B1	#######	0.80895	0.952	0.482	#######	cDC	ATP5B
PID11	#######	0.353353	0.361	0.071	#######	cDC	PID1
SCNM11	#######	0.342301	0.733	0.214	#######	cDC	SCNM1
EEF1G5	#######	0.972667	0.919	0.518	#######	cDC	EEF1G
MPC2	#######	0.279656	0.58	0.149	#######	cDC	MPC2
CLNS1A3	#######	0.522728	0.789	0.253	#######	cDC	CLNS1A
NUBP1	#######	0.275274	0.454	0.102	#######	cDC	NUBP1
HSD17B1C	#######	0.381333	0.663	0.186	#######	cDC	HSD17B10
MRPL4	#######	0.297862	0.562	0.142	#######	cDC	MRPL4
KIAA1598	#######	0.342523	0.611	0.165	#######	cDC	KIAA1598
SLC25A62	#######	0.864296	0.979	0.687	#######	cDC	SLC25A6
GDI22	#######	0.791274	0.938	0.425	#######	cDC	GDI2
YBX11	#######	0.842715	0.981	0.68	#######	cDC	YBX1
ATP5A11	#######	0.756131	0.941	0.43	#######	cDC	ATP5A1
IRF52	#######	0.32632	0.55	0.14	#######	cDC	IRF5
TPMT1	#######	0.297872	0.386	0.08	#######	cDC	TPMT
SNRPF4	#######	0.582281	0.831	0.283	#######	cDC	SNRPF
ARL5A	#######	0.428626	0.712	0.216	#######	cDC	ARL5A
SULF21	#######	0.256193	0.669	0.188	#######	cDC	SULF2
GRSF1	#######	0.383403	0.701	0.205	#######	cDC	GRSF1
FIBP	#######	0.35493	0.711	0.209	#######	cDC	FIBP
LPXN1	#######	0.351295	0.648	0.18	#######	cDC	LPXN
SNX20	#######	0.285145	0.576	0.151	#######	cDC	SNX20
APH1A1	#######	0.476579	0.774	0.245	#######	cDC	APH1A
PHB1	#######	0.356087	0.655	0.184	#######	cDC	PHB
ECHS1	#######	0.269961	0.609	0.162	#######	cDC	ECHS1
DNAJC4	#######	0.303466	0.745	0.227	#######	cDC	DNAJC4
PDCL31	#######	0.263226	0.455	0.105	#######	cDC	PDCL3
CNDP21	#######	0.386322	0.625	0.174	#######	cDC	CNDP2
LAP31	#######	0.375179	0.74	0.228	#######	cDC	LAP3
ABCE11	#######	0.258322	0.439	0.098	#######	cDC	ABCE1
NDUFS4	#######	0.291189	0.56	0.146	#######	cDC	NDUFS4
CCT25	#######	0.41274	0.687	0.202	#######	cDC	CCT2
RBPJ1	#######	0.398785	0.81	0.267	#######	cDC	RBPJ
HSPD15	#######	0.484956	0.794	0.262	#######	cDC	HSPD1
NDUFS8	#######	0.455533	0.849	0.289	#######	cDC	NDUFS8
SRSF21	#######	0.494807	0.801	0.267	#######	cDC	SRSF2
NANS2	#######	0.30096	0.523	0.131	#######	cDC	NANS
ATP5G1	#######	0.401698	0.821	0.267	#######	cDC	ATP5G1
AMICA11	#######	0.663381	0.95	0.418	#######	cDC	AMICA1
NAGA2	#######	0.284329	0.565	0.149	#######	cDC	NAGA
APPL11	#######	0.359013	0.711	0.215	#######	cDC	APPL1
MPV17	#######	0.273997	0.516	0.129	#######	cDC	MPV17
MRPL9	#######	0.251394	0.428	0.097	#######	cDC	MRPL9
NSMAF	#######	0.253824	0.394	0.086	#######	cDC	NSMAF
CAPG1	#######	0.671921	0.919	0.367	#######	cDC	CAPG
RPS185	#######	0.74917	0.996	0.786	#######	cDC	RPS18
CCT6A1	#######	0.501208	0.839	0.289	#######	cDC	CCT6A
NDUFB51	#######	0.451347	0.8	0.264	#######	cDC	NDUFB5
MZT2A5	#######	0.383348	0.635	0.183	#######	cDC	MZT2A
RPS6KA41	#######	0.281326	0.533	0.139	#######	cDC	RPS6KA4
JAK21	#######	0.283587	0.604	0.17	#######	cDC	JAK2
PRDX31	#######	0.416173	0.795	0.264	#######	cDC	PRDX3
IL6R1	#######	0.267559	0.684	0.204	#######	cDC	IL6R

RPS243	#######	0.586989	0.999	0.882	#######	cDC	RPS24
ACTG1	#######	0.579771	0.996	0.878	#######	cDC	ACTG1
ATP5C11	#######	0.566356	0.907	0.358	#######	cDC	ATP5C1
TIMM17A	#######	0.264388	0.477	0.117	#######	cDC	TIMM17A
HEXB1	#######	0.274312	0.717	0.215	#######	cDC	HEXB
CLEC11A	#######	0.297998	0.34	0.07	#######	cDC	CLEC11A
RPS65	#######	0.730695	0.99	0.78	#######	cDC	RPS6
DPYSL21	#######	0.277046	0.716	0.219	#######	cDC	DPYSL2
RPS55	#######	0.739054	0.991	0.702	#######	cDC	RPS5
EIF4A12	#######	0.789149	0.977	0.625	#######	cDC	EIF4A1
NFKB1	#######	0.32934	0.624	0.183	#######	cDC	NFKB1
NDUFV22	#######	0.502422	0.86	0.318	#######	cDC	NDUFV2
TXN2	#######	0.332105	0.713	0.221	#######	cDC	TXN2
SSR11	#######	0.489183	0.828	0.292	#######	cDC	SSR1
SNRPA3	#######	0.31512	0.668	0.197	#######	cDC	SNRPA
HINT15	#######	0.78399	0.976	0.606	#######	cDC	HINT1
SDHD3	#######	0.367234	0.657	0.198	#######	cDC	SDHD
SLC25A31	#######	0.758728	0.96	0.557	#######	cDC	SLC25A3
TREX11	#######	0.269821	0.591	0.167	#######	cDC	TREX1
NDUFS3	#######	0.320998	0.698	0.21	#######	cDC	NDUFS3
HDAC2	#######	0.280849	0.61	0.174	#######	cDC	HDAC2
SPOP	#######	0.263862	0.592	0.168	#######	cDC	SPOP
NDUFA10	#######	0.309613	0.668	0.201	#######	cDC	NDUFA10
OSGEP1	#######	0.254207	0.472	0.118	#######	cDC	OSGEP
FBL5	#######	0.439737	0.814	0.279	#######	cDC	FBL
RPL266	#######	0.745861	0.986	0.804	#######	cDC	RPL26
EEF1B25	#######	0.742455	0.985	0.669	#######	cDC	EEF1B2
EIF61	#######	0.337078	0.719	0.226	#######	cDC	EIF6
RPL55	#######	0.662681	0.994	0.798	#######	cDC	RPL5
RPL10A5	#######	0.754014	0.982	0.685	#######	cDC	RPL10A
NHP24	#######	0.386074	0.764	0.25	#######	cDC	NHP2
RTFDC1	#######	0.297078	0.757	0.245	#######	cDC	RTFDC1
PHB25	#######	0.524666	0.879	0.34	#######	cDC	PHB2
ATP5G32	#######	0.687573	0.952	0.487	#######	cDC	ATP5G3
UBL7	#######	0.251252	0.575	0.163	#######	cDC	UBL7
COMMD8	#######	0.336704	0.603	0.174	#######	cDC	COMMD8
SSRP1	#######	0.26387	0.503	0.135	#######	cDC	SSRP1
EZR4	#######	0.51214	0.785	0.282	#######	cDC	EZR
RAB7A	#######	0.474894	0.892	0.354	#######	cDC	RAB7A
RPL86	#######	0.627427	0.995	0.856	#######	cDC	RPL8
IRF81	#######	0.351085	0.447	0.115	#######	cDC	IRF8
LSM53	#######	0.35186	0.668	0.205	#######	cDC	LSM5
COX5A2	#######	0.629417	0.962	0.481	#######	cDC	COX5A
SNRPA11	#######	0.306773	0.47	0.122	#######	cDC	SNRPA1
CRTAP1	#######	0.429528	0.865	0.318	#######	cDC	CRTAP
PFDN2	#######	0.36257	0.733	0.24	#######	cDC	PFDN2
ODF3B2	#######	0.265184	0.649	0.197	#######	cDC	ODF3B
VPS352	#######	0.390758	0.818	0.294	#######	cDC	VPS35
SNRPD1	#######	0.342839	0.717	0.231	#######	cDC	SNRPD1
RPSA5	#######	0.721766	0.987	0.695	#######	cDC	RPSA
PSMC42	#######	0.280468	0.585	0.17	#######	cDC	PSMC4
IAH1	#######	0.278071	0.548	0.154	#######	cDC	IAH1
PRMT14	#######	0.272106	0.57	0.164	#######	cDC	PRMT1
TNFSF132	#######	0.260456	0.464	0.124	#######	cDC	TNFSF13
NDUFV11	#######	0.301673	0.714	0.23	#######	cDC	NDUFV1
GNB2L15	#######	0.641841	0.992	0.797	#######	cDC	GNB2L1
ARF51	#######	0.577212	0.962	0.496	#######	cDC	ARF5
UQCRC21	#######	0.326147	0.704	0.227	#######	cDC	UQCRC2

CDC1232	#######	0.252844	0.537	0.149	#######	cDC	CDC123
MYD882	#######	0.291787	0.707	0.23	#######	cDC	MYD88
TOMM22	#######	0.413676	0.774	0.27	#######	cDC	TOMM22
SDHC2	#######	0.299977	0.684	0.218	#######	cDC	SDHC
ESD	#######	0.263804	0.672	0.21	#######	cDC	ESD
AAMP1	#######	0.26461	0.577	0.169	#######	cDC	AAMP
CCT75	#######	0.337697	0.708	0.228	#######	cDC	CCT7
GSTP12	#######	0.741354	0.99	0.67	#######	cDC	GSTP1
CCT35	#######	0.3448	0.717	0.237	#######	cDC	CCT3
SUCLG1	#######	0.254038	0.672	0.21	#######	cDC	SUCLG1
POLR2E	#######	0.282328	0.796	0.279	#######	cDC	POLR2E
ILF23	#######	0.342382	0.733	0.245	#######	cDC	ILF2
GPRIN32	#######	0.250547	0.406	0.101	#######	cDC	GPRIN3
TTC33	#######	0.358762	0.678	0.223	#######	cDC	TTC3
PSMG2	#######	0.272943	0.735	0.239	#######	cDC	PSMG2
SRP72	#######	0.333063	0.712	0.235	#######	cDC	SRP72
EEF1A16	#######	0.584635	0.999	0.891	#######	cDC	EEF1A1
RBM31	#######	0.657681	0.935	0.46	#######	cDC	RBM3
FCGRT2	#######	0.495483	0.93	0.378	#######	cDC	FCGRT
MYADM1	#######	0.505845	0.719	0.256	#######	cDC	MYADM
TUFM	#######	0.441703	0.833	0.316	#######	cDC	TUFM
CAPN2	#######	0.371033	0.782	0.281	#######	cDC	CAPN2
MDH21	#######	0.468114	0.872	0.345	#######	cDC	MDH2
EIF3F5	#######	0.682787	0.969	0.536	#######	cDC	EIF3F
EIF2A2	#######	0.265928	0.613	0.189	#######	cDC	EIF2A
LAMTOR2	#######	0.346435	0.849	0.32	#######	cDC	LAMTOR2
RPL18A5	#######	0.567472	0.996	0.825	#######	cDC	RPL18A
FDFT11	#######	0.351971	0.647	0.21	#######	cDC	FDFT1
CRIP13	#######	1.172752	0.933	0.522	#######	cDC	CRIP1
LSM41	#######	0.306095	0.702	0.234	#######	cDC	LSM4
BTF35	#######	0.641912	0.99	0.739	#######	cDC	BTF3
HSPA91	#######	0.251461	0.613	0.191	#######	cDC	HSPA9
CCT81	#######	0.433859	0.805	0.301	#######	cDC	CCT8
PHPT1	#######	0.279407	0.697	0.229	#######	cDC	PHPT1
MDH13	#######	0.301365	0.693	0.227	#######	cDC	MDH1
NDUFS2	#######	0.334089	0.714	0.24	#######	cDC	NDUFS2
ACTB	#######	0.264032	1	0.993	#######	cDC	ACTB
VDAC21	#######	0.379932	0.804	0.29	#######	cDC	VDAC2
GRN1	#######	0.669204	0.972	0.484	#######	cDC	GRN
C14orf166	#######	0.461363	0.868	0.348	#######	cDC	C14orf166
MEF2C2	#######	0.253597	0.874	0.333	#######	cDC	MEF2C
LINC00493	#######	0.324506	0.828	0.305	#######	cDC	LINC00493
CPPED12	#######	0.261902	0.736	0.249	#######	cDC	CPPED1
BID1	#######	0.259453	0.727	0.247	#######	cDC	BID
PARVG2	#######	0.340252	0.854	0.329	#######	cDC	PARVG
CSTB2	#######	0.62246	0.967	0.534	#######	cDC	CSTB
C1orf54	#######	0.362031	0.164	0.024	#######	cDC	C1orf54
RPS155	#######	0.537346	0.994	0.865	#######	cDC	RPS15
IGSF62	#######	0.474902	0.897	0.371	#######	cDC	IGSF6
TRAPPC6A	#######	0.276178	0.508	0.147	#######	cDC	TRAPPC6A
NAPSA	#######	0.406761	0.112	0.012	#######	cDC	NAPSA
EIF3I	#######	0.357592	0.787	0.286	#######	cDC	EIF3I
ATP5F1	#######	0.559598	0.932	0.455	#######	cDC	ATP5F1
HADHA1	#######	0.509624	0.912	0.409	#######	cDC	HADHA
ATG32	#######	0.339733	0.857	0.329	#######	cDC	ATG3
UBE2N3	#######	0.25203	0.601	0.188	#######	cDC	UBE2N
RPS3A5	#######	0.527559	0.996	0.856	#######	cDC	RPS3A
EIF4H	#######	0.345498	0.732	0.255	#######	cDC	EIF4H

FAM96A3	#######	0.282767	0.761	0.272	#######	cDC	FAM96A
LDHA3	#######	0.470884	0.883	0.381	#######	cDC	LDHA
IDH22	#######	0.251419	0.626	0.203	#######	cDC	IDH2
PPM1G1	#######	0.304848	0.786	0.288	#######	cDC	PPM1G
ALKBH72	#######	0.311573	0.826	0.309	#######	cDC	ALKBH7
RPL317	#######	0.595233	0.987	0.743	#######	cDC	RPL3
NDUFA12:	#######	0.479379	0.873	0.359	#######	cDC	NDUFA12
SRSF3	#######	0.434849	0.908	0.399	#######	cDC	SRSF3
FAM49B	#######	0.394862	0.899	0.381	#######	cDC	FAM49B
EIF3D4	#######	0.429372	0.813	0.319	#######	cDC	EIF3D
RPL195	#######	0.491894	0.996	0.868	#######	cDC	RPL19
S100A101	#######	0.831852	0.987	0.768	#######	cDC	S100A10
SSR31	#######	0.329984	0.828	0.313	#######	cDC	SSR3
AKR1A11	#######	0.255738	0.743	0.263	#######	cDC	AKR1A1
HMGN31	#######	0.38308	0.864	0.346	#######	cDC	HMGN3
DNAJC151	#######	0.267683	0.854	0.338	#######	cDC	DNAJC15
HSPE15	#######	0.30989	0.686	0.241	#######	cDC	HSPE1
CNN25	#######	0.368506	0.879	0.379	#######	cDC	CNN2
HNRNPA1	#######	0.702665	0.971	0.625	#######	cDC	HNRNPA1
DOCK101	#######	0.255713	0.468	0.138	#######	cDC	DOCK10
RPL355	#######	0.588117	0.989	0.768	#######	cDC	RPL35
RPL45	#######	0.614197	0.976	0.72	#######	cDC	RPL4
RPS4X5	#######	0.522422	0.995	0.81	#######	cDC	RPS4X
PDIA61	#######	0.261471	0.74	0.268	#######	cDC	PDIA6
NDUFAB1	#######	0.328433	0.865	0.342	#######	cDC	NDUFAB1
PSMD82	#######	0.276313	0.808	0.302	#######	cDC	PSMD8
NPM15	#######	0.54964	0.967	0.519	#######	cDC	NPM1
ANXA11	#######	0.790539	0.958	0.582	#######	cDC	ANXA1
UQCRFS1	#######	0.350732	0.825	0.33	#######	cDC	UQCRFS1
EIF3E4	#######	0.566843	0.94	0.485	#######	cDC	EIF3E
RPL7A5	#######	0.516283	0.995	0.837	#######	cDC	RPL7A
BANF11	#######	0.30753	0.825	0.322	#######	cDC	BANF1
RPL36A6	#######	0.615124	0.98	0.628	#######	cDC	RPL36A
VPS291	#######	0.294627	0.834	0.325	#######	cDC	VPS29
EEF24	#######	0.645406	0.972	0.695	#######	cDC	EEF2
RPS75	#######	0.49111	0.997	0.863	#######	cDC	RPS7
COX7A2L	#######	0.36832	0.858	0.353	#######	cDC	COX7A2L
SMDT14	#######	0.383999	0.893	0.405	#######	cDC	SMDT1
ID22	#######	0.305329	0.642	0.236	#######	cDC	ID2
RSL1D14	#######	0.366396	0.864	0.365	#######	cDC	RSL1D1
EIF5A4	#######	0.437092	0.892	0.399	#######	cDC	EIF5A
CASP12	#######	0.371136	0.855	0.366	#######	cDC	CASP1
SUB11	#######	0.5214	0.962	0.548	#######	cDC	SUB1
HNRNPA0	#######	0.316384	0.747	0.285	#######	cDC	HNRNPA0
RPS84	#######	0.474691	1	0.877	1.21E-99	cDC	RPS8
HNRNPL2	#######	0.264419	0.775	0.298	1.43E-99	cDC	HNRNPL
EIF3H4	#######	0.5332	0.958	0.522	2.11E-99	cDC	EIF3H
GLIPR11	#######	0.431191	0.93	0.437	3.33E-98	cDC	GLIPR1
LGALS12	#######	0.666022	0.976	0.617	1.07E-97	cDC	LGALS1
RPL37A5	#######	0.54186	0.991	0.806	8.99E-97	cDC	RPL37A
SNRPB2	#######	0.304187	0.803	0.318	1.05E-96	cDC	SNRPB
SNX24	#######	0.251678	0.752	0.285	2.37E-96	cDC	SNX2
PSMA61	#######	0.273661	0.813	0.326	6.01E-96	cDC	PSMA6
SRSF75	#######	0.311965	0.825	0.337	9.97E-96	cDC	SRSF7
DBI	#######	0.517729	0.938	0.491	1.36E-95	cDC	DBI
HNRNPC	7.11E-99	0.444375	0.926	0.452	2.33E-94	cDC	HNRNPC
AP1S21	1.03E-98	0.471056	0.969	0.503	3.38E-94	cDC	AP1S2
SFPQ	1.70E-98	0.356492	0.82	0.347	5.56E-94	cDC	SFPQ

PSMB6	5.94E-98	0.311243	0.844	0.346	1.94E-93	cDC	PSMB6
RPL375	1.57E-97	0.461396	0.996	0.866	5.13E-93	cDC	RPL37
XRCC63	2.24E-97	0.307721	0.842	0.354	7.32E-93	cDC	XRCC6
NDUFS71	5.46E-97	0.291573	0.821	0.335	1.79E-92	cDC	NDUFS7
RPS35	5.67E-97	0.394951	0.994	0.83	1.86E-92	cDC	RPS3
ECH11	1.24E-96	0.272088	0.741	0.282	4.05E-92	cDC	ECH1
HSP90AB1	3.23E-95	0.511081	0.964	0.546	1.06E-90	cDC	HSP90AB1
TPI12	7.72E-95	0.477888	0.977	0.618	2.53E-90	cDC	TPI1
RPL145	1.12E-94	0.431427	0.992	0.809	3.68E-90	cDC	RPL14
TMSB102	2.19E-94	0.432758	1	0.909	7.17E-90	cDC	TMSB10
UFC11	9.69E-94	0.299309	0.836	0.355	3.17E-89	cDC	UFC1
TAF151	7.54E-93	0.282027	0.701	0.271	2.47E-88	cDC	TAF15
BRK1	6.54E-92	0.367488	0.947	0.518	2.14E-87	cDC	BRK1
CPNE3	6.81E-92	0.347249	0.634	0.238	2.23E-87	cDC	CPNE3
SSBP14	4.83E-90	0.264236	0.767	0.306	1.58E-85	cDC	SSBP1
RPL295	2.00E-89	0.44362	0.995	0.833	6.53E-85	cDC	RPL29
RPL175	7.74E-89	0.562456	0.981	0.712	2.53E-84	cDC	RPL17
RPS165	1.01E-88	0.501871	0.989	0.763	3.32E-84	cDC	RPS16
RAN5	9.16E-88	0.380561	0.918	0.447	3.00E-83	cDC	RAN
PRELID12	1.65E-87	0.45053	0.962	0.551	5.41E-83	cDC	PRELID1
RPS112	7.47E-87	0.495299	0.982	0.751	2.44E-82	cDC	RPS11
PPIB1	1.90E-86	0.469539	0.971	0.584	6.24E-82	cDC	PPIB
CNPY31	3.35E-86	0.319727	0.948	0.473	1.10E-81	cDC	CNPY3
RNF1301	7.14E-86	0.348704	0.918	0.449	2.34E-81	cDC	RNF130
PCBP24	1.62E-85	0.497202	0.974	0.635	5.30E-81	cDC	PCBP2
SNX31	9.69E-85	0.40929	0.94	0.499	3.17E-80	cDC	SNX3
PSMB1	1.52E-84	0.350564	0.907	0.429	4.97E-80	cDC	PSMB1
RNASET22	2.37E-84	0.285104	0.94	0.455	7.75E-80	cDC	RNASET2
COTL12	3.52E-84	0.520123	0.991	0.721	1.15E-79	cDC	COTL1
NCL5	4.42E-84	0.466264	0.94	0.492	1.45E-79	cDC	NCL
C8orf591	1.01E-83	0.27432	0.912	0.427	3.32E-79	cDC	C8orf59
ATP5O5	1.36E-83	0.403576	0.945	0.483	4.44E-79	cDC	ATP5O
RPL105	1.46E-83	0.384674	1	0.878	4.79E-79	cDC	RPL10
RPL65	3.33E-83	0.428002	0.994	0.863	1.09E-78	cDC	RPL6
PFN12	1.47E-82	0.358918	0.994	0.911	4.82E-78	cDC	PFN1
COX7B1	1.74E-82	0.367379	0.914	0.452	5.68E-78	cDC	COX7B
HSP90B11	2.65E-82	0.406872	0.921	0.467	8.68E-78	cDC	HSP90B1
NDUFB4	5.13E-82	0.3039	0.882	0.406	1.68E-77	cDC	NDUFB4
EIF3M1	3.59E-81	0.296722	0.897	0.426	1.18E-76	cDC	EIF3M
RPL23A5	3.69E-81	0.482117	0.986	0.737	1.21E-76	cDC	RPL23A
HNRNPF2	4.42E-81	0.300614	0.848	0.381	1.45E-76	cDC	HNRNPF
RPL325	3.07E-80	0.319358	0.999	0.869	1.00E-75	cDC	RPL32
FGL22	6.71E-80	0.323774	0.96	0.462	2.20E-75	cDC	FGL2
PGLS2	1.60E-79	0.291078	0.956	0.497	5.25E-75	cDC	PGLS
EIF3K1	1.61E-78	0.446496	0.984	0.696	5.26E-74	cDC	EIF3K
SPI12	2.19E-78	0.436872	0.969	0.497	7.16E-74	cDC	SPI1
MT-ND63	5.39E-78	0.411606	0.818	0.409	1.76E-73	cDC	MT-ND6
SET2	1.18E-77	0.398203	0.937	0.515	3.87E-73	cDC	SET
CALM21	1.47E-77	0.489867	0.961	0.665	4.81E-73	cDC	CALM2
NDUFA41	1.49E-77	0.322026	0.948	0.531	4.88E-73	cDC	NDUFA4
SNRPG2	2.12E-76	0.269071	0.902	0.416	6.94E-72	cDC	SNRPG
CANX1	1.92E-75	0.304567	0.834	0.385	6.29E-71	cDC	CANX
RPS105	2.54E-75	0.460054	0.979	0.761	8.32E-71	cDC	RPS10
RPS196	3.97E-75	0.389868	0.999	0.813	1.30E-70	cDC	RPS19
RPS255	4.60E-75	0.336212	0.986	0.773	1.51E-70	cDC	RPS25
RPL318	4.96E-75	0.466099	0.979	0.732	1.62E-70	cDC	RPL31
SRSF9	2.82E-74	0.320222	0.909	0.452	9.24E-70	cDC	SRSF9
HIGD2A2	3.24E-74	0.393824	0.974	0.597	1.06E-69	cDC	HIGD2A

MT-ND4L5	1.18E-73	0.452334	0.986	0.772	3.86E-69	cDC	MT-ND4L
RPS15A5	2.24E-73	0.316393	0.997	0.867	7.34E-69	cDC	RPS15A
RPS295	4.89E-73	0.372833	0.981	0.763	1.60E-68	cDC	RPS29
CORO1A2	1.00E-72	0.468492	0.979	0.729	3.27E-68	cDC	CORO1A
ACTR31	1.61E-72	0.380906	0.937	0.528	5.26E-68	cDC	ACTR3
SEC11A2	2.56E-72	0.286618	0.898	0.432	8.39E-68	cDC	SEC11A
EID1	1.24E-71	0.292256	0.911	0.463	4.04E-67	cDC	EID1
RPL275	1.29E-70	0.470135	0.981	0.734	4.23E-66	cDC	RPL27
RPL185	1.67E-70	0.364653	0.994	0.849	5.45E-66	cDC	RPL18
NDUFA131	1.21E-69	0.293646	0.909	0.467	3.96E-65	cDC	NDUFA13
MZT2B5	1.65E-69	0.294218	0.932	0.48	5.40E-65	cDC	MZT2B
RPL365	9.26E-69	0.361352	0.994	0.829	3.03E-64	cDC	RPL36
SHFM11	2.15E-68	0.256367	0.912	0.445	7.05E-64	cDC	SHFM1
MTDH1	2.55E-68	0.254415	0.945	0.51	8.35E-64	cDC	MTDH
RPS218	4.13E-68	0.330795	0.995	0.834	1.35E-63	cDC	RPS21
MT-ATP86	4.35E-68	0.497992	0.965	0.734	1.42E-63	cDC	MT-ATP8
RPL231	4.79E-68	0.427713	0.977	0.66	1.57E-63	cDC	RPL23
NACA6	1.92E-67	0.424489	0.99	0.819	6.27E-63	cDC	NACA
EIF4B4	3.98E-66	0.287555	0.888	0.44	1.30E-61	cDC	EIF4B
RPL135	2.18E-65	0.303991	0.994	0.861	7.13E-61	cDC	RPL13
GAPDH1	2.53E-65	0.347385	1	0.922	8.28E-61	cDC	GAPDH
SERBP1	1.14E-64	0.289608	0.927	0.498	3.72E-60	cDC	SERBP1
VAMP81	1.40E-64	0.333776	0.964	0.58	4.59E-60	cDC	VAMP8
RPL115	1.62E-64	0.316162	0.997	0.882	5.31E-60	cDC	RPL11
ATP5J21	2.52E-63	0.299496	0.964	0.587	8.26E-59	cDC	ATP5J2
LYZ1	3.75E-63	0.56348	0.997	0.677	1.23E-58	cDC	LYZ
RPL27A4	1.15E-59	0.380489	0.964	0.641	3.77E-55	cDC	RPL27A
ERP292	1.58E-59	0.306045	0.943	0.54	5.16E-55	cDC	ERP29
CFL1	5.81E-59	0.26098	0.99	0.879	1.90E-54	cDC	CFL1
RPS134	2.12E-58	0.328504	0.997	0.887	6.95E-54	cDC	RPS13
HSPA86	4.13E-58	0.355619	0.969	0.571	1.35E-53	cDC	HSPA8
PABPC13	5.95E-58	0.389629	0.99	0.835	1.95E-53	cDC	PABPC1
RPL13A5	5.55E-57	0.404358	0.99	0.764	1.82E-52	cDC	RPL13A
RPS145	2.76E-56	0.300881	0.996	0.872	9.03E-52	cDC	RPS14
RPL224	3.45E-56	0.334398	0.989	0.839	1.13E-51	cDC	RPL22
NDUFB21	4.41E-56	0.261029	0.946	0.539	1.45E-51	cDC	NDUFB2
ANXA52	1.33E-55	0.293005	0.97	0.56	4.35E-51	cDC	ANXA5
COX6A11	5.84E-55	0.316754	0.951	0.608	1.91E-50	cDC	COX6A1
ARPC32	3.09E-54	0.319218	0.989	0.798	1.01E-49	cDC	ARPC3
CHCHD21	6.08E-54	0.314129	0.969	0.618	1.99E-49	cDC	CHCHD2
LIMD26	9.59E-54	0.324866	0.958	0.586	3.14E-49	cDC	LIMD2
ATP5G21	1.18E-52	0.306283	0.987	0.805	3.85E-48	cDC	ATP5G2
MIF5	1.58E-52	0.263846	0.95	0.565	5.16E-48	cDC	MIF
RPL125	6.03E-52	0.322395	0.992	0.867	1.97E-47	cDC	RPL12
COX7C6	1.84E-49	0.370563	0.975	0.702	6.03E-45	cDC	COX7C
RPL35A5	1.53E-48	0.281048	0.994	0.87	5.00E-44	cDC	RPL35A
RPL245	3.50E-44	0.314301	0.987	0.799	1.15E-39	cDC	RPL24
RPL415	7.05E-41	0.261588	0.996	0.883	2.31E-36	cDC	RPL41
RPL215	6.91E-40	0.294864	0.985	0.813	2.26E-35	cDC	RPL21
AHNAK1	8.19E-40	0.318454	0.938	0.599	2.68E-35	cDC	AHNAK
RPS4Y15	1.72E-39	0.310983	0.755	0.461	5.62E-35	cDC	RPS4Y1
RPL285	5.60E-39	0.271141	0.997	0.896	1.83E-34	cDC	RPL28
ATP5L1	1.02E-38	0.276559	0.984	0.77	3.34E-34	cDC	ATP5L
RPLP14	1.52E-38	0.260178	1	0.944	4.97E-34	cDC	RPLP1
FXYD51	2.35E-38	0.259905	0.982	0.757	7.68E-34	cDC	FXYD5
RPS94	1.83E-32	0.283073	0.985	0.829	6.00E-28	cDC	RPS9
MS4A6A1	5.58E-25	0.410723	0.635	0.441	1.83E-20	cDC	MS4A6A
MTRNR2L1	1.47E-06	0.440206	0.213	0.142	0.048174	cDC	MTRNR2L12

HBA2	0	10.06514	0.996	0.075	0	Eryth	HBA2
HBB	0	10.00815	0.996	0.143	0	Eryth	HBB
HBA1	0	7.383945	0.996	0.016	0	Eryth	HBA1
ALAS2	0	5.243745	0.967	0.002	0	Eryth	ALAS2
CA1	0	4.598029	0.896	0.001	0	Eryth	CA1
AHSP	0	3.898876	0.896	0.001	0	Eryth	AHSP
HBM	0	3.616673	0.786	0	0	Eryth	HBM
SLC25A37	0	3.4152	0.991	0.187	0	Eryth	SLC25A37
UBB1	0	3.193393	0.98	0.578	0	Eryth	UBB
HBD	0	3.128692	0.895	0.047	0	Eryth	HBD
SLC25A39	0	3.111079	0.946	0.153	0	Eryth	SLC25A39
GLRX5	0	2.328115	0.88	0.179	0	Eryth	GLRX5
FAM210B	0	2.263373	0.865	0.096	0	Eryth	FAM210B
DCAF12	0	2.21756	0.85	0.116	0	Eryth	DCAF12
GYPC4	0	2.151342	0.909	0.25	0	Eryth	GYPC
SELENBP1	0	2.091324	0.713	0.001	0	Eryth	SELENBP1
SNCA	0	2.040525	0.922	0.103	0	Eryth	SNCA
STRADB	0	1.9759	0.773	0.042	0	Eryth	STRADB
YBX31	0	1.917465	0.933	0.282	0	Eryth	YBX3
EIF1AY1	0	1.846749	0.656	0.121	0	Eryth	EIF1AY
ADIPOR1	0	1.796985	0.932	0.248	0	Eryth	ADIPOR1
TRIM58	0	1.697552	0.797	0.043	0	Eryth	TRIM58
FECH	0	1.586852	0.604	0.027	0	Eryth	FECH
BPGM	0	1.526143	0.638	0.03	0	Eryth	BPGM
MXI1	0	1.479429	0.706	0.081	0	Eryth	MXI1
FAM46C	0	1.43872	0.636	0.017	0	Eryth	FAM46C
NUDT4	0	1.328431	0.678	0.115	0	Eryth	NUDT4
SLC4A1	0	1.278696	0.505	0	0	Eryth	SLC4A1
GYPB	0	1.204786	0.442	0	0	Eryth	GYPB
BCL2L1	0	1.139389	0.654	0.102	0	Eryth	BCL2L1
EPB42	0	1.084298	0.44	0.001	0	Eryth	EPB42
HBQ1	0	1.0522	0.481	0.016	0	Eryth	HBQ1
GMPR	0	0.990934	0.713	0.054	0	Eryth	GMPR
SMIM1	0	0.846313	0.346	0.013	0	Eryth	SMIM1
TMOD1	0	0.844665	0.355	0.002	0	Eryth	TMOD1
MYL4	0	0.798026	0.294	0	0	Eryth	MYL4
HEMGN	0	0.766922	0.351	0.01	0	Eryth	HEMGN
CTA-363E	0	0.726502	0.285	0	0	Eryth	CTA-363E6.6
IFIT1B	0	0.716494	0.227	0	0	Eryth	IFIT1B
PDZK1IP1	0	0.664567	0.344	0.013	0	Eryth	PDZK1IP1
GYPA	0	0.644893	0.244	0	0	Eryth	GYPA
DMTN	0	0.599938	0.512	0.043	0	Eryth	DMTN
PHOSPHO	0	0.521878	0.231	0.002	0	Eryth	PHOSPHO1
LINC0057	0	0.491706	0.201	0	0	Eryth	LINC00570
SLC14A1	0	0.440375	0.196	0.003	0	Eryth	SLC14A1
GPR146	0	0.417771	0.181	0.009	0	Eryth	GPR146
CR1L	0	0.323157	0.128	0.001	0	Eryth	CR1L
RP11-20D	#######	0.282792	0.113	0.003	#######	Eryth	RP11-20D14.6
BNIP3L1	#######	1.887865	0.922	0.345	#######	Eryth	BNIP3L
NFIX	#######	0.528412	0.262	0.021	#######	Eryth	NFIX
FBXO7	#######	1.401803	0.784	0.197	#######	Eryth	FBXO7
ISCA1	#######	0.859856	0.623	0.122	#######	Eryth	ISCA1
RNF10	#######	1.120082	0.726	0.175	#######	Eryth	RNF10
RBM381	#######	1.285343	0.63	0.134	#######	Eryth	RBM38
MPP1	#######	0.935419	0.837	0.238	#######	Eryth	MPP1
CTNNAL1	#######	0.339723	0.181	0.012	#######	Eryth	CTNNAL1
MKRN1	#######	1.2624	0.797	0.247	#######	Eryth	MKRN1
IFI27	#######	0.430921	0.1	0.004	#######	Eryth	IFI27

GSPT1	#######	1.184318	0.689	0.194	#######	Eryth	GSPT1
RIOK3	#######	0.985002	0.837	0.286	#######	Eryth	RIOK3
TSPAN5	#######	0.548397	0.255	0.029	#######	Eryth	TSPAN5
PRDX24	#######	1.381972	0.702	0.217	#######	Eryth	PRDX2
YOD1	#######	0.403967	0.201	0.02	#######	Eryth	YOD1
SLC38A5	#######	0.366112	0.166	0.014	#######	Eryth	SLC38A5
CHPT11	#######	0.760545	0.532	0.137	#######	Eryth	CHPT1
CA2	#######	0.290778	0.312	0.051	#######	Eryth	CA2
OPTN2	#######	0.627276	0.482	0.108	#######	Eryth	OPTN
NCOA4	#######	0.833381	0.891	0.421	#######	Eryth	NCOA4
FKBP8	#######	0.958324	0.902	0.469	#######	Eryth	FKBP8
TCP11L2	#######	0.543093	0.318	0.055	#######	Eryth	TCP11L2
HAGH1	#######	0.769924	0.536	0.145	#######	Eryth	HAGH
SIAH2	#######	0.642651	0.538	0.142	#######	Eryth	SIAH2
BLVRB1	#######	1.288992	0.86	0.381	#######	Eryth	BLVRB
GABARAPI	#######	0.997045	0.88	0.439	#######	Eryth	GABARAPL2
CISD2	#######	0.726043	0.494	0.137	#######	Eryth	CISD2
RPIA1	#######	0.63725	0.434	0.11	#######	Eryth	RPIA
UBE2H	#######	0.650277	0.518	0.148	#######	Eryth	UBE2H
PINK1	#######	0.485985	0.366	0.081	#######	Eryth	PINK1
EPB413	#######	0.697065	0.604	0.205	#######	Eryth	EPB41
GUK11	#######	0.899815	0.884	0.506	5.84E-99	Eryth	GUK1
CDC34	#######	0.548118	0.414	0.112	4.36E-97	Eryth	CDC34
BSG	1.82E-99	0.957018	0.734	0.342	5.97E-95	Eryth	BSG
ASCC2	7.57E-94	0.532896	0.405	0.114	2.48E-89	Eryth	ASCC2
EIF1B	1.21E-88	0.665051	0.68	0.289	3.97E-84	Eryth	EIF1B
DPM2	2.79E-88	0.515372	0.373	0.104	9.14E-84	Eryth	DPM2
PITHD11	6.56E-88	0.609187	0.381	0.108	2.15E-83	Eryth	PITHD1
CLIC21	1.72E-87	0.283713	0.135	0.018	5.62E-83	Eryth	CLIC2
8-Mar	2.16E-79	0.370791	0.229	0.048	7.06E-75	Eryth	8-Mar
R3HDM4	2.37E-76	0.308102	0.645	0.27	7.76E-72	Eryth	R3HDM4
RGCC3	2.75E-76	0.39368	0.338	0.091	9.01E-72	Eryth	RGCC
CCNDBP1:	8.73E-73	0.726533	0.575	0.248	2.86E-68	Eryth	CCNDBP1
RAD23A	1.14E-72	0.737023	0.66	0.318	3.72E-68	Eryth	RAD23A
MAP2K3	1.27E-72	0.463413	0.593	0.243	4.16E-68	Eryth	MAP2K3
C9orf783	7.59E-69	0.624573	0.64	0.286	2.48E-64	Eryth	C9orf78
YPEL3	3.53E-64	0.55356	0.86	0.516	1.16E-59	Eryth	YPEL3
FAM104A	2.93E-63	0.365127	0.322	0.098	9.60E-59	Eryth	FAM104A
MOSPD1	3.70E-62	0.306325	0.144	0.026	1.21E-57	Eryth	MOSPD1
GLUL1	9.04E-62	0.333282	0.651	0.29	2.96E-57	Eryth	GLUL
MAP1LC3E	1.76E-58	0.398051	0.713	0.364	5.76E-54	Eryth	MAP1LC3B
JAZF1	2.46E-58	0.38506	0.329	0.106	8.04E-54	Eryth	JAZF1
LGALS32	2.76E-54	0.641788	0.865	0.476	9.04E-50	Eryth	LGALS3
BAG1	4.35E-52	0.475552	0.518	0.23	1.42E-47	Eryth	BAG1
EMC3	9.00E-51	0.274628	0.47	0.197	2.95E-46	Eryth	EMC3
SELK1	1.19E-49	0.493429	0.521	0.236	3.90E-45	Eryth	SELK
PSMF1	8.52E-47	0.441453	0.516	0.236	2.79E-42	Eryth	PSMF1
SESN32	2.24E-42	0.344321	0.287	0.101	7.34E-38	Eryth	SESN3
HBG2	8.92E-42	5.119096	0.107	0.021	2.92E-37	Eryth	HBG2
TERF2IP1	3.37E-37	0.45481	0.584	0.323	1.10E-32	Eryth	TERF2IP
DCUN1D1	6.14E-33	0.275031	0.233	0.085	2.01E-28	Eryth	DCUN1D1
ACP11	8.69E-31	0.37529	0.46	0.239	2.85E-26	Eryth	ACP1
CNPPD13	1.68E-30	0.252854	0.394	0.187	5.48E-26	Eryth	CNPPD1
HIST1H1C	2.74E-25	0.260114	0.275	0.123	8.97E-21	Eryth	HIST1H1C
SEC621	1.69E-18	0.274341	0.749	0.516	5.52E-14	Eryth	SEC62
CAT3	3.84E-17	0.328773	0.458	0.285	1.26E-12	Eryth	CAT
SOD16	1.57E-07	0.260701	0.508	0.393	0.005147	Eryth	SOD1
GNLY1	0	5.630257	0.985	0.047	0	NK	GNLY

NKG71	0	4.270077	0.999	0.164	0	NK	NKG7
PRF11	0	4.084946	0.978	0.069	0	NK	PRF1
GZMB1	0	3.665826	0.916	0.039	0	NK	GZMB
GZMA1	0	3.498957	0.959	0.074	0	NK	GZMA
SPON2	0	3.418345	0.8	0.024	0	NK	SPON2
FGFBP21	0	3.397632	0.799	0.027	0	NK	FGFBP2
CTSW2	0	3.275495	0.978	0.138	0	NK	CTSW
CST71	0	3.186955	0.974	0.12	0	NK	CST7
KLRD11	0	3.089074	0.916	0.039	0	NK	KLRD1
CCL41	0	3.042338	0.78	0.047	0	NK	CCL4
CD76	0	3.030854	0.946	0.15	0	NK	CD7
HOPX1	0	2.99883	0.865	0.055	0	NK	HOPX
CLIC31	0	2.977069	0.783	0.035	0	NK	CLIC3
CD2474	0	2.919842	0.919	0.121	0	NK	CD247
IFITM14	0	2.86965	0.994	0.292	0	NK	IFITM1
KLRB12	0	2.645878	0.833	0.055	0	NK	KLRB1
PTGDS	0	2.555175	0.181	0.009	0	NK	PTGDS
KLRF11	0	2.548295	0.794	0.015	0	NK	KLRF1
GZMH1	0	2.504698	0.722	0.039	0	NK	GZMH
GZMM2	0	2.354607	0.83	0.092	0	NK	GZMM
PTPRCAP5	0	2.284346	0.97	0.229	0	NK	PTPRCAP
IL2RB1	0	2.280133	0.686	0.024	0	NK	IL2RB
MATK1	0	2.200305	0.745	0.045	0	NK	MATK
TBX211	0	2.093612	0.681	0.027	0	NK	TBX21
FCGR3A1	0	2.051482	0.84	0.124	0	NK	FCGR3A
ARL4C3	0	2.000799	0.789	0.149	0	NK	ARL4C
PTGDR1	0	1.955147	0.634	0.023	0	NK	PTGDR
IL2RG5	0	1.953544	0.926	0.292	0	NK	IL2RG
SH2D1B	0	1.946959	0.606	0.013	0	NK	SH2D1B
XBP11	0	1.944916	0.818	0.227	0	NK	XBP1
GPR561	0	1.939276	0.588	0.016	0	NK	GPR56
CCL51	0	1.895131	0.803	0.199	0	NK	CCL5
XCL21	0	1.892868	0.44	0.015	0	NK	XCL2
CX3CR12	0	1.885527	0.754	0.218	0	NK	CX3CR1
TTC381	0	1.839228	0.6	0.038	0	NK	TTC38
PYHIN11	0	1.797792	0.592	0.044	0	NK	PYHIN1
ABHD17A1	0	1.790592	0.769	0.202	0	NK	ABHD17A
RARRES34	0	1.787182	0.88	0.329	0	NK	RARRES3
S1PR51	0	1.774278	0.567	0.02	0	NK	S1PR5
ZAP704	0	1.759875	0.661	0.086	0	NK	ZAP70
DDIT44	0	1.753602	0.576	0.071	0	NK	DDIT4
SYNE22	0	1.737415	0.609	0.091	0	NK	SYNE2
PRSS231	0	1.737358	0.493	0.011	0	NK	PRSS23
IGFBP71	0	1.700276	0.562	0.11	0	NK	IGFBP7
APMAP1	0	1.687473	0.703	0.121	0	NK	APMAP
CMC11	0	1.655495	0.553	0.128	0	NK	CMC1
APOBEC3G	0	1.639588	0.637	0.123	0	NK	APOBEC3G
SAMD31	0	1.626884	0.582	0.05	0	NK	SAMD3
KLRC11	0	1.625722	0.343	0.009	0	NK	KLRC1
EVL5	0	1.622989	0.805	0.255	0	NK	EVL
NCR32	0	1.588702	0.516	0.036	0	NK	NCR3
DENND2D	0	1.574227	0.682	0.156	0	NK	DENND2D
JAK11	0	1.563923	0.922	0.518	0	NK	JAK1
AKR1C3	0	1.55241	0.455	0.009	0	NK	AKR1C3
STK17A5	0	1.532024	0.727	0.192	0	NK	STK17A
RAC25	0	1.530214	0.948	0.556	0	NK	RAC2
SKAP14	0	1.530118	0.643	0.109	0	NK	SKAP1
GNPTAB1	0	1.527303	0.597	0.137	0	NK	GNPTAB

PTPN41	0	1.524793	0.567	0.079	0	NK	PTPN4
TXK3	0	1.522912	0.516	0.041	0	NK	TXK
PRKCH4	0	1.513175	0.559	0.083	0	NK	PRKCH
SLC9A3R1	0	1.509516	0.803	0.285	0	NK	SLC9A3R1
PLEKHF11	0	1.509025	0.515	0.034	0	NK	PLEKHF1
SH2D2A1	0	1.503311	0.482	0.039	0	NK	SH2D2A
CHST121	0	1.502083	0.572	0.074	0	NK	CHST12
ID23	0	1.4959	0.664	0.214	0	NK	ID2
KLF26	0	1.485659	0.962	0.501	0	NK	KLF2
CXXC51	0	1.467349	0.556	0.095	0	NK	CXXC5
LINC00861	0	1.454607	0.701	0.152	0	NK	LINC00861
FCRL61	0	1.436786	0.444	0.019	0	NK	FCRL6
NCR1	0	1.427461	0.432	0.007	0	NK	NCR1
MYBL11	0	1.427376	0.444	0.032	0	NK	MYBL1
HCST1	0	1.420878	0.953	0.592	0	NK	HCST
LITAF1	0	1.412215	0.846	0.361	0	NK	LITAF
NFATC22	0	1.389331	0.471	0.055	0	NK	NFATC2
ETS15	0	1.379532	0.652	0.159	0	NK	ETS1
GNG22	0	1.377768	0.658	0.217	0	NK	GNG2
ARPC5L1	0	1.3747	0.643	0.188	0	NK	ARPC5L
AES5	0	1.370314	0.851	0.391	0	NK	AES
MBP1	0	1.361966	0.805	0.363	0	NK	MBP
CD812	0	1.348026	0.66	0.216	0	NK	CD81
IL324	0	1.347166	0.504	0.198	0	NK	IL32
SPN2	0	1.342611	0.676	0.252	0	NK	SPN
LCK4	0	1.342447	0.67	0.159	0	NK	LCK
CEP781	0	1.331113	0.426	0.039	0	NK	CEP78
CD1601	0	1.324164	0.36	0.011	0	NK	CD160
GBP52	0	1.309838	0.602	0.133	0	NK	GBP5
SUN24	0	1.302684	0.692	0.256	0	NK	SUN2
PDIA31	0	1.295936	0.81	0.394	0	NK	PDIA3
LY6E3	0	1.278639	0.764	0.347	0	NK	LY6E
C1orf211	0	1.256095	0.419	0.021	0	NK	C1orf21
CD300A1	0	1.239338	0.562	0.168	0	NK	CD300A
SYTL31	0	1.234934	0.445	0.063	0	NK	SYTL3
TXNIP6	0	1.226234	0.976	0.732	0	NK	TXNIP
SLFN54	0	1.221923	0.536	0.162	0	NK	SLFN5
CALR1	0	1.217239	0.691	0.348	0	NK	CALR
STAT44	0	1.211521	0.439	0.05	0	NK	STAT4
EFHD21	0	1.204104	0.928	0.548	0	NK	EFHD2
XCL11	0	1.203294	0.212	0.008	0	NK	XCL1
TGFBR31	0	1.2003	0.37	0.022	0	NK	TGFBR3
ABI33	0	1.194421	0.564	0.172	0	NK	ABI3
RUNX32	0	1.189549	0.449	0.085	0	NK	RUNX3
LBH5	0	1.188976	0.559	0.14	0	NK	LBH
CTSC2	0	1.184431	0.788	0.371	0	NK	CTSC
C5orf561	0	1.173171	0.488	0.116	0	NK	C5orf56
HLA-A1	0	1.172362	0.99	0.857	0	NK	HLA-A
MYOM2	0	1.161823	0.201	0.006	0	NK	MYOM2
ZNF6001	0	1.159901	0.391	0.047	0	NK	ZNF600
CLEC2B2	0	1.155982	0.615	0.232	0	NK	CLEC2B
CD38	0	1.153205	0.345	0.033	0	NK	CD38
KLRG11	0	1.150907	0.34	0.063	0	NK	KLRG1
ITGB73	0	1.144416	0.422	0.104	0	NK	ITGB7
HSPA87	0	1.133743	0.936	0.553	0	NK	HSPA8
HLA-B1	0	1.126603	0.996	0.919	0	NK	HLA-B
RORA2	0	1.125642	0.408	0.066	0	NK	RORA
SYTL13	0	1.12297	0.508	0.144	0	NK	SYTL1

RASSF11	0	1.12234	0.498	0.138	0	NK	RASSF1
CD25	0	1.117687	0.464	0.161	0	NK	CD2
MT-ND4L(	0	1.115243	0.989	0.761	0	NK	MT-ND4L
B2M2	0	1.112808	1	0.978	0	NK	B2M
TFDP21	0	1.10836	0.384	0.046	0	NK	TFDP2
TBC1D10C	0	1.107068	0.572	0.189	0	NK	TBC1D10C
PRMT24	0	1.105842	0.759	0.365	0	NK	PRMT2
ADRB21	0	1.099477	0.387	0.068	0	NK	ADRB2
HMOX24	0	1.09036	0.516	0.153	0	NK	HMOX2
SYNE11	0	1.089203	0.536	0.132	0	NK	SYNE1
KLRC31	0	1.083931	0.261	0.011	0	NK	KLRC3
RHOC1	0	1.080924	0.603	0.164	0	NK	RHOC
CALM12	0	1.06624	0.974	0.742	0	NK	CALM1
DHRS71	0	1.061729	0.737	0.35	0	NK	DHRS7
MT-ATP87	0	1.054811	0.938	0.724	0	NK	MT-ATP8
ANXA63	0	1.052876	0.735	0.339	0	NK	ANXA6
DIP2A1	0	1.050264	0.461	0.134	0	NK	DIP2A
LAIR21	0	1.04398	0.23	0.012	0	NK	LAIR2
NMUR1	0	1.032034	0.299	0.004	0	NK	NMUR1
BIN15	0	1.028642	0.484	0.132	0	NK	BIN1
OPTN3	0	1.018164	0.42	0.09	0	NK	OPTN
Sep-71	0	1.017867	0.836	0.466	0	NK	7-Sep
EOMES1	0	1.01469	0.316	0.025	0	NK	EOMES
TSEN541	0	1.003582	0.366	0.059	0	NK	TSEN54
C9orf1421	0	0.999769	0.619	0.272	0	NK	C9orf142
ARHGEF33	0	0.994902	0.435	0.13	0	NK	ARHGEF3
IFITM21	0	0.990542	0.976	0.773	0	NK	IFITM2
HSH2D1	0	0.987055	0.388	0.075	0	NK	HSH2D
15-Sep	0	0.98665	0.456	0.123	0	NK	1-Sep
GIMAP74	0	0.976584	0.915	0.551	0	NK	GIMAP7
CD532	0	0.976086	0.787	0.441	0	NK	CD53
GPR652	0	0.970768	0.538	0.219	0	NK	GPR65
S1PR15	0	0.968895	0.402	0.092	0	NK	S1PR1
TMIGD22	0	0.967087	0.29	0.026	0	NK	TMIGD2
RNF1251	0	0.966145	0.426	0.119	0	NK	RNF125
HSPA51	0	0.965784	0.591	0.299	0	NK	HSPA5
FASLG1	0	0.964296	0.292	0.009	0	NK	FASLG
FKBP112	0	0.959235	0.36	0.072	0	NK	FKBP11
PTPRC2	0	0.955543	0.971	0.74	0	NK	PTPRC
FAIM35	0	0.952796	0.455	0.115	0	NK	FAIM3
CDK2AP22	0	0.94077	0.555	0.262	0	NK	CDK2AP2
CHST21	0	0.936214	0.309	0.043	0	NK	CHST2
TPST2	0	0.931361	0.618	0.175	0	NK	TPST2
PPP3CC4	0	0.927482	0.411	0.105	0	NK	PPP3CC
UBB2	0	0.924722	0.894	0.561	0	NK	UBB
PPIB2	0	0.923985	0.881	0.57	0	NK	PPIB
PFN13	0	0.920638	0.991	0.907	0	NK	PFN1
PTGER22	0	0.919385	0.371	0.095	0	NK	PTGER2
KIR3DL1	0	0.918583	0.181	0.002	0	NK	KIR3DL1
MYL12A2	0	0.915052	0.975	0.746	0	NK	MYL12A
CD472	0	0.914123	0.63	0.289	0	NK	CD47
UCP23	0	0.914108	0.83	0.532	0	NK	UCP2
RASAL31	0	0.912897	0.411	0.123	0	NK	RASAL3
HLA-C1	0	0.912144	0.993	0.88	0	NK	HLA-C
CD244	0	0.910226	0.346	0.068	0	NK	CD244
MCTP2	0	0.907399	0.346	0.045	0	NK	MCTP2
CCL3	0	0.899958	0.194	0.013	0	NK	CCL3
TIGIT1	0	0.897404	0.265	0.026	0	NK	TIGIT

MALAT15	0	0.89725	0.999	0.941	0	NK	MALAT1
OSTF1	0	0.893501	0.687	0.358	0	NK	OSTF1
SLAMF71	0	0.889977	0.323	0.058	0	NK	SLAMF7
CD964	0	0.88495	0.374	0.079	0	NK	CD96
RAB7L11	0	0.882259	0.402	0.123	0	NK	RAB7L1
PLEK	0	0.880266	0.864	0.481	0	NK	PLEK
SLC38A16	0	0.879862	0.37	0.105	0	NK	SLC38A1
CBLB1	0	0.878727	0.321	0.064	0	NK	CBLB
PARP152	0	0.875461	0.282	0.042	0	NK	PARP15
CYFIP26	0	0.872752	0.442	0.153	0	NK	CYFIP2
HIST1H4C!	0	0.867365	0.785	0.464	0	NK	HIST1H4C
PRKACB3	0	0.8652	0.414	0.131	0	NK	PRKACB
NCL6	0	0.865075	0.786	0.48	0	NK	NCL
RHOF3	0	0.864416	0.442	0.145	0	NK	RHOF
FUT111	0	0.863659	0.319	0.059	0	NK	FUT11
IL18RAP	0	0.855704	0.256	0.008	0	NK	IL18RAP
SASH31	0	0.853817	0.533	0.247	0	NK	SASH3
GIMAP54	0	0.850917	0.612	0.254	0	NK	GIMAP5
HDDC23	0	0.847888	0.375	0.122	0	NK	HDDC2
TAP11	0	0.847265	0.516	0.229	0	NK	TAP1
FAM49B1	0	0.84327	0.694	0.368	0	NK	FAM49B
TRAF3IP35	0	0.841041	0.715	0.412	0	NK	TRAF3IP3
PPP2R2B1	0	0.839718	0.256	0.018	0	NK	PPP2R2B
SH2D1A3	0	0.835244	0.308	0.052	0	NK	SH2D1A
ITGB21	0	0.831885	0.939	0.659	0	NK	ITGB2
CD3202	0	0.828815	0.269	0.045	0	NK	CD320
PLEKHA11	0	0.823573	0.296	0.06	0	NK	PLEKHA1
ATP2B41	0	0.823159	0.383	0.13	0	NK	ATP2B4
NCAM1	0	0.822385	0.228	0.004	0	NK	NCAM1
ACAA21	0	0.810313	0.39	0.139	0	NK	ACAA2
RFTN11	0	0.810127	0.339	0.094	0	NK	RFTN1
USP281	0	0.809441	0.273	0.046	0	NK	USP28
BTN3A21	0	0.808107	0.459	0.181	0	NK	BTN3A2
CCND23	0	0.806162	0.298	0.071	0	NK	CCND2
PLAC81	0	0.805123	0.754	0.37	0	NK	PLAC8
CCDC1072	0	0.797041	0.424	0.16	0	NK	CCDC107
NCALD1	0	0.794191	0.251	0.016	0	NK	NCALD
LINC00299	0	0.789758	0.205	0.003	0	NK	LINC00299
GYPC5	0	0.788758	0.567	0.236	0	NK	GYPC
PPP1R181	0	0.787565	0.691	0.382	0	NK	PPP1R18
LPCAT1	0	0.782087	0.343	0.103	0	NK	LPCAT1
CD693	0	0.77904	0.275	0.047	0	NK	CD69
PITPNC11	0	0.779019	0.328	0.099	0	NK	PITPNC1
PILRB1	0	0.761108	0.289	0.062	0	NK	PILRB
PPP1CA2	0	0.760212	0.812	0.525	0	NK	PPP1CA
ARAP22	0	0.758215	0.341	0.108	0	NK	ARAP2
PIM13	0	0.755865	0.434	0.156	0	NK	PIM1
ARID5A1	0	0.754043	0.299	0.077	0	NK	ARID5A
SH3BP53	0	0.748055	0.436	0.167	0	NK	SH3BP5
PRDM12	0	0.738733	0.234	0.034	0	NK	PRDM1
BIN2	0	0.735871	0.762	0.423	0	NK	BIN2
RASA32	0	0.734901	0.386	0.132	0	NK	RASA3
GFOD1	0	0.733368	0.231	0.026	0	NK	GFOD1
FCRL32	0	0.733324	0.211	0.013	0	NK	FCRL3
SYTL21	0	0.730469	0.228	0.025	0	NK	SYTL2
PSME12	0	0.728535	0.927	0.675	0	NK	PSME1
PSMB92	0	0.724393	0.834	0.53	0	NK	PSMB9
LLGL21	0	0.721735	0.232	0.022	0	NK	LLGL2

AC006129	0	0.719308	0.304	0.083	0	NK	AC006129.2
MIB21	0	0.712856	0.263	0.058	0	NK	MIB2
HLA-E1	0	0.710178	0.981	0.795	0	NK	HLA-E
CD991	0	0.708589	0.902	0.568	0	NK	CD99
TOX1	0	0.704331	0.211	0.014	0	NK	TOX
KLF122	0	0.702403	0.249	0.056	0	NK	KLF12
SLFN12L2	0	0.698522	0.225	0.032	0	NK	SLFN12L
PTPN221	0	0.695075	0.292	0.078	0	NK	PTPN22
RGS3	0	0.69266	0.269	0.059	0	NK	RGS3
PRR5L1	0	0.684978	0.219	0.028	0	NK	PRR5L
KIR2DL3	0	0.681163	0.133	0.001	0	NK	KIR2DL3
AGK	0	0.680629	0.226	0.039	0	NK	AGK
GLCCI11	0	0.678076	0.22	0.034	0	NK	GLCCI1
IKZF32	0	0.675464	0.222	0.033	0	NK	IKZF3
CARD112	0	0.672967	0.225	0.034	0	NK	CARD11
GK5	0	0.662322	0.193	0.021	0	NK	GK5
MMP23B	0	0.659434	0.189	0.006	0	NK	MMP23B
ZBTB16	0	0.65741	0.213	0.02	0	NK	ZBTB16
NPRL21	0	0.656609	0.249	0.059	0	NK	NPRL2
TNIK4	0	0.650288	0.267	0.057	0	NK	TNIK
SLAMF62	0	0.647317	0.218	0.033	0	NK	SLAMF6
RP11-81H	0	0.646313	0.196	0.015	0	NK	RP11-81H14.2
PTPN72	0	0.645118	0.255	0.056	0	NK	PTPN7
GATA32	0	0.64398	0.233	0.044	0	NK	GATA3
RAP1B	0	0.632252	0.865	0.475	0	NK	RAP1B
KIR2DL2	0	0.62783	0.113	0.003	0	NK	KIR2DL2
PDGFD	0	0.623064	0.173	0.005	0	NK	PDGFD
C12orf751	0	0.618	0.485	0.125	0	NK	C12orf75
RPL319	0	0.615154	0.962	0.732	0	NK	RPL3
OSBPL5	0	0.614636	0.211	0.031	0	NK	OSBPL5
RPS275	0	0.612689	0.978	0.822	0	NK	RPS27
CCND3	0	0.612222	0.798	0.422	0	NK	CCND3
YPEL1	0	0.610663	0.182	0.012	0	NK	YPEL1
RP11-94L1	0	0.609933	0.2	0.037	0	NK	RP11-94L15.2
CXCR2	0	0.606245	0.226	0.025	0	NK	CXCR2
FEZ1	0	0.600605	0.16	0.004	0	NK	FEZ1
TNFRSF18	0	0.599329	0.145	0.009	0	NK	TNFRSF18
RRAS22	0	0.589814	0.187	0.018	0	NK	RRAS2
RP11-25K1	0	0.587967	0.175	0.014	0	NK	RP11-25K19.1
CAPN12	0	0.58222	0.165	0.012	0	NK	CAPN12
PRR52	0	0.578996	0.191	0.03	0	NK	PRR5
ZBP1	0	0.576482	0.199	0.03	0	NK	ZBP1
MLC1	0	0.565956	0.178	0.01	0	NK	MLC1
F2R	0	0.560639	0.231	0.031	0	NK	F2R
CCDC28B	0	0.558915	0.193	0.029	0	NK	CCDC28B
DHRS3	0	0.558071	0.179	0.021	0	NK	DHRS3
RP11-473I	0	0.550081	0.183	0.026	0	NK	RP11-473M20.7
RHOBTB3	0	0.545299	0.154	0.01	0	NK	RHOBTB3
PAM	0	0.543529	0.187	0.029	0	NK	PAM
KIR2DL1	0	0.535476	0.101	0.001	0	NK	KIR2DL1
YES1	0	0.534945	0.166	0.009	0	NK	YES1
ADAMTS1	0	0.510192	0.13	0.001	0	NK	ADAMTS1
MIR181A2	0	0.506297	0.131	0.002	0	NK	MIR181A2HG
DTHD1	0	0.495154	0.129	0.005	0	NK	DTHD1
GPR114	0	0.492208	0.15	0.011	0	NK	GPR114
ENPP4	0	0.489975	0.15	0.015	0	NK	ENPP4
SLCO4C1	0	0.488626	0.152	0.015	0	NK	SLCO4C1
RP6-206I1	0	0.481362	0.173	0.029	0	NK	RP6-206I17.1

PLEKHG3	0	0.472074	0.167	0.026	0	NK	PLEKHG3
KLRK11	0	0.456744	0.134	0.009	0	NK	KLRK1
RAMP1	0	0.451775	0.123	0.003	0	NK	RAMP1
APBA22	0	0.441575	0.138	0.018	0	NK	APBA2
COLQ	0	0.440024	0.113	0.005	0	NK	COLQ
ABCB1	0	0.439144	0.129	0.01	0	NK	ABCB1
IKZF2	0	0.430959	0.117	0.011	0	NK	IKZF2
CCDC102A	0	0.428532	0.132	0.012	0	NK	CCDC102A
RP11-222I	0	0.422053	0.125	0.01	0	NK	RP11-222K16.2
LGR6	0	0.41658	0.106	0.003	0	NK	LGR6
LAT5	0	0.409582	0.452	0.16	0	NK	LAT
ZNF595	0	0.402343	0.116	0.012	0	NK	ZNF595
BZRAP1	0	0.401784	0.109	0.005	0	NK	BZRAP1
NEIL1	0	0.401448	0.108	0.01	0	NK	NEIL1
HOXB4	0	0.398317	0.118	0.014	0	NK	HOXB4
GPR68	0	0.390052	0.11	0.006	0	NK	GPR68
JAKMIP2	0	0.389863	0.111	0.009	0	NK	JAKMIP2
SBK1	0	0.36278	0.102	0.009	0	NK	SBK1
CXCR1	0	0.31331	0.102	0.006	0	NK	CXCR1
ATM3	#######	0.907565	0.542	0.257	#######	NK	ATM
TES2	#######	0.809206	0.548	0.262	#######	NK	TES
STARD3NL	#######	0.764348	0.373	0.131	#######	NK	STARD3NL
FYN1	#######	0.824977	0.536	0.25	#######	NK	FYN
S100B1	#######	1.199332	0.11	0.012	#######	NK	S100B
CLIC1	#######	0.630213	0.955	0.724	#######	NK	CLIC1
IGF2R1	#######	0.874572	0.474	0.198	#######	NK	IGF2R
CDK171	#######	0.607926	0.237	0.058	#######	NK	CDK17
RPA24	#######	0.726499	0.361	0.125	#######	NK	RPA2
MVD	#######	0.548278	0.194	0.039	#######	NK	MVD
HMGN32	#######	0.782232	0.625	0.337	#######	NK	HMGN3
PPIA6	#######	0.666323	0.948	0.72	#######	NK	PPIA
ADAM81	#######	0.666401	0.367	0.126	#######	NK	ADAM8
AOAH1	#######	0.805893	0.604	0.306	#######	NK	AOAH
DOK21	#######	0.731224	0.648	0.344	#######	NK	DOK2
RNF2131	#######	0.76539	0.804	0.517	#######	NK	RNF213
KIF21A1	#######	0.427933	0.127	0.017	#######	NK	KIF21A
CIRBP6	#######	0.766259	0.716	0.43	#######	NK	CIRBP
HENMT11	#######	0.547371	0.199	0.043	#######	NK	HENMT1
PRKCQ3	#######	0.496857	0.18	0.035	#######	NK	PRKCQ
RALY	#######	0.698875	0.58	0.302	#######	NK	RALY
MT-ND53	#######	0.675745	0.974	0.781	#######	NK	MT-ND5
MAPRE2	#######	0.570237	0.362	0.127	#######	NK	MAPRE2
TMC84	#######	0.751508	0.38	0.141	#######	NK	TMC8
CLSTN3	#######	0.407424	0.121	0.016	#######	NK	CLSTN3
SSR25	#######	0.725883	0.758	0.467	#######	NK	SSR2
STK43	#######	0.744282	0.71	0.429	#######	NK	STK4
ISG205	#######	0.730628	0.423	0.164	#######	NK	ISG20
HSP90B12	#######	0.7626	0.75	0.455	#######	NK	HSP90B1
CCDC88C1	#######	0.727977	0.386	0.149	#######	NK	CCDC88C
RCN24	#######	0.684019	0.323	0.11	#######	NK	RCN2
ALOX5AP1	#######	0.533714	0.615	0.297	#######	NK	ALOX5AP
ACAP15	#######	0.73006	0.513	0.238	#######	NK	ACAP1
SIGIRR3	#######	0.768293	0.517	0.252	#######	NK	SIGIRR
CORO1A3	#######	0.685373	0.933	0.719	#######	NK	CORO1A
TBC1D31	#######	0.411327	0.127	0.018	#######	NK	TBC1D31
IL165	#######	0.781285	0.505	0.242	#######	NK	IL16
RPS36	#######	0.48124	0.978	0.823	#######	NK	RPS3
DBI1	#######	0.716389	0.756	0.481	#######	NK	DBI

KLHDC4	#######	0.512901	0.184	0.039	#######	NK	KLHDC4
PSMB102	#######	0.741936	0.747	0.474	#######	NK	PSMB10
ARHGEF9	#######	0.54594	0.195	0.044	#######	NK	ARHGEF9
AC092580	#######	0.521946	0.182	0.038	#######	NK	AC092580.4
SIDT1	#######	0.436448	0.142	0.024	#######	NK	SIDT1
YWHAZ	#######	0.319054	0.893	0.648	#######	NK	YWHAZ
RASA23	#######	0.698466	0.339	0.121	#######	NK	RASA2
UCHL51	#######	0.655982	0.304	0.101	#######	NK	UCHL5
RALGDS1	#######	0.551331	0.203	0.048	#######	NK	RALGDS
MT-CO13	#######	0.392314	0.999	0.985	#######	NK	MT-CO1
MLLT61	#######	0.645504	0.26	0.076	#######	NK	MLLT6
HNRNPF3	#######	0.7491	0.639	0.372	#######	NK	HNRNPF
RMDN3	#######	0.522095	0.195	0.045	#######	NK	RMDN3
MYL12B2	#######	0.614853	0.873	0.624	#######	NK	MYL12B
SNTB2	#######	0.559461	0.233	0.062	#######	NK	SNTB2
LYAR1	#######	0.764582	0.347	0.129	#######	NK	LYAR
PLA2G161	#######	0.670908	0.312	0.105	#######	NK	PLA2G16
DEF63	#######	0.721789	0.474	0.222	#######	NK	DEF6
UTRN1	#######	0.800052	0.523	0.261	#######	NK	UTRN
POLR3GL2	#######	0.708219	0.428	0.188	#######	NK	POLR3GL
TRAPPC10	#######	0.669502	0.357	0.135	#######	NK	TRAPPC10
MRPS61	#######	0.769475	0.467	0.22	#######	NK	MRPS6
DUSP21	#######	0.695982	0.18	0.039	#######	NK	DUSP2
HNRNPA1	#######	0.646945	0.894	0.612	#######	NK	HNRNPA1
Sep-65	#######	0.630995	0.568	0.295	#######	NK	6-Sep
NFATC31	#######	0.70048	0.355	0.137	#######	NK	NFATC3
PCBP12	#######	0.620103	0.876	0.638	#######	NK	PCBP1
MT-ND64	#######	0.894518	0.672	0.399	#######	NK	MT-ND6
CYTIP5	#######	0.737506	0.507	0.252	#######	NK	CYTIP
RBL24	#######	0.779398	0.494	0.242	#######	NK	RBL2
SDF4	#######	0.731893	0.427	0.193	#######	NK	SDF4
SRPK2	#######	0.717587	0.437	0.197	#######	NK	SRPK2
USP113	#######	0.56733	0.221	0.059	#######	NK	USP11
MAT2B5	#######	0.715046	0.522	0.271	#######	NK	MAT2B
RNF1672	#######	0.705656	0.44	0.201	#######	NK	RNF167
TSTD15	#######	0.649821	0.391	0.158	#######	NK	TSTD1
NDUFB22	#######	0.645301	0.79	0.529	#######	NK	NDUFB2
IRF13	#######	0.685045	0.746	0.462	#######	NK	IRF1
TMEM109	#######	0.685095	0.337	0.129	#######	NK	TMEM109
ARHGEF12	#######	0.731712	0.553	0.299	#######	NK	ARHGEF1
CYBA2	#######	0.568802	0.983	0.803	#######	NK	CYBA
PARP81	#######	0.653802	0.367	0.146	#######	NK	PARP8
ITGAL2	#######	0.759373	0.48	0.235	#######	NK	ITGAL
NAALADL1	#######	0.429076	0.145	0.028	#######	NK	NAALADL1
BZW11	#######	0.704208	0.55	0.303	#######	NK	BZW1
LIMD27	#######	0.687086	0.83	0.575	#######	NK	LIMD2
EIF3G2	#######	0.758831	0.744	0.504	#######	NK	EIF3G
SELPLG1	#######	0.641115	0.665	0.401	#######	NK	SELPLG
ARL6IP11	#######	0.635224	0.528	0.276	#######	NK	ARL6IP1
CD972	#######	0.634859	0.661	0.384	#######	NK	CD97
BUB35	#######	0.682344	0.43	0.198	#######	NK	BUB3
ZNF2761	#######	0.581859	0.259	0.082	#######	NK	ZNF276
HOXB21	#######	0.470398	0.153	0.032	#######	NK	HOXB2
MIR1422	#######	0.685078	0.476	0.232	#######	NK	MIR142
FUS5	#######	0.738526	0.66	0.413	#######	NK	FUS
DDX52	#######	0.704501	0.833	0.591	#######	NK	DDX5
KIFAP3	#######	0.473146	0.244	0.074	#######	NK	KIFAP3
CISD31	#######	0.720014	0.414	0.187	#######	NK	CISD3

MANF1	#######	0.658078	0.291	0.103	#######	NK	MANF
RNF1261	#######	0.660704	0.339	0.135	#######	NK	RNF126
CD164	#######	0.660729	0.604	0.354	#######	NK	CD164
TUBB6	#######	0.685525	0.615	0.367	#######	NK	TUBB
ATP8A11	#######	0.483863	0.188	0.047	#######	NK	ATP8A1
ITK4	#######	0.563736	0.231	0.068	#######	NK	ITK
LNPEP1	#######	0.712354	0.459	0.226	#######	NK	LNPEP
C19orf121	#######	0.518791	0.195	0.051	#######	NK	C19orf12
ADH53	#######	0.656878	0.383	0.17	#######	NK	ADH5
ADD11	#######	0.669558	0.447	0.219	#######	NK	ADD1
TPD523	#######	0.487136	0.172	0.041	#######	NK	TPD52
HLA-F1	#######	0.673402	0.61	0.365	#######	NK	HLA-F
BTN3A11	#######	0.600563	0.274	0.095	#######	NK	BTN3A1
PTPRA	#######	0.636951	0.504	0.263	#######	NK	PTPRA
RAB37	#######	0.342504	0.292	0.099	#######	NK	RAB37
ARHGAP2!	#######	0.633737	0.375	0.162	#######	NK	ARHGAP25
MST4	#######	0.529099	0.227	0.069	#######	NK	MST4
TGFBR1	#######	0.525512	0.206	0.058	#######	NK	TGFBR1
PSMB82	#######	0.646013	0.694	0.449	#######	NK	PSMB8
CCDC85B	#######	0.538873	0.592	0.344	#######	NK	CCDC85B
CYB5B1	#######	0.597313	0.28	0.101	#######	NK	CYB5B
ZNF223	#######	0.654175	0.34	0.141	#######	NK	ZNF22
TMED91	#######	0.689638	0.45	0.23	#######	NK	TMED9
RPL23A6	#######	0.52462	0.943	0.727	#######	NK	RPL23A
PDIA62	#######	0.724652	0.491	0.261	#######	NK	PDIA6
CDC42SE1	#######	0.663169	0.564	0.327	#######	NK	CDC42SE1
GIMAP44	#######	0.626877	0.766	0.496	#######	NK	GIMAP4
IL18R1	#######	0.341667	0.105	0.017	#######	NK	IL18R1
IMP35	#######	0.649637	0.491	0.262	#######	NK	IMP3
TAGAP5	#######	0.703394	0.47	0.233	#######	NK	TAGAP
RAP1GAP2	#######	0.569329	0.24	0.078	#######	NK	RAP1GAP2
FCGR3B1	#######	0.362092	0.138	0.028	#######	NK	FCGR3B
TSPAN2	#######	0.311536	0.158	0.036	#######	NK	TSPAN2
ICAM22	#######	0.503373	0.47	0.237	#######	NK	ICAM2
LCP22	#######	0.670626	0.534	0.3	#######	NK	LCP2
SCML44	#######	0.498411	0.223	0.067	#######	NK	SCML4
ZNF4283	#######	0.611332	0.37	0.164	#######	NK	ZNF428
MIF6	#######	0.584785	0.801	0.556	#######	NK	MIF
HIST1H1D	#######	0.710265	0.313	0.123	#######	NK	HIST1H1D
MT-CYB5	#######	0.500702	0.998	0.927	#######	NK	MT-CYB
EBP1	#######	0.531692	0.204	0.06	#######	NK	EBP
FAM107B3	#######	0.570788	0.55	0.305	#######	NK	FAM107B
OFD12	#######	0.578697	0.273	0.098	#######	NK	OFD1
STK101	#######	0.679548	0.502	0.275	#######	NK	STK10
MSN1	#######	0.552233	0.789	0.543	#######	NK	MSN
ZFYVE28	#######	0.340143	0.105	0.018	#######	NK	ZFYVE28
ARPC21	#######	0.483953	0.953	0.787	#######	NK	ARPC2
RAB9A1	#######	0.559978	0.283	0.107	#######	NK	RAB9A
DNAJC11	#######	0.621609	0.372	0.168	#######	NK	DNAJC1
FAM43A1	#######	0.443819	0.167	0.043	#######	NK	FAM43A
BSG1	#######	0.55413	0.566	0.331	#######	NK	BSG
PIK3R12	#######	0.661396	0.41	0.2	#######	NK	PIK3R1
HNRNPA2	#######	0.530007	0.92	0.703	#######	NK	HNRNPA2B1
IQGAP2	#######	0.490421	0.588	0.336	#######	NK	IQGAP2
TRIB21	#######	0.395193	0.134	0.029	#######	NK	TRIB2
ACTR32	#######	0.595365	0.75	0.52	#######	NK	ACTR3
MYLIP	#######	0.497578	0.225	0.072	#######	NK	MYLIP
YARS1	#######	0.537767	0.236	0.08	#######	NK	YARS

GPR141	#######	0.414053	0.143	0.032	#######	NK	GPR141
SPCS22	#######	0.610019	0.536	0.31	#######	NK	SPCS2
TSPAN32	#######	0.540835	0.28	0.106	#######	NK	TSPAN32
MEAF62	#######	0.607507	0.42	0.212	#######	NK	MEAF6
ITCH	#######	0.584834	0.336	0.144	#######	NK	ITCH
PTPN12	#######	0.473425	0.387	0.176	#######	NK	PTPN12
TNFSF141	#######	0.428895	0.182	0.05	#######	NK	TNFSF14
DDOST2	#######	0.668602	0.437	0.231	#######	NK	DDOST
SYNRG2	#######	0.645857	0.391	0.188	#######	NK	SYNRG
BHLHE40	#######	0.467347	0.167	0.044	#######	NK	BHLHE40
CERS41	#######	0.400045	0.135	0.03	#######	NK	CERS4
HNRNPDL	#######	0.586365	0.681	0.446	#######	NK	HNRNPDL
SOCS21	#######	0.296855	0.108	0.02	#######	NK	SOCS2
SACM1L	#######	0.555485	0.259	0.095	#######	NK	SACM1L
SCP21	#######	0.596598	0.465	0.254	#######	NK	SCP2
RBM382	#######	0.431633	0.314	0.127	#######	NK	RBM38
SET3	#######	0.548101	0.74	0.508	#######	NK	SET
SLC44A2	#######	0.326512	0.36	0.155	#######	NK	SLC44A2
MRFAP1L1	#######	0.572835	0.317	0.134	#######	NK	MRFAP1L1
MAP3K82	#######	0.595914	0.317	0.134	#######	NK	MAP3K8
CTDSP1	#######	0.57686	0.431	0.222	#######	NK	CTDSP1
PXN1	#######	0.570048	0.412	0.202	#######	NK	PXN
RP4-728D	#######	0.385485	0.132	0.03	#######	NK	RP4-728D4.2
RINL2	#######	0.492592	0.19	0.057	#######	NK	RINL
SDF2L11	#######	0.653083	0.337	0.151	#######	NK	SDF2L1
CD631	#######	0.453054	0.712	0.447	#######	NK	CD63
PAFAH2	#######	0.376999	0.125	0.027	#######	NK	PAFAH2
C12orf575	#######	0.450504	0.552	0.287	#######	NK	C12orf57
BTG15	#######	0.533939	0.893	0.67	#######	NK	BTG1
BANF12	#######	0.621551	0.53	0.317	#######	NK	BANF1
RBM39	#######	0.555899	0.685	0.461	#######	NK	RBM39
GBP42	#######	0.566677	0.308	0.127	#######	NK	GBP4
CSK2	#######	0.558537	0.652	0.423	#######	NK	CSK
ZFP36L21	#######	0.560412	0.885	0.641	#######	NK	ZFP36L2
NDUFV12	#######	0.623074	0.425	0.226	#######	NK	NDUFV1
SRGN	#######	0.262667	0.958	0.767	#######	NK	SRGN
STX8	#######	0.593444	0.318	0.14	#######	NK	STX8
GOLGA8B4	#######	0.422638	0.16	0.043	#######	NK	GOLGA8B
NDUFA124	#######	0.580523	0.574	0.354	#######	NK	NDUFA12
IL12RB11	#######	0.450463	0.174	0.05	#######	NK	IL12RB1
GPATCH8	#######	0.616831	0.311	0.133	#######	NK	GPATCH8
RPS27A5	#######	0.331103	0.979	0.861	#######	NK	RPS27A
DSTN	#######	0.474628	0.465	0.25	#######	NK	DSTN
VAV3	#######	0.504683	0.245	0.089	#######	NK	VAV3
TRAF52	#######	0.398541	0.136	0.032	#######	NK	TRAF5
MAPK1	#######	0.514672	0.486	0.27	#######	NK	MAPK1
ACTG11	#######	0.362624	0.976	0.873	#######	NK	ACTG1
CFL11	#######	0.356665	0.975	0.874	#######	NK	CFL1
PRKCQ-AS	#######	0.451231	0.283	0.107	#######	NK	PRKCQ-AS1
SUMO22	#######	0.470499	0.824	0.596	#######	NK	SUMO2
EPC15	#######	0.584661	0.492	0.284	#######	NK	EPC1
ARHGAP9	#######	0.556241	0.46	0.252	#######	NK	ARHGAP9
LPAR51	#######	0.281199	0.113	0.023	#######	NK	LPAR5
ICAM33	#######	0.538224	0.612	0.389	#######	NK	ICAM3
LMAN21	#######	0.562319	0.505	0.298	#######	NK	LMAN2
DRAP11	#######	0.498566	0.67	0.436	#######	NK	DRAP1
RASSF51	#######	0.558769	0.404	0.208	#######	NK	RASSF5
RPSA6	#######	0.372339	0.939	0.684	#######	NK	RPSA

CYTH1	#######	0.60534	0.483	0.278	#######	NK	CYTH1
TSPAN51	#######	0.337309	0.118	0.026	#######	NK	TSPAN5
MACF1	#######	0.602954	0.439	0.235	#######	NK	MACF1
NPC1	#######	0.440014	0.159	0.044	#######	NK	NPC1
TAF74	#######	0.560038	0.402	0.207	#######	NK	TAF7
DDX64	#######	0.584304	0.358	0.173	#######	NK	DDX6
LINC00623	#######	0.408392	0.16	0.045	#######	NK	LINC00623
NEDD91	#######	0.504365	0.238	0.089	#######	NK	NEDD9
DTNBP1	#######	0.511623	0.237	0.089	#######	NK	DTNBP1
DERL11	#######	0.522933	0.303	0.133	#######	NK	DERL1
POLR2G	#######	0.500795	0.44	0.242	#######	NK	POLR2G
ADA2	#######	0.470957	0.19	0.062	#######	NK	ADA
TC2N4	#######	0.504222	0.273	0.106	#######	NK	TC2N
GIMAP62	#######	0.570328	0.323	0.147	#######	NK	GIMAP6
CASP81	#######	0.586585	0.321	0.147	#######	NK	CASP8
NT5C3	#######	0.55529	0.328	0.151	#######	NK	NT5C
BATF1	#######	0.504475	0.259	0.102	#######	NK	BATF
NDUFB71	#######	0.508125	0.607	0.394	#######	NK	NDUFB7
TRPV21	#######	0.540627	0.243	0.095	#######	NK	TRPV2
CYCS2	#######	0.543259	0.4	0.212	#######	NK	CYCS
EIF5A5	#######	0.587226	0.597	0.395	#######	NK	EIF5A
BST21	#######	0.54181	0.63	0.408	#######	NK	BST2
TBCB2	#######	0.52227	0.52	0.314	#######	NK	TBCB
ARF62	#######	0.612799	0.448	0.258	#######	NK	ARF6
ZMYM6NE	#######	0.560731	0.291	0.128	#######	NK	ZMYM6NB
RNPS12	#######	0.53269	0.46	0.264	#######	NK	RNPS1
HMGN16	#######	0.435685	0.579	0.346	#######	NK	HMGN1
FBXO6	#######	0.482605	0.191	0.064	#######	NK	FBXO6
LIME14	#######	0.501269	0.244	0.091	#######	NK	LIME1
GSAP	#######	0.440042	0.294	0.125	#######	NK	GSAP
ADD32	#######	0.492735	0.547	0.333	#######	NK	ADD3
RPS296	#######	0.339137	0.954	0.754	#######	NK	RPS29
HSP90AA1	#######	0.48307	0.825	0.603	#######	NK	HSP90AA1
AAK14	#######	0.525919	0.453	0.251	#######	NK	AAK1
RP11-100(	#######	0.55432	0.315	0.145	#######	NK	RP11-1000B6.3
DAXX1	#######	0.522986	0.311	0.142	#######	NK	DAXX
MTG12	#######	0.497756	0.237	0.092	#######	NK	MTG1
SELK2	#######	0.54105	0.415	0.228	#######	NK	SELK
PSMA51	#######	0.571223	0.474	0.281	#######	NK	PSMA5
UBC2	#######	0.411222	0.931	0.748	#######	NK	UBC
TMEM591	#######	0.475076	0.618	0.406	#######	NK	TMEM59
PPP2R5C1	#######	0.548738	0.386	0.198	#######	NK	PPP2R5C
ZMYND11	#######	0.448536	0.19	0.064	#######	NK	ZMYND11
C14orf12	#######	0.422137	0.172	0.054	#######	NK	C14orf1
ACTN41	#######	0.492992	0.487	0.282	#######	NK	ACTN4
INPP4A	#######	0.57103	0.308	0.142	#######	NK	INPP4A
YWHAQ1	#######	0.460542	0.46	0.265	#######	NK	YWHAQ
ECH12	#######	0.532787	0.469	0.278	#######	NK	ECH1
TMC63	#######	0.556269	0.321	0.152	#######	NK	TMC6
PNRC14	#######	0.504368	0.863	0.657	#######	NK	PNRC1
MED105	#######	0.543232	0.37	0.192	#######	NK	MED10
CCSER22	#######	0.514907	0.244	0.097	#######	NK	CCSER2
CCT26	#######	0.556948	0.377	0.199	#######	NK	CCT2
ASCL2	#######	0.545416	0.225	0.086	#######	NK	ASCL2
UAP1	#######	0.300226	0.1	0.021	#######	NK	UAP1
NPM16	#######	0.387619	0.772	0.51	#######	NK	NPM1
H3F3B	#######	0.373595	0.962	0.819	#######	NK	H3F3B
MAP2K21	#######	0.539126	0.484	0.294	#######	NK	MAP2K2

SUB12	#######	0.430922	0.759	0.541	#######	NK	SUB1
HNRNPA0	#######	0.556599	0.47	0.281	#######	NK	HNRNPA0
ITM2A4	#######	0.42621	0.261	0.104	#######	NK	ITM2A
HNRNPL3	#######	0.555359	0.486	0.295	#######	NK	HNRNPL
LRP10	#######	0.481963	0.374	0.194	#######	NK	LRP10
IDI1	#######	0.459525	0.265	0.112	#######	NK	IDI1
KAT6B	#######	0.507111	0.219	0.083	#######	NK	KAT6B
ATPIF11	#######	0.501646	0.6	0.402	#######	NK	ATPIF1
HAVCR21	#######	0.492126	0.197	0.071	#######	NK	HAVCR2
ARHGDIB1	#######	0.280895	0.943	0.779	#######	NK	ARHGDIB
NUCB23	#######	0.538052	0.328	0.156	#######	NK	NUCB2
SIRT2	#######	0.439891	0.266	0.114	#######	NK	SIRT2
TMED101	#######	0.545916	0.454	0.267	#######	NK	TMED10
RAN6	#######	0.494568	0.647	0.442	#######	NK	RAN
PPP1R12A	#######	0.444518	0.538	0.336	#######	NK	PPP1R12A
TECR4	#######	0.61326	0.395	0.219	#######	NK	TECR
PUF601	#######	0.537407	0.355	0.185	#######	NK	PUF60
WIPF1	#######	0.374535	0.574	0.367	#######	NK	WIPF1
LPIN11	#######	0.456603	0.192	0.068	#######	NK	LPIN1
CELF2	#######	0.511188	0.583	0.39	#######	NK	CELF2
RBM8A1	#######	0.480752	0.535	0.342	#######	NK	RBM8A
BCL2L11	#######	0.271047	0.248	0.099	#######	NK	BCL2L1
AKR1B13	#######	0.50648	0.293	0.135	#######	NK	AKR1B1
DNAJB141	#######	0.515001	0.339	0.17	#######	NK	DNAJB14
LRRC8A	#######	0.358807	0.124	0.033	#######	NK	LRRC8A
ARIH21	#######	0.542154	0.337	0.17	#######	NK	ARIH2
CTBP22	#######	0.505294	0.299	0.14	#######	NK	CTBP2
SHISA51	#######	0.520883	0.452	0.267	#######	NK	SHISA5
IDS	#######	0.522246	0.407	0.226	#######	NK	IDS
EPB414	#######	0.449737	0.379	0.198	#######	NK	EPB41
ITGA63	#######	0.4018	0.168	0.054	#######	NK	ITGA6
PRPF38B	#######	0.549329	0.439	0.257	#######	NK	PRPF38B
HIST1H1E	#######	0.662065	0.348	0.177	#######	NK	HIST1H1E
PTGER41	#######	0.538307	0.27	0.12	#######	NK	PTGER4
URI15	#######	0.551931	0.354	0.185	#######	NK	URI1
SNRK	#######	0.489367	0.282	0.128	#######	NK	SNRK
SAMD91	#######	0.537813	0.313	0.152	#######	NK	SAMD9
MIEN11	#######	0.496397	0.464	0.28	#######	NK	MIEN1
MNAT1	#######	0.39447	0.151	0.047	#######	NK	MNAT1
CLDND12	#######	0.545867	0.275	0.125	#######	NK	CLDND1
NAP1L43	#######	0.470118	0.439	0.256	#######	NK	NAP1L4
ODF2L2	#######	0.442029	0.194	0.071	#######	NK	ODF2L
PSME22	#######	0.431131	0.73	0.51	#######	NK	PSME2
TERF2IP2	#######	0.406992	0.504	0.313	#######	NK	TERF2IP
DAD12	#######	0.423674	0.583	0.394	#######	NK	DAD1
PPP2R1A	#######	0.504467	0.44	0.263	#######	NK	PPP2R1A
INPP5D	#######	0.51071	0.382	0.209	#######	NK	INPP5D
UBE2V11	#######	0.47536	0.457	0.275	#######	NK	UBE2V1
CDC25B4	#######	0.465481	0.208	0.079	#######	NK	CDC25B
PSMA62	#######	0.4518	0.512	0.323	#######	NK	PSMA6
SRSF111	#######	0.50255	0.574	0.384	#######	NK	SRSF11
CMKLR1	#######	0.326893	0.105	0.025	#######	NK	CMKLR1
C19orf661	#######	0.509354	0.276	0.126	#######	NK	C19orf66
LSP13	#######	0.336257	0.888	0.656	#######	NK	LSP1
KMT2E2	#######	0.508954	0.557	0.366	#######	NK	KMT2E
APOBEC3C	#######	0.509641	0.291	0.138	#######	NK	APOBEC3C
PRMT15	#######	0.532345	0.319	0.161	#######	NK	PRMT1
MRPL101	#######	0.475872	0.216	0.086	#######	NK	MRPL10

TADA3	#######	0.439961	0.366	0.196	#######	NK	TADA3
RASGRP14	#######	0.374341	0.151	0.047	#######	NK	RASGRP1
VPS37B	#######	0.41804	0.226	0.09	#######	NK	VPS37B
RP11-640I	#######	0.361413	0.161	0.052	#######	NK	RP11-640M9.1
ERN12	#######	0.464181	0.221	0.088	#######	NK	ERN1
THRAP32	#######	0.499208	0.504	0.322	#######	NK	THRAP3
IVNS1ABP	#######	0.503546	0.298	0.142	#######	NK	IVNS1ABP
RBBP72	#######	0.481631	0.298	0.144	#######	NK	RBBP7
MGAT4A4	#######	0.456093	0.249	0.106	#######	NK	MGAT4A
CTBP1	#######	0.51254	0.342	0.18	#######	NK	CTBP1
DIAPH1	#######	0.367118	0.493	0.302	#######	NK	DIAPH1
SPTAN12	#######	0.550038	0.311	0.153	#######	NK	SPTAN1
ARNTL	#######	0.415254	0.184	0.066	#######	NK	ARNTL
PRKD22	#######	0.380252	0.184	0.066	#######	NK	PRKD2
GPRIN33	#######	0.5147	0.231	0.098	#######	NK	GPRIN3
VPS13A1	#######	0.377307	0.164	0.055	#######	NK	VPS13A
PSMB11	#######	0.428732	0.617	0.425	#######	NK	PSMB1
SNRPB3	#######	0.514096	0.495	0.315	#######	NK	SNRPB
SRP94	#######	0.467152	0.455	0.279	#######	NK	SRP9
PIK3R5	#######	0.460467	0.277	0.128	#######	NK	PIK3R5
ATP8B4	#######	0.30885	0.125	0.035	#######	NK	ATP8B4
KRT105	#######	0.484508	0.498	0.316	#######	NK	KRT10
TSEN15	#######	0.414795	0.189	0.071	#######	NK	TSEN15
TROVE21	#######	0.481698	0.33	0.17	#######	NK	TROVE2
RPS197	#######	0.363753	0.974	0.805	#######	NK	RPS19
IDH23	#######	0.509679	0.363	0.2	#######	NK	IDH2
SPOCK24	#######	0.527253	0.264	0.115	#######	NK	SPOCK2
ZBTB381	#######	0.508622	0.272	0.127	#######	NK	ZBTB38
SLC25A38:	#######	0.346427	0.144	0.045	#######	NK	SLC25A38
MATR34	#######	0.491002	0.51	0.329	#######	NK	MATR3
HNRNPR1	#######	0.496907	0.467	0.292	#######	NK	HNRNPR
APBB1IP1	#######	0.45769	0.612	0.422	#######	NK	APBB1IP
AKNA1	#######	0.483976	0.461	0.281	#######	NK	AKNA
GIPC11	#######	0.310504	0.12	0.034	#######	NK	GIPC1
ARHGAP1!	#######	0.436768	0.371	0.196	#######	NK	ARHGAP15
OCIAD25	#######	0.428123	0.235	0.098	#######	NK	OCIAD2
TRANK12	#######	0.457466	0.237	0.102	#######	NK	TRANK1
C19orf101	#######	0.516432	0.388	0.226	#######	NK	C19orf10
DCAF5	#######	0.451666	0.229	0.097	#######	NK	DCAF5
GPR89B	#######	0.299581	0.105	0.027	#######	NK	GPR89B
DNAJC8	#######	0.435341	0.547	0.364	#######	NK	DNAJC8
YPEL31	#######	0.379471	0.698	0.507	#######	NK	YPEL3
SNRPD25	#######	0.376355	0.775	0.563	#######	NK	SNRPD2
P4HB	#######	0.42904	0.535	0.351	#######	NK	P4HB
GUK12	#######	0.379621	0.692	0.497	#######	NK	GUK1
EWSR1	#######	0.503474	0.442	0.272	#######	NK	EWSR1
SP1403	#######	0.421909	0.185	0.069	#######	NK	SP140
UBLCP11	#######	0.45471	0.283	0.138	#######	NK	UBLCP1
USB1	#######	0.426988	0.216	0.09	#######	NK	USB1
CYB561D2	#######	0.500883	0.288	0.143	#######	NK	CYB561D2
GLS3	#######	0.493144	0.284	0.139	#######	NK	GLS
VPS26A	#######	0.487006	0.278	0.135	#######	NK	VPS26A
NUCKS13	#######	0.502579	0.443	0.273	#######	NK	NUCKS1
EIF4A25	#######	0.484678	0.47	0.295	#######	NK	EIF4A2
ORAI12	#######	0.415629	0.393	0.225	#######	NK	ORAI1
BRD2	#######	0.489474	0.454	0.283	#######	NK	BRD2
TOMM51	#######	0.427902	0.431	0.261	#######	NK	TOMM5
FNTA3	#######	0.522633	0.365	0.206	#######	NK	FNTA

CCT76	#######	0.507102	0.386	0.226	#######	NK	CCT7
TAPSAR15	#######	0.511654	0.395	0.231	#######	NK	TAPSAR1
WDSUB1	#######	0.34038	0.133	0.041	#######	NK	WDSUB1
C1orf174	#######	0.404054	0.192	0.076	#######	NK	C1orf174
BCL7C1	#######	0.473136	0.355	0.196	#######	NK	BCL7C
KIAA1430	#######	0.457206	0.219	0.093	#######	NK	KIAA1430
HNRNPA3	#######	0.442112	0.632	0.448	#######	NK	HNRNPA3
HSPB112	#######	0.48596	0.275	0.134	#######	NK	HSPB11
NMT23	#######	0.299334	0.108	0.029	#######	NK	NMT2
MAD2L2	#######	0.467756	0.257	0.121	#######	NK	MAD2L2
FHL3	#######	0.37033	0.246	0.11	#######	NK	FHL3
PCSK7	#######	0.485335	0.295	0.147	#######	NK	PCSK7
RBM44	#######	0.47083	0.336	0.182	#######	NK	RBM4
LSM22	#######	0.450788	0.362	0.203	#######	NK	LSM2
EIF5	#######	0.421899	0.521	0.348	#######	NK	EIF5
IMMP1L	#######	0.316684	0.121	0.035	#######	NK	IMMP1L
SAP182	#######	0.385254	0.595	0.414	#######	NK	SAP18
XRCC64	#######	0.448596	0.527	0.351	#######	NK	XRCC6
FDPS	#######	0.489782	0.295	0.152	#######	NK	FDPS
FDFT12	#######	0.498614	0.365	0.207	#######	NK	FDFT1
RP11-47L3	#######	0.291667	0.112	0.031	#######	NK	RP11-47L3.1
UFC12	#######	0.469587	0.524	0.353	#######	NK	UFC1
SP4	#######	0.321647	0.119	0.034	#######	NK	SP4
HDAC14	#######	0.484821	0.366	0.209	#######	NK	HDAC1
SIGLEC71	#######	0.471417	0.238	0.107	#######	NK	SIGLEC7
ZCCHC11	#######	0.469883	0.273	0.131	#######	NK	ZCCHC11
PDIA41	#######	0.492445	0.272	0.134	#######	NK	PDIA4
KDM4C1	#######	0.421596	0.212	0.091	#######	NK	KDM4C
SKP12	#######	0.311237	0.759	0.561	#######	NK	SKP1
ZNF841	#######	0.3259	0.115	0.033	#######	NK	ZNF841
TMEM223	#######	0.401504	0.191	0.077	#######	NK	TMEM223
PDE4D	#######	0.263661	0.121	0.036	#######	NK	PDE4D
UBE2D25	#######	0.400482	0.59	0.412	#######	NK	UBE2D2
GNGT21	#######	0.343795	0.119	0.036	#######	NK	GNGT2
HCG18	#######	0.371126	0.159	0.057	#######	NK	HCG18
SSBP42	#######	0.491867	0.334	0.185	#######	NK	SSBP4
LIMA11	#######	0.317494	0.104	0.028	#######	NK	LIMA1
PCYT2	#######	0.29589	0.116	0.034	#######	NK	PCYT2
RNF115	#######	0.408232	0.262	0.126	#######	NK	RNF115
TINF21	#######	0.423499	0.291	0.149	#######	NK	TINF2
SP1001	#######	0.406408	0.529	0.355	#######	NK	SP100
ACP12	#######	0.412617	0.388	0.231	#######	NK	ACP1
CCT36	#######	0.50499	0.391	0.236	#######	NK	CCT3
G6PD	#######	0.32213	0.402	0.234	#######	NK	G6PD
SF3B2	#######	0.38491	0.613	0.437	#######	NK	SF3B2
PHB3	#######	0.488749	0.329	0.183	#######	NK	PHB
ARL6IP5	#######	0.31277	0.756	0.563	#######	NK	ARL6IP5
RPAP21	#######	0.384104	0.169	0.064	#######	NK	RPAP2
NCOR1	#######	0.393894	0.519	0.345	#######	NK	NCOR1
RPUSD3	#######	0.430809	0.21	0.091	#######	NK	RPUSD3
OBFC1	#######	0.389773	0.181	0.072	1.01E-99	NK	OBFC1
ANKRD44	#######	0.484069	0.383	0.226	2.49E-99	NK	ANKRD44
FAM8A1	#######	0.305402	0.131	0.042	2.68E-99	NK	FAM8A1
FPGS	#######	0.425308	0.193	0.081	4.65E-99	NK	FPGS
TRIM222	#######	0.470165	0.412	0.251	5.76E-99	NK	TRIM22
MCM32	#######	0.373733	0.161	0.06	9.96E-99	NK	MCM3
TCEAL8	#######	0.401103	0.217	0.096	1.08E-98	NK	TCEAL8
ABLIM14	#######	0.334716	0.209	0.087	2.18E-98	NK	ABLIM1

CUTA4	#######	0.414429	0.547	0.376	3.54E-98	NK	CUTA
LSM14A1	#######	0.45251	0.423	0.266	9.26E-98	NK	LSM14A
ATXN103	#######	0.468497	0.326	0.182	9.40E-98	NK	ATXN10
FAM172A	#######	0.38092	0.207	0.089	9.78E-98	NK	FAM172A
MAGOH1	#######	0.452384	0.38	0.229	2.17E-97	NK	MAGOH
ANKRD36(	#######	0.323463	0.12	0.037	3.19E-97	NK	ANKRD36C
PPIH4	#######	0.437418	0.24	0.114	3.89E-97	NK	PPIH
VCP1	#######	0.398991	0.487	0.321	4.14E-97	NK	VCP
TAPBP1	#######	0.374887	0.584	0.404	4.92E-97	NK	TAPBP
FAM3C1	#######	0.347484	0.146	0.051	8.37E-97	NK	FAM3C
CHCHD22	#######	0.333622	0.808	0.612	8.78E-97	NK	CHCHD2
MEI12	#######	0.3238	0.127	0.041	9.25E-97	NK	MEI1
ANAPC16!	#######	0.437361	0.491	0.329	1.05E-96	NK	ANAPC16
KCTD10	#######	0.290811	0.131	0.043	1.10E-96	NK	KCTD10
ANXA111	#######	0.337442	0.589	0.416	1.13E-96	NK	ANXA11
PIP4K2A	#######	0.303826	0.288	0.146	1.41E-96	NK	PIP4K2A
PWP2	#######	0.307044	0.12	0.038	1.62E-96	NK	PWP2
PSMD83	#######	0.433928	0.46	0.301	1.89E-96	NK	PSMD8
GIMAP14	#######	0.41698	0.578	0.401	1.95E-96	NK	GIMAP1
IKZF13	#######	0.444346	0.503	0.333	2.96E-96	NK	IKZF1
SLC25A20	#######	0.374873	0.16	0.06	3.06E-96	NK	SLC25A20
SERBP11	#######	0.381752	0.676	0.494	3.17E-96	NK	SERBP1
ZNF8001	#######	0.449487	0.314	0.171	5.26E-96	NK	ZNF800
UBE2I1	#######	0.382644	0.545	0.377	2.84E-95	NK	UBE2I
PRR42	#######	0.379272	0.167	0.065	2.85E-95	NK	PRR4
PPP5C	#######	0.385058	0.166	0.065	3.07E-95	NK	PPP5C
CRELD1	#######	0.294124	0.117	0.036	3.15E-95	NK	CRELD1
ANXA2R4	1.67E-99	0.405038	0.197	0.083	5.47E-95	NK	ANXA2R
APOA1BP1	2.36E-99	0.441284	0.304	0.164	7.72E-95	NK	APOA1BP
SRSF91	2.47E-99	0.369625	0.623	0.449	8.08E-95	NK	SRSF9
NPRL3	7.31E-99	0.30789	0.144	0.05	2.39E-94	NK	NPRL3
STIM2	1.30E-98	0.418806	0.192	0.081	4.24E-94	NK	STIM2
CCDC121	1.52E-98	0.452565	0.406	0.253	4.97E-94	NK	CCDC12
KRTCAP21	2.03E-98	0.343086	0.666	0.486	6.65E-94	NK	KRTCAP2
TSR21	2.95E-98	0.401133	0.217	0.098	9.67E-94	NK	TSR2
TMCO11	3.08E-98	0.435472	0.444	0.286	1.01E-93	NK	TMCO1
CDC1233	5.26E-98	0.438247	0.282	0.148	1.72E-93	NK	CDC123
GLMN	8.85E-98	0.307442	0.117	0.037	2.90E-93	NK	GLMN
SRPR1	2.43E-97	0.440936	0.392	0.24	7.94E-93	NK	SRPR
COPS61	2.63E-97	0.426615	0.368	0.22	8.60E-93	NK	COPS6
PLCB11	2.67E-97	0.323543	0.194	0.081	8.73E-93	NK	PLCB1
TSC22D41	3.64E-97	0.411018	0.374	0.225	1.19E-92	NK	TSC22D4
PIN11	4.33E-97	0.4584	0.338	0.196	1.42E-92	NK	PIN1
CARHSP1	6.15E-97	0.408146	0.278	0.143	2.01E-92	NK	CARHSP1
ITGA42	8.00E-97	0.471977	0.478	0.308	2.62E-92	NK	ITGA4
KCNAB2	1.32E-96	0.492434	0.352	0.207	4.32E-92	NK	KCNAB2
TMEM2	1.95E-96	0.430407	0.214	0.096	6.39E-92	NK	TMEM2
TMEM665	3.04E-96	0.253559	0.767	0.537	9.96E-92	NK	TMEM66
ZRANB23	3.11E-96	0.419808	0.428	0.269	1.02E-91	NK	ZRANB2
DYRK24	3.79E-96	0.4086	0.164	0.064	1.24E-91	NK	DYRK2
GLG1	6.53E-96	0.481933	0.398	0.247	2.14E-91	NK	GLG1
COMMD7	9.15E-96	0.370916	0.357	0.209	3.00E-91	NK	COMMD7
PRRC2C	1.16E-95	0.384747	0.64	0.465	3.79E-91	NK	PRRC2C
GPBP1	2.38E-95	0.404924	0.386	0.234	7.80E-91	NK	GPBP1
UBE2L62	3.45E-95	0.403159	0.476	0.312	1.13E-90	NK	UBE2L6
DGKZ	6.50E-95	0.402772	0.414	0.257	2.13E-90	NK	DGKZ
DCXR	8.10E-95	0.423485	0.392	0.241	2.65E-90	NK	DCXR
COX6C5	1.05E-94	0.354594	0.759	0.569	3.42E-90	NK	COX6C

IFNAR21	1.75E-94	0.460709	0.324	0.185	5.74E-90	NK	IFNAR2
RBBP4	1.94E-94	0.427976	0.429	0.276	6.36E-90	NK	RBBP4
NCK11	2.94E-94	0.425615	0.257	0.129	9.64E-90	NK	NCK1
BEX42	4.65E-94	0.337851	0.194	0.084	1.52E-89	NK	BEX4
ATP5G33	1.14E-93	0.335965	0.677	0.482	3.73E-89	NK	ATP5G3
LAMP1	1.60E-93	0.351074	0.431	0.272	5.25E-89	NK	LAMP1
VPS16	1.97E-93	0.414898	0.179	0.075	6.45E-89	NK	VPS16
AMZ21	3.70E-93	0.380988	0.195	0.085	1.21E-88	NK	AMZ2
MOAP1	3.96E-93	0.322578	0.136	0.048	1.30E-88	NK	MOAP1
B4GALT4	4.48E-93	0.279302	0.101	0.029	1.47E-88	NK	B4GALT4
SRSF76	4.79E-93	0.438704	0.494	0.335	1.57E-88	NK	SRSF7
RHOH5	4.95E-93	0.372818	0.228	0.103	1.62E-88	NK	RHOH
SNRNP402	9.51E-93	0.429496	0.251	0.127	3.11E-88	NK	SNRNP40
POLR2K1	9.98E-93	0.443235	0.282	0.152	3.27E-88	NK	POLR2K
SMARCE12	1.14E-92	0.437462	0.335	0.195	3.75E-88	NK	SMARCE1
RPL36AL4	1.58E-92	0.327565	0.9	0.711	5.17E-88	NK	RPL36AL
CD2BP2	1.64E-92	0.40513	0.284	0.151	5.36E-88	NK	CD2BP2
SRSF53	2.12E-92	0.395994	0.621	0.455	6.95E-88	NK	SRSF5
HMGN43	2.27E-92	0.414645	0.208	0.096	7.44E-88	NK	HMGN4
EMC41	2.32E-92	0.450986	0.322	0.187	7.59E-88	NK	EMC4
LRBA1	2.91E-92	0.318832	0.178	0.073	9.51E-88	NK	LRBA
MDH14	5.18E-92	0.433257	0.37	0.227	1.70E-87	NK	MDH1
SIPA11	5.53E-92	0.40378	0.329	0.186	1.81E-87	NK	SIPA1
AC093323	1.08E-91	0.275842	0.119	0.039	3.55E-87	NK	AC093323.3
LMAN12	1.16E-91	0.411126	0.274	0.143	3.80E-87	NK	LMAN1
SRI2	1.99E-91	0.383183	0.428	0.274	6.51E-87	NK	SRI
TMX4	2.18E-91	0.40674	0.332	0.189	7.13E-87	NK	TMX4
SDF2	2.33E-91	0.41499	0.264	0.137	7.61E-87	NK	SDF2
COQ10B	2.87E-91	0.359992	0.194	0.085	9.41E-87	NK	COQ10B
JADE21	3.19E-91	0.371909	0.158	0.062	1.04E-86	NK	JADE2
ITGB1BP11	3.47E-91	0.428467	0.303	0.169	1.14E-86	NK	ITGB1BP1
PDCD71	3.66E-91	0.424868	0.266	0.139	1.20E-86	NK	PDCD7
RPL7A6	5.21E-91	0.289466	0.978	0.83	1.71E-86	NK	RPL7A
KIF21B	6.90E-91	0.288261	0.123	0.041	2.26E-86	NK	KIF21B
SESN14	1.23E-90	0.335802	0.158	0.061	4.04E-86	NK	SESN1
ZCCHC17	2.33E-90	0.339812	0.249	0.124	7.63E-86	NK	ZCCHC17
SF13	2.57E-90	0.356022	0.571	0.407	8.41E-86	NK	SF1
ABCF11	4.15E-90	0.464102	0.33	0.191	1.36E-85	NK	ABCF1
PPM1L	5.35E-90	0.355729	0.128	0.044	1.75E-85	NK	PPM1L
TBCC3	5.49E-90	0.401055	0.216	0.102	1.80E-85	NK	TBCC
UBE2V2	5.97E-90	0.406034	0.248	0.126	1.95E-85	NK	UBE2V2
RPL176	1.19E-89	0.331709	0.903	0.704	3.91E-85	NK	RPL17
TMED21	1.74E-89	0.439252	0.369	0.228	5.70E-85	NK	TMED2
DNMT11	2.19E-89	0.466867	0.302	0.169	7.17E-85	NK	DNMT1
TAPBPL	2.93E-89	0.410467	0.194	0.087	9.59E-85	NK	TAPBPL
UBXN4	2.99E-89	0.391032	0.531	0.37	9.77E-85	NK	UBXN4
CREM	4.21E-89	0.277951	0.109	0.035	1.38E-84	NK	CREM
SPCS12	5.18E-89	0.338232	0.588	0.421	1.70E-84	NK	SPCS1
CRELD21	7.61E-89	0.400396	0.19	0.084	2.49E-84	NK	CRELD2
BTN3A31	8.40E-89	0.39968	0.194	0.086	2.75E-84	NK	BTN3A3
PCNXL4	9.45E-89	0.290111	0.124	0.042	3.09E-84	NK	PCNXL4
ENSA	9.64E-89	0.391061	0.407	0.26	3.15E-84	NK	ENSA
EDF11	2.06E-88	0.331353	0.717	0.542	6.74E-84	NK	EDF1
MTSS12	3.56E-88	0.411345	0.281	0.144	1.17E-83	NK	MTSS1
C19orf431	4.37E-88	0.329087	0.761	0.582	1.43E-83	NK	C19orf43
CA5B1	1.51E-87	0.379833	0.178	0.076	4.94E-83	NK	CA5B
KEAP1	1.87E-87	0.36361	0.181	0.078	6.12E-83	NK	KEAP1
SPSB31	2.07E-87	0.448549	0.326	0.19	6.78E-83	NK	SPSB3

APOL61	2.55E-87	0.426814	0.359	0.214	8.35E-83	NK	APOL6
XRCC5	2.78E-87	0.360504	0.582	0.421	9.11E-83	NK	XRCC5
KDELR2	3.10E-87	0.411203	0.35	0.211	1.02E-82	NK	KDELR2
BLOC1S42	5.43E-87	0.404074	0.253	0.131	1.78E-82	NK	BLOC1S4
PSMC43	8.23E-87	0.416823	0.299	0.17	2.69E-82	NK	PSMC4
N4BP2L22	8.24E-87	0.368983	0.523	0.367	2.70E-82	NK	N4BP2L2
C11orf48	8.39E-87	0.399054	0.348	0.207	2.75E-82	NK	C11orf48
GTF3C1	8.74E-87	0.389371	0.193	0.086	2.86E-82	NK	GTF3C1
RNF51	1.83E-86	0.412679	0.266	0.142	6.00E-82	NK	RNF5
GTF3A3	5.12E-86	0.412792	0.491	0.339	1.68E-81	NK	GTF3A
UHMK1	7.17E-86	0.432807	0.323	0.189	2.35E-81	NK	UHMK1
KRR15	1.24E-85	0.380972	0.191	0.086	4.06E-81	NK	KRR1
ABT12	1.34E-85	0.407184	0.23	0.115	4.39E-81	NK	ABT1
ARID4B	2.09E-85	0.419101	0.429	0.282	6.85E-81	NK	ARID4B
COPS81	2.70E-85	0.362458	0.187	0.084	8.85E-81	NK	COPS8
C11orf581	8.93E-85	0.31307	0.611	0.451	2.92E-80	NK	C11orf58
NCOA71	1.05E-84	0.405581	0.237	0.119	3.45E-80	NK	NCOA7
RAP1GDS1	1.70E-84	0.393679	0.22	0.107	5.56E-80	NK	RAP1GDS1
SUMO11	1.96E-84	0.37129	0.426	0.283	6.43E-80	NK	SUMO1
TNRC6C1	2.59E-84	0.285202	0.114	0.038	8.48E-80	NK	TNRC6C
SEP151	2.84E-84	0.353997	0.421	0.278	9.30E-80	NK	15-Sep
MPHOSPH	7.88E-84	0.383454	0.492	0.339	2.58E-79	NK	MPHOSPH8
SMIM19	8.67E-84	0.385801	0.235	0.119	2.84E-79	NK	SMIM19
IL27RA1	9.32E-84	0.452775	0.299	0.17	3.05E-79	NK	IL27RA
PPIE	1.11E-83	0.377235	0.214	0.103	3.63E-79	NK	PPIE
MOB41	1.95E-83	0.372197	0.228	0.114	6.38E-79	NK	MOB4
SEC11C2	4.32E-83	0.444841	0.306	0.177	1.42E-78	NK	SEC11C
MYO1G2	4.33E-83	0.362096	0.514	0.353	1.42E-78	NK	MYO1G
VAMP2	4.86E-83	0.37533	0.323	0.187	1.59E-78	NK	VAMP2
JAZF11	6.82E-83	0.372335	0.212	0.101	2.23E-78	NK	JAZF1
FAM65B1	9.07E-83	0.319335	0.623	0.455	2.97E-78	NK	FAM65B
SLC35B2	2.86E-82	0.282637	0.121	0.043	9.35E-78	NK	SLC35B2
NR3C1	3.37E-82	0.393082	0.435	0.29	1.10E-77	NK	NR3C1
RNF19A	8.64E-82	0.395783	0.221	0.108	2.83E-77	NK	RNF19A
G3BP13	1.13E-81	0.445649	0.322	0.192	3.71E-77	NK	G3BP1
PIM25	1.38E-81	0.33859	0.175	0.075	4.52E-77	NK	PIM2
PGM1	1.86E-81	0.329144	0.169	0.073	6.10E-77	NK	PGM1
ZNF720	2.67E-81	0.303083	0.123	0.044	8.74E-77	NK	ZNF720
DDX244	3.90E-81	0.380288	0.513	0.358	1.28E-76	NK	DDX24
GOLGA7	5.51E-81	0.369765	0.331	0.198	1.80E-76	NK	GOLGA7
GLT8D1	9.12E-81	0.332402	0.148	0.059	2.99E-76	NK	GLT8D1
HSPA92	1.16E-80	0.425134	0.319	0.19	3.79E-76	NK	HSPA9
PRDX5	1.45E-80	0.269295	0.6	0.443	4.75E-76	NK	PRDX5
PA2G41	1.49E-80	0.350365	0.56	0.413	4.88E-76	NK	PA2G4
FNDC3B1	3.73E-80	0.333112	0.235	0.118	1.22E-75	NK	FNDC3B
EML2	7.79E-80	0.320674	0.137	0.053	2.55E-75	NK	EML2
ARHGDIA	1.35E-79	0.323542	0.536	0.383	4.44E-75	NK	ARHGDIA
FTSJ1	1.99E-79	0.328339	0.156	0.066	6.52E-75	NK	FTSJ1
COPZ1	2.05E-79	0.417261	0.347	0.217	6.70E-75	NK	COPZ1
ATXN7L3B	2.15E-79	0.411511	0.27	0.15	7.03E-75	NK	ATXN7L3B
DENND4C	2.77E-79	0.307702	0.184	0.083	9.06E-75	NK	DENND4C
TRAPPC6A	3.60E-79	0.430642	0.263	0.146	1.18E-74	NK	TRAPPC6A
RP11-488I	3.86E-79	0.306165	0.138	0.054	1.26E-74	NK	RP11-488L18.8
TCF251	4.19E-79	0.320615	0.664	0.505	1.37E-74	NK	TCF25
TFIP11	5.36E-79	0.339801	0.159	0.067	1.76E-74	NK	TFIP11
ATG4D	1.06E-78	0.31445	0.135	0.052	3.47E-74	NK	ATG4D
STK39	1.33E-78	0.273573	0.114	0.04	4.35E-74	NK	STK39
ATP5B2	1.96E-78	0.319427	0.655	0.479	6.43E-74	NK	ATP5B

NAE12	2.00E-78	0.326664	0.15	0.061	6.55E-74	NK	NAE1
SEPW11	2.02E-78	0.3208	0.501	0.347	6.62E-74	NK	SEPW1
GORASP22	2.08E-78	0.378283	0.236	0.124	6.82E-74	NK	GORASP2
LAIR1	3.48E-78	0.402623	0.284	0.159	1.14E-73	NK	LAIR1
RPS6KA11	3.88E-78	0.311529	0.4	0.257	1.27E-73	NK	RPS6KA1
WHAMM	4.15E-78	0.378786	0.2	0.095	1.36E-73	NK	WHAMM
SF3B1	4.83E-78	0.360742	0.518	0.37	1.58E-73	NK	SF3B1
DGUOK	1.21E-77	0.407315	0.374	0.243	3.96E-73	NK	DGUOK
TMED42	1.66E-77	0.394119	0.299	0.175	5.43E-73	NK	TMED4
NDNL24	2.08E-77	0.371861	0.201	0.097	6.79E-73	NK	NDNL2
TMEM9B	2.27E-77	0.364362	0.296	0.173	7.43E-73	NK	TMEM9B
PPM1K5	2.58E-77	0.353811	0.217	0.106	8.43E-73	NK	PPM1K
DENND1C	2.95E-77	0.392238	0.216	0.109	9.64E-73	NK	DENND1C
DCTN3	5.39E-77	0.388016	0.398	0.266	1.76E-72	NK	DCTN3
SLC15A4	6.19E-77	0.322954	0.183	0.083	2.03E-72	NK	SLC15A4
ZNF32	8.02E-77	0.362093	0.176	0.079	2.62E-72	NK	ZNF32
TEX2641	9.43E-77	0.393525	0.3	0.177	3.09E-72	NK	TEX264
SYNC	9.83E-77	0.355075	0.159	0.069	3.22E-72	NK	SYNC
PEX2	1.43E-76	0.368702	0.194	0.093	4.69E-72	NK	PEX2
BAX2	1.68E-76	0.430417	0.507	0.365	5.51E-72	NK	BAX
VPS26B	1.84E-76	0.380251	0.249	0.134	6.04E-72	NK	VPS26B
DUSP113	2.77E-76	0.363961	0.177	0.081	9.08E-72	NK	DUSP11
SSNA11	8.23E-76	0.396174	0.409	0.275	2.69E-71	NK	SSNA1
XRN1	1.98E-75	0.393244	0.293	0.17	6.47E-71	NK	XRN1
COX6A12	2.47E-75	0.272902	0.78	0.603	8.08E-71	NK	COX6A1
METTL232	2.52E-75	0.380936	0.225	0.116	8.25E-71	NK	METTL23
HIBCH	3.25E-75	0.302225	0.126	0.048	1.07E-70	NK	HIBCH
SLC16A7	3.41E-75	0.327993	0.15	0.063	1.12E-70	NK	SLC16A7
RSBN1L	3.76E-75	0.3509	0.376	0.243	1.23E-70	NK	RSBN1L
CENPC1	4.04E-75	0.372317	0.201	0.098	1.32E-70	NK	CENPC
B3GAT3	6.03E-75	0.368539	0.22	0.113	1.97E-70	NK	B3GAT3
LRIF11	6.51E-75	0.331893	0.163	0.071	2.13E-70	NK	LRIF1
PATL21	8.56E-75	0.281698	0.114	0.041	2.80E-70	NK	PATL2
CDK13	1.23E-74	0.349195	0.221	0.113	4.02E-70	NK	CDK13
SDHC3	1.74E-74	0.403859	0.342	0.218	5.69E-70	NK	SDHC
INO80E1	2.64E-74	0.369496	0.24	0.129	8.63E-70	NK	INO80E
FIP1L1	2.68E-74	0.393821	0.253	0.139	8.78E-70	NK	FIP1L1
RBM48	3.12E-74	0.351035	0.148	0.062	1.02E-69	NK	RBM48
ATF6B	3.24E-74	0.410833	0.291	0.172	1.06E-69	NK	ATF6B
PMS1	3.80E-74	0.323839	0.138	0.056	1.24E-69	NK	PMS1
HLTF	4.21E-74	0.281039	0.109	0.038	1.38E-69	NK	HLTF
SYT11	4.86E-74	0.276621	0.121	0.045	1.59E-69	NK	SYT11
SELT	1.07E-73	0.264567	0.405	0.267	3.51E-69	NK	SELT
STK38	1.25E-73	0.364572	0.442	0.303	4.08E-69	NK	STK38
TTC13	1.33E-73	0.310135	0.13	0.051	4.36E-69	NK	TTC13
HELZ1	1.52E-73	0.357916	0.319	0.191	4.97E-69	NK	HELZ
ECHDC21	1.62E-73	0.277199	0.104	0.036	5.31E-69	NK	ECHDC2
LINC-PINT	2.09E-73	0.306772	0.144	0.06	6.83E-69	NK	LINC-PINT
SPATA13	2.45E-73	0.328642	0.194	0.093	8.01E-69	NK	SPATA13
ORMDL34	2.49E-73	0.30375	0.15	0.063	8.16E-69	NK	ORMDL3
RANGRF	2.53E-73	0.354344	0.19	0.091	8.28E-69	NK	RANGRF
PLCG21	2.56E-73	0.335501	0.186	0.088	8.37E-69	NK	PLCG2
HSP90AB1	3.19E-73	0.374465	0.709	0.543	1.04E-68	NK	HSP90AB1
GS1-251I9	6.07E-73	0.346377	0.251	0.136	1.99E-68	NK	GS1-251I9.4
SAR1A	8.18E-73	0.357547	0.232	0.122	2.68E-68	NK	SAR1A
MAP4K5	9.79E-73	0.266447	0.164	0.072	3.21E-68	NK	MAP4K5
SSBP15	1.12E-72	0.374131	0.438	0.306	3.66E-68	NK	SSBP1
MESDC21	1.13E-72	0.407118	0.35	0.224	3.70E-68	NK	MESDC2

C9orf784	1.73E-72	0.386293	0.414	0.281	5.66E-68	NK	C9orf78
TAF152	6.22E-72	0.422287	0.4	0.27	2.04E-67	NK	TAF15
ESYT13	7.70E-72	0.403516	0.206	0.105	2.52E-67	NK	ESYT1
SON	8.49E-72	0.281552	0.808	0.64	2.78E-67	NK	SON
POLR2L1	8.61E-72	0.272292	0.729	0.563	2.82E-67	NK	POLR2L
GZMK2	1.27E-71	0.361407	0.122	0.044	4.17E-67	NK	GZMK
RAB8B	1.63E-71	0.33921	0.337	0.208	5.35E-67	NK	RAB8B
CIAO1	3.67E-71	0.373904	0.269	0.154	1.20E-66	NK	CIAO1
DEXI	3.71E-71	0.259705	0.112	0.041	1.21E-66	NK	DEXI
CLASP1	4.44E-71	0.274517	0.114	0.042	1.46E-66	NK	CLASP1
C12orf233	5.38E-71	0.264188	0.105	0.037	1.76E-66	NK	C12orf23
USP47	6.17E-71	0.346599	0.244	0.133	2.02E-66	NK	USP47
ADRM11	6.98E-71	0.355543	0.357	0.232	2.28E-66	NK	ADRM1
OSTC2	7.87E-71	0.402904	0.386	0.261	2.58E-66	NK	OSTC
TIAL1	1.18E-70	0.374174	0.306	0.186	3.86E-66	NK	TIAL1
PDK3	1.23E-70	0.277211	0.151	0.066	4.03E-66	NK	PDK3
PSMC31	1.26E-70	0.375833	0.293	0.177	4.12E-66	NK	PSMC3
ARFGAP3	1.54E-70	0.382558	0.233	0.126	5.05E-66	NK	ARFGAP3
ZNF4931	1.81E-70	0.284217	0.127	0.05	5.94E-66	NK	ZNF493
TRAPPC41	3.17E-70	0.368434	0.23	0.124	1.04E-65	NK	TRAPPC4
IARS	3.84E-70	0.327252	0.145	0.062	1.26E-65	NK	IARS
UPF3A	3.98E-70	0.338593	0.24	0.13	1.30E-65	NK	UPF3A
PCED1B4	6.34E-70	0.275083	0.205	0.098	2.08E-65	NK	PCED1B
PPM1G2	6.56E-70	0.324636	0.422	0.288	2.15E-65	NK	PPM1G
OTUB12	8.71E-70	0.355353	0.445	0.315	2.85E-65	NK	OTUB1
SEMA4D	1.09E-69	0.286967	0.325	0.197	3.57E-65	NK	SEMA4D
GMCL1	1.39E-69	0.290977	0.227	0.119	4.54E-65	NK	GMCL1
MICU21	1.81E-69	0.366515	0.237	0.129	5.93E-65	NK	MICU2
UNC50	2.00E-69	0.343198	0.206	0.106	6.54E-65	NK	UNC50
PYURF1	2.09E-69	0.35111	0.418	0.291	6.85E-65	NK	PYURF
TBK1	2.30E-69	0.325192	0.163	0.074	7.54E-65	NK	TBK1
FGFR1OP2	3.04E-69	0.317986	0.349	0.223	9.94E-65	NK	FGFR1OP2
ILKAP	4.40E-69	0.37266	0.2	0.102	1.44E-64	NK	ILKAP
SAMD9L	5.39E-69	0.335471	0.239	0.128	1.77E-64	NK	SAMD9L
NUB1	5.64E-69	0.388737	0.33	0.208	1.85E-64	NK	NUB1
NDUFS81	7.95E-69	0.378457	0.417	0.291	2.60E-64	NK	NDUFS8
RNF113A1	8.65E-69	0.353292	0.201	0.102	2.83E-64	NK	RNF113A
HERPUD2[illegible]	9.34E-69	0.349324	0.21	0.109	3.06E-64	NK	HERPUD2
PPP2R5A	9.76E-69	0.33094	0.29	0.172	3.19E-64	NK	PPP2R5A
HARS1	1.00E-68	0.357981	0.185	0.091	3.29E-64	NK	HARS
OCIAD13	1.32E-68	0.356901	0.413	0.285	4.31E-64	NK	OCIAD1
ATAD11	1.71E-68	0.294736	0.139	0.059	5.59E-64	NK	ATAD1
EHMT1	2.14E-68	0.390341	0.23	0.125	7.01E-64	NK	EHMT1
C1GALT1	3.06E-68	0.324016	0.222	0.117	1.00E-63	NK	C1GALT1
COPB1	3.67E-68	0.404654	0.325	0.206	1.20E-63	NK	COPB1
LPXN2	4.15E-68	0.3944	0.298	0.181	1.36E-63	NK	LPXN
HIPK2	6.57E-68	0.362228	0.249	0.138	2.15E-63	NK	HIPK2
PITHD12	6.62E-68	0.328383	0.204	0.105	2.17E-63	NK	PITHD1
ATP5F11	1.03E-67	0.303477	0.604	0.454	3.37E-63	NK	ATP5F1
P2RY83	1.24E-67	0.369708	0.218	0.115	4.07E-63	NK	P2RY8
MRPS9	1.49E-67	0.31358	0.148	0.065	4.89E-63	NK	MRPS9
HNRNPK	1.55E-67	0.293336	0.704	0.561	5.08E-63	NK	HNRNPK
PSIP15	2.46E-67	0.369248	0.349	0.222	8.06E-63	NK	PSIP1
CASP3	2.71E-67	0.259829	0.169	0.079	8.86E-63	NK	CASP3
RECQL	2.81E-67	0.367392	0.29	0.175	9.20E-63	NK	RECQL
SF3B4	3.61E-67	0.354153	0.3	0.185	1.18E-62	NK	SF3B4
AC010642	4.85E-67	0.310145	0.151	0.067	1.59E-62	NK	AC010642.1
DIABLO	5.01E-67	0.325211	0.177	0.086	1.64E-62	NK	DIABLO

VOPP1	5.36E-67	0.304248	0.248	0.137	1.76E-62	NK	VOPP1
CCDC913	5.53E-67	0.358858	0.213	0.112	1.81E-62	NK	CCDC91
PARP13	7.76E-67	0.403736	0.323	0.205	2.54E-62	NK	PARP1
STUB1	7.85E-67	0.307087	0.421	0.291	2.57E-62	NK	STUB1
RBMS11	8.32E-67	0.291841	0.455	0.319	2.72E-62	NK	RBMS1
AL592183.	9.39E-67	0.402035	0.355	0.23	3.08E-62	NK	AL592183.1
AHSA11	1.09E-66	0.375755	0.236	0.131	3.58E-62	NK	AHSA1
ZNF708	1.99E-66	0.288399	0.12	0.047	6.50E-62	NK	ZNF708
WBP1	2.00E-66	0.317599	0.218	0.116	6.54E-62	NK	WBP1
RBM32	2.03E-66	0.347628	0.604	0.459	6.65E-62	NK	RBM3
NARF	2.26E-66	0.299402	0.218	0.115	7.38E-62	NK	NARF
MTA2	3.19E-66	0.348098	0.181	0.089	1.04E-61	NK	MTA2
TMEM181	3.84E-66	0.304476	0.122	0.049	1.26E-61	NK	TMEM181
ABI1	4.31E-66	0.33298	0.298	0.182	1.41E-61	NK	ABI1
MED27	4.94E-66	0.301965	0.139	0.06	1.62E-61	NK	MED27
LAT22	5.23E-66	0.309189	0.357	0.227	1.71E-61	NK	LAT2
SETD3	7.91E-66	0.329102	0.226	0.122	2.59E-61	NK	SETD3
VPS4B	8.51E-66	0.287862	0.404	0.275	2.79E-61	NK	VPS4B
RAD9A	1.24E-65	0.250963	0.106	0.039	4.05E-61	NK	RAD9A
PDAP11	1.43E-65	0.336416	0.319	0.2	4.69E-61	NK	PDAP1
CDKN1B5	1.86E-65	0.419966	0.3	0.186	6.09E-61	NK	CDKN1B
DDB2	2.19E-65	0.2784	0.118	0.046	7.18E-61	NK	DDB2
SDHA1	2.20E-65	0.337487	0.274	0.162	7.21E-61	NK	SDHA
CNOT6L	2.25E-65	0.372018	0.24	0.135	7.35E-61	NK	CNOT6L
APRT4	3.27E-65	0.292187	0.66	0.507	1.07E-60	NK	APRT
CCT45	3.42E-65	0.383282	0.388	0.266	1.12E-60	NK	CCT4
NUP210	3.89E-65	0.403531	0.234	0.13	1.27E-60	NK	NUP210
FLI1	3.90E-65	0.328444	0.378	0.254	1.28E-60	NK	FLI1
ZNRD11	5.34E-65	0.330198	0.298	0.183	1.75E-60	NK	ZNRD1
SNRPD31	7.02E-65	0.345286	0.435	0.312	2.30E-60	NK	SNRPD3
CXCR32	7.40E-65	0.281464	0.117	0.045	2.42E-60	NK	CXCR3
CCNDBP1:	9.72E-65	0.304989	0.367	0.244	3.18E-60	NK	CCNDBP1
U2SURP1	1.06E-64	0.397319	0.404	0.28	3.47E-60	NK	U2SURP
THAP112	1.65E-64	0.37519	0.24	0.136	5.41E-60	NK	THAP11
OGT	1.73E-64	0.362752	0.319	0.2	5.68E-60	NK	OGT
CNBP3	1.88E-64	0.284006	0.669	0.515	6.14E-60	NK	CNBP
ILF24	2.41E-64	0.372913	0.364	0.246	7.88E-60	NK	ILF2
PNISR3	2.70E-64	0.299261	0.555	0.416	8.84E-60	NK	PNISR
REST2	3.34E-64	0.381093	0.324	0.207	1.09E-59	NK	REST
POLR2B	5.21E-64	0.361251	0.272	0.163	1.71E-59	NK	POLR2B
UBTF2	5.49E-64	0.408073	0.248	0.142	1.80E-59	NK	UBTF
SUPT16H	5.98E-64	0.373706	0.285	0.174	1.96E-59	NK	SUPT16H
HMCES3	7.16E-64	0.288749	0.138	0.06	2.34E-59	NK	HMCES
FNBP42	7.86E-64	0.376848	0.342	0.224	2.57E-59	NK	FNBP4
CHD31	1.01E-63	0.365682	0.311	0.193	3.29E-59	NK	CHD3
GPKOW1	1.38E-63	0.276733	0.122	0.05	4.52E-59	NK	GPKOW
RRP1B4	2.33E-63	0.344588	0.201	0.105	7.64E-59	NK	RRP1B
TMEM230:	3.35E-63	0.343745	0.347	0.231	1.10E-58	NK	TMEM230
SDHD4	3.57E-63	0.379238	0.311	0.199	1.17E-58	NK	SDHD
ZC3HAV1:	5.02E-63	0.381162	0.31	0.195	1.64E-58	NK	ZC3HAV1
COX201	5.39E-63	0.371727	0.285	0.175	1.76E-58	NK	COX20
FXN1	5.78E-63	0.320514	0.151	0.069	1.89E-58	NK	FXN
S100PBP	6.11E-63	0.283334	0.152	0.069	2.00E-58	NK	S100PBP
DENND1B	8.99E-63	0.286872	0.135	0.058	2.94E-58	NK	DENND1B
MIF4GD	9.64E-63	0.286396	0.261	0.151	3.16E-58	NK	MIF4GD
KIAA1551:	1.04E-62	0.282735	0.434	0.297	3.39E-58	NK	KIAA1551
PWP12	1.08E-62	0.383146	0.229	0.128	3.53E-58	NK	PWP1
ARGLU1	1.15E-62	0.270254	0.53	0.393	3.76E-58	NK	ARGLU1

CUTC	1.22E-62	0.313126	0.167	0.08	3.99E-58	NK	CUTC
BCAS21	2.14E-62	0.34366	0.192	0.1	6.99E-58	NK	BCAS2
UBE2F	2.28E-62	0.259267	0.228	0.126	7.48E-58	NK	UBE2F
PPP1R351	2.72E-62	0.291946	0.175	0.086	8.91E-58	NK	PPP1R35
RPS19BP1	6.70E-62	0.263566	0.393	0.268	2.19E-57	NK	RPS19BP1
DNAJB111	8.94E-62	0.355421	0.211	0.115	2.93E-57	NK	DNAJB11
DOCK102	9.24E-62	0.417283	0.239	0.137	3.02E-57	NK	DOCK10
COMMD4	9.31E-62	0.324008	0.175	0.087	3.05E-57	NK	COMMD4
ELP6	1.08E-61	0.286647	0.138	0.061	3.53E-57	NK	ELP6
PDCD44	1.68E-61	0.361879	0.34	0.221	5.51E-57	NK	PDCD4
MMADHC	1.82E-61	0.358337	0.313	0.201	5.97E-57	NK	MMADHC
SLC25A45:	2.80E-61	0.279168	0.121	0.05	9.16E-57	NK	SLC25A45
TBRG1	3.11E-61	0.308303	0.378	0.256	1.02E-56	NK	TBRG1
PPP2R5E	4.13E-61	0.323904	0.246	0.142	1.35E-56	NK	PPP2R5E
LDHA4	4.52E-61	0.307607	0.516	0.382	1.48E-56	NK	LDHA
NUP1071	6.32E-61	0.313725	0.153	0.072	2.07E-56	NK	NUP107
MGMT	7.20E-61	0.365042	0.242	0.14	2.36E-56	NK	MGMT
SDR39U12	1.09E-60	0.345318	0.2	0.106	3.57E-56	NK	SDR39U1
GCHFR	1.37E-60	0.404763	0.215	0.117	4.49E-56	NK	GCHFR
EIF3I1	1.83E-60	0.359285	0.404	0.288	5.98E-56	NK	EIF3I
C14orf119	2.05E-60	0.355866	0.288	0.18	6.72E-56	NK	C14orf119
GPS2	2.20E-60	0.314955	0.221	0.122	7.19E-56	NK	GPS2
PPP1R71	2.36E-60	0.358695	0.306	0.197	7.74E-56	NK	PPP1R7
ESYT2	5.53E-60	0.295891	0.139	0.062	1.81E-55	NK	ESYT2
DNAJB92	6.72E-60	0.289465	0.14	0.063	2.20E-55	NK	DNAJB9
GLOD42	6.87E-60	0.36033	0.236	0.136	2.25E-55	NK	GLOD4
SRSF4	1.31E-59	0.361507	0.35	0.234	4.27E-55	NK	SRSF4
ARL2BP	1.55E-59	0.35353	0.274	0.168	5.07E-55	NK	ARL2BP
IER3IP1.11	1.73E-59	0.334557	0.267	0.163	5.66E-55	NK	IER3IP1.1
CDK62	1.76E-59	0.312462	0.134	0.059	5.78E-55	NK	CDK6
SUPV3L1	1.89E-59	0.261478	0.104	0.04	6.20E-55	NK	SUPV3L1
NRBP1	1.94E-59	0.33828	0.307	0.198	6.36E-55	NK	NRBP1
B4GALT3	2.25E-59	0.290965	0.171	0.085	7.37E-55	NK	B4GALT3
TERF1	3.41E-59	0.321021	0.182	0.094	1.12E-54	NK	TERF1
POLR2C3	3.49E-59	0.322288	0.179	0.092	1.14E-54	NK	POLR2C
VPS72	3.65E-59	0.334597	0.188	0.098	1.19E-54	NK	VPS72
RAD211	5.41E-59	0.333346	0.409	0.292	1.77E-54	NK	RAD21
ACSS1	5.61E-59	0.259999	0.122	0.052	1.84E-54	NK	ACSS1
TNFSF12	7.94E-59	0.344727	0.293	0.184	2.60E-54	NK	TNFSF12
PREX11	8.09E-59	0.326901	0.352	0.236	2.65E-54	NK	PREX1
NFKBIB	1.01E-58	0.28712	0.153	0.072	3.29E-54	NK	NFKBIB
GCC24	2.58E-58	0.37396	0.339	0.222	8.44E-54	NK	GCC2
CDC42SE2	3.10E-58	0.27284	0.338	0.222	1.02E-53	NK	CDC42SE2
UBE2Q2	3.51E-58	0.296441	0.21	0.114	1.15E-53	NK	UBE2Q2
ATRX	4.86E-58	0.306673	0.463	0.336	1.59E-53	NK	ATRX
LASP11	4.97E-58	0.30599	0.338	0.224	1.63E-53	NK	LASP1
FBXO3	6.46E-58	0.278226	0.148	0.069	2.11E-53	NK	FBXO3
AGL	7.74E-58	0.260167	0.132	0.058	2.53E-53	NK	AGL
SMARCC2	1.04E-57	0.352195	0.27	0.167	3.41E-53	NK	SMARCC2
TLE41	1.41E-57	0.311796	0.32	0.207	4.63E-53	NK	TLE4
MDH22	1.66E-57	0.282159	0.47	0.346	5.43E-53	NK	MDH2
TMUB1	1.76E-57	0.325519	0.208	0.114	5.76E-53	NK	TMUB1
MRPS121	1.77E-57	0.368165	0.276	0.173	5.78E-53	NK	MRPS12
FAM103A1	2.35E-57	0.30982	0.204	0.112	7.68E-53	NK	FAM103A1
PDZD11	3.02E-57	0.274046	0.135	0.061	9.90E-53	NK	PDZD11
DERL2	3.21E-57	0.338364	0.215	0.12	1.05E-52	NK	DERL2
ATP5C12	6.91E-57	0.277597	0.488	0.36	2.26E-52	NK	ATP5C1
MAP41	7.55E-57	0.365482	0.203	0.111	2.47E-52	NK	MAP4

STRAP	8.04E-57	0.269832	0.309	0.201	2.63E-52	NK	STRAP
SCAMP3	1.55E-56	0.347883	0.241	0.144	5.09E-52	NK	SCAMP3
SARS1	1.61E-56	0.372059	0.34	0.231	5.26E-52	NK	SARS
RBM421	1.66E-56	0.333393	0.264	0.164	5.43E-52	NK	RBM42
PDCD6	1.78E-56	0.298009	0.433	0.315	5.84E-52	NK	PDCD6
FRYL	2.11E-56	0.320853	0.279	0.173	6.91E-52	NK	FRYL
TMEM222	2.37E-56	0.253431	0.117	0.049	7.75E-52	NK	TMEM222
GRK6	3.16E-56	0.275634	0.354	0.237	1.03E-51	NK	GRK6
ERH3	3.45E-56	0.300638	0.464	0.347	1.13E-51	NK	ERH
CCM2	3.65E-56	0.272875	0.302	0.193	1.20E-51	NK	CCM2
DHX36	3.81E-56	0.341696	0.336	0.224	1.25E-51	NK	DHX36
PHF16	4.19E-56	0.27108	0.146	0.068	1.37E-51	NK	PHF1
NGLY11	4.71E-56	0.32899	0.196	0.106	1.54E-51	NK	NGLY1
PRKAG1	4.94E-56	0.322473	0.168	0.086	1.62E-51	NK	PRKAG1
ANP32E2	5.73E-56	0.347074	0.261	0.158	1.88E-51	NK	ANP32E
HNRNPM	9.24E-56	0.323312	0.464	0.343	3.03E-51	NK	HNRNPM
PEF1	9.49E-56	0.272758	0.186	0.098	3.11E-51	NK	PEF1
SETD5-AS	1.35E-55	0.318526	0.216	0.122	4.42E-51	NK	SETD5-AS1
ANKRD12	1.80E-55	0.33615	0.51	0.385	5.89E-51	NK	ANKRD12
SS18L23	2.51E-55	0.325042	0.272	0.169	8.21E-51	NK	SS18L2
MFNG1	3.33E-55	0.328236	0.268	0.167	1.09E-50	NK	MFNG
TMEM216	3.53E-55	0.281085	0.123	0.054	1.16E-50	NK	TMEM216
PSMD13	3.93E-55	0.289864	0.341	0.232	1.29E-50	NK	PSMD13
EIF62	4.47E-55	0.319892	0.337	0.228	1.46E-50	NK	EIF6
IGSF82	5.58E-55	0.258701	0.114	0.048	1.83E-50	NK	IGSF8
ZGPAT1	5.92E-55	0.308351	0.152	0.074	1.94E-50	NK	ZGPAT
MORC31	7.40E-55	0.327903	0.231	0.135	2.42E-50	NK	MORC3
ASNA1	7.45E-55	0.299923	0.271	0.17	2.44E-50	NK	ASNA1
EIF4E	7.55E-55	0.270954	0.227	0.131	2.47E-50	NK	EIF4E
HYOU1	8.10E-55	0.293199	0.124	0.054	2.65E-50	NK	HYOU1
CHD9	8.62E-55	0.29705	0.279	0.174	2.82E-50	NK	CHD9
RP3-477O	9.90E-55	0.269741	0.118	0.05	3.24E-50	NK	RP3-477O4.14
ILF3	1.07E-54	0.358587	0.374	0.263	3.50E-50	NK	ILF3
PACS1	1.46E-54	0.298597	0.209	0.116	4.78E-50	NK	PACS1
UBA24	1.91E-54	0.350143	0.254	0.155	6.26E-50	NK	UBA2
EXOC2	2.07E-54	0.259912	0.118	0.051	6.78E-50	NK	EXOC2
GOPC	2.17E-54	0.327861	0.23	0.134	7.10E-50	NK	GOPC
ARL5A1	2.34E-54	0.338894	0.324	0.218	7.67E-50	NK	ARL5A
ZFP90	2.76E-54	0.280769	0.139	0.065	9.04E-50	NK	ZFP90
PCMTD1	3.86E-54	0.27887	0.303	0.196	1.26E-49	NK	PCMTD1
FAM120B	3.94E-54	0.282432	0.162	0.081	1.29E-49	NK	FAM120B
ZNF3022	4.59E-54	0.282082	0.18	0.094	1.50E-49	NK	ZNF302
U2AF1	5.06E-54	0.251157	0.525	0.403	1.66E-49	NK	U2AF1
SLC2A4RG	6.00E-54	0.265418	0.116	0.05	1.96E-49	NK	SLC2A4RG
TESPA13	7.49E-54	0.267411	0.141	0.065	2.45E-49	NK	TESPA1
TCP1	7.59E-54	0.327805	0.278	0.178	2.48E-49	NK	TCP1
URM1	1.96E-53	0.332332	0.228	0.135	6.40E-49	NK	URM1
CTAGE5	2.39E-53	0.297315	0.191	0.104	7.83E-49	NK	CTAGE5
ASMTL	3.26E-53	0.254264	0.132	0.06	1.07E-48	NK	ASMTL
ACTR6	5.03E-53	0.265005	0.149	0.073	1.65E-48	NK	ACTR6
PNN	5.07E-53	0.347801	0.323	0.218	1.66E-48	NK	PNN
NRROS2	5.13E-53	0.351344	0.271	0.171	1.68E-48	NK	NRROS
ACAT2	5.38E-53	0.25708	0.127	0.058	1.76E-48	NK	ACAT2
ZNF224	6.08E-53	0.256765	0.121	0.053	1.99E-48	NK	ZNF224
NUDC	7.39E-53	0.340539	0.32	0.217	2.42E-48	NK	NUDC
SF3A2	7.76E-53	0.304989	0.28	0.179	2.54E-48	NK	SF3A2
FYTTD1	8.23E-53	0.327144	0.251	0.153	2.69E-48	NK	FYTTD1
MED15	9.04E-53	0.338572	0.21	0.119	2.96E-48	NK	MED15

CCNT1	9.87E-53	0.287247	0.166	0.085	3.23E-48	NK	CCNT1
RDH142	1.18E-52	0.271665	0.141	0.068	3.88E-48	NK	RDH14
DNAJC17	1.32E-52	0.27788	0.133	0.062	4.33E-48	NK	DNAJC17
NFAT5	1.34E-52	0.265778	0.174	0.09	4.40E-48	NK	NFAT5
KRI1	1.39E-52	0.298536	0.173	0.09	4.54E-48	NK	KRI1
EMC71	1.42E-52	0.333162	0.263	0.165	4.64E-48	NK	EMC7
DSCR3	1.46E-52	0.266367	0.174	0.091	4.78E-48	NK	DSCR3
FAM195B	1.87E-52	0.323602	0.21	0.121	6.12E-48	NK	FAM195B
CDK45	3.37E-52	0.330477	0.182	0.099	1.10E-47	NK	CDK4
S1PR43	3.37E-52	0.260803	0.388	0.273	1.10E-47	NK	S1PR4
RFC11	4.22E-52	0.361337	0.275	0.176	1.38E-47	NK	RFC1
ZNF2921	5.31E-52	0.343411	0.279	0.178	1.74E-47	NK	ZNF292
PRPS12	6.72E-52	0.257592	0.129	0.059	2.20E-47	NK	PRPS1
FKBP2	8.29E-52	0.346119	0.36	0.252	2.72E-47	NK	FKBP2
ANKRD36	9.67E-52	0.269138	0.117	0.051	3.17E-47	NK	ANKRD36
MECP2	1.15E-51	0.289261	0.243	0.146	3.78E-47	NK	MECP2
TMED1	1.16E-51	0.289723	0.13	0.06	3.78E-47	NK	TMED1
ASL	1.37E-51	0.263486	0.14	0.067	4.48E-47	NK	ASL
HP1BP3	2.19E-51	0.271397	0.509	0.391	7.17E-47	NK	HP1BP3
KLHDC23	2.60E-51	0.305208	0.173	0.092	8.52E-47	NK	KLHDC2
IRAK4	2.64E-51	0.279203	0.246	0.149	8.64E-47	NK	IRAK4
CRBN	2.77E-51	0.251493	0.284	0.181	9.05E-47	NK	CRBN
TMEM126I	3.58E-51	0.289242	0.182	0.099	1.17E-46	NK	TMEM126B
UFL1	3.70E-51	0.322903	0.232	0.139	1.21E-46	NK	UFL1
SLC39A7	3.97E-51	0.292661	0.148	0.073	1.30E-46	NK	SLC39A7
NABP2	6.34E-51	0.282538	0.129	0.06	2.08E-46	NK	NABP2
BRIX14	1.06E-50	0.280258	0.151	0.076	3.46E-46	NK	BRIX1
RNF4	1.06E-50	0.314276	0.211	0.123	3.48E-46	NK	RNF4
RAB22A	1.11E-50	0.307094	0.176	0.095	3.63E-46	NK	RAB22A
ACD	1.12E-50	0.256648	0.132	0.062	3.65E-46	NK	ACD
NGDN3	1.60E-50	0.328711	0.2	0.114	5.24E-46	NK	NGDN
MPC21	1.84E-50	0.262039	0.247	0.151	6.01E-46	NK	MPC2
NDUFAF44	1.94E-50	0.299994	0.158	0.081	6.33E-46	NK	NDUFAF4
FGD3	2.10E-50	0.277869	0.302	0.198	6.89E-46	NK	FGD3
PSMB2	2.35E-50	0.290144	0.38	0.273	7.68E-46	NK	PSMB2
C12orf655	2.35E-50	0.294618	0.166	0.086	7.70E-46	NK	C12orf65
INPP5K	2.55E-50	0.278196	0.178	0.096	8.34E-46	NK	INPP5K
CCT6A2	2.76E-50	0.329754	0.397	0.293	9.04E-46	NK	CCT6A
PARN	3.65E-50	0.255138	0.137	0.066	1.20E-45	NK	PARN
C7orf55-L	3.77E-50	0.322826	0.25	0.156	1.24E-45	NK	C7orf55-LUC7L2
PDCD21	3.79E-50	0.33824	0.285	0.187	1.24E-45	NK	PDCD2
ZNF4311	4.43E-50	0.267559	0.136	0.065	1.45E-45	NK	ZNF431
DDHD1	4.93E-50	0.255403	0.112	0.049	1.62E-45	NK	DDHD1
KRIT1	5.67E-50	0.312296	0.2	0.112	1.86E-45	NK	KRIT1
UGP2	6.53E-50	0.252221	0.319	0.215	2.14E-45	NK	UGP2
C6orf1	7.28E-50	0.295535	0.221	0.13	2.38E-45	NK	C6orf1
PSMA31	1.01E-49	0.320932	0.315	0.215	3.31E-45	NK	PSMA3
ERLEC1	1.06E-49	0.303983	0.27	0.172	3.46E-45	NK	ERLEC1
STARD3	1.14E-49	0.300474	0.191	0.107	3.72E-45	NK	STARD3
PCID21	1.70E-49	0.308075	0.156	0.081	5.58E-45	NK	PCID2
SH3BP11	1.90E-49	0.322009	0.302	0.202	6.22E-45	NK	SH3BP1
RP11-773I	2.17E-49	0.264314	0.16	0.083	7.10E-45	NK	RP11-773D16.1
ERAP1	2.55E-49	0.283289	0.172	0.092	8.34E-45	NK	ERAP1
UPF3B1	3.46E-49	0.29499	0.161	0.084	1.13E-44	NK	UPF3B
TIPRL	3.62E-49	0.30013	0.209	0.123	1.19E-44	NK	TIPRL
MED30	3.93E-49	0.277425	0.182	0.101	1.29E-44	NK	MED30
ZFAND2B	4.17E-49	0.273777	0.218	0.128	1.36E-44	NK	ZFAND2B
USO1	4.26E-49	0.325465	0.235	0.143	1.39E-44	NK	USO1

ATF7IP1	4.36E-49	0.289974	0.245	0.15	1.43E-44	NK	ATF7IP
SDAD12	4.80E-49	0.352045	0.238	0.147	1.57E-44	NK	SDAD1
RRAGA	8.80E-49	0.321868	0.225	0.137	2.88E-44	NK	RRAGA
ESCO1	2.13E-48	0.271021	0.154	0.079	6.97E-44	NK	ESCO1
LCP12	2.33E-48	0.250835	0.824	0.668	7.63E-44	NK	LCP1
UBE2N4	3.09E-48	0.300394	0.286	0.189	1.01E-43	NK	UBE2N
KRCC1	3.43E-48	0.306878	0.244	0.152	1.12E-43	NK	KRCC1
AP1M12	4.37E-48	0.302858	0.254	0.16	1.43E-43	NK	AP1M1
NDUFA91	4.74E-48	0.297228	0.214	0.128	1.55E-43	NK	NDUFA9
PSMC51	5.80E-48	0.299434	0.38	0.279	1.90E-43	NK	PSMC5
M6PR1	9.14E-48	0.306877	0.453	0.342	2.99E-43	NK	M6PR
HSPE16	9.75E-48	0.314402	0.347	0.242	3.19E-43	NK	HSPE1
UBXN17	1.10E-47	0.258014	0.578	0.454	3.60E-43	NK	UBXN1
SRSF22	1.16E-47	0.306641	0.372	0.27	3.79E-43	NK	SRSF2
WDR33	1.20E-47	0.305262	0.239	0.148	3.93E-43	NK	WDR33
LY95	2.05E-47	0.268998	0.131	0.062	6.70E-43	NK	LY9
DDX39A	2.05E-47	0.326563	0.243	0.152	6.73E-43	NK	DDX39A
REXO22	2.18E-47	0.268559	0.191	0.107	7.13E-43	NK	REXO2
CNPPD14	2.39E-47	0.297033	0.278	0.183	7.82E-43	NK	CNPPD1
RWDD11	2.42E-47	0.255571	0.454	0.343	7.94E-43	NK	RWDD1
AIP3	3.14E-47	0.290776	0.348	0.247	1.03E-42	NK	AIP
POP41	3.73E-47	0.294073	0.201	0.117	1.22E-42	NK	POP4
TUFM1	6.56E-47	0.310904	0.423	0.318	2.15E-42	NK	TUFM
ZNF160	8.81E-47	0.251582	0.137	0.067	2.88E-42	NK	ZNF160
TMEM106l	9.68E-47	0.268548	0.158	0.082	3.17E-42	NK	TMEM106B
SMARCA2	1.14E-46	0.278902	0.264	0.17	3.73E-42	NK	SMARCA2
HTATSF1	1.15E-46	0.329651	0.237	0.148	3.76E-42	NK	HTATSF1
NIP71	1.21E-46	0.270059	0.135	0.067	3.95E-42	NK	NIP7
CYC12	1.30E-46	0.342761	0.331	0.235	4.24E-42	NK	CYC1
ZBTB1	1.31E-46	0.284254	0.179	0.099	4.30E-42	NK	ZBTB1
MRPL22	1.53E-46	0.318425	0.187	0.107	4.99E-42	NK	MRPL22
HNRNPD	2.31E-46	0.259625	0.484	0.373	7.56E-42	NK	HNRNPD
BBC3	2.73E-46	0.255475	0.13	0.063	8.94E-42	NK	BBC3
ENOPH11	3.26E-46	0.272149	0.142	0.072	1.07E-41	NK	ENOPH1
B3GALT4	3.48E-46	0.259704	0.165	0.089	1.14E-41	NK	B3GALT4
PSMD14	3.70E-46	0.295393	0.185	0.105	1.21E-41	NK	PSMD14
PBDC11	4.64E-46	0.32633	0.198	0.116	1.52E-41	NK	PBDC1
AASDHPP'	7.01E-46	0.290834	0.225	0.138	2.30E-41	NK	AASDHPPT
B3GALT6	1.02E-45	0.250397	0.123	0.058	3.33E-41	NK	B3GALT6
GOLGA4	1.26E-45	0.336552	0.333	0.233	4.13E-41	NK	GOLGA4
SFPQ1	1.42E-45	0.297207	0.457	0.348	4.64E-41	NK	SFPQ
CCDC137	1.63E-45	0.302663	0.158	0.084	5.32E-41	NK	CCDC137
CIB13	2.00E-45	0.276126	0.467	0.359	6.55E-41	NK	CIB1
TMEM50B	2.68E-45	0.333877	0.193	0.112	8.76E-41	NK	TMEM50B
GON4L	2.70E-45	0.327173	0.222	0.136	8.83E-41	NK	GON4L
ETNK1	3.23E-45	0.298955	0.202	0.12	1.06E-40	NK	ETNK1
SF3A1	3.61E-45	0.29866	0.278	0.185	1.18E-40	NK	SF3A1
RPN2	5.22E-45	0.256655	0.428	0.324	1.71E-40	NK	RPN2
SLC52A2	6.62E-45	0.285149	0.138	0.07	2.17E-40	NK	SLC52A2
ATG5	7.41E-45	0.264395	0.181	0.102	2.43E-40	NK	ATG5
UBE3A	7.49E-45	0.314777	0.287	0.194	2.45E-40	NK	UBE3A
BCL11B4	1.40E-44	0.250053	0.13	0.063	4.59E-40	NK	BCL11B
SECISBP2L	1.41E-44	0.283943	0.213	0.128	4.60E-40	NK	SECISBP2L
ZMYM5	1.42E-44	0.261376	0.139	0.07	4.66E-40	NK	ZMYM5
SMC3	1.43E-44	0.340234	0.254	0.165	4.69E-40	NK	SMC3
GPAA1	1.62E-44	0.260431	0.243	0.154	5.30E-40	NK	GPAA1
DNM2	1.68E-44	0.28476	0.285	0.191	5.51E-40	NK	DNM2
IK	1.72E-44	0.292121	0.4	0.299	5.63E-40	NK	IK

GPS1	2.57E-44	0.30814	0.228	0.144	8.42E-40	NK	GPS1
MAF1	2.70E-44	0.29651	0.316	0.222	8.83E-40	NK	MAF1
BRD7	3.30E-44	0.287463	0.361	0.261	1.08E-39	NK	BRD7
TSSC4	3.31E-44	0.253499	0.227	0.141	1.08E-39	NK	TSSC4
ELAC2	3.33E-44	0.257978	0.122	0.059	1.09E-39	NK	ELAC2
PSMD2	3.33E-44	0.260319	0.307	0.213	1.09E-39	NK	PSMD2
ELOVL12	3.46E-44	0.317512	0.254	0.167	1.13E-39	NK	ELOVL1
PEBP16	4.03E-44	0.282539	0.345	0.239	1.32E-39	NK	PEBP1
CTCF	7.09E-44	0.284603	0.277	0.185	2.32E-39	NK	CTCF
RPS27L3	8.30E-44	0.283602	0.461	0.359	2.72E-39	NK	RPS27L
SYAP1	1.70E-43	0.305024	0.231	0.145	5.58E-39	NK	SYAP1
PDLIM21	1.76E-43	0.306948	0.278	0.187	5.75E-39	NK	PDLIM2
SNRPA12	2.15E-43	0.324174	0.204	0.123	7.05E-39	NK	SNRPA1
ALDH9A11	2.27E-43	0.277244	0.221	0.136	7.44E-39	NK	ALDH9A1
NCBP2-AS	2.44E-43	0.294211	0.227	0.142	7.97E-39	NK	NCBP2-AS2
ZNF91	2.48E-43	0.257178	0.134	0.068	8.11E-39	NK	ZNF91
PRKAA1	3.18E-43	0.25887	0.158	0.085	1.04E-38	NK	PRKAA1
LYPLA21	3.19E-43	0.274376	0.227	0.142	1.04E-38	NK	LYPLA2
ZNF721	3.78E-43	0.277241	0.157	0.085	1.24E-38	NK	ZNF721
KHDRBS13	3.81E-43	0.269383	0.494	0.391	1.25E-38	NK	KHDRBS1
CCDC82	3.97E-43	0.268778	0.174	0.098	1.30E-38	NK	CCDC82
POM121C	4.97E-43	0.264697	0.177	0.1	1.63E-38	NK	POM121C
FAF1	5.22E-43	0.259301	0.14	0.072	1.71E-38	NK	FAF1
MED4	5.51E-43	0.306386	0.261	0.173	1.80E-38	NK	MED4
POP51	6.07E-43	0.304708	0.213	0.131	1.99E-38	NK	POP5
ITFG23	6.12E-43	0.297079	0.166	0.092	2.00E-38	NK	ITFG2
ISY1	6.79E-43	0.269862	0.174	0.099	2.22E-38	NK	ISY1
PRDX25	6.82E-43	0.313896	0.314	0.217	2.23E-38	NK	PRDX2
PPID	9.26E-43	0.276293	0.188	0.11	3.03E-38	NK	PPID
PIGC1	1.01E-42	0.281072	0.182	0.106	3.30E-38	NK	PIGC
ZDHHC24	1.33E-42	0.273614	0.161	0.088	4.34E-38	NK	ZDHHC24
GADD45B1	1.68E-42	0.317541	0.208	0.127	5.49E-38	NK	GADD45B
MOGS	1.99E-42	0.26151	0.184	0.107	6.51E-38	NK	MOGS
DECR12	2.29E-42	0.279382	0.338	0.242	7.50E-38	NK	DECR1
ZNF75A1	2.75E-42	0.257261	0.122	0.059	9.00E-38	NK	ZNF75A
MRPS30	2.95E-42	0.302876	0.149	0.08	9.64E-38	NK	MRPS30
ELMO2	3.26E-42	0.253271	0.112	0.052	1.07E-37	NK	ELMO2
RBM25	4.19E-42	0.276455	0.454	0.348	1.37E-37	NK	RBM25
RANBP11	6.55E-42	0.281272	0.272	0.183	2.14E-37	NK	RANBP1
WDR37	7.44E-42	0.253583	0.147	0.079	2.44E-37	NK	WDR37
IL10RA1	8.45E-42	0.268963	0.432	0.326	2.77E-37	NK	IL10RA
PSD4	1.15E-41	0.25715	0.163	0.09	3.77E-37	NK	PSD4
CCDC124	1.19E-41	0.282885	0.272	0.185	3.89E-37	NK	CCDC124
PEX11B	1.54E-41	0.289977	0.174	0.1	5.05E-37	NK	PEX11B
NUDCD22	1.64E-41	0.299718	0.199	0.121	5.37E-37	NK	NUDCD2
SPPL2A	1.80E-41	0.287119	0.231	0.146	5.91E-37	NK	SPPL2A
PDHB	1.83E-41	0.310168	0.225	0.143	5.99E-37	NK	PDHB
RPL7L1	2.16E-41	0.289324	0.269	0.181	7.08E-37	NK	RPL7L1
RNF187	2.52E-41	0.297157	0.235	0.152	8.24E-37	NK	RNF187
CPSF6	3.20E-41	0.286543	0.195	0.117	1.05E-36	NK	CPSF6
MUM1	4.67E-41	0.25381	0.119	0.058	1.53E-36	NK	MUM1
HIAT1	5.70E-41	0.251073	0.167	0.094	1.87E-36	NK	HIAT1
ZFP141	6.15E-41	0.250347	0.11	0.052	2.01E-36	NK	ZFP14
CABIN1	6.61E-41	0.290826	0.173	0.098	2.16E-36	NK	CABIN1
NDUFB6	6.69E-41	0.283297	0.319	0.229	2.19E-36	NK	NDUFB6
MRPS262	6.72E-41	0.301274	0.21	0.131	2.20E-36	NK	MRPS26
DNAJC194	7.50E-41	0.288311	0.236	0.152	2.45E-36	NK	DNAJC19
LSM3	1.20E-40	0.263244	0.336	0.245	3.91E-36	NK	LSM3

UROS	1.23E-40	0.256788	0.182	0.107	4.02E-36	NK	UROS
ARID5B3	1.25E-40	0.290391	0.138	0.072	4.09E-36	NK	ARID5B
WDR45	1.48E-40	0.271172	0.202	0.123	4.85E-36	NK	WDR45
CHD21	1.68E-40	0.314408	0.29	0.2	5.48E-36	NK	CHD2
KPNA3	2.14E-40	0.260852	0.19	0.113	7.01E-36	NK	KPNA3
AUP1	2.46E-40	0.302194	0.354	0.264	8.05E-36	NK	AUP1
SEPHS21	2.58E-40	0.294355	0.243	0.16	8.45E-36	NK	SEPHS2
SLC20A1	2.98E-40	0.276862	0.202	0.122	9.74E-36	NK	SLC20A1
ANXA4	3.46E-40	0.295538	0.217	0.137	1.13E-35	NK	ANXA4
NELFCD1	3.92E-40	0.274656	0.175	0.102	1.28E-35	NK	NELFCD
PCM1	4.34E-40	0.289337	0.32	0.226	1.42E-35	NK	PCM1
CCDC1152	4.53E-40	0.306752	0.208	0.13	1.48E-35	NK	CCDC115
PNKP	5.24E-40	0.254136	0.206	0.126	1.72E-35	NK	PNKP
ERCC5	5.77E-40	0.272363	0.218	0.136	1.89E-35	NK	ERCC5
BOLA33	6.38E-40	0.29596	0.141	0.075	2.09E-35	NK	BOLA3
TRA2B	8.31E-40	0.266724	0.396	0.302	2.72E-35	NK	TRA2B
FAM173A	1.24E-39	0.278272	0.258	0.173	4.04E-35	NK	FAM173A
MRPS36	1.26E-39	0.250501	0.296	0.207	4.12E-35	NK	MRPS36
SS18	1.29E-39	0.250517	0.124	0.063	4.21E-35	NK	SS18
DAZAP1	1.45E-39	0.284351	0.257	0.173	4.76E-35	NK	DAZAP1
VPS514	1.56E-39	0.320258	0.29	0.203	5.10E-35	NK	VPS51
PITPNB	1.63E-39	0.254004	0.174	0.101	5.35E-35	NK	PITPNB
NASP1	3.37E-39	0.310754	0.233	0.152	1.10E-34	NK	NASP
NBN1	3.59E-39	0.269385	0.235	0.152	1.18E-34	NK	NBN
BRWD1	4.44E-39	0.279331	0.228	0.146	1.45E-34	NK	BRWD1
PIGT	4.69E-39	0.28391	0.177	0.105	1.54E-34	NK	PIGT
NDUFS21	4.99E-39	0.28735	0.331	0.244	1.63E-34	NK	NDUFS2
CWF19L24	5.95E-39	0.289587	0.208	0.129	1.95E-34	NK	CWF19L2
ZBTB8OS	7.65E-39	0.273542	0.27	0.185	2.51E-34	NK	ZBTB8OS
TRPM7	8.41E-39	0.2736	0.156	0.088	2.75E-34	NK	TRPM7
PRKX	8.46E-39	0.287843	0.191	0.115	2.77E-34	NK	PRKX
ELF2	9.54E-39	0.301078	0.282	0.195	3.12E-34	NK	ELF2
RBCK1	9.65E-39	0.279565	0.313	0.226	3.16E-34	NK	RBCK1
COPB2	9.90E-39	0.262756	0.299	0.212	3.24E-34	NK	COPB2
NPAT2	1.95E-38	0.2523	0.15	0.083	6.38E-34	NK	NPAT
STX4	2.16E-38	0.270267	0.196	0.121	7.08E-34	NK	STX4
DNTTIP2	2.80E-38	0.303957	0.251	0.168	9.18E-34	NK	DNTTIP2
NUMA1	3.43E-38	0.313997	0.226	0.145	1.12E-33	NK	NUMA1
NOL11	5.58E-38	0.272812	0.154	0.087	1.83E-33	NK	NOL11
COMMD2	5.63E-38	0.272698	0.195	0.12	1.84E-33	NK	COMMD2
C1D	5.71E-38	0.252592	0.273	0.188	1.87E-33	NK	C1D
SAP30BP	7.01E-38	0.261232	0.238	0.156	2.29E-33	NK	SAP30BP
TXNDC91	7.09E-38	0.259853	0.179	0.106	2.32E-33	NK	TXNDC9
SPRYD3	8.52E-38	0.252262	0.132	0.07	2.79E-33	NK	SPRYD3
ATRAID	1.10E-37	0.309594	0.315	0.232	3.59E-33	NK	ATRAID
STOML2	1.30E-37	0.285641	0.212	0.136	4.27E-33	NK	STOML2
DCK3	2.03E-37	0.278177	0.214	0.137	6.66E-33	NK	DCK
SRP54	3.24E-37	0.255252	0.228	0.148	1.06E-32	NK	SRP54
RNF166	3.54E-37	0.2729	0.286	0.201	1.16E-32	NK	RNF166
RSF1	4.20E-37	0.280809	0.337	0.248	1.37E-32	NK	RSF1
RBM22	4.45E-37	0.283512	0.209	0.133	1.46E-32	NK	RBM22
CLK1	4.57E-37	0.250801	0.211	0.133	1.50E-32	NK	CLK1
SLMO2	5.09E-37	0.256872	0.163	0.095	1.67E-32	NK	SLMO2
CDK11B	7.52E-37	0.277408	0.209	0.133	2.46E-32	NK	CDK11B
FUBP1	9.85E-37	0.276743	0.201	0.126	3.22E-32	NK	FUBP1
PREB	1.06E-36	0.250252	0.153	0.087	3.45E-32	NK	PREB
DNAJC3	1.21E-36	0.256915	0.296	0.21	3.97E-32	NK	DNAJC3
PFDN1	1.62E-36	0.265426	0.283	0.2	5.30E-32	NK	PFDN1

TCF12	1.95E-36	0.276269	0.161	0.093	6.38E-32	NK	TCF12
CCDC90B	1.98E-36	0.295197	0.196	0.123	6.48E-32	NK	CCDC90B
MRPS7	2.10E-36	0.264983	0.248	0.168	6.86E-32	NK	MRPS7
TSR3	2.50E-36	0.259304	0.231	0.154	8.19E-32	NK	TSR3
FOXN3	2.84E-36	0.311189	0.293	0.209	9.28E-32	NK	FOXN3
MRPL381	3.00E-36	0.281297	0.171	0.103	9.84E-32	NK	MRPL38
MAD1L12	4.12E-36	0.270863	0.187	0.115	1.35E-31	NK	MAD1L1
BBX2	4.24E-36	0.306657	0.261	0.179	1.39E-31	NK	BBX
NAA20	4.27E-36	0.303245	0.222	0.146	1.40E-31	NK	NAA20
XPA	4.28E-36	0.278914	0.23	0.152	1.40E-31	NK	XPA
APH1A2	5.23E-36	0.264076	0.334	0.25	1.71E-31	NK	APH1A
DUSP12	7.26E-36	0.260772	0.126	0.067	2.38E-31	NK	DUSP12
RPIA2	8.08E-36	0.268191	0.179	0.109	2.64E-31	NK	RPIA
PSENEN1	8.65E-36	0.291038	0.274	0.193	2.83E-31	NK	PSENEN
NGRN5	1.26E-35	0.255677	0.143	0.08	4.14E-31	NK	NGRN
SATB13	1.58E-35	0.257984	0.194	0.119	5.16E-31	NK	SATB1
MFAP1	1.65E-35	0.260318	0.154	0.089	5.40E-31	NK	MFAP1
ZBTB44	1.76E-35	0.254313	0.163	0.095	5.75E-31	NK	ZBTB44
STIP1	2.44E-35	0.289793	0.208	0.133	8.00E-31	NK	STIP1
MRPL91	3.08E-35	0.26436	0.166	0.099	1.01E-30	NK	MRPL9
ERGIC24	3.83E-35	0.270229	0.191	0.12	1.25E-30	NK	ERGIC2
YDJC	4.95E-35	0.252871	0.151	0.087	1.62E-30	NK	YDJC
SMARCC1	4.96E-35	0.292482	0.253	0.173	1.62E-30	NK	SMARCC1
PHF5A	5.46E-35	0.254242	0.224	0.148	1.79E-30	NK	PHF5A
HEXA1	9.29E-35	0.250673	0.253	0.172	3.04E-30	NK	HEXA
MRPL36	1.00E-34	0.256395	0.237	0.159	3.28E-30	NK	MRPL36
CCNH	1.14E-34	0.25186	0.176	0.107	3.73E-30	NK	CCNH
THYN13	1.30E-34	0.252015	0.227	0.151	4.25E-30	NK	THYN1
NDUFA8	1.59E-34	0.277165	0.214	0.141	5.21E-30	NK	NDUFA8
TSTA3	2.97E-34	0.253449	0.168	0.101	9.72E-30	NK	TSTA3
CEBPZ-AS	3.40E-34	0.256648	0.166	0.1	1.11E-29	NK	CEBPZ-AS1
HAX14	4.45E-34	0.272885	0.284	0.204	1.46E-29	NK	HAX1
B4GALT1	5.00E-34	0.254734	0.2	0.127	1.64E-29	NK	B4GALT1
PPWD11	5.32E-34	0.259654	0.183	0.113	1.74E-29	NK	PPWD1
BCKDHA1	1.05E-33	0.270816	0.148	0.086	3.43E-29	NK	BCKDHA
EIF3B	1.30E-33	0.25775	0.251	0.174	4.27E-29	NK	EIF3B
PHAX1	1.40E-33	0.259079	0.174	0.107	4.57E-29	NK	PHAX
NUDT51	1.57E-33	0.273118	0.219	0.146	5.14E-29	NK	NUDT5
ACTR1B	1.75E-33	0.258801	0.144	0.083	5.71E-29	NK	ACTR1B
PPHLN12	2.44E-33	0.262484	0.22	0.146	8.00E-29	NK	PPHLN1
SSB3	2.46E-33	0.262182	0.382	0.297	8.06E-29	NK	SSB
RELA	4.21E-33	0.256257	0.183	0.114	1.38E-28	NK	RELA
SUMF2	4.68E-33	0.273078	0.208	0.137	1.53E-28	NK	SUMF2
ST3GAL13	5.71E-33	0.265714	0.214	0.139	1.87E-28	NK	ST3GAL1
HINT2	5.80E-33	0.262971	0.233	0.159	1.90E-28	NK	HINT2
PHPT11	6.47E-33	0.259868	0.315	0.233	2.12E-28	NK	PHPT1
JAGN1	9.54E-33	0.288178	0.208	0.138	3.12E-28	NK	JAGN1
PRRC2B	1.30E-32	0.28864	0.247	0.17	4.27E-28	NK	PRRC2B
TBP	3.84E-32	0.252192	0.114	0.061	1.26E-27	NK	TBP
MRPS18B4	4.16E-32	0.313166	0.235	0.163	1.36E-27	NK	MRPS18B
MCRS1	6.82E-32	0.257392	0.177	0.112	2.23E-27	NK	MCRS1
CEP572	1.05E-31	0.276687	0.234	0.16	3.45E-27	NK	CEP57
FBL6	1.24E-31	0.285826	0.364	0.284	4.05E-27	NK	FBL
DBP2	1.65E-31	0.267206	0.151	0.09	5.41E-27	NK	DBP
APEH	2.29E-31	0.27973	0.198	0.13	7.51E-27	NK	APEH
ARL14EP3	2.93E-31	0.263481	0.196	0.128	9.59E-27	NK	ARL14EP
API5	7.87E-31	0.253144	0.196	0.128	2.58E-26	NK	API5
TMEM141	8.66E-31	0.252199	0.227	0.155	2.84E-26	NK	TMEM141

PSMC21	2.36E-30	0.257286	0.191	0.126	7.73E-26	NK	PSMC2
GIMAP24	2.89E-30	0.256579	0.276	0.199	9.45E-26	NK	GIMAP2
MAP4K14	3.26E-30	0.264412	0.128	0.073	1.07E-25	NK	MAP4K1
ADCY71	3.64E-30	0.274323	0.224	0.153	1.19E-25	NK	ADCY7
EIF5B1	6.65E-30	0.250441	0.361	0.281	2.18E-25	NK	EIF5B
ATIC5	1.05E-29	0.252497	0.151	0.092	3.44E-25	NK	ATIC
WBP11	1.14E-29	0.252737	0.228	0.158	3.72E-25	NK	WBP11
MRPL16	1.92E-29	0.291207	0.251	0.181	6.29E-25	NK	MRPL16
CALHM22	4.83E-29	0.303991	0.196	0.132	1.58E-24	NK	CALHM2
CPSF3L	7.76E-29	0.268619	0.199	0.134	2.54E-24	NK	CPSF3L
CACYBP4	1.40E-28	0.26817	0.286	0.215	4.59E-24	NK	CACYBP
CAMK1D3	1.51E-28	0.289835	0.179	0.116	4.94E-24	NK	CAMK1D
TMEM87A	2.44E-27	0.252011	0.193	0.129	7.98E-23	NK	TMEM87A
SAMM501	2.50E-26	0.27056	0.18	0.121	8.20E-22	NK	SAMM50
NOLC11	6.45E-26	0.253891	0.149	0.094	2.11E-21	NK	NOLC1
CCAR1	6.14E-25	0.263232	0.249	0.184	2.01E-20	NK	CCAR1
EPRS1	3.33E-23	0.269602	0.253	0.192	1.09E-18	NK	EPRS
MTRNR2L1	0.004777	0.649654	0.158	0.143	1	NK	MTRNR2L12
PTGDS1	0	4.337953	0.396	0.018	0	pDC	PTGDS
PLD43	0	4.030255	0.991	0.049	0	pDC	PLD4
IGJ1	0	3.971751	0.953	0.02	0	pDC	IGJ
ITM2C1	0	3.924569	0.997	0.031	0	pDC	ITM2C
LILRA4	0	3.81334	0.976	0.003	0	pDC	LILRA4
GZMB2	0	3.720584	0.953	0.092	0	pDC	GZMB
IRF7	0	3.566379	0.979	0.163	0	pDC	IRF7
TCF41	0	3.559891	0.988	0.06	0	pDC	TCF4
UGCG1	0	3.544727	0.988	0.068	0	pDC	UGCG
IRF82	0	3.523173	0.991	0.114	0	pDC	IRF8
CCDC502	0	3.419884	0.988	0.088	0	pDC	CCDC50
MZB11	0	3.077505	0.917	0.009	0	pDC	MZB1
RP11-38J2	0	2.910806	0.944	0.006	0	pDC	RP11-38J22.6
IL3RA1	0	2.907877	0.944	0.017	0	pDC	IL3RA
SERPINF1	0	2.88456	0.926	0.011	0	pDC	SERPINF1
PPP1R14B	0	2.850805	0.944	0.078	0	pDC	PPP1R14B
CLEC4C	0	2.805998	0.938	0.003	0	pDC	CLEC4C
FAM129C1	0	2.766011	0.914	0.015	0	pDC	FAM129C
TPM2	0	2.698088	0.852	0.007	0	pDC	TPM2
SPIB1	0	2.684334	0.929	0.014	0	pDC	SPIB
DERL3	0	2.683128	0.873	0.003	0	pDC	DERL3
HERPUD11	0	2.586242	0.991	0.27	0	pDC	HERPUD1
CLIC32	0	2.579009	0.917	0.079	0	pDC	CLIC3
SCT	0	2.53207	0.784	0.001	0	pDC	SCT
LRRC26	0	2.485036	0.822	0.001	0	pDC	LRRC26
APP	0	2.448605	0.973	0.172	0	pDC	APP
STMN15	0	2.342613	0.914	0.067	0	pDC	STMN1
BCL11A2	0	2.310238	0.882	0.04	0	pDC	BCL11A
C12orf752	0	2.276478	0.967	0.143	0	pDC	C12orf75
TCL1A1	0	2.261951	0.382	0.013	0	pDC	TCL1A
CYB561A3	0	2.243418	0.917	0.097	0	pDC	CYB561A3
BLNK1	0	2.171601	0.879	0.016	0	pDC	BLNK
TSPAN131	0	2.094442	0.867	0.021	0	pDC	TSPAN13
DNASE1L3	0	2.055291	0.769	0.003	0	pDC	DNASE1L3
LINC0099	0	1.998233	0.678	0.003	0	pDC	LINC00996
SMPD3	0	1.944535	0.734	0.005	0	pDC	SMPD3
OFD13	0	1.852166	0.852	0.103	0	pDC	OFD1
PTPRS	0	1.741259	0.731	0.001	0	pDC	PTPRS
GAPT2	0	1.712173	0.902	0.151	0	pDC	GAPT
PTCRA	0	1.704916	0.775	0.024	0	pDC	PTCRA

OPN32	0	1.690385	0.82	0.108	0	pDC	OPN3
CXCR33	0	1.672054	0.734	0.044	0	pDC	CXCR3
LILRB42	0	1.651158	0.817	0.128	0	pDC	LILRB4
RUNX21	0	1.646405	0.713	0.036	0	pDC	RUNX2
GAS61	0	1.644856	0.754	0.025	0	pDC	GAS6
PPM1K6	0	1.608885	0.828	0.108	0	pDC	PPM1K
ZFAT	0	1.582396	0.654	0.02	0	pDC	ZFAT
IDH3A1	0	1.565664	0.799	0.073	0	pDC	IDH3A
GNG72	0	1.50377	0.737	0.025	0	pDC	GNG7
C12orf451	0	1.502157	0.787	0.071	0	pDC	C12orf45
MAP1A	0	1.499774	0.719	0.025	0	pDC	MAP1A
P2RY142	0	1.4919	0.663	0.009	0	pDC	P2RY14
LAMP5	0	1.476241	0.536	0.003	0	pDC	LAMP5
CIB2	0	1.465802	0.663	0.004	0	pDC	CIB2
LGMN1	0	1.448543	0.689	0.014	0	pDC	LGMN
FCHSD21	0	1.420902	0.746	0.094	0	pDC	FCHSD2
COBLL11	0	1.41836	0.645	0.007	0	pDC	COBLL1
ERN13	0	1.41766	0.716	0.092	0	pDC	ERN1
TRAF41	0	1.384338	0.648	0.013	0	pDC	TRAF4
CD2AP	0	1.369276	0.707	0.064	0	pDC	CD2AP
ST6GALN/	0	1.346757	0.683	0.033	0	pDC	ST6GALNAC4
TNFRSF21	0	1.342247	0.648	0.002	0	pDC	TNFRSF21
MYO1E2	0	1.324602	0.627	0.043	0	pDC	MYO1E
RP11-117I	0	1.323271	0.645	0.021	0	pDC	RP11-117D22.2
AFF32	0	1.310986	0.636	0.029	0	pDC	AFF3
SLC15A41	0	1.306	0.716	0.085	0	pDC	SLC15A4
SMC61	0	1.290331	0.678	0.08	0	pDC	SMC6
NPC11	0	1.252774	0.618	0.047	0	pDC	NPC1
SEL1L31	0	1.251804	0.624	0.029	0	pDC	SEL1L3
PTMS2	0	1.214776	0.645	0.061	0	pDC	PTMS
EPHB1	0	1.214517	0.595	0.001	0	pDC	EPHB1
SCAMP5	0	1.211381	0.541	0.002	0	pDC	SCAMP5
GPR1834	0	1.205419	0.666	0.079	0	pDC	GPR183
KIAA0226L	0	1.202778	0.615	0.019	0	pDC	KIAA0226L
PHACTR12	0	1.196545	0.586	0.017	0	pDC	PHACTR1
MAPKAPK.	0	1.182646	0.698	0.085	0	pDC	MAPKAPK2
AD000671	0	1.17483	0.672	0.083	0	pDC	AD000671.6
RASD1	0	1.16641	0.408	0.001	0	pDC	RASD1
MYBL2	0	1.165803	0.435	0.002	0	pDC	MYBL2
VEGFB	0	1.152701	0.58	0.02	0	pDC	VEGFB
SLAMF72	0	1.127562	0.654	0.071	0	pDC	SLAMF7
BCL7A1	0	1.112412	0.547	0.015	0	pDC	BCL7A
SLC35F3	0	1.112374	0.518	0.003	0	pDC	SLC35F3
SNRNP251	0	1.093494	0.639	0.072	0	pDC	SNRNP25
FAM213A	0	1.09222	0.497	0.013	0	pDC	FAM213A
SCN9A	0	1.085523	0.482	0.004	0	pDC	SCN9A
TLR9	0	1.085106	0.476	0.001	0	pDC	TLR9
ADA3	0	1.076658	0.63	0.066	0	pDC	ADA
ST141	0	1.071572	0.592	0.049	0	pDC	ST14
C1orf186	0	1.063344	0.515	0.001	0	pDC	C1orf186
FCER1A1	0	1.060961	0.476	0.03	0	pDC	FCER1A
DAB2	0	1.015682	0.66	0.07	0	pDC	DAB2
DUSP51	0	1.014289	0.521	0.045	0	pDC	DUSP5
HS3ST11	0	1.012916	0.45	0.002	0	pDC	HS3ST1
PACSIN1	0	1.01111	0.521	0.001	0	pDC	PACSIN1
TMEM8B1	0	0.997937	0.506	0.021	0	pDC	TMEM8B
AHI1	0	0.987791	0.488	0.021	0	pDC	AHI1
FLNB	0	0.986201	0.479	0.023	0	pDC	FLNB

FLT31	0	0.985828	0.497	0.037	0	pDC	FLT3
AC023590	0	0.978615	0.473	0.001	0	pDC	AC023590.1
ATP13A2	0	0.978042	0.58	0.046	0	pDC	ATP13A2
TUBB61	0	0.973818	0.521	0.029	0	pDC	TUBB6
PPM1J	0	0.968038	0.497	0.002	0	pDC	PPM1J
SHD	0	0.951742	0.411	0	0	pDC	SHD
RP11-73G	0	0.95116	0.438	0	0	pDC	RP11-73G16.2
CARD113	0	0.95008	0.541	0.043	0	pDC	CARD11
KCNK17	0	0.944606	0.42	0	0	pDC	KCNK17
TBC1D41	0	0.928918	0.476	0.033	0	pDC	TBC1D4
KCTD5	0	0.899014	0.509	0.046	0	pDC	KCTD5
ZDHHC17	0	0.894027	0.544	0.048	0	pDC	ZDHHC17
SPNS32	0	0.893512	0.533	0.038	0	pDC	SPNS3
ABHD15	0	0.892084	0.47	0.021	0	pDC	ABHD15
SMIM5	0	0.890521	0.512	0.017	0	pDC	SMIM5
NOTCH4	0	0.859293	0.399	0.01	0	pDC	NOTCH4
CYP46A1	0	0.8574	0.385	0	0	pDC	CYP46A1
PNOC1	0	0.849631	0.263	0.009	0	pDC	PNOC
STRBP1	0	0.849295	0.459	0.032	0	pDC	STRBP
SLC7A5	0	0.845624	0.429	0.01	0	pDC	SLC7A5
RP11-356I	0	0.831084	0.396	0.015	0	pDC	RP11-356I2.4
CDCA7L1	0	0.829428	0.456	0.021	0	pDC	CDCA7L
SIDT11	0	0.828637	0.456	0.029	0	pDC	SIDT1
MCOLN2	0	0.809641	0.391	0.014	0	pDC	MCOLN2
NRP1	0	0.808021	0.376	0.004	0	pDC	NRP1
KMO1	0	0.801585	0.438	0.035	0	pDC	KMO
CSNK1E	0	0.800492	0.473	0.042	0	pDC	CSNK1E
AEBP1	0	0.799671	0.379	0.004	0	pDC	AEBP1
TIFAB	0	0.784905	0.405	0.006	0	pDC	TIFAB
CMKLR11	0	0.781963	0.402	0.027	0	pDC	CMKLR1
GPR1141	0	0.768256	0.405	0.017	0	pDC	GPR114
RP4-647C	0	0.754163	0.367	0.003	0	pDC	RP4-647C14.2
AP000783	0	0.749845	0.379	0.014	0	pDC	AP000783.1
PFKFB2	0	0.728677	0.42	0.019	0	pDC	PFKFB2
SERPINF21	0	0.726429	0.385	0.01	0	pDC	SERPINF2
HS3ST3B1	0	0.715853	0.358	0.011	0	pDC	HS3ST3B1
RP11-24F	0	0.705853	0.379	0.024	0	pDC	RP11-24F11.2
LEPREL1	0	0.700965	0.346	0.001	0	pDC	LEPREL1
DRD4	0	0.699908	0.228	0	0	pDC	DRD4
PMEPA1	0	0.69348	0.349	0.004	0	pDC	PMEPA1
P2RY61	0	0.692976	0.358	0.009	0	pDC	P2RY6
CUX2	0	0.688708	0.331	0	0	pDC	CUX2
ASIP	0	0.686496	0.322	0	0	pDC	ASIP
ST3GAL4	0	0.684881	0.411	0.031	0	pDC	ST3GAL4
SLC2A8	0	0.684248	0.376	0.019	0	pDC	SLC2A8
PHEX	0	0.672101	0.334	0	0	pDC	PHEX
FMNL3	0	0.654016	0.364	0.025	0	pDC	FMNL3
CYYR1	0	0.642823	0.343	0.002	0	pDC	CYYR1
TEX2	0	0.636196	0.358	0.02	0	pDC	TEX2
DACH1	0	0.629712	0.349	0.009	0	pDC	DACH1
PLXNA4	0	0.616628	0.322	0.002	0	pDC	PLXNA4
GPM6B	0	0.613062	0.299	0.001	0	pDC	GPM6B
PLVAP	0	0.612034	0.275	0.002	0	pDC	PLVAP
RP1-244F	0	0.609255	0.32	0.003	0	pDC	RP1-244F24.1
KRT5	0	0.603186	0.192	0	0	pDC	KRT5
RGS7	0	0.601486	0.296	0.001	0	pDC	RGS7
IGFBP3	0	0.601274	0.192	0.005	0	pDC	IGFBP3
TTC39A	0	0.597101	0.278	0	0	pDC	TTC39A

RP11-542I	0	0.576392	0.26	0.004	0	pDC	RP11-542M13.3
PROC	0	0.570692	0.269	0	0	pDC	PROC
LINC0086!	0	0.530455	0.213	0.002	0	pDC	LINC00865
C16orf93	0	0.524886	0.275	0.008	0	pDC	C16orf93
AP000476.	0	0.521212	0.296	0.015	0	pDC	AP000476.1
BEND6	0	0.520139	0.269	0.001	0	pDC	BEND6
SLC12A3	0	0.50454	0.246	0	0	pDC	SLC12A3
HHIP-AS1	0	0.49889	0.24	0	0	pDC	HHIP-AS1
IFNLR1	0	0.494621	0.281	0.002	0	pDC	IFNLR1
NLRP7	0	0.489258	0.234	0	0	pDC	NLRP7
NUDT17	0	0.488403	0.257	0.01	0	pDC	NUDT17
TXNDC5	0	0.488311	0.24	0.008	0	pDC	TXNDC5
GGH	0	0.48352	0.269	0.012	0	pDC	GGH
CCDC183	0	0.479417	0.219	0.001	0	pDC	CCDC183
CA8	0	0.47128	0.21	0.001	0	pDC	CA8
CHPF	0	0.46711	0.225	0.008	0	pDC	CHPF
E2F51	0	0.464738	0.243	0.008	0	pDC	E2F5
EGLN3	0	0.464705	0.302	0.014	0	pDC	EGLN3
KCNK10	0	0.454123	0.231	0	0	pDC	KCNK10
ADC	0	0.446355	0.237	0.003	0	pDC	ADC
PTK7	0	0.443756	0.213	0.003	0	pDC	PTK7
KIRREL3	0	0.438712	0.16	0	0	pDC	KIRREL3
WNT10A	0	0.430397	0.192	0.003	0	pDC	WNT10A
KCNA5	0	0.425239	0.189	0	0	pDC	KCNA5
TRIP10	0	0.420605	0.231	0.008	0	pDC	TRIP10
TNFRSF17	0	0.414989	0.21	0.002	0	pDC	TNFRSF17
MCC	0	0.414567	0.216	0.002	0	pDC	MCC
SLC7A11	0	0.41429	0.21	0.001	0	pDC	SLC7A11
LTK	0	0.405004	0.201	0.003	0	pDC	LTK
SCARA5	0	0.396201	0.148	0	0	pDC	SCARA5
ARL5C	0	0.391079	0.172	0	0	pDC	ARL5C
RP11-610I	0	0.385763	0.183	0	0	pDC	RP11-610P16.1
SH3BP4	0	0.382212	0.198	0.005	0	pDC	SH3BP4
DENND5B	0	0.3811	0.201	0.007	0	pDC	DENND5B
SIGLEC6	0	0.37781	0.151	0.002	0	pDC	SIGLEC6
CRYM-AS:	0	0.377663	0.172	0.001	0	pDC	CRYM-AS1
RP11-783I	0	0.376703	0.175	0.001	0	pDC	RP11-783K16.5
RP11-385I	0	0.375978	0.169	0.001	0	pDC	RP11-385F7.1
EPHA2	0	0.37037	0.186	0	0	pDC	EPHA2
TTC24	0	0.36885	0.18	0.003	0	pDC	TTC24
DAPK2	0	0.367977	0.216	0.008	0	pDC	DAPK2
LRRC36	0	0.362986	0.186	0.003	0	pDC	LRRC36
FAM160A:	0	0.348113	0.166	0.001	0	pDC	FAM160A1
SRPX	0	0.34605	0.151	0	0	pDC	SRPX
SLC41A2	0	0.344796	0.169	0.003	0	pDC	SLC41A2
FZD3	0	0.340963	0.183	0.004	0	pDC	FZD3
DPPA4	0	0.340063	0.178	0.001	0	pDC	DPPA4
CADM4	0	0.322105	0.166	0.001	0	pDC	CADM4
LCNL1	0	0.318393	0.133	0.002	0	pDC	LCNL1
BSPRY	0	0.312025	0.154	0.002	0	pDC	BSPRY
PMP22	0	0.306588	0.157	0.003	0	pDC	PMP22
SMIM6	0	0.304849	0.166	0	0	pDC	SMIM6
RP11-439I	0	0.298555	0.121	0	0	pDC	RP11-439L8.3
AC007880	0	0.279696	0.139	0.001	0	pDC	AC007880.1
COL24A1	0	0.279199	0.142	0.001	0	pDC	COL24A1
SMOC1	0	0.26933	0.136	0	0	pDC	SMOC1
PLEKHD1	0	0.26309	0.115	0	0	pDC	PLEKHD1
SCML2	0	0.262575	0.124	0.001	0	pDC	SCML2

CDH1	0	0.254019	0.112	0.001	0	pDC	CDH1
ZNF556	0	0.250233	0.115	0	0	pDC	ZNF556
CXXC52	#######	1.385466	0.799	0.121	#######	pDC	CXXC5
IGFLR11	#######	1.418507	0.84	0.141	#######	pDC	IGFLR1
C10orf118	#######	1.414354	0.737	0.11	#######	pDC	C10orf118
LIME15	#######	1.298068	0.704	0.096	#######	pDC	LIME1
PDXP	#######	0.551561	0.331	0.022	#######	pDC	PDXP
SLC2A1	#######	0.519382	0.317	0.02	#######	pDC	SLC2A1
VIMP	#######	2.076993	0.938	0.225	#######	pDC	VIMP
CLN8	#######	0.939852	0.53	0.057	#######	pDC	CLN8
TSPAN33	#######	0.993099	0.577	0.068	#######	pDC	TSPAN3
IL18R11	#######	0.555045	0.314	0.02	#######	pDC	IL18R1
SETBP11	#######	0.570544	0.272	0.016	#######	pDC	SETBP1
SNX91	#######	1.008332	0.612	0.077	#######	pDC	SNX9
MSRB3	#######	0.356016	0.213	0.009	#######	pDC	MSRB3
NREP1	#######	0.676191	0.45	0.043	#######	pDC	NREP
HIGD1A	#######	1.589923	0.861	0.173	#######	pDC	HIGD1A
MAGED1	#######	0.640489	0.346	0.026	#######	pDC	MAGED1
ADAM191	#######	0.794107	0.423	0.038	#######	pDC	ADAM19
Mar-91	#######	0.679081	0.382	0.031	#######	pDC	9-Mar
SLC9A7	#######	0.394778	0.189	0.008	#######	pDC	SLC9A7
CRIM1	#######	0.289832	0.148	0.004	#######	pDC	CRIM1
FUT71	#######	1.048807	0.521	0.058	#######	pDC	FUT7
GPX72	#######	0.603057	0.391	0.033	#######	pDC	GPX7
IRF42	#######	0.595353	0.346	0.026	#######	pDC	IRF4
ZNF2961	#######	0.624629	0.396	0.034	#######	pDC	ZNF296
CBFA2T3	#######	0.941668	0.547	0.065	#######	pDC	CBFA2T3
NEK8	#######	0.549427	0.26	0.015	#######	pDC	NEK8
RNASE62	#######	1.789444	0.956	0.234	#######	pDC	RNASE6
NUCB24	#######	1.53043	0.834	0.162	#######	pDC	NUCB2
SLC1A52	#######	1.150369	0.636	0.092	#######	pDC	SLC1A5
GPATCH1:	#######	0.909077	0.609	0.082	#######	pDC	GPATCH11
RPS6KA42	#######	1.352119	0.775	0.141	#######	pDC	RPS6KA4
PPP1R14A	#######	0.823927	0.349	0.028	#######	pDC	PPP1R14A
PHB4	#######	1.567507	0.855	0.187	#######	pDC	PHB
MIR155HG	#######	0.294726	0.178	0.007	#######	pDC	MIR155HG
MYL6B	#######	0.629589	0.379	0.034	#######	pDC	MYL6B
ZDHHC14	#######	0.367662	0.21	0.01	#######	pDC	ZDHHC14
WDFY42	#######	0.862349	0.476	0.054	#######	pDC	WDFY4
SNTA1	#######	0.319641	0.166	0.007	#######	pDC	SNTA1
TMEM109·	#######	1.248528	0.757	0.138	#######	pDC	TMEM109
N4BP2	#######	0.820322	0.509	0.062	#######	pDC	N4BP2
AMIGO2	#######	0.415659	0.257	0.016	#######	pDC	AMIGO2
HLA-DQA	#######	1.395715	0.893	0.186	#######	pDC	HLA-DQA1
PAFAH21	#######	0.585194	0.355	0.031	#######	pDC	PAFAH2
ALOX5AP2	#######	1.953818	0.988	0.313	#######	pDC	ALOX5AP
FAM60A2	#######	1.035978	0.68	0.112	#######	pDC	FAM60A
LHFPL2	#######	0.619123	0.388	0.037	#######	pDC	LHFPL2
RPS6KA2	#######	0.378427	0.198	0.01	#######	pDC	RPS6KA2
DSN1	#######	0.530279	0.287	0.021	#######	pDC	DSN1
SIT15	#######	1.12661	0.55	0.076	#######	pDC	SIT1
C12orf44	#######	0.990796	0.654	0.105	#######	pDC	C12orf44
ABI2	#######	0.562173	0.379	0.036	#######	pDC	ABI2
TMEM191	#######	0.72404	0.447	0.05	#######	pDC	TMEM19
CEP128	#######	0.399866	0.207	0.011	#######	pDC	CEP128
UCK21	#######	0.495306	0.317	0.026	#######	pDC	UCK2
TASP1	#######	0.575015	0.37	0.035	#######	pDC	TASP1
PDIA42	#######	1.304084	0.731	0.139	#######	pDC	PDIA4

SCRN11	#######	0.6894	0.405	0.043	#######	pDC	SCRN1
ST3GAL2	#######	0.737977	0.485	0.06	#######	pDC	ST3GAL2
POLB	#######	0.881338	0.55	0.078	#######	pDC	POLB
TATDN3	#######	0.776353	0.494	0.064	#######	pDC	TATDN3
MGST22	#######	0.949069	0.66	0.112	#######	pDC	MGST2
SIK1	#######	0.386198	0.18	0.009	#######	pDC	SIK1
ZDHHC4	#######	0.731798	0.497	0.065	#######	pDC	ZDHHC4
MYCL1	#######	0.797576	0.5	0.066	#######	pDC	MYCL
PAIP1	#######	0.910545	0.621	0.101	#######	pDC	PAIP1
LRRK11	#######	0.722532	0.476	0.061	#######	pDC	LRRK1
DAAM1	#######	0.732072	0.479	0.061	#######	pDC	DAAM1
SPCS13	#######	2.016646	0.985	0.428	#######	pDC	SPCS1
ZNF789	#######	0.401462	0.234	0.016	#######	pDC	ZNF789
HYOU11	#######	0.836193	0.45	0.056	#######	pDC	HYOU1
BTLA1	#######	0.501724	0.284	0.023	#######	pDC	BTLA
SCARB1	#######	0.459831	0.263	0.02	#######	pDC	SCARB1
MIF4GD1	#######	1.064531	0.751	0.154	#######	pDC	MIF4GD
HSP90B13	#######	2.045022	0.994	0.471	#######	pDC	HSP90B1
B4GALT11	#######	1.090159	0.678	0.127	#######	pDC	B4GALT1
SUSD1	#######	0.962527	0.58	0.093	#######	pDC	SUSD1
NGLY12	#######	0.954578	0.627	0.108	#######	pDC	NGLY1
MANF2	#######	0.96944	0.639	0.111	#######	pDC	MANF
TMIGD23	#######	0.709121	0.382	0.041	#######	pDC	TMIGD2
SLC38A17	#######	1.133036	0.663	0.118	#######	pDC	SLC38A1
CD743	#######	2.377683	1	0.775	#######	pDC	CD74
SEPT111	#######	0.693974	0.494	0.068	#######	pDC	11-Sep
DCPS	#######	0.870981	0.586	0.096	#######	pDC	DCPS
SEC61B1	#######	1.989278	0.997	0.539	#######	pDC	SEC61B
UBE2J11	#######	1.304821	0.902	0.25	#######	pDC	UBE2J1
HLA-DOA	#######	0.524484	0.331	0.033	#######	pDC	HLA-DOA
MAN2B13	#######	1.247379	0.879	0.229	#######	pDC	MAN2B1
ZDHHC21	#######	0.552156	0.355	0.037	#######	pDC	ZDHHC21
LAIR11	#######	1.054494	0.769	0.163	#######	pDC	LAIR1
PLAC82	#######	1.834173	0.979	0.392	#######	pDC	PLAC8
UNC93B12	#######	1.17311	0.778	0.176	#######	pDC	UNC93B1
CNP	#######	0.664211	0.438	0.056	#######	pDC	CNP
MAP2K6	#######	0.522885	0.343	0.035	#######	pDC	MAP2K6
EFHC1	#######	0.36038	0.243	0.018	#######	pDC	EFHC1
ALG2	#######	0.743743	0.488	0.07	#######	pDC	ALG2
REPIN11	#######	0.803464	0.518	0.079	#######	pDC	REPIN1
DCK4	#######	1.057909	0.68	0.137	#######	pDC	DCK
MDN1	#######	0.776865	0.396	0.048	#######	pDC	MDN1
STT3A	#######	0.725318	0.503	0.075	#######	pDC	STT3A
CUEDC1	#######	0.497118	0.331	0.034	#######	pDC	CUEDC1
RRBP11	#######	1.459902	0.814	0.21	#######	pDC	RRBP1
CHAF1A	#######	0.511452	0.343	0.037	#######	pDC	CHAF1A
MRPL361	#######	1.107235	0.737	0.16	#######	pDC	MRPL36
SLC39A6	#######	0.813591	0.432	0.059	#######	pDC	SLC39A6
HIP11	#######	0.610456	0.396	0.049	#######	pDC	HIP1
MMP23B1	#######	0.564868	0.228	0.017	#######	pDC	MMP23B
CDYL	#######	0.836367	0.559	0.094	#######	pDC	CDYL
CKS2	#######	0.71192	0.379	0.046	#######	pDC	CKS2
HLA-DMA	#######	1.431717	0.953	0.299	#######	pDC	HLA-DMA
ALDH5A1	#######	0.319736	0.189	0.012	#######	pDC	ALDH5A1
DHTKD1	#######	0.564292	0.373	0.044	#######	pDC	DHTKD1
SFT2D21	#######	1.17734	0.754	0.178	#######	pDC	SFT2D2
OSTC3	#######	1.336039	0.882	0.264	#######	pDC	OSTC
ARHGAP17	#######	0.836967	0.645	0.125	#######	pDC	ARHGAP17

FKBP21	#######	1.285116	0.882	0.254	#######	pDC	FKBP2
TNNI21	#######	0.856564	0.399	0.053	#######	pDC	TNNI2
EIF4A3	#######	0.966165	0.669	0.138	#######	pDC	EIF4A3
CCDC102E	#######	0.294699	0.166	0.009	#######	pDC	CCDC102B
MAP4K15	#######	0.72673	0.482	0.073	#######	pDC	MAP4K1
PPIB3	#######	1.702979	0.991	0.588	#######	pDC	PPIB
TLR7	#######	0.582704	0.388	0.049	#######	pDC	TLR7
PPAP2A	#######	0.36377	0.219	0.017	#######	pDC	PPAP2A
SPINT21	#######	0.918937	0.799	0.197	#######	pDC	SPINT2
TP53I13	#######	0.731376	0.414	0.057	#######	pDC	TP53I13
MDFIC2	#######	1.021592	0.678	0.149	#######	pDC	MDFIC
PLA2G162	#######	0.92103	0.604	0.115	#######	pDC	PLA2G16
SMARCB1	#######	0.995343	0.713	0.161	#######	pDC	SMARCB1
NIPA1	#######	0.300136	0.195	0.014	#######	pDC	NIPA1
TARBP1	#######	0.478112	0.269	0.026	#######	pDC	TARBP1
CHD91	#######	0.986408	0.751	0.176	#######	pDC	CHD9
MYB	#######	0.345785	0.157	0.009	#######	pDC	MYB
FEZ21	#######	1.022872	0.781	0.194	#######	pDC	FEZ2
GGA21	#######	0.768789	0.497	0.082	#######	pDC	GGA2
AHCY2	#######	0.773974	0.506	0.085	#######	pDC	AHCY
LMAN13	#######	1.000532	0.68	0.147	#######	pDC	LMAN1
ASPH	#######	0.718271	0.405	0.056	#######	pDC	ASPH
BIK	#######	0.280187	0.151	0.008	#######	pDC	BIK
HYI	#######	0.459056	0.284	0.029	#######	pDC	HYI
CCDC88A	#######	1.324284	0.879	0.271	#######	pDC	CCDC88A
ALCAM1	#######	0.640784	0.393	0.054	#######	pDC	ALCAM
CTSC3	#######	1.650444	0.95	0.395	#######	pDC	CTSC
STAMBPL1	#######	0.390193	0.249	0.022	#######	pDC	STAMBPL1
CYSLTR11	#######	0.727411	0.533	0.094	#######	pDC	CYSLTR1
KPTN	#######	0.334475	0.201	0.015	#######	pDC	KPTN
FABP51	#######	0.828543	0.521	0.092	#######	pDC	FABP5
HLA-DPA1	#######	1.571972	0.997	0.445	#######	pDC	HLA-DPA1
CLCN5	#######	0.600259	0.32	0.037	#######	pDC	CLCN5
NME4	#######	0.667331	0.562	0.103	#######	pDC	NME4
PLP21	#######	1.39952	0.953	0.376	#######	pDC	PLP2
NPC22	#######	1.680242	0.985	0.497	#######	pDC	NPC2
KIAA0125	#######	0.258904	0.127	0.006	#######	pDC	KIAA0125
TMED102	#######	1.207743	0.885	0.274	#######	pDC	TMED10
IVD1	#######	0.720345	0.521	0.092	#######	pDC	IVD
PARP10	#######	0.724769	0.494	0.084	#######	pDC	PARP10
FAAH	#######	0.32406	0.216	0.018	#######	pDC	FAAH
ANTXR2	#######	0.758494	0.5	0.088	#######	pDC	ANTXR2
TSPYL2	#######	0.426021	0.275	0.029	#######	pDC	TSPYL2
16-Sep	#######	0.894826	0.657	0.141	#######	pDC	1-Sep
HLTF1	#######	0.553971	0.328	0.041	#######	pDC	HLTF
TGFBI1	#######	1.126708	0.858	0.251	#######	pDC	TGFBI
HLA-DRA	#######	1.466658	1	0.493	#######	pDC	HLA-DRA
RECQL5	#######	0.400391	0.237	0.022	#######	pDC	RECQL5
KIF20B1	#######	0.593563	0.346	0.046	#######	pDC	KIF20B
CERS6	#######	0.829	0.476	0.084	#######	pDC	CERS6
DNAJB93	#######	0.717158	0.42	0.066	#######	pDC	DNAJB9
MINA	#######	0.586037	0.367	0.051	#######	pDC	MINA
FUZ	#######	0.31333	0.213	0.018	#######	pDC	FUZ
GSN1	#######	0.694075	0.784	0.202	#######	pDC	GSN
RNF52	#######	0.855776	0.642	0.147	#######	pDC	RNF5
SEC11C3	#######	0.945869	0.722	0.182	#######	pDC	SEC11C
CDK2AP2	#######	1.252337	0.852	0.277	#######	pDC	CDK2AP2
KLC4	#######	0.307312	0.18	0.014	#######	pDC	KLC4

SFMBT21	#######	0.491221	0.325	0.042	#######	pDC	SFMBT2
SCAMP4	#######	0.603127	0.42	0.067	#######	pDC	SCAMP4
UVRAG2	#######	0.89204	0.698	0.17	#######	pDC	UVRAG
PRMT7	#######	0.563201	0.337	0.045	#######	pDC	PRMT7
TRAM1	#######	1.144007	0.893	0.314	#######	pDC	TRAM1
C9orf1422	#######	1.265029	0.861	0.29	#######	pDC	C9orf142
CREB3L2	#######	0.59343	0.42	0.067	#######	pDC	CREB3L2
RASA41	#######	0.650974	0.402	0.063	#######	pDC	RASA4
HLA-DPB1	#######	1.282952	0.985	0.422	#######	pDC	HLA-DPB1
TXN1	#######	1.732171	0.923	0.449	#######	pDC	TXN
RNASET23	#######	1.288127	0.976	0.46	#######	pDC	RNASET2
EPS8L2	#######	0.409161	0.201	0.017	#######	pDC	EPS8L2
SERPING1	#######	0.3848	0.225	0.022	#######	pDC	SERPING1
ERP293	#######	1.317252	0.976	0.544	#######	pDC	ERP29
CCDC152	#######	0.394123	0.195	0.017	#######	pDC	CCDC152
MLEC1	#######	1.064164	0.781	0.236	#######	pDC	MLEC
EIF2AK41	#######	0.76642	0.568	0.122	#######	pDC	EIF2AK4
A1BG1	#######	0.822604	0.654	0.154	#######	pDC	A1BG
RAP1GDS1	#######	0.691131	0.544	0.111	#######	pDC	RAP1GDS1
ZCCHC116	#######	0.744507	0.618	0.137	#######	pDC	ZCCHC11
DDAH21	#######	0.69389	0.565	0.12	#######	pDC	DDAH2
ERCC11	#######	0.82092	0.678	0.169	#######	pDC	ERCC1
PEBP17	#######	1.044414	0.811	0.242	#######	pDC	PEBP1
SPATS2	#######	0.375686	0.228	0.023	#######	pDC	SPATS2
HMGN17	#######	1.082269	0.929	0.357	#######	pDC	HMGN1
UCP24	#######	1.42461	0.97	0.548	#######	pDC	UCP2
ARHGAP31	#######	0.474886	0.317	0.043	#######	pDC	ARHGAP31
AKAP21	#######	0.267587	0.21	0.02	#######	pDC	AKAP2
SULF22	#######	0.873926	0.719	0.193	#######	pDC	SULF2
CAT4	#######	1.054213	0.852	0.283	#######	pDC	CAT
PTPRE1	#######	1.204962	0.97	0.43	#######	pDC	PTPRE
PRKRA	#######	0.589419	0.396	0.065	#######	pDC	PRKRA
PARK7	#######	1.191332	0.959	0.512	#######	pDC	PARK7
MEF2C3	#######	1.049682	0.923	0.339	#######	pDC	MEF2C
MNAT11	#######	0.43742	0.349	0.051	#######	pDC	MNAT1
RNASEH2B	#######	0.802073	0.672	0.17	#######	pDC	RNASEH2B
PLEK1	#######	1.237079	0.982	0.503	#######	pDC	PLEK
SMIM141	#######	0.831951	0.657	0.165	#######	pDC	SMIM14
SLC12A2	#######	0.303981	0.178	0.015	#######	pDC	SLC12A2
TM7SF2	#######	0.290416	0.183	0.016	#######	pDC	TM7SF2
ERO1LB	#######	0.597158	0.391	0.064	#######	pDC	ERO1LB
ZNF589	#######	0.32533	0.213	0.021	#######	pDC	ZNF589
SVIP1	#######	0.359719	0.633	0.143	#######	pDC	SVIP
CRYBG3	#######	0.484576	0.379	0.061	#######	pDC	CRYBG3
ZFYVE26	#######	0.432771	0.293	0.039	#######	pDC	ZFYVE26
EEF2K	#######	0.365485	0.189	0.017	#######	pDC	EEF2K
RPLP06	#######	1.17534	0.982	0.777	#######	pDC	RPLP0
GMPPB	#######	0.37076	0.249	0.029	#######	pDC	GMPPB
TMEM53	#######	0.313672	0.186	0.017	#######	pDC	TMEM53
RPP25	#######	0.320683	0.198	0.019	#######	pDC	RPP25
UBE2E23	#######	0.697304	0.595	0.139	#######	pDC	UBE2E2
BLK1	#######	0.265713	0.154	0.012	#######	pDC	BLK
PPP1R16B	#######	0.344979	0.216	0.022	#######	pDC	PPP1R16B
CHD1L	#######	0.478204	0.388	0.065	#######	pDC	CHD1L
CD1641	#######	1.196535	0.914	0.367	#######	pDC	CD164
TRIP6	#######	0.278301	0.154	0.012	#######	pDC	TRIP6
C19orf102	#######	0.910755	0.769	0.233	#######	pDC	C19orf10
CD3EAP	#######	0.331291	0.189	0.018	#######	pDC	CD3EAP

ZBTB10	#######	0.273702	0.166	0.014	#######	pDC	ZBTB10
CCS	#######	0.716458	0.621	0.153	#######	pDC	CCS
THBD	#######	0.392841	0.222	0.024	#######	pDC	THBD
MFSD2A	#######	0.330069	0.219	0.023	#######	pDC	MFSD2A
C1orf109	#######	0.38714	0.237	0.027	#######	pDC	C1orf109
ZBTB33	#######	0.420524	0.287	0.039	#######	pDC	ZBTB33
BTG22	#######	0.975536	0.749	0.227	#######	pDC	BTG2
UBL71	#######	0.747675	0.645	0.167	#######	pDC	UBL7
TMED92	#######	0.962003	0.763	0.241	#######	pDC	TMED9
SPCS23	#######	1.01026	0.888	0.32	#######	pDC	SPCS2
SND1	#######	0.892199	0.68	0.191	#######	pDC	SND1
SSR4	#######	1.128152	0.976	0.593	#######	pDC	SSR4
GLO11	#######	0.739959	0.615	0.155	#######	pDC	GLO1
SLC3A2	#######	0.805574	0.651	0.172	#######	pDC	SLC3A2
CXorf212	#######	0.639942	0.453	0.092	#######	pDC	CXorf21
GNA15	#######	0.502078	0.42	0.079	#######	pDC	GNA15
TM9SF21	#######	1.015363	0.84	0.296	#######	pDC	TM9SF2
PDCD45	#######	0.873185	0.749	0.225	#######	pDC	PDCD4
NFX1	#######	0.574522	0.42	0.079	#######	pDC	NFX1
C9orf91	#######	0.371179	0.281	0.038	#######	pDC	C9orf91
NDUFV23	#######	0.990169	0.867	0.324	#######	pDC	NDUFV2
CSF2RA2	#######	0.65301	0.553	0.13	#######	pDC	CSF2RA
CHAMP1	#######	0.467563	0.331	0.052	#######	pDC	CHAMP1
SCMH1	#######	0.284946	0.163	0.014	#######	pDC	SCMH1
CD42	#######	0.9972	0.825	0.285	#######	pDC	CD4
TLR101	#######	0.297046	0.163	0.014	#######	pDC	TLR10
CCR21	#######	0.759818	0.636	0.164	#######	pDC	CCR2
ALG5	#######	0.571408	0.45	0.09	#######	pDC	ALG5
HLA-DRB1	#######	1.166775	0.985	0.51	#######	pDC	HLA-DRB1
LY97	#######	0.508229	0.376	0.064	#######	pDC	LY9
IFNAR22	#######	0.835589	0.672	0.19	#######	pDC	IFNAR2
HLA-DMB	#######	0.75497	0.781	0.236	#######	pDC	HLA-DMB
ERGIC25	#######	0.66825	0.53	0.121	#######	pDC	ERGIC2
TAGLN2	#######	0.468158	0.985	0.635	#######	pDC	TAGLN2
TMEM14C	#######	0.719267	0.669	0.183	#######	pDC	TMEM14C
WDR741	#######	0.892456	0.66	0.185	#######	pDC	WDR74
ZNRF2	#######	0.366453	0.257	0.034	#######	pDC	ZNRF2
RPN21	#######	0.99196	0.858	0.326	1.40E-99	pDC	RPN2
SLC35C2	#######	0.563623	0.456	0.094	1.81E-99	pDC	SLC35C2
FAM214A1	#######	0.552628	0.317	0.05	1.91E-99	pDC	FAM214A
QDPR	#######	0.558983	0.361	0.064	7.98E-99	pDC	QDPR
CPED1	#######	0.400392	0.251	0.033	1.63E-98	pDC	CPED1
ARL6IP6	#######	0.490511	0.423	0.082	2.53E-98	pDC	ARL6IP6
NUDT11	#######	0.623799	0.589	0.147	4.68E-98	pDC	NUDT1
SLC29A3	#######	0.362915	0.234	0.029	1.02E-97	pDC	SLC29A3
POLD21	#######	0.449285	0.373	0.067	1.98E-97	pDC	POLD2
SERTAD2	#######	0.537187	0.45	0.093	3.50E-97	pDC	SERTAD2
SGK223	#######	0.334033	0.204	0.023	3.88E-97	pDC	SGK223
APEX15	#######	0.9306	0.754	0.251	5.74E-97	pDC	APEX1
SH2D3C1	#######	0.597761	0.538	0.128	1.08E-96	pDC	SH2D3C
CD3203	#######	0.455422	0.346	0.058	1.49E-96	pDC	CD320
SCARB22	#######	0.746496	0.536	0.13	1.63E-96	pDC	SCARB2
NUP160	#######	0.417728	0.34	0.057	4.00E-96	pDC	NUP160
NUDT22	#######	0.694447	0.601	0.158	1.29E-95	pDC	NUDT22
TMEM206	1.27E-99	0.402294	0.287	0.043	4.17E-95	pDC	TMEM206
HINT21	1.41E-99	0.620713	0.612	0.16	4.62E-95	pDC	HINT2
SRPR2	3.00E-99	0.873586	0.76	0.246	9.83E-95	pDC	SRPR
KDELR21	3.81E-99	0.708632	0.719	0.217	1.25E-94	pDC	KDELR2

NUDT52	6.76E-99	0.597891	0.58	0.147	2.21E-94	pDC	NUDT5
HSD17B12	1.26E-98	0.425065	0.429	0.086	4.14E-94	pDC	HSD17B12
DNAJA11	1.27E-98	0.959806	0.879	0.361	4.17E-94	pDC	DNAJA1
CTNS	1.66E-98	0.277496	0.213	0.025	5.43E-94	pDC	CTNS
PPIA7	2.03E-98	1.000739	0.97	0.733	6.66E-94	pDC	PPIA
PHYKPL	2.47E-98	0.745361	0.68	0.198	8.07E-94	pDC	PHYKPL
SNHG74	3.04E-98	0.736506	0.701	0.208	9.95E-94	pDC	SNHG7
SSR32	3.80E-98	0.940401	0.849	0.318	1.24E-93	pDC	SSR3
MYO9A	5.55E-98	0.493844	0.302	0.048	1.82E-93	pDC	MYO9A
HINT16	8.50E-98	1.106794	0.962	0.61	2.78E-93	pDC	HINT1
DDIT45	8.77E-98	0.616383	0.473	0.102	2.87E-93	pDC	DDIT4
PVRL1	9.61E-98	0.253018	0.136	0.011	3.15E-93	pDC	PVRL1
VAMP82	9.97E-98	1.091876	0.967	0.584	3.26E-93	pDC	VAMP8
SEC61G1	1.06E-97	1.079916	0.953	0.486	3.46E-93	pDC	SEC61G
FAM96A4	1.13E-97	0.823915	0.799	0.277	3.69E-93	pDC	FAM96A
USP114	1.14E-97	0.511068	0.37	0.068	3.74E-93	pDC	USP11
SLC7A61	1.67E-97	0.515209	0.367	0.068	5.47E-93	pDC	SLC7A6
PPM1G3	7.70E-97	0.86946	0.808	0.293	2.52E-92	pDC	PPM1G
TIMM131	1.68E-96	0.754193	0.719	0.224	5.50E-92	pDC	TIMM13
HMGA11	1.96E-96	0.68187	0.553	0.14	6.42E-92	pDC	HMGA1
TMX1	2.33E-96	0.624841	0.604	0.16	7.63E-92	pDC	TMX1
PDIA63	3.78E-96	0.911251	0.793	0.272	1.24E-91	pDC	PDIA6
DHRS72	6.72E-96	0.867484	0.902	0.372	2.20E-91	pDC	DHRS7
C5orf45	1.69E-95	0.33193	0.228	0.029	5.53E-91	pDC	C5orf45
SELL4	3.44E-95	1.039145	0.973	0.46	1.13E-90	pDC	SELL
ZNF225	8.61E-95	0.634406	0.583	0.151	2.82E-90	pDC	ZNF22
USP24	1.23E-94	0.420802	0.355	0.065	4.04E-90	pDC	USP24
ITCH1	3.34E-94	0.606129	0.586	0.154	1.09E-89	pDC	ITCH
SIVA11	1.24E-93	0.86244	0.763	0.261	4.07E-89	pDC	SIVA1
CANX2	2.15E-93	1.12086	0.879	0.389	7.04E-89	pDC	CANX
NDRG1	3.41E-93	0.455252	0.382	0.074	1.12E-88	pDC	NDRG1
RP11-100(	8.24E-93	0.590202	0.583	0.153	2.70E-88	pDC	RP11-1000B6.3
TUBG1	1.05E-92	0.315616	0.204	0.025	3.42E-88	pDC	TUBG1
PDCD2L	1.39E-92	0.25519	0.169	0.017	4.55E-88	pDC	PDCD2L
EIF4ENIF1	1.40E-92	0.455873	0.287	0.046	4.60E-88	pDC	EIF4ENIF1
TMED22	3.63E-92	0.767716	0.722	0.233	1.19E-87	pDC	TMED2
HLA-DQB	4.71E-92	0.876486	0.908	0.357	1.54E-87	pDC	HLA-DQB1
ARID1B	7.13E-92	0.525856	0.479	0.111	2.34E-87	pDC	ARID1B
GALNT3	1.27E-91	0.309647	0.243	0.034	4.14E-87	pDC	GALNT3
C18orf8	1.40E-91	0.47378	0.391	0.079	4.59E-87	pDC	C18orf8
ABHD61	4.21E-91	0.382889	0.246	0.035	1.38E-86	pDC	ABHD6
OGT1	6.10E-91	0.75605	0.666	0.204	2.00E-86	pDC	OGT
PTPRCAP6	1.25E-90	0.625139	0.896	0.274	4.10E-86	pDC	PTPRCAP
PDIA32	1.27E-90	1.009132	0.914	0.418	4.15E-86	pDC	PDIA3
HNRNPA1	2.56E-90	0.384029	0.251	0.037	8.37E-86	pDC	HNRNPA1L2
EXOSC51	4.11E-90	0.530077	0.441	0.098	1.35E-85	pDC	EXOSC5
DDOST3	4.12E-90	0.82112	0.737	0.241	1.35E-85	pDC	DDOST
PBX3	4.65E-90	0.415711	0.281	0.045	1.52E-85	pDC	PBX3
CD1802	1.22E-89	0.349852	0.284	0.046	4.00E-85	pDC	CD180
DTX2	1.54E-89	0.436079	0.364	0.071	5.03E-85	pDC	DTX2
CPNE31	3.66E-89	0.677911	0.743	0.242	1.20E-84	pDC	CPNE3
ELMSAN1	5.25E-89	0.526803	0.432	0.095	1.72E-84	pDC	ELMSAN1
NHP25	7.83E-89	0.810057	0.737	0.255	2.56E-84	pDC	NHP2
GLCE	2.48E-88	0.311402	0.231	0.032	8.10E-84	pDC	GLCE
AP3S1	3.12E-88	0.636968	0.636	0.189	1.02E-83	pDC	AP3S1
NUS1	8.57E-88	0.477677	0.399	0.085	2.81E-83	pDC	NUS1
R3HDM1	9.94E-88	0.513232	0.393	0.082	3.25E-83	pDC	R3HDM1
TACC11	1.19E-87	0.774329	0.763	0.272	3.88E-83	pDC	TACC1

RALGPS21	1.71E-87	0.290327	0.287	0.047	5.59E-83	pDC	RALGPS2
CYTH42	4.03E-87	0.712475	0.713	0.232	1.32E-82	pDC	CYTH4
ANKRD12:	5.10E-87	0.960744	0.879	0.389	1.67E-82	pDC	ANKRD12
PPP1R15A	6.42E-87	0.463074	0.592	0.161	2.10E-82	pDC	PPP1R15A
IRF2BP21	7.34E-87	0.672851	0.672	0.205	2.40E-82	pDC	IRF2BP2
GNL3	8.28E-87	0.510847	0.453	0.104	2.71E-82	pDC	GNL3
CTSZ3	9.02E-87	0.956075	0.947	0.43	2.95E-82	pDC	CTSZ
TRABD	9.67E-87	0.60921	0.695	0.216	3.16E-82	pDC	TRABD
KRI11	1.55E-86	0.511081	0.423	0.093	5.07E-82	pDC	KRI1
ACN9	2.18E-86	0.476332	0.349	0.067	7.12E-82	pDC	ACN9
BICD1	5.10E-86	0.308156	0.169	0.018	1.67E-81	pDC	BICD1
SNRPN4	6.68E-86	0.475923	0.435	0.098	2.19E-81	pDC	SNRPN
DTWD1	8.69E-86	0.403399	0.334	0.063	2.84E-81	pDC	DTWD1
UCHL31	9.09E-86	0.508905	0.479	0.117	2.98E-81	pDC	UCHL3
YPEL11	9.35E-86	0.274893	0.186	0.022	3.06E-81	pDC	YPEL1
MPEG11	1.11E-85	1.027676	0.956	0.431	3.65E-81	pDC	MPEG1
NIPA21	1.35E-85	0.539467	0.441	0.102	4.43E-81	pDC	NIPA2
PON21	1.72E-85	0.2968	0.231	0.033	5.63E-81	pDC	PON2
WDFY21	4.56E-85	0.462197	0.399	0.086	1.49E-80	pDC	WDFY2
TRIT1	4.78E-85	0.346862	0.257	0.04	1.57E-80	pDC	TRIT1
ZEB1	5.41E-85	0.288051	0.189	0.023	1.77E-80	pDC	ZEB1
CEP68	1.01E-84	0.334961	0.237	0.035	3.29E-80	pDC	CEP68
BTAF1	2.23E-84	0.488711	0.379	0.08	7.30E-80	pDC	BTAF1
MLF2	2.79E-84	0.623836	0.731	0.242	9.14E-80	pDC	MLF2
XBP12	3.57E-84	0.778305	0.76	0.263	1.17E-79	pDC	XBP1
TSPAN31	5.56E-84	0.373127	0.266	0.043	1.82E-79	pDC	TSPAN31
SLC37A1	6.55E-84	0.338159	0.204	0.027	2.15E-79	pDC	SLC37A1
TRIM223	1.25E-83	0.731256	0.743	0.258	4.09E-79	pDC	TRIM22
BAIAP2	3.24E-83	0.333169	0.222	0.032	1.06E-78	pDC	BAIAP2
TUBB7	5.18E-83	0.888829	0.867	0.38	1.70E-78	pDC	TUBB
GRSF11	6.04E-83	0.718175	0.657	0.211	1.98E-78	pDC	GRSF1
SFT2D1	8.41E-83	0.644958	0.707	0.231	2.75E-78	pDC	SFT2D1
SLC39A10	9.04E-83	0.525097	0.361	0.075	2.96E-78	pDC	SLC39A10
TTC34	9.97E-83	0.815679	0.683	0.228	3.26E-78	pDC	TTC3
FCRLA1	1.05E-82	0.276705	0.127	0.011	3.44E-78	pDC	FCRLA
FAM65A	1.73E-82	0.576752	0.399	0.089	5.67E-78	pDC	FAM65A
NAPSA1	2.23E-82	0.490869	0.136	0.013	7.30E-78	pDC	NAPSA
SLC33A1	4.25E-82	0.443055	0.299	0.054	1.39E-77	pDC	SLC33A1
SOX42	7.52E-82	0.620848	0.58	0.164	2.46E-77	pDC	SOX4
TGFBR21	1.50E-81	0.801392	0.76	0.279	4.92E-77	pDC	TGFBR2
SRP14	1.58E-81	0.816254	0.985	0.738	5.18E-77	pDC	SRP14
KLHL6	1.89E-81	0.34671	0.343	0.068	6.19E-77	pDC	KLHL6
SEP152	2.54E-81	0.74129	0.769	0.283	8.32E-77	pDC	15-Sep
CCNB1IP1	3.13E-81	0.386037	0.296	0.053	1.03E-76	pDC	CCNB1IP1
C11orf241	5.48E-81	0.404441	0.275	0.047	1.79E-76	pDC	C11orf24
C1orf2282	7.95E-81	0.54093	0.391	0.086	2.60E-76	pDC	C1orf228
LL22NC03	1.06E-80	0.337961	0.219	0.031	3.47E-76	pDC	LL22NC03-2H8.5
SEC63	1.24E-80	0.67659	0.583	0.171	4.06E-76	pDC	SEC63
LYSMD21	3.09E-80	0.626589	0.648	0.202	1.01E-75	pDC	LYSMD2
ELL	1.67E-79	0.331817	0.32	0.061	5.48E-75	pDC	ELL
SCYL3	3.30E-79	0.302764	0.24	0.037	1.08E-74	pDC	SCYL3
LTV1	4.22E-79	0.437326	0.364	0.077	1.38E-74	pDC	LTV1
INPP4A1	5.49E-79	0.545654	0.547	0.15	1.80E-74	pDC	INPP4A
MORF4L2	6.71E-79	0.465572	0.414	0.096	2.20E-74	pDC	MORF4L2
PXMP2	6.91E-79	0.265837	0.189	0.025	2.26E-74	pDC	PXMP2
TP53I11	7.57E-79	0.326382	0.243	0.038	2.48E-74	pDC	TP53I11
P4HB1	1.25E-78	0.815381	0.84	0.359	4.09E-74	pDC	P4HB
ARL6IP12	3.92E-78	0.751213	0.754	0.289	1.28E-73	pDC	ARL6IP1

CCDC1073	5.40E-78	0.570867	0.598	0.174	1.77E-73	pDC		CCDC107
RPN1	6.31E-78	0.725914	0.831	0.319	2.07E-73	pDC		RPN1
SMIM191	1.83E-77	0.465333	0.485	0.123	5.98E-73	pDC		SMIM19
SYS1	1.95E-77	0.471794	0.396	0.09	6.38E-73	pDC		SYS1
COPG1	3.34E-77	0.507253	0.456	0.116	1.09E-72	pDC		COPG1
SYNC1	3.47E-77	0.40084	0.349	0.073	1.14E-72	pDC		SYNC
CBX6	4.82E-77	0.484666	0.453	0.113	1.58E-72	pDC		CBX6
RPS3A6	8.18E-77	0.748883	0.982	0.858	2.68E-72	pDC		RPS3A
BEX43	9.14E-77	0.416414	0.393	0.089	2.99E-72	pDC		BEX4
RDH143	1.28E-76	0.413099	0.343	0.071	4.20E-72	pDC		RDH14
TMEM63A	1.48E-76	0.416161	0.331	0.067	4.86E-72	pDC		TMEM63A
LDHB4	3.37E-76	0.63206	0.917	0.389	1.10E-71	pDC		LDHB
ETV62	3.75E-76	0.586627	0.666	0.222	1.23E-71	pDC		ETV6
Sep-66	3.97E-76	0.701944	0.793	0.309	1.30E-71	pDC		6-Sep
EMC72	4.96E-76	0.539139	0.574	0.168	1.62E-71	pDC		EMC7
KYNU2	1.21E-75	0.430628	0.426	0.104	3.96E-71	pDC		KYNU
RPS85	1.70E-75	0.717957	1	0.879	5.56E-71	pDC		RPS8
HADH	3.93E-75	0.372828	0.296	0.056	1.29E-70	pDC		HADH
IRF2BPL	9.54E-75	0.373647	0.302	0.058	3.12E-70	pDC		IRF2BPL
UBE2S	1.06E-74	0.456308	0.45	0.113	3.46E-70	pDC		UBE2S
SOGA1	1.12E-74	0.279031	0.198	0.028	3.68E-70	pDC		SOGA1
EIF4EBP3	1.24E-74	0.443395	0.317	0.064	4.04E-70	pDC		EIF4EBP3
CALR2	1.30E-74	0.809916	0.855	0.367	4.27E-70	pDC		CALR
BET1	1.44E-74	0.39854	0.364	0.08	4.71E-70	pDC		BET1
ABCE12	1.57E-74	0.505298	0.417	0.102	5.15E-70	pDC		ABCE1
TMEM38B	2.65E-74	0.292855	0.201	0.029	8.69E-70	pDC		TMEM38B
TTC37	3.10E-74	0.457293	0.435	0.107	1.02E-69	pDC		TTC37
ZNF4284	3.44E-74	0.552422	0.586	0.175	1.13E-69	pDC		ZNF428
BRI3BP1	4.29E-74	0.551984	0.453	0.118	1.40E-69	pDC		BRI3BP
CNDP22	4.89E-74	0.606817	0.583	0.18	1.60E-69	pDC		CNDP2
PSENEN2	5.12E-74	0.605904	0.618	0.195	1.68E-69	pDC		PSENEN
APPL12	6.74E-74	0.646822	0.657	0.221	2.21E-69	pDC		APPL1
CSF2RB1	1.15E-73	0.339491	0.408	0.097	3.77E-69	pDC		CSF2RB
STX72	1.27E-73	0.570567	0.71	0.246	4.17E-69	pDC		STX7
GTF2E2	2.03E-73	0.483805	0.444	0.114	6.64E-69	pDC		GTF2E2
SGSM31	2.15E-73	0.465368	0.343	0.074	7.05E-69	pDC		SGSM3
UTP61	2.37E-73	0.456992	0.414	0.1	7.75E-69	pDC		UTP6
G6PC3	3.20E-73	0.398454	0.337	0.072	1.05E-68	pDC		G6PC3
ZNF7061	4.70E-73	0.843443	0.808	0.352	1.54E-68	pDC		ZNF706
NUPL2	4.71E-73	0.439456	0.402	0.096	1.54E-68	pDC		NUPL2
RPS236	5.13E-73	0.766855	0.982	0.825	1.68E-68	pDC		RPS23
PIP5K1A	1.17E-72	0.332025	0.293	0.056	3.82E-68	pDC		PIP5K1A
CUX11	1.25E-72	0.650804	0.737	0.266	4.08E-68	pDC		CUX1
GLT8D11	1.79E-72	0.408661	0.314	0.063	5.85E-68	pDC		GLT8D1
TUBA1B2	3.48E-72	0.741085	0.891	0.442	1.14E-67	pDC		TUBA1B
BAG11	3.90E-72	0.594367	0.669	0.231	1.28E-67	pDC		BAG1
S100PBP1	5.26E-72	0.499894	0.337	0.072	1.72E-67	pDC		S100PBP
PSMA32	5.66E-72	0.580678	0.657	0.218	1.85E-67	pDC		PSMA3
RPS56	5.95E-72	0.807399	0.985	0.705	1.95E-67	pDC		RPS5
HLA-DRB5	6.90E-72	0.972264	0.796	0.315	2.26E-67	pDC		HLA-DRB5
ZNF775	8.24E-72	0.272192	0.192	0.027	2.70E-67	pDC		ZNF775
THUMPD2	1.16E-71	0.327081	0.287	0.055	3.79E-67	pDC		THUMPD2
CORO1C	2.27E-71	0.388144	0.506	0.143	7.43E-67	pDC		CORO1C
SSR12	8.11E-71	0.701282	0.766	0.299	2.65E-66	pDC		SSR1
RERE	9.13E-71	0.49024	0.518	0.147	2.99E-66	pDC		RERE
TM9SF1	1.01E-70	0.332169	0.263	0.048	3.32E-66	pDC		TM9SF1
KDM1A	1.02E-70	0.447908	0.379	0.089	3.33E-66	pDC		KDM1A
ADI1	1.31E-70	0.583009	0.651	0.222	4.27E-66	pDC		ADI1

WDR541	1.52E-70	0.309751	0.251	0.044	4.98E-66	pDC	WDR54
ARFGAP31	1.68E-70	0.530105	0.476	0.13	5.51E-66	pDC	ARFGAP3
CAPG2	1.97E-70	0.675153	0.899	0.373	6.46E-66	pDC	CAPG
DST1	2.77E-70	0.313437	0.225	0.037	9.07E-66	pDC	DST
ITPR2	6.43E-70	0.505655	0.476	0.131	2.10E-65	pDC	ITPR2
MTMR1	1.29E-69	0.268533	0.154	0.019	4.24E-65	pDC	MTMR1
DNAJB112	3.09E-69	0.49742	0.45	0.119	1.01E-64	pDC	DNAJB11
HSPA52	3.62E-69	0.828162	0.778	0.315	1.19E-64	pDC	HSPA5
PRMT16	3.71E-69	0.591431	0.547	0.169	1.21E-64	pDC	PRMT1
SUB13	3.71E-69	0.817543	0.944	0.552	1.21E-64	pDC	SUB1
RBL1	7.03E-69	0.272341	0.186	0.027	2.30E-64	pDC	RBL1
TFRC	1.53E-68	0.301834	0.201	0.031	5.00E-64	pDC	TFRC
COMMD6:	2.75E-68	0.844169	0.962	0.701	9.02E-64	pDC	COMMD6
LEMD3	3.47E-68	0.340927	0.305	0.062	1.14E-63	pDC	LEMD3
CNPY32	4.42E-68	0.785432	0.914	0.479	1.45E-63	pDC	CNPY3
CYFIP27	5.18E-68	0.570526	0.556	0.169	1.70E-63	pDC	CYFIP2
DNAJC71	7.30E-68	0.652706	0.719	0.278	2.39E-63	pDC	DNAJC7
CCDC57	9.37E-68	0.440087	0.411	0.105	3.07E-63	pDC	CCDC57
TGIF2	1.55E-67	0.329554	0.287	0.057	5.07E-63	pDC	TGIF2
GGNBP2	2.17E-67	0.636786	0.701	0.256	7.11E-63	pDC	GGNBP2
SELPLG2	2.62E-67	0.815818	0.849	0.416	8.59E-63	pDC	SELPLG
RASGEF1B	2.78E-67	0.282705	0.154	0.019	9.09E-63	pDC	RASGEF1B
PLAGL1	4.49E-67	0.297768	0.263	0.05	1.47E-62	pDC	PLAGL1
STK11IP	7.09E-67	0.301021	0.234	0.041	2.32E-62	pDC	STK11IP
LPXN3	1.29E-66	0.596306	0.571	0.186	4.21E-62	pDC	LPXN
TMEM243!	1.40E-66	0.477373	0.553	0.167	4.60E-62	pDC	TMEM243
GRN2	2.76E-66	0.791751	0.973	0.489	9.05E-62	pDC	GRN
VPS36	3.42E-66	0.628327	0.66	0.236	1.12E-61	pDC	VPS36
DYRK4	4.72E-66	0.387351	0.278	0.056	1.54E-61	pDC	DYRK4
CTPS1	4.80E-66	0.294702	0.163	0.022	1.57E-61	pDC	CTPS1
LRRC61	6.68E-66	0.294289	0.166	0.022	2.19E-61	pDC	LRRC61
MRPL161	6.90E-66	0.530454	0.574	0.183	2.26E-61	pDC	MRPL16
ALDH22	9.20E-66	0.519916	0.66	0.221	3.01E-61	pDC	ALDH2
ARMCX3	1.19E-65	0.333468	0.405	0.103	3.91E-61	pDC	ARMCX3
MIIP1	1.25E-65	0.432607	0.488	0.14	4.10E-61	pDC	MIIP
C5orf42	1.73E-65	0.255366	0.175	0.025	5.66E-61	pDC	C5orf42
CYBA3	1.73E-65	0.766257	0.997	0.813	5.68E-61	pDC	CYBA
RPL22L11	1.80E-65	0.659506	0.71	0.27	5.91E-61	pDC	RPL22L1
SAP30BP1	2.09E-65	0.453484	0.53	0.159	6.84E-61	pDC	SAP30BP
TCF3	2.33E-65	0.27048	0.183	0.027	7.62E-61	pDC	TCF3
ITGAE1	2.66E-65	0.434999	0.547	0.168	8.71E-61	pDC	ITGAE
NFATC2IP	2.70E-65	0.533923	0.533	0.164	8.82E-61	pDC	NFATC2IP
ARHGAP9:	2.92E-65	0.565725	0.707	0.262	9.54E-61	pDC	ARHGAP9
UNC1192	3.12E-65	0.485781	0.538	0.165	1.02E-60	pDC	UNC119
SUPT5H	3.83E-65	0.495433	0.515	0.154	1.25E-60	pDC	SUPT5H
IQGAP21	4.22E-65	0.593469	0.831	0.349	1.38E-60	pDC	IQGAP2
SLC30A5	4.58E-65	0.392537	0.358	0.085	1.50E-60	pDC	SLC30A5
C12orf576	5.04E-65	0.494093	0.808	0.301	1.65E-60	pDC	C12orf57
RRAGC	5.13E-65	0.418659	0.447	0.122	1.68E-60	pDC	RRAGC
AGPAT51	1.31E-64	0.35512	0.293	0.061	4.29E-60	pDC	AGPAT5
SPCS31	1.52E-64	0.635349	0.749	0.298	4.98E-60	pDC	SPCS3
KIAA0226	1.63E-64	0.335865	0.284	0.058	5.34E-60	pDC	KIAA0226
USF21	2.11E-64	0.481664	0.565	0.179	6.90E-60	pDC	USF2
CTA-292E	4.23E-64	0.311916	0.251	0.047	1.38E-59	pDC	CTA-292E10.6
JKAMP	4.99E-64	0.410023	0.432	0.116	1.63E-59	pDC	JKAMP
GLRX51	5.26E-64	0.393664	0.58	0.185	1.72E-59	pDC	GLRX5
FIP1L11	5.40E-64	0.477366	0.491	0.144	1.77E-59	pDC	FIP1L1
NOP56	5.54E-64	0.49578	0.473	0.137	1.82E-59	pDC	NOP56

PNN1	1.43E-63	0.556146	0.639	0.221	4.68E-59	pDC	PNN
MRPS10	1.60E-63	0.437194	0.432	0.117	5.22E-59	pDC	MRPS10
MESDC22	1.61E-63	0.558742	0.654	0.229	5.26E-59	pDC	MESDC2
ERLEC11	1.94E-63	0.468596	0.559	0.175	6.34E-59	pDC	ERLEC1
LINC0090{	2.75E-63	0.321374	0.29	0.06	9.02E-59	pDC	LINC00909
SEC131	9.17E-63	0.555458	0.636	0.222	3.00E-58	pDC	SEC13
ARHGAP5:	1.83E-62	0.385772	0.26	0.052	5.99E-58	pDC	ARHGAP5
DBNL	1.94E-62	0.507285	0.805	0.336	6.35E-58	pDC	DBNL
HSPA13	2.39E-62	0.289004	0.195	0.031	7.81E-58	pDC	HSPA13
PHC3	2.69E-62	0.401589	0.391	0.1	8.79E-58	pDC	PHC3
FYCO1	3.50E-62	0.307765	0.192	0.031	1.15E-57	pDC	FYCO1
COMT1	3.61E-62	0.531915	0.784	0.311	1.18E-57	pDC	COMT
CYB5B2	5.07E-62	0.412117	0.414	0.11	1.66E-57	pDC	CYB5B
HPS4	5.39E-62	0.312277	0.257	0.051	1.77E-57	pDC	HPS4
SYK1	6.23E-62	0.516481	0.799	0.321	2.04E-57	pDC	SYK
WBSCR22:	7.20E-62	0.470936	0.429	0.118	2.36E-57	pDC	WBSCR22
MCM51	7.84E-62	0.362228	0.34	0.081	2.57E-57	pDC	MCM5
MPZL1	9.99E-62	0.307316	0.266	0.054	3.27E-57	pDC	MPZL1
RBBP41	1.14E-61	0.614127	0.716	0.283	3.73E-57	pDC	RBBP4
ATP2B42	1.21E-61	0.51864	0.488	0.145	3.95E-57	pDC	ATP2B4
ILF25	1.25E-61	0.573358	0.675	0.251	4.10E-57	pDC	ILF2
ZNF791	1.75E-61	0.349055	0.302	0.066	5.74E-57	pDC	ZNF791
FAM43A2	1.87E-61	0.408558	0.251	0.049	6.11E-57	pDC	FAM43A
HEATR5B	1.90E-61	0.326178	0.311	0.069	6.22E-57	pDC	HEATR5B
SLC38A21	3.95E-61	0.555397	0.648	0.232	1.29E-56	pDC	SLC38A2
TMEM175	3.99E-61	0.335717	0.308	0.069	1.31E-56	pDC	TMEM175
EEF1B26	4.79E-61	0.756955	0.973	0.673	1.57E-56	pDC	EEF1B2
RMDN31	5.56E-61	0.290609	0.266	0.054	1.82E-56	pDC	RMDN3
GPAA11	6.74E-61	0.393475	0.518	0.157	2.21E-56	pDC	GPAA1
INTS6	6.86E-61	0.32938	0.302	0.067	2.24E-56	pDC	INTS6
MARS	1.02E-60	0.362593	0.391	0.102	3.35E-56	pDC	MARS
NCOA72	1.03E-60	0.335746	0.447	0.124	3.38E-56	pDC	NCOA7
CDC16	1.19E-60	0.353602	0.376	0.095	3.90E-56	pDC	CDC16
IMMP2L	1.54E-60	0.263045	0.169	0.025	5.04E-56	pDC	IMMP2L
FGD24	1.60E-60	0.479868	0.536	0.172	5.24E-56	pDC	FGD2
KATNBL1	1.71E-60	0.391299	0.37	0.094	5.59E-56	pDC	KATNBL1
C21orf2	2.09E-60	0.355649	0.278	0.059	6.84E-56	pDC	C21orf2
DNAJC31	3.39E-60	0.535688	0.609	0.213	1.11E-55	pDC	DNAJC3
TECR5	4.39E-60	0.475982	0.654	0.228	1.44E-55	pDC	TECR
SEC31A	4.70E-60	0.521343	0.592	0.202	1.54E-55	pDC	SEC31A
AKR7A2	4.88E-60	0.45108	0.536	0.172	1.60E-55	pDC	AKR7A2
PI4KA	9.82E-60	0.432458	0.396	0.107	3.22E-55	pDC	PI4KA
SLC23A2	1.04E-59	0.323814	0.234	0.044	3.41E-55	pDC	SLC23A2
PTRHD12	1.05E-59	0.524375	0.663	0.244	3.45E-55	pDC	PTRHD1
SNORD3A	1.75E-59	0.385851	0.296	0.066	5.71E-55	pDC	SNORD3A
TRDMT11	1.79E-59	0.289376	0.237	0.045	5.87E-55	pDC	TRDMT1
ESD1	1.81E-59	0.557756	0.612	0.215	5.93E-55	pDC	ESD
FAM105A:	3.56E-59	0.563063	0.515	0.168	1.16E-54	pDC	FAM105A
RPL10A6	5.45E-59	0.757517	0.964	0.688	1.78E-54	pDC	RPL10A
NOP585	5.98E-59	0.460819	0.53	0.167	1.96E-54	pDC	NOP58
USO11	9.22E-59	0.409719	0.485	0.147	3.02E-54	pDC	USO1
UBE2D26	1.07E-58	0.622682	0.87	0.421	3.50E-54	pDC	UBE2D2
HSPE17	1.22E-58	0.581111	0.663	0.246	3.99E-54	pDC	HSPE1
LARS	1.26E-58	0.461486	0.538	0.175	4.13E-54	pDC	LARS
GORASP2:	1.40E-58	0.438495	0.444	0.129	4.60E-54	pDC	GORASP2
PACS2	1.64E-58	0.315414	0.243	0.048	5.36E-54	pDC	PACS2
WDR411	1.82E-58	0.328998	0.293	0.066	5.95E-54	pDC	WDR41
RPS4X6	2.20E-58	0.649225	0.994	0.812	7.19E-54	pDC	RPS4X

PNISR4	2.76E-58	0.672395	0.876	0.422	9.03E-54	pDC	PNISR
ARMCX6	2.78E-58	0.250613	0.314	0.073	9.11E-54	pDC	ARMCX6
PFDN21	2.85E-58	0.512005	0.666	0.246	9.32E-54	pDC	PFDN2
HMGN33	3.10E-58	0.607061	0.811	0.352	1.02E-53	pDC	HMGN3
ACYP21	4.04E-58	0.445976	0.396	0.109	1.32E-53	pDC	ACYP2
PTPMT1	4.80E-58	0.399856	0.402	0.111	1.57E-53	pDC	PTPMT1
SLC25A46	5.63E-58	0.366743	0.346	0.087	1.84E-53	pDC	SLC25A46
SRSF23	6.08E-58	0.56627	0.701	0.273	1.99E-53	pDC	SRSF2
POMGNT1	7.00E-58	0.297904	0.18	0.029	2.29E-53	pDC	POMGNT1
KRTCAP22	7.49E-58	0.630737	0.929	0.495	2.45E-53	pDC	KRTCAP2
BIN16	1.10E-57	0.435075	0.506	0.153	3.61E-53	pDC	BIN1
NIN1	1.71E-57	0.493277	0.698	0.271	5.61E-53	pDC	NIN
TFPT	1.84E-57	0.332414	0.29	0.065	6.03E-53	pDC	TFPT
CTC-228N	2.65E-57	0.283165	0.216	0.04	8.66E-53	pDC	CTC-228N24.3
C1QBP6	2.80E-57	0.62745	0.701	0.287	9.18E-53	pDC	C1QBP
MYCBP	2.94E-57	0.406224	0.536	0.173	9.63E-53	pDC	MYCBP
SENP6	3.46E-57	0.570454	0.636	0.235	1.13E-52	pDC	SENP6
KIAA0100	6.93E-57	0.411487	0.473	0.146	2.27E-52	pDC	KIAA0100
ARF4	7.11E-57	0.390064	0.541	0.18	2.33E-52	pDC	ARF4
NPM17	7.71E-57	0.706169	0.944	0.524	2.52E-52	pDC	NPM1
MGAT4A5	8.02E-57	0.380484	0.411	0.113	2.63E-52	pDC	MGAT4A
COPZ11	8.18E-57	0.504222	0.618	0.223	2.68E-52	pDC	COPZ1
RHOF4	9.22E-57	0.406302	0.518	0.162	3.02E-52	pDC	RHOF
TMEM258	1.06E-56	0.715388	0.935	0.543	3.46E-52	pDC	TMEM258
GLS4	1.61E-56	0.454434	0.476	0.146	5.28E-52	pDC	GLS
SRM4	1.69E-56	0.471973	0.553	0.185	5.55E-52	pDC	SRM
MED301	1.70E-56	0.362675	0.385	0.104	5.55E-52	pDC	MED30
NDUFA92	1.78E-56	0.470604	0.438	0.131	5.84E-52	pDC	NDUFA9
CTSB1	1.88E-56	0.643172	0.893	0.43	6.17E-52	pDC	CTSB
DPP7	2.23E-56	0.523676	0.707	0.274	7.31E-52	pDC	DPP7
IAH11	2.85E-56	0.431069	0.503	0.159	9.34E-52	pDC	IAH1
APBB1IP2	3.14E-56	0.687137	0.867	0.431	1.03E-51	pDC	APBB1IP
NIPSNAP3	3.33E-56	0.362615	0.355	0.093	1.09E-51	pDC	NIPSNAP3A
TMEM208	3.84E-56	0.442525	0.506	0.163	1.26E-51	pDC	TMEM208
ACADVL	4.14E-56	0.486261	0.612	0.22	1.35E-51	pDC	ACADVL
ACSL3	4.77E-56	0.423087	0.334	0.084	1.56E-51	pDC	ACSL3
XPNPEP1	8.56E-56	0.272005	0.352	0.091	2.80E-51	pDC	XPNPEP1
TMED43	1.47E-55	0.419266	0.55	0.181	4.80E-51	pDC	TMED4
Sep-93	1.58E-55	0.722134	0.831	0.395	5.18E-51	pDC	9-Sep
FAM49B2	2.18E-55	0.586408	0.843	0.387	7.14E-51	pDC	FAM49B
HDAC31	2.27E-55	0.349355	0.42	0.121	7.44E-51	pDC	HDAC3
PCNXL41	2.30E-55	0.259514	0.234	0.046	7.54E-51	pDC	PCNXL4
NENF	2.31E-55	0.403776	0.648	0.242	7.57E-51	pDC	NENF
LIMD11	2.93E-55	0.308829	0.24	0.049	9.60E-51	pDC	LIMD1
ATG4C	3.25E-55	0.322912	0.281	0.064	1.06E-50	pDC	ATG4C
RPL320	3.92E-55	0.673922	0.976	0.746	1.28E-50	pDC	RPL3
SLC25A52	4.08E-55	0.7414	0.923	0.506	1.34E-50	pDC	SLC25A5
NDUFC2	4.51E-55	0.44151	0.612	0.222	1.48E-50	pDC	NDUFC2
MICAL11	4.55E-55	0.355735	0.414	0.119	1.49E-50	pDC	MICAL1
MAN1A1	5.24E-55	0.302061	0.275	0.061	1.72E-50	pDC	MAN1A1
ZMPSTE24	5.85E-55	0.342096	0.343	0.088	1.92E-50	pDC	ZMPSTE24
HDAC21	7.47E-55	0.448893	0.536	0.179	2.44E-50	pDC	HDAC2
CIITA2	7.89E-55	0.34381	0.34	0.087	2.58E-50	pDC	CIITA
STX18	1.18E-54	0.40095	0.251	0.054	3.87E-50	pDC	STX18
CCT77	1.32E-54	0.540689	0.621	0.234	4.32E-50	pDC	CCT7
UFL11	1.49E-54	0.477235	0.459	0.143	4.88E-50	pDC	UFL1
CHMP5	1.53E-54	0.377908	0.553	0.185	5.03E-50	pDC	CHMP5
MGME1	1.78E-54	0.263887	0.281	0.064	5.81E-50	pDC	MGME1

UBA5	1.85E-54	0.286876	0.251	0.053	6.07E-50	pDC	UBA5
ARL4C4	2.18E-54	0.359423	0.571	0.189	7.15E-50	pDC	ARL4C
ABCD4	2.27E-54	0.296084	0.249	0.052	7.44E-50	pDC	ABCD4
UBE2E3	2.27E-54	0.395794	0.589	0.211	7.45E-50	pDC	UBE2E3
TBCC4	2.68E-54	0.365011	0.388	0.107	8.76E-50	pDC	TBCC
GTF3A4	2.87E-54	0.586481	0.784	0.346	9.38E-50	pDC	GTF3A
LETM1	7.58E-54	0.293963	0.225	0.044	2.48E-49	pDC	LETM1
RP11-312(	9.43E-54	0.354216	0.379	0.105	3.09E-49	pDC	RP11-312O7.2
HCCS	9.50E-54	0.317583	0.287	0.068	3.11E-49	pDC	HCCS
LSM54	1.36E-53	0.497746	0.586	0.211	4.46E-49	pDC	LSM5
HNRNPL4	2.08E-53	0.535426	0.731	0.304	6.82E-49	pDC	HNRNPL
LTB5	2.23E-53	0.423201	0.722	0.282	7.29E-49	pDC	LTB
CMTM3	2.26E-53	0.484737	0.624	0.234	7.39E-49	pDC	CMTM3
DNAJC41	2.29E-53	0.427985	0.63	0.233	7.50E-49	pDC	DNAJC4
MEF2D	2.66E-53	0.308028	0.278	0.064	8.72E-49	pDC	MEF2D
RPSA7	2.73E-53	0.666055	0.973	0.699	8.93E-49	pDC	RPSA
ARMC10	2.84E-53	0.435633	0.411	0.121	9.30E-49	pDC	ARMC10
CCT27	3.60E-53	0.534585	0.574	0.208	1.18E-48	pDC	CCT2
KIAA1586	3.66E-53	0.263818	0.249	0.053	1.20E-48	pDC	KIAA1586
TMEM14B	3.75E-53	0.500267	0.657	0.253	1.23E-48	pDC	TMEM14B
PAN3	4.61E-53	0.330131	0.334	0.086	1.51E-48	pDC	PAN3
CDC1234	8.46E-53	0.410708	0.485	0.154	2.77E-48	pDC	CDC123
MRPL171	9.47E-53	0.426236	0.37	0.103	3.10E-48	pDC	MRPL17
PTBP31	9.47E-53	0.571629	0.689	0.28	3.10E-48	pDC	PTBP3
NSMCE4A	1.32E-52	0.317736	0.337	0.087	4.32E-48	pDC	NSMCE4A
MYL12A3	1.35E-52	0.467763	0.982	0.76	4.42E-48	pDC	MYL12A
COMMD3	1.64E-52	0.298311	0.367	0.1	5.37E-48	pDC	COMMD3
MAGOHB	1.77E-52	0.25286	0.266	0.059	5.79E-48	pDC	MAGOHB
NTAN1	1.78E-52	0.29002	0.331	0.085	5.82E-48	pDC	NTAN1
SH3TC12	1.78E-52	0.29548	0.358	0.097	5.82E-48	pDC	SH3TC1
OAS12	1.90E-52	0.425791	0.536	0.182	6.22E-48	pDC	OAS1
ATIC6	1.93E-52	0.330064	0.352	0.094	6.33E-48	pDC	ATIC
COPB11	2.33E-52	0.425124	0.595	0.211	7.63E-48	pDC	COPB1
ZDHHC24:	2.67E-52	0.346886	0.343	0.091	8.74E-48	pDC	ZDHHC24
HSP90AB1	3.12E-52	0.739922	0.956	0.551	1.02E-47	pDC	HSP90AB1
SMU1	4.06E-52	0.412851	0.473	0.151	1.33E-47	pDC	SMU1
NAPA	5.57E-52	0.433301	0.74	0.303	1.82E-47	pDC	NAPA
KTN11	6.69E-52	0.626878	0.846	0.423	2.19E-47	pDC	KTN1
FNBP43	8.09E-52	0.499094	0.612	0.229	2.65E-47	pDC	FNBP4
DDX27	1.45E-51	0.383166	0.536	0.183	4.75E-47	pDC	DDX27
FKBP3	1.47E-51	0.406292	0.435	0.135	4.80E-47	pDC	FKBP3
CCT37	1.52E-51	0.48362	0.645	0.243	4.96E-47	pDC	CCT3
C11orf582	1.96E-51	0.597621	0.873	0.459	6.40E-47	pDC	C11orf58
PPP1R24	2.66E-51	0.482149	0.686	0.273	8.71E-47	pDC	PPP1R2
C17orf89	3.46E-51	0.387689	0.547	0.189	1.13E-46	pDC	C17orf89
COA4	4.23E-51	0.391134	0.476	0.154	1.38E-46	pDC	COA4
SDR39U13	5.34E-51	0.369695	0.385	0.11	1.75E-46	pDC	SDR39U1
CHCHD23	6.12E-51	0.652395	0.938	0.622	2.00E-46	pDC	CHCHD2
TMEM179I	6.98E-51	0.468148	0.683	0.272	2.29E-46	pDC	TMEM179B
PRKCB1	7.23E-51	0.453198	0.852	0.4	2.37E-46	pDC	PRKCB
ECHDC1	8.29E-51	0.43689	0.538	0.19	2.72E-46	pDC	ECHDC1
ATP8A12	9.04E-51	0.310067	0.251	0.055	2.96E-46	pDC	ATP8A1
BHLHE401	9.66E-51	0.25145	0.24	0.051	3.16E-46	pDC	BHLHE40
ZRANB24	1.02E-50	0.470127	0.686	0.277	3.35E-46	pDC	ZRANB2
MPG	1.06E-50	0.461652	0.568	0.207	3.46E-46	pDC	MPG
SCCPDH	1.10E-50	0.281883	0.257	0.058	3.60E-46	pDC	SCCPDH
UBXN41	1.12E-50	0.517293	0.84	0.377	3.66E-46	pDC	UBXN4
TMED3	1.75E-50	0.322425	0.411	0.122	5.74E-46	pDC	TMED3

MAP3K21	2.22E-50	0.392089	0.601	0.22	7.28E-46	pDC	MAP3K2
YPEL5	2.55E-50	0.270512	0.592	0.215	8.34E-46	pDC	YPEL5
CCDC47	2.57E-50	0.433134	0.479	0.157	8.43E-46	pDC	CCDC47
SUMF21	2.98E-50	0.422665	0.441	0.14	9.75E-46	pDC	SUMF2
RPS106	3.09E-50	0.680181	0.956	0.764	1.01E-45	pDC	RPS10
MGAT2	3.96E-50	0.327087	0.334	0.09	1.30E-45	pDC	MGAT2
YLPM1	4.06E-50	0.38553	0.355	0.098	1.33E-45	pDC	YLPM1
STOML21	5.33E-50	0.369113	0.441	0.138	1.74E-45	pDC	STOML2
RINL3	5.68E-50	0.266765	0.275	0.064	1.86E-45	pDC	RINL
ATP5B3	5.78E-50	0.62717	0.899	0.488	1.89E-45	pDC	ATP5B
PIGF	5.91E-50	0.29826	0.317	0.082	1.93E-45	pDC	PIGF
OCIAD26	6.73E-50	0.363943	0.379	0.105	2.20E-45	pDC	OCIAD2
HHEX3	6.94E-50	0.290775	0.464	0.148	2.27E-45	pDC	HHEX
ACADM1	7.25E-50	0.387132	0.355	0.099	2.37E-45	pDC	ACADM
SRD5A31	7.34E-50	0.367225	0.246	0.055	2.40E-45	pDC	SRD5A3
GLIPR12	7.68E-50	0.615038	0.888	0.443	2.51E-45	pDC	GLIPR1
XXYLT1	8.68E-50	0.282179	0.207	0.041	2.84E-45	pDC	XXYLT1
DNM21	1.57E-49	0.422232	0.547	0.195	5.14E-45	pDC	DNM2
RAN7	1.92E-49	0.633848	0.867	0.452	6.28E-45	pDC	RAN
MRPS62	2.60E-49	0.446305	0.615	0.233	8.51E-45	pDC	MRPS6
VEZF1	4.14E-49	0.393822	0.497	0.168	1.35E-44	pDC	VEZF1
NMT11	4.64E-49	0.397957	0.506	0.174	1.52E-44	pDC	NMT1
POP52	4.75E-49	0.385047	0.429	0.134	1.56E-44	pDC	POP5
TMEM248	6.60E-49	0.353499	0.494	0.165	2.16E-44	pDC	TMEM248
HM132	7.30E-49	0.614743	0.734	0.328	2.39E-44	pDC	HM13
GCC25	7.79E-49	0.503524	0.604	0.227	2.55E-44	pDC	GCC2
SMARCE1:	8.64E-49	0.469139	0.55	0.202	2.83E-44	pDC	SMARCE1
TMX3	9.00E-49	0.351638	0.299	0.076	2.95E-44	pDC	TMX3
BTK2	9.19E-49	0.309944	0.473	0.156	3.01E-44	pDC	BTK
AKAP91	1.03E-48	0.649369	0.746	0.34	3.36E-44	pDC	AKAP9
ADK2	1.25E-48	0.385221	0.393	0.119	4.10E-44	pDC	ADK
PPP6R1	1.42E-48	0.275535	0.34	0.094	4.66E-44	pDC	PPP6R1
SAMHD12	1.65E-48	0.732527	0.947	0.595	5.39E-44	pDC	SAMHD1
CDK63	2.01E-48	0.335859	0.263	0.062	6.58E-44	pDC	CDK6
SNX182	2.20E-48	0.378623	0.464	0.155	7.20E-44	pDC	SNX18
C11orf313	2.65E-48	0.643256	0.888	0.474	8.69E-44	pDC	C11orf31
RAB4B	2.86E-48	0.32527	0.373	0.109	9.38E-44	pDC	RAB4B
SCAMP21	3.01E-48	0.460257	0.683	0.28	9.85E-44	pDC	SCAMP2
ISCA2	3.17E-48	0.371549	0.396	0.12	1.04E-43	pDC	ISCA2
DLST	3.20E-48	0.283778	0.29	0.073	1.05E-43	pDC	DLST
SNX32	3.21E-48	0.408881	0.92	0.504	1.05E-43	pDC	SNX3
DNAJC12	3.34E-48	0.428227	0.515	0.179	1.09E-43	pDC	DNAJC1
XPA1	3.40E-48	0.369207	0.47	0.155	1.11E-43	pDC	XPA
KIF5B	3.83E-48	0.488731	0.663	0.271	1.25E-43	pDC	KIF5B
PSMA63	4.30E-48	0.479098	0.751	0.332	1.41E-43	pDC	PSMA6
TRPT1	4.43E-48	0.344819	0.29	0.074	1.45E-43	pDC	TRPT1
TOR3A	4.61E-48	0.361559	0.325	0.089	1.51E-43	pDC	TOR3A
HDAC92	5.06E-48	0.334514	0.32	0.086	1.66E-43	pDC	HDAC9
CIRBP7	6.40E-48	0.579774	0.885	0.446	2.10E-43	pDC	CIRBP
C15orf571	9.25E-48	0.284768	0.284	0.071	3.03E-43	pDC	C15orf57
GABARAPI	9.74E-48	0.395262	0.864	0.441	3.19E-43	pDC	GABARAPL2
MB21D1	1.24E-47	0.250607	0.334	0.091	4.07E-43	pDC	MB21D1
TBL1XR1	1.49E-47	0.312762	0.47	0.156	4.88E-43	pDC	TBL1XR1
RPAIN	1.67E-47	0.356281	0.473	0.156	5.47E-43	pDC	RPAIN
NACA7	2.01E-47	0.608251	0.988	0.82	6.59E-43	pDC	NACA
PCM11	2.15E-47	0.500667	0.598	0.23	7.05E-43	pDC	PCM1
ARID3A1	2.24E-47	0.336703	0.426	0.135	7.33E-43	pDC	ARID3A
EEF1A17	2.43E-47	0.535045	0.994	0.892	7.95E-43	pDC	EEF1A1

LINC-PINT	2.91E-47	0.346922	0.266	0.064	9.52E-43	pDC	LINC-PINT
TSSC1	3.20E-47	0.270988	0.243	0.055	1.05E-42	pDC	TSSC1
TMEM141	3.30E-47	0.36423	0.467	0.157	1.08E-42	pDC	TMEM141
PHF141	3.58E-47	0.42983	0.453	0.15	1.17E-42	pDC	PHF14
KPNA2	3.87E-47	0.281523	0.228	0.05	1.27E-42	pDC	KPNA2
APH1A3	3.94E-47	0.474043	0.633	0.252	1.29E-42	pDC	APH1A
TTC7A1	5.10E-47	0.332782	0.408	0.127	1.67E-42	pDC	TTC7A
NRD1	5.61E-47	0.328209	0.657	0.26	1.84E-42	pDC	NRD1
SRP721	5.86E-47	0.51658	0.612	0.241	1.92E-42	pDC	SRP72
SDF2L12	5.90E-47	0.379167	0.476	0.161	1.93E-42	pDC	SDF2L1
PSMA2.1	6.33E-47	0.348388	0.426	0.137	2.07E-42	pDC	PSMA2.1
ANKRD36C	6.44E-47	0.282842	0.204	0.041	2.11E-42	pDC	ANKRD36C
TRIM44	6.47E-47	0.421222	0.488	0.167	2.12E-42	pDC	TRIM44
PAICS1	7.32E-47	0.25862	0.207	0.043	2.40E-42	pDC	PAICS
TXNL4A	1.09E-46	0.478709	0.793	0.372	3.56E-42	pDC	TXNL4A
ANKRD11	1.31E-46	0.517713	0.716	0.311	4.29E-42	pDC	ANKRD11
HNRNPA1	1.43E-46	0.663334	0.959	0.629	4.68E-42	pDC	HNRNPA1
AMICA12	1.50E-46	0.414343	0.902	0.425	4.92E-42	pDC	AMICA1
TRRAP	1.51E-46	0.293656	0.24	0.055	4.95E-42	pDC	TRRAP
POLR2K2	1.71E-46	0.337321	0.476	0.159	5.61E-42	pDC	POLR2K
PIN12	2.25E-46	0.346744	0.562	0.203	7.37E-42	pDC	PIN1
U2AF1L42	2.76E-46	0.349647	0.281	0.072	9.02E-42	pDC	U2AF1L4
MIB22	2.86E-46	0.278284	0.281	0.07	9.38E-42	pDC	MIB2
ATP5G34	3.75E-46	0.592355	0.905	0.492	1.23E-41	pDC	ATP5G3
GSPT2	4.43E-46	0.267777	0.172	0.032	1.45E-41	pDC	GSPT2
YIF1A1	5.42E-46	0.367608	0.408	0.13	1.78E-41	pDC	YIF1A
LAMTOR5	6.41E-46	0.431927	0.74	0.324	2.10E-41	pDC	LAMTOR5
SLC25A20	6.57E-46	0.256428	0.266	0.065	2.15E-41	pDC	SLC25A20
LMAN22	6.67E-46	0.479954	0.713	0.309	2.18E-41	pDC	LMAN2
CPSF61	6.93E-46	0.305976	0.393	0.12	2.27E-41	pDC	CPSF6
UBE3A1	7.38E-46	0.45161	0.536	0.197	2.42E-41	pDC	UBE3A
PSMB5	7.54E-46	0.350604	0.473	0.159	2.47E-41	pDC	PSMB5
CHMP4B2	7.93E-46	0.404166	0.822	0.374	2.60E-41	pDC	CHMP4B
RPS125	1.37E-45	0.41551	0.985	0.882	4.50E-41	pDC	RPS12
CYC13	1.38E-45	0.430777	0.621	0.239	4.51E-41	pDC	CYC1
RRP151	1.67E-45	0.276875	0.278	0.07	5.46E-41	pDC	RRP15
UBTF3	2.00E-45	0.350364	0.447	0.147	6.53E-41	pDC	UBTF
TSTD16	2.29E-45	0.39562	0.497	0.171	7.50E-41	pDC	TSTD1
C1orf43	2.33E-45	0.405426	0.666	0.272	7.61E-41	pDC	C1orf43
ZMYND11	2.82E-45	0.326673	0.278	0.071	9.23E-41	pDC	ZMYND11
DNAJC195	3.23E-45	0.322973	0.47	0.156	1.06E-40	pDC	DNAJC19
RPS276	4.42E-45	0.509753	0.985	0.831	1.45E-40	pDC	RPS27
N4BP2L1	5.47E-45	0.281043	0.364	0.109	1.79E-40	pDC	N4BP2L1
RUFY31	5.51E-45	0.302187	0.26	0.065	1.80E-40	pDC	RUFY3
COX7A2L1	7.38E-45	0.524965	0.772	0.359	2.42E-40	pDC	COX7A2L
CRELD22	8.74E-45	0.32704	0.32	0.089	2.86E-40	pDC	CRELD2
SEC61A1	9.22E-45	0.385141	0.476	0.164	3.02E-40	pDC	SEC61A1
DAD13	1.12E-44	0.483756	0.822	0.403	3.67E-40	pDC	DAD1
NUDCD23	1.20E-44	0.398903	0.393	0.124	3.93E-40	pDC	NUDCD2
NUMA11	1.30E-44	0.413603	0.441	0.148	4.24E-40	pDC	NUMA1
MAP2K1	1.57E-44	0.314877	0.462	0.156	5.14E-40	pDC	MAP2K1
TMEM129	2.04E-44	0.259862	0.201	0.042	6.67E-40	pDC	TMEM129
DR1	2.60E-44	0.322461	0.473	0.163	8.52E-40	pDC	DR1
SLC46A3	3.11E-44	0.27723	0.24	0.057	1.02E-39	pDC	SLC46A3
INTS10	3.12E-44	0.370334	0.399	0.127	1.02E-39	pDC	INTS10
FAM207A	3.12E-44	0.278103	0.302	0.081	1.02E-39	pDC	FAM207A
LANCL1	3.19E-44	0.250041	0.175	0.033	1.04E-39	pDC	LANCL1
VPS13A2	3.36E-44	0.265212	0.251	0.061	1.10E-39	pDC	VPS13A

RMDN1	4.18E-44	0.306729	0.34	0.099	1.37E-39	pDC	RMDN1
RPL66	4.48E-44	0.497843	0.988	0.864	1.47E-39	pDC	RPL6
NUCKS14	5.02E-44	0.476077	0.657	0.281	1.64E-39	pDC	NUCKS1
ITPR11	5.20E-44	0.321684	0.284	0.075	1.70E-39	pDC	ITPR1
MTDH2	6.10E-44	0.578114	0.899	0.516	2.00E-39	pDC	MTDH
CHST122	8.73E-44	0.354628	0.355	0.105	2.86E-39	pDC	CHST12
NCL7	8.93E-44	0.564164	0.902	0.497	2.92E-39	pDC	NCL
DMXL1	9.29E-44	0.267014	0.272	0.07	3.04E-39	pDC	DMXL1
ATP5G11	9.92E-44	0.405404	0.66	0.274	3.25E-39	pDC	ATP5G1
SYNRG3	1.09E-43	0.355972	0.541	0.199	3.57E-39	pDC	SYNRG
KRR16	1.36E-43	0.33982	0.322	0.091	4.46E-39	pDC	KRR1
ERH4	1.76E-43	0.465118	0.763	0.351	5.77E-39	pDC	ERH
CNOT71	1.95E-43	0.326325	0.577	0.213	6.39E-39	pDC	CNOT7
C14orf13	1.97E-43	0.287001	0.249	0.061	6.44E-39	pDC	C14orf1
SERTAD3	2.08E-43	0.333752	0.311	0.088	6.83E-39	pDC	SERTAD3
GALNT2	2.19E-43	0.30232	0.322	0.092	7.16E-39	pDC	GALNT2
ENDOG	2.26E-43	0.258247	0.246	0.06	7.40E-39	pDC	ENDOG
DSTN1	2.41E-43	0.277591	0.645	0.262	7.90E-39	pDC	DSTN
ADPGK1	2.77E-43	0.346426	0.506	0.181	9.07E-39	pDC	ADPGK
PIK3CG1	2.89E-43	0.277037	0.275	0.072	9.46E-39	pDC	PIK3CG
HOXB22	2.91E-43	0.262777	0.189	0.039	9.52E-39	pDC	HOXB2
ELP2	3.00E-43	0.296653	0.308	0.085	9.82E-39	pDC	ELP2
ATP11B	3.22E-43	0.266698	0.34	0.099	1.05E-38	pDC	ATP11B
CCDC691	3.83E-43	0.343878	0.698	0.291	1.25E-38	pDC	CCDC69
TMCO12	4.00E-43	0.480845	0.683	0.294	1.31E-38	pDC	TMCO1
NDUFA7.1	4.25E-43	0.327234	0.379	0.12	1.39E-38	pDC	NDUFA7.1
MRP63	4.71E-43	0.380121	0.716	0.307	1.54E-38	pDC	MRP63
RBM6	4.89E-43	0.376725	0.544	0.205	1.60E-38	pDC	RBM6
EAF22	5.38E-43	0.270179	0.254	0.064	1.76E-38	pDC	EAF2
2-Sep	5.60E-43	0.403116	0.678	0.297	1.83E-38	pDC	2-Sep
CEBPZ-AS	5.78E-43	0.343073	0.343	0.103	1.89E-38	pDC	CEBPZ-AS1
APOA1BP2	6.24E-43	0.376521	0.482	0.171	2.04E-38	pDC	APOA1BP
MKKS1	6.27E-43	0.357944	0.456	0.159	2.05E-38	pDC	MKKS
CUL4B	7.01E-43	0.294158	0.24	0.058	2.29E-38	pDC	CUL4B
HVCN12	7.73E-43	0.384838	0.482	0.172	2.53E-38	pDC	HVCN1
CHPT12	8.30E-43	0.37242	0.417	0.14	2.72E-38	pDC	CHPT1
SDHC4	9.56E-43	0.385054	0.577	0.224	3.13E-38	pDC	SDHC
HSPB113	1.08E-42	0.316965	0.426	0.141	3.53E-38	pDC	HSPB11
MIA3	1.17E-42	0.426034	0.423	0.142	3.82E-38	pDC	MIA3
SYNGR23	1.65E-42	0.551407	0.772	0.371	5.40E-38	pDC	SYNGR2
HMGN22	1.69E-42	0.53976	0.902	0.526	5.54E-38	pDC	HMGN2
PBRM1	1.84E-42	0.404608	0.533	0.201	6.03E-38	pDC	PBRM1
ERGIC31	2.10E-42	0.421222	0.74	0.335	6.86E-38	pDC	ERGIC3
NUP2101	2.15E-42	0.301654	0.414	0.135	7.05E-38	pDC	NUP210
FAM96B	2.20E-42	0.431482	0.76	0.35	7.19E-38	pDC	FAM96B
ARID5A2	2.26E-42	0.303682	0.32	0.091	7.40E-38	pDC	ARID5A
RPA31	3.33E-42	0.299655	0.376	0.118	1.09E-37	pDC	RPA3
MED106	3.50E-42	0.339482	0.544	0.201	1.15E-37	pDC	MED10
ARHGEF7	4.82E-42	0.274655	0.281	0.075	1.58E-37	pDC	ARHGEF7
KIAA1430!	4.92E-42	0.274141	0.34	0.1	1.61E-37	pDC	KIAA1430
DARS2	6.03E-42	0.352735	0.464	0.163	1.98E-37	pDC	DARS
CCDC18	6.41E-42	0.279061	0.278	0.074	2.10E-37	pDC	CCDC18
DIMT11	8.01E-42	0.273914	0.24	0.059	2.62E-37	pDC	DIMT1
DYRK1B	8.02E-42	0.252348	0.18	0.037	2.63E-37	pDC	DYRK1B
ADD12	9.20E-42	0.396156	0.577	0.232	3.01E-37	pDC	ADD1
LMF2	9.43E-42	0.290102	0.325	0.095	3.09E-37	pDC	LMF2
RP11-773I	1.05E-41	0.265638	0.308	0.087	3.44E-37	pDC	RP11-773D16.1
HSDL2	1.09E-41	0.275967	0.302	0.085	3.56E-37	pDC	HSDL2

EIF51	1.12E-41	0.401811	0.772	0.356	3.68E-37	pDC	EIF5
IKZF14	1.19E-41	0.438889	0.754	0.341	3.90E-37	pDC	IKZF1
HIST1H4C	1.25E-41	0.530417	0.885	0.482	4.11E-37	pDC	HIST1H4C
FRYL1	1.32E-41	0.319029	0.494	0.177	4.33E-37	pDC	FRYL
TMEM242	1.41E-41	0.250622	0.24	0.059	4.60E-37	pDC	TMEM242
NCLN	1.42E-41	0.25403	0.281	0.076	4.64E-37	pDC	NCLN
ZMYM6NE	1.47E-41	0.361457	0.411	0.136	4.80E-37	pDC	ZMYM6NB
CDH231	1.54E-41	0.260165	0.243	0.06	5.05E-37	pDC	CDH23
GTF2A2	1.54E-41	0.37476	0.666	0.281	5.06E-37	pDC	GTF2A2
RPS66	1.58E-41	0.51553	0.988	0.782	5.17E-37	pDC	RPS6
QTRT1	1.71E-41	0.262135	0.231	0.055	5.59E-37	pDC	QTRT1
OS91	1.71E-41	0.467012	0.663	0.284	5.61E-37	pDC	OS9
HPS51	2.33E-41	0.256487	0.254	0.065	7.63E-37	pDC	HPS5
ACP51	2.45E-41	0.25003	0.263	0.068	8.04E-37	pDC	ACP5
DDX53	2.52E-41	0.589565	0.959	0.605	8.25E-37	pDC	DDX5
TMBIM6	2.78E-41	0.578542	0.956	0.621	9.09E-37	pDC	TMBIM6
DTNBP11	2.85E-41	0.378368	0.325	0.097	9.35E-37	pDC	DTNBP1
HDGFRP2	3.24E-41	0.251571	0.29	0.08	1.06E-36	pDC	HDGFRP2
FPGS1	3.89E-41	0.364789	0.302	0.086	1.27E-36	pDC	FPGS
IL2RG6	4.46E-41	0.327952	0.769	0.331	1.46E-36	pDC	IL2RG
TOP1	5.16E-41	0.41869	0.68	0.3	1.69E-36	pDC	TOP1
ATP5A12	5.66E-41	0.539201	0.849	0.437	1.85E-36	pDC	ATP5A1
M6PR2	7.50E-41	0.489712	0.743	0.346	2.46E-36	pDC	M6PR
PAG11	8.63E-41	0.321662	0.42	0.143	2.83E-36	pDC	PAG1
POLR3C	9.76E-41	0.295551	0.254	0.066	3.20E-36	pDC	POLR3C
C18orf32	1.15E-40	0.358484	0.53	0.206	3.77E-36	pDC	C18orf32
SUMO23	1.43E-40	0.51967	0.953	0.608	4.67E-36	pDC	SUMO2
NME1-NM	1.55E-40	0.600332	0.917	0.566	5.08E-36	pDC	NME1-NME2
HNRNPA0	1.64E-40	0.440268	0.66	0.291	5.36E-36	pDC	HNRNPA0
H2AFV	1.68E-40	0.422099	0.825	0.403	5.48E-36	pDC	H2AFV
OSBPL9	1.69E-40	0.291964	0.379	0.122	5.54E-36	pDC	OSBPL9
PTS	1.71E-40	0.250703	0.237	0.059	5.60E-36	pDC	PTS
ZMYM2	2.03E-40	0.33351	0.364	0.115	6.65E-36	pDC	ZMYM2
HERC2	2.45E-40	0.27481	0.402	0.134	8.03E-36	pDC	HERC2
KRT106	2.46E-40	0.461337	0.719	0.325	8.07E-36	pDC	KRT10
AFF1	2.70E-40	0.257563	0.317	0.093	8.86E-36	pDC	AFF1
H1FX1	2.84E-40	0.603247	0.775	0.405	9.30E-36	pDC	H1FX
CHCHD10	3.06E-40	0.435221	0.76	0.351	1.00E-35	pDC	CHCHD10
UBA25	3.17E-40	0.317827	0.456	0.16	1.04E-35	pDC	UBA2
TAPBPL1	3.66E-40	0.321662	0.314	0.093	1.20E-35	pDC	TAPBPL
ATP6V0A2	4.64E-40	0.308614	0.231	0.057	1.52E-35	pDC	ATP6V0A2
PTPN1	5.14E-40	0.340228	0.536	0.208	1.68E-35	pDC	PTPN1
NDUFB41	5.61E-40	0.419664	0.837	0.411	1.84E-35	pDC	NDUFB4
HAGH2	6.10E-40	0.374491	0.423	0.148	2.00E-35	pDC	HAGH
MAPK1IP1	6.12E-40	0.316904	0.479	0.177	2.00E-35	pDC	MAPK1IP1L
PWP13	6.45E-40	0.288913	0.402	0.133	2.11E-35	pDC	PWP1
C10orf32	7.78E-40	0.311392	0.379	0.123	2.55E-35	pDC	C10orf32
EEF1G6	9.22E-40	0.561173	0.885	0.523	3.02E-35	pDC	EEF1G
MDM41	1.14E-39	0.335243	0.462	0.167	3.74E-35	pDC	MDM4
AKAP17A	1.24E-39	0.272237	0.367	0.117	4.06E-35	pDC	AKAP17A
NDUFS82	1.26E-39	0.386917	0.689	0.296	4.12E-35	pDC	NDUFS8
SNAP291	1.46E-39	0.30951	0.45	0.16	4.76E-35	pDC	SNAP29
CHCHD3	1.53E-39	0.285114	0.352	0.111	5.01E-35	pDC	CHCHD3
RBX1	2.50E-39	0.315204	0.849	0.441	8.19E-35	pDC	RBX1
MTG13	2.80E-39	0.2662	0.331	0.1	9.16E-35	pDC	MTG1
SPATS2L1	3.32E-39	0.293059	0.213	0.051	1.09E-34	pDC	SPATS2L
HNRNPAB	3.49E-39	0.357957	0.609	0.248	1.14E-34	pDC	HNRNPAB
DAPK12	3.91E-39	0.296033	0.349	0.111	1.28E-34	pDC	DAPK1

UPF3B2	4.10E-39	0.303731	0.302	0.088	1.34E-34	pDC	UPF3B
ZNF506	4.34E-39	0.251429	0.222	0.054	1.42E-34	pDC	ZNF506
MMADHC	4.43E-39	0.342878	0.527	0.206	1.45E-34	pDC	MMADHC
MED11	4.94E-39	0.280307	0.308	0.09	1.62E-34	pDC	MED11
STARD7	5.48E-39	0.322443	0.459	0.166	1.80E-34	pDC	STARD7
DUT2	5.56E-39	0.379389	0.618	0.26	1.82E-34	pDC	DUT
CALM22	5.80E-39	0.555647	0.956	0.669	1.90E-34	pDC	CALM2
NAGA3	6.07E-39	0.309345	0.438	0.155	1.99E-34	pDC	NAGA
MCTS1	6.88E-39	0.334142	0.435	0.154	2.25E-34	pDC	MCTS1
EIF1AY2	9.04E-39	0.262785	0.382	0.126	2.96E-34	pDC	EIF1AY
GS1-251I9	1.15E-38	0.271391	0.414	0.141	3.76E-34	pDC	GS1-251I9.4
RCN25	1.15E-38	0.29839	0.379	0.122	3.77E-34	pDC	RCN2
TTC17	1.15E-38	0.328916	0.444	0.16	3.77E-34	pDC	TTC17
CHST11	1.28E-38	0.250006	0.337	0.104	4.20E-34	pDC	CHST11
RPS285	1.44E-38	0.430454	0.985	0.9	4.70E-34	pDC	RPS28
SLC20A11	1.51E-38	0.28902	0.382	0.125	4.95E-34	pDC	SLC20A1
ANAPC16(	2.09E-38	0.40468	0.722	0.336	6.86E-34	pDC	ANAPC16
GPN3	2.19E-38	0.264098	0.281	0.08	7.15E-34	pDC	GPN3
SLC25A36	2.35E-38	0.293153	0.343	0.108	7.70E-34	pDC	SLC25A36
UPF3A1	2.87E-38	0.285627	0.399	0.136	9.39E-34	pDC	UPF3A
TMEM131	3.18E-38	0.310032	0.396	0.135	1.04E-33	pDC	TMEM131
RELT2	3.22E-38	0.268285	0.328	0.102	1.05E-33	pDC	RELT
ZNF8002	3.39E-38	0.366284	0.476	0.178	1.11E-33	pDC	ZNF800
CCT82	4.29E-38	0.441821	0.68	0.308	1.40E-33	pDC	CCT8
RPL23A7	4.76E-38	0.544311	0.967	0.74	1.56E-33	pDC	RPL23A
RNF1302	5.00E-38	0.394858	0.917	0.454	1.64E-33	pDC	RNF130
PSMD9	7.42E-38	0.302547	0.473	0.175	2.43E-33	pDC	PSMD9
TMEM55B	7.81E-38	0.32245	0.299	0.089	2.56E-33	pDC	TMEM55B
LYN2	8.17E-38	0.378569	0.834	0.412	2.68E-33	pDC	LYN
UFM1	9.35E-38	0.327841	0.5	0.19	3.06E-33	pDC	UFM1
RPL106	9.77E-38	0.430949	0.997	0.879	3.20E-33	pDC	RPL10
RING1	1.02E-37	0.280331	0.302	0.09	3.34E-33	pDC	RING1
PPHLN13	1.28E-37	0.325365	0.423	0.149	4.20E-33	pDC	PPHLN1
NBPF102	1.56E-37	0.359847	0.604	0.254	5.11E-33	pDC	NBPF10
ETNK11	1.80E-37	0.269152	0.373	0.123	5.89E-33	pDC	ETNK1
LSM42	1.81E-37	0.395758	0.58	0.24	5.92E-33	pDC	LSM4
DDB1	1.83E-37	0.334785	0.414	0.146	6.01E-33	pDC	DDB1
AASDHPP	2.02E-37	0.295738	0.405	0.142	6.62E-33	pDC	AASDHPPT
EIF63	2.39E-37	0.303279	0.58	0.232	7.83E-33	pDC	EIF6
TNKS2	2.40E-37	0.364967	0.467	0.179	7.86E-33	pDC	TNKS2
C14orf166	2.56E-37	0.407922	0.757	0.355	8.39E-33	pDC	C14orf166
EP400	2.67E-37	0.309155	0.305	0.091	8.73E-33	pDC	EP400
LINC0087	2.96E-37	0.281653	0.231	0.06	9.68E-33	pDC	LINC00877
RPS6KA31	3.27E-37	0.262788	0.426	0.152	1.07E-32	pDC	RPS6KA3
TOR1AIP1	3.48E-37	0.31535	0.426	0.152	1.14E-32	pDC	TOR1AIP1
SCAF111	3.57E-37	0.441398	0.831	0.444	1.17E-32	pDC	SCAF11
SNHG86	3.69E-37	0.419138	0.669	0.293	1.21E-32	pDC	SNHG8
C1orf86	3.77E-37	0.328929	0.645	0.277	1.24E-32	pDC	C1orf86
PDPK1	3.80E-37	0.257488	0.379	0.127	1.24E-32	pDC	PDPK1
LMO41	4.23E-37	0.290771	0.562	0.226	1.39E-32	pDC	LMO4
NHP2L14	4.26E-37	0.41274	0.754	0.356	1.39E-32	pDC	NHP2L1
PCMTD11	4.64E-37	0.254349	0.515	0.201	1.52E-32	pDC	PCMTD1
TAF75	4.69E-37	0.284746	0.556	0.217	1.54E-32	pDC	TAF7
LAGE31	5.57E-37	0.303429	0.417	0.147	1.82E-32	pDC	LAGE3
CPT1A	6.56E-37	0.34893	0.355	0.119	2.15E-32	pDC	CPT1A
RPL376	8.02E-37	0.445665	0.982	0.868	2.62E-32	pDC	RPL37
IFNAR11	8.09E-37	0.340121	0.565	0.233	2.65E-32	pDC	IFNAR1
PHB26	8.77E-37	0.441007	0.734	0.347	2.87E-32	pDC	PHB2

THOC6	8.87E-37	0.284712	0.281	0.082	2.90E-32	pDC	THOC6
SETD5-AS	1.04E-36	0.330427	0.373	0.126	3.41E-32	pDC	SETD5-AS1
MFSD121	1.06E-36	0.264518	0.251	0.069	3.48E-32	pDC	MFSD12
HSPD16	1.12E-36	0.391677	0.627	0.269	3.67E-32	pDC	HSPD1
UBE2A	1.23E-36	0.267906	0.462	0.171	4.02E-32	pDC	UBE2A
COPE1	1.23E-36	0.447701	0.867	0.454	4.03E-32	pDC	COPE
BDP1	1.25E-36	0.338607	0.485	0.187	4.11E-32	pDC	BDP1
SUPT16H1	1.29E-36	0.379496	0.467	0.179	4.24E-32	pDC	SUPT16H
OCIAD14	1.54E-36	0.364234	0.651	0.29	5.06E-32	pDC	OCIAD1
LMBRD11	1.64E-36	0.318022	0.459	0.17	5.37E-32	pDC	LMBRD1
NDUFB61	2.09E-36	0.333346	0.562	0.232	6.84E-32	pDC	NDUFB6
TOMM204	2.12E-36	0.418423	0.743	0.345	6.95E-32	pDC	TOMM20
SMARCA5	2.38E-36	0.316303	0.55	0.222	7.79E-32	pDC	SMARCA5
UXT5	2.85E-36	0.444793	0.825	0.433	9.34E-32	pDC	UXT
CBX5	2.87E-36	0.259394	0.237	0.063	9.39E-32	pDC	CBX5
REST3	3.28E-36	0.357742	0.53	0.212	1.07E-31	pDC	REST
ERCC3	3.57E-36	0.257911	0.246	0.067	1.17E-31	pDC	ERCC3
IER3IP1.12	4.69E-36	0.289134	0.456	0.167	1.54E-31	pDC	IER3IP1.1
LPGAT11	5.00E-36	0.267166	0.571	0.234	1.64E-31	pDC	LPGAT1
NOP2	5.68E-36	0.285136	0.249	0.068	1.86E-31	pDC	NOP2
CHD22	5.91E-36	0.319572	0.521	0.204	1.94E-31	pDC	CHD2
SERBP12	7.57E-36	0.464827	0.876	0.503	2.48E-31	pDC	SERBP1
RNF132	9.59E-36	0.295516	0.604	0.25	3.14E-31	pDC	RNF13
REXO23	9.67E-36	0.276824	0.34	0.111	3.17E-31	pDC	REXO2
IMP4	9.91E-36	0.308143	0.379	0.13	3.24E-31	pDC	IMP4
FUS6	9.91E-36	0.440931	0.82	0.427	3.25E-31	pDC	FUS
DYNLL1	1.14E-35	0.37385	0.873	0.494	3.73E-31	pDC	DYNLL1
RBBP73	1.17E-35	0.330261	0.42	0.152	3.82E-31	pDC	RBBP7
PIK3AP12	1.25E-35	0.274377	0.541	0.217	4.10E-31	pDC	PIK3AP1
MRPS71	1.53E-35	0.302561	0.459	0.171	4.99E-31	pDC	MRPS7
HSPH11	1.62E-35	0.38871	0.34	0.114	5.29E-31	pDC	HSPH1
SKAP21	1.69E-35	0.265528	0.725	0.337	5.53E-31	pDC	SKAP2
PLEKHJ1	1.71E-35	0.322026	0.456	0.174	5.60E-31	pDC	PLEKHJ1
USP14	2.01E-35	0.259004	0.388	0.134	6.59E-31	pDC	USP14
SLC39A71	2.26E-35	0.261315	0.266	0.076	7.39E-31	pDC	SLC39A7
NCKAP1L2	2.31E-35	0.33284	0.612	0.263	7.55E-31	pDC	NCKAP1L
TMA161	2.43E-35	0.268638	0.352	0.117	7.96E-31	pDC	TMA16
MRPL19	2.65E-35	0.279835	0.355	0.119	8.67E-31	pDC	MRPL19
IGF2R2	2.74E-35	0.375398	0.53	0.214	8.98E-31	pDC	IGF2R
RPL7A7	3.21E-35	0.460065	0.991	0.839	1.05E-30	pDC	RPL7A
SUMO3	3.76E-35	0.257058	0.601	0.255	1.23E-30	pDC	SUMO3
PSMD11	4.40E-35	0.268575	0.488	0.186	1.44E-30	pDC	PSMD11
PIK3CD	4.75E-35	0.361991	0.491	0.196	1.55E-30	pDC	PIK3CD
NR3C11	5.09E-35	0.274281	0.675	0.297	1.67E-30	pDC	NR3C1
NDUFS41	5.16E-35	0.27249	0.417	0.152	1.69E-30	pDC	NDUFS4
CCT46	5.39E-35	0.364112	0.63	0.271	1.77E-30	pDC	CCT4
LSM31	5.76E-35	0.271503	0.595	0.248	1.89E-30	pDC	LSM3
OXR11	7.34E-35	0.318	0.373	0.131	2.40E-30	pDC	OXR1
NAA101	7.71E-35	0.280119	0.524	0.209	2.52E-30	pDC	NAA10
AZIN1	7.77E-35	0.266101	0.37	0.127	2.54E-30	pDC	AZIN1
SNRNP27	8.70E-35	0.253354	0.37	0.126	2.85E-30	pDC	SNRNP27
TFAM	9.43E-35	0.293471	0.432	0.161	3.09E-30	pDC	TFAM
SLC4A7	9.62E-35	0.35047	0.278	0.083	3.15E-30	pDC	SLC4A7
RPL356	9.65E-35	0.497123	0.976	0.77	3.16E-30	pDC	RPL35
EBNA1BP2	1.10E-34	0.253482	0.266	0.078	3.60E-30	pDC	EBNA1BP2
APRT5	1.11E-34	0.468477	0.885	0.514	3.64E-30	pDC	APRT
RP3-477O	1.21E-34	0.266635	0.21	0.054	3.97E-30	pDC	RP3-477O4.14
ANKRD26	1.22E-34	0.254828	0.201	0.05	3.99E-30	pDC	ANKRD26

AHSA12	1.45E-34	0.311117	0.388	0.136	4.73E-30	pDC	AHSA1
ACAA12	1.70E-34	0.30094	0.538	0.217	5.58E-30	pDC	ACAA1
RTFDC11	2.02E-34	0.303383	0.586	0.252	6.62E-30	pDC	RTFDC1
MTIF31	2.05E-34	0.309678	0.58	0.247	6.70E-30	pDC	MTIF3
FAM13B	2.16E-34	0.254894	0.349	0.117	7.07E-30	pDC	FAM13B
SSB4	2.17E-34	0.395969	0.654	0.3	7.11E-30	pDC	SSB
LAPTM4A1	2.21E-34	0.357178	0.645	0.285	7.22E-30	pDC	LAPTM4A
ATP5C13	2.31E-34	0.390687	0.746	0.366	7.56E-30	pDC	ATP5C1
MCCC2	2.53E-34	0.256288	0.207	0.053	8.29E-30	pDC	MCCC2
SCAMP31	2.92E-34	0.331642	0.408	0.148	9.58E-30	pDC	SCAMP3
URI16	3.17E-34	0.374211	0.485	0.194	1.04E-29	pDC	URI1
GGCT1	4.01E-34	0.291921	0.331	0.11	1.31E-29	pDC	GGCT
C19orf534	4.41E-34	0.393637	0.817	0.421	1.44E-29	pDC	C19orf53
SCOC	4.65E-34	0.271069	0.254	0.073	1.52E-29	pDC	SCOC
RFC12	5.84E-34	0.293501	0.47	0.18	1.91E-29	pDC	RFC1
PCF11	5.99E-34	0.313579	0.399	0.145	1.96E-29	pDC	PCF11
PEA152	6.18E-34	0.278837	0.396	0.143	2.02E-29	pDC	PEA15
ZNF326	6.51E-34	0.279656	0.266	0.079	2.13E-29	pDC	ZNF326
HNRNPC1	6.59E-34	0.444193	0.837	0.458	2.16E-29	pDC	HNRNPC
PSMD3	7.38E-34	0.274364	0.367	0.127	2.42E-29	pDC	PSMD3
TRPV22	9.09E-34	0.260699	0.32	0.103	2.98E-29	pDC	TRPV2
RPL56	9.66E-34	0.426686	0.985	0.8	3.16E-29	pDC	RPL5
SERINC3	1.10E-33	0.269212	0.568	0.244	3.61E-29	pDC	SERINC3
RPS186	1.13E-33	0.439242	0.985	0.789	3.71E-29	pDC	RPS18
ARCN1	1.14E-33	0.272009	0.393	0.142	3.75E-29	pDC	ARCN1
MRPL18	1.55E-33	0.257114	0.432	0.159	5.08E-29	pDC	MRPL18
CHRAC1	1.68E-33	0.288469	0.388	0.14	5.50E-29	pDC	CHRAC1
NUCB1	1.73E-33	0.25237	0.645	0.289	5.66E-29	pDC	NUCB1
RRP7A	1.94E-33	0.250284	0.479	0.186	6.35E-29	pDC	RRP7A
SET4	2.17E-33	0.428345	0.902	0.52	7.10E-29	pDC	SET
RANBP12	2.46E-33	0.273595	0.479	0.187	8.04E-29	pDC	RANBP1
HNRNPD1	2.57E-33	0.347011	0.769	0.378	8.40E-29	pDC	HNRNPD
SNRPB4	2.87E-33	0.368759	0.689	0.324	9.39E-29	pDC	SNRPB
NDUFS31	3.00E-33	0.325215	0.53	0.217	9.81E-29	pDC	NDUFS3
MAD2L21	3.04E-33	0.270948	0.364	0.128	9.96E-29	pDC	MAD2L2
SELK3	3.35E-33	0.286415	0.562	0.238	1.10E-28	pDC	SELK
VCP2	3.40E-33	0.353182	0.695	0.329	1.11E-28	pDC	VCP
PRPF191	3.70E-33	0.297455	0.322	0.108	1.21E-28	pDC	PRPF19
NDUFA81	3.80E-33	0.290963	0.393	0.144	1.24E-28	pDC	NDUFA8
SRSF77	4.55E-33	0.418769	0.71	0.343	1.49E-28	pDC	SRSF7
SUGT1	4.84E-33	0.304473	0.518	0.212	1.58E-28	pDC	SUGT1
USP36	5.59E-33	0.252974	0.225	0.062	1.83E-28	pDC	USP36
SRSF112	7.38E-33	0.37946	0.775	0.394	2.42E-28	pDC	SRSF11
TM9SF31	7.74E-33	0.295147	0.47	0.185	2.53E-28	pDC	TM9SF3
TMEM230:	7.94E-33	0.292985	0.562	0.236	2.60E-28	pDC	TMEM230
TCERG1	1.07E-32	0.253379	0.376	0.133	3.50E-28	pDC	TCERG1
SIRPB11	1.21E-32	0.260615	0.379	0.135	3.95E-28	pDC	SIRPB1
NSMCE13	1.28E-32	0.330833	0.432	0.166	4.18E-28	pDC	NSMCE1
WDR331	1.36E-32	0.265033	0.411	0.152	4.46E-28	pDC	WDR33
MX1	1.56E-32	0.418113	0.308	0.102	5.12E-28	pDC	MX1
NASP2	1.62E-32	0.279853	0.42	0.156	5.31E-28	pDC	NASP
TAF153	2.28E-32	0.373847	0.607	0.276	7.45E-28	pDC	TAF15
EMC42	2.53E-32	0.268	0.488	0.194	8.30E-28	pDC	EMC4
SYPL14	2.58E-32	0.268756	0.426	0.16	8.44E-28	pDC	SYPL1
RPS27A6	2.81E-32	0.334459	0.982	0.868	9.20E-28	pDC	RPS27A
HPS3	2.94E-32	0.278209	0.325	0.11	9.63E-28	pDC	HPS3
TMEM592	3.28E-32	0.374096	0.814	0.418	1.07E-27	pDC	TMEM59
RBM34	4.48E-32	0.451533	0.843	0.466	1.47E-27	pDC	RBM3

GTF2I1	5.06E-32	0.298222	0.636	0.291	1.66E-27	pDC	GTF2I
FOXP14	5.56E-32	0.251148	0.589	0.252	1.82E-27	pDC	FOXP1
CLPX	6.90E-32	0.259646	0.296	0.096	2.26E-27	pDC	CLPX
SRSF1	7.10E-32	0.284268	0.456	0.179	2.32E-27	pDC	SRSF1
MT-ND65	7.16E-32	0.394983	0.793	0.414	2.34E-27	pDC	MT-ND6
SNRPF5	8.55E-32	0.325629	0.642	0.291	2.80E-27	pDC	SNRPF
SMC31	8.55E-32	0.270735	0.438	0.169	2.80E-27	pDC	SMC3
CLPTM1	9.90E-32	0.300703	0.305	0.101	3.24E-27	pDC	CLPTM1
MGAT11	1.00E-31	0.288095	0.666	0.306	3.27E-27	pDC	MGAT1
RPL156	1.06E-31	0.471748	0.973	0.802	3.48E-27	pDC	RPL15
FAM162A(	1.07E-31	0.270653	0.388	0.141	3.50E-27	pDC	FAM162A
EIF3L5	1.23E-31	0.447205	0.891	0.54	4.04E-27	pDC	EIF3L
RBM8A2	1.23E-31	0.282508	0.74	0.352	4.04E-27	pDC	RBM8A
AP1M13	1.40E-31	0.268093	0.429	0.164	4.58E-27	pDC	AP1M1
BBX3	1.61E-31	0.287811	0.462	0.183	5.27E-27	pDC	BBX
RBM251	1.63E-31	0.335474	0.728	0.353	5.35E-27	pDC	RBM25
HNRNPUL	1.74E-31	0.300432	0.615	0.277	5.68E-27	pDC	HNRNPUL1
BRD21	2.23E-31	0.31161	0.636	0.292	7.31E-27	pDC	BRD2
PCNT	2.25E-31	0.272571	0.228	0.065	7.35E-27	pDC	PCNT
METTL233	2.34E-31	0.301469	0.346	0.122	7.66E-27	pDC	METTL23
AKR1A12	2.73E-31	0.291785	0.612	0.269	8.93E-27	pDC	AKR1A1
GNL2	2.74E-31	0.262508	0.284	0.091	8.97E-27	pDC	GNL2
ZNF652	2.77E-31	0.267259	0.423	0.163	9.08E-27	pDC	ZNF652
NAA201	2.77E-31	0.251367	0.402	0.149	9.08E-27	pDC	NAA20
FCGRT3	2.89E-31	0.336422	0.793	0.385	9.46E-27	pDC	FCGRT
HNRNPA3	2.94E-31	0.378937	0.843	0.457	9.61E-27	pDC	HNRNPA3
SF14	3.06E-31	0.380859	0.796	0.415	1.00E-26	pDC	SF1
SNRPA4	3.19E-31	0.344025	0.491	0.204	1.04E-26	pDC	SNRPA
U2AF11	3.39E-31	0.345854	0.793	0.408	1.11E-26	pDC	U2AF1
RPL37A6	3.66E-31	0.473682	0.973	0.808	1.20E-26	pDC	RPL37A
UFC13	3.88E-31	0.401456	0.725	0.361	1.27E-26	pDC	UFC1
BRK11	3.94E-31	0.322248	0.908	0.523	1.29E-26	pDC	BRK1
CCAR11	3.97E-31	0.276996	0.464	0.186	1.30E-26	pDC	CCAR1
ASCC3	4.06E-31	0.291191	0.314	0.107	1.33E-26	pDC	ASCC3
JMJD1C1	4.23E-31	0.315528	0.559	0.245	1.38E-26	pDC	JMJD1C
TREX12	5.01E-31	0.266757	0.438	0.173	1.64E-26	pDC	TREX1
BAZ1B	5.35E-31	0.296197	0.441	0.176	1.75E-26	pDC	BAZ1B
SERINC1	5.51E-31	0.294147	0.565	0.248	1.80E-26	pDC	SERINC1
CCDC914	7.87E-31	0.26619	0.337	0.117	2.58E-26	pDC	CCDC91
PSMC32	8.98E-31	0.257381	0.459	0.182	2.94E-26	pDC	PSMC3
SNRPD32	9.16E-31	0.294202	0.689	0.317	3.00E-26	pDC	SNRPD3
RPS37	1.05E-30	0.306251	0.982	0.832	3.43E-26	pDC	RPS3
SSRP11	1.14E-30	0.3024	0.376	0.14	3.73E-26	pDC	SSRP1
MAGT1	1.33E-30	0.254232	0.305	0.103	4.37E-26	pDC	MAGT1
TNRC6B1	1.40E-30	0.27049	0.642	0.291	4.59E-26	pDC	TNRC6B
LSM71	1.74E-30	0.378324	0.793	0.416	5.71E-26	pDC	LSM7
NAA38	1.85E-30	0.296156	0.601	0.272	6.07E-26	pDC	NAA38
SNRPG3	1.87E-30	0.338725	0.805	0.422	6.12E-26	pDC	SNRPG
TOP2B	1.94E-30	0.325985	0.361	0.132	6.34E-26	pDC	TOP2B
MTMR142	2.04E-30	0.268772	0.476	0.194	6.67E-26	pDC	MTMR14
GOLGA41	2.29E-30	0.284107	0.547	0.237	7.49E-26	pDC	GOLGA4
ORMDL1	2.39E-30	0.263162	0.473	0.193	7.82E-26	pDC	ORMDL1
ECI21	2.54E-30	0.265524	0.278	0.089	8.32E-26	pDC	ECI2
RPL7L11	2.57E-30	0.30498	0.459	0.185	8.40E-26	pDC	RPL7L1
SRSF92	2.84E-30	0.348962	0.84	0.458	9.31E-26	pDC	SRSF9
KDM5A1	2.91E-30	0.384275	0.601	0.278	9.51E-26	pDC	KDM5A
ITFG24	3.20E-30	0.304691	0.29	0.095	1.05E-25	pDC	ITFG2
TMEM50B	3.23E-30	0.301483	0.328	0.116	1.06E-25	pDC	TMEM50B

TAPBP2	3.57E-30	0.346841	0.79	0.413	1.17E-25	pDC	TAPBP
MRPS21	4.98E-30	0.391182	0.731	0.378	1.63E-25	pDC	MRPS21
TTC14	5.29E-30	0.26038	0.444	0.177	1.73E-25	pDC	TTC14
BST22	5.47E-30	0.458402	0.787	0.42	1.79E-25	pDC	BST2
DIDO1	6.08E-30	0.256549	0.34	0.12	1.99E-25	pDC	DIDO1
RFTN12	7.52E-30	0.267314	0.317	0.109	2.46E-25	pDC	RFTN1
EIF1AX	1.14E-29	0.327554	0.571	0.261	3.74E-25	pDC	EIF1AX
ATRAID1	1.22E-29	0.283124	0.541	0.235	4.00E-25	pDC	ATRAID
CTBP11	1.31E-29	0.255127	0.464	0.189	4.29E-25	pDC	CTBP1
EPC16	1.38E-29	0.288358	0.636	0.295	4.52E-25	pDC	EPC1
CLPP4	1.46E-29	0.278531	0.396	0.153	4.77E-25	pDC	CLPP
SAP183	1.49E-29	0.345774	0.799	0.423	4.89E-25	pDC	SAP18
PRPF40A1	1.60E-29	0.341912	0.686	0.336	5.23E-25	pDC	PRPF40A
SLC25A32	1.76E-29	0.461568	0.905	0.562	5.75E-25	pDC	SLC25A3
SNRNP20(	1.83E-29	0.332566	0.45	0.185	5.98E-25	pDC	SNRNP200
C8orf592	2.00E-29	0.300119	0.822	0.433	6.55E-25	pDC	C8orf59
SMIM15	2.29E-29	0.266907	0.331	0.118	7.51E-25	pDC	SMIM15
PRKDC	2.53E-29	0.286886	0.524	0.224	8.28E-25	pDC	PRKDC
TAP12	2.62E-29	0.29584	0.55	0.246	8.58E-25	pDC	TAP1
LSP14	2.72E-29	0.457575	0.95	0.67	8.90E-25	pDC	LSP1
MRPS333	2.75E-29	0.275934	0.364	0.135	8.99E-25	pDC	MRPS33
EEF25	2.86E-29	0.48984	0.97	0.699	9.37E-25	pDC	EEF2
KIAA0020:	3.08E-29	0.25805	0.299	0.101	1.01E-24	pDC	KIAA0020
SFPQ2	3.12E-29	0.361618	0.704	0.353	1.02E-24	pDC	SFPQ
RPS219	3.49E-29	0.427668	0.994	0.816	1.14E-24	pDC	RPS2
UBE2L3	4.22E-29	0.261482	0.683	0.327	1.38E-24	pDC	UBE2L3
BPTF1	4.42E-29	0.330912	0.607	0.282	1.45E-24	pDC	BPTF
HSP90AA1	4.73E-29	0.425864	0.95	0.616	1.55E-24	pDC	HSP90AA1
HLA-DQA	4.80E-29	0.444192	0.121	0.025	1.57E-24	pDC	HLA-DQA2
FBL7	5.67E-29	0.338966	0.618	0.286	1.86E-24	pDC	FBL
CD533	6.87E-29	0.374048	0.834	0.461	2.25E-24	pDC	CD53
TOMM52	6.99E-29	0.257173	0.598	0.269	2.29E-24	pDC	TOMM5
PRPF38B1	8.27E-29	0.293243	0.583	0.266	2.71E-24	pDC	PRPF38B
PRDX32	1.64E-28	0.269007	0.598	0.271	5.37E-24	pDC	PRDX3
ARGLU11	1.69E-28	0.290363	0.757	0.4	5.54E-24	pDC	ARGLU1
POLE3	2.81E-28	0.304506	0.399	0.16	9.21E-24	pDC	POLE3
TUFM2	2.98E-28	0.300225	0.675	0.323	9.75E-24	pDC	TUFM
ORAI31	3.03E-28	0.253953	0.37	0.141	9.93E-24	pDC	ORAI3
DENR	4.54E-28	0.254987	0.494	0.211	1.49E-23	pDC	DENR
PABPN11	5.69E-28	0.284885	0.482	0.211	1.86E-23	pDC	PABPN1
KDELR11	7.77E-28	0.302795	0.663	0.321	2.54E-23	pDC	KDELR1
TRMT1123	7.92E-28	0.33782	0.787	0.435	2.59E-23	pDC	TRMT112
RAD23A1	8.61E-28	0.295232	0.66	0.319	2.82E-23	pDC	RAD23A
RPL18A6	8.90E-28	0.374617	0.985	0.827	2.91E-23	pDC	RPL18A
EIF5A6	9.08E-28	0.315692	0.778	0.406	2.97E-23	pDC	EIF5A
CAPRIN1	9.22E-28	0.27923	0.456	0.196	3.02E-23	pDC	CAPRIN1
CCT6A3	9.41E-28	0.3606	0.621	0.297	3.08E-23	pDC	CCT6A
RPS2110	1.82E-27	0.332361	0.994	0.836	5.96E-23	pDC	RPS21
ANAPC11:	1.98E-27	0.266671	0.769	0.409	6.47E-23	pDC	ANAPC11
RPS198	2.26E-27	0.364192	0.979	0.815	7.40E-23	pDC	RPS19
TSPYL1	2.81E-27	0.253394	0.305	0.108	9.21E-23	pDC	TSPYL1
RAC26	3.04E-27	0.307985	0.944	0.579	9.94E-23	pDC	RAC2
BCLAF1	3.14E-27	0.323229	0.615	0.295	1.03E-22	pDC	BCLAF1
SSR26	3.20E-27	0.364573	0.864	0.483	1.05E-22	pDC	SSR2
GIT21	3.46E-27	0.272235	0.473	0.202	1.13E-22	pDC	GIT2
AUP11	3.94E-27	0.287776	0.577	0.267	1.29E-22	pDC	AUP1
PPID1	4.19E-27	0.275409	0.314	0.113	1.37E-22	pDC	PPID
TMEM666	4.58E-27	0.299176	0.926	0.549	1.50E-22	pDC	TMEM66

SUCLA2	4.96E-27	0.273409	0.269	0.091	1.62E-22	pDC	SUCLA2
ARID4B1	5.03E-27	0.298348	0.615	0.289	1.65E-22	pDC	ARID4B
SUZ12	5.08E-27	0.252459	0.393	0.158	1.66E-22	pDC	SUZ12
MYL12B3	5.81E-27	0.384263	0.935	0.639	1.90E-22	pDC	MYL12B
COX7A21	6.25E-27	0.339194	0.911	0.558	2.05E-22	pDC	COX7A2
RPL177	6.93E-27	0.472572	0.973	0.715	2.27E-22	pDC	RPL17
NANS3	7.72E-27	0.265429	0.352	0.137	2.53E-22	pDC	NANS
HNRNPDL	9.15E-27	0.335635	0.834	0.459	2.99E-22	pDC	HNRNPDL
SNX52	1.18E-26	0.259206	0.5	0.218	3.85E-22	pDC	SNX5
DNTTIP21	1.43E-26	0.252698	0.417	0.171	4.70E-22	pDC	DNTTIP2
GLG11	1.81E-26	0.257048	0.565	0.255	5.91E-22	pDC	GLG1
MS4A6A2	1.83E-26	0.569099	0.749	0.442	5.98E-22	pDC	MS4A6A
PRPF8	1.90E-26	0.264734	0.553	0.257	6.22E-22	pDC	PRPF8
RPS166	2.03E-26	0.416179	0.97	0.766	6.65E-22	pDC	RPS16
COX6A13	3.67E-26	0.364078	0.92	0.612	1.20E-21	pDC	COX6A1
ZCCHC71	3.73E-26	0.262125	0.287	0.101	1.22E-21	pDC	ZCCHC7
COX5A3	3.86E-26	0.319995	0.864	0.487	1.26E-21	pDC	COX5A
COX16	4.33E-26	0.269226	0.601	0.285	1.42E-21	pDC	COX16
MATR35	5.39E-26	0.288852	0.675	0.339	1.76E-21	pDC	MATR3
PSMD141	7.85E-26	0.25397	0.302	0.109	2.57E-21	pDC	PSMD14
RPS15A6	8.00E-26	0.280178	0.985	0.869	2.62E-21	pDC	RPS15A
JTB	8.41E-26	0.331475	0.781	0.436	2.75E-21	pDC	JTB
SNRPB21	9.72E-26	0.256055	0.636	0.306	3.18E-21	pDC	SNRPB2
DBI2	1.14E-25	0.359105	0.846	0.496	3.73E-21	pDC	DBI
NDUFAB1	1.30E-25	0.29327	0.683	0.349	4.24E-21	pDC	NDUFAB1
CDC401	2.15E-25	0.28818	0.518	0.235	7.03E-21	pDC	CDC40
FDFT13	2.35E-25	0.260235	0.491	0.216	7.70E-21	pDC	FDFT1
EIF4G1	3.04E-25	0.272825	0.47	0.209	9.94E-21	pDC	EIF4G1
PNRC15	4.24E-25	0.425425	0.964	0.669	1.39E-20	pDC	PNRC1
C15orf61	7.22E-25	0.253943	0.308	0.115	2.36E-20	pDC	C15orf61
C11orf57	1.05E-24	0.255386	0.287	0.104	3.42E-20	pDC	C11orf57
ATP5F12	1.26E-24	0.297958	0.834	0.461	4.12E-20	pDC	ATP5F1
RBM391	1.27E-24	0.280614	0.837	0.473	4.16E-20	pDC	RBM39
USMG5	1.41E-24	0.307291	0.899	0.562	4.60E-20	pDC	USMG5
ATPIF12	1.85E-24	0.299925	0.763	0.413	6.06E-20	pDC	ATPIF1
SDAD13	2.09E-24	0.267013	0.37	0.151	6.86E-20	pDC	SDAD1
H2AFY2	2.71E-24	0.269816	0.814	0.442	8.86E-20	pDC	H2AFY
EIF4A26	3.51E-24	0.288119	0.621	0.305	1.15E-19	pDC	EIF4A2
RPS297	4.98E-24	0.318483	0.964	0.765	1.63E-19	pDC	RPS29
HNRNPM1	1.09E-23	0.251476	0.678	0.348	3.56E-19	pDC	HNRNPM
CD372	1.26E-23	0.308899	0.973	0.695	4.11E-19	pDC	CD37
RPL136	1.35E-23	0.28523	0.985	0.862	4.42E-19	pDC	RPL13
KHDRBS14	1.39E-23	0.283546	0.746	0.395	4.54E-19	pDC	KHDRBS1
EID11	2.12E-23	0.251785	0.825	0.469	6.96E-19	pDC	EID1
EDF12	2.69E-23	0.342992	0.867	0.551	8.81E-19	pDC	EDF1
VPS13C1	3.26E-23	0.2755	0.636	0.327	1.07E-18	pDC	VPS13C
GUK13	3.44E-23	0.262079	0.861	0.508	1.12E-18	pDC	GUK1
RPL232	6.23E-23	0.368959	0.947	0.664	2.04E-18	pDC	RPL23
RPS76	7.20E-23	0.336854	0.991	0.865	2.36E-18	pDC	RPS7
RPS256	1.54E-22	0.268443	0.973	0.776	5.04E-18	pDC	RPS25
H3F3B1	2.40E-22	0.30941	0.994	0.828	7.87E-18	pDC	H3F3B
POLR2J3	3.48E-22	0.263617	0.379	0.163	1.14E-17	pDC	POLR2J3
RPL36A7	4.14E-22	0.400574	0.938	0.632	1.36E-17	pDC	RPL36A
ERAP11	7.17E-22	0.27655	0.26	0.096	2.35E-17	pDC	ERAP1
PSMB103	2.21E-21	0.251951	0.843	0.489	7.24E-17	pDC	PSMB10
THRAP33	2.68E-21	0.262014	0.639	0.332	8.78E-17	pDC	THRAP3
UBC3	2.78E-21	0.338322	0.979	0.758	9.10E-17	pDC	UBC
RBMX1	2.95E-21	0.252385	0.612	0.317	9.66E-17	pDC	RBMX

XRCC51	3.67E-21	0.263704	0.772	0.429	1.20E-16	pDC	XRCC5
HNRNPA2	1.78E-20	0.322148	0.973	0.715	5.82E-16	pDC	HNRNPA2B1
SRSF54	1.86E-20	0.262564	0.799	0.464	6.08E-16	pDC	SRSF5
PCBP25	2.69E-20	0.318908	0.932	0.639	8.81E-16	pDC	PCBP2
RPL276	3.46E-20	0.382	0.944	0.737	1.13E-15	pDC	RPL27
COX6C6	3.96E-20	0.305105	0.896	0.58	1.30E-15	pDC	COX6C
ATRX1	4.49E-20	0.259106	0.633	0.342	1.47E-15	pDC	ATRX
SRRM21	7.67E-20	0.271704	0.864	0.535	2.51E-15	pDC	SRRM2
RPS113	7.99E-20	0.372739	0.956	0.754	2.62E-15	pDC	RPS11
CCNI4	1.17E-19	0.345752	0.947	0.693	3.82E-15	pDC	CCNI
RPL216	1.28E-19	0.327124	0.962	0.815	4.20E-15	pDC	RPL21
RPS205	2.89E-19	0.339404	0.882	0.591	9.45E-15	pDC	RPS20
EIF3F6	3.38E-19	0.32039	0.87	0.541	1.11E-14	pDC	EIF3F
HSPA88	6.59E-19	0.276045	0.92	0.576	2.16E-14	pDC	HSPA8
GNB2L16	7.53E-19	0.333096	0.991	0.8	2.47E-14	pDC	GNB2L1
TPT13	2.79E-18	0.27749	0.985	0.912	9.15E-14	pDC	TPT1
SON1	8.64E-18	0.282989	0.938	0.649	2.83E-13	pDC	SON
RPL13A6	3.54E-15	0.294521	0.967	0.767	1.16E-10	pDC	RPL13A
RPL267	9.21E-15	0.324602	0.979	0.806	3.02E-10	pDC	RPL26
RPL246	1.22E-13	0.273936	0.979	0.801	3.99E-09	pDC	RPL24
XIST1	7.58E-13	0.269553	0.254	0.121	2.48E-08	pDC	XIST
PPBP	0	7.441733	0.987	0.108	0	Platelet	PPBP
TUBB11	0	6.062211	0.96	0.04	0	Platelet	TUBB1
NRGN	0	5.914844	0.984	0.249	0	Platelet	NRGN
PF4	0	5.775636	0.945	0.055	0	Platelet	PF4
MYL9	0	5.256372	0.837	0.033	0	Platelet	MYL9
PRKAR2B	0	5.247683	0.903	0.069	0	Platelet	PRKAR2B
SDPR	0	5.190061	0.882	0.039	0	Platelet	SDPR
SPARC	0	5.152781	0.881	0.035	0	Platelet	SPARC
GP9	0	5.10491	0.865	0.022	0	Platelet	GP9
C6orf25	0	4.936009	0.853	0.026	0	Platelet	C6orf25
GNG11	0	4.904264	0.849	0.033	0	Platelet	GNG11
HIST1H2A	0	4.90213	0.825	0.114	0	Platelet	HIST1H2AC
TREML1	0	4.701821	0.798	0.023	0	Platelet	TREML1
ITGA2B	0	4.565204	0.757	0.021	0	Platelet	ITGA2B
CLU	0	4.485526	0.873	0.161	0	Platelet	CLU
CMTM5	0	4.425432	0.752	0.014	0	Platelet	CMTM5
F13A1	0	4.33976	0.81	0.147	0	Platelet	F13A1
HBD1	0	4.276415	0.502	0.022	0	Platelet	HBD
RGS18	0	4.217497	0.803	0.272	0	Platelet	RGS18
TAGLN21	0	4.192353	0.986	0.61	0	Platelet	TAGLN2
TSC22D1	0	4.18698	0.502	0.028	0	Platelet	TSC22D1
GRAP2	0	4.028398	0.725	0.082	0	Platelet	GRAP2
TUBA4A	0	4.019115	0.775	0.228	0	Platelet	TUBA4A
TPM1	0	4.012072	0.712	0.092	0	Platelet	TPM1
VCL	0	3.979902	0.769	0.21	0	Platelet	VCL
PDLIM11	0	3.941386	0.697	0.05	0	Platelet	PDLIM1
NGFRAP1	0	3.902217	0.668	0.054	0	Platelet	NGFRAP1
TPM4	0	3.894716	0.857	0.366	0	Platelet	TPM4
PTGS1	0	3.8272	0.663	0.052	0	Platelet	PTGS1
YWHAH	0	3.805543	0.783	0.296	0	Platelet	YWHAH
SNCA1	0	3.797498	0.668	0.07	0	Platelet	SNCA
TMEM40	0	3.773554	0.529	0.01	0	Platelet	TMEM40
PF4V1	0	3.748047	0.527	0.016	0	Platelet	PF4V1
MMD	0	3.727241	0.508	0.037	0	Platelet	MMD
CTSA	0	3.668366	0.852	0.331	0	Platelet	CTSA
PGRMC1	0	3.658102	0.64	0.079	0	Platelet	PGRMC1
THBS1	0	3.607994	0.522	0.042	0	Platelet	THBS1

GPX1	0	3.607098	0.974	0.697	0	Platelet	GPX1
2-Mar	0	3.560745	0.693	0.171	0	Platelet	2-Mar
ESAM	0	3.541833	0.522	0.009	0	Platelet	ESAM
CD9	0	3.519442	0.522	0.028	0	Platelet	CD9
RGS10	0	3.498878	0.818	0.455	0	Platelet	RGS10
ODC1	0	3.472077	0.561	0.102	0	Platelet	ODC1
MPP11	0	3.451572	0.668	0.212	0	Platelet	MPP1
KIF2A	0	3.448018	0.656	0.224	0	Platelet	KIF2A
ITGB3	0	3.447444	0.533	0.011	0	Platelet	ITGB3
LIMS1	0	3.435495	0.699	0.284	0	Platelet	LIMS1
TLN1	0	3.42647	0.902	0.625	0	Platelet	TLN1
GMPR1	0	3.416186	0.539	0.024	0	Platelet	GMPR
OST4	0	3.390068	0.953	0.728	0	Platelet	OST4
TGFB1	0	3.365369	0.886	0.591	0	Platelet	TGFB1
HIST1H3H	0	3.345526	0.393	0.022	0	Platelet	HIST1H3H
PARVB	0	3.304455	0.542	0.121	0	Platelet	PARVB
C2orf88	0	3.295752	0.47	0.013	0	Platelet	C2orf88
STOM	0	3.28387	0.65	0.165	0	Platelet	STOM
ILK	0	3.277067	0.636	0.262	0	Platelet	ILK
CALM3	0	3.250525	0.843	0.463	0	Platelet	CALM3
RSU1	0	3.204009	0.623	0.208	0	Platelet	RSU1
SNN	0	3.196639	0.554	0.123	0	Platelet	SNN
AP003068.	0	3.195442	0.47	0.013	0	Platelet	AP003068.23
NT5C3A	0	3.142667	0.451	0.14	0	Platelet	NT5C3A
MAX	0	3.138533	0.621	0.267	0	Platelet	MAX
TMEM158	0	3.136788	0.435	0.009	0	Platelet	TMEM158
RAP1B1	0	3.112501	0.712	0.485	0	Platelet	RAP1B
HIST1H2B.	0	3.111816	0.371	0.015	0	Platelet	HIST1H2BJ
FERMT3	0	3.105185	0.759	0.418	0	Platelet	FERMT3
CCL52	0	3.10435	0.893	0.188	0	Platelet	CCL5
CYB5R3	0	3.083925	0.603	0.208	0	Platelet	CYB5R3
ARHGAP1	0	3.070915	0.496	0.096	0	Platelet	ARHGAP18
RP11-367(	0	3.062896	0.342	0.023	0	Platelet	RP11-367G6.3
TRIM581	0	3.052729	0.425	0.023	0	Platelet	TRIM58
RNF11	0	3.025519	0.535	0.126	0	Platelet	RNF11
ENDOD1	0	3.022286	0.453	0.037	0	Platelet	ENDOD1
ACRBP	0	3.021333	0.399	0.027	0	Platelet	ACRBP
5-Sep	0	3.0154	0.463	0.006	0	Platelet	5-Sep
ACTN1	0	2.975137	0.631	0.208	0	Platelet	ACTN1
CDKN1A	0	2.97091	0.392	0.055	0	Platelet	CDKN1A
RAB27B	0	2.966827	0.393	0.026	0	Platelet	RAB27B
DMTN1	0	2.936688	0.44	0.018	0	Platelet	DMTN
WBP2	0	2.932736	0.58	0.203	0	Platelet	WBP2
NAP1L11	0	2.923752	0.808	0.516	0	Platelet	NAP1L1
CCND31	0	2.886676	0.729	0.425	0	Platelet	CCND3
NCOA41	0	2.876953	0.684	0.406	0	Platelet	NCOA4
MFSD1	0	2.865179	0.648	0.289	0	Platelet	MFSD1
C12orf753	0	2.863432	0.494	0.122	0	Platelet	C12orf75
GAS2L1	0	2.845209	0.422	0.039	0	Platelet	GAS2L1
RAB11A	0	2.842213	0.551	0.284	0	Platelet	RAB11A
SLC40A1	0	2.836717	0.393	0.122	0	Platelet	SLC40A1
MAP3K7CI	0	2.833646	0.288	0.046	0	Platelet	MAP3K7CL
PLA2G12A	0	2.8313	0.434	0.048	0	Platelet	PLA2G12A
CLEC1B	0	2.816364	0.369	0.006	0	Platelet	CLEC1B
CD151	0	2.80703	0.499	0.152	0	Platelet	CD151
FAXDC2	0	2.805235	0.407	0.009	0	Platelet	FAXDC2
SH3BGRL2	0	2.802421	0.404	0.011	0	Platelet	SH3BGRL2
EIF2AK1	0	2.780217	0.444	0.175	0	Platelet	EIF2AK1

GP1BA	0	2.775175	0.368	0.005	0	Platelet	GP1BA
RUFY1	0	2.762796	0.492	0.153	0	Platelet	RUFY1
TIMP11	0	2.755396	0.803	0.5	0	Platelet	TIMP1
CDKN2D	0	2.751121	0.595	0.274	0	Platelet	CDKN2D
PRDX6	0	2.724821	0.635	0.343	0	Platelet	PRDX6
GUCY1B3	0	2.721542	0.381	0.015	0	Platelet	GUCY1B3
ZYX	0	2.660733	0.714	0.545	0	Platelet	ZYX
WDR1	0	2.646009	0.669	0.408	0	Platelet	WDR1
PKM	0	2.642572	0.852	0.631	0	Platelet	PKM
SH3BGRL3	0	2.608999	0.979	0.946	0	Platelet	SH3BGRL3
CA21	0	2.607854	0.323	0.033	0	Platelet	CA2
LMNA1	0	2.604582	0.376	0.038	0	Platelet	LMNA
MYLK	0	2.603522	0.356	0.008	0	Platelet	MYLK
MCUR1	0	2.592241	0.409	0.092	0	Platelet	MCUR1
MTURN	0	2.590899	0.362	0.025	0	Platelet	MTURN
YWHAZ1	0	2.578561	0.816	0.653	0	Platelet	YWHAZ
TUBA8	0	2.573414	0.348	0.004	0	Platelet	TUBA8
GPX4	0	2.573061	0.797	0.63	0	Platelet	GPX4
LAMTOR1	0	2.572716	0.737	0.486	0	Platelet	LAMTOR1
OAZ1	0	2.556278	0.98	0.91	0	Platelet	OAZ1
FHL1	0	2.552864	0.346	0.022	0	Platelet	FHL1
TMSB4X	0	2.524585	0.987	0.972	0	Platelet	TMSB4X
ARHGAP6	0	2.513872	0.326	0.014	0	Platelet	ARHGAP6
C19orf33	0	2.482884	0.314	0.014	0	Platelet	C19orf33
TUBA1C	0	2.480937	0.373	0.119	0	Platelet	TUBA1C
LTBP1	0	2.472973	0.331	0.005	0	Platelet	LTBP1
ANO6	0	2.470163	0.442	0.182	0	Platelet	ANO6
FRMD3	0	2.467454	0.209	0.018	0	Platelet	FRMD3
TMEM140	0	2.455274	0.261	0.044	0	Platelet	TMEM140
MGLL	0	2.4417	0.344	0.036	0	Platelet	MGLL
ADIPOR11	0	2.440395	0.474	0.24	0	Platelet	ADIPOR1
LY6G6F	0	2.437182	0.328	0.004	0	Platelet	LY6G6F
LAT6	0	2.434125	0.43	0.16	0	Platelet	LAT
SVIP2	0	2.420183	0.38	0.128	0	Platelet	SVIP
C9orf89	0	2.41645	0.395	0.145	0	Platelet	C9orf89
C9orf16	0	2.415137	0.63	0.473	0	Platelet	C9orf16
DAB21	0	2.40721	0.292	0.057	0	Platelet	DAB2
CDK2AP11	0	2.403414	0.4	0.135	0	Platelet	CDK2AP1
TPST21	0	2.39298	0.432	0.187	0	Platelet	TPST2
NDUFAF3	0	2.377249	0.52	0.305	0	Platelet	NDUFAF3
HBG21	0	2.374267	0.201	0.008	0	Platelet	HBG2
AC147651	0	2.373693	0.285	0.005	0	Platelet	AC147651.3
PPDPF	0	2.372469	0.798	0.667	0	Platelet	PPDPF
SELP	0	2.363829	0.307	0.005	0	Platelet	SELP
ARF1	0	2.361712	0.669	0.502	0	Platelet	ARF1
TPTEP1	0	2.34576	0.296	0.033	0	Platelet	TPTEP1
RP11-501J	0	2.342016	0.29	0.004	0	Platelet	RP11-501J20.5
NEXN	0	2.330649	0.284	0.038	0	Platelet	NEXN
ACTB1	0	2.317064	0.992	0.993	0	Platelet	ACTB
ALOX12	0	2.312759	0.303	0.004	0	Platelet	ALOX12
RDH11	0	2.308791	0.375	0.117	0	Platelet	RDH11
CD226	0	2.304743	0.331	0.036	0	Platelet	CD226
MTPN	0	2.298242	0.708	0.54	0	Platelet	MTPN
RBPMS2	0	2.296922	0.285	0.006	0	Platelet	RBPMS2
GSN2	0	2.285815	0.424	0.189	0	Platelet	GSN
SNAP23	0	2.278201	0.468	0.247	0	Platelet	SNAP23
CAPN1	0	2.267242	0.42	0.175	0	Platelet	CAPN1
ABCC3	0	2.255915	0.328	0.045	0	Platelet	ABCC3

MYL12A4	0	2.252893	0.868	0.753	0	Platelet	MYL12A
TLK1	0	2.239478	0.376	0.154	0	Platelet	TLK1
YIF1B	0	2.238982	0.406	0.184	0	Platelet	YIF1B
FTH11	0	2.238594	0.998	0.968	0	Platelet	FTH1
HLA-E2	0	2.236178	0.904	0.8	0	Platelet	HLA-E
PTCRA1	0	2.233215	0.263	0.011	0	Platelet	PTCRA
PRKAR1B	0	2.22478	0.295	0.023	0	Platelet	PRKAR1B
NT5M	0	2.222691	0.278	0.007	0	Platelet	NT5M
CD992	0	2.21413	0.746	0.578	0	Platelet	CD99
MMRN1	0	2.205446	0.26	0.006	0	Platelet	MMRN1
FLNA	0	2.205051	0.652	0.563	0	Platelet	FLNA
TAX1BP3	0	2.1986	0.324	0.078	0	Platelet	TAX1BP3
HIST1H2BI	0	2.191281	0.336	0.092	0	Platelet	HIST1H2BK
ITGB11	0	2.18724	0.599	0.416	0	Platelet	ITGB1
MYH9	0	2.181821	0.652	0.544	0	Platelet	MYH9
F11R	0	2.181356	0.324	0.072	0	Platelet	F11R
LGALSL	0	2.173899	0.257	0.005	0	Platelet	LGALSL
ARPC1B	0	2.17024	0.914	0.849	0	Platelet	ARPC1B
TUBA1B3	0	2.167901	0.587	0.434	0	Platelet	TUBA1B
EHD3	0	2.157117	0.284	0.011	0	Platelet	EHD3
TUBB4B	0	2.15389	0.389	0.172	0	Platelet	TUBB4B
MAP1A1	0	2.144074	0.264	0.012	0	Platelet	MAP1A
CTTN	0	2.117426	0.27	0.005	0	Platelet	CTTN
AGPAT1	0	2.087018	0.318	0.082	0	Platelet	AGPAT1
STON2	0	2.085033	0.232	0.008	0	Platelet	STON2
PROS1	0	2.077984	0.245	0.005	0	Platelet	PROS1
PDE5A	0	2.060289	0.241	0.005	0	Platelet	PDE5A
TMEM91	0	2.05877	0.354	0.12	0	Platelet	TMEM91
GNAS	0	2.055458	0.629	0.513	0	Platelet	GNAS
SLA2	0	2.044547	0.265	0.036	0	Platelet	SLA2
CALD1	0	2.022334	0.234	0.005	0	Platelet	CALD1
RP11-879I	0	2.017421	0.238	0.004	0	Platelet	RP11-879F14.2
LINC0098(	0	2.009269	0.236	0.004	0	Platelet	LINC00989
C12orf39	0	1.994484	0.239	0.003	0	Platelet	C12orf39
RAB371	0	1.976179	0.317	0.096	0	Platelet	RAB37
ANKRD9	0	1.970858	0.231	0.009	0	Platelet	ANKRD9
ACTG12	0	1.962348	0.869	0.881	0	Platelet	ACTG1
SLC10A3	0	1.957233	0.292	0.073	0	Platelet	SLC10A3
TGFB1I1	0	1.943557	0.242	0.004	0	Platelet	TGFB1I1
PDGFA	0	1.931643	0.217	0.003	0	Platelet	PDGFA
HIST1H2B(	0	1.927715	0.214	0.033	0	Platelet	HIST1H2BC
MYL6	0	1.927325	0.915	0.938	0	Platelet	MYL6
PPP1R14A	0	1.926892	0.23	0.015	0	Platelet	PPP1R14A
FAM63A	0	1.897425	0.271	0.068	0	Platelet	FAM63A
GNB5	0	1.871876	0.244	0.036	0	Platelet	GNB5
EGFL7	0	1.86604	0.221	0.008	0	Platelet	EGFL7
ABLIM3	0	1.860448	0.218	0.004	0	Platelet	ABLIM3
GP6	0	1.858018	0.231	0.002	0	Platelet	GP6
ITM2B	0	1.851859	0.882	0.864	0	Platelet	ITM2B
SMOX	0	1.823187	0.192	0.01	0	Platelet	SMOX
GFI1B	0	1.821258	0.205	0.004	0	Platelet	GFI1B
ARG2	0	1.797561	0.189	0.005	0	Platelet	ARG2
SMIM51	0	1.788797	0.165	0.01	0	Platelet	SMIM5
SAT11	0	1.784164	0.751	0.622	0	Platelet	SAT1
HBE1	0	1.772126	0.105	0.002	0	Platelet	HBE1
ARHGDIB2	0	1.745224	0.842	0.786	0	Platelet	ARHGDIB
DNM3	0	1.70771	0.196	0.004	0	Platelet	DNM3
MFAP3L	0	1.705108	0.191	0.004	0	Platelet	MFAP3L

PNMA1	0	1.703631	0.183	0.027	0	Platelet	PNMA1
MFSD2B	0	1.694283	0.202	0.003	0	Platelet	MFSD2B
F2R1	0	1.689637	0.198	0.032	0	Platelet	F2R
H3F3A1	0	1.686482	0.967	0.939	0	Platelet	H3F3A
ZNF185	0	1.676556	0.218	0.046	0	Platelet	ZNF185
ABCC4	0	1.673494	0.185	0.019	0	Platelet	ABCC4
CLDN5	0	1.660768	0.147	0.003	0	Platelet	CLDN5
AP001189.	0	1.651999	0.134	0.006	0	Platelet	AP001189.4
JAM3	0	1.643415	0.184	0.005	0	Platelet	JAM3
ENKUR	0	1.641905	0.154	0.003	0	Platelet	ENKUR
SAMD14	0	1.62827	0.176	0.002	0	Platelet	SAMD14
AQP10	0	1.627582	0.167	0.002	0	Platelet	AQP10
BEND2	0	1.622701	0.177	0.003	0	Platelet	BEND2
SSX2IP	0	1.616304	0.187	0.02	0	Platelet	SSX2IP
TFPI	0	1.593831	0.171	0.004	0	Platelet	TFPI
FAH	0	1.58869	0.202	0.032	0	Platelet	FAH
TAL1	0	1.583976	0.176	0.003	0	Platelet	TAL1
BZRAP1-A	0	1.576723	0.202	0.042	0	Platelet	BZRAP1-AS1
PRUNE	0	1.537604	0.187	0.036	0	Platelet	PRUNE
IGF2BP3	0	1.515777	0.156	0.011	0	Platelet	IGF2BP3
SRGN1	0	1.514401	0.773	0.781	0	Platelet	SRGN
CTDSPL	0	1.512719	0.184	0.005	0	Platelet	CTDSPL
LAPTM4B	0	1.500068	0.158	0.012	0	Platelet	LAPTM4B
TSPAN9	0	1.493435	0.168	0.003	0	Platelet	TSPAN9
LGALS121	0	1.487518	0.173	0.018	0	Platelet	LGALS12
SCN1B	0	1.484968	0.146	0.005	0	Platelet	SCN1B
ELOVL7	0	1.480529	0.162	0.003	0	Platelet	ELOVL7
PDZK1IP1:	0	1.480207	0.123	0.009	0	Platelet	PDZK1IP1
TNNC2	0	1.46514	0.147	0.004	0	Platelet	TNNC2
GNAZ	0	1.453242	0.166	0.002	0	Platelet	GNAZ
TBXA2R	0	1.431549	0.163	0.005	0	Platelet	TBXA2R
CDC14B	0	1.423935	0.17	0.011	0	Platelet	CDC14B
ASAP2	0	1.406094	0.16	0.008	0	Platelet	ASAP2
HBQ11	0	1.393503	0.152	0.011	0	Platelet	HBQ1
KIFC3	0	1.358166	0.139	0.014	0	Platelet	KIFC3
RAB6B	0	1.356869	0.147	0.002	0	Platelet	RAB6B
ITGB5	0	1.356786	0.147	0.004	0	Platelet	ITGB5
TSPAN132	0	1.349206	0.161	0.018	0	Platelet	TSPAN13
SYTL4	0	1.343874	0.148	0.002	0	Platelet	SYTL4
GUCY1A3	0	1.343413	0.15	0.012	0	Platelet	GUCY1A3
GATA1	0	1.329425	0.129	0.002	0	Platelet	GATA1
BMP6	0	1.315962	0.122	0.001	0	Platelet	BMP6
EGLN31	0	1.312696	0.127	0.007	0	Platelet	EGLN3
PLOD2	0	1.281668	0.137	0.002	0	Platelet	PLOD2
XK	0	1.268633	0.119	0.004	0	Platelet	XK
CFL12	0	1.25809	0.826	0.885	0	Platelet	CFL1
MSANTD3	0	1.194988	0.138	0.012	0	Platelet	MSANTD3
TRAPPC3L	0	1.192545	0.115	0.003	0	Platelet	TRAPPC3L
PKHD1L1	0	1.189472	0.13	0.003	0	Platelet	PKHD1L1
TSPAN331	0	1.170652	0.122	0.011	0	Platelet	TSPAN33
HOMER2	0	1.1612	0.123	0.008	0	Platelet	HOMER2
C1orf198	0	1.15001	0.109	0.005	0	Platelet	C1orf198
MEIS1	0	1.149311	0.116	0.002	0	Platelet	MEIS1
SERF21	0	1.143491	0.899	0.957	0	Platelet	SERF2
PKIG1	0	1.130245	0.121	0.013	0	Platelet	PKIG
RHOBTB1	0	1.122262	0.12	0.005	0	Platelet	RHOBTB1
TNFSF4	0	1.090414	0.102	0.003	0	Platelet	TNFSF4
HGD	0	1.065667	0.101	0.002	0	Platelet	HGD

PBX1	0	1.045945	0.102	0.003	0	Platelet	PBX1
B2M3	0	1.039448	0.999	0.977	0	Platelet	B2M
CPNE51	0	1.025953	0.1	0.007	0	Platelet	CPNE5
UBE2C	0	1.012569	0.1	0.002	0	Platelet	UBE2C
FN3K	0	1.006446	0.108	0.009	0	Platelet	FN3K
HLA-A2	0	1.005604	0.855	0.866	0	Platelet	HLA-A
TSPAN18	0	0.995067	0.113	0.01	0	Platelet	TSPAN18
NCK2	#######	1.583433	0.19	0.043	#######	Platelet	NCK2
APP1	#######	2.125607	0.375	0.163	#######	Platelet	APP
SLC44A21	#######	2.057499	0.363	0.154	#######	Platelet	SLC44A2
CLCN3	#######	1.947645	0.282	0.094	#######	Platelet	CLCN3
RHOA1	#######	1.301657	0.721	0.767	#######	Platelet	RHOA
BCL2L12	#######	1.85404	0.283	0.095	#######	Platelet	BCL2L1
GADD45A	#######	1.515197	0.184	0.04	#######	Platelet	GADD45A
RAB1B	#######	2.151871	0.461	0.262	#######	Platelet	RAB1B
CRAT	#######	1.502824	0.191	0.044	#######	Platelet	CRAT
ATP2A3	#######	1.77293	0.275	0.09	#######	Platelet	ATP2A3
INF2	#######	1.289781	0.149	0.028	#######	Platelet	INF2
SPINT22	#######	2.122697	0.39	0.187	#######	Platelet	SPINT2
HLA-B2	#######	0.722596	0.914	0.925	#######	Platelet	HLA-B
FKBP1B	#######	1.073322	0.11	0.015	#######	Platelet	FKBP1B
CAPZB	#######	1.698532	0.637	0.614	#######	Platelet	CAPZB
MAGED2	#######	1.825468	0.273	0.096	#######	Platelet	MAGED2
ST3GAL3	#######	1.087285	0.11	0.016	#######	Platelet	ST3GAL3
HIST1H1C	#######	2.071966	0.294	0.111	#######	Platelet	HIST1H1C
ISCA11	#######	1.91857	0.298	0.115	#######	Platelet	ISCA1
UQCRH	#######	1.743433	0.631	0.61	#######	Platelet	UQCRH
CCDC92	#######	1.63938	0.208	0.058	#######	Platelet	CCDC92
MIR4435-1	#######	2.10008	0.32	0.133	#######	Platelet	MIR4435-1HG
SIAE	#######	1.103289	0.108	0.016	#######	Platelet	SIAE
PTGIR	#######	1.609465	0.174	0.042	#######	Platelet	PTGIR
UBL4A	#######	1.677149	0.231	0.071	#######	Platelet	UBL4A
R3HDM41	#######	2.155632	0.445	0.261	#######	Platelet	R3HDM4
ALDOA	#######	1.256007	0.729	0.792	#######	Platelet	ALDOA
PRR7	#######	1.219362	0.134	0.025	#######	Platelet	PRR7
ACTR3B	#######	1.102225	0.109	0.017	#######	Platelet	ACTR3B
SENCR	#######	1.19545	0.134	0.026	#######	Platelet	SENCR
P2RX1	#######	1.539549	0.225	0.069	#######	Platelet	P2RX1
HIST1H4H	#######	1.491922	0.113	0.019	#######	Platelet	HIST1H4H
CD27-AS1	#######	1.184216	0.14	0.03	#######	Platelet	CD27-AS1
HLA-C2	#######	0.776612	0.856	0.89	#######	Platelet	HLA-C
MLH3	#######	1.537762	0.176	0.047	#######	Platelet	MLH3
TMBIM1	#######	2.085725	0.325	0.151	#######	Platelet	TMBIM1
DAP	#######	1.959162	0.321	0.147	#######	Platelet	DAP
TPM3	#######	1.295205	0.682	0.763	#######	Platelet	TPM3
PTK2	#######	1.187255	0.131	0.027	#######	Platelet	PTK2
MAP4K2	#######	1.489345	0.2	0.062	#######	Platelet	MAP4K2
TALDO11	#######	1.304512	0.658	0.666	#######	Platelet	TALDO1
LEPROT	#######	2.004127	0.365	0.194	#######	Platelet	LEPROT
ETFA	#######	2.165896	0.368	0.201	#######	Platelet	ETFA
SPHK1	#######	1.117162	0.126	0.027	#######	Platelet	SPHK1
GCLM	#######	1.280559	0.17	0.048	#######	Platelet	GCLM
IFRD1	#######	1.810959	0.203	0.069	#######	Platelet	IFRD1
RAB4A	#######	1.930142	0.337	0.18	#######	Platelet	RAB4A
CMIP	#######	1.932315	0.36	0.204	#######	Platelet	CMIP
HMGB1	#######	1.257448	0.671	0.764	#######	Platelet	HMGB1
VDAC3	#######	1.987874	0.359	0.212	#######	Platelet	VDAC3
DYNLL11	#######	1.76657	0.539	0.494	#######	Platelet	DYNLL1

SOD21	#######	1.524027	0.502	0.383	#######	Platelet	SOD2
PTPLAD2	#######	1.846607	0.442	0.32	#######	Platelet	PTPLAD2
TMEM55A	#######	1.687916	0.252	0.11	#######	Platelet	TMEM55A
GAPDH2	#######	0.661465	0.871	0.927	#######	Platelet	GAPDH
MOB1B	#######	1.569813	0.195	0.071	#######	Platelet	MOB1B
DAPP1	#######	1.839684	0.348	0.204	#######	Platelet	DAPP1
HDGF	#######	1.87939	0.361	0.223	#######	Platelet	HDGF
TMEM185,	#######	1.170437	0.129	0.035	#######	Platelet	TMEM185A
AKIRIN2	#######	2.062075	0.336	0.197	#######	Platelet	AKIRIN2
WRB	#######	1.196009	0.153	0.049	#######	Platelet	WRB
CNST	#######	2.128714	0.242	0.11	#######	Platelet	CNST
XPNPEP11	#######	1.494512	0.206	0.084	#######	Platelet	XPNPEP1
FKBP1A1	#######	1.476621	0.574	0.57	#######	Platelet	FKBP1A
FKBP81	#######	1.722641	0.513	0.472	#######	Platelet	FKBP8
MAPRE21	#######	1.709691	0.265	0.133	#######	Platelet	MAPRE2
IFITM22	#######	1.05772	0.695	0.795	#######	Platelet	IFITM2
RAB32	#######	1.98762	0.414	0.303	#######	Platelet	RAB32
CD681	#######	1.551994	0.496	0.414	#######	Platelet	CD68
H1F0	#######	1.227641	0.162	0.057	#######	Platelet	H1F0
BIN21	#######	1.730232	0.49	0.442	#######	Platelet	BIN2
CALM13	#######	1.030554	0.665	0.765	#######	Platelet	CALM1
GSTO1	#######	1.988043	0.471	0.414	#######	Platelet	GSTO1
GLA	#######	1.402558	0.183	0.074	#######	Platelet	GLA
CMPK1	#######	1.851597	0.388	0.282	#######	Platelet	CMPK1
PTPN18	#######	2.063677	0.409	0.312	#######	Platelet	PTPN18
PADI41	#######	1.280383	0.262	0.13	#######	Platelet	PADI4
CAPZA2	#######	1.800682	0.437	0.362	#######	Platelet	CAPZA2
GRK5	#######	0.946376	0.114	0.034	#######	Platelet	GRK5
RHOC2	#######	1.569477	0.307	0.184	#######	Platelet	RHOC
TRAPPC1	#######	1.626951	0.521	0.524	#######	Platelet	TRAPPC1
CDIP1	#######	1.261451	0.167	0.067	#######	Platelet	CDIP1
IKBKG	#######	1.553465	0.248	0.131	#######	Platelet	IKBKG
TSC22D31	#######	1.629379	0.496	0.462	#######	Platelet	TSC22D3
C7orf73	#######	1.654623	0.471	0.423	#######	Platelet	C7orf73
CLIC4	#######	1.108095	0.142	0.052	#######	Platelet	CLIC4
BSCL2	#######	1.242062	0.157	0.063	6.22E-99	Platelet	BSCL2
RABGAP1L	#######	1.668278	0.322	0.21	1.28E-98	Platelet	RABGAP1L
WIPI1	1.02E-97	1.21327	0.155	0.063	3.35E-93	Platelet	WIPI1
PLEK2	7.34E-97	1.694518	0.503	0.507	2.40E-92	Platelet	PLEK
ANKRD28	1.34E-96	0.991716	0.101	0.031	4.39E-92	Platelet	ANKRD28
LDLRAP13	2.27E-96	1.482507	0.189	0.087	7.42E-92	Platelet	LDLRAP1
PTPRJ	6.35E-96	1.411846	0.209	0.104	2.08E-91	Platelet	PTPRJ
EMC31	3.20E-95	1.676485	0.299	0.192	1.05E-90	Platelet	EMC3
LINC00657	5.62E-95	1.874373	0.359	0.264	1.84E-90	Platelet	LINC00657
PDLIM7	2.58E-94	1.194739	0.199	0.095	8.43E-90	Platelet	PDLIM7
DERA	3.34E-94	1.455255	0.183	0.084	1.09E-89	Platelet	DERA
HPSE	7.26E-94	1.380495	0.198	0.095	2.38E-89	Platelet	HPSE
ABHD16A	3.20E-92	1.23789	0.161	0.069	1.05E-87	Platelet	ABHD16A
CD821	6.90E-91	1.422376	0.233	0.125	2.26E-86	Platelet	CD82
MORF4L1	5.15E-88	0.255729	0.237	0.529	1.69E-83	Platelet	MORF4L1
STRN4	1.28E-87	1.343861	0.207	0.106	4.18E-83	Platelet	STRN4
MGAT4B	3.49E-86	1.153734	0.144	0.059	1.14E-81	Platelet	MGAT4B
MTHFD2L	3.65E-86	1.192267	0.134	0.053	1.19E-81	Platelet	MTHFD2L
ARHGAP2:	2.97E-85	1.110268	0.141	0.058	9.73E-81	Platelet	ARHGAP21
CORO1C1	6.01E-83	1.526625	0.239	0.139	1.97E-78	Platelet	CORO1C
SEPT112	2.08E-82	1.321583	0.15	0.065	6.82E-78	Platelet	11-Sep
ATP2C1	6.82E-82	1.004657	0.133	0.053	2.23E-77	Platelet	ATP2C1
NUTF2	8.13E-82	1.895612	0.302	0.21	2.66E-77	Platelet	NUTF2

TST	1.47E-81	1.241706	0.175	0.084	4.82E-77	Platelet	TST
YPEL51	3.82E-81	1.71368	0.305	0.211	1.25E-76	Platelet	YPEL5
GSTM4	7.00E-80	1.14934	0.137	0.057	2.29E-75	Platelet	GSTM4
DAAM11	1.21E-79	1.128786	0.139	0.059	3.96E-75	Platelet	DAAM1
AIG1	3.18E-79	0.960212	0.126	0.05	1.04E-74	Platelet	AIG1
HMG20B	1.04E-73	1.366532	0.213	0.121	3.42E-69	Platelet	HMG20B
HIST2H2A	1.09E-72	2.019733	0.215	0.123	3.55E-68	Platelet	HIST2H2AA3
SH3BGRL1	2.31E-72	1.461711	0.5	0.535	7.55E-68	Platelet	SH3BGRL
CDK5RAP2	4.05E-72	1.129177	0.14	0.062	1.32E-67	Platelet	CDK5RAP2
ICAM23	4.18E-70	1.803658	0.322	0.247	1.37E-65	Platelet	ICAM2
C12orf76	9.13E-70	1.597291	0.181	0.096	2.99E-65	Platelet	C12orf76
OSTF11	9.70E-70	0.258852	0.181	0.396	3.18E-65	Platelet	OSTF1
TMEM189	2.16E-68	1.124273	0.151	0.072	7.07E-64	Platelet	TMEM189
PIP4K2A1	3.88E-68	1.511108	0.238	0.149	1.27E-63	Platelet	PIP4K2A
CAMTA1	2.16E-67	1.590521	0.288	0.204	7.08E-63	Platelet	CAMTA1
TSPAN21	1.98E-66	0.769828	0.103	0.039	6.50E-62	Platelet	TSPAN2
CAP11	8.48E-66	1.200594	0.551	0.652	2.77E-61	Platelet	CAP1
AMFR	8.77E-66	1.354241	0.177	0.094	2.87E-61	Platelet	AMFR
LYPLAL1	7.56E-65	1.265214	0.164	0.084	2.48E-60	Platelet	LYPLAL1
GDI1	1.83E-64	1.33081	0.235	0.148	5.99E-60	Platelet	GDI1
P4HB2	4.62E-62	0.38292	0.175	0.378	1.51E-57	Platelet	P4HB
ARPC52	7.87E-62	1.036578	0.575	0.706	2.58E-57	Platelet	ARPC5
RBM383	3.33E-61	1.539679	0.217	0.134	1.09E-56	Platelet	RBM38
WDR83OS	4.26E-60	0.349627	0.28	0.551	1.39E-55	Platelet	WDR83OS
PRMT25	1.94E-59	0.305366	0.2	0.407	6.36E-55	Platelet	PRMT2
PARK71	5.02E-59	1.436826	0.477	0.519	1.64E-54	Platelet	PARK7
CTSW3	3.29E-58	0.694137	0.296	0.187	1.08E-53	Platelet	CTSW
RIOK31	1.26E-57	1.867146	0.344	0.289	4.13E-53	Platelet	RIOK3
RAB8A	4.28E-57	1.558099	0.331	0.272	1.40E-52	Platelet	RAB8A
SNX33	4.75E-57	1.43347	0.472	0.51	1.56E-52	Platelet	SNX3
TMBIM61	1.02E-56	0.285557	0.341	0.646	3.33E-52	Platelet	TMBIM6
ANXA112	3.29E-56	0.321373	0.221	0.444	1.08E-51	Platelet	ANXA11
KCNA32	4.21E-56	1.074142	0.112	0.049	1.38E-51	Platelet	KCNA3
TMEM50A	4.22E-56	1.504773	0.437	0.444	1.38E-51	Platelet	TMEM50A
FCER1G2	2.38E-55	0.68632	0.665	0.704	7.80E-51	Platelet	FCER1G
CYB5R1	8.01E-55	1.270382	0.166	0.092	2.62E-50	Platelet	CYB5R1
PYCR2	1.41E-54	0.920547	0.115	0.053	4.63E-50	Platelet	PYCR2
C1orf431	4.37E-54	0.277074	0.128	0.287	1.43E-49	Platelet	C1orf43
TAF101	5.22E-54	0.395701	0.247	0.485	1.71E-49	Platelet	TAF10
PPP3R1	4.06E-53	1.361903	0.21	0.133	1.33E-48	Platelet	PPP3R1
CENPT	1.03E-52	0.94421	0.121	0.057	3.37E-48	Platelet	CENPT
MEPCE	1.75E-52	1.046743	0.115	0.053	5.72E-48	Platelet	MEPCE
TSC22D42	1.11E-50	0.253992	0.107	0.245	3.64E-46	Platelet	TSC22D4
ARF52	3.11E-50	0.301572	0.278	0.523	1.02E-45	Platelet	ARF5
UBE2V12	5.96E-50	0.317486	0.141	0.299	1.95E-45	Platelet	UBE2V1
VAMP7	7.95E-49	1.182996	0.162	0.093	2.60E-44	Platelet	VAMP7
AMD1	9.18E-47	1.610851	0.257	0.193	3.01E-42	Platelet	AMD1
AP2S11	2.63E-46	0.263671	0.317	0.576	8.62E-42	Platelet	AP2S1
EMD	1.10E-45	1.48471	0.28	0.222	3.60E-41	Platelet	EMD
UBE2E31	5.09E-45	1.413337	0.272	0.21	1.67E-40	Platelet	UBE2E3
ELF1	1.04E-44	0.870722	0.186	0.372	3.39E-40	Platelet	ELF1
GLRX52	4.85E-44	0.305931	0.082	0.196	1.59E-39	Platelet	GLRX5
LINC0015	5.63E-44	1.5373	0.233	0.166	1.84E-39	Platelet	LINC00152
SNRPN5	4.18E-43	1.201597	0.16	0.096	1.37E-38	Platelet	SNRPN
TACC12	5.10E-43	0.264411	0.143	0.287	1.67E-38	Platelet	TACC1
PPM1A	2.62E-42	1.153316	0.166	0.101	8.57E-38	Platelet	PPM1A
STAT3	3.56E-42	0.329393	0.158	0.311	1.17E-37	Platelet	STAT3
PCBP13	4.35E-42	0.284416	0.379	0.676	1.42E-37	Platelet	PCBP1

RBBP6	5.40E-42	1.738364	0.304	0.259	1.77E-37	Platelet	RBBP6
CNN26	5.63E-42	0.41573	0.212	0.403	1.84E-37	Platelet	CNN2
ARMCX61	2.15E-41	1.053053	0.129	0.071	7.05E-37	Platelet	ARMCX6
VPS28	5.27E-41	0.463714	0.252	0.466	1.72E-36	Platelet	VPS28
ATP6AP1	6.09E-41	0.304359	0.121	0.252	1.99E-36	Platelet	ATP6AP1
YWHAQ2	8.24E-41	1.442849	0.317	0.275	2.70E-36	Platelet	YWHAQ
ASNA11	1.00E-40	0.250239	0.078	0.185	3.28E-36	Platelet	ASNA1
CTDSP2	1.02E-40	0.280061	0.084	0.194	3.33E-36	Platelet	CTDSP2
MYL12B4	1.22E-40	0.262197	0.368	0.663	3.98E-36	Platelet	MYL12B
USP12	2.45E-40	1.016003	0.109	0.055	8.02E-36	Platelet	USP12
DSTN2	3.21E-40	1.500349	0.305	0.262	1.05E-35	Platelet	DSTN
SWI5	3.22E-40	0.951169	0.124	0.066	1.06E-35	Platelet	SWI5
ATP6V0C1	1.78E-39	1.184945	0.485	0.555	5.84E-35	Platelet	ATP6V0C
RASGRP2	2.12E-39	1.813668	0.351	0.333	6.95E-35	Platelet	RASGRP2
STAU1	1.59E-38	0.280101	0.096	0.207	5.21E-34	Platelet	STAU1
GRK61	2.87E-38	0.329072	0.126	0.255	9.41E-34	Platelet	GRK6
SPPL3	3.22E-38	1.250766	0.181	0.119	1.06E-33	Platelet	SPPL3
PRKAR1A1	3.82E-38	0.367832	0.203	0.375	1.25E-33	Platelet	PRKAR1A
CUX12	4.82E-38	0.257721	0.146	0.28	1.58E-33	Platelet	CUX1
LSMD11	8.52E-38	0.454622	0.244	0.45	2.79E-33	Platelet	LSMD1
RAB13	2.18E-37	0.92405	0.124	0.069	7.14E-33	Platelet	RAB13
SF3A21	4.17E-37	0.32133	0.088	0.194	1.37E-32	Platelet	SF3A2
TRAPPC51	2.26E-36	0.42243	0.237	0.425	7.41E-32	Platelet	TRAPPC5
CHD92	3.18E-36	0.345437	0.087	0.189	1.04E-31	Platelet	CHD9
PYGB	6.85E-36	0.909604	0.117	0.064	2.24E-31	Platelet	PYGB
PNP	6.90E-36	1.340732	0.221	0.165	2.26E-31	Platelet	PNP
HTATIP2	2.68E-35	1.383505	0.219	0.162	8.76E-31	Platelet	HTATIP2
SCP22	2.79E-35	0.50967	0.143	0.278	9.15E-31	Platelet	SCP2
CDC42SE2	5.41E-35	0.464979	0.118	0.239	1.77E-30	Platelet	CDC42SE2
IFITM31	1.28E-34	0.773395	0.483	0.468	4.17E-30	Platelet	IFITM3
UXS1	1.61E-34	1.093119	0.15	0.094	5.27E-30	Platelet	UXS1
NENF1	2.87E-34	1.446413	0.284	0.242	9.39E-30	Platelet	NENF
RTN31	7.45E-34	1.251511	0.365	0.349	2.44E-29	Platelet	RTN3
NAP1L44	8.76E-34	0.474618	0.146	0.278	2.87E-29	Platelet	NAP1L4
LYL1	5.32E-33	1.619807	0.244	0.195	1.74E-28	Platelet	LYL1
PRDX51	1.08E-32	0.487857	0.263	0.469	3.52E-28	Platelet	PRDX5
NFE21	2.98E-32	1.492784	0.257	0.208	9.75E-28	Platelet	NFE2
MTFR1L	3.04E-32	0.967836	0.127	0.075	9.95E-28	Platelet	MTFR1L
KLF31	4.52E-32	0.495276	0.145	0.275	1.48E-27	Platelet	KLF3
DYNLRB1	9.31E-32	1.46936	0.341	0.329	3.05E-27	Platelet	DYNLRB1
RASA33	1.48E-31	1.143189	0.201	0.145	4.83E-27	Platelet	RASA3
PPP1R15A	7.29E-31	1.273405	0.214	0.161	2.39E-26	Platelet	PPP1R15A
ABHD17A:	1.40E-30	0.309727	0.134	0.249	4.59E-26	Platelet	ABHD17A
BICD21	1.88E-30	0.293666	0.089	0.182	6.17E-26	Platelet	BICD2
LMAN14	2.93E-30	0.272387	0.073	0.158	9.60E-26	Platelet	LMAN1
ARL8B	3.40E-30	0.356197	0.107	0.209	1.11E-25	Platelet	ARL8B
SLC50A11	3.41E-30	0.93144	0.135	0.083	1.12E-25	Platelet	SLC50A1
PPP6R11	5.14E-30	1.088328	0.143	0.092	1.68E-25	Platelet	PPP6R1
YIPF3	6.96E-30	0.36937	0.108	0.212	2.28E-25	Platelet	YIPF3
ITFG1	9.88E-30	1.152593	0.176	0.123	3.23E-25	Platelet	ITFG1
PPP1R182	1.02E-29	0.524592	0.237	0.416	3.35E-25	Platelet	PPP1R18
SIAH21	1.81E-29	1.401985	0.195	0.143	5.93E-25	Platelet	SIAH2
MTRF1L	2.97E-29	1.025163	0.136	0.085	9.71E-25	Platelet	MTRF1L
SZRD1	3.82E-29	0.33264	0.088	0.179	1.25E-24	Platelet	SZRD1
RALY1	6.28E-29	0.508858	0.188	0.332	2.06E-24	Platelet	RALY
INSIG11	6.30E-29	1.216314	0.167	0.114	2.06E-24	Platelet	INSIG1
RGCC4	7.00E-29	1.064824	0.142	0.091	2.29E-24	Platelet	RGCC
ABI11	7.82E-29	0.328079	0.101	0.197	2.56E-24	Platelet	ABI1

POLR2G1	9.25E-29	0.469611	0.144	0.264	3.03E-24	Platelet	POLR2G
CCDC88C2	3.05E-28	0.412872	0.084	0.171	9.99E-24	Platelet	CCDC88C
FAM89B1	2.31E-27	0.275171	0.081	0.165	7.57E-23	Platelet	FAM89B
SLC2A31	2.36E-27	1.043996	0.263	0.216	7.72E-23	Platelet	SLC2A3
LINC01003	7.70E-27	0.94059	0.11	0.065	2.52E-22	Platelet	LINC01003
ZFAND6	1.45E-26	0.318326	0.097	0.186	4.74E-22	Platelet	ZFAND6
CAB39	2.11E-26	0.363651	0.104	0.197	6.92E-22	Platelet	CAB39
MTHFD21	5.34E-26	0.357377	0.077	0.157	1.75E-21	Platelet	MTHFD2
CRBN1	9.48E-26	0.490883	0.102	0.195	3.10E-21	Platelet	CRBN
LAMP11	1.40E-25	0.469148	0.168	0.292	4.59E-21	Platelet	LAMP1
UBA7	1.81E-25	1.007506	0.153	0.104	5.93E-21	Platelet	UBA7
CHMP3	4.72E-25	0.459015	0.116	0.213	1.54E-20	Platelet	CHMP3
WIPF11	4.98E-25	1.298404	0.368	0.382	1.63E-20	Platelet	WIPF1
PTPN121	5.99E-25	1.299139	0.229	0.187	1.96E-20	Platelet	PTPN12
WRNIP1	1.82E-24	1.003277	0.133	0.087	5.97E-20	Platelet	WRNIP1
HBP1	1.98E-24	0.334041	0.083	0.161	6.48E-20	Platelet	HBP1
CCNG1	2.11E-24	1.325867	0.187	0.141	6.90E-20	Platelet	CCNG1
UGP21	2.15E-24	0.426977	0.128	0.229	7.03E-20	Platelet	UGP2
MAF11	2.60E-24	0.505845	0.132	0.236	8.51E-20	Platelet	MAF1
NME41	3.62E-24	1.099501	0.149	0.103	1.19E-19	Platelet	NME4
DNAJB6	5.74E-24	1.54536	0.276	0.252	1.88E-19	Platelet	DNAJB6
ZCCHC171	6.42E-24	1.153981	0.175	0.129	2.10E-19	Platelet	ZCCHC17
TBPL1	7.54E-24	1.170223	0.143	0.098	2.47E-19	Platelet	TBPL1
ARF3	1.08E-23	1.307076	0.257	0.226	3.52E-19	Platelet	ARF3
CRYL1	1.32E-23	0.982917	0.137	0.092	4.31E-19	Platelet	CRYL1
RHEB	1.95E-23	1.243605	0.214	0.174	6.38E-19	Platelet	RHEB
SECISBP2	2.58E-23	0.328216	0.091	0.172	8.44E-19	Platelet	SECISBP2
DAD14	2.87E-23	0.72382	0.245	0.42	9.41E-19	Platelet	DAD1
RTN41	4.72E-23	0.545464	0.266	0.451	1.54E-18	Platelet	RTN4
LGALS8	4.91E-23	0.309072	0.079	0.154	1.61E-18	Platelet	LGALS8
SSR41	7.74E-23	1.135111	0.48	0.606	2.53E-18	Platelet	SSR4
PHF20L11	7.99E-23	0.44703	0.145	0.25	2.62E-18	Platelet	PHF20L1
CREG11	9.48E-23	0.477463	0.125	0.222	3.10E-18	Platelet	CREG1
FAM32A	1.23E-22	0.494098	0.12	0.213	4.03E-18	Platelet	FAM32A
CDC421	1.70E-22	0.404699	0.385	0.645	5.57E-18	Platelet	CDC42
CDC37	2.78E-22	0.652172	0.245	0.412	9.09E-18	Platelet	CDC37
SSBP21	3.35E-22	0.836593	0.12	0.077	1.10E-17	Platelet	SSBP2
UFD1L	5.84E-22	0.423842	0.11	0.196	1.91E-17	Platelet	UFD1L
MLEC2	6.62E-22	0.51436	0.143	0.248	2.17E-17	Platelet	MLEC
LPP	7.20E-22	0.313506	0.073	0.142	2.36E-17	Platelet	LPP
HIPK21	9.73E-22	0.311323	0.079	0.151	3.19E-17	Platelet	HIPK2
DIAPH11	1.06E-21	1.366321	0.317	0.314	3.46E-17	Platelet	DIAPH1
TMEM219	1.72E-21	1.183039	0.404	0.452	5.64E-17	Platelet	TMEM219
UBE2H1	1.95E-21	1.238071	0.191	0.15	6.40E-17	Platelet	UBE2H
MAFG	2.17E-21	0.834335	0.127	0.085	7.11E-17	Platelet	MAFG
PRKCB2	3.02E-21	1.217723	0.376	0.406	9.88E-17	Platelet	PRKCB
SUSD33	5.31E-21	0.84302	0.111	0.071	1.74E-16	Platelet	SUSD3
TANGO2	1.71E-20	0.863577	0.135	0.093	5.59E-16	Platelet	TANGO2
ANKRD10	1.73E-20	0.258699	0.068	0.133	5.67E-16	Platelet	ANKRD10
ARMCX31	2.39E-20	1.033257	0.144	0.103	7.82E-16	Platelet	ARMCX3
PIK3CB	3.34E-20	0.979006	0.134	0.093	1.09E-15	Platelet	PIK3CB
CTD-2336	3.65E-20	0.984069	0.149	0.108	1.19E-15	Platelet	CTD-2336O2.1
LRBA2	4.43E-20	0.92851	0.117	0.077	1.45E-15	Platelet	LRBA
TPP2	5.32E-20	0.280368	0.082	0.151	1.74E-15	Platelet	TPP2
UQCR11.1	7.99E-20	0.294364	0.471	0.778	2.62E-15	Platelet	UQCR11.1
ADD33	8.98E-20	0.599554	0.217	0.358	2.94E-15	Platelet	ADD3
TPP11	1.10E-19	0.522775	0.216	0.351	3.60E-15	Platelet	TPP1
VIM-AS1	2.56E-19	0.974826	0.121	0.082	8.39E-15	Platelet	VIM-AS1

VGLL4	3.47E-19	0.930669	0.131	0.091	1.14E-14	Platelet	VGLL4
DSE1	7.52E-19	1.194516	0.165	0.126	2.46E-14	Platelet	DSE
SLC44A1	7.94E-19	0.867638	0.131	0.091	2.60E-14	Platelet	SLC44A1
UPF3A2	8.59E-19	0.452867	0.077	0.143	2.81E-14	Platelet	UPF3A
FCGR2A1	1.00E-18	1.146436	0.272	0.25	3.27E-14	Platelet	FCGR2A
BCAP31	2.01E-18	1.272036	0.387	0.441	6.59E-14	Platelet	BCAP31
KIFAP31	2.95E-18	1.057493	0.12	0.082	9.65E-14	Platelet	KIFAP3
ATP6V0E1	7.89E-18	1.018576	0.488	0.626	2.58E-13	Platelet	ATP6V0E1
RN7SL11	8.83E-18	0.334479	0.087	0.154	2.89E-13	Platelet	RN7SL1
SKAP22	9.55E-18	0.531445	0.22	0.349	3.13E-13	Platelet	SKAP2
MT-ATP88	9.99E-18	0.415721	0.671	0.744	3.27E-13	Platelet	MT-ATP8
PACSIN2	1.59E-17	0.965824	0.154	0.116	5.20E-13	Platelet	PACSIN2
RIT1	1.94E-17	0.938905	0.149	0.11	6.36E-13	Platelet	RIT1
DAZAP2	4.15E-17	0.525692	0.38	0.632	1.36E-12	Platelet	DAZAP2
SLBP	4.24E-17	0.487044	0.104	0.178	1.39E-12	Platelet	SLBP
COTL13	4.42E-17	0.422203	0.656	0.732	1.45E-12	Platelet	COTL1
TMED31	6.54E-17	0.329671	0.07	0.129	2.14E-12	Platelet	TMED3
RAB5B	7.78E-17	0.267794	0.069	0.126	2.55E-12	Platelet	RAB5B
FOXO31	9.36E-17	0.257141	0.079	0.139	3.06E-12	Platelet	FOXO3
C18orf321	1.17E-16	0.547777	0.131	0.215	3.82E-12	Platelet	C18orf32
ANP32B3	1.49E-16	0.64447	0.304	0.5	4.88E-12	Platelet	ANP32B
ARL6IP51	1.76E-16	1.06923	0.461	0.585	5.77E-12	Platelet	ARL6IP5
RAB311	1.78E-16	1.1093	0.379	0.408	5.82E-12	Platelet	RAB31
BNIP3L2	1.83E-16	0.806826	0.226	0.363	5.99E-12	Platelet	BNIP3L
PDLIM51	1.87E-16	0.325706	0.093	0.158	6.13E-12	Platelet	PDLIM5
Sep-67	2.14E-16	1.272065	0.308	0.314	7.01E-12	Platelet	6-Sep
AP2M1	3.55E-16	1.303891	0.35	0.383	1.16E-11	Platelet	AP2M1
EPB41L31	6.51E-16	0.322818	0.052	0.101	2.13E-11	Platelet	EPB41L3
VPS41	8.03E-16	0.282389	0.066	0.12	2.63E-11	Platelet	VPS41
PACS11	8.43E-16	0.283655	0.071	0.127	2.76E-11	Platelet	PACS1
ITM2A5	1.02E-15	0.285046	0.066	0.119	3.35E-11	Platelet	ITM2A
H2AFJ	1.89E-15	1.457574	0.289	0.287	6.18E-11	Platelet	H2AFJ
CERS2	2.62E-15	0.98551	0.157	0.123	8.59E-11	Platelet	CERS2
SKP13	3.74E-15	0.609233	0.356	0.592	1.22E-10	Platelet	SKP1
TIA1	5.00E-15	0.360097	0.081	0.139	1.64E-10	Platelet	TIA1
SMS	5.80E-15	0.568542	0.135	0.216	1.90E-10	Platelet	SMS
PFN14	6.28E-15	0.30028	0.8	0.922	2.06E-10	Platelet	PFN1
ABTB11	6.91E-15	0.453788	0.158	0.247	2.26E-10	Platelet	ABTB1
VTI1B	7.03E-15	0.598332	0.188	0.296	2.30E-10	Platelet	VTI1B
AP2B1	7.25E-15	0.526294	0.123	0.198	2.37E-10	Platelet	AP2B1
C11orf211	1.03E-14	0.27789	0.069	0.121	3.36E-10	Platelet	C11orf21
NCOA73	1.57E-14	0.31805	0.076	0.131	5.14E-10	Platelet	NCOA7
ACP13	1.62E-14	0.646301	0.157	0.248	5.30E-10	Platelet	ACP1
MAP4K51	1.75E-14	0.921422	0.108	0.076	5.72E-10	Platelet	MAP4K5
SHARPIN	2.90E-14	0.430035	0.086	0.145	9.48E-10	Platelet	SHARPIN
FAM104A1	2.91E-14	0.260303	0.058	0.104	9.54E-10	Platelet	FAM104A
SPNS1	5.10E-14	1.196996	0.168	0.138	1.67E-09	Platelet	SPNS1
FRMD4B	6.37E-14	1.132345	0.136	0.104	2.08E-09	Platelet	FRMD4B
MPC22	8.53E-14	0.389893	0.099	0.162	2.79E-09	Platelet	MPC2
PEF11	1.07E-13	0.321497	0.06	0.108	3.49E-09	Platelet	PEF1
GLUL2	1.24E-13	1.273693	0.292	0.295	4.06E-09	Platelet	GLUL
RNF53	2.42E-13	0.448987	0.095	0.155	7.91E-09	Platelet	RNF5
HPCAL1	3.86E-13	1.100005	0.22	0.2	1.26E-08	Platelet	HPCAL1
VAPA	4.91E-13	0.812695	0.248	0.39	1.61E-08	Platelet	VAPA
UROD	5.00E-13	0.265479	0.074	0.125	1.64E-08	Platelet	UROD
NUDT41	5.28E-13	0.4755	0.075	0.126	1.73E-08	Platelet	NUDT4
SNX92	6.16E-13	0.833264	0.11	0.08	2.02E-08	Platelet	SNX9
DOK1	1.30E-12	0.330775	0.076	0.127	4.27E-08	Platelet	DOK1

NEK7	1.37E-12	0.277824	0.067	0.113	4.50E-08	Platelet	NEK7
SNAP292	1.66E-12	0.487953	0.105	0.167	5.44E-08	Platelet	SNAP29
PHTF21	1.67E-12	0.830685	0.112	0.082	5.46E-08	Platelet	PHTF2
BRE	1.92E-12	0.334737	0.071	0.12	6.28E-08	Platelet	BRE
CD361	2.79E-12	1.011198	0.352	0.376	9.12E-08	Platelet	CD36
EHD1	3.04E-12	0.842893	0.157	0.127	9.95E-08	Platelet	EHD1
RAB6A	4.63E-12	0.564094	0.133	0.205	1.52E-07	Platelet	RAB6A
MKRN11	4.66E-12	1.275218	0.257	0.254	1.52E-07	Platelet	MKRN1
TPGS2	7.33E-12	0.343365	0.061	0.105	2.40E-07	Platelet	TPGS2
ARL6IP13	8.15E-12	0.782939	0.195	0.301	2.67E-07	Platelet	ARL6IP1
ASAH12	8.17E-12	1.278654	0.376	0.431	2.68E-07	Platelet	ASAH1
GTF3C6	9.70E-12	1.235808	0.238	0.23	3.18E-07	Platelet	GTF3C6
FBXW5	1.32E-11	0.673991	0.164	0.252	4.31E-07	Platelet	FBXW5
MTERFD2	1.61E-11	0.400757	0.067	0.112	5.28E-07	Platelet	MTERFD2
UBC4	1.71E-11	0.399662	0.478	0.783	5.60E-07	Platelet	UBC
MINK1	2.08E-11	0.827828	0.117	0.089	6.80E-07	Platelet	MINK1
BTK3	7.03E-11	0.488806	0.105	0.162	2.30E-06	Platelet	BTK
BRD3	8.76E-11	0.424508	0.094	0.146	2.87E-06	Platelet	BRD3
TUBB8	8.82E-11	0.762215	0.257	0.394	2.89E-06	Platelet	TUBB
SUSD11	2.38E-10	0.777542	0.122	0.095	7.81E-06	Platelet	SUSD1
BSG2	5.09E-10	0.823734	0.234	0.356	1.67E-05	Platelet	BSG
CMC2	5.84E-10	0.703893	0.087	0.136	1.91E-05	Platelet	CMC2
ANXA7	6.19E-10	0.636003	0.155	0.23	2.03E-05	Platelet	ANXA7
HIGD1A1	1.32E-09	1.026	0.193	0.177	4.32E-05	Platelet	HIGD1A
ABLIM15	2.26E-09	0.826096	0.118	0.093	7.40E-05	Platelet	ABLIM1
UBXN111	2.58E-09	0.630918	0.155	0.228	8.43E-05	Platelet	UBXN11
KCTD20	2.76E-09	1.100453	0.179	0.16	9.03E-05	Platelet	KCTD20
UBE2F1	2.87E-09	0.46486	0.09	0.136	9.40E-05	Platelet	UBE2F
CCS1	3.45E-09	0.530844	0.108	0.161	0.000113	Platelet	CCS
ARPC41	3.75E-09	0.574763	0.254	0.372	0.000123	Platelet	ARPC4
FYB1	3.77E-09	0.34335	0.468	0.722	0.000123	Platelet	FYB
NRD11	5.15E-09	0.689312	0.185	0.27	0.000169	Platelet	NRD1
HSPB14	6.65E-09	1.141606	0.226	0.219	0.000218	Platelet	HSPB1
TBC1D20	8.79E-09	0.876967	0.124	0.102	0.000288	Platelet	TBC1D20
RNF1151	1.25E-08	0.504836	0.093	0.139	0.00041	Platelet	RNF115
C7orf501	1.28E-08	0.559597	0.109	0.161	0.000419	Platelet	C7orf50
ZFAND2B1	1.36E-08	0.501432	0.092	0.138	0.000444	Platelet	ZFAND2B
ARMC8	1.40E-08	0.366417	0.073	0.112	0.000459	Platelet	ARMC8
FAM177A1	1.57E-08	0.502225	0.105	0.154	0.000515	Platelet	FAM177A1
STK40	1.72E-08	0.971015	0.159	0.139	0.000563	Platelet	STK40
PCNP	1.88E-08	1.185378	0.219	0.213	0.000615	Platelet	PCNP
GPI1	1.93E-08	0.769937	0.18	0.266	0.000631	Platelet	GPI
TSPAN321	1.97E-08	0.317891	0.081	0.121	0.000645	Platelet	TSPAN32
TCEAL81	2.01E-08	0.353175	0.069	0.107	0.000657	Platelet	TCEAL8
GNAQ1	2.27E-08	0.489029	0.134	0.193	0.000742	Platelet	GNAQ
MAP1LC3B	2.45E-08	1.195723	0.328	0.372	0.000802	Platelet	MAP1LC3B
RAB11B	3.32E-08	1.160176	0.24	0.244	0.001088	Platelet	RAB11B
DOK22	5.85E-08	1.186211	0.323	0.368	0.001914	Platelet	DOK2
EIF11	6.96E-08	0.48058	0.714	0.892	0.00228	Platelet	EIF1
CHST123	6.98E-08	0.266983	0.074	0.11	0.002285	Platelet	CHST12
ATP6V1E1	7.08E-08	0.607519	0.146	0.21	0.002319	Platelet	ATP6V1E1
DNAJC42	8.18E-08	0.701257	0.168	0.242	0.002678	Platelet	DNAJC4
PGD1	8.32E-08	0.408984	0.326	0.462	0.002724	Platelet	PGD
MIF4GD2	8.65E-08	0.588555	0.112	0.162	0.002831	Platelet	MIF4GD
LRRFIP2	1.12E-07	0.501585	0.118	0.169	0.003654	Platelet	LRRFIP2
ARPP19	1.41E-07	0.40845	0.092	0.134	0.004602	Platelet	ARPP19
STXBP22	1.96E-07	0.756528	0.435	0.522	0.006418	Platelet	STXBP2
SDHAF2	2.05E-07	0.382466	0.067	0.101	0.006723	Platelet	SDHAF2

BTN3A22	2.20E-07	0.594282	0.144	0.204	0.007198	Platelet	BTN3A2
BNIP2	2.26E-07	0.850707	0.23	0.336	0.007394	Platelet	BNIP2
DCTN2	4.90E-07	0.640639	0.157	0.223	0.016044	Platelet	DCTN2
TOLLIP	5.30E-07	0.51092	0.113	0.159	0.017339	Platelet	TOLLIP
RC3H2	6.39E-07	0.436559	0.072	0.106	0.020908	Platelet	RC3H2
ZFAND3	7.40E-07	1.018595	0.174	0.16	0.024229	Platelet	ZFAND3
GOLGA2	8.41E-07	0.32893	0.083	0.119	0.027542	Platelet	GOLGA2
REXO24	8.78E-07	0.392964	0.08	0.115	0.028733	Platelet	REXO2
ARL2	1.01E-06	0.529548	0.091	0.13	0.033178	Platelet	ARL2
MAP3K5	1.04E-06	0.335036	0.084	0.119	0.033912	Platelet	MAP3K5
ARRB11	1.56E-06	0.544528	0.118	0.165	0.05118	Platelet	ARRB1
SUMO31	1.89E-06	0.750084	0.187	0.263	0.061724	Platelet	SUMO3
6-Mar	4.55E-06	0.47349	0.084	0.119	0.148848	Platelet	6-Mar
BCL7B	5.60E-06	0.39204	0.095	0.132	0.183389	Platelet	BCL7B
EPB415	6.73E-06	0.777218	0.154	0.215	0.220432	Platelet	EPB41
USP39	6.91E-06	0.467532	0.071	0.102	0.226254	Platelet	USP39
CD632	7.83E-06	0.558494	0.33	0.475	0.25618	Platelet	CD63
GSAP1	9.25E-06	0.44874	0.101	0.139	0.302696	Platelet	GSAP
SIRT21	9.82E-06	0.47582	0.091	0.127	0.321335	Platelet	SIRT2
ORAI21	1.14E-05	0.882412	0.139	0.124	0.374087	Platelet	ORAI2
EIF1B1	1.15E-05	0.893253	0.212	0.301	0.377552	Platelet	EIF1B
GABARAPI	1.28E-05	1.070167	0.364	0.451	0.419643	Platelet	GABARAPL2
HMGN18	1.34E-05	1.151086	0.314	0.365	0.438078	Platelet	HMGN1
PHKB	1.34E-05	0.86166	0.152	0.14	0.438318	Platelet	PHKB
PCMTD12	1.64E-05	0.734795	0.151	0.208	0.53546	Platelet	PCMTD1
PCMT1	1.82E-05	1.075666	0.237	0.247	0.595264	Platelet	PCMT1
TERF2IP3	2.27E-05	0.812711	0.234	0.334	0.741546	Platelet	TERF2IP
NUDT21	4.85E-05	0.625962	0.118	0.16	1	Platelet	NUDT21
PTP4A21	6.82E-05	0.960295	0.452	0.622	1	Platelet	PTP4A2
ADI11	7.09E-05	1.056545	0.218	0.226	1	Platelet	ADI1
ORAI13	8.49E-05	0.740512	0.177	0.241	1	Platelet	ORAI1
PYGL1	0.000103	0.805534	0.198	0.194	1	Platelet	PYGL
TMOD3	0.000111	0.836477	0.249	0.35	1	Platelet	TMOD3
YBX32	0.000117	1.033742	0.267	0.293	1	Platelet	YBX3
G3BP2	0.000118	0.790304	0.175	0.238	1	Platelet	G3BP2
UBAP1	0.000131	0.419507	0.088	0.117	1	Platelet	UBAP1
ADIPOR2	0.000136	0.735436	0.111	0.098	1	Platelet	ADIPOR2
CTNNBIP1	0.000146	0.492567	0.099	0.132	1	Platelet	CTNNBIP1
YWHAE	0.000153	1.064547	0.329	0.396	1	Platelet	YWHAE
Sep-72	0.000178	0.80599	0.34	0.503	1	Platelet	7-Sep
RBX11	0.000189	1.295081	0.357	0.451	1	Platelet	RBX1
CD84	0.000196	0.427088	0.092	0.122	1	Platelet	CD84
NUCB11	0.000229	1.17543	0.263	0.294	1	Platelet	NUCB1
PNKD	0.000234	1.092218	0.225	0.237	1	Platelet	PNKD
TADA31	0.000249	1.044953	0.202	0.208	1	Platelet	TADA3
COQ4	0.000276	0.488396	0.079	0.106	1	Platelet	COQ4
RABAC1	0.00029	0.863395	0.301	0.435	1	Platelet	RABAC1
CMTM61	0.000298	1.087704	0.317	0.379	1	Platelet	CMTM6
CPQ	0.000334	0.651529	0.109	0.144	1	Platelet	CPQ
CALCOCO	0.000341	0.455564	0.091	0.12	1	Platelet	CALCOCO1
EIF4E1	0.000381	0.595113	0.106	0.14	1	Platelet	EIF4E
PSTPIP2	0.000407	1.011831	0.174	0.171	1	Platelet	PSTPIP2
MCTP1	0.000463	0.770515	0.146	0.138	1	Platelet	MCTP1
NUTM2A-	0.000464	1.104258	0.145	0.14	1	Platelet	NUTM2A-AS1
UBAC22	0.000508	0.993545	0.179	0.18	1	Platelet	UBAC2
RNF101	0.000518	0.898868	0.182	0.182	1	Platelet	RNF10
MMP24-A	0.000521	1.023349	0.29	0.332	1	Platelet	MMP24-AS1
BLOC1S6	0.000568	0.667308	0.155	0.204	1	Platelet	BLOC1S6

SERINC31	0.000581	0.84077	0.186	0.252	1	Platelet	SERINC3
AKR7A21	0.000677	1.115876	0.174	0.175	1	Platelet	AKR7A2
SRSF6	0.000717	0.598592	0.106	0.139	1	Platelet	SRSF6
RAC11	0.000787	0.460712	0.457	0.678	1	Platelet	RAC1
SEC14L1	0.000837	1.292965	0.232	0.251	1	Platelet	SEC14L1
RHOF5	0.000874	0.635804	0.128	0.168	1	Platelet	RHOF
STK24	0.001071	0.462424	0.085	0.111	1	Platelet	STK24
EIF4G3	0.001171	0.784948	0.129	0.12	1	Platelet	EIF4G3
RPA1	0.001274	0.869399	0.107	0.097	1	Platelet	RPA1
TRAM11	0.001344	0.843847	0.236	0.325	1	Platelet	TRAM1
EIF4G2	0.00218	0.781611	0.334	0.489	1	Platelet	EIF4G2
FBXO71	0.002188	0.724159	0.159	0.208	1	Platelet	FBXO7
MTSS13	0.002501	0.533698	0.123	0.156	1	Platelet	MTSS1
KAT6A	0.003264	0.541731	0.113	0.144	1	Platelet	KAT6A
LRP101	0.003562	0.715761	0.163	0.21	1	Platelet	LRP10
CCDC85B1	0.005982	1.166322	0.301	0.366	1	Platelet	CCDC85B
KIAA0513	0.006644	0.437101	0.098	0.122	1	Platelet	KIAA0513
POLR2E1	0.006782	1.09672	0.254	0.292	1	Platelet	POLR2E
RYBP	0.007118	0.884088	0.117	0.111	1	Platelet	RYBP
UNC13D	0.008155	0.517193	0.1	0.125	1	Platelet	UNC13D
CAPN21	0.009673	1.062351	0.257	0.293	1	Platelet	CAPN2
FOXP3	0	1.397086	0.379	0.003	0	Treg	FOXP3
RTKN2	0	1.356144	0.311	0.004	0	Treg	RTKN2
CTLA4	#######	0.803603	0.189	0.005	#######	Treg	CTLA4
IL2RA	#######	0.878846	0.223	0.007	#######	Treg	IL2RA
IKZF21	#######	1.133755	0.282	0.017	#######	Treg	IKZF2
ID34	#######	1.441771	0.437	0.046	#######	Treg	ID3
CD274	#######	1.611622	0.626	0.101	#######	Treg	CD27
CD3E4	#######	1.882753	0.908	0.205	#######	Treg	CD3E
CD3G4	#######	1.721309	0.762	0.155	#######	Treg	CD3G
CD3D4	#######	1.613384	0.816	0.171	#######	Treg	CD3D
LEF13	#######	1.463908	0.539	0.089	#######	Treg	LEF1
IL325	#######	2.140731	0.854	0.216	#######	Treg	IL32
FAIM36	5.00E-93	1.484943	0.602	0.136	1.64E-88	Treg	FAIM3
TIGIT2	5.79E-90	1.212566	0.32	0.041	1.90E-85	Treg	TIGIT
LINC00402	7.80E-80	0.54448	0.15	0.011	2.55E-75	Treg	LINC00402
C12orf577	6.03E-79	1.704354	0.816	0.303	1.97E-74	Treg	C12orf57
RGCC5	7.20E-79	1.180013	0.466	0.093	2.36E-74	Treg	RGCC
LTB6	4.37E-78	1.550449	0.825	0.283	1.43E-73	Treg	LTB
LDHB5	9.62E-78	1.672907	0.869	0.391	3.15E-73	Treg	LDHB
RHOH6	2.97E-72	1.158157	0.485	0.109	9.73E-68	Treg	RHOH
RPL305	6.56E-72	1.151569	0.981	0.887	2.15E-67	Treg	RPL30
RPL107	4.21E-71	1.166945	0.99	0.88	1.38E-66	Treg	RPL10
RPL326	1.13E-69	1.234349	0.971	0.871	3.71E-65	Treg	RPL32
RPS15A7	1.50E-69	1.180475	0.981	0.869	4.90E-65	Treg	RPS15A
RPL146	3.45E-69	1.191498	0.961	0.812	1.13E-64	Treg	RPL14
RPL57	2.88E-67	1.328534	0.971	0.801	9.43E-63	Treg	RPL5
PTPRCAP7	6.68E-66	1.187245	0.845	0.277	2.19E-61	Treg	PTPRCAP
RPS257	2.24E-65	1.23737	0.976	0.776	7.33E-61	Treg	RPS25
RPS38	4.77E-65	1.213911	0.966	0.833	1.56E-60	Treg	RPS3
RPS126	6.75E-65	1.24813	0.976	0.882	2.21E-60	Treg	RPS12
RPL18A7	3.19E-64	1.090219	0.951	0.828	1.05E-59	Treg	RPL18A
RPS277	2.10E-63	1.345232	0.976	0.832	6.88E-59	Treg	RPS27
SPOCK25	2.34E-63	1.279718	0.495	0.123	7.65E-59	Treg	SPOCK2
RPS156	7.35E-63	0.922079	0.966	0.867	2.41E-58	Treg	RPS15
RPL116	9.19E-63	1.019949	0.981	0.884	3.01E-58	Treg	RPL11
PIK3IP14	1.68E-62	1.191794	0.456	0.11	5.50E-58	Treg	PIK3IP1
MAL3	1.93E-62	1.055186	0.374	0.073	6.30E-58	Treg	MAL

TTN1	6.43E-62	0.817716	0.16	0.015	2.10E-57	Treg	TTN
RPL137	1.92E-60	1.081389	0.951	0.863	6.29E-56	Treg	RPL13
RPS77	1.44E-59	0.894541	0.995	0.865	4.72E-55	Treg	RPS7
FXYD2	1.60E-59	0.52005	0.126	0.01	5.23E-55	Treg	FXYD2
RPL196	1.77E-59	0.948357	0.961	0.87	5.81E-55	Treg	RPL19
IL7R4	4.03E-59	0.521631	0.626	0.159	1.32E-54	Treg	IL7R
CAMK44	4.97E-59	1.139201	0.379	0.08	1.63E-54	Treg	CAMK4
RPL296	7.14E-59	0.964528	0.951	0.836	2.34E-54	Treg	RPL29
RPS187	7.45E-59	1.187696	0.961	0.789	2.44E-54	Treg	RPS18
EEF1A18	1.08E-58	1.120646	0.966	0.892	3.54E-54	Treg	EEF1A1
RPS67	3.45E-58	1.171416	0.937	0.783	1.13E-53	Treg	RPS6
RPS3A7	8.54E-58	1.067705	0.956	0.858	2.79E-53	Treg	RPS3A
RPL366	9.90E-58	1.074001	0.961	0.831	3.24E-53	Treg	RPL36
RPS4X7	1.25E-57	1.065104	0.951	0.813	4.09E-53	Treg	RPS4X
RPL345	2.09E-57	0.929292	0.966	0.874	6.85E-53	Treg	RPL34
RPL327	3.39E-57	1.233487	0.947	0.747	1.11E-52	Treg	RPL3
RPS57	5.72E-57	1.287413	0.937	0.707	1.87E-52	Treg	RPS5
LCK5	1.64E-56	1.195693	0.617	0.191	5.35E-52	Treg	LCK
RPS237	2.74E-56	1.028184	0.971	0.826	8.96E-52	Treg	RPS23
RPS298	4.79E-56	1.20817	0.947	0.766	1.57E-51	Treg	RPS29
RPS86	1.59E-55	0.969198	0.971	0.879	5.20E-51	Treg	RPS8
RPL377	4.41E-55	0.926612	0.961	0.868	1.45E-50	Treg	RPL37
RPS27A7	7.30E-55	1.021564	0.966	0.868	2.39E-50	Treg	RPS27A
RPL35A6	1.38E-54	0.930685	0.981	0.872	4.51E-50	Treg	RPL35A
CCDC141	1.57E-54	0.420972	0.102	0.007	5.14E-50	Treg	CCDC141
RPL186	1.80E-54	0.926599	0.971	0.851	5.91E-50	Treg	RPL18
RPS2111	2.99E-54	0.972181	0.947	0.837	9.78E-50	Treg	RPS21
AES6	6.73E-54	1.360782	0.816	0.421	2.20E-49	Treg	AES
RPS286	5.61E-53	0.790377	0.981	0.9	1.84E-48	Treg	RPS28
AQP34	2.73E-51	1.04402	0.354	0.077	8.95E-47	Treg	AQP3
NPM18	1.69E-50	1.265448	0.879	0.526	5.54E-46	Treg	NPM1
RPL87	3.00E-50	0.908491	0.971	0.859	9.81E-46	Treg	RPL8
CCR4	1.18E-49	0.834898	0.112	0.009	3.86E-45	Treg	CCR4
EEF1B27	1.45E-49	1.24217	0.932	0.674	4.75E-45	Treg	EEF1B2
RPSA8	3.13E-49	1.182049	0.908	0.7	1.03E-44	Treg	RPSA
RPL126	5.25E-49	0.911902	0.961	0.869	1.72E-44	Treg	RPL12
TMEM667	6.89E-49	1.262259	0.854	0.551	2.26E-44	Treg	TMEM66
PRKCQ-AS	1.70E-48	1.048727	0.437	0.117	5.55E-44	Treg	PRKCQ-AS1
ISG206	1.89E-48	1.204625	0.539	0.18	6.19E-44	Treg	ISG20
RPL10A7	1.90E-48	1.181623	0.908	0.689	6.21E-44	Treg	RPL10A
LBH6	3.51E-48	1.102538	0.529	0.166	1.15E-43	Treg	LBH
RPS199	1.08E-46	0.944827	0.976	0.816	3.53E-42	Treg	RPS19
ETS16	1.15E-46	1.287225	0.558	0.19	3.77E-42	Treg	ETS1
EVL6	2.98E-46	1.243545	0.694	0.291	9.76E-42	Treg	EVL
RPL67	3.36E-46	0.773041	0.966	0.865	1.10E-41	Treg	RPL6
RPL286	7.74E-46	0.683973	0.971	0.897	2.53E-41	Treg	RPL28
GNB2L17	8.77E-46	0.891589	0.932	0.801	2.87E-41	Treg	GNB2L1
BCL25	9.48E-46	0.941156	0.291	0.06	3.10E-41	Treg	BCL2
BIRC33	3.33E-45	1.092163	0.403	0.11	1.09E-40	Treg	BIRC3
KLF27	3.54E-45	1.172241	0.874	0.53	1.16E-40	Treg	KLF2
BTG16	5.55E-45	1.089805	0.942	0.684	1.82E-40	Treg	BTG1
RCAN33	8.93E-45	1.071402	0.408	0.113	2.92E-40	Treg	RCAN3
THEM44	1.05E-44	1.137683	0.335	0.081	3.44E-40	Treg	THEM4
CD284	1.46E-44	0.829285	0.223	0.038	4.77E-40	Treg	CD28
RPL247	1.05E-43	0.872729	0.961	0.801	3.44E-39	Treg	RPL24
LINC00861	1.17E-43	0.981277	0.573	0.188	3.82E-39	Treg	LINC00861
IFITM15	1.72E-43	0.869491	0.83	0.337	5.62E-39	Treg	IFITM1
RPL7A8	1.74E-43	0.799015	0.976	0.84	5.69E-39	Treg	RPL7A

FAM102A3	2.58E-43	0.65755	0.209	0.034	8.44E-39	Treg	FAM102A
RPS220	7.50E-41	0.848868	0.951	0.817	2.45E-36	Treg	RPS2
CCR74	1.17E-40	0.864402	0.393	0.102	3.84E-36	Treg	CCR7
ARID5B4	1.72E-40	0.975901	0.311	0.075	5.63E-36	Treg	ARID5B
TPT14	1.81E-40	0.717937	0.951	0.912	5.93E-36	Treg	TPT1
RPL416	2.03E-40	0.963107	0.971	0.884	6.65E-36	Treg	RPL41
RPL23A8	1.82E-39	0.894041	0.942	0.741	5.97E-35	Treg	RPL23A
SKAP15	2.58E-39	1.100248	0.447	0.144	8.43E-35	Treg	SKAP1
MT-ND4L7	5.17E-39	0.860411	0.976	0.775	1.69E-34	Treg	MT-ND4L
AC006129	9.58E-39	0.944454	0.354	0.097	3.14E-34	Treg	AC006129.2
RPS146	5.14E-38	0.747023	0.961	0.874	1.68E-33	Treg	RPS14
CD26	6.36E-38	1.056559	0.519	0.179	2.08E-33	Treg	CD2
HSPA89	3.61E-37	1.063806	0.859	0.578	1.18E-32	Treg	HSPA8
MALAT16	4.44E-36	0.943385	0.985	0.944	1.45E-31	Treg	MALAT1
RPL225	7.44E-36	0.768866	0.956	0.841	2.44E-31	Treg	RPL22
RPS167	1.21E-35	0.804431	0.937	0.767	3.97E-31	Treg	RPS16
ADTRP2	1.75E-35	0.408688	0.136	0.018	5.72E-31	Treg	ADTRP
EEF1D5	8.42E-35	0.791045	0.922	0.788	2.76E-30	Treg	EEF1D
FLT3LG4	1.65E-34	0.900992	0.422	0.142	5.39E-30	Treg	FLT3LG
CD2475	5.74E-34	0.655797	0.505	0.173	1.88E-29	Treg	CD247
GLTSCR25	1.64E-33	1.02238	0.762	0.458	5.36E-29	Treg	GLTSCR2
RPL385	1.75E-33	0.868705	0.932	0.728	5.72E-29	Treg	RPL38
CD77	2.91E-33	0.638187	0.568	0.202	9.52E-29	Treg	CD7
SH3YL13	2.58E-32	0.822529	0.291	0.078	8.46E-28	Treg	SH3YL1
LEPROTL14	3.39E-32	1.074358	0.544	0.252	1.11E-27	Treg	LEPROTL1
RPL94	4.62E-32	0.763024	0.927	0.803	1.51E-27	Treg	RPL9
SHMT22	2.47E-31	1.033036	0.364	0.122	8.09E-27	Treg	SHMT2
RPLP07	2.54E-31	0.789156	0.942	0.778	8.31E-27	Treg	RPLP0
OCIAD27	3.85E-31	0.793626	0.345	0.106	1.26E-26	Treg	OCIAD2
B2M4	4.04E-31	0.631476	1	0.979	1.32E-26	Treg	B2M
RPL394	3.05E-30	0.583305	0.981	0.915	9.97E-26	Treg	RPL39
HIST1H4C7	6.99E-30	1.033263	0.777	0.484	2.29E-25	Treg	HIST1H4C
ARL4C5	9.64E-30	0.936174	0.485	0.191	3.16E-25	Treg	ARL4C
NACA8	1.65E-29	0.717408	0.956	0.821	5.39E-25	Treg	NACA
SLC38A18	1.95E-29	0.927856	0.364	0.121	6.39E-25	Treg	SLC38A1
SYNE23	2.20E-29	1.009083	0.374	0.124	7.19E-25	Treg	SYNE2
DGKA3	3.21E-29	0.91368	0.35	0.117	1.05E-24	Treg	DGKA
RPL268	3.72E-29	0.728357	0.966	0.807	1.22E-24	Treg	RPL26
PEBP18	6.61E-29	0.984625	0.534	0.245	2.16E-24	Treg	PEBP1
TRAF3IP36	1.36E-28	1.023447	0.718	0.431	4.45E-24	Treg	TRAF3IP3
IL2RG7	4.79E-28	0.88001	0.675	0.333	1.57E-23	Treg	IL2RG
LIME16	8.87E-28	0.878742	0.32	0.1	2.90E-23	Treg	LIME1
ITK5	1.23E-27	0.704398	0.277	0.078	4.03E-23	Treg	ITK
RPL178	6.12E-27	0.889765	0.879	0.716	2.00E-22	Treg	RPL17
BCL11B5	8.37E-27	0.763798	0.248	0.066	2.74E-22	Treg	BCL11B
PBXIP13	8.89E-27	1.023977	0.34	0.117	2.91E-22	Treg	PBXIP1
RPS265	1.25E-26	1.225298	0.937	0.83	4.08E-22	Treg	RPS26
RPL217	1.96E-26	0.698592	0.942	0.816	6.40E-22	Treg	RPL21
TRAF12	2.37E-26	0.621749	0.165	0.033	7.75E-22	Treg	TRAF1
ITGB74	3.41E-26	0.800287	0.354	0.124	1.11E-21	Treg	ITGB7
TCF74	5.46E-26	0.747421	0.354	0.117	1.79E-21	Treg	TCF7
TRAT13	8.97E-26	0.740434	0.248	0.067	2.94E-21	Treg	TRAT1
CRIP21	1.12E-25	0.520172	0.131	0.023	3.65E-21	Treg	CRIP2
STAM	1.16E-25	0.6587	0.238	0.064	3.81E-21	Treg	STAM
PIM26	1.74E-25	0.877757	0.272	0.081	5.70E-21	Treg	PIM2
RPL357	2.08E-25	0.701811	0.932	0.771	6.80E-21	Treg	RPL35
TBC1D42	2.59E-25	0.593552	0.17	0.036	8.49E-21	Treg	TBC1D4
RPS135	2.92E-25	0.635629	0.981	0.889	9.56E-21	Treg	RPS13

CDC25B5	3.71E-25	0.708354	0.282	0.087	1.22E-20	Treg	CDC25B
PSIP16	3.85E-25	0.908899	0.485	0.229	1.26E-20	Treg	PSIP1
GATA33	4.67E-25	0.762961	0.218	0.056	1.53E-20	Treg	GATA3
SLAMF13	1.25E-24	0.50002	0.16	0.033	4.08E-20	Treg	SLAMF1
PCED1B5	3.96E-24	0.957423	0.311	0.105	1.29E-19	Treg	PCED1B
RPLP25	4.25E-24	0.522168	0.961	0.878	1.39E-19	Treg	RPLP2
CIRBP8	5.60E-24	0.833894	0.704	0.448	1.83E-19	Treg	CIRBP
PRDX26	2.24E-23	0.746126	0.481	0.222	7.34E-19	Treg	PRDX2
CD57	4.56E-23	0.515414	0.209	0.054	1.49E-18	Treg	CD5
TRIB22	6.75E-23	0.548079	0.16	0.035	2.21E-18	Treg	TRIB2
TSPAN52	7.96E-23	0.405716	0.15	0.031	2.60E-18	Treg	TSPAN5
LAIR22	8.20E-23	0.440398	0.136	0.026	2.68E-18	Treg	LAIR2
ATP8B21	1.54E-22	0.485644	0.121	0.022	5.05E-18	Treg	ATP8B2
CD526	2.62E-22	0.764041	0.879	0.651	8.59E-18	Treg	CD52
ZNF1012	5.31E-22	0.57381	0.209	0.057	1.74E-17	Treg	ZNF101
TSTD17	8.09E-22	0.720949	0.408	0.173	2.65E-17	Treg	TSTD1
RPS107	8.83E-22	0.782617	0.854	0.765	2.89E-17	Treg	RPS10
CTB-133G	2.48E-21	0.814162	0.379	0.158	8.10E-17	Treg	CTB-133G6.1
RAN8	3.25E-21	0.828903	0.699	0.454	1.06E-16	Treg	RAN
INPP4B3	3.31E-21	0.529025	0.209	0.057	1.08E-16	Treg	INPP4B
EEF26	7.02E-21	0.681089	0.874	0.7	2.30E-16	Treg	EEF2
GIMAP75	1.39E-20	0.836459	0.796	0.574	4.54E-16	Treg	GIMAP7
SUSD34	1.79E-20	0.527865	0.238	0.073	5.85E-16	Treg	SUSD3
RORA3	2.33E-20	0.727162	0.267	0.088	7.62E-16	Treg	RORA
GBP53	3.72E-20	1.127235	0.383	0.164	1.22E-15	Treg	GBP5
FBLN53	4.61E-20	0.471014	0.131	0.027	1.51E-15	Treg	FBLN5
RPL157	4.95E-20	0.564542	0.961	0.802	1.62E-15	Treg	RPL15
CTC-523E2	5.33E-20	0.501113	0.121	0.024	1.75E-15	Treg	CTC-523E23.11
MT-ATP89	6.64E-20	0.807919	0.883	0.738	2.18E-15	Treg	MT-ATP8
LSR2	9.24E-20	0.329685	0.126	0.026	3.02E-15	Treg	LSR
RPL46	1.17E-19	0.653032	0.898	0.724	3.84E-15	Treg	RPL4
RPL37A7	1.24E-19	0.551402	0.947	0.809	4.07E-15	Treg	RPL37A
TNFRSF41	1.40E-19	0.34525	0.102	0.018	4.58E-15	Treg	TNFRSF4
SELL5	1.61E-19	0.777128	0.709	0.463	5.27E-15	Treg	SELL
SIT16	1.82E-19	0.672415	0.243	0.079	5.97E-15	Treg	SIT1
SELM4	2.29E-19	0.620034	0.204	0.06	7.49E-15	Treg	SELM
NMT24	2.29E-19	0.540933	0.146	0.034	7.49E-15	Treg	NMT2
CASK	2.97E-19	0.381366	0.112	0.021	9.73E-15	Treg	CASK
EEF1G7	3.11E-19	0.910873	0.723	0.525	1.02E-14	Treg	EEF1G
TMEM2384	3.51E-19	0.580132	0.209	0.062	1.15E-14	Treg	TMEM238
PRKCQ4	5.16E-19	0.552544	0.17	0.044	1.69E-14	Treg	PRKCQ
FCRL33	6.21E-19	0.352148	0.126	0.026	2.03E-14	Treg	FCRL3
BTF36	1.00E-18	0.569803	0.922	0.743	3.29E-14	Treg	BTF3
SOD17	2.56E-18	0.895976	0.597	0.394	8.38E-14	Treg	SOD1
KCNA33	2.59E-18	0.436626	0.189	0.053	8.49E-14	Treg	KCNA3
LINC00649	3.15E-18	0.493048	0.16	0.041	1.03E-13	Treg	LINC00649
ANKH	3.74E-18	0.438426	0.136	0.031	1.23E-13	Treg	ANKH
TOMM75	4.20E-18	0.63287	0.874	0.702	1.37E-13	Treg	TOMM7
HNRNPA1	6.11E-18	0.659123	0.845	0.63	2.00E-13	Treg	HNRNPA1
CUTA5	1.25E-17	0.86905	0.597	0.387	4.08E-13	Treg	CUTA
GYPC6	1.73E-17	0.705683	0.49	0.258	5.66E-13	Treg	GYPC
ITM2A6	2.81E-17	0.634154	0.296	0.114	9.21E-13	Treg	ITM2A
EPHX23	3.32E-17	0.364004	0.136	0.032	1.09E-12	Treg	EPHX2
RPS206	3.86E-17	0.760313	0.796	0.592	1.26E-12	Treg	RPS20
OPTN4	3.86E-17	0.611608	0.291	0.112	1.26E-12	Treg	OPTN
CLEC2D5	4.02E-17	0.61577	0.252	0.09	1.32E-12	Treg	CLEC2D
RPL13A7	8.23E-17	0.558673	0.927	0.767	2.69E-12	Treg	RPL13A
SLFN55	1.51E-16	0.859883	0.383	0.186	4.94E-12	Treg	SLFN5

TMEM256	1.80E-16	0.659041	0.291	0.121	5.90E-12	Treg	TMEM256-PLSCR3
SLFN12L3	6.17E-16	0.49955	0.16	0.045	2.02E-11	Treg	SLFN12L
FOXP15	6.36E-16	0.819178	0.451	0.254	2.08E-11	Treg	FOXP1
MT-CYB6	6.58E-16	0.452743	0.976	0.931	2.16E-11	Treg	MT-CYB
RPL3110	9.83E-16	0.61123	0.874	0.736	3.22E-11	Treg	RPL31
RPL36AL5	9.97E-16	0.740822	0.874	0.723	3.26E-11	Treg	RPL36AL
CLDND13	1.18E-15	0.86115	0.306	0.134	3.85E-11	Treg	CLDND1
RPS4Y16	1.82E-15	1.006557	0.621	0.466	5.97E-11	Treg	RPS4Y1
ATP6V0E2	4.13E-15	0.477593	0.189	0.062	1.35E-10	Treg	ATP6V0E2
MIF7	4.14E-15	0.653113	0.777	0.571	1.36E-10	Treg	MIF
C14orf644	6.68E-15	0.40731	0.136	0.036	2.19E-10	Treg	C14orf64
FAU4	6.85E-15	0.395927	0.947	0.904	2.24E-10	Treg	FAU
RPL277	6.93E-15	0.561552	0.888	0.738	2.27E-10	Treg	RPL27
TTC39C2	1.18E-14	0.76944	0.301	0.136	3.87E-10	Treg	TTC39C
BEX25	1.61E-14	0.490503	0.165	0.051	5.25E-10	Treg	BEX2
MAD1L13	1.82E-14	0.638153	0.277	0.119	5.94E-10	Treg	MAD1L1
SNHG87	2.13E-14	0.793901	0.49	0.295	6.99E-10	Treg	SNHG8
LAT7	2.15E-14	0.390664	0.379	0.179	7.03E-10	Treg	LAT
PTGES33	2.23E-14	0.659485	0.665	0.447	7.29E-10	Treg	PTGES3
RASGRP15	3.57E-14	0.499783	0.17	0.053	1.17E-09	Treg	RASGRP1
CXCR45	5.37E-14	0.615591	0.35	0.169	1.76E-09	Treg	CXCR4
LIMD28	1.21E-13	0.598163	0.786	0.592	3.96E-09	Treg	LIMD2
PRKCH5	1.70E-13	0.551559	0.272	0.115	5.57E-09	Treg	PRKCH
ACAP16	1.77E-13	0.752774	0.456	0.256	5.79E-09	Treg	ACAP1
CD65	1.81E-13	0.521708	0.194	0.069	5.92E-09	Treg	CD6
CDKN1B6	2.55E-13	0.643383	0.369	0.193	8.36E-09	Treg	CDKN1B
SESN33	2.72E-13	0.554259	0.248	0.103	8.92E-09	Treg	SESN3
RAC27	2.85E-13	0.48963	0.782	0.581	9.34E-09	Treg	RAC2
EIF3E5	4.55E-13	0.598059	0.68	0.492	1.49E-08	Treg	EIF3E
17-Sep	6.45E-13	0.479087	0.316	0.144	2.11E-08	Treg	1-Sep
CD591	6.70E-13	0.515117	0.146	0.045	2.19E-08	Treg	CD59
ST134	6.71E-13	0.557709	0.621	0.436	2.20E-08	Treg	ST13
RNF2141	9.92E-13	0.569065	0.17	0.059	3.25E-08	Treg	RNF214
WHSC1L1	1.21E-12	0.703773	0.447	0.27	3.95E-08	Treg	WHSC1L1
SNRPD26	1.95E-12	0.549604	0.762	0.577	6.40E-08	Treg	SNRPD2
ODF2L3	1.97E-12	0.508646	0.204	0.078	6.46E-08	Treg	ODF2L
FAM134B3	2.28E-12	0.483796	0.131	0.039	7.46E-08	Treg	FAM134B
SSR27	2.59E-12	0.612626	0.66	0.486	8.49E-08	Treg	SSR2
CISH4	2.98E-12	0.612173	0.214	0.083	9.74E-08	Treg	CISH
GIMAP55	3.02E-12	0.618993	0.481	0.277	9.89E-08	Treg	GIMAP5
SIRPG4	3.07E-12	0.463318	0.131	0.039	1.01E-07	Treg	SIRPG
GCC26	3.55E-12	0.655341	0.398	0.229	1.16E-07	Treg	GCC2
HINT17	4.40E-12	0.50261	0.791	0.612	1.44E-07	Treg	HINT1
MLLT33	4.81E-12	0.465292	0.16	0.054	1.57E-07	Treg	MLLT3
TMEM123	6.72E-12	0.551488	0.519	0.34	2.20E-07	Treg	TMEM123
COX7C7	9.02E-12	0.516008	0.869	0.706	2.95E-07	Treg	COX7C
CMTM82	1.20E-11	0.353066	0.102	0.027	3.94E-07	Treg	CMTM8
HSP90AB1	1.67E-11	0.668729	0.748	0.553	5.47E-07	Treg	HSP90AB1
BIN17	1.92E-11	0.615685	0.311	0.155	6.27E-07	Treg	BIN1
TECR6	2.32E-11	0.59154	0.393	0.23	7.60E-07	Treg	TECR
PTMA1	3.84E-11	0.303354	0.976	0.906	1.26E-06	Treg	PTMA
ANKRD12	4.08E-11	0.630777	0.558	0.393	1.34E-06	Treg	ANKRD12
COMMD6	4.35E-11	0.455615	0.83	0.702	1.42E-06	Treg	COMMD6
MRFAP1L1	4.62E-11	0.515514	0.291	0.145	1.51E-06	Treg	MRFAP1L1
AKTIP3	4.64E-11	0.419466	0.136	0.044	1.52E-06	Treg	AKTIP
ZAP705	4.85E-11	0.517302	0.272	0.124	1.59E-06	Treg	ZAP70
MZT2A6	4.99E-11	0.62691	0.345	0.191	1.63E-06	Treg	MZT2A
ITGA64	5.01E-11	0.413104	0.17	0.061	1.64E-06	Treg	ITGA6

UBE2D27	5.21E-11	0.522923	0.583	0.424	1.71E-06	Treg	UBE2D2
THOC33	6.34E-11	0.54139	0.223	0.098	2.07E-06	Treg	THOC3
PDE3B3	6.46E-11	0.423874	0.175	0.066	2.12E-06	Treg	PDE3B
MZT2B6	7.45E-11	0.503406	0.641	0.488	2.44E-06	Treg	MZT2B
SNHG75	7.89E-11	0.602108	0.369	0.211	2.58E-06	Treg	SNHG7
RBM384	9.32E-11	0.392864	0.286	0.139	3.05E-06	Treg	RBM38
FAS2	1.04E-10	0.539857	0.155	0.056	3.41E-06	Treg	FAS
ABHD14B5	1.05E-10	0.581285	0.32	0.174	3.45E-06	Treg	ABHD14B
PYHIN12	1.60E-10	0.33214	0.204	0.08	5.23E-06	Treg	PYHIN1
ARHGAP15	1.64E-10	0.527252	0.369	0.207	5.38E-06	Treg	ARHGAP15
NDFIP13	1.82E-10	0.5663	0.495	0.339	5.95E-06	Treg	NDFIP1
PIM14	2.25E-10	0.533357	0.33	0.174	7.36E-06	Treg	PIM1
CCNI5	2.32E-10	0.4697	0.845	0.694	7.59E-06	Treg	CCNI
HMGN19	2.75E-10	0.416126	0.544	0.361	8.99E-06	Treg	HMGN1
HNRNPDL	2.89E-10	0.547195	0.617	0.462	9.46E-06	Treg	HNRNPDL
TMEM2043	2.98E-10	0.336301	0.146	0.051	9.75E-06	Treg	TMEM204
OXNAD14	3.24E-10	0.473253	0.189	0.077	1.06E-05	Treg	OXNAD1
TNIK5	3.54E-10	0.412131	0.18	0.07	1.16E-05	Treg	TNIK
STMN34	3.57E-10	0.398822	0.136	0.046	1.17E-05	Treg	STMN3
STMN16	3.82E-10	0.393037	0.184	0.073	1.25E-05	Treg	STMN1
GLCCI12	3.99E-10	0.397002	0.136	0.046	1.31E-05	Treg	GLCCI1
BUB36	4.98E-10	0.529135	0.364	0.213	1.63E-05	Treg	BUB3
AAK15	5.66E-10	0.589515	0.422	0.264	1.85E-05	Treg	AAK1
RPL36A8	6.64E-10	0.492849	0.816	0.634	2.17E-05	Treg	RPL36A
LY98	7.45E-10	0.488753	0.17	0.066	2.44E-05	Treg	LY9
EIF3F7	7.74E-10	0.468939	0.684	0.543	2.53E-05	Treg	EIF3F
TAGAP6	8.61E-10	0.667815	0.403	0.249	2.82E-05	Treg	TAGAP
PPP1R25	9.00E-10	0.512408	0.427	0.275	2.95E-05	Treg	PPP1R2
EIF4A27	1.11E-09	0.646316	0.456	0.306	3.62E-05	Treg	EIF4A2
CWF19L25	1.25E-09	0.459352	0.267	0.134	4.09E-05	Treg	CWF19L2
CYLD2	1.26E-09	0.64155	0.354	0.209	4.11E-05	Treg	CYLD
DNAJB15	1.43E-09	0.501996	0.267	0.136	4.69E-05	Treg	DNAJB1
GOLGA8B5	1.46E-09	0.35707	0.141	0.05	4.77E-05	Treg	GOLGA8B
DDX245	1.54E-09	0.421634	0.529	0.368	5.04E-05	Treg	DDX24
IL6ST2	1.55E-09	0.543324	0.296	0.157	5.06E-05	Treg	IL6ST
EIF3H5	1.56E-09	0.590665	0.68	0.53	5.10E-05	Treg	EIF3H
GS1-251I9	1.60E-09	0.576771	0.277	0.143	5.23E-05	Treg	GS1-251I9.4
TMEM2436	1.68E-09	0.547715	0.306	0.17	5.51E-05	Treg	TMEM243
RPLP15	1.71E-09	0.298611	0.956	0.945	5.59E-05	Treg	RPLP1
RPS95	1.91E-09	0.347797	0.888	0.831	6.25E-05	Treg	RPS9
MYC4	1.93E-09	0.612982	0.218	0.1	6.33E-05	Treg	MYC
FUS7	2.08E-09	0.583112	0.583	0.429	6.81E-05	Treg	FUS
LDLRAP14	2.10E-09	0.329213	0.214	0.094	6.87E-05	Treg	LDLRAP1
RARRES35	3.01E-09	0.417415	0.544	0.365	9.87E-05	Treg	RARRES3
Sep-68	3.70E-09	0.548195	0.456	0.313	0.000121	Treg	6-Sep
EPB416	4.03E-09	0.486836	0.354	0.209	0.000132	Treg	EPB41
FXYD52	4.25E-09	0.378901	0.883	0.76	0.000139	Treg	FXYD5
ANXA2R5	4.53E-09	0.571945	0.199	0.09	0.000148	Treg	ANXA2R
RPS27L4	6.56E-09	0.878036	0.505	0.365	0.000215	Treg	RPS27L
NOP586	8.11E-09	0.726379	0.296	0.17	0.000265	Treg	NOP58
ZNF8623	8.47E-09	0.473204	0.184	0.081	0.000277	Treg	ZNF862
C12orf656	9.85E-09	0.456691	0.199	0.091	0.000322	Treg	C12orf65
PLCG14	1.00E-08	0.354569	0.112	0.037	0.000328	Treg	PLCG1
LITAF2	1.45E-08	0.257255	0.558	0.393	0.000474	Treg	LITAF
RIC34	1.53E-08	0.270453	0.102	0.033	0.000499	Treg	RIC3
TXK4	1.54E-08	0.434418	0.175	0.073	0.000503	Treg	TXK
STK44	2.18E-08	0.549007	0.583	0.448	0.000712	Treg	STK4
P2RY102	2.18E-08	0.311087	0.102	0.033	0.000714	Treg	P2RY10

RASA24	2.40E-08	0.527349	0.257	0.135	0.000785	Treg	RASA2
PHF17	2.48E-08	0.386586	0.17	0.073	0.000813	Treg	PHF1
MAT2B6	2.92E-08	0.426912	0.427	0.287	0.000956	Treg	MAT2B
UXS11	4.29E-08	0.307317	0.204	0.097	0.001404	Treg	UXS1
HOXB23	4.50E-08	0.336243	0.112	0.039	0.001472	Treg	HOXB2
RP11-18H	4.75E-08	0.275143	0.117	0.042	0.001554	Treg	RP11-18H21.1
CD965	5.24E-08	0.415355	0.209	0.099	0.001716	Treg	CD96
RPSAP585	6.30E-08	0.510744	0.248	0.134	0.002062	Treg	RPSAP58
PDE4B2	6.43E-08	0.590818	0.155	0.065	0.002105	Treg	PDE4B
EPC17	8.21E-08	0.482118	0.427	0.297	0.002687	Treg	EPC1
ASF1A4	9.73E-08	0.430281	0.189	0.091	0.003186	Treg	ASF1A
TBC1D10C	1.02E-07	0.56289	0.345	0.214	0.003349	Treg	TBC1D10C
EIF3L6	1.19E-07	0.502819	0.65	0.543	0.003902	Treg	EIF3L
ZNF274	1.20E-07	0.262131	0.117	0.043	0.003912	Treg	ZNF274
SPTAN13	1.22E-07	0.549004	0.282	0.163	0.004001	Treg	SPTAN1
DYRK25	1.25E-07	0.328348	0.16	0.07	0.004091	Treg	DYRK2
TNFRSF10.	1.35E-07	0.350842	0.117	0.044	0.004431	Treg	TNFRSF10A
S1PR44	1.40E-07	0.44557	0.408	0.28	0.004583	Treg	S1PR4
CCDC663	1.43E-07	0.472795	0.17	0.078	0.004668	Treg	CCDC66
TMEM50B	1.67E-07	0.412061	0.223	0.117	0.005464	Treg	TMEM50B
SYPL15	1.73E-07	0.512551	0.277	0.161	0.005661	Treg	SYPL1
TUBB9	1.82E-07	0.391803	0.524	0.383	0.005955	Treg	TUBB
NCL8	1.85E-07	0.400527	0.65	0.5	0.006047	Treg	NCL
TXNIP7	1.92E-07	0.32269	0.883	0.747	0.006289	Treg	TXNIP
CNBP4	2.00E-07	0.495817	0.636	0.525	0.006549	Treg	CNBP
LINC-PINT	2.11E-07	0.343021	0.15	0.065	0.006905	Treg	LINC-PINT
SFXN14	2.36E-07	0.408506	0.175	0.082	0.007715	Treg	SFXN1
CXCR34	2.81E-07	0.389178	0.126	0.049	0.009188	Treg	CXCR3
TOMM205	3.09E-07	0.6385	0.461	0.348	0.010126	Treg	TOMM20
PPP3CC5	3.16E-07	0.48341	0.233	0.125	0.010345	Treg	PPP3CC
DAD15	3.22E-07	1.209178	0.534	0.406	0.010545	Treg	DAD1
TRAF53	3.24E-07	0.302354	0.107	0.039	0.010602	Treg	TRAF5
OBFC11	3.41E-07	0.376095	0.17	0.079	0.01115	Treg	OBFC1
GPR1835	4.36E-07	0.50068	0.175	0.083	0.014285	Treg	GPR183
DDX65	4.37E-07	0.442842	0.306	0.185	0.014321	Treg	DDX6
PABPC14	4.38E-07	0.29467	0.913	0.837	0.014333	Treg	PABPC1
HLA-A3	4.60E-07	0.324744	0.927	0.865	0.015058	Treg	HLA-A
SS18L24	4.70E-07	0.384273	0.291	0.175	0.015371	Treg	SS18L2
HNRNPA0	5.45E-07	0.458354	0.413	0.294	0.017829	Treg	HNRNPA0
ZBTB382	5.71E-07	0.545766	0.243	0.136	0.018686	Treg	ZBTB38
MPHOSPH	5.82E-07	0.433157	0.466	0.348	0.019068	Treg	MPHOSPH8
SNHG122	6.10E-07	0.433338	0.136	0.058	0.019959	Treg	SNHG12
RP11-51J9	8.38E-07	0.355367	0.112	0.043	0.027442	Treg	RP11-51J9.5
ZNF921	9.29E-07	0.274742	0.112	0.043	0.030404	Treg	ZNF92
NFYB	1.24E-06	0.383428	0.131	0.057	0.040657	Treg	NFYB
EML44	1.50E-06	0.684165	0.345	0.227	0.049153	Treg	EML4
DEF64	1.61E-06	0.470832	0.354	0.238	0.05281	Treg	DEF6
CCDC1672	1.66E-06	0.285469	0.155	0.072	0.054249	Treg	CCDC167
FAM107B4	1.76E-06	0.288235	0.451	0.32	0.0575	Treg	FAM107B
HSPB15	1.81E-06	0.449324	0.335	0.219	0.059258	Treg	HSPB1
CNN27	1.81E-06	0.366277	0.51	0.388	0.059395	Treg	CNN2
EIF2S34	1.85E-06	0.484886	0.432	0.322	0.060728	Treg	EIF2S3
PTGER23	2.07E-06	0.352489	0.214	0.114	0.067753	Treg	PTGER2
UQCRB4	2.18E-06	0.367977	0.816	0.684	0.071375	Treg	UQCRB
SESN15	2.52E-06	0.384969	0.146	0.067	0.082421	Treg	SESN1
GLOD43	2.69E-06	0.473936	0.243	0.142	0.088127	Treg	GLOD4
RP11-94L1	2.72E-06	0.38225	0.117	0.048	0.089127	Treg	RP11-94L15.2
APRT6	2.75E-06	0.34945	0.636	0.516	0.089974	Treg	APRT

CD473	2.82E-06	0.437293	0.422	0.312	0.092355	Treg	CD47
IMP36	2.86E-06	0.413962	0.383	0.277	0.093713	Treg	IMP3
IL166	2.95E-06	0.450636	0.369	0.259	0.096484	Treg	IL16
CNST1	3.31E-06	0.29051	0.218	0.119	0.108299	Treg	CNST
CCDC915	3.51E-06	0.405805	0.214	0.119	0.114901	Treg	CCDC91
ATP5O6	3.58E-06	0.388686	0.597	0.491	0.117229	Treg	ATP5O
SFI11	3.99E-06	0.317377	0.126	0.055	0.130512	Treg	SFI1
PPIA8	4.00E-06	0.303304	0.854	0.734	0.131064	Treg	PPIA
ISCU3	4.02E-06	0.338617	0.456	0.344	0.131671	Treg	ISCU
TMED44	4.08E-06	0.454201	0.286	0.183	0.133606	Treg	TMED4
CCDC109E	4.78E-06	0.450689	0.398	0.29	0.156363	Treg	CCDC109B
UXT6	5.26E-06	0.424217	0.553	0.435	0.17208	Treg	UXT
KIAA0040	5.33E-06	0.329857	0.228	0.131	0.174614	Treg	KIAA0040
CCND24	5.49E-06	0.479294	0.17	0.086	0.179684	Treg	CCND2
CARD114	5.68E-06	0.31594	0.112	0.046	0.185906	Treg	CARD11
TRABD2A	6.63E-06	0.328533	0.126	0.056	0.216969	Treg	TRABD2A
FOXO13	7.30E-06	0.39361	0.15	0.073	0.239027	Treg	FOXO1
UGP22	8.66E-06	0.519374	0.32	0.221	0.283436	Treg	UGP2
CCDC104	8.98E-06	0.415123	0.112	0.048	0.294022	Treg	CCDC104
GIMAP63	9.57E-06	0.404959	0.257	0.159	0.313397	Treg	GIMAP6
RPP21	1.05E-05	0.408636	0.243	0.148	0.344646	Treg	RPP21
MORC32	1.06E-05	0.535392	0.233	0.141	0.345633	Treg	MORC3
WDR75	1.13E-05	0.389195	0.126	0.058	0.370705	Treg	WDR75
HSPD17	1.19E-05	0.693844	0.369	0.271	0.388561	Treg	HSPD1
ERH5	1.26E-05	0.541458	0.456	0.354	0.412146	Treg	ERH
NAP1L45	1.29E-05	0.380882	0.374	0.268	0.422772	Treg	NAP1L4
MED107	1.33E-05	0.438855	0.301	0.204	0.436515	Treg	MED10
PPM1K7	1.37E-05	0.596296	0.199	0.113	0.447832	Treg	PPM1K
DBF4	1.45E-05	0.373463	0.121	0.055	0.475471	Treg	DBF4
DCXR1	1.50E-05	0.359508	0.354	0.25	0.49109	Treg	DCXR
PLEKHA12	1.75E-05	0.347424	0.15	0.076	0.571327	Treg	PLEKHA1
ANKRD36	1.82E-05	0.329039	0.121	0.055	0.594984	Treg	ANKRD36
CTD-3184	1.94E-05	0.30279	0.15	0.076	0.635443	Treg	CTD-3184A7.4
NGRN6	1.97E-05	0.403898	0.16	0.084	0.643983	Treg	NGRN
C19orf535	1.98E-05	0.388668	0.519	0.424	0.649145	Treg	C19orf53
NUDCD24	2.23E-05	0.29764	0.214	0.125	0.728711	Treg	NUDCD2
MRPL172	2.40E-05	0.415446	0.184	0.105	0.786284	Treg	MRPL17
UBXN18	2.43E-05	0.363805	0.558	0.462	0.795393	Treg	UBXN1
ZFP901	2.64E-05	0.350117	0.141	0.07	0.864702	Treg	ZFP90
TESPA14	2.99E-05	0.310249	0.141	0.07	0.980119	Treg	TESPA1
SMCHD11	3.11E-05	0.26494	0.5	0.391	1	Treg	SMCHD1
KIAA1430	3.61E-05	0.34209	0.18	0.101	1	Treg	KIAA1430
URI17	3.77E-05	0.412159	0.286	0.196	1	Treg	URI1
RCN26	4.31E-05	0.308656	0.209	0.124	1	Treg	RCN2
IFNAR23	4.76E-05	0.371581	0.286	0.193	1	Treg	IFNAR2
SBDS4	4.83E-05	0.481142	0.199	0.118	1	Treg	SBDS
NOL72	4.85E-05	0.362019	0.383	0.293	1	Treg	NOL7
ZNF5061	5.01E-05	0.412454	0.117	0.055	1	Treg	ZNF506
KLHL22	5.72E-05	0.313262	0.102	0.045	1	Treg	KLHL22
C14orf166	6.34E-05	0.461862	0.442	0.358	1	Treg	C14orf166
CHD32	6.50E-05	0.380333	0.291	0.201	1	Treg	CHD3
TFB1M1	6.64E-05	0.305634	0.121	0.059	1	Treg	TFB1M
ERGIC26	7.28E-05	0.435941	0.204	0.124	1	Treg	ERGIC2
CYTIP6	8.46E-05	0.323489	0.364	0.268	1	Treg	CYTIP
CHMP74	9.27E-05	0.362141	0.175	0.101	1	Treg	CHMP7
RBL25	9.38E-05	0.322538	0.354	0.258	1	Treg	RBL2
ZNF321	9.45E-05	0.333392	0.155	0.085	1	Treg	ZNF32
SRSF78	9.57E-05	0.549449	0.422	0.346	1	Treg	SRSF7

ANKRD49	9.76E-05	0.469456	0.228	0.146	1	Treg	ANKRD49
SPCS14	0.000104	0.392075	0.529	0.432	1	Treg	SPCS1
BET11	0.000106	0.514936	0.15	0.082	1	Treg	BET1
IPCEF11	0.000111	0.251169	0.184	0.105	1	Treg	IPCEF1
MDN11	0.000113	0.384186	0.107	0.051	1	Treg	MDN1
GSTK13	0.000119	0.466605	0.573	0.514	1	Treg	GSTK1
POLR2C4	0.000131	0.269389	0.17	0.097	1	Treg	POLR2C
ACTR1B1	0.000136	0.303015	0.155	0.086	1	Treg	ACTR1B
RSL1D15	0.000137	0.453894	0.456	0.375	1	Treg	RSL1D1
CCNDBP1:	0.000139	0.304626	0.34	0.252	1	Treg	CCNDBP1
PIK3R13	0.000144	0.322386	0.306	0.214	1	Treg	PIK3R1
BEX44	0.000148	0.563394	0.16	0.091	1	Treg	BEX4
TMC85	0.000164	0.483522	0.238	0.157	1	Treg	TMC8
DCP1A	0.000172	0.281145	0.146	0.079	1	Treg	DCP1A
ZFAS14	0.000175	0.655774	0.573	0.484	1	Treg	ZFAS1
SMDT15	0.000176	0.371259	0.49	0.414	1	Treg	SMDT1
IL10RA2	0.000181	0.407205	0.417	0.333	1	Treg	IL10RA
RAD212	0.000187	0.348372	0.379	0.299	1	Treg	RAD21
CCDC1011	0.000189	0.294411	0.165	0.095	1	Treg	CCDC101
RASA34	0.000194	0.273057	0.233	0.149	1	Treg	RASA3
IGSF83	0.000203	0.265989	0.107	0.052	1	Treg	IGSF8
EZR5	0.000206	0.405932	0.374	0.291	1	Treg	EZR
REXO25	0.000214	0.440333	0.184	0.112	1	Treg	REXO2
AIP4	0.000239	0.462264	0.33	0.254	1	Treg	AIP
MT-ND66	0.000244	0.271903	0.519	0.416	1	Treg	MT-ND6
UBE2N5	0.000246	0.324841	0.277	0.195	1	Treg	UBE2N
MEAF63	0.000246	0.369154	0.306	0.226	1	Treg	MEAF6
SLC7A62	0.000247	0.353165	0.131	0.07	1	Treg	SLC7A6
PARP16	0.000257	0.493647	0.291	0.212	1	Treg	PARP1
EIF5A7	0.000268	0.391434	0.49	0.408	1	Treg	EIF5A
S1PR16	0.000271	0.280059	0.189	0.112	1	Treg	S1PR1
IMPDH25	0.000275	0.332916	0.209	0.134	1	Treg	IMPDH2
PIGC2	0.000306	0.298264	0.18	0.111	1	Treg	PIGC
FGFR1OP2	0.000317	0.429398	0.311	0.231	1	Treg	FGFR1OP2
DCK5	0.000334	0.269145	0.218	0.142	1	Treg	DCK
FBL8	0.000343	0.399365	0.364	0.289	1	Treg	FBL
FNTA4	0.000347	0.363909	0.296	0.217	1	Treg	FNTA
EAPP2	0.000354	0.303388	0.311	0.229	1	Treg	EAPP
SIGMAR1	0.000359	0.251983	0.112	0.057	1	Treg	SIGMAR1
MED21	0.000374	0.258939	0.102	0.05	1	Treg	MED21
MRPS2	0.000374	0.274294	0.146	0.083	1	Treg	MRPS2
MAGOHB:	0.000378	0.301405	0.117	0.061	1	Treg	MAGOHB
GIMAP15	0.000392	0.417045	0.485	0.412	1	Treg	GIMAP1
SERINC11	0.000402	0.356512	0.33	0.25	1	Treg	SERINC1
LMAN15	0.00042	0.276255	0.228	0.151	1	Treg	LMAN1
GLS5	0.000445	0.331904	0.223	0.148	1	Treg	GLS
CDKN2AIP	0.000483	0.345264	0.141	0.079	1	Treg	CDKN2AIP
ZNF911	0.000535	0.255176	0.131	0.072	1	Treg	ZNF91
HIST1H1D	0.000543	0.528706	0.209	0.135	1	Treg	HIST1H1D
TYW1	0.00055	0.266469	0.107	0.055	1	Treg	TYW1
EXOC4	0.000556	0.351266	0.18	0.112	1	Treg	EXOC4
EIF4E4	0.000583	0.316222	0.209	0.137	1	Treg	EIF4E
ARID5A3	0.0006	0.341332	0.155	0.092	1	Treg	ARID5A
THAP72	0.000605	0.284141	0.141	0.081	1	Treg	THAP7
GORASP2	0.000609	0.325192	0.199	0.131	1	Treg	GORASP2
TRAPPC6A	0.000632	0.396536	0.223	0.153	1	Treg	TRAPPC6A
ZMYM6NE	0.000704	0.367916	0.209	0.138	1	Treg	ZMYM6NB
PTPN73	0.000706	0.280318	0.126	0.069	1	Treg	PTPN7

BATF2	0.000714	0.26926	0.18	0.112	1	Treg	BATF
FRA10AC1	0.000743	0.252264	0.155	0.093	1	Treg	FRA10AC1
VBP11	0.00076	0.291657	0.209	0.139	1	Treg	VBP1
ICAM34	0.000761	0.256116	0.49	0.404	1	Treg	ICAM3
ZBTB241	0.000833	0.275741	0.102	0.052	1	Treg	ZBTB24
TPR	0.00086	0.292436	0.427	0.349	1	Treg	TPR
ACP14	0.00088	0.33053	0.316	0.241	1	Treg	ACP1
ZRANB25	0.00089	0.326044	0.35	0.28	1	Treg	ZRANB2
C15orf611	0.000896	0.43258	0.18	0.116	1	Treg	C15orf61
ZNF83	0.000902	0.304477	0.102	0.053	1	Treg	ZNF83
PIM3	0.000914	0.340795	0.17	0.106	1	Treg	PIM3
SIGIRR4	0.000933	0.370853	0.345	0.27	1	Treg	SIGIRR
EIF4B5	0.000935	0.329995	0.51	0.448	1	Treg	EIF4B
NAE13	0.000958	0.37022	0.121	0.067	1	Treg	NAE1
CDKN2AIF	0.000959	0.250925	0.102	0.053	1	Treg	CDKN2AIPNL
BRWD11	0.001051	0.377959	0.218	0.151	1	Treg	BRWD1
DENND1C	0.00108	0.447254	0.18	0.115	1	Treg	DENND1C
CHORDC1	0.001095	0.25698	0.18	0.115	1	Treg	CHORDC1
C1QBP7	0.001185	0.323479	0.364	0.29	1	Treg	C1QBP
CYFIP28	0.001186	0.352718	0.243	0.172	1	Treg	CYFIP2
HNRNPF4	0.001255	0.296996	0.461	0.39	1	Treg	HNRNPF
RASSF52	0.001281	0.341377	0.291	0.221	1	Treg	RASSF5
VPS515	0.001333	0.37511	0.277	0.208	1	Treg	VPS51
MAP4K16	0.001423	0.295068	0.131	0.076	1	Treg	MAP4K1
FAM162A7	0.00148	0.400665	0.204	0.143	1	Treg	FAM162A
ILF3-AS12	0.001486	0.270358	0.184	0.121	1	Treg	ILF3-AS1
FBXO21	0.001566	0.30241	0.131	0.077	1	Treg	FBXO21
HSPE18	0.001603	0.509323	0.316	0.249	1	Treg	HSPE1
CCNG11	0.001629	0.288165	0.209	0.144	1	Treg	CCNG1
METTL234	0.001754	0.294776	0.184	0.123	1	Treg	METTL23
EIF3D5	0.001811	0.317137	0.393	0.328	1	Treg	EIF3D
CCT78	0.001842	0.308124	0.301	0.237	1	Treg	CCT7
RPA32	0.001882	0.444031	0.18	0.119	1	Treg	RPA3
TRIM69	0.001894	0.339387	0.126	0.074	1	Treg	TRIM69
ELOVL53	0.00193	0.332208	0.257	0.189	1	Treg	ELOVL5
RFTN13	0.002046	0.288468	0.17	0.11	1	Treg	RFTN1
NHP2L15	0.002316	0.257826	0.417	0.359	1	Treg	NHP2L1
FYN2	0.002417	0.300497	0.34	0.269	1	Treg	FYN
GTF3A5	0.002479	0.359973	0.408	0.349	1	Treg	GTF3A
SSB5	0.00248	0.403469	0.364	0.302	1	Treg	SSB
ANKHD1	0.002537	0.349882	0.17	0.111	1	Treg	ANKHD1
PDCD22	0.002745	0.25818	0.257	0.193	1	Treg	PDCD2
CACYBP5	0.002953	0.402507	0.282	0.219	1	Treg	CACYBP
LINC0049:	0.002961	0.298343	0.374	0.314	1	Treg	LINC00493
ARHGEF34	0.002961	0.254593	0.214	0.151	1	Treg	ARHGEF3
RBM45	0.002964	0.257734	0.257	0.192	1	Treg	RBM4
CCT47	0.002975	0.350194	0.335	0.274	1	Treg	CCT4
AL592284.	0.003007	0.251964	0.131	0.079	1	Treg	AL592284.1
RANGRF1	0.003403	0.309159	0.15	0.098	1	Treg	RANGRF
RPA25	0.003533	0.318863	0.199	0.141	1	Treg	RPA2
CORO1B2	0.003585	0.485337	0.277	0.224	1	Treg	CORO1B
MAPK1IP1	0.003634	0.290317	0.238	0.179	1	Treg	MAPK1IP1L
BCAS22	0.003801	0.256291	0.16	0.106	1	Treg	BCAS2
LEO1	0.00383	0.280757	0.117	0.069	1	Treg	LEO1
TMEM134:	0.003867	0.289268	0.184	0.127	1	Treg	TMEM134
MED41	0.003942	0.340448	0.238	0.179	1	Treg	MED4
DNAJC196	0.004048	0.377077	0.214	0.158	1	Treg	DNAJC19
DNPH14	0.004155	0.258774	0.189	0.132	1	Treg	DNPH1

SNRPF6	0.004234	0.26084	0.354	0.294	1	Treg	SNRPF
CCDC90B1	0.004281	0.309356	0.184	0.128	1	Treg	CCDC90B
SNRNP403	0.004428	0.255777	0.194	0.136	1	Treg	SNRNP40
SKP14	0.004458	0.28179	0.631	0.574	1	Treg	SKP1
C6orf484	0.004595	0.275669	0.199	0.143	1	Treg	C6orf48
PMPCB2	0.00465	0.279596	0.233	0.175	1	Treg	PMPCB
APBB1IP3	0.00471	0.272294	0.49	0.434	1	Treg	APBB1IP
LIX1L	0.004792	0.269973	0.131	0.083	1	Treg	LIX1L
ADH54	0.004811	0.285796	0.243	0.184	1	Treg	ADH5
CASP82	0.004983	0.297901	0.218	0.159	1	Treg	CASP8
TAF91	0.005124	0.342901	0.262	0.205	1	Treg	TAF9
MRPL42	0.005303	0.285441	0.117	0.07	1	Treg	MRPL42
CHURC11	0.005812	0.319504	0.422	0.376	1	Treg	CHURC1
B4GALT31	0.005904	0.269895	0.141	0.09	1	Treg	B4GALT3
TOPORS-A	0.00606	0.28661	0.18	0.125	1	Treg	TOPORS-AS1
DUSP114	0.006371	0.25034	0.136	0.088	1	Treg	DUSP11
CCDC821	0.00647	0.256871	0.155	0.103	1	Treg	CCDC82
PPP1CC1	0.006613	0.342149	0.345	0.291	1	Treg	PPP1CC
FDFT14	0.006678	0.306085	0.277	0.218	1	Treg	FDFT1
DHPS	0.007418	0.30884	0.218	0.164	1	Treg	DHPS
TBC1D10A	0.007474	0.276114	0.126	0.081	1	Treg	TBC1D10A
KIAA15513	0.007613	0.721697	0.354	0.306	1	Treg	KIAA1551
TMC64	0.00762	0.339922	0.218	0.163	1	Treg	TMC6
LMF21	0.007661	0.311777	0.146	0.096	1	Treg	LMF2
SRSF10	0.00773	0.380751	0.117	0.073	1	Treg	SRSF10
ASXL1	0.007752	0.265326	0.155	0.104	1	Treg	ASXL1
PHB27	0.007814	0.38326	0.388	0.35	1	Treg	PHB2
DBP3	0.007916	0.272759	0.141	0.094	1	Treg	DBP
C15orf40	0.008011	0.33942	0.16	0.11	1	Treg	C15orf40
ARHGEF13	0.008375	0.285029	0.369	0.316	1	Treg	ARHGEF1
TATDN11	0.008648	0.302118	0.146	0.099	1	Treg	TATDN1
MAGOH2	0.009032	0.405387	0.286	0.239	1	Treg	MAGOH
TMEM63A	0.009217	0.277169	0.112	0.069	1	Treg	TMEM63A
TMEM208	0.009407	0.290905	0.092	0.166	1	Treg	TMEM208
ZBTB202	0.009773	0.266245	0.102	0.061	1	Treg	ZBTB20
NUDT16L1	0.009908	0.337391	0.15	0.102	1	Treg	NUDT16L1
